# Epigenetic-Based Regulation of Transcriptome in Escherichia coli Adaptive Antibiotic Resistance

**DOI:** 10.1128/spectrum.04583-22

**Published:** 2023-05-15

**Authors:** Patrizia D’Aquila, Francesco De Rango, Ersilia Paparazzo, Giuseppe Passarino, Dina Bellizzi

**Affiliations:** a Department of Biology, Ecology and Earth Sciences, University of Calabria, Rende, Italy; Ludwig-Maximilians-Universitat Munchen Pettenkofer Institute

**Keywords:** DNA methylation, *Escherichia coli*, RNA methylation, transcriptome, antibiotic resistance

## Abstract

Adaptive antibiotic resistance is a transient metabolic adaptation of bacteria limiting their sensitivity to low, progressively increased, concentrations of antibiotics. Unlike innate and acquired resistance, adaptive resistance is dependent on the presence of antibiotics, and it disappears when the triggering factor is removed. Low concentrations of antibiotics are largely diffused in natural environments, in the food industry or in certain body compartments of humans when used therapeutically, or in animals when used for growth promotion. However, molecular mechanisms underlying this phenomenon are still poorly characterized. Here, we present experiments suggesting that epigenetic modifications, triggered by low concentrations of ampicillin, gentamicin, and ciprofloxacin, may modulate the sensitivity of bacteria to antibiotics. The epigenetic modifications we observed were paralleled by modifications of the expression pattern of many genes, including some of those that have been found mutated in strains with permanent antibiotic resistance. As the use of low concentrations of antibiotics is spreading in different contexts, our findings may suggest new targets and strategies to avoid adaptive antibiotic resistance. This might be very important as, in the long run, this transient adaptation may increase the chance, allowing the survival and the flourishing of bacteria populations, of the onset of mutations leading to stable resistance.

**IMPORTANCE** In this study, we characterized the modifications of epigenetic marks and of the whole transcriptome in the adaptive response of Escherichia coli cells to low concentrations of ampicillin, gentamicin, and ciprofloxacin. As the transient adaptation does increase the chance of permanent resistance, possibly allowing the survival and flourishing of bacteria populations where casual mutations providing resistance may give an immediate advantage, the importance of this study is not only in the identification of possible molecular mechanisms underlying adaptive resistance to antibiotics, but also in suggesting new strategies to avoid adaptation.

## INTRODUCTION

Adaptive resistance to antibiotics refers to the temporary increase in the ability of bacteria to survive an antibiotic attack, as seen in a variety of species, including Escherichia coli, Salmonella enterica, Staphylococcus aureus, and Pseudomonas aeruginosa (1–3). Unlike intrinsic and acquired resistance, in adaptive resistance a proportion of about 95% of the bacterial population becomes susceptible again in a few generations after the triggering factor is removed (4, 5). The phenomenon stems from the observation that microorganisms cultured with subinhibitory antibiotic concentration as well as concentration gradients exhibit great resistance to this molecule and other drugs of the same or different classes. It has been suggested that the ability of bacteria to proliferate under such conditions through adaptive resistance may allow the development of more effective and durable resistance mechanisms (6, 7). The rates of increase and duration of persistence after removal of the antibiotic are bacteria-specific and correlate with the type of antibiotic, dose, and exposure time. Considering that drug-resistant phenotype in adaptive resistance cannot be readily detected by a clinical microbiology laboratory, it is difficult to establish a drug regimen. The development of adaptive resistance has been documented for several antibiotic families, including β-lactams, aminoglycosides, fluoroquinolones, and polymyxins (8–10). Sandoval-Motta and Aldana propose that adaptive resistance is the conjunction of two types of mechanisms: the fast and transient mechanism (FTM), occurring rapidly and causing cumulative phenotypic changes and contributing to the heterogeneity observed in the bacterial population, and the slow and stable mechanism (SSM), which lasts longer and is responsible for a durable response and an irreversible phenotype after long-term exposure to antibiotics (6, 11). Generation of reactive oxygen species (ROS), SOS response, changes in the production of efflux pumps, activation of antibiotic-degrading enzymes, and biofilm formation have been demonstrated in a few studies as the underlying mechanisms involved in the adaptive response (3, 12–16). More recently, epigenetic inheritance mediated by DNA methylation, nucleoid modifications, and superhelical domain configuration was hypothesized as a possible explanation for adaptive resistance. More specifically, DNA methylation may provide a mechanism for the observed metastable resistance (17–19). The DNA of E. coli contains 19,120 6-methyladenines and 12,045 5-methylcytosines (20, 21). Three DNA methyltransferases have been described in E. coli K-12. DNA adenine methyltransferase (Dam) was the first orphan DNA methyltransferase characterized. It methylates the N (6) position of the adenine in the palindromic 5′-GATC-3′ motif (22, 23). DNA cytosine methyltransferase (Dcm) is an orphan cytosine methyltransferase that methylates the C (5) position of the second cytosine in the 5′-CC(A/T)GG-3′ motif (24). DNA methyltransferase M (HsdM) is an adenine methyltransferase belonging to an R-M system (21). Deletion of *dam*, *dcm*, and *hsdM* in E. coli K-12 determines a complete lack of methylation on the genome, thus demonstrating that the three enzymes are the only active methyltransferases in that strain (22).

In addition, more than 160 RNA modifications have been identified to date since the first-modified RNA nucleoside discovered by Cohn and Volkin in 1951 (25, 26). These nucleotides derivate vary in complexity, including methylation, cyclization, and glycosylation, thus forming the so-called epitranscriptome (27). In E. coli, 36 modified sites were found on rRNA, 11 on the small subunit 16S rRNA and 25 on the large subunit 23S rRNA. They have been implicated in proper ribosome biogenesis, assisting ribosome stability, interacting with ribosomal ligands such as transfer RNAs (tRNAs). It was demonstrated that demethylation of 23S rRNA residue A2058 by macrolides confers antibiotic resistance and alters the translatome by allowing the synthesis of certain proteins (28).

Given the importance of antibiotic resistance in modern medicine, and of resistance to low levels of antibiotics, to elucidate this phenomenon, we selected resistant cells of E. coli JM109 by growing the strain at low concentrations of ampicillin, gentamicin, and ciprofloxacin. Global profiles of cytosine and adenine methylation in the DNA genome, adenine methylation in RNA molecules and whole-genome transcriptome were obtained for the control strain and the resistant cell lines. Differentially expressed genes were subjected to functional enrichment, pathway, and protein-protein interaction network analyses thus allowing the identification of key genes involved in the adaptive response to the three antibiotics.

## RESULTS

### Antibiotic-resistant cells isolation.

Three antibiotics, namely, ampicillin, gentamicin, and ciprofloxacin, which belong to different antibiotic classes and have different mechanisms of action, were selected for the study. To determine the experimental conditions for isolation of antibiotic-resistant cells, we cultured E. coli JM109 cells with increasing concentrations of the three antibiotics and measured the MIC values for each antibiotic. Under our laboratory conditions, the MIC was 12 μg/mL for ampicillin, 0.4 μg/mL for gentamicin, and 2 ng/mL for ciprofloxacin. Therefore, we selected resistant cells from a susceptible population following in detail the protocol described in Gullberg et al. (2011) (7). To this end, we grew E. coli JM109 control strain for 700 generations at sub-MICs and continuously screened for resistant cells by exposing them to different concentrations of ampicillin, gentamicin, and ciprofloxacin (6, 7). We selected ampicillin-resistant cells with MIC about 4-folds higher (50 μg/mL), gentamicin-resistant cells with MIC 5-folds higher (2 μg/mL), and ciprofloxacin-resistant cells with MIC 10-folds higher (20 ng/mL) than that of the starting strain. These cells, cultured first in the absence and then in the presence of the three antibiotics, have not grown thus suggesting that the resistance phenotype is transient and, therefore, not due to mutations.

### Analysis of global DNA and RNA methylation profiles.

**FIG 1:**
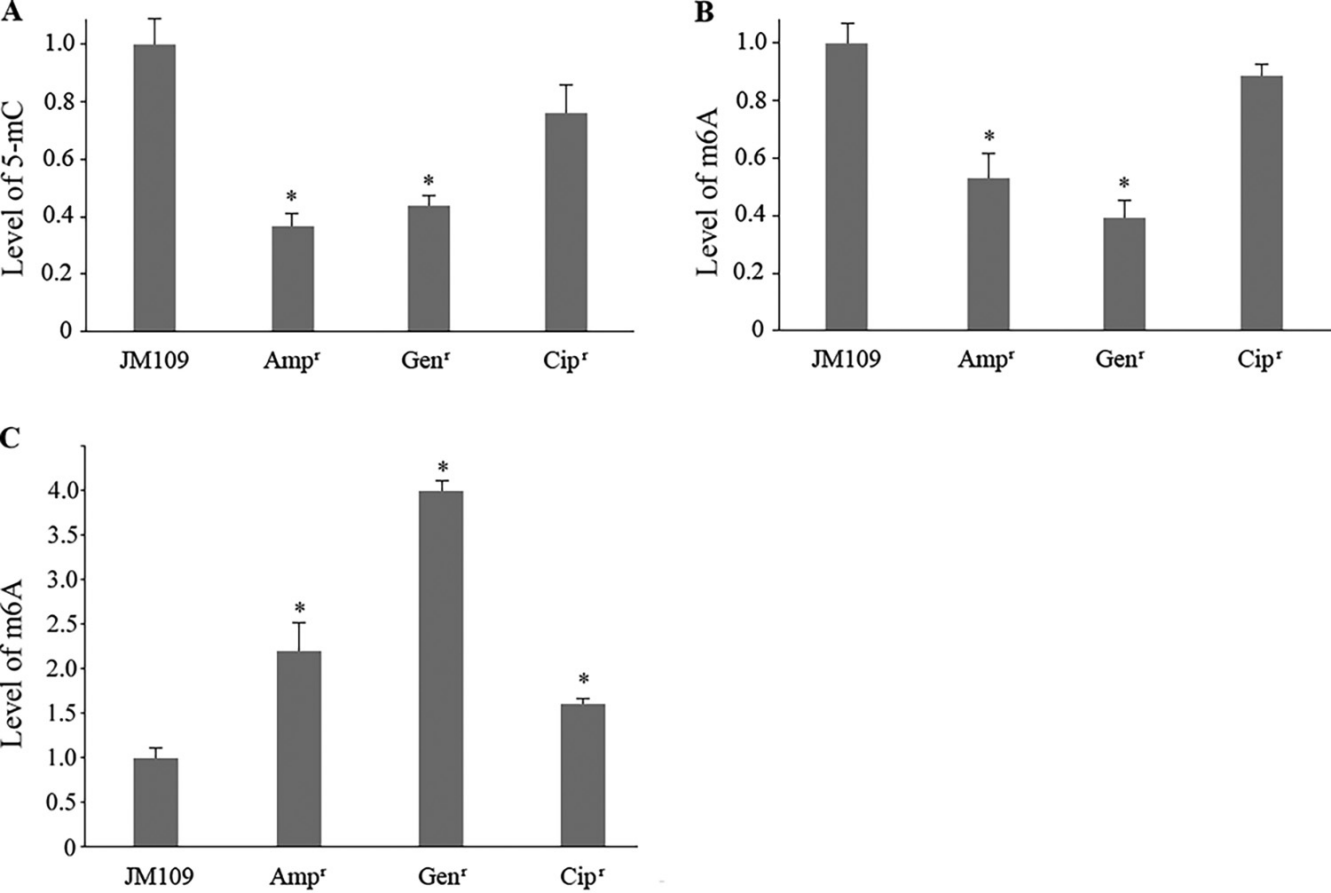
Global DNA methylation levels in DNA and RNA of JM109 control strain and antibiotic-resistant cells. Levels of 5-methylcytosine (5-mC) (A) and N6-methyladenosine (m6A) (B) residues located in genomic DNA of the JM109 control strain and ampicillin-(Amp^r^), gentamicin-(Gen^r^), and ciprofloxacin-(Cip^r^) resistant cells. (C) Global methylation levels of N6-methyladenosine (m6A) residues located in RNA molecules are reported. The values represent the mean of three independent triplicate experiments with standard deviation. *, *P* value < 0.01.

Levels of 5-methylcytosine (5-mC) and N6-methyladenosine (m6A) were determined by ELISAs in DNA samples extracted from both the control strain and ampicillin-, gentamicin-, and ciprofloxacin-resistant cells. The effectiveness of the experimental conditions was confirmed by the correlation values of 0.9986 (5-mC) and 0.9559 (m6A) of two standard curves obtained by plotting the absorbance values of a mixture of equivalent molar concentration of unmethylated and methylated control samples against the percentage of 5-mC and m6A values. Fig. 1 shows that the global methylation levels of both cytosines (A) and adenines (B) are lower in the ampicillin- and gentamicin-resistant cells than in the JM109 control strain. As for ciprofloxacin-resistant cells, their methylation levels are lower than those of the JM109 strain, but such difference is not statistically significant.

Global m6A methylation levels were also quantified by ELISA in total RNA samples extracted from both the control strain and the ampicillin-, gentamicin-, and ciprofloxacin-resistant cells. A logarithmic relationship of the standard curve obtained by plotting the absorbance values of a mixture of equivalent molar concentration of unmethylated and methylated control samples against the percentage of m6A values was found with a correlation of 0.975, confirming the efficacy of the experimental conditions. In all three antibiotic-resistant cell lines, the methylation levels of m6A are higher than in JM109 cells (Fig. 1C).

Therefore, the adaptive response of the cells to the tested antibiotics appears to be related to a global remodeling in methylation levels at both DNA and RNA levels.

### Whole-transcriptome analysis.

**FIG 2:**
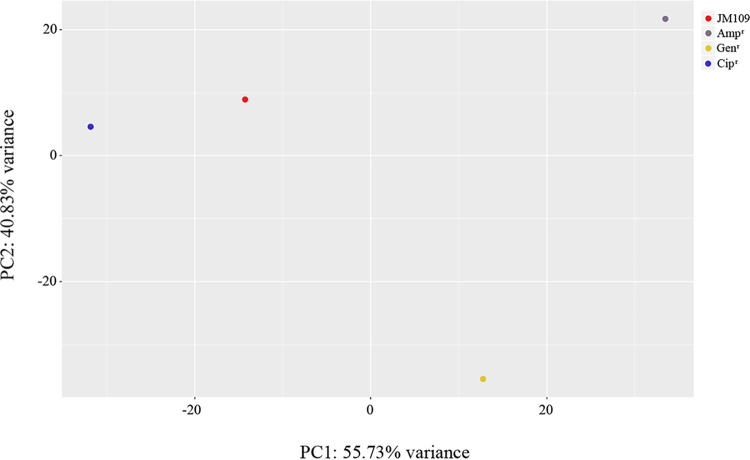
Principal component analysis (PCA) for the JM109 control strain and antibiotic-resistant cells. The axis labels show that PC1 explains 55.73% of the total variance, and PC2 explains 40.83%. Red, gray, yellow, and blue dots represent JM109 control strain, ampicillin-(Amp^r^), gentamicin-(Gen^r^), and ciprofloxacin-(Cip^r^) resistant cells, respectively.

To investigate the molecular basis of adaptive resistance to the three antibiotics, next-generation sequencing of total RNA of the JM109 control strain (untreated) and ampicillin-, gentamicin-, and ciprofloxacin-resistant cells was performed. We submitted the RNA-seq data to gene expression omnibus (GEO)-NCBI. The accession number is GSE228373. To visually represent differences between the four cell lines, we carried out a principal component analysis (PCA) using normalized gene expression values of all genes (4,236). Results are presented in a scoring diagram in which strongly correlated data are grouped together, while samples with different values are further apart (Fig. 2). PCA revealed that ciprofloxacin-resistant cells in both PC1 and PC2 share more similarities with the JM109 control strain, whereas gentamicin-resistant cells are separated from the other two populations in both PC1 and PC2, and ampicillin-resistant cells are separated mainly in PC1. This suggests an overall difference between the transcriptomic profiles of the gentamicin- and ampicillin-resistant cells and the JM109 control strain. In contrast, there is a high similarity in overall gene expression between ciprofloxacin-resistant cells and the control JM109 strain.

We averaged gene expression values across replicates for each cell line after normalization and compared each antibiotic-resistant cell to the JM109 control strain by calculating fold change (FC) as the ratio between the average values. With a significance threshold of log_2_(FC) > 1 and a *P* value < 0.05, we found 561 genes with altered expression levels in ampicillin-resistant cells, 735 genes in gentamicin-resistant cells, and 46 genes in ciprofloxacin-resistant cells compared with JM109 control strain. The complete list of upregulated and downregulated genes can be found in Tables S1, S2, and S3.

**FIG 3:**
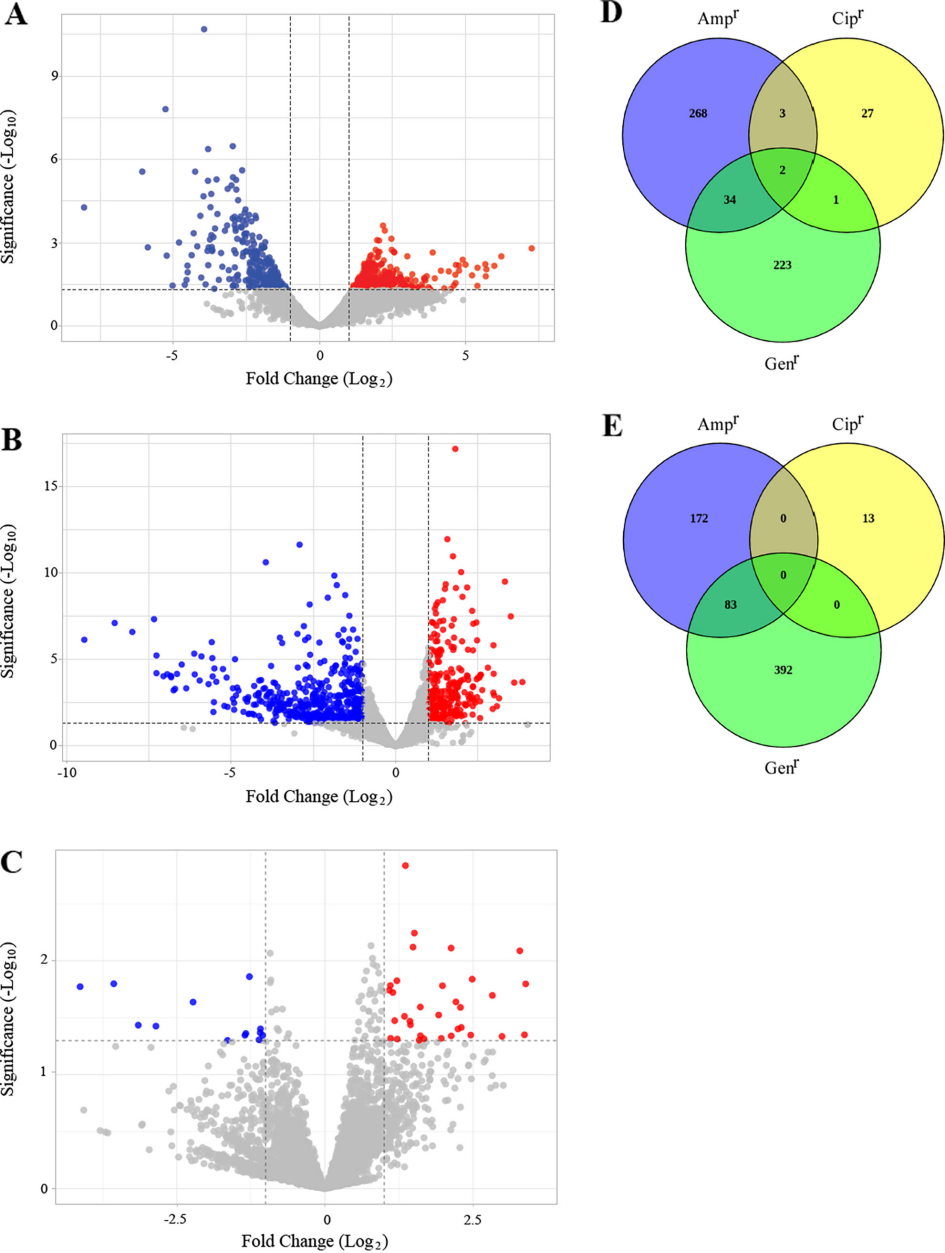
Volcano plot of differentially expressed genes. Red and blue dots represent upregulated and downregulated genes, respectively, in ampicillin- (A), gentamicin- (B), and ciprofloxacin-resistant (C) cells. Gray dots represent not differentially expressed genes. Venn diagrams show the number of up-expressed genes (D) and down-expressed genes (E) overlapping between the groups. Blue, green, and yellow areas represent the genes in ampicillin-(Amp^r^), gentamicin-(Gen^r^), and ciprofloxacin-(Cip^r^) resistant cells, respectively.

Notably, the volcano diagram shows that many more genes were upregulated (307) than downregulated (255) in the ampicillin-resistant cells compared with the JM109 control strain (Fig. 3A), whereas the opposite is true in the gentamicin-resistant cells (260 upregulated and 475 downregulated genes) (Fig. 3B). In the ciprofloxacin-resistant cells, 33 upregulated and 13 downregulated genes were observed. Common genes in the three cell lines were shown in Venn diagrams, where we observed that in the case of both upregulated (Fig. 3D) and downregulated (Fig. 3E) genes, the highest number of genes is shared by the ampicillin- and gentamicin-resistant cells, thus leading us to hypothesize that they are involved in resistance to multiple antibiotics.

### Functional enrichment analysis.

Functional annotation of all those upregulated or downregulated genes detected in the three antibiotic-resistant cells was carried out through the gene ontology (GO) tool. To establish the potential GO classification, terms into biological processes, molecular functions, and cellular components were used (Table S4, Table S5, Table S6, and Table S7). In Table 1, we report the 10 most enriched biological processes being the most informative about the significance of the alterations in gene expression levels. Among the upregulated genes in ampicillin-resistant cells, we can observe that processes range from regulation of the developmental process, cell, and anatomical structure morphogenesis to different biosynthetic processes. Among the downregulated genes in ampicillin-resistant cells, biological processes include nitrogen and organic compound metabolism, establishment of localization, transport, and metabolic processes. Among the upregulated genes in gentamicin-resistant cells, biological processes include organic phosphonate metabolism, siderophore-dependent iron import into cell, and primary alcohol metabolism. Among the downregulated genes in gentamicin-resistant cells, we observe that all biological processes include functions correlated with structure and function of ribosomes. In regard to up- and downregulated genes in ciprofloxacin resistant-cells, no functional enrichment was observed.

**TABLE 1:** Top 10 enriched biological process GO terms of the differently expressed genes in the ampicillin- and gentamicin-resistant cells

Ampicillin up-regulated genes	GO:0006567 Threonine catabolic process GO:0050793 Regulation of developmental process GO:0022604 Regulation of cell morphogenesis GO:0022603 Regulation of anatomical structure morphogenesis GO:0008360 Regulation of cell shape GO:0006024 Glycosaminoglican biosynthetic process GO:0006023 Aminoglycan biosynthetic process GO:0009252 Peptidoglycan biosynthetic process GO:0044038 Cell wall macromolecule biosynthetic process GO:0070589 Cellular component macromolecule biosynthetic process
Ampicillin down-regulated genes	GO:0008150 Biological processes GO:0009987 Cellular process GO:0006807 Nitrogen compound metabolic process GO:0044237 Cellular metabolic process GO:0008152 Metabolic process GO:0044238 Primary metabolic process GO:0071704 Organic substance metabolic process GO:0051234 Establishment of localization GO:0006810 Transport GO:1901564 Organonitrogen compound metabolic process
Gentamicin up-regulated genes	GO:0019700 Organic phosphonate catabolic process GO:0019634 Organic phosphonate metabolic process GO:0033214 Siderophore dependent iron import into cell GO:0034310 Primary alcohol catabolic process GO:0033212 Iron import into cell GO:0034308 Primary alcohol metabolic process GO:1901678 Iron coordination entity transport GO:0000041 Transition metal ion transport GO:0006826 Iron ion transport GO:0030001 Metal ion transport
Gentamicin down-regulated genes	GO:0000028 Ribosomal small subunit assembly GO:0071826 Ribonucleoprotein complex subunit organization GO:0022618 Ribonucleoprotein complex assembly GO:0000027 Ribosomal large subunit assembly GO:0042274 Ribosomal small subunit biogenesis GO:0042273 Ribosomal large subunit biogenesis GO:0042255 Ribosome assembly GO:0034249 Negative regulation of cellular amide metabolic process GO:2000113 Negative regulation of cellular macromolecule biosynthetic process GO:0017148 Negative regulation of translation

### KEGG pathway analysis.

**FIG 4:**
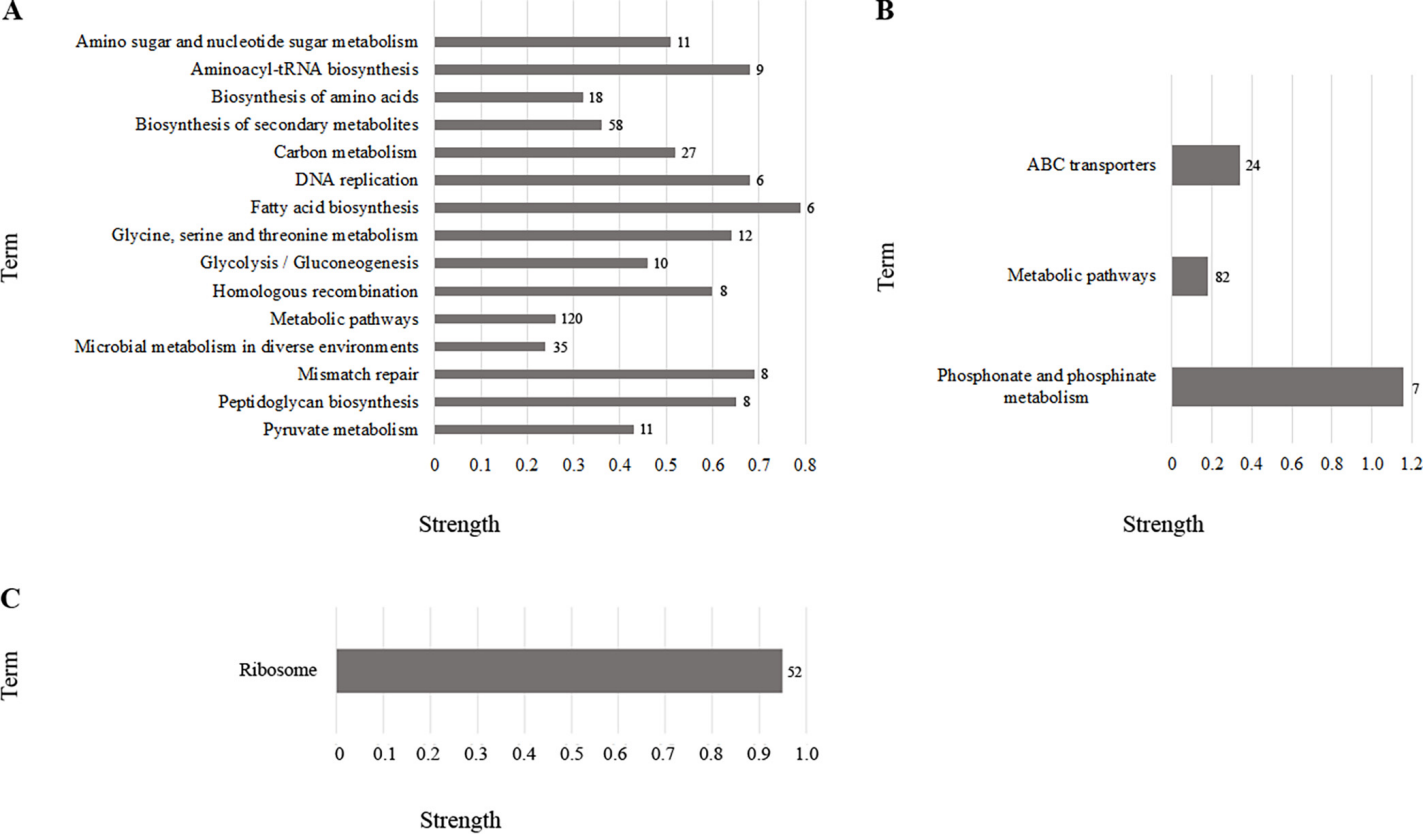
KEGG pathway analysis of the differently expressed genes in the antibiotic-resistant cells. The pathways relative to up-expressed genes in ampicillin-resistant cells (A), up-expressed genes in gentamicin-resistant cells (B), and down-expressed genes in gentamicin-resistant cells (C) are reported. Strength is the Log_10_ (observed/expected) namely, the ratio between the number of proteins observed in the network of our study annotated with a term and the number of proteins expected to be annotated with the term in a random network of the same size. FDR < 0.05 was considered. To the right of each bar graph, the number of genes from each pathway is indicated.

To identify the relevant biological functions and enriched signaling pathways of the differentially expressed genes, KEGG analyses were performed by using the STRING tool. The identified pathways in the three antibiotic-resistant cells are reported in detail in Fig. 4 and Table S8. In regard to upregulated genes in ampicillin-resistant cells, the main pathways may be related to survival or recovery, such as mismatch repair, peptidoglycan biosynthesis, homologous recombination, and DNA replication beyond those involved in different metabolic pathways (Fig. 4A).

In regard to upregulated genes in gentamicin-resistant cells, phosphonate and phosphinate metabolism, metabolic pathways, and ABC transporters were identified (Fig. 4B).

Lastly, in regard to downregulated genes in gentamicin-resistant cells, the sole pathway we observed was correlated with ribosome (Fig. 4C). Regarding downregulated genes in ampicillin-resistant cells, up- and downregulated genes in ciprofloxacin resistant-cells, no statistically significant results were observed.

### Protein-protein interaction network analysis.

We used STRING to construct the protein-protein interaction (PPI) network associated with upregulated and downregulated genes detected in the three antibiotic-resistant cells. The minimum required interaction score was set to the confidence of 0.7.

**FIG 5:**
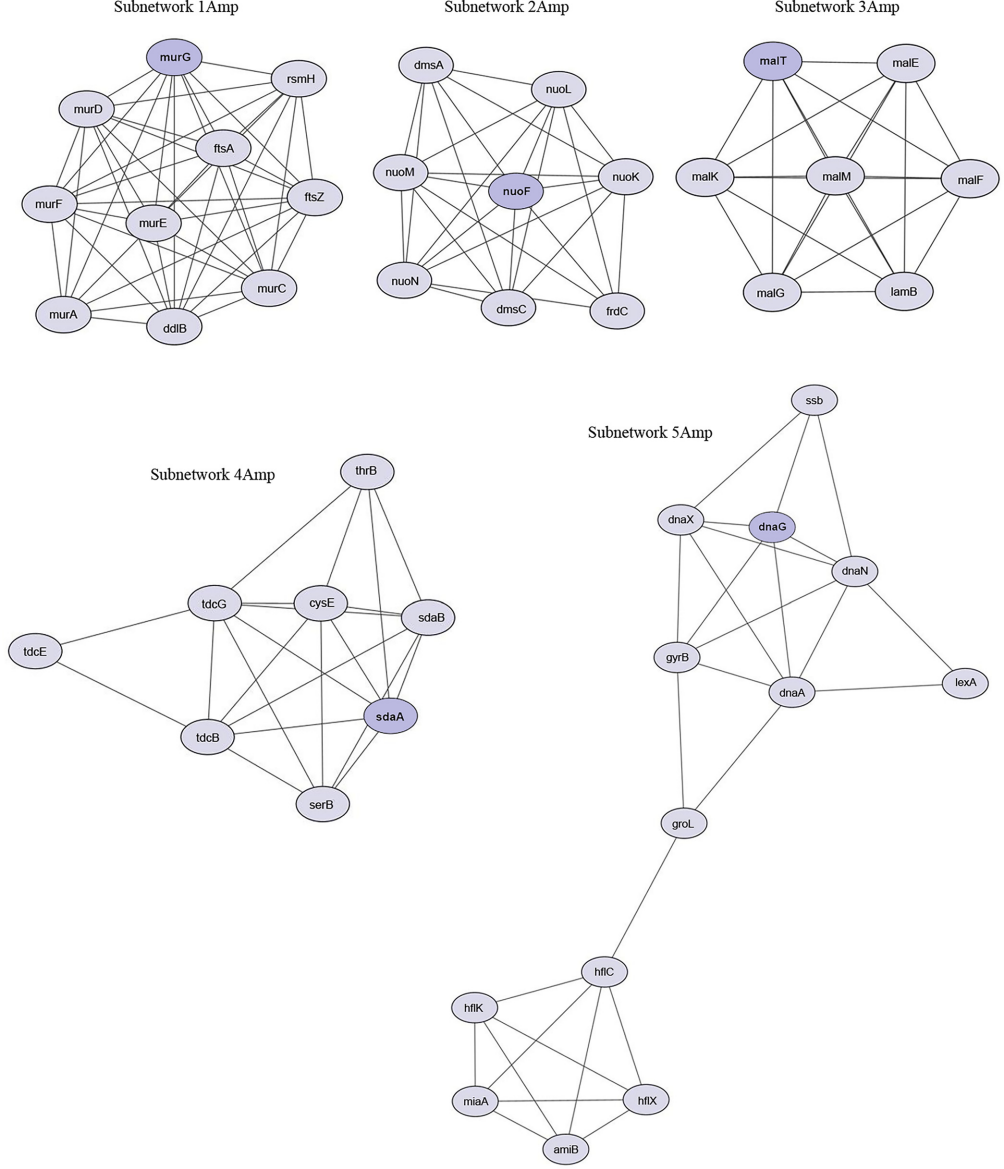
Protein-protein interaction (PPI) subnetworks of upregulated genes in ampicillin-resistant cells. The best five subnetworks, constructed using STRING and visualized in Cytoscape, are reported. Nodes and edges represent protein and protein-protein association, respectively. The network from which the subnetworks were extracted is shown in Fig. S1A.

The PPI network, constructed by using the 307 upregulated genes in ampicillin-resistant cells, consisted of 307 nodes and 685 edges (Fig. S1A; Table S9). The clusters of subnetworks were analyzed and visualized by MCODE in Cytoscape. Subnetwork 1Amp was derived from node *murG* (Undecaprenyl-PP-MurNAc-pentapeptide-UDPGlcNAc GlcNAc transferase) and it is composed by 10 nodes and 43 edges, subnetwork 2Amp was derived from node *nuoF* (NADH-quinone oxidoreductase subunit F) and it is composed of eight nodes and 26 edges, subnetwork 3Amp was derived from *malT* (ATP-dependent transcriptional activator MalT) and it is composed of seven nodes and 21 edges, subnetwork 4Amp was derived from *sdaA* (l-serine dehydratase 1) and it is composed of eight nodes and 21 edges, and subnetwork 5Amp was derived from *dnaG* (DNA primase) and it is composed of 13 nodes and 28 edges (Fig. 5).

**FIG 6:**
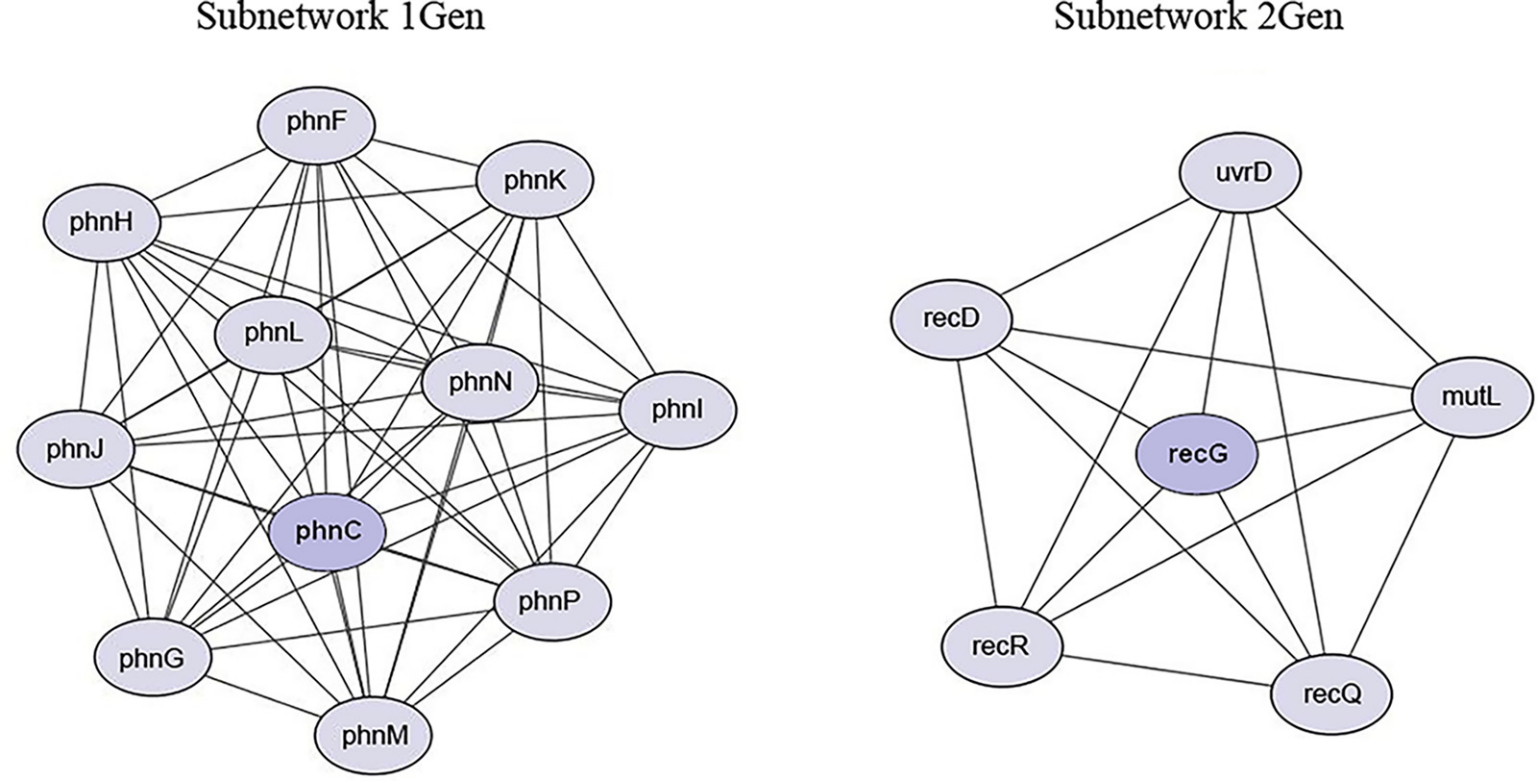
Protein-protein interaction (PPI) subnetworks of upregulated genes in gentamicin-resistant cells. The best two subnetworks, constructed using STRING and visualized in Cytoscape, are reported. Nodes and edges represent protein and protein-protein association, respectively. The network from which the subnetworks were extracted is shown in Fig. S1B.

The PPI network, using the 260 upregulated genes in gentamicin-resistant cells, formed a network consisting of 252 nodes and 311 edges (Fig. S1B; Table S9). Subnetwork 1Gen was derived from node *phnC* (Phosphonates import ATP-binding protein PhnC) and it is composed of 11 nodes and 54 edges, and subnetwork 2Gen was derived from node *recG* (ATP-dependent DNA helicase RecG) and it is composed of six nodes and 15 edges (Fig. 6).

**FIG 7:**
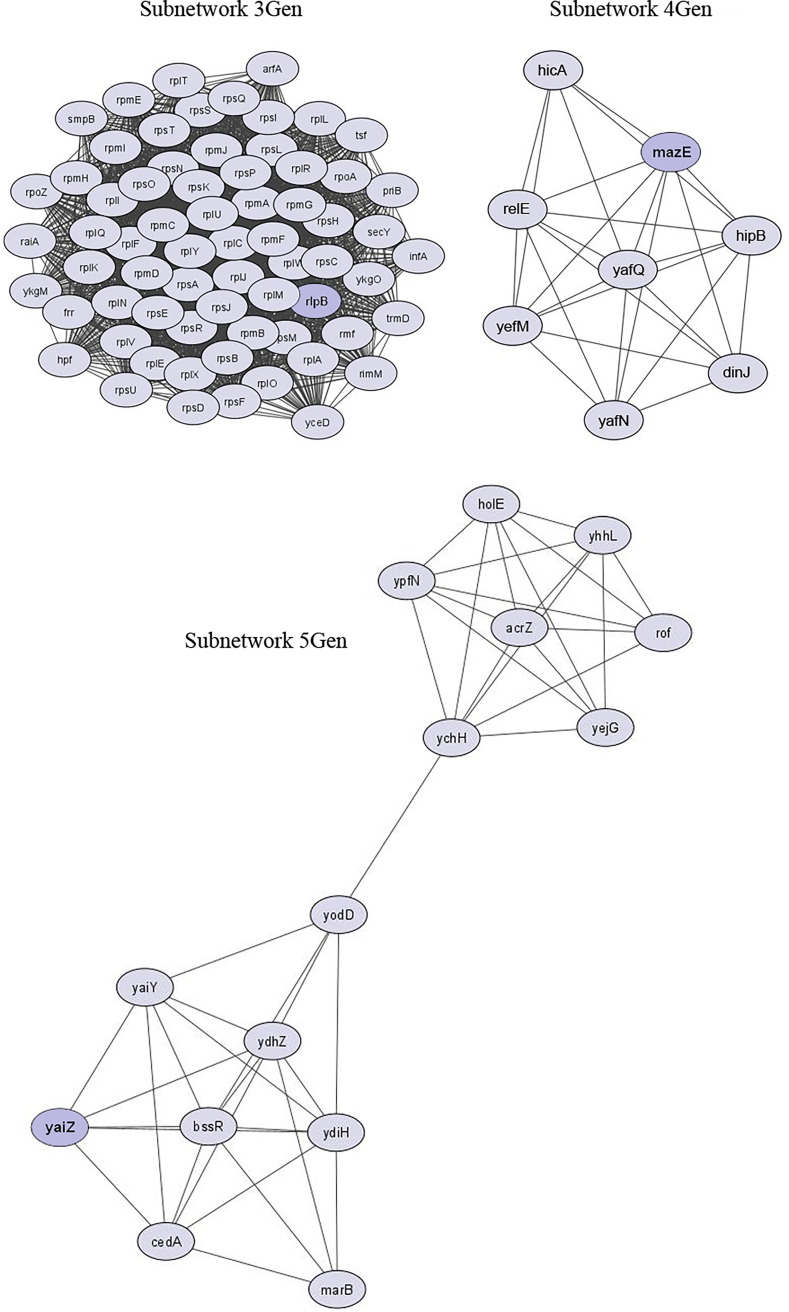
Protein-protein interaction (PPI) subnetworks of downregulated genes in gentamicin-resistant cells. The best five subnetworks, constructed using STRING and visualized in Cytoscape, are reported. Nodes and edges represent protein and protein-protein association, respectively. The network from which the subnetworks were extracted is shown in Fig. S1C.

The PPI network, using the 475 downregulated genes in gentamicin-resistant cells, formed a network consisting of 432 nodes and 2,630 edges (Fig. S1C; Table S9). Subnetwork 3Gen was derived from *rlpB* (50S ribosomal protein L2) and it is composed of 67 nodes and 2,051 edges, subnetwork 4Gen was derived from *mazE* (Antitoxin MazE) and it is composed of eight nodes and 26 edges, and subnetwork 5Gen was derived from *yaiZ* (Uncharacterized protein YaiZ) and it is composed of 15 nodes and 44 edges (Fig. 7). For each PPI network described above, the nodes with large degree, identified as hubs, are reported in detail in Table S9.

STRING analysis did not lead to the construction of any PPI network regarding the downregulated genes in ampicillin-resistant cells as well as the up- and downregulated genes in ciprofloxacin-resistant cells.

## DISCUSSION

There is growing evidence that bacteria acquire transient resistance to adapt to and overcome various stimuli, such as ion density, temperature, osmotic stress, and, most importantly, exposure to increasingly nonlethal doses of antibiotics. Unlike innate and acquired resistance, adaptive resistance is so dependent on the presence of antibiotics that it disappears when the triggering factor is removed. It should also be considered that, while the selection of resistant bacteria by high concentrations of therapeutically used antibiotics has been widely described, the role of low concentrations, such as those found in many natural environments, in the food industry or certain body compartments of humans and animals when used therapeutically or for growth promotion, remains largely understudied in the above selection. This is an issue sought in the clinical field (7, 29–33). Note that the molecular mechanisms underlying adaptive resistance at low antibiotic concentrations have not been elucidated to date.

In this study, we selected resistant E. coli cell lines by their progressive growth over 700 generations in increasing concentrations of ampicillin, gentamicin, and ciprofloxacin, three antibiotics with different modes of action and great importance in the treatment of bacterial infections in humans. As specified by Gullberg et al., cells with high resistance levels were enriched despite the low antibiotic concentrations (7). The evidence we observed that these cells become sensitive again after some generations of growth in culture media without the three antibiotics suggests that the resistance is not due to mutations in genes involved in this phenotype, which are known to confer durable and heritable resistance to cells. This is confirmed by the fact that the number of resistant cells is not as high as expected if they were due to genetic mutations (34). Thus, this suggests that epigenetic changes may maintain the phenotype of antibiotic resistance. We used an ELISA to detect and quantify the global DNA methylation of adenines and cytosines in the genome of E. coli JM109 and the three antibiotic-resistant cells. Ampicillin-, gentamicin-, and ciprofloxacin-resistant cells have lower 5-mC and m6A levels than sensitive JM109 control cells. To our knowledge, this is the first study in which global methylation levels of 5-mC and 6 mA are correlated with resistance at low antibiotic concentrations. Yugendran et al. reported a similar result, restricted to cytosine methylation, in both cultured and isolated ciprofloxacin-resistant cells (35). The results we obtained shed new light on the role of DNA methylation in bacterial microorganisms and show that, in addition to its known functions such as genome protection, chromosome replication and segregation, nucleoid organization, cell cycle control, DNA repair, and regulation of transcription, genome methylation is also involved in the phenomenon of antibiotic resistance (36, 37).

Interestingly, we have shown that RNA methylation is also associated with antibiotic-resistance but intracellular patterns exhibit an opposite trend with respect to DNA methylation. RNA methylation levels are higher in the three antibiotic-resistant strains than in the control cell line. Because epigenetic modifications in eukaryotic RNAs are known to modulate a variety of cellular processes, the hypothesis that such modifications in bacterial RNAs may be associated with critical functions that maintain the antibiotic resistance phenomenon may not be speculative (38, 39). Consistently, we observed that genes such as *trmJ* (tRNA Cm32/Um32 methyltransferase), *rlmH* (23S rRNA m3Ψ1915 methyltransferase), and *rlmB* (23S rRNA 2′-O-ribose G2251 methyltransferase) as well as *rsmJ* (16S rRNA m2G1516 methyltransferase), involved in the modification of different RNA molecules, are overexpressed in ampicillin- and gentamicin-resistant cells, respectively.

On the other hand, apart from the downregulation of *dcm* gene in the gentamicin-resistant line, *dam* and *dcm* genes, widely described to be involved in methylation of the bacterial genome, as well as *rlmJ*, *rlmF*, and *yfiC* genes, encoding methyltransferases responsible for methylation RNA molecules, show no significant changes in the antibiotic-resistant cells. We might even speculate that other, yet unidentified, enzymes may be involved in the methylation of both nucleic acids. Some of our ongoing studies are aimed at pursuing this identification also considering the increasing number of proteins with S-adenosyl methionine (SAM)-binding domain annotated in genome databases.

The above observations are of relevance not only as a basic science discovery but also for its potential integration into clinical applications, as epigenetic changes are reversible.

Because a precise functional role of the bacterial epigenome has not been previously recognized, we performed a whole-genome transcriptome study in ampicillin-, gentamicin-, and ciprofloxacin-resistant cells. Our work supports the idea that adaptive resistance to antibiotics is enabled by changes in gene expression, as indicated by the observation of the different transcriptional profiles in the three antibiotic-resistant cells. The similarity between the transcriptome profiles of ciprofloxacin-resistant cells and the sensitive JM109 control strain resulting from PCA, as well as the distance between ampicillin- and gentamicin-resistant cells and the control strain, confirm the methylation patterns and highlight how antibiotic resistance is determined by antibiotic-specific transcriptional regulation. Consistently, a few differentially expressed genes were observed in ciprofloxacin-resistant cells compared with the JM109 control cells. Therefore, data suggest that ampicillin and gentamicin have a long-range effect on E. coli with respect to ciprofloxacin and thus highly remodel the transcriptome. According to the GO enrichment analysis, the upregulated genes in ampicillin-resistant cells indicate the importance of cell morphogenesis, cell division, DNA synthesis, and peptidoglycan-based cell wall formation during adaptation to the presence of the antibiotic. This observation also emerges from the PPI network analysis, which highlights a role for the proteins MurG, associated with peptidoglycan biosynthesis, and DnaG that is the essential component in the replisome (40, 41). The identification of pathways in KEGG analysis that are implicated in DNA replication and repair highlights that all above processes are tightly linked and essential for bacterial growth and are normally affected by antibiotic activity (29). Thus, our results suggest that adaptation to ampicillin is achieved by the enrichment of processes that can ensure the replication of a bacterial genome and maintain constant shape, size, and, considering the role of the wall, intracellular homeostasis. The enrichment of GO biological processes, including carbohydrate, secondary metabolism, amino acid, pyruvate, nitrogen and keto acid metabolism, and glycolysis/gluconeogenesis, in conjunction with high expression of NADH quinone oxidoreductase genes and genes belonging to the network associated with *sdaA*, suggests that adaptive resistance, similar to that reported in previous research on acquired antibiotic resistance, requires the concerted action of many components, several of which play important roles in bacterial metabolism and energy production (42). Finally, it appears that reduced accumulation of misfolded cytoplasmic membrane proteins in response to ampicillin is induced by increased expression of genes encoding components of the HflKC complex, which contributes to the quality control system as a regulatory factor of FtsH and as a possible membrane chaperone (43). In the analysis of downregulated genes, enrichment in GO metabolic processes was also observed, leading to the assumption that these processes are strongly regulated by both up- and downregulation of specific genes. Interestingly, the observed underactivation of processes that localize substances such as carbohydrates, ascorbate, and succinate or cellular components is consistent with literature data reporting delocalization or mislocalization of several proteins in response to various drugs (44). As previously reported in studies of adaptive evolution, the motility-associated genes *fliQ*, *fliR*, and *cheW* and the curli-specific genes *csgE* and *csgF* were also underexpressed in response to ampicillin (45). It is plausible that reduced expression of the above genes is a way to conserve energy in particularly critical situations such as exposure to antibiotics.

Although processes involved in metabolic remodeling and energy production as well as DNA replication and repair were also enriched in gentamicin-resistant cells, these cells differed from ampicillin-resistant cells in some biological processes, confirming the specific effects associated with the two antibiotics. In gentamicin-resistant cells, in addition to the activation of ABC transporters known to pump antibiotics out of cells, the disruption of iron homeostasis appears to be associated with adaptive resistance to this antibiotic (46). In agreement with literature data reporting that this homeostasis alters the sensitivity of bacteria to different antibiotics, we observed that genes involved in iron homeostasis, siderophore-mediated uptake, and enterobactin biosynthesis were simultaneously upregulated with the downregulation of the *Fur* gene (47). This gene represents a key transcriptional regulator of iron homeostasis whose inactivation is involved in antibiotic resistance in E. coli (48). As previously reported in the literature, our study showed that the Nuo-, Sdh-, and Sdc-complexes, which contain numerous Fe-S clusters and are critical for generating a proton motive force that enables gentamicin uptake, are downregulated in response to gentamicin, contributing to cells becoming resistant to the antibiotic (49). Our results indicate that an event promoting adaptive resistance includes the SOS response promoted by *recA* and *lexA* genes, two upregulated genes that enable survival to otherwise lethal antibiotic exposure, the double-strand break repair, and the protection against aberrant DNA replication (50).

Finally, it is noteworthy that the most enriched functions among the underexpressed genes in gentamicin-resistant cells are all related to ribosome structure and protein synthesis (51, 52). We can hypothesize that these changes are a compensatory mechanism of the cells to render these major substrates of gentamicin useless. Interestingly, the survival of resistant cells to gentamicin also appears to be mediated by a slowdown of the programmed cell death that is triggered by several antibiotics that inhibit the continuous expression of the antitoxin MazE (53).

Difficult to refute is the high expression of genes of the maltose regulon in ampicillin-resistant cells, of which MalT is a positive regulator activated in E. coli by an inducer such as maltose that stimulates gene transcription by activating RNA polymerase (54). Similarly, the *phn* operon in gentamicin-resistant cells, which components belong to a phosphonate/phosphate transport system of the ABC type that is activated when cells enter phosphate limitation (55). The absence of maltose and phosphonates in the culture medium suggests that differently regulated genes, not yet identified, induce expression of the maltose system and the *pnc* operon in the adaptive response to antibiotics.

Overall, this study reports for the first time that epigenetic changes may represent a mechanism explaining the adaptive antibiotic resistance of bacteria. Such epigenetic changes appear antibiotic- dependent and seem to be implicated in the regulation of the expression of specific genes. Interestingly, some of these regulated genes, such as *ampC*, *ompF*, *ompC*, and *murCDEF*, have been found to be mutated in acquired and durable resistance (56–58).

Finally, note that, among those differently expressed between the three resistant cell lines, two genes, *ssb* and *yhdE*, overlap between them. The first is involved in DNA replication, recombination, and repair, and in the protection of ssDNA from degradation (59). The second is mainly involved in monitoring the ribonucleotide pool, thus preventing unspecific incorporation of modified bases into cellular RNAs, and in retarding cell growth under stress conditions (60).

It has been reported that transient resistance may favor intrinsic and acquired resistance. This may occur because the long-term exposure to low concentrations of antibiotics could allow the survival and flourishing of bacteria populations where the onset of mutations leading to resistance becomes more and more likely due to the growing number of individuals and to the selective advantage that a mutated cell would have in the presence of antibiotics. As low levels of antibiotics are largely used, this may contribute to creating larger and larger populations of bacteria resistant to these concentrations with a growing chance of mutations. On the other hand, our study has highlighted new targets to contrast antibiotic resistance that may be particularly efficient considering that epigenetic changes are reversible.

## MATERIALS AND METHODS

### E. coli cells.

E. coli strain JM109 (Stratagene) (e14–(McrA–) recA1 endA1 gyrA96 thi-1 hsdR17 (rK– mK+) supE44 relA1 Δ(lac-proAB) [F´ traD36 proAB lacIqZΔM15]) was streaked on Luria Bertani (LB) agar plate and resulting single colonies were inoculated in LB liquid, aliquots of which were frozen and later used for all experiments.

### MIC determination.

MIC assays of ampicillin, gentamicin, and ciprofloxacin were performed by broth dilution in 10 mL tubes. Approximately 10^6^ cells from an overnight LB culture were inoculated into each tube of a 2-fold serial dilution of ampicillin, gentamicin, and ciprofloxacin containing 3 mL of LB. The tubes were incubated at 37°C for 18 h to 20 h with vigorous agitation. The MIC values were determined to be the lowest concentration of antibiotic resulting in optical density (OD) reading at 600 nm comparable to those of the grown medium lacking cells (control).

### Enrichment of ampicillin-, gentamicin-, and ciprofloxacin-resistant cells.

To select for resistant cells to ampicillin, gentamicin, and ciprofloxacin, we have applied the protocol of Gullberg et al. in detail (7). In particular, 10^4^ E. coli JM109 parental cells from an overnight culture were serially passaged by 1,000-fold dilution in 1 mL batch cultures every 24 h for 700 generations (10 generations of growth per passage) in LB medium containing the three antibiotics at 1/2, 1/4, and 1/10 of the MIC values and, more specifically, at 6 μg/mL, 3 μg/mL, and 1.2 μg/mL of ampicillin; 0.1 μg/mL, 0.05 μg/mL, and 0.02 μg/mL of gentamicin; 0.5 ng/mL, 0.25 ng/mL, and 0.1 ng/mL of ciprofloxacin (6, 7, 16). Cultures of E. coli JM109 cells were also analyzed (control) in the absence of antibiotics following the same selection procedure. Every 100 generations, the resistant cells were selected by plating approximatively 10^5^ cells onto LB agar containing different concentrations of antibiotics, thus monitoring the percentage of resistant cells. At the end of the procedure, resistant cells were isolated and maintained over time in LB agar containing 50 μg/mL of ampicillin, 2 μg/mL of gentamicin, and 2 ng/mL of ciprofloxacin. Colonies of these cells were inoculated in LB broth in the absence of the antibiotics and were grown for 100 generations with continuous shaking at 37°C. A 1,000-fold dilution of the resulting growths were plated on LB agar plates in absence and presence of the three antibiotics.

### DNA extraction.

Genomic DNA was extracted from the bacterial cultures incubated overnight at 37°C by using DNeasy UltraClean Microbial Kit (Qiagen) following the manufacturer’s protocol. Briefly, 1.8 mL of culture were centrifuged at 10.000 g for 30 s at room temperature in provided 2-mL collection tubes. Pellets were suspended in 300 μL of PowerBead Solution and vortexed. Resuspended cells were transferred to PowerBead Tubes and then 50 μL of Solution SL was added. After vortex for 10 min, the tubes were centrifuged at 10,000 g for 30 s. Supernatants were transferred to clean 2-mL collection tubes and then 100 μL of Solution IRS were added. After incubation at 4°C for 5 min, the tubes were centrifuged at 10,000 g for 1 min. Supernatants were transferred to clean 2-mL collection tubes and then 900 μL of Solution SB were added. 700 μL was loaded into MB Spin Columns and centrifuged at 10,000 × *g* for 30 s. The centrifugation was repeated after adding 300 μL of Solution CB. After discarding the flow-through, the samples were centrifuged at 10,000 × *g* for 1 min. The MB spin Columns were placed in new 2 mL collection tubes and 50 μL of Solution EB was added to the filter membrane. The samples were centrifuged at 10,000 × *g* for 30 s, the MB Spin Columns were discarded, and DNA sample recuperated and then stored at −20°C until further use. DNA concentration and 260/280 ratio were determined in a Nanodrop.

### RNA extraction.

RNA samples were extracted starting from 1× 10^9^ bacteria by using RNeasy Micro Kit (Qiagen) according to the manufacturer’s recommendation. Shortly, cells were resuspended in 700 μL of Buffer RTL and vortexed for 10 s. One volume of 70% ethanol was added to the lysate, the samples were transferred to a RNeasy MinElute spin column in a 2-mL collection tube and centrifuged for 15 s at 8,000 × *g*. After discarding the flow-through, 350 μL of Buffer RW1 were added to spin column and the samples were centrifuged for 15 s at 8,000 × *g*. After discarding the flow-through, 80 μL of Buffer RDD containing DNase I were added and, after incubation at 20°C for 15 min, 350 μL Buffer RW1 were added. The samples were centrifuged for 15 s at 8,000 × *g* and, after discarding the collection tube, the spin column was placed in a new 2-mL collection tube. Then, 500 μL Buffer RPE were added to the spin columns and centrifuged for 15 s at 8,000 × *g*. After discarding the flow-through, 500 μL of 80% ethanol were added to the spin column and centrifuged for 2 min at 8,000 × *g*. Therefore, the spin columns were placed in a new 2-mL collection tube and, after an additional centrifugation, 14 μL RNase-free water were added. The samples were centrifuged for 1 min to elute the RNA and checked for purity with an Agilent 2100 Bioanalyzer.

### Quantification of global 5-methylcytosine and N6-methyladenosine levels.

Global DNA methylation levels of 5-methylcytosines (5-mC) and N6-methyladenosines (m6A) were determined by using the MethylFlash Global DNA Methylation (5-mC) ELISA Easy Kit and MethylFlash m6A DNA Methylation ELISA Kit (Epigentek), respectively, following the manufacturer’s instructions. Briefly, the methylated fraction of 100 ng of bacterial genomic DNA, bounded to strip wells using DNA high binding solutions, was recognized by the 5-mC or m6A antibodies and quantified colorimetrically by reading the absorbance at 450 nm in a microplate spectrophotometer. The percentage of methylated DNA was calculated as a proportion of optical density measured. As a quality control, global methylation levels of samples obtained by mixing equivalent molar concentrations of the unmethylated (negative) and methylated (positive) controls at various ratios were also evaluated for each assay. Each sample was run in triplicate.

Global RNA methylation levels of 6-methyladenosine were quantified by the m6A RNA methylation quantification kit (Epigentek, Farmingdale, NY). A total of 200 ng of RNAs were coated on assay well, and the m6A content was captured and detected according to the manufacturer’s instructions. Shortly, the methylated fraction of total RNA, through ELISA-like reactions, was recognized by the m6A antibody and quantified in a microplate spectrophotometer by reading the absorbance at 450 nm.

In each experiment, the percentage of m6A was calculated using the second-order regression equation of a standard curve that was constructed by mixing equivalent molar concentrations at different ratios of full unmethylated and methylated control DNA. Each sample was analyzed in triplicate.

### Whole-transcriptome analysis.

RNA integrity (RIN value) of the samples was evaluated through an Agilent 2100 Bioanalyzer exhibiting values between 8.2 and 9.1. RNA sequencing was carried out by using the service provided by BMR Genomics (Padua, Italy) at ultra-high throughput on the Illumina NovaSeq 6000 (Illumina Inc.).

### Statistical analysis.

Differential expressed genes were identified using DESeq2 Bioconductor R package. The DESeq2 R package for differential gene expression uses negative binomial generalized linear models. Expression counts were normalized to correct the sequencing depth and batch differences among samples for the pairwise group. The normalization was based on the Relative Log- Expression approach, which is implemented in the DESeq2 package. DESeq2 performs an internal normalization where geometric mean is calculated for each gene across all samples. The counts for a gene in each sample is then divided by this mean. The median of these ratios in a sample is the size factor for that sample. After finding the differences in the expression of all genes between the different treatments, combined with the fold change (FC) and *P*-value of genes with differences, we set absolute value of a log_2_(FC) >1 and *P*-value < 0.05 as filter conditions to select significantly up and downregulated expressed genes. The R package PCA tool was used to calculate and plot the principal components. The volcano plots graphs used to display the results of the differential expression analyses were created by the Enhanced Volcano R package (https://github.com/kevinblighe/EnhancedVolcano).

Venn diagrams were created using Venn Diagram R Package version 1.7.1 (https://CRAN.R-project.org/package=VennDiagram).

All statistical analysis was performed using R Statistical Software (version 4.1.2, R Foundation for Statistical Computing, Vienna, Austria) (http://www.R-project.org/).

### GO functional enrichment.

Functional enrichment analysis on up- and downregulated genes in the three antibiotic-resistant cells was performed by using the GO Resource (http://geneontology.org/) that it is based on the PANTHER Classification System. It was used to describe GO functional classification of biological processes, molecular functions, and cellular components. The analysis was performed using the Fisher's exact test and the Bonferroni correction for multiple testing for *P*-value < 0.05.

### Kyoto encyclopedia of genes and genome pathway analysis.

Kyoto encyclopedia of genes and genome (KEGG) pathway analysis was implemented by using Search Tool for the Retrieval of Interacting Gene/Proteins database (STRING, https://string-db.org/). Strength values represent Log_10_(observed/expected). It is the ratio between the number of proteins in the network of the study that are annotated with a term and the number of proteins that we expect to be annotated with this term in a random network of the same size. Benjamini-Hochberg procedure was used to determine false discovery rate (FDR). We considered only results with FDR < 0.05.

### Protein-protein interaction network.

The PPI networks associated with up- and downregulated genes described above were constructed using STRING. We selected interactions derived from all active interaction sources (textmining, experiments, co-expression, neighborhood, gene-fusion, and co-occurrence) at a high level of confidence (score > 0.7). All nodes not connected to the main network were excluded from the analysis. Pathway analysis was performed using the Fisher’s Exact test and Benjamini-Hochberg procedure was used as FDR correction for multiple testing within each category. We considered only results with FDR <0.05. The Cytoscape software v3.7.2 was used to visualize and analyze the structure of the PPI networks. The Molecular Complex Detection (MCODE) tool was used to search for high modularity clusters within the network (degree cutoff = 2, node score cutoff = 0.2, k-score = 2, maximum depth = 100) and modules which consisted of at least six nodes and had an MCODE score of at least of 4 were considered significant. The PPI network was analyzed by Cytoscape, and nodes with degree higher than the mean values were classified as the hub genes.

### Data availability.

The raw data supporting the conclusions of this article will be made available by the corresponding author upon request.
