# Supplementary material for: Epigenetic-Based Regulation of Transcriptome in Escherichia coli Adaptive Antibiotic Resistance
Source: Microbiol Spectr. 2023 May 15;11(3):e04583-22. doi: 10.1128/spectrum.04583-22 (PMC10269836; doi:10.1128/spectrum.04583-22)
Supplement: Supplemental file 1 — Supplemental material. Download spectrum.04583-22-s0001.pdf, PDF file, 7.3 MB [file spectrum.04583-22-s0001.pdf]

Supplemental Figure Legends

Figure S1. Protein-Protein Interaction (PPI) networks of differentially expressed genes. Structure of the PPI networks constructed by analyzing the 307 up-regulated genes in ampicillin-resistant cells (**A**), the 260 up-regulated genes in gentamicin-resistant cells (**B**), and the 475 down-regulated genes in gentamicin-resistant cells (**C**) using STRING. Network nodes and edges represent protein and protein-protein association, respectively.

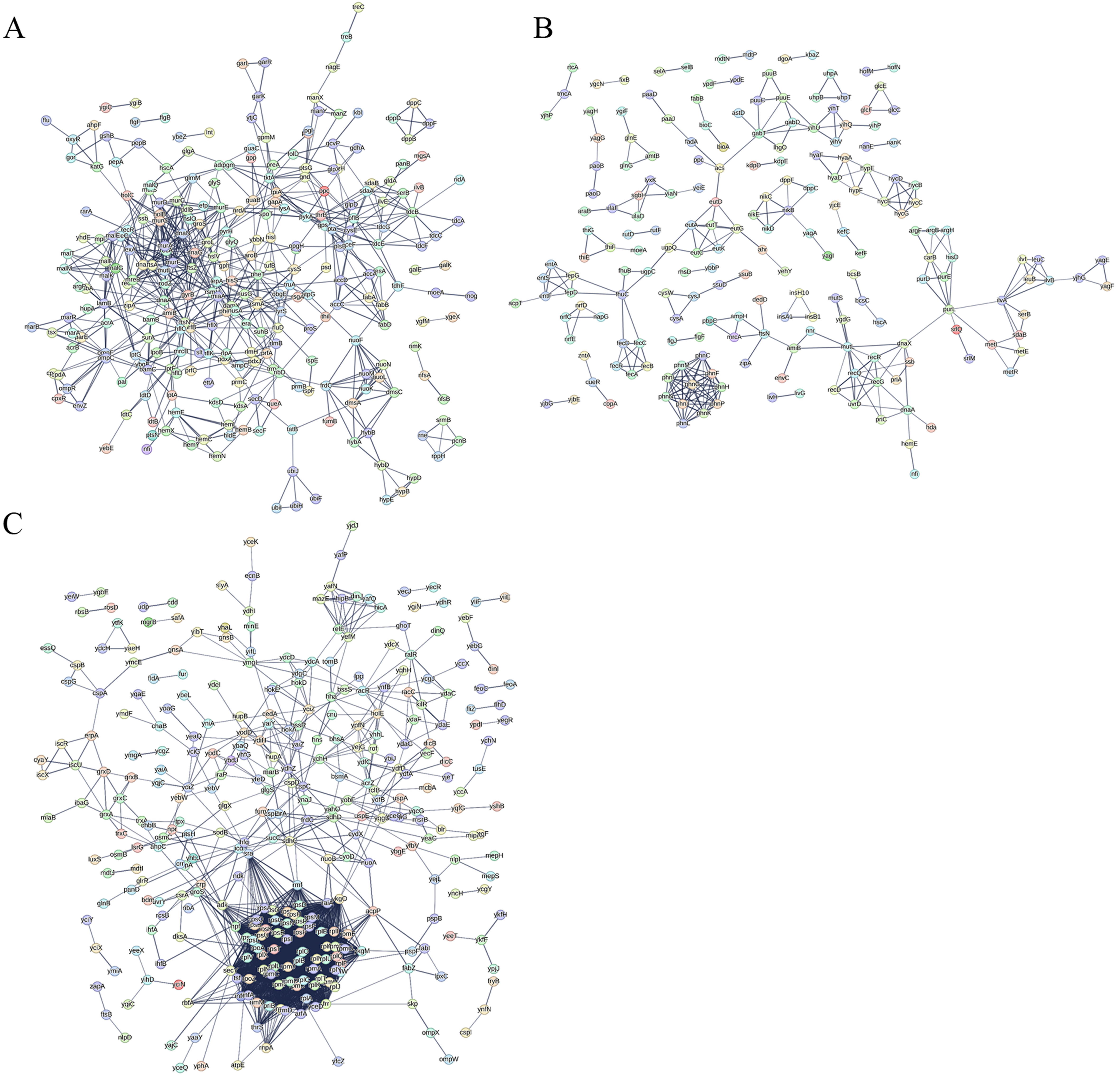
A
B
C

Table S1. Complete list of up- and down-regulated genes in ampicillin-resistant cells.

GENE	log2FoldChange	Standard error of the log2Fold Change	p-value	Corrected p-value
tdcF	7.256289128	2.296347383	0.001578089	0.08417859
tdcE	6.219753346	2.100285012	0.003062585	0.113271675
malE	5.978811362	2.205576706	0.006712715	0.17223638
tdcC	5.708468801	2.184934907	0.008984342	0.204620921
ompF	5.685716507	2.359316659	0.015956907	0.258757199
tnaA	5.670141907	2.058190227	0.005870714	0.164265687
lamB	5.402253493	2.031655044	0.007836349	0.184929184
ompC	5.398738146	2.564309996	0.035261935	0.32752865
tdcB	5.217471668	2.138682163	0.014704624	0.253499259
tdcG	4.994652033	1.820421498	0.006075494	0.164265687
cspA	4.912210551	3.111590305	0.114409093	0.403550127
marB	4.897389826	1.703676266	0.004045381	0.13666646
manY	4.772847794	1.851017326	0.009923036	0.21477476
marA	4.770420005	1.751236759	0.006449081	0.16992893
manZ	4.665830446	1.70544343	0.006222015	0.167046687
tsx	4.651889113	2.115498164	0.027880993	0.305485257
manX	4.647361752	1.89032548	0.013951948	0.250352735
pflB	4.593706903	2.168833046	0.03417044	0.326714539
hns	4.497008824	2.244869662	0.04515172	0.346676271
ompA	4.445073478	2.295950288	0.052861635	0.357424257
acrA	4.420641099	1.730110054	0.010615079	0.219558829
ahpC	4.288172949	2.338510656	0.06669562	0.367589495
gapA	4.266675292	2.112965063	0.043457771	0.346511754
lpp	4.241726811	2.757143061	0.123938619	0.409405752
fusA	4.164226879	2.417439786	0.084964994	0.382674906
malM	4.151346089	1.612777577	0.010052008	0.21504636
rplT	4.102330168	2.521564761	0.103758636	0.39608052
ptsH	4.012431958	2.194842387	0.06753177	0.367589495
csrB	4.012333672	2.544897755	0.114883262	0.403550127
deoD	4.00574789	2.242974404	0.074114014	0.373744519
tufA	4.005588032	2.120641181	0.058910987	0.36375562
infC	3.991894711	2.477083869	0.107064276	0.396303494
rpsS	3.984774455	2.34519307	0.089295772	0.385449519
rplW	3.950817477	2.460082439	0.108280601	0.398282207
ansB	3.941303734	1.678963108	0.018901752	0.274420156
acpP	3.934082232	2.437796294	0.10657379	0.396303494
rpmI	3.931201201	2.488470788	0.114160496	0.403550127
ompX	3.896149245	2.248995388	0.083203188	0.381205511
rrlB	3.886400682	3.267039283	0.234211981	0.517780307
rpsG	3.886255352	2.182246211	0.074936795	0.373744519
rpmF	3.883398151	2.087077871	0.062788425	0.36375562
malK	3.870511384	1.265145097	0.002218238	0.095667434
rplJ	3.85653469	2.528245349	0.127164364	0.409405752
yceD	3.854502119	2.452854029	0.116081505	0.403550127

yccA	3.847017936	2.282016355	0.09183477	0.387027055
thrS	3.826745784	2.210641695	0.083441679	0.381205511
ompW	3.820167422	2.243429249	0.088600822	0.384981492
eno	3.80805706	1.943694874	0.050091172	0.357424257
rpsL	3.786265843	2.616967287	0.147948925	0.4313125
rpsD	3.782917042	2.448481987	0.122345041	0.409049461
rpsA	3.778409973	2.360360365	0.109426377	0.398679812
hupA	3.76711463	1.877417712	0.044798615	0.346511754
rplB	3.755409457	2.32371004	0.106066958	0.396303494
aspA	3.752882588	2.352349994	0.110628193	0.399980076
frdB	3.732762677	1.931570953	0.053297707	0.357424257
tufB	3.731394968	1.80918615	0.039162863	0.337743189
rpsB	3.714395357	2.331983942	0.111203975	0.400259817
rplV	3.70059929	2.205377896	0.093349296	0.387027055
rplK	3.69757877	2.346690746	0.115104964	0.403550127
rpsQ	3.679882977	2.435464307	0.130799288	0.413297205
treB	3.666287237	1.51973495	0.015845635	0.258150962
adhE	3.664807902	1.961344621	0.061689891	0.36375562
rplC	3.657529194	2.414914364	0.129884236	0.412011498
rplL	3.645546178	2.085933014	0.080519224	0.37803293
rplA	3.638229122	2.190967641	0.096802225	0.390380788
rplX	3.633796952	2.448028098	0.137708895	0.423075749
rplE	3.632045588	2.477219775	0.142599751	0.427567449
cpxP	3.628324748	2.022360166	0.072796548	0.37206423
frdC	3.62714391	1.841482589	0.048874279	0.356018847
yebE	3.623269953	1.586814584	0.022409134	0.282132391
rpoA	3.616966908	2.438184336	0.137950279	0.423353847
grcA	3.61094551	2.365460334	0.126877994	0.409405752
hfq	3.594400515	1.853406224	0.052458921	0.357424257
udp	3.58594581	2.507901793	0.152757507	0.435805021
ampC	3.583359533	1.51840595	0.018277482	0.269443321
marR	3.580056425	1.541786342	0.020231939	0.277602331
glyT	3.575231079	4.31801801	0.40768202	0.675692113
rpsH	3.567439359	2.160953294	0.098766409	0.392933288
deoB	3.566822265	1.967578423	0.06986298	0.367589495
hupB	3.535022268	1.836729924	0.054275885	0.358362275
rplD	3.531858415	2.113816133	0.094752363	0.388800372
rplI	3.531372704	1.937272996	0.068324969	0.367589495
rpsR	3.530610822	2.058778523	0.086362535	0.382674906
psrD	3.522891474	2.036117089	0.0835944	0.381205511
acrB	3.506972411	1.691134495	0.03810351	0.335910045
groL	3.498240588	1.626816738	0.031526801	0.320451274
nupG	3.495324985	1.822781333	0.05516457	0.35965674
secY	3.491720022	2.377126002	0.141864521	0.427561927
rpmJ	3.482148286	2.864617607	0.224148095	0.508788564
rpsE	3.47672842	2.273765722	0.126249112	0.409405752
rpsC	3.47229635	2.214623246	0.116905677	0.404381925
tff	3.467811407	3.153324148	0.271448917	0.555504245
yniA	3.464238141	2.013677398	0.085368097	0.382674906

rpmE	3.461152443	2.249060177	0.123820706	0.409405752
raiA	3.448995656	2.689049883	0.199629607	0.485231275
rpmC	3.446211702	2.153924491	0.109605534	0.398679812
rbsB	3.437564584	2.389165012	0.150203168	0.431773005
pspE	3.428660431	1.828207599	0.060734715	0.36375562
pck	3.423471099	2.041526078	0.093558588	0.387027055
tsf	3.408070496	2.08427623	0.102021004	0.395053485
fbaA	3.407075249	2.10981851	0.106339607	0.396303494
rpmB	3.402544468	2.509364216	0.17511862	0.465410268
crr	3.382421219	2.116066299	0.109943385	0.399124575
rpmH	3.37543536	2.39895956	0.15941512	0.443535602
ptsI	3.365176632	1.825952004	0.065333242	0.367589495
tdcA	3.360910945	1.643737442	0.040886784	0.340359392
ytfK	3.351315931	2.015658496	0.096384371	0.390380788
priB	3.338886074	2.194582057	0.128153897	0.409405752
tig	3.335510972	1.97018086	0.090455972	0.386415711
ridA	3.331595577	1.603585366	0.037746985	0.334793019
rplR	3.314423659	1.972602343	0.092912769	0.387027055
rplO	3.311308688	2.380637976	0.164245647	0.451424986
rplP	3.311099134	1.882322926	0.078569107	0.374816185
rpsJ	3.309345723	2.338092504	0.156950047	0.44124514
rpsN	3.301561552	2.078894946	0.11225628	0.400636634
dsbA	3.286158473	1.443760093	0.022839357	0.283152147
rpsK	3.283541762	2.114286997	0.120417027	0.406808855
rpmD	3.274236841	1.811151815	0.070634489	0.367589495
hybC	3.263645946	1.711028192	0.056466482	0.361064857
frdA	3.262009218	1.790963624	0.068550479	0.367589495
dcuA	3.251813488	2.062073239	0.114804007	0.403550127
rplF	3.250359476	2.304958339	0.158492518	0.443018227
rplM	3.248733767	2.625586909	0.215962288	0.499435891
spr	3.248544939	1.987441008	0.102146241	0.395053485
dps	3.24534732	1.61332882	0.04426375	0.346511754
infA	3.244935941	2.030211844	0.10997093	0.399124575
rpsF	3.242444145	2.353095415	0.168219209	0.4570782
ihfA	3.241884477	1.806074792	0.072655444	0.37206423
rpmG	3.239884518	1.913122797	0.090359235	0.386409641
tpx	3.215724507	1.960690948	0.100984877	0.395053485
rplQ	3.212899321	1.933518068	0.09657581	0.390380788
rpsU	3.202923576	2.313024991	0.166133921	0.453730509
prs	3.201031265	1.7809629	0.072278506	0.371865892
sodB	3.199997705	2.088449581	0.125464199	0.409405752
rimM	3.196427614	2.252708875	0.155920537	0.440489098
atpA	3.194574646	1.897255199	0.092222738	0.387027055
pnp	3.179227385	1.806939214	0.078500337	0.374816185
hflC	3.173123031	1.285607762	0.013579857	0.248300374
rpsM	3.170566871	2.42414996	0.190904272	0.48030403
rpsI	3.166308806	2.044518068	0.121458127	0.407401922
rplU	3.154129298	2.242129518	0.159499735	0.443535602
rihA	3.151475068	1.500292291	0.035678373	0.32752865

rpoB	3.149766752	1.930674775	0.102798963	0.39523855
malF	3.147852874	1.472197688	0.032500527	0.321852891
cydA	3.1359314	2.121683052	0.139397201	0.426425317
atpD	3.132259395	1.690350259	0.063878936	0.366790439
zapB	3.127333562	1.723966117	0.069672455	0.367589495
deoC	3.120105679	1.787082175	0.080824546	0.37803293
crp	3.116392392	2.004305231	0.119981988	0.406808855
fabI	3.115157444	1.702300291	0.067254514	0.367589495
melA	3.111518822	1.351133576	0.021284749	0.277602331
rplS	3.101268615	1.814993781	0.087507684	0.383361441
talB	3.101180785	1.783777551	0.082114508	0.379645316
preT	3.089512209	1.502099764	0.03970589	0.338654556
deoA	3.083982502	1.612492881	0.055804952	0.359671341
rplY	3.082625231	1.886353144	0.102222769	0.395053485
pykF	3.076076857	1.63828475	0.060433114	0.36375562
yajC	3.075265428	1.643003776	0.061243464	0.36375562
ygiC	3.073896639	1.55304927	0.047786136	0.353745052
secG	3.070140219	1.708591417	0.072354071	0.371865892
ackA	3.066006097	1.642855526	0.062004101	0.36375562
yfcZ	3.054516959	2.184767074	0.162083849	0.447302875
trxA	3.05006818	1.99284467	0.125890604	0.409405752
trmD	3.049136089	2.067979564	0.14036	0.426551303
miaA	3.047197822	1.461838292	0.037114965	0.33362321
sdaC	3.037054938	1.662845631	0.067786983	0.367589495
fabG	3.036127831	1.559227511	0.05151076	0.357424257
tolC	3.032240903	1.673242657	0.06995617	0.367589495
ybgT	3.031125912	3.048689928	0.320106607	0.607490627
rpmA	3.020843508	1.841190574	0.100859464	0.395053485
aceE	3.016925008	1.683849542	0.07318372	0.37206423
purA	3.014767952	1.64831553	0.067399713	0.367589495
sspA	3.009690954	1.86255854	0.106117955	0.396303494
dgoA	3.003335445	1.606009332	0.061475345	0.36375562
malG	3.002365497	1.012952644	0.003036938	0.113271675
rpsO	2.995731517	2.708935851	0.268782691	0.551160865
bamE	2.992121972	1.667162815	0.072695308	0.37206423
infB	2.982850935	1.77673258	0.093183098	0.387027055
efp	2.980167027	1.508737165	0.048237233	0.353805533
ygiB	2.979407673	1.287739477	0.020685682	0.277602331
dppA	2.975874214	1.793541574	0.097072124	0.390380788
rpsT	2.975459294	2.250519433	0.186127819	0.473701881
ihfB	2.968205497	1.759182948	0.091552661	0.387027055
agp	2.965481444	1.685860531	0.078572192	0.374816185
preA	2.96148769	1.311922492	0.023985288	0.286927951
rpoC	2.952473745	1.848804642	0.110273818	0.399865884
lrhA	2.946661801	1.601461277	0.065770708	0.367589495
ftsH	2.943947766	1.623418405	0.069766273	0.367589495
slyD	2.941993123	1.545735889	0.057001917	0.362203401
lpd	2.941002249	1.844893187	0.110906596	0.399980076
atpG	2.938148587	1.420822556	0.038647466	0.336799028

yidC	2.934228584	1.512160906	0.052328562	0.357424257
tpiA	2.934100973	1.383451697	0.033933975	0.325991311
cspE	2.932854267	2.741819225	0.284765822	0.568970253
nusA	2.929913949	1.393534002	0.035508953	0.32752865
groS	2.924449094	1.390188902	0.035410357	0.32752865
cdd	2.918991146	2.189031204	0.182380055	0.472440092
yhbY	2.918544865	1.753321517	0.095996472	0.390380788
uspA	2.917924648	2.057431586	0.156121718	0.44075031
ppiB	2.913869367	1.654370442	0.078184909	0.374816185
rdoA	2.90350026	1.609714894	0.071272746	0.369488125
hybA	2.902743386	1.479162504	0.049713189	0.357424257
dnaK	2.899948734	1.504276836	0.053879689	0.357756229
rplN	2.897765809	2.426296905	0.232354285	0.516947839
phnI	2.892637686	1.823541663	0.112677048	0.400636634
rnpB	2.890986177	2.148506194	0.178438124	0.468515643
gnd	2.884407695	1.407910969	0.040490035	0.339781285
typA	2.878939422	1.676801696	0.085993239	0.382674906
nusB	2.878292443	1.541230246	0.061827727	0.36375562
ybfA	2.877862389	1.87675258	0.125170771	0.409405752
pykA	2.877487991	1.328171673	0.030272766	0.315490476
fis	2.876705358	1.689226967	0.088573405	0.384981492
rpsP	2.875221602	2.074401812	0.165732082	0.453664998
tatA	2.861879263	1.611963814	0.075831996	0.373744519
dksA	2.859966172	1.717168909	0.095810565	0.390380788
hybB	2.844050838	1.382124567	0.039615249	0.338654556
fecC	2.842688672	1.481150033	0.054953431	0.35965674
glnA	2.831047142	1.53833921	0.065720248	0.367589495
nlpI	2.827349001	2.215052405	0.201805206	0.486396139
yciN	2.826518295	1.700661094	0.096510469	0.390380788
treC	2.823129478	1.293477697	0.029066131	0.312860316
atpE	2.816431892	1.808836628	0.11946087	0.406500994
lrp	2.813381688	1.520073947	0.064195602	0.366945102
atpH	2.804756319	1.567996871	0.07365489	0.373246153
phnK	2.80209828	1.585419463	0.077158316	0.374512315
sdaA	2.795152854	1.209223972	0.020803809	0.277602331
rho	2.794184105	1.795674418	0.119692903	0.406500994
gyrA	2.793185648	1.732108499	0.10683301	0.396303494
cspD	2.792938609	1.767000589	0.113967263	0.403513785
frdD	2.785872218	1.489619243	0.061457325	0.36375562
hybO	2.782167315	1.58867816	0.079903432	0.377979595
uspE	2.781333079	1.829803979	0.128506713	0.409893472
kdsA	2.780816896	1.172407661	0.017697567	0.266133831
deaD	2.778420246	1.69896556	0.101973611	0.395053485
pal	2.775996462	1.348789663	0.039576803	0.338654556
lptE	2.771163056	1.396542763	0.047222147	0.352557243
ftnA	2.77114345	1.271461218	0.029294911	0.312860316
rpoH	2.768730602	1.758185146	0.115310413	0.403550127
atpF	2.768209171	1.420480653	0.051321292	0.357424257
pgk	2.768113945	1.766390053	0.117090778	0.404676908

gcvH	2.765846971	1.223486522	0.023782349	0.286927951
hybG	2.76438799	1.526008627	0.070061121	0.367589495
lpxC	2.762464469	2.318592938	0.233480881	0.516947839
cydB	2.762181339	1.829362049	0.131065095	0.413814561
ftsZ	2.758745159	1.396675776	0.048242925	0.353805533
pta	2.75825099	1.158132138	0.017235852	0.263454633
yobF	2.757518712	3.167410131	0.383977519	0.656812179
phnM	2.750625947	1.89217514	0.146033757	0.430851022
fabF	2.74955381	1.408691338	0.050956048	0.357424257
hflK	2.749305456	1.114100956	0.01359711	0.248300374
glgS	2.745083052	1.651486859	0.096474914	0.390380788
secB	2.736366491	1.489335675	0.066164624	0.367589495
mgsA	2.735219248	1.362929879	0.044763807	0.346511754
rimP	2.731813552	1.453753821	0.06022476	0.36375562
serS	2.723132192	1.60496302	0.089754402	0.385449519
cspB	2.719245516	2.081125158	0.19134086	0.48030403
lptA	2.718268196	1.352302998	0.044420298	0.346511754
lysS	2.71821002	1.418768807	0.055378645	0.35965674
rmf	2.711776919	2.232310998	0.224448274	0.509184836
rbsD	2.703491203	2.478579781	0.2753864	0.559046803
luxS	2.699294698	1.479222804	0.068030305	0.367589495
ygdH	2.684949102	1.570529924	0.087343266	0.383361441
mokB	2.680634405	2.157772756	0.214120202	0.497251883
rpoD	2.680175908	1.371676102	0.050708046	0.357424257
rrsH	2.677420468	1.369412815	0.050564248	0.357424257
yrbL	2.676369446	1.602013696	0.094795116	0.388800372
zapA	2.674918647	2.146688794	0.212739699	0.496229425
htpX	2.673738497	1.158859844	0.021042853	0.277602331
pepQ	2.671265174	1.386391421	0.0540076	0.357756229
seqA	2.670397406	1.43647672	0.063028403	0.36375562
glmZ	2.66898311	1.956892286	0.172602624	0.462863982
iscS	2.666373911	1.464798708	0.068713246	0.367589495
phnH	2.663481833	1.786865449	0.136069456	0.421088226
ppa	2.659806038	1.459156222	0.068327865	0.367589495
rne	2.655516578	1.18726289	0.025307845	0.295116348
slyB	2.641570275	1.47658307	0.073618833	0.373246153
gpr	2.64071766	1.638021023	0.106931684	0.396303494
fur	2.638848013	1.796739217	0.141917632	0.427561927
garR	2.637932602	1.010952442	0.00907134	0.205047566
glyA	2.634966366	1.458171545	0.070756892	0.36775441
ssrA	2.626117856	2.716701256	0.333715609	0.618885214
yjfN	2.625545578	1.751109598	0.133779985	0.417851314
rbsK	2.622503517	1.699617836	0.122831747	0.409386299
atpB	2.620107333	1.686070077	0.120190863	0.406808855
yibN	2.613599359	1.558172793	0.093474283	0.387027055
hybD	2.612265892	1.129385914	0.020722821	0.277602331
grxB	2.594751888	1.489197537	0.081441517	0.379063044
hybE	2.590410443	1.377567853	0.060050435	0.36375562
mipA	2.566654848	1.310451859	0.050159306	0.357424257

hflX	2.559928064	1.100482345	0.02000856	0.277602331
rihC	2.559304546	0.835930756	0.002201408	0.095667434
rrlC	2.557684526	1.582632035	0.106073665	0.396303494
cpxR	2.556848632	1.171701494	0.029097191	0.312860316
rlmE	2.554647963	1.442157335	0.076493003	0.373744519
nupC	2.545292042	1.479864465	0.085441634	0.382674906
accC	2.542451988	1.097704673	0.020549916	0.277602331
zipA	2.540700522	0.82589657	0.002095912	0.095180594
tktA	2.537505052	1.09098221	0.020024398	0.277602331
yifE	2.536771678	1.479622002	0.086441928	0.382674906
suhB	2.535282956	0.988949999	0.010358961	0.216948897
eptB	2.526378654	1.536104275	0.100038684	0.394894668
ygfM	2.523687015	1.143989258	0.027381141	0.301638982
nuoK	2.512920836	1.011480611	0.012977052	0.245836311
rnpA	2.509438808	1.501497936	0.094664368	0.388800372
bamA	2.50646024	1.579980969	0.112651695	0.400636634
ahpF	2.505329146	1.19507727	0.03604901	0.328323548
clpP	2.501190084	1.348563372	0.063638062	0.365941422
secE	2.500362367	1.344998903	0.063026147	0.36375562
osmB	2.497513627	1.618893869	0.122896676	0.409386299
aceF	2.49637683	1.155567629	0.03074918	0.31710311
fabB	2.492880857	1.058626268	0.018531371	0.271841357
dapA	2.490316689	1.562568759	0.110995951	0.399980076
nusG	2.484749258	1.260449789	0.048687341	0.355636903
rcsB	2.482301466	1.561844793	0.111983788	0.400636634
hemB	2.481637374	1.025123201	0.0154856	0.257639363
yebC	2.481227552	1.210047027	0.040313559	0.339178316
arcA	2.473100634	1.389635688	0.075129077	0.373744519
degP	2.472312021	1.269942819	0.051559912	0.357424257
hypD	2.469981524	0.790471964	0.001779912	0.091532809
bamD	2.464826002	1.452129907	0.089623574	0.385449519
sdaB	2.458361825	0.726218195	0.000711378	0.051950319
leuS	2.456629661	1.303892754	0.059555047	0.36375562
dusB	2.456235691	1.812979215	0.175479274	0.465571319
dppC	2.456007436	1.133054833	0.030189283	0.315490476
iscA	2.449077205	1.499800798	0.10248267	0.39523855
accB	2.448725588	1.42266296	0.08520968	0.382674906
ygiN	2.448198844	1.51634135	0.106409622	0.396303494
tomB	2.444873016	1.647116504	0.137720015	0.423075749
clpA	2.441144979	1.659028514	0.141174759	0.427561927
dinQ	2.441123647	3.114685947	0.433188978	0.694678843
iscU	2.440901358	1.540118417	0.112993825	0.401118186
ydcH	2.440858447	1.881199922	0.194458922	0.482342452
nfsB	2.438098027	1.145724765	0.033337393	0.324660621
grxD	2.43801163	1.632631212	0.135358124	0.419527397
cptB	2.435399454	1.698623048	0.151643843	0.433849076
grpE	2.434693532	1.38390497	0.078527852	0.374816185
sra	2.433648734	3.025170232	0.42112749	0.685918378
ppiA	2.426074774	1.341477566	0.070527257	0.367589495

nuoM	2.425931485	1.089147756	0.025922773	0.297707999
secF	2.423795321	1.221034888	0.04714073	0.352557243
frr	2.423579047	1.469607219	0.099120211	0.393168651
rbsC	2.42353775	1.739701179	0.163596499	0.450251328
guaA	2.422989645	1.309486491	0.064265176	0.366945102
glyS	2.420715658	0.902904449	0.007339551	0.179028999
atpC	2.419035728	1.295134524	0.061791734	0.36375562
psd	2.412995166	1.092262717	0.027162746	0.300520559
yjbG	2.411254611	1.57698766	0.126258081	0.409405752
malP	2.409116945	1.229742961	0.050107979	0.357424257
rbfA	2.403702305	1.542495813	0.119157375	0.406500994
bsmA	2.402319911	2.01899689	0.234102148	0.517780307
gdhA	2.394567298	1.108900404	0.030818605	0.31710311
nrdD	2.394553597	1.268651199	0.059095812	0.36375562
pgi	2.393530783	1.412254764	0.090107803	0.386149085
ptsG	2.390584966	1.21035199	0.048255043	0.353805533
nikC	2.389300162	1.426715585	0.093995918	0.388278036
ybaB	2.38610668	1.428508199	0.094850412	0.388800372
fepD	2.381972613	1.585410706	0.132985441	0.416632905
ypdE	2.381738859	1.626491327	0.143100645	0.427567449
mreB	2.379973523	0.945119699	0.011796585	0.231030713
secD	2.376320682	1.134528263	0.036211445	0.328323548
fabD	2.373911878	0.940802441	0.011626442	0.228803855
rhlB	2.371543199	1.294601906	0.066970698	0.367589495
accD	2.367907958	1.169691734	0.042930417	0.346511754
tyrS	2.36788356	1.09118416	0.030005879	0.315424071
sspB	2.365889451	0.968928732	0.014615942	0.253499259
pheT	2.364274815	0.983585649	0.016228733	0.261076519
nuoG	2.361010366	1.253410525	0.059609782	0.36375562
hha	2.359348838	1.623394618	0.146128607	0.430851022
ucpA	2.359298218	1.374280767	0.086024633	0.382674906
aspC	2.354489466	1.282580298	0.066395483	0.367589495
mdh	2.349634903	1.445134448	0.103972284	0.396150037
ompR	2.348889344	0.935561254	0.012050091	0.231521653
skp	2.347365666	1.541741588	0.127873436	0.409405752
phnL	2.346611684	1.696703388	0.166652054	0.454342586
ppiD	2.346449617	1.28960127	0.068832806	0.367589495
phnF	2.340795239	1.691155098	0.166315623	0.453730509
cspC	2.336854775	1.616932564	0.148390631	0.4313125
yijD	2.329897881	1.423147585	0.101600333	0.395053485
ribE	2.32880954	1.197637796	0.051834899	0.357424257
yfbU	2.32592337	1.361992067	0.087685273	0.383469359
phnC	2.324187278	1.530697506	0.128917511	0.410551132
gcvP	2.321278527	1.097305341	0.034392858	0.327274732
ydhF	2.320143033	1.120994336	0.038478687	0.336799028
xdhD	2.318945641	1.354556021	0.086904052	0.383361441
hldD	2.318191969	1.217025033	0.056805787	0.362092233
erpA	2.317933604	1.553637942	0.135715042	0.420312284
dmsA	2.317801266	1.138057083	0.041687128	0.344194738

uxaC	2.316033673	1.397842064	0.097547049	0.390380788
yajO	2.315094956	1.197941608	0.053290329	0.357424257
zntA	2.307056763	1.237967521	0.062380112	0.36375562
lepA	2.304904964	0.984075339	0.019170245	0.274420156
glnS	2.304505104	1.244056346	0.06396666	0.366790439
nuoE	2.302244707	1.224516187	0.060090909	0.36375562
lysA	2.29836515	1.058710705	0.029938012	0.315424071
nfsA	2.293833553	1.158793223	0.04775951	0.353745052
icd	2.290399788	1.977667476	0.246810214	0.526615058
nuoL	2.289588999	0.932087407	0.014033288	0.250352735
rhsD	2.288732319	1.900856191	0.228568948	0.513487682
add	2.285502476	1.112453219	0.039930162	0.338654556
ispG	2.284576116	1.199342344	0.056798613	0.362092233
rbsA	2.282708697	1.855520758	0.218612725	0.502753402
nuoC	2.2759061	1.161512258	0.050062033	0.357424257
fabA	2.272178182	0.929091433	0.014461472	0.253499259
hpf	2.267828694	1.572172042	0.149166657	0.4313125
tilS	2.267818046	1.326183119	0.087259611	0.383361441
smpB	2.265204552	1.495421922	0.129833322	0.412011498
prfA	2.264667539	0.976500524	0.020385997	0.277602331
ybgE	2.264343961	1.524343059	0.137423172	0.423017114
fumB	2.261164545	0.947317025	0.016990452	0.263454633
nuoA	2.257234857	1.382247249	0.102465178	0.39523855
clpX	2.256769264	1.4417575	0.117514819	0.405451127
yhbT	2.256347718	1.173245315	0.054458968	0.358660795
aspS	2.256017743	1.235978413	0.067957436	0.367589495
thiF	2.255056269	1.417136758	0.111547387	0.400636634
pgm	2.254180421	0.925653719	0.014882295	0.253499259
fabZ	2.253704593	1.432727801	0.115714978	0.403550127
lpxA	2.253020717	1.170507542	0.054251412	0.358362275
bamB	2.252828445	1.007973823	0.025416896	0.295116348
tgt	2.249434747	1.324908338	0.089544751	0.385449519
accA	2.248717903	0.96643034	0.019974381	0.277602331
adk	2.248455164	1.444949669	0.119689419	0.406500994
aroK	2.247489637	1.370944944	0.101135675	0.395053485
ycfP	2.247296039	1.328197229	0.090647936	0.386603764
ygfB	2.245849646	1.21282992	0.064062697	0.366822278
bamC	2.244162824	0.993892955	0.023948637	0.286927951
fecA	2.241801411	1.340526776	0.094459522	0.388800372
prfB	2.241221293	1.127571369	0.046850014	0.352374686
yoaE	2.240210917	0.972942137	0.021306317	0.277602331
ybiS	2.238427941	0.884547394	0.011387044	0.225664635
dtpA	2.236375708	1.236548125	0.070518855	0.367589495
hypE	2.23389465	0.626166793	0.000360305	0.032978924
rrsD	2.23291554	1.381361973	0.105995026	0.396303494
wzzB	2.231439592	1.307120785	0.087795833	0.383539124
malQ	2.229179214	0.866889301	0.0101268	0.21504636
csrA	2.227349625	1.918592552	0.245670797	0.525469563
nrdB	2.22548526	1.144191714	0.051771573	0.357424257

greA	2.224772308	1.296656845	0.086203103	0.382674906
gshB	2.221151327	0.981154013	0.023585486	0.286927951
hisS	2.220474235	1.12202601	0.047817535	0.353745052
speA	2.21909893	1.137208109	0.051014601	0.357424257
lexA	2.218745016	0.979213687	0.023460964	0.286927951
pyrH	2.217044536	1.045207282	0.033908931	0.325991311
uxaA	2.216054605	1.223567497	0.070118858	0.367589495
ybeD	2.210873921	1.630777351	0.175188966	0.465410268
cpdA	2.206164509	1.023566436	0.031132883	0.317916139
gpmM	2.201738494	1.083522059	0.042151631	0.345787635
ybjC	2.201188934	1.409143862	0.118270232	0.405691946
ubiE	2.199764369	1.166213929	0.059262042	0.36375562
ycbL	2.199707266	1.353310282	0.104071576	0.396156027
flgB	2.19965885	1.118960937	0.049321156	0.357424257
dnaA	2.198411608	0.769436152	0.004274338	0.140879412
ycbK	2.192058765	1.667133317	0.188554568	0.476558741
ycgL	2.188522777	1.469754503	0.13647692	0.421705361
yqgC	2.187683347	1.668420137	0.189779906	0.47904868
nanA	2.185571496	0.924419484	0.018065945	0.268142043
yeeN	2.184035103	1.256225251	0.082110488	0.379645316
fecR	2.181524105	1.513357169	0.149439794	0.4313125
eutG	2.176363275	1.574439433	0.166876556	0.454525976
cspG	2.170993395	1.774161568	0.221075654	0.505144582
recR	2.170458933	0.590821451	0.00023913	0.025511361
yfeC	2.170285943	1.545321513	0.160192759	0.443593883
proQ	2.170047258	1.066502355	0.041877772	0.345066032
pssA	2.165634267	1.280859994	0.090881542	0.386603764
def	2.163090717	1.411514309	0.125408699	0.409405752
hypC	2.1627848	1.140920516	0.058006477	0.36375562
ycfS	2.162375447	0.956433376	0.023767055	0.286927951
ybhL	2.154627969	1.33130466	0.105569222	0.396303494
pitA	2.153555827	1.20948844	0.074985834	0.373744519
uidR	2.150406326	1.375428112	0.117947942	0.405691946
kbl	2.148149339	0.896863524	0.01661222	0.262886141
rraB	2.147899303	1.3364257	0.108011172	0.398070265
pheS	2.147191447	0.951247442	0.023993235	0.286927951
asd	2.145889018	1.096218938	0.050284378	0.357424257
gcvT	2.145476909	1.094808991	0.050033031	0.357424257
ileS	2.144494793	0.965974172	0.02641651	0.299970731
csrC	2.144346928	1.55152294	0.166943556	0.454525976
nuoN	2.1434566	0.872119253	0.013980801	0.250352735
ybjN	2.142069592	1.353484823	0.113505163	0.402580869
nudB	2.141341226	1.296767526	0.098679062	0.392933288
valS	2.140200349	1.13799304	0.06001551	0.36375562
gshA	2.136058999	1.097341695	0.051585589	0.357424257
secA	2.132060757	1.208592665	0.077717591	0.374816185
nuoJ	2.130677017	1.186224144	0.072465233	0.371865892
glyQ	2.130172809	0.771565227	0.005765185	0.164265687
rsmA	2.124326609	0.920067801	0.020950213	0.277602331

nuoB	2.122210232	1.391004548	0.127092728	0.409405752
ddlB	2.120407983	1.068149405	0.047130785	0.352557243
aspT	2.119549843	3.10865847	0.495351896	0.739780307
yidD	2.118825066	1.350852139	0.116762162	0.404381925
ftsA	2.116113872	1.005399068	0.03531306	0.32752865
rob	2.115644525	1.175776301	0.071961796	0.37116173
obgE	2.111956216	0.985932935	0.032186311	0.321852891
ydfG	2.111504145	1.162385449	0.069289624	0.367589495
apt	2.108844964	1.249016862	0.091333719	0.387027055
ptsN	2.103640722	1.040125601	0.043126044	0.346511754
garL	2.10360349	0.762226418	0.00578342	0.164265687
ffh	2.100593547	1.141170552	0.065660277	0.367589495
flhD	2.099248182	1.52813737	0.169525513	0.459396009
fepG	2.098351844	1.47209393	0.154035886	0.43699194
upp	2.097957721	1.315619535	0.110789669	0.399980076
alaS	2.097878167	1.183512816	0.076297345	0.373744519
rseB	2.094550557	1.297052287	0.106342235	0.396303494
rhsB	2.093916913	2.011506651	0.297889741	0.582202085
hokB	2.093010881	1.818874885	0.249848538	0.529475156
nanE	2.091895841	1.221570079	0.086811034	0.383361441
opgG	2.091171894	1.278364143	0.10187755	0.395053485
hinT	2.08892559	1.464482764	0.153755401	0.436807564
lysU	2.087370172	1.282594143	0.103639519	0.395998689
ydbJ	2.086941463	1.341280658	0.119724269	0.406500994
thiE	2.082797412	1.324680613	0.115880628	0.403550127
btuB	2.081482374	1.079933976	0.053927767	0.357756229
tolB	2.080521516	1.086138439	0.055426032	0.35965674
relB	2.078535404	1.580114447	0.188364269	0.476558741
glpK	2.078317653	1.32214103	0.115965996	0.403550127
yebA	2.078309406	1.119522145	0.063392939	0.365049682
rpoE	2.07583155	1.598269273	0.1940127	0.48194086
mlaD	2.074021988	1.402443042	0.139176769	0.426425317
sfsA	2.069008937	1.318118487	0.116492276	0.404162056
yaeH	2.066153062	1.452355499	0.154845728	0.438982224
rmuC	2.065695465	1.15959775	0.074848813	0.373744519
slyA	2.063729494	1.427309901	0.148208768	0.4313125
iscX	2.063459465	1.555530395	0.184663837	0.472916736
nikD	2.063387144	1.1189304	0.065172788	0.367589495
mlaC	2.060972958	1.220406863	0.091265642	0.387027055
rodZ	2.060484288	0.98002858	0.035511804	0.32752865
mrp	2.060281622	1.104471101	0.062125201	0.36375562
ftnB	2.05837904	1.296543281	0.1123791	0.400636634
gyrB	2.057517356	1.022143972	0.044120647	0.346511754
smg	2.05722603	1.305716859	0.11512887	0.403550127
glgA	2.05099429	0.667188987	0.002111507	0.095180594
gltX	2.048909027	1.157844277	0.076795556	0.373744519
yeeX	2.048516548	1.542204677	0.184077719	0.472916736
lpxD	2.044865934	1.149357056	0.07521698	0.373744519
surA	2.044807638	1.011086817	0.043136509	0.346511754

gltP	2.043628867	1.26676774	0.106687494	0.396303494
yihA	2.042492696	1.10811044	0.065296715	0.367589495
yhcB	2.042308832	1.508789306	0.175861567	0.465671321
ilvA	2.041093006	1.174069662	0.082126814	0.379645316
yejL	2.039938625	1.666832416	0.221012039	0.505144582
flgF	2.036838563	0.854009382	0.017077698	0.263454633
murF	2.036738244	0.609251581	0.000828729	0.055085064
yjjI	2.034674499	0.97658024	0.037208509	0.333724107
gltW	2.033880903	4.562151049	0.65573001	0.838288833
pfkA	2.033821999	1.10310553	0.065223392	0.367589495
serV	2.031341623	4.494631006	0.651306103	0.835568019
trxB	2.029586162	1.108215367	0.067040789	0.367589495
ugpC	2.029363328	1.603688931	0.205715608	0.489461478
damX	2.029153917	0.750204811	0.006834554	0.173701536
nanK	2.028323248	1.146088737	0.076764041	0.373744519
yedF	2.028270246	1.454226654	0.163093809	0.44917276
fbp	2.027941208	1.226819093	0.09832933	0.39234951
pyrG	2.02753067	1.263868192	0.108664166	0.398282207
yajG	2.026969712	1.235249964	0.1008101	0.395053485
fkpA	2.026547756	1.38769281	0.144187858	0.428546609
msbA	2.026488929	1.172642297	0.0839633	0.381835591
tusE	2.026177587	1.429295129	0.156305779	0.440962859
hycE	2.026143757	1.430859355	0.156765906	0.441176167
narP	2.025922438	1.289363449	0.116122768	0.403550127
fdx	2.024992505	1.283776365	0.114710024	0.403550127
purU	2.02437122	1.122724419	0.071374355	0.369542316
metL	2.023811098	1.203659456	0.092688779	0.387027055
fabH	2.020897333	1.038006711	0.051546486	0.357424257
yqgB	2.017375903	1.783975242	0.2581259	0.538596601
fhuB	2.013650871	1.544250581	0.192245039	0.48030403
nrdA	2.012442927	0.930879268	0.030627952	0.31710311
ynfK	2.00528482	1.099350746	0.068142576	0.367589495
pepA	2.001141948	0.675173217	0.003037728	0.113271675
lon	1.999455564	1.217779845	0.100613651	0.395053485
selB	1.999414292	1.066285082	0.060775983	0.36375562
yfiF	1.997989556	0.650690402	0.002136516	0.095180594
dnaX	1.996527194	0.876699787	0.022767039	0.283121405
rnk	1.995844973	1.355291135	0.140850316	0.427080912
mutL	1.995520476	0.784502289	0.010969245	0.223463909
pepD	1.994310999	1.624273831	0.219515678	0.503631329
rseA	1.991484581	1.750785773	0.255337495	0.535508641
ssnA	1.990764318	1.089010496	0.06754223	0.367589495
lpxP	1.988754772	1.262421514	0.115175854	0.403550127
ydgH	1.988573167	1.187635217	0.094052548	0.388278036
chaA	1.988416662	1.147964797	0.083251598	0.381205511
tnaB	1.987510803	1.388487188	0.15230937	0.434832525
rrlH	1.987409097	1.61123401	0.217400397	0.500762051
lptC	1.979479972	1.246877484	0.112388201	0.400636634
lptD	1.978776009	1.357787475	0.145018891	0.429756275

fldA	1.971582805	1.532635739	0.198303455	0.485231275
kdsB	1.970516419	1.205801065	0.102217503	0.395053485
dapD	1.969065593	1.143367366	0.085039897	0.382674906
envC	1.967607681	1.020367257	0.053813741	0.357756229
rimK	1.967386188	0.586697896	0.000798481	0.055085064
nuoF	1.962453341	0.865939695	0.023434844	0.286927951
truB	1.961648201	1.052608767	0.06237703	0.36375562
mltD	1.961221074	1.173078147	0.09455216	0.388800372
proS	1.960624282	0.832902206	0.018574261	0.271841357
metG	1.959358048	1.165444918	0.092722065	0.387027055
dppB	1.959075696	0.667767615	0.003348737	0.120571195
yicG	1.955594066	0.883893556	0.026933731	0.299970731
yihD	1.950485784	1.357692978	0.150826647	0.432426611
ybgF	1.946638648	0.91421201	0.033228813	0.324660621
ygeY	1.946003417	1.099389217	0.076714337	0.373744519
speE	1.94395252	1.20006876	0.105260718	0.396303494
rhlE	1.94232014	0.71367333	0.006497036	0.16992893
lolA	1.940259827	1.217356426	0.110974	0.399980076
gloA	1.938508745	1.26219126	0.124580558	0.409405752
der	1.936394911	1.026057019	0.059130796	0.36375562
hscA	1.935666435	0.861789591	0.024697541	0.294481861
pheA	1.933131097	1.109104633	0.081339643	0.37902404
hspQ	1.929943215	1.426200659	0.175989846	0.465706811
gmk	1.925566478	1.413044152	0.172974576	0.462863982
ycaR	1.923997565	1.556581418	0.216443536	0.499978401
acnB	1.92393318	1.682033858	0.252700954	0.532458247
pepP	1.923921584	1.076120517	0.073803283	0.373531224
fmt	1.922139672	1.067121081	0.071665282	0.370575325
fnr	1.919566289	1.11559507	0.085311485	0.382674906
galE	1.918709254	0.614170158	0.001783693	0.091532809
guaB	1.916321685	0.86461492	0.026664976	0.299970731
uspF	1.915849929	1.3544183	0.157209679	0.441668772
dmsC	1.914417715	0.739786605	0.009659183	0.210528636
ychH	1.913331611	2.213849271	0.3874475	0.658855774
racR	1.911144323	1.415741847	0.177040266	0.466641624
yajQ	1.910699155	1.252011708	0.126984149	0.409405752
exuT	1.910597358	1.171972448	0.103050604	0.39523855
yjhP	1.907676207	1.223463359	0.118938968	0.406500994
gcvB	1.906656507	1.584584107	0.228878205	0.513487682
nuoH	1.904704318	1.147887728	0.097052862	0.390380788
yaiE	1.90305564	1.409384501	0.17692802	0.466641624
clpB	1.898789023	1.08736907	0.080772187	0.37803293
flu	1.898135194	0.797133593	0.0172563	0.263454633
plsB	1.896213689	0.735760337	0.009959954	0.21477476
rpoZ	1.887411386	1.368304541	0.167777742	0.456184418
rpoN	1.886055212	1.113330396	0.090252735	0.386361761
dcrB	1.885593424	1.240514866	0.128508782	0.409893472
yqjA	1.885082801	1.120280897	0.092435579	0.387027055
serB	1.884799937	0.850987951	0.026771227	0.299970731

hypB	1.880263843	0.86572412	0.029863413	0.315424071
uxaB	1.879246455	1.46032834	0.198141101	0.485231275
rrsA	1.875872214	1.560982967	0.229470102	0.514246431
glmU	1.875547935	1.009098115	0.063078491	0.36375562
folD	1.875310161	0.690038601	0.006573964	0.170334607
focA	1.874154189	1.129382146	0.097024972	0.390380788
uspG	1.873873741	1.49781836	0.210909396	0.495093626
glgP	1.873435492	1.034740933	0.07021274	0.367589495
mprA	1.873107483	1.273822619	0.141436728	0.427561927
yhfS	1.870802289	1.499951957	0.21230897	0.495978403
cysA	1.869619881	1.452722832	0.198102543	0.485231275
speD	1.867960407	1.147735363	0.103627047	0.395998689
nuoI	1.865638295	1.019580253	0.067278333	0.367589495
rsfS	1.864202924	1.475959728	0.206573162	0.489735437
ilvB	1.864110101	0.783510555	0.017351353	0.263454633
can	1.862920949	1.285630895	0.147328507	0.4313125
fecD	1.8628878	1.435983707	0.194531305	0.482342452
yfeX	1.862113756	0.867924655	0.031914602	0.321852891
lipA	1.859079889	1.115563209	0.095614974	0.390380788
hemE	1.858978934	0.673198421	0.005755183	0.164265687
selD	1.856059879	0.988406527	0.060404362	0.36375562
yicR	1.854887221	1.344160503	0.167599077	0.456004469
thiG	1.85295506	1.405237142	0.187300645	0.475464504
trmJ	1.852225626	0.834086011	0.026373659	0.299970731
brnQ	1.851987366	0.746170086	0.013065061	0.246046435
yceQ	1.851745666	1.444224947	0.199782103	0.485231275
sstT	1.850939487	1.045383443	0.076629858	0.373744519
minD	1.850068106	1.186756683	0.119013445	0.406500994
ibpA	1.846621275	1.34742771	0.170537451	0.460905883
ribA	1.845423185	1.206318171	0.126066725	0.409405752
copA	1.844390178	1.110109677	0.096623382	0.390380788
fdoG	1.843725401	1.439204496	0.200168178	0.485231275
yqiB	1.843432383	1.361227223	0.175658878	0.465671321
rhsA	1.842955733	1.996287051	0.355907288	0.634177488
htpG	1.841905141	1.046672113	0.078446332	0.374816185
plsX	1.841542989	1.06578751	0.084011412	0.381835591
grxC	1.837335621	1.478202983	0.213885456	0.497251883
cmk	1.837229172	1.324717356	0.165477168	0.453568742
map	1.836890625	1.182212458	0.120238406	0.406808855
nfi	1.83587075	0.838779132	0.028615629	0.310222676
dicA	1.827125472	1.340432126	0.172855125	0.462863982
fadA	1.827112913	1.279451378	0.153279183	0.436372057
yciO	1.824907882	1.183276195	0.123012819	0.409405752
dmsB	1.822243128	1.004386421	0.069633873	0.367589495
yceB	1.82198009	1.062179538	0.086286179	0.382674906
dacA	1.821069389	1.163110689	0.117421487	0.405451127
priA	1.820900182	1.156289757	0.115307594	0.403550127
ychA	1.817124909	1.189325898	0.126547	0.409405752
mlaB	1.813514075	1.39349523	0.193116815	0.48030403

malT	1.812319573	0.871222632	0.037506955	0.334793019
aroG	1.811828263	1.160247663	0.118385193	0.405691946
trpS	1.810930427	1.062468148	0.088295891	0.384480713
mnmA	1.806912685	1.044059319	0.083512255	0.381205511
ispE	1.805183194	0.916648643	0.048915268	0.356018847
tesA	1.80226644	0.638134846	0.004738814	0.150754652
ppc	1.802187401	0.87378019	0.039158442	0.337743189
thyA	1.800904563	1.162983971	0.121497021	0.407401922
clpS	1.798679104	1.416390545	0.204119058	0.487965088
yeaO	1.798210799	1.345668818	0.181452801	0.471847116
dcd	1.798195006	0.675494661	0.007766824	0.184929184
mtn	1.796922799	1.121354053	0.109054363	0.39865319
uhpB	1.795779466	1.222834899	0.141958153	0.427561927
bssS	1.793358282	1.77925004	0.313488395	0.599885026
rsmJ	1.793149855	1.374823809	0.192139456	0.48030403
glmS	1.791817997	0.915320103	0.050278589	0.357424257
ibaG	1.791441188	1.471134895	0.223327603	0.50806403
mraZ	1.790917701	1.511899694	0.236196222	0.519106803
yfbV	1.787201279	1.372936143	0.19300643	0.48030403
metJ	1.786449756	1.319459992	0.175760334	0.465671321
ubiH	1.785606356	0.774248642	0.021096997	0.277602331
hflD	1.784431229	0.805718552	0.026780122	0.299970731
uvrD	1.782042766	1.150212756	0.12130583	0.407401922
yidF	1.778190028	0.640391063	0.005490958	0.164265687
yecA	1.77730016	1.07058098	0.09688895	0.390380788
yigA	1.776634383	0.887253963	0.045242086	0.346713453
lepB	1.772389374	1.006777618	0.07833024	0.374816185
galU	1.772310006	1.105070258	0.108758479	0.398282207
yadG	1.771973075	1.051365567	0.09191114	0.387027055
ygfK	1.771970456	1.251285036	0.156740212	0.441176167
yjeI	1.771373365	1.107106965	0.109598138	0.398679812
dsbC	1.77018893	1.215694183	0.145361197	0.430141819
dppF	1.769675858	0.590948145	0.002747706	0.109049815
rsmE	1.76925951	1.035636389	0.087566032	0.383361441
murE	1.768484924	0.715639896	0.013466279	0.24823952
rpiA	1.767831396	1.219248003	0.147076408	0.4313125
yfgM	1.761852466	1.202042048	0.142725627	0.427567449
rtcA	1.760879735	1.390098427	0.205251758	0.489461478
fecB	1.760742347	1.201870475	0.142920441	0.427567449
nagB	1.759548691	0.927491589	0.057814116	0.36375562
yagG	1.759203505	1.794811636	0.32700689	0.614597093
nfuA	1.756846459	1.288845936	0.172845969	0.462863982
usg	1.756242121	0.886256034	0.04751933	0.353745052
yiaF	1.756235639	0.970877962	0.070464221	0.367589495
ybeL	1.755881419	1.381444485	0.203711787	0.487965088
pepN	1.754914943	0.965872521	0.069229072	0.367589495
yhbJ	1.753913706	1.12923123	0.120377104	0.406808855
gldA	1.753349311	0.634966968	0.00575676	0.164265687
yqjC	1.746315785	1.336494472	0.191335348	0.48030403

ychF	1.745212284	1.026445055	0.08908408	0.385449519
emrD	1.744211745	1.209935114	0.149422414	0.4313125
prc	1.743449845	1.143841775	0.127457402	0.409405752
phnN	1.743238052	1.597108342	0.275054502	0.558871656
nagE	1.735536619	0.734249137	0.018093994	0.268142043
gpp	1.734892206	0.878041976	0.048170095	0.353805533
hemY	1.734752614	0.667652413	0.009369014	0.20869221
yjjK	1.734221206	0.752821427	0.021243483	0.277602331
mreC	1.733529721	0.584881187	0.003037664	0.113271675
flgE	1.73137998	0.927624342	0.061976093	0.36375562
hemX	1.729060544	0.823997043	0.035871416	0.32752865
yagH	1.728577365	1.96437199	0.37887861	0.65374791
epd	1.728098702	1.070486462	0.106459857	0.396303494
phnG	1.727736663	1.79210888	0.335005726	0.620079601
puuB	1.72497639	1.532257375	0.26026095	0.54191833
gor	1.7238792	0.628339304	0.006077911	0.164265687
ruvC	1.722791965	0.987947274	0.081192292	0.378772785
rph	1.722226834	0.627537157	0.006061754	0.164265687
fdoH	1.721570895	1.06881386	0.107238513	0.396303494
era	1.721073947	0.871406241	0.048262077	0.353805533
gltA	1.719769268	1.758413832	0.328062921	0.614967825
lptG	1.717836308	0.646137222	0.007846033	0.184929184
ybeX	1.713946127	1.033546686	0.097253873	0.390380788
yieP	1.713841575	1.136877217	0.131682731	0.414795486
yagA	1.713470671	1.668775818	0.304522648	0.590295953
prmB	1.711784391	0.798575517	0.032069178	0.321852891
bcp	1.711747319	1.158291167	0.139455624	0.426425317
yrdD	1.710956609	1.119444545	0.126413794	0.409405752
aroB	1.709208683	0.74744717	0.02221168	0.280517603
ydcA	1.709108877	1.642199221	0.297994407	0.582202085
hypF	1.70669319	1.360939666	0.209822016	0.493398174
gabD	1.706192942	1.585462978	0.281861035	0.564557626
tdh	1.701887446	0.953310302	0.074222437	0.373744519
yhbS	1.701669229	0.668434132	0.010904183	0.223260397
rluB	1.699939667	0.968582991	0.079245796	0.376626561
yciI	1.698130767	1.415682663	0.230328256	0.514941258
parE	1.697598386	0.824864451	0.039586951	0.338654556
bssR	1.696341745	1.713454809	0.322167991	0.609458252
fabR	1.695429363	0.961552041	0.077863116	0.374816185
hemC	1.695246511	0.589672867	0.004041642	0.13666646
gmhB	1.693235303	1.235025635	0.17037113	0.46076355
hycD	1.691352913	1.449360917	0.243224657	0.523887136
hslU	1.690176636	0.905751496	0.062034516	0.36375562
argR	1.688706291	1.268571234	0.183127292	0.472775241
asnS	1.688542196	0.939889837	0.072409944	0.371865892
ynfD	1.688499845	1.158480993	0.144975126	0.429756275
mntS	1.680794221	4.374292666	0.700797862	0.86432447
spoT	1.680490529	0.837873411	0.044892364	0.346511754
hisD	1.68000353	0.887154651	0.058265026	0.36375562

eutA	1.679691338	1.514358864	0.267354027	0.549605773
minC	1.674571489	1.117467992	0.133992775	0.417851314
pepB	1.671866073	0.596683175	0.005079748	0.15483682
murC	1.667415386	0.684665994	0.014876547	0.253499259
yacL	1.666633076	1.154510691	0.148856049	0.4313125
sdhC	1.6660965	1.653220657	0.313556085	0.599885026
glnB	1.665400387	1.374809337	0.225754301	0.510610481
ycbZ	1.664435694	1.010448518	0.099511881	0.393965983
glgC	1.662787828	0.917810164	0.070034054	0.367589495
minE	1.65683472	1.591202902	0.297761081	0.582202085
opgH	1.655229413	0.784386149	0.034839183	0.32752865
metQ	1.654263047	1.075848688	0.124137783	0.409405752
potA	1.652314184	0.984334719	0.09322806	0.387027055
plaP	1.65088646	0.930040242	0.075886343	0.373744519
ygeW	1.648076841	1.149372298	0.151602812	0.433849076
rluD	1.646820958	0.811258138	0.042360682	0.345787635
maeA	1.645547006	1.123748496	0.14310177	0.427567449
dppD	1.644841376	0.611879619	0.007184307	0.179028999
amiB	1.643584577	0.768835718	0.032536403	0.321852891
ycbB	1.641772438	0.770528366	0.033113047	0.324660621
ribD	1.639034032	0.611180153	0.007323757	0.179028999
thiI	1.633617731	0.738264065	0.026912651	0.299970731
exuR	1.633284296	1.133444325	0.14958692	0.4313125
lpoB	1.633124275	0.755955954	0.030746114	0.31710311
dtpB	1.633053442	1.072929251	0.127996208	0.409405752
glpF	1.632543355	1.250604166	0.191755406	0.48030403
mutS	1.632512445	0.797540347	0.040664566	0.339905467
tatC	1.629459192	1.046479689	0.119449996	0.406500994
garK	1.627682104	0.751174974	0.030246434	0.315490476
folE	1.626685816	1.189565919	0.171480639	0.462222414
fdhE	1.624481249	0.906704972	0.073192003	0.37206423
cueR	1.622530933	0.589770211	0.005939117	0.164265687
ribB	1.62076099	1.060907943	0.126584248	0.409405752
hscB	1.619220791	0.884577706	0.067174987	0.367589495
ybeZ	1.618376636	0.79948559	0.042942129	0.346511754
recA	1.615904228	0.72301399	0.025420258	0.295116348
galK	1.615896002	0.760286922	0.033555205	0.324660621
yagI	1.613476117	1.218431933	0.185428851	0.473701881
rlpA	1.612760049	0.627215276	0.010131686	0.21504636
guaC	1.611375814	0.586124138	0.005973988	0.164265687
eutT	1.607338171	1.462163873	0.271642772	0.555507522
yibT	1.606719017	1.504814345	0.285647241	0.569888737
astD	1.60528278	1.532101377	0.294746717	0.578925965
pflA	1.603548356	1.10915657	0.148251067	0.4313125
rpe	1.603239351	0.955405591	0.093333048	0.387027055
araB	1.60037395	1.462873544	0.273957888	0.55726306
priC	1.60026876	0.941769744	0.089278784	0.385449519
dcuC	1.599499727	0.892287434	0.073039547	0.37206423
ssb	1.597823905	0.733600643	0.029401805	0.312860316

glpX	1.597278249	0.645686995	0.01336974	0.24823952
ygjR	1.595625786	0.970030344	0.099985572	0.394894668
yncJ	1.594422	1.43373237	0.266104683	0.548164829
ddlA	1.594137822	0.966452479	0.099050653	0.393168651
argS	1.593549096	0.944705062	0.091637622	0.387027055
secM	1.592723989	0.99173133	0.10827314	0.398282207
dnaN	1.591299726	0.678704048	0.019046733	0.274420156
ubiD	1.591021395	0.84177563	0.058747562	0.36375562
ycdZ	1.590152642	1.360964613	0.242644984	0.523497849
hpt	1.589797641	1.119575642	0.155607622	0.440489098
yhdE	1.589673648	0.767511927	0.038339751	0.336427168
hycB	1.587021893	1.436558552	0.269272809	0.551886738
rsmB	1.585348996	0.964545692	0.100254205	0.395053485
yhbE	1.584514586	1.078404875	0.141747841	0.427561927
ispA	1.581497383	0.835197796	0.058283398	0.36375562
garD	1.580804719	0.891271735	0.076120949	0.373744519
hslO	1.58019819	0.757033531	0.036856001	0.332031619
tldD	1.579662352	0.615245825	0.010242581	0.216267834
hemN	1.57892004	0.672940243	0.018960813	0.274420156
glnG	1.578321662	0.854807861	0.064833396	0.367589495
rrsG	1.577916269	1.358384594	0.245392979	0.525469563
yfbT	1.577281295	0.741027096	0.033295078	0.324660621
ybiT	1.57660121	0.840631354	0.060724443	0.36375562
mlaF	1.575800498	1.025079176	0.124232689	0.409405752
selA	1.575494643	0.835826762	0.059436106	0.36375562
citX	1.573855094	1.412648651	0.265229234	0.547633578
ftsK	1.572231565	1.044979637	0.132437975	0.416330989
yaeP	1.571945998	1.262928505	0.213248193	0.49681679
narJ	1.570953758	1.118256164	0.160073445	0.44356647
fixB	1.569664261	1.48429589	0.290276891	0.5746692
udk	1.568507637	1.20222783	0.192006076	0.48030403
pdxJ	1.566591276	0.605026208	0.009617269	0.210528636
ubiF	1.565694568	0.745437218	0.035696235	0.32752865
dcuB	1.564673153	0.952156085	0.100321951	0.395053485
uvrA	1.562702307	0.884601092	0.077301679	0.374512315
oxyR	1.562486793	0.656383585	0.017291616	0.263454633
pepT	1.56013896	0.975232762	0.1096517	0.398679812
yejG	1.559259573	1.486743571	0.29428166	0.57883171
moeA	1.558197442	0.68386378	0.022695602	0.283101448
maeB	1.556253114	0.932186996	0.095025356	0.389124035
nadE	1.555821714	1.054896991	0.14025112	0.426540165
yhiI	1.555817532	1.236641754	0.20835602	0.491661993
yicC	1.55578221	0.86849994	0.0732381	0.37206423
phoP	1.554195497	1.225620862	0.204766413	0.488882825
srmB	1.554031936	0.671045199	0.020567119	0.277602331
sanA	1.552144121	1.118458378	0.16521223	0.453159932
yfeY	1.549241689	0.999153358	0.121008494	0.407110735
katG	1.546978552	0.768121696	0.044012068	0.346511754
ycfL	1.545910215	1.143666955	0.176466942	0.466641624

xseB	1.544464224	1.146291426	0.177865157	0.467616956
yhbU	1.542075558	0.96177596	0.108854634	0.398282207
ybbN	1.541899346	0.577657382	0.007602579	0.182975376
ydfZ	1.541726272	1.698363442	0.363998852	0.641370025
thiP	1.540433642	1.41631747	0.276757149	0.560426315
gsk	1.540253553	0.896436932	0.085761033	0.382674906
ulaF	1.539051555	1.193670395	0.197278581	0.485231275
yggW	1.538211633	0.620051631	0.013109529	0.246046435
bioC	1.537600474	1.486522463	0.300967551	0.585752498
yjaG	1.537153293	1.057998423	0.146254832	0.430851022
rrsC	1.533225817	1.558967213	0.325367233	0.612651539
yagE	1.532525858	1.569777941	0.328931078	0.615301461
holC	1.531000346	0.552512876	0.005588841	0.164265687
dut	1.529962152	0.87867612	0.081646275	0.379146961
yraP	1.528797365	0.728051592	0.035742252	0.32752865
slt	1.528168195	0.68374036	0.025416534	0.295116348
gph	1.527940569	0.561351065	0.006490809	0.16992893
yrfG	1.527695388	1.03407921	0.139582239	0.426425317
hemA	1.527586985	0.803436351	0.057260538	0.362709717
gpmA	1.52664042	1.08262274	0.15850086	0.443018227
yejM	1.525817469	0.758423919	0.044238211	0.346511754
hrpB	1.524971314	1.168621338	0.191916023	0.48030403
yobB	1.523011904	0.98175093	0.120824516	0.407110735
gutM	1.522371991	0.642227961	0.017766243	0.266133831
bax	1.522034313	0.983508185	0.121729153	0.407842965
yebY	1.52183293	1.254739355	0.225180378	0.510559984
yecS	1.521292106	0.583291781	0.009104233	0.205047566
ispF	1.520186301	0.662533834	0.021761586	0.27830116
flgC	1.518759649	1.013126264	0.133852283	0.417851314
rraA	1.518590202	1.179653118	0.197983026	0.485231275
ftsN	1.518225376	0.706413075	0.031618281	0.320451274
puuE	1.518105646	1.513539499	0.315852723	0.603424571
rsuA	1.516498662	0.97024365	0.118050695	0.405691946
relA	1.513968196	0.87381316	0.083166888	0.381205511
dedA	1.513874058	1.201823929	0.207796687	0.490627705
yigP	1.51292202	0.645800268	0.019144315	0.274420156
glnE	1.512563736	1.070369619	0.157619614	0.442513792
yiaQ	1.510022821	1.597676714	0.344589115	0.626843182
topA	1.508689851	1.126742756	0.180576076	0.47047263
truA	1.508502628	0.688087326	0.028356931	0.309862525
mukB	1.508128525	0.849499242	0.075845933	0.373744519
ycbJ	1.507109475	1.005020483	0.13372301	0.417851314
hldE	1.502714505	0.67585058	0.026186016	0.299881667
rseP	1.501967299	1.06402691	0.158071396	0.442556244
kdsD	1.501410021	0.7539104	0.046426315	0.351679413
lyxK	1.500553959	1.480735945	0.310876829	0.597119553
galM	1.500383439	0.780288712	0.054498039	0.358660795
rrlA	1.499523558	1.778930034	0.39926443	0.666780619
flhC	1.499290379	1.129471311	0.184367571	0.472916736

cysS	1.497593653	0.619517491	0.015633765	0.257639363
ydgA	1.496914574	1.167325609	0.199721524	0.485231275
rlmN	1.495689306	0.865636117	0.084015133	0.381835591
rppH	1.494785929	1.26783335	0.238394906	0.520998895
nikB	1.493595447	1.378090333	0.278446681	0.561735111
lolE	1.492294643	0.878375676	0.089333263	0.385449519
ftsQ	1.490538759	0.94639361	0.115264048	0.403550127
napG	1.489064349	1.260368559	0.237423402	0.519997013
fadR	1.488465135	1.1264654	0.186381708	0.473725042
mioC	1.486585521	1.226207313	0.225380589	0.510610481
envZ	1.485906249	0.679605658	0.028784586	0.31118056
eutD	1.485538212	1.471626083	0.312757155	0.599046731
hokD	1.484460753	1.85416577	0.423358367	0.687464692
argP	1.482538906	0.615673754	0.01604043	0.259075318
purL	1.48098119	1.320269581	0.261978806	0.543532281
cpxA	1.479948176	0.607349729	0.014820682	0.253499259
sseA	1.47983317	1.112463855	0.183442391	0.472775241
gpsA	1.479013094	0.611232159	0.015532293	0.257639363
zwf	1.478683166	1.084686687	0.17280837	0.462863982
rsgA	1.478337363	0.721003696	0.040326578	0.339178316
pcnB	1.477217275	0.610448985	0.015525351	0.257639363
rutD	1.475555014	1.568017481	0.346688334	0.627724209
caiF	1.473275888	1.246851311	0.237365607	0.519997013
ynfB	1.472255239	1.387818836	0.288762128	0.573843954
pntB	1.471619348	0.843012502	0.080868782	0.37803293
fdhF	1.471399413	0.664551477	0.026820246	0.299970731
spy	1.470917449	0.942521886	0.118613702	0.406131712
prmC	1.470727005	0.589006808	0.012526275	0.238410881
gabT	1.469846928	1.537565161	0.339093713	0.62332179
murD	1.469638045	0.633246307	0.020297639	0.277602331
rrsE	1.466803571	2.485169432	0.555041312	0.780158646
hslV	1.466423881	0.705074764	0.03754266	0.334793019
rrsB	1.462999412	2.946926764	0.619577525	0.818965533
dctA	1.461237979	1.957144522	0.455294596	0.709909343
yhfA	1.459354423	1.275172447	0.252442485	0.532458247
ugpQ	1.459091967	1.094008991	0.182298546	0.472440092
ribF	1.456566836	0.819406408	0.075471579	0.373744519
entA	1.456493338	1.21509438	0.230657517	0.515198664
yagF	1.4548625	2.055397389	0.479053684	0.72819034
tsaB	1.454534316	0.668095386	0.029470444	0.312860316
wcaN	1.454338982	0.89193035	0.102984845	0.39523855
fadL	1.454165259	1.095406297	0.184339296	0.472916736
yjbE	1.453547622	1.203302478	0.227060615	0.511182936
mraY	1.45338226	0.815265867	0.074633593	0.373744519
ygbK	1.451840903	1.145633326	0.205054346	0.489282119
fliI	1.451532082	1.373259777	0.290512751	0.5746692
sthA	1.451043843	0.787527773	0.065397608	0.367589495
mrcA	1.450708526	0.898049488	0.106224249	0.396303494
nikE	1.449706101	1.296536343	0.263508208	0.544753837

eutK	1.449433147	0.954971126	0.129070642	0.410716155
thrB	1.448367788	0.628750047	0.021246968	0.277602331
rfbB	1.448004078	0.967260268	0.1343891	0.418122342
ydhR	1.442172941	1.274973037	0.257996191	0.538596601
flgD	1.440986637	0.886248777	0.103962588	0.396150037
yffB	1.440411135	1.196928696	0.228812797	0.513487682
hyaD	1.438820189	1.179349595	0.222460477	0.506375504
ilvE	1.438011809	0.668904538	0.03157094	0.320451274
mdtP	1.437117737	1.445810154	0.320228779	0.607490627
fdrA	1.437070533	1.472258247	0.329015161	0.615301461
yshB	1.435044511	1.933767545	0.458028123	0.712253936
ychJ	1.433981626	1.137240486	0.207333758	0.490627705
hslJ	1.433455143	1.141633743	0.209254735	0.493208545
fre	1.432633371	0.952938354	0.132739754	0.416330989
gutQ	1.432552521	1.018642459	0.159624678	0.443535602
flgJ	1.432013722	1.117324601	0.199967133	0.485231275
recJ	1.428233012	0.700468432	0.04145318	0.343662965
puuC	1.42592337	1.511578143	0.345509984	0.626990813
panD	1.425601121	1.307904061	0.275717962	0.559159889
recG	1.422301239	1.1031251	0.197280565	0.485231275
yciS	1.422004993	1.364461879	0.297331593	0.582153488
ycdX	1.421137633	1.07421259	0.185849502	0.473701881
rlmH	1.420738973	0.698982469	0.042094802	0.345787635
yeeZ	1.419414539	0.961649997	0.139938548	0.426485017
prfC	1.418921881	0.707961586	0.045044819	0.346511754
lptB	1.416576763	0.796280194	0.075240936	0.373744519
tatB	1.415813195	0.635226816	0.025825154	0.297429473
mnmG	1.414458474	0.729049166	0.052362507	0.357424257
flgG	1.413070223	0.738789938	0.055788774	0.359671341
lnt	1.412841699	0.709405243	0.046416478	0.351679413
evgA	1.412786167	1.19463075	0.236962454	0.519829973
dnaG	1.409989693	0.707942239	0.046406909	0.351679413
ydcY	1.408822328	1.242975864	0.257035007	0.53740068
yheO	1.408799208	1.031380297	0.171959134	0.462863982
ygeX	1.408780339	0.602084006	0.019291994	0.274420156
eutE	1.408368604	1.529627151	0.357193117	0.635672036
rutF	1.408241124	1.319979415	0.286032362	0.570376387
ygiM	1.407010368	1.149870803	0.221093821	0.505144582
lacI	1.406230462	1.187378868	0.236288441	0.519106803
purR	1.404061369	0.723079722	0.052163802	0.357424257
fnrS	1.40352733	3.308058361	0.671365078	0.84716196
rsmH	1.398078604	0.712704153	0.049802838	0.357424257
diaA	1.398018102	1.019504525	0.170290152	0.46076355
pka	1.397755561	0.853603093	0.101530809	0.395053485
acpT	1.396509863	1.036799452	0.177998561	0.467664397
miaB	1.394741854	0.815315255	0.087140575	0.383361441
yajD	1.394456304	1.189932994	0.241246039	0.52345551
murI	1.394135911	1.024080798	0.173402024	0.463147989
mlaA	1.388756762	0.941078972	0.140022585	0.426485017

rimO	1.387634937	0.883244613	0.116167489	0.403550127
cysK	1.387016336	0.953235999	0.14565243	0.430375328
yeaY	1.384763149	0.991463938	0.162508214	0.448168911
mtlA	1.384012194	0.732420657	0.058805973	0.36375562
lhgO	1.381883354	1.401958429	0.324289828	0.611759405
yehY	1.378416224	1.589293743	0.385770157	0.657847271
purD	1.378132437	1.13722619	0.225574896	0.510610481
sdhA	1.377851668	0.980653888	0.160011285	0.44356647
dfp	1.377776245	0.714037995	0.053661884	0.357756229
hemL	1.377502707	0.599615142	0.021601011	0.277602331
glmM	1.376828224	0.678356462	0.042391824	0.345787635
yeiG	1.372341597	0.863644132	0.112057499	0.400636634
ygiF	1.371914451	0.830648576	0.098612372	0.392933288
yebG	1.371579459	1.337230248	0.305039222	0.590558265
lpoA	1.371107204	0.708148521	0.052844933	0.357424257
glpD	1.370668948	0.640763448	0.032426072	0.321852891
rlmB	1.370252833	0.572018918	0.016599177	0.262886141
tolR	1.370081469	1.092126274	0.209657313	0.493398174
ratA	1.368906639	1.133089744	0.227001699	0.511182936
rrlD	1.368603716	1.786479835	0.44362296	0.702273064
panB	1.368142695	0.640041489	0.03255049	0.321852891
murA	1.367878607	0.677134276	0.043373069	0.346511754
yehU	1.364183209	0.812644176	0.093211143	0.387027055
hchA	1.363474412	1.006108778	0.175355176	0.465546747
bioF	1.363075258	1.469702006	0.353692894	0.633528487
yejK	1.362710937	0.815809417	0.094844398	0.388800372
ybbP	1.362438798	1.068230814	0.202161997	0.486536435
thrA	1.362129246	1.150443684	0.236411712	0.519106803
iscR	1.362074388	1.186071548	0.250807014	0.530951246
ppsA	1.360377127	0.738688212	0.065531914	0.367589495
sdhD	1.360111493	1.434543112	0.343071037	0.626208907
recQ	1.358831665	0.942928709	0.149563293	0.4313125
dedD	1.358696638	0.703454672	0.053425826	0.357424257
pmbA	1.357777596	0.787825606	0.084807282	0.382674906
fliY	1.354822664	1.075130522	0.207616284	0.490627705
pbpC	1.352475438	1.328923919	0.308809971	0.595583075
msrC	1.352447951	1.038958668	0.19300729	0.48030403
yqjD	1.347994395	0.869158785	0.120921229	0.407110735
rhsC	1.347846153	1.661636144	0.417276081	0.683875756
lptF	1.346675409	0.760465396	0.076584498	0.373744519
mzrA	1.346597355	0.884101492	0.127727359	0.409405752
livG	1.346043626	1.347645094	0.317885939	0.605312164
holB	1.345569857	0.576786022	0.019654692	0.277602331
hisB	1.345007057	0.81705011	0.099727838	0.39443576
glnL	1.344886883	0.70999752	0.058196678	0.36375562
dusA	1.343827468	0.824260705	0.103029076	0.39523855
nnr	1.342834189	1.226036837	0.27340093	0.556688785
aphA	1.342646702	1.074566083	0.211490288	0.495360959
curA	1.342488387	0.769059141	0.080876243	0.37803293

yciT	1.341884154	1.125593921	0.233199871	0.516947839
fldB	1.341101733	1.24127757	0.279954971	0.562128506
hyaA	1.340506663	1.223780313	0.273349411	0.556688785
yehS	1.340229807	1.220086024	0.271998626	0.555507522
hycG	1.339746025	1.346314549	0.319677366	0.607297114
chaC	1.339129257	0.878910597	0.127602671	0.409405752
pgl	1.339085683	0.597886465	0.025110474	0.295116348
guaD	1.338423463	0.914497515	0.143313766	0.427567449
dnaE	1.338297633	0.796390795	0.09286913	0.387027055
tsaC	1.337577494	0.849262675	0.115259494	0.403550127
ispB	1.335978345	0.893904098	0.135033684	0.419033677
hycC	1.335609606	1.512367178	0.377168616	0.653142047
nagZ	1.333926649	0.713852973	0.061674393	0.36375562
glnQ	1.332393695	0.872161372	0.1265891	0.409405752
mrcB	1.331242759	0.640547145	0.037682652	0.334793019
mpl	1.330663808	0.602503791	0.027205487	0.300520559
serC	1.329980071	1.064042434	0.2113246	0.495360959
yqaA	1.328722244	1.069984702	0.214305106	0.497305609
hofN	1.328416895	1.27722486	0.298302421	0.582436515
sdhB	1.32838653	1.228222501	0.279451633	0.561943644
mukF	1.328310136	0.764303392	0.082222148	0.379645316
ulaD	1.328284631	1.266855527	0.294413169	0.57883171
ybiH	1.327774585	0.56722784	0.019241776	0.274420156
ubiB	1.325307929	0.927173036	0.152887194	0.435868274
rnr	1.319711701	0.906216071	0.145312987	0.430141819
yciC	1.318601221	1.046467109	0.207651208	0.490627705
garP	1.318384236	0.833873204	0.11386962	0.403513785
ybgK	1.317601763	0.704524719	0.061456122	0.36375562
ycdY	1.317500866	0.891786136	0.139575647	0.426425317
bglA	1.316111968	0.678882178	0.052544503	0.357424257
bcsC	1.31597851	1.396420462	0.345990868	0.627418179
yfgC	1.31594531	0.65336494	0.043998556	0.346511754
yjjY	1.315795093	1.751988352	0.452634848	0.708213692
rlmJ	1.315691484	0.844320365	0.119165781	0.406500994
mukE	1.314716635	0.859907157	0.126287915	0.409405752
metH	1.313568128	0.986541725	0.183028604	0.472775241
ybgL	1.312679102	1.024846157	0.200244678	0.485231275
yhjJ	1.311994677	0.771320565	0.088948247	0.385449519
gmhA	1.311760008	1.062388226	0.21693192	0.500536144
yhgF	1.31082868	0.929720576	0.158564245	0.443018227
mog	1.307950635	0.569502472	0.021638465	0.277602331
queA	1.301984403	0.582786851	0.025478718	0.295116348
acuI	1.301541569	0.770021108	0.090976811	0.386603764
rpoS	1.298418497	1.39213464	0.350984338	0.63145218
uvrY	1.296909681	1.017517267	0.202457062	0.486809568
glcE	1.293984823	1.518393257	0.394099409	0.664451982
ycfD	1.29347211	0.963582002	0.179479912	0.469426815
ynjC	1.292773961	1.331845521	0.331715826	0.617436161
cptA	1.289465137	1.107449071	0.24427971	0.524806542

sseB	1.288551861	0.970287076	0.18417449	0.472916736
aroC	1.285891563	0.760603262	0.090909283	0.386603764
kgtP	1.285604294	1.713629213	0.453120322	0.708484422
dld	1.284374685	0.913913625	0.15991522	0.44356647
ampH	1.282010785	0.789544004	0.104432229	0.396303494
ilvN	1.281702117	1.371842923	0.350153196	0.631178772
feoB	1.279636193	0.718272573	0.074823169	0.373744519
yheU	1.279442408	1.558458487	0.411665278	0.678686879
visC	1.278731916	0.637520751	0.044878797	0.346511754
hisI	1.27827261	0.619304766	0.039013495	0.337743189
yadH	1.277856761	0.987366016	0.195593667	0.483203367
rapA	1.275460428	0.684948664	0.062585391	0.36375562
potD	1.274876277	0.955528944	0.182135042	0.472440092
yciB	1.273775123	1.028821045	0.215681943	0.499072259
entF	1.27369438	1.402622457	0.363835587	0.641370025
bioA	1.272974847	1.351962746	0.346410076	0.627724209
polA	1.27219224	0.863864627	0.140838566	0.427080912
nsrR	1.272112829	1.198726624	0.288589871	0.573782903
rnb	1.270746194	0.856181348	0.137755301	0.423075749
ynjE	1.268216157	0.647009374	0.04998174	0.357424257
lolC	1.267026925	0.72078068	0.07877242	0.374816185
rnlA	1.266484976	1.116520925	0.256662987	0.536899767
yegQ	1.262430526	0.879842633	0.151333629	0.433573521
mdlB	1.262028546	0.924357666	0.172157751	0.462863982
ygdG	1.261019345	0.77955147	0.105744158	0.396303494
cysW	1.260886002	1.318999085	0.339101776	0.62332179
bglX	1.26084608	0.65828478	0.05544782	0.35965674
yedD	1.259379971	0.613741486	0.040172443	0.339178316
qseB	1.258413743	1.076348894	0.24234304	0.52345551
ygfZ	1.257703767	0.827865654	0.128709041	0.410209475
eda	1.257497959	0.83850943	0.133696654	0.417851314
ygdI	1.257428916	1.362422333	0.356040444	0.634177488
kdpD	1.256825328	1.307757662	0.336525172	0.620658879
apaG	1.25597099	1.0209813	0.218636963	0.502753402
ynjA	1.25576495	1.285610138	0.328675507	0.615301461
cra	1.253933539	0.79538317	0.114907153	0.403550127
metK	1.252858862	0.825915223	0.129283355	0.411070369
ldrD	1.250974482	2.321334977	0.589953758	0.799883279
cysE	1.249788337	0.609442157	0.040295323	0.339178316
argH	1.24970866	1.225980847	0.308034867	0.59493728
ynfH	1.249578688	0.941923617	0.184633019	0.472916736
putA	1.247473171	1.367033146	0.361484236	0.639100346
rcsD	1.247067377	0.996784676	0.21090163	0.495093626
qorA	1.247034135	0.57725037	0.030749431	0.31710311
dnaQ	1.244991944	0.954907348	0.192307588	0.48030403
yidQ	1.243288661	1.174409996	0.289759393	0.574417887
yqgE	1.242313216	1.048391333	0.236028955	0.519106803
nlpD	1.238670717	1.352761446	0.359844797	0.637642927
yhjE	1.237218295	1.242099757	0.319216139	0.606705216

phnJ	1.233580527	1.392904292	0.375823635	0.651942241
ftsI	1.232620094	0.854385478	0.149105605	0.4313125
rrlG	1.231612665	2.117040039	0.560727252	0.783322752
yjjW	1.23126471	0.643406926	0.055663094	0.359671341
glnH	1.23076652	1.231888993	0.317751666	0.605312164
xdhA	1.230463305	0.85649689	0.150825087	0.432426611
aegA	1.230355882	1.057171089	0.244497339	0.524995875
rnt	1.228695689	0.822251321	0.135095465	0.419033677
purE	1.226597973	1.18822276	0.301933328	0.586505852
carB	1.225252684	1.339206947	0.360239328	0.637903575
speG	1.224576094	1.130354534	0.278650545	0.561735111
yqeA	1.224089182	0.803609679	0.127699054	0.409405752
ampD	1.221667107	0.848720321	0.15003086	0.431773005
acnA	1.220648099	1.046534127	0.243464076	0.523887136
pncA	1.21997326	0.806761843	0.130486774	0.412631342
tmcA	1.216905901	1.066398108	0.253813087	0.533415372
recD	1.215604063	1.026893485	0.236504752	0.519106803
thrC	1.213705875	0.787958404	0.123483055	0.409405752
topB	1.213165747	0.714533132	0.089537221	0.385449519
ybbJ	1.212949261	1.036440467	0.241879104	0.52345551
sbmC	1.21289853	1.092195668	0.266777619	0.54899313
lolD	1.212407942	0.760309567	0.110796325	0.399980076
acrR	1.208841275	1.334924398	0.365173891	0.642823404
cysJ	1.208103478	1.27175635	0.342138245	0.625712378
yqiA	1.207415458	1.047580327	0.249084649	0.528408773
glgX	1.207215527	0.661774667	0.06812043	0.367589495
paoB	1.204874187	1.621117031	0.457338195	0.711734519
nrfE	1.202455155	1.345985031	0.371662043	0.648608662
ytfP	1.201809636	1.184891352	0.310449955	0.597119553
napC	1.201017987	0.936545364	0.19970528	0.485231275
recF	1.200598378	0.713045582	0.092227857	0.387027055
rarA	1.199930527	0.587451391	0.041091661	0.341363917
yedE	1.199580958	0.749603585	0.109534904	0.398679812
frmA	1.1987428	0.581284642	0.039185815	0.337743189
yqcA	1.195452869	1.104943474	0.279291124	0.561943644
yiaD	1.194266637	0.826830715	0.148629091	0.4313125
pldA	1.194262541	0.780092943	0.125788325	0.409405752
tas	1.192125476	0.613973503	0.052178731	0.357424257
tsaD	1.192012378	0.898577553	0.184655992	0.472916736
entS	1.191411126	1.342221655	0.37473383	0.651724988
ybeY	1.19140398	1.084221664	0.271830681	0.555507522
ogt	1.190462128	0.74032238	0.107828156	0.397757364
dxs	1.189713079	0.666935232	0.07444788	0.373744519
sodC	1.184451442	1.025320597	0.2480081	0.527782067
slmA	1.184185854	0.993261756	0.23317526	0.516947839
yehT	1.183193909	0.837897642	0.157920994	0.442556244
glcF	1.180625868	1.125679299	0.29426469	0.57883171
glxK	1.179569456	1.441925658	0.413327778	0.680044972
cca	1.17877436	0.587045779	0.044645747	0.346511754

ruvB	1.178271591	0.624921614	0.059366274	0.36375562
yidR	1.177139055	0.773496067	0.128048593	0.409405752
alr	1.175972905	0.757898045	0.120752183	0.407110735
yeiS	1.175591681	1.486819077	0.429133273	0.690456087
speB	1.174994283	0.565780854	0.037823188	0.334793019
ycaO	1.173234774	0.840342866	0.162672883	0.448318062
murG	1.171354904	0.58398701	0.044878731	0.346511754
napA	1.169102779	0.913881275	0.200801269	0.485440792
ilvD	1.167386541	0.829488018	0.159321279	0.443535602
yphD	1.16698907	1.375057378	0.396057203	0.664992388
ribC	1.166834948	1.082724458	0.28117475	0.563740078
radA	1.16570026	1.117685885	0.296967404	0.581878132
alaC	1.165319477	0.637102255	0.06738563	0.367589495
msrB	1.16182395	1.521150356	0.444998463	0.703423195
ubiG	1.161610594	0.596259272	0.051395394	0.357424257
yjcE	1.161118144	0.980100675	0.236138958	0.519106803
ypdF	1.160405624	1.375016864	0.398713546	0.666569049
gntR	1.159715889	0.668268002	0.082669027	0.381205511
dnaJ	1.15914324	0.676143269	0.086465272	0.382674906
pepE	1.158854555	0.663690817	0.08079776	0.37803293
lgt	1.158302885	1.046654416	0.268435869	0.551007095
gstA	1.157184027	0.924654463	0.210760408	0.495093626
cysQ	1.155911097	0.620361794	0.062422118	0.36375562
hemD	1.154078469	0.799849014	0.149056982	0.4313125
ada	1.154060194	1.166981254	0.322698472	0.60989259
holA	1.150587725	0.657825635	0.080277701	0.37803293
ssuD	1.150445031	1.431997076	0.421752218	0.686108945
ratB	1.149612174	1.263230727	0.362792363	0.641133496
yhcN	1.149313157	1.226321746	0.348653838	0.629388959
ytjC	1.14903751	0.54315872	0.034389669	0.327274732
pdxA	1.148356187	0.572442109	0.044849293	0.346511754
ykgR	1.148345183	4.562115027	0.80126265	0.918382466
ybhG	1.148334456	0.650686549	0.077596653	0.374816185
dapB	1.148054034	0.600010529	0.055698168	0.359671341
purH	1.147689286	0.988134971	0.24545075	0.525469563
glmY	1.146174295	1.475931955	0.437408581	0.697582372
eco	1.145879738	1.149719781	0.318929563	0.606705216
yigZ	1.144903651	0.709410134	0.106553206	0.396303494
metR	1.144858307	1.188505496	0.335409251	0.620125741
yrdA	1.14297792	0.944429647	0.226190313	0.510641844
loiP	1.141848571	1.005801586	0.256265454	0.536512635
yccJ	1.14171841	1.226745122	0.352014369	0.63210664
rnc	1.141464423	0.838839544	0.173587615	0.463147989
truD	1.140069558	0.657526538	0.082940067	0.381205511
glpG	1.139606421	0.627468269	0.069340052	0.367589495
kdsC	1.139301137	0.631505232	0.071215	0.369488125
ydiK	1.138851017	0.639579541	0.07497381	0.373744519
yjdM	1.135808739	1.327870345	0.39235123	0.66274662
yheS	1.134307965	0.663741321	0.087458193	0.383361441

cytR	1.13278373	0.956541612	0.236314394	0.519106803
ybiW	1.132554544	0.981498722	0.248539846	0.528082041
yciK	1.132243136	0.691101092	0.101355442	0.395053485
osmE	1.130099086	1.348020375	0.401839908	0.668469015
dsbD	1.124757443	0.739120726	0.128071572	0.409405752
yeiE	1.123549664	0.630155778	0.074590976	0.373744519
yiiD	1.122147089	0.696345862	0.10707525	0.396303494
ivbL	1.121437568	2.272473592	0.62166809	0.820166358
moeB	1.119317069	0.631960441	0.076530412	0.373744519
yciM	1.118806105	0.938672704	0.233299577	0.516947839
ybhC	1.118108478	0.761589067	0.142070083	0.427567449
yhaM	1.117940397	0.924146933	0.226394091	0.510741038
yfcH	1.115364434	0.719778148	0.121238816	0.407401922
rna	1.113837075	0.907099848	0.219480714	0.503631329
metE	1.113322112	1.326210971	0.401202323	0.668145369
yfgJ	1.110711627	1.41453784	0.432329471	0.694124228
lolB	1.109820855	0.935176521	0.235326196	0.519049184
folC	1.10843116	0.74910344	0.138959456	0.426128317
pdxB	1.108397988	0.727679129	0.12770966	0.409405752
yigL	1.107813143	0.732358089	0.130364533	0.412631342
yadI	1.107415494	1.10608145	0.316727178	0.603956717
srlR	1.10514825	0.918897697	0.229096651	0.513693486
livF	1.10479742	1.272522738	0.385287788	0.657847271
paaJ	1.103851386	1.325201562	0.404862397	0.672067326
yjbB	1.103189068	1.006693266	0.273142575	0.556688785
glnP	1.102095251	1.004019142	0.272342715	0.555930194
dcm	1.100746527	0.837125129	0.188539219	0.476558741
recB	1.100359637	0.977854772	0.26047074	0.54191833
rlmL	1.100059881	0.674223489	0.102764767	0.39523855
tatE	1.099234749	1.485608103	0.459347067	0.712996526
yceH	1.098882655	0.813710628	0.17686887	0.466641624
yqjE	1.09877599	0.932369191	0.23860635	0.521180033
gcvR	1.096198275	0.800235736	0.170735544	0.461133841
emrA	1.094018159	0.777827151	0.159574075	0.443535602
manA	1.092924498	0.976937914	0.263257638	0.544753837
uhpA	1.092075249	0.740343615	0.1401877	0.426540165
yheT	1.091766274	0.609041023	0.073037081	0.37206423
mmuM	1.090465418	1.225790749	0.373679844	0.650450016
fhuC	1.090118827	0.98308115	0.26748204	0.549605773
nadK	1.089904171	0.810709035	0.178824279	0.469045144
yjhG	1.089210011	1.074947808	0.310932259	0.597119553
yqeC	1.088518112	0.87321222	0.212556067	0.496086525
parC	1.087984752	0.645672102	0.091980426	0.387027055
nrfC	1.086600486	1.054982569	0.303024049	0.58834267
nagA	1.085859497	0.829756773	0.190653632	0.48030403
plsC	1.084238152	0.911750637	0.234367751	0.517780307
ftsE	1.083255128	0.627712649	0.084398126	0.382635615
pyrF	1.083068159	0.747784963	0.147514029	0.4313125
gltS	1.082684046	0.597991511	0.070212995	0.367589495

rlmG	1.082264125	0.715648006	0.13046124	0.412631342
mscM	1.081541112	1.034362678	0.295740616	0.580315807
fdoI	1.080897355	1.078860848	0.31639786	0.60361267
atpI	1.080470336	1.171639793	0.356431332	0.634594914
kdpB	1.079691102	1.3275438	0.416046194	0.683131336
yihX	1.078885823	0.80759832	0.181575797	0.471864284
yqiC	1.078541942	1.397114723	0.44012741	0.700815601
rof	1.078148497	1.406015134	0.44319363	0.702273064
idi	1.075716088	0.667030934	0.106811232	0.396303494
livH	1.070341278	1.25617939	0.39418032	0.664451982
acs	1.070275971	1.324578504	0.419083394	0.685150556
cyaR	1.069840312	1.826781413	0.558115974	0.782636513
yqjK	1.069803317	1.058526234	0.312182267	0.598669304
exoX	1.064834075	0.890086004	0.231568908	0.51581338
lspA	1.063859588	0.981754168	0.278528288	0.561735111
ybiB	1.062959821	0.565537005	0.060168289	0.36375562
nrdR	1.062172611	1.147417938	0.354598363	0.633836756
rfbC	1.062152171	0.8562065	0.214778423	0.498118836
ptsP	1.062040225	0.6936319	0.125737404	0.409405752
pncC	1.061795591	0.607380667	0.08043718	0.37803293
sibE	1.060636197	1.626385712	0.514308872	0.750179195
ecnA	1.060478819	2.336944585	0.649980987	0.835568019
mdtB	1.060218623	1.25687662	0.398929654	0.666636775
rsmG	1.05659894	0.754541857	0.161417956	0.446072525
yghB	1.05410989	0.784558467	0.1790872	0.46930802
malZ	1.05371364	0.81569156	0.196425021	0.484077226
uvrC	1.05361698	0.796269843	0.185771914	0.473701881
yhbW	1.052796993	0.72812541	0.148205074	0.4313125
corA	1.051929217	0.83381914	0.2071001	0.490627705
glcC	1.051045166	1.082630143	0.331635083	0.617436161
potB	1.050831322	0.845856986	0.214115785	0.497251883
pldB	1.049314615	0.697372453	0.132409232	0.416330989
yggT	1.04897621	0.81094343	0.195829283	0.483373856
ygcO	1.048942023	1.312685399	0.424243419	0.687894336
ypeB	1.046090298	1.343587639	0.436227237	0.696794806
gpt	1.045874166	1.310739945	0.424913131	0.688394721
artI	1.043768709	0.80134372	0.192737625	0.48030403
dtd	1.042639987	0.783226884	0.1831197	0.472775241
moaA	1.041614591	0.684614921	0.128144305	0.409405752
ftsB	1.041213782	1.161331979	0.369949161	0.648130467
yeiP	1.040314468	0.773906012	0.178871187	0.469045144
mltA	1.039608787	0.911525397	0.254071625	0.533682056
rssA	1.039284204	0.876442899	0.235702097	0.519106803
ynfG	1.038555469	0.66173195	0.11654303	0.404162056
ispH	1.038182131	0.551635573	0.059835035	0.36375562
ybhQ	1.037760912	0.785083381	0.186218765	0.473701881
basS	1.036995177	0.819193951	0.205558698	0.489461478
rep	1.036934261	0.637071797	0.103597672	0.395998689
mtlD	1.0369212	0.649604164	0.110436234	0.399980076

yggX	1.036291649	1.286163788	0.420402628	0.685587156
ygcN	1.035690199	0.707867677	0.143436539	0.427567449
purB	1.035477	0.815346896	0.204090553	0.487965088
glcB	1.035152044	0.831758659	0.213303044	0.49681679
ycfH	1.033953494	0.893082806	0.246972072	0.526683209
prlF	1.033127156	1.344198508	0.442141327	0.701542443
ybgC	1.032485457	1.230213029	0.401315731	0.668145369
cdaR	1.031362308	0.595438413	0.083254733	0.381205511
yajL	1.028819373	0.908488698	0.257444671	0.537979741
chbB	1.028616122	1.301125434	0.429201515	0.690456087
murB	1.026712348	0.812822488	0.206537213	0.489735437
sucA	1.025664748	1.444811107	0.477769008	0.726905286
yehM	1.024603679	1.390867079	0.461325842	0.713839114
gudD	1.024498661	0.60563895	0.090722325	0.386603764
pbpG	1.024058505	0.882259757	0.245754882	0.525469563
murP	1.020688141	1.202081919	0.395825473	0.664992388
lldR	1.018823336	0.778879151	0.190851795	0.48030403
insG	1.018806748	1.113997418	0.360427725	0.637903575
tamB	1.018548779	0.920792038	0.268654707	0.551160865
dnaT	1.018381778	0.574736675	0.076409465	0.373744519
yjbQ	1.017988864	1.094909484	0.352502067	0.632320079
yhhS	1.017283129	1.081699344	0.346987269	0.627984994
leuB	1.016859699	1.03832073	0.327416448	0.61460689
fruB	1.014953334	0.809135446	0.209708402	0.493398174
bcsB	1.013333533	1.294308399	0.433676995	0.694746477
yhiN	1.010655764	0.690239491	0.1431365	0.427567449
ade	1.009853955	1.128872561	0.37101774	0.648608662
ndh	1.00952363	0.629158812	0.108590472	0.398282207
cyaA	1.009280447	0.742823013	0.174238762	0.464102458
kbaZ	1.008529601	1.307954283	0.440663043	0.70082364
oppA	1.008131183	1.07887808	0.350084452	0.631178772
gfcA	1.008011552	0.811466663	0.214159296	0.497251883
pncB	1.006772876	0.83875934	0.230018202	0.514905463
yfeD	1.005843414	1.191861369	0.398710469	0.666569049
orn	1.004571215	0.817864346	0.219339676	0.503631329
mnmE	1.002688927	0.623208571	0.107635172	0.397407091
opgD	1.00138465	0.842614987	0.234666043	0.518156937
ybjX	1.000552369	1.039888086	0.335962647	0.620497754
hisH	1.000194071	0.574424384	0.081646595	0.379146961
rfbD	0.998891631	0.853010026	0.241590818	0.52345551
ptsA	0.997847821	1.320780397	0.449949835	0.706193044
ruvA	0.996677691	0.739625903	0.177805422	0.467616956
mrdA	0.994550084	0.676288103	0.141399004	0.427561927
amtB	0.993914858	1.296900612	0.443451988	0.702273064
pcm	0.993907898	0.667437696	0.136450614	0.421705361
yeaP	0.993794225	1.021161922	0.330454182	0.616219529
scpA	0.992709239	1.338847189	0.458411135	0.7125645
prlC	0.992380511	0.654380722	0.129388281	0.411081575
sbcD	0.991914436	0.661884274	0.133971682	0.417851314

yacC	0.991223688	1.273930865	0.436520205	0.696988149
exbD	0.991064971	0.931574685	0.287392162	0.571681954
glrR	0.989243318	0.936849512	0.291002239	0.5746692
sapC	0.988768442	1.099347246	0.368432118	0.647431212
ybiC	0.988471463	0.927625086	0.286607341	0.570400668
nrfD	0.987760547	1.14773359	0.389448296	0.660043224
feoA	0.987407592	1.564250612	0.527887776	0.7567387
ydeH	0.986887349	1.025617812	0.335930595	0.620497754
selU	0.986661374	0.763345436	0.196167091	0.483735637
mtfA	0.986072033	1.112557467	0.375449909	0.651793399
yciX	0.985445698	1.514153523	0.515160854	0.750705284
amn	0.985081836	0.861143639	0.252655657	0.532458247
hisA	0.984050231	0.847044754	0.245338954	0.525469563
argB	0.983324212	1.196672583	0.41123893	0.678686879
waaF	0.982733466	0.683143519	0.150279159	0.431773005
ypdK	0.979797234	3.971407592	0.805130471	0.91948485
ftsX	0.97837689	0.609840889	0.108644635	0.398282207
tyrR	0.977882355	0.630480322	0.120898881	0.407110735
rluA	0.977223233	0.59658944	0.101418291	0.395053485
cmoB	0.976411496	0.758697617	0.198109144	0.485231275
yfgG	0.976245326	1.814342577	0.590527956	0.799883279
xerD	0.975534017	0.674760059	0.148247475	0.4313125
ispD	0.973577973	0.555136187	0.079470919	0.377254222
ssuB	0.969961585	1.195948732	0.417343024	0.683875756
sucB	0.968845138	1.238370771	0.434006174	0.694889822
amiA	0.968707738	1.032579036	0.348170457	0.629283563
ycgM	0.968019099	0.781732404	0.215604909	0.499072259
yhaL	0.967221351	1.791531144	0.589276128	0.799302528
flgH	0.967216906	0.702126005	0.168340847	0.45710234
dmsD	0.966340346	0.724364877	0.182186845	0.472440092
mdtN	0.965034602	1.375971223	0.483085894	0.730927125
yihU	0.964805327	1.194506152	0.419261998	0.685150556
insB1	0.964201313	1.10385464	0.382398363	0.655677468
fucO	0.961997368	0.605907535	0.112354949	0.400636634
ybgI	0.960784729	0.604144247	0.111761928	0.400636634
dam	0.960540402	1.568851747	0.540367761	0.768962727
lpxK	0.960108945	0.638231519	0.132497174	0.416330989
xerC	0.95939251	0.634014596	0.130228214	0.412631342
ispU	0.958239507	0.964464117	0.320443924	0.607614438
yaaA	0.958104766	0.629705703	0.128131409	0.409405752
rsmC	0.957699149	0.680720992	0.159460187	0.443535602
uhpT	0.957003549	1.012197531	0.3444184	0.626843182
fklB	0.956545825	0.566640722	0.091392162	0.387027055
yraQ	0.956533276	1.007548176	0.342433844	0.625712378
purK	0.956113851	1.064400972	0.369044432	0.64755889
cydC	0.955479113	0.652859878	0.14332279	0.427567449
wrbA	0.951468031	0.590158709	0.106913206	0.396303494
fieF	0.950860055	0.814927457	0.24328981	0.523887136
kdpE	0.950098521	1.250697041	0.44746071	0.705015824

wecE	0.9497181	0.777541732	0.221920674	0.505721241
cstA	0.948940859	1.192500938	0.426173343	0.688805982
proW	0.9488558	1.091559622	0.384701588	0.657495885
ydbC	0.948398489	0.656896127	0.148807295	0.4313125
ynjB	0.946385245	1.041377071	0.363464953	0.641370025
frlD	0.946381629	1.115315161	0.396141247	0.664992388
rimL	0.943827225	1.072464589	0.378829818	0.65374791
ilvI	0.943130758	1.055292418	0.371474349	0.648608662
mak	0.942965215	0.872092494	0.279578168	0.561943644
nagD	0.942071429	0.920170581	0.305929346	0.591434224
fdhD	0.941243128	0.802888148	0.241067958	0.52345551
rnd	0.941230063	0.602594589	0.118296858	0.405691946
nrdI	0.94063447	1.271063579	0.459277356	0.712996526
ynhF	0.93927398	3.846836048	0.807100767	0.91948485
lpxB	0.93893017	0.696330437	0.177530591	0.467343516
xdhB	0.936440739	0.811243496	0.248365894	0.528082041
yciV	0.936153669	0.574620017	0.103277045	0.395732645
phoU	0.935687048	0.644072597	0.146288616	0.430851022
yegD	0.935675255	0.772691723	0.225922399	0.510610481
citE	0.935324354	1.281678152	0.465533604	0.717094577
dapE	0.931152748	0.576703578	0.106394659	0.396303494
wbbI	0.930169566	1.059039375	0.379773107	0.654070987
wecB	0.928706008	0.561123879	0.097907875	0.391052732
pntA	0.926441504	0.797718924	0.245494176	0.525469563
nfrA	0.926437219	1.176263745	0.430924798	0.692143079
gtrA	0.925044099	1.079144278	0.391333531	0.661769773
yjgB	0.924151052	1.040987912	0.374667991	0.651724988
dapF	0.924124565	0.642135927	0.150110391	0.431773005
ppiC	0.92286637	1.187322944	0.437001545	0.697481993
ygiQ	0.922317106	0.641484244	0.150494765	0.432086244
ymdB	0.922221672	0.552765242	0.095240604	0.389611916
norR	0.921448536	1.306693998	0.480701053	0.728900347
rcsF	0.921079894	0.706683044	0.192443445	0.48030403
xseA	0.920317561	0.715902672	0.19860558	0.485231275
zapD	0.915275086	0.706239235	0.194980833	0.48257161
ypfG	0.914160938	1.134214708	0.420251042	0.685587156
ligA	0.913860871	0.762001662	0.230415254	0.514941258
yqjH	0.91366978	1.009303812	0.365334268	0.642823404
waaM	0.912972225	0.705230633	0.195467724	0.483186679
ushA	0.912527427	0.73937048	0.217130211	0.500708689
kefC	0.912010568	1.317027619	0.48863819	0.734075985
tmk	0.911657367	0.966179166	0.345389592	0.626990813
qorB	0.911656647	1.180261058	0.439866013	0.700674584
yjjG	0.909356148	0.562652848	0.106052379	0.396303494
ccmC	0.907585189	0.760469629	0.232691788	0.516947839
ymjA	0.907033001	1.396420636	0.515988503	0.750820131
yfiH	0.905175263	0.643934002	0.159814502	0.44356647
rimJ	0.904208062	1.044927624	0.386857247	0.658678346
ilvY	0.903282032	1.056404399	0.39252166	0.662758354

aroE	0.903116144	0.706325033	0.201033482	0.485691142
hyaF	0.902554976	1.140147925	0.428586847	0.690456087
pdxH	0.901628881	1.120280243	0.420921016	0.685857636
glcD	0.901014849	1.405831777	0.521579677	0.753939132
rluC	0.900531025	0.782379683	0.24972592	0.529475156
ydfH	0.897584335	0.853905542	0.293188883	0.577598508
sixA	0.897273586	0.965865017	0.352897143	0.632469061
kdgK	0.896763137	0.697565923	0.198596519	0.485231275
ldrA	0.896102494	1.944391289	0.644895241	0.834339918
ybjL	0.895217948	0.678395334	0.186964927	0.474909657
hofM	0.893903864	1.208598297	0.459530406	0.712996526
yicH	0.893836955	0.798799182	0.263150462	0.544753837
ybfE	0.891886286	1.219142563	0.464431986	0.716713845
kefF	0.891460804	1.261231061	0.479679593	0.728322498
trkA	0.890917575	0.685384201	0.193641921	0.481314745
trmH	0.889571365	0.544243057	0.102151559	0.395053485
ftsY	0.888467796	0.758075712	0.241195435	0.52345551
eutB	0.888466861	0.652734736	0.173467755	0.463147989
yidA	0.887813999	0.654779884	0.175131982	0.465410268
xdhC	0.887776333	1.044164113	0.395198994	0.664992388
leuC	0.887675607	1.25543358	0.479524866	0.728322498
fadH	0.887104781	1.285300975	0.490073292	0.735563541
tolA	0.886905807	0.701644499	0.206216134	0.489461478
yacH	0.885780674	1.165495306	0.447252409	0.704961613
dxr	0.885369051	0.691701468	0.200549624	0.485440792
yihQ	0.884865215	1.341822944	0.509606808	0.746907447
rbsR	0.883959121	0.933357971	0.343601043	0.626612068
gntX	0.883772871	0.544823361	0.104776085	0.396303494
yecF	0.883418324	1.528881643	0.563385681	0.784867886
yciZ	0.883377372	1.503252234	0.556771174	0.781639879
gloB	0.881984854	0.815708118	0.279585676	0.561943644
cysB	0.881922104	0.73649841	0.231130104	0.51581338
pgpA	0.881844739	0.988263124	0.372222637	0.648973612
yghU	0.879531189	0.820284158	0.283617885	0.567235771
yqeB	0.878907579	0.765897107	0.251153111	0.531406426
wecD	0.87890649	0.902414631	0.330081498	0.61580782
dnaB	0.877564262	0.683682711	0.199287513	0.485231275
frvB	0.876752245	1.35865491	0.51872696	0.751849157
fucI	0.874432068	0.620937738	0.159058725	0.443535602
panC	0.87437244	0.831923499	0.293247083	0.577598508
apaH	0.874079694	0.618973587	0.157907633	0.442556244
mutY	0.870752677	0.792544906	0.271907978	0.555507522
proC	0.86933867	0.650167208	0.181189949	0.471466017
gltI	0.868304597	1.167470218	0.457028448	0.711734519
stfR	0.867769484	1.485801149	0.559192413	0.783322752
pdxY	0.86739346	0.648053706	0.180746785	0.470614942
mlc	0.866142697	0.665317155	0.192967894	0.48030403
gspH	0.864386656	1.362303432	0.525752111	0.755815269
dinG	0.8642659	0.680444893	0.204031822	0.487965088

trkH	0.863213736	0.623378478	0.166133641	0.453730509
dsdA	0.861694702	0.735886197	0.24161409	0.52345551
panE	0.860705956	0.642334716	0.180256765	0.470245126
yggF	0.858070942	1.286432725	0.504762046	0.744381716
ycjQ	0.857597163	1.338338117	0.521657441	0.753939132
dgoR	0.85600616	0.918725217	0.351474777	0.631595188
cueO	0.855174301	1.054069906	0.417189387	0.683875756
ulaE	0.854554703	1.27646641	0.503196344	0.743875155
yfjR	0.85402775	1.193965212	0.474431743	0.725120962
relE	0.850776281	1.331424834	0.522824978	0.754366262
zraR	0.850089117	0.991648374	0.391307544	0.661769773
asmA	0.849248172	0.766347934	0.267785935	0.54995146
ygdR	0.848971314	1.380051861	0.538440156	0.767523344
wecC	0.846313061	0.585994028	0.148672894	0.4313125
thiL	0.846036614	0.560947241	0.131496056	0.414529559
aroP	0.844564542	0.620193347	0.173268524	0.463147989
cfa	0.844098192	1.02036034	0.408092544	0.676096107
nagC	0.843334699	0.573437207	0.14138142	0.427561927
yihV	0.842323028	1.305883348	0.518913158	0.751849157
gss	0.840246485	0.649006129	0.195435306	0.483186679
bolA	0.840092837	1.279749765	0.511534074	0.748649507
yddM	0.840003423	1.45622752	0.564050728	0.785254688
entC	0.837665453	1.155674945	0.468557612	0.720065413
yphG	0.836801905	1.424195201	0.556826831	0.781639879
ybhP	0.836764472	1.352430311	0.536106294	0.766082099
ybaL	0.836269744	0.63638321	0.188813311	0.476915367
exbB	0.832759243	0.731778827	0.255123371	0.535336515
nepI	0.832295021	1.100504724	0.449478445	0.706193044
ung	0.82898208	0.702356038	0.237885872	0.520728578
anmK	0.828484029	0.62681459	0.186255747	0.473701881
rsxC	0.82805471	1.058937213	0.434233549	0.694979395
paaG	0.828012034	1.209729696	0.493684506	0.739555949
paoC	0.827889732	1.719312814	0.630144396	0.826632681
ybdH	0.826465521	0.967855118	0.393152363	0.663546912
hrpA	0.826302429	0.853985871	0.333252543	0.618592404
mazE	0.825990996	1.286307122	0.520781408	0.75321007
epmB	0.825387024	0.69991251	0.238289998	0.520998895
uidA	0.82537942	0.661877456	0.212387397	0.495978403
ysaA	0.825122393	0.947691793	0.383936911	0.656812179
amiD	0.823919352	0.841635138	0.327604313	0.61460689
hemF	0.823696474	0.735061556	0.262465989	0.544264512
recO	0.820267129	0.705654103	0.245064508	0.525469563
eutC	0.820122881	1.160294377	0.479676411	0.728322498
nanT	0.817723764	0.80692765	0.31087903	0.597119553
asnA	0.817093275	0.705048252	0.246489515	0.526484981
pdhR	0.816695848	0.575473277	0.155848732	0.440489098
ytjB	0.815054285	0.649121718	0.2092515	0.493208545
maa	0.813525209	0.948279122	0.390949693	0.661757853
narL	0.812560526	0.829323521	0.327191199	0.61460689

hisC	0.812509596	0.697315089	0.243939175	0.52463099
modE	0.811712984	0.694437065	0.242452338	0.52345551
mgrB	0.811577249	1.768050409	0.646217062	0.835020719
ybhA	0.811422011	0.552451309	0.141896116	0.427561927
ylbF	0.810355739	1.32044229	0.539413435	0.768640445
ybgJ	0.810132058	0.843475467	0.336819214	0.620665952
amiC	0.80874689	0.59089539	0.171099226	0.461808429
galT	0.808730494	0.639411351	0.20594134	0.489461478
yafD	0.80776294	0.961543231	0.400869537	0.667951132
queF	0.805546842	0.870550664	0.354794161	0.63390223
eutJ	0.80505154	1.343160808	0.548925559	0.775111187
rdgB	0.80059704	0.668852361	0.231317765	0.51581338
ndk	0.800360399	1.011708291	0.42888681	0.690456087
ppk	0.800196716	0.922830032	0.385880812	0.657847271
syd	0.799972556	0.718149185	0.26530645	0.547633578
ycjX	0.797655081	1.002846055	0.426385888	0.688805982
ygaH	0.797017199	0.766561944	0.298465511	0.582436515
uvrB	0.796850824	0.627201198	0.203911292	0.487965088
matP	0.796525451	0.912059179	0.38248514	0.655677468
rng	0.796302018	0.583624901	0.172439442	0.462863982
pptA	0.79435426	1.450691195	0.583987533	0.796190372
tsgA	0.793968748	0.751199261	0.29054128	0.5746692
holD	0.793114489	0.595921788	0.183220713	0.472775241
fucR	0.792659645	0.830461483	0.33984026	0.62357939
ygjK	0.792499941	1.365030793	0.561528314	0.783626776
queC	0.791805294	0.575762546	0.169060491	0.458645071
lldP	0.791391775	0.824517285	0.33714362	0.620759427
yafV	0.790651528	0.615662215	0.199061695	0.485231275
nrdG	0.790201363	0.987522145	0.423603059	0.687464692
wcaI	0.7901494	1.452086797	0.586339976	0.797658477
rlmA	0.788162061	0.845456151	0.351216163	0.63145218
sbcC	0.786816173	0.858747985	0.359542911	0.637614594
mscK	0.785885834	0.688330734	0.253567484	0.533415372
tauA	0.785249946	1.349458947	0.560634148	0.783322752
yeiR	0.785174538	0.666737043	0.238941157	0.521630291
phr	0.784503296	1.025301927	0.444185989	0.702586812
ccmH	0.784281189	0.591721898	0.185031242	0.473439107
ycjG	0.783641889	0.61258063	0.200810116	0.485440792
yaiZ	0.782594325	1.534529652	0.610058674	0.811904322
ynfF	0.782506308	0.82322131	0.341836999	0.625712378
znuA	0.781600475	0.947833192	0.409588401	0.677660966
dcyD	0.780106392	0.61342782	0.203473801	0.487965088
nirB	0.779522703	0.880909634	0.376207036	0.652087587
sugE	0.779352389	0.777862461	0.316384448	0.60361267
ftsW	0.777628357	0.788084516	0.323773947	0.611209405
yjbJ	0.776205851	1.488456889	0.60202993	0.805646453
ybhF	0.77558376	0.732316789	0.289562293	0.574307993
sbcB	0.77471002	0.757475736	0.306424996	0.592110073
mlaE	0.774648538	0.742692276	0.296935513	0.581878132

qmcA	0.774521011	0.755579825	0.305330909	0.590840815
hyaB	0.767675403	1.158760546	0.507652747	0.746201681
hisG	0.766075213	0.70188992	0.275076513	0.558871656
yebK	0.764337367	0.652274315	0.241275931	0.52345551
yggR	0.763875883	1.120025645	0.495228567	0.739780307
fadI	0.760490391	0.672822659	0.258350498	0.538761791
uup	0.760090613	0.679709569	0.263456851	0.544753837
panM	0.759818576	0.749608166	0.310763635	0.597119553
ftsP	0.758969062	0.869877679	0.382935175	0.655859399
ytfT	0.758578251	1.324518556	0.566834302	0.786799618
ygiW	0.758549187	0.819076584	0.354392515	0.633836756
ydbK	0.758267326	0.782829975	0.332733191	0.618477926
dacB	0.757757452	0.633023277	0.231288952	0.51581338
lhr	0.757510894	1.261751563	0.548263316	0.774715749
recC	0.756716359	0.792218302	0.339483307	0.623508927
trmA	0.756046774	0.782623771	0.334023587	0.619173123
yecD	0.755999181	0.595497026	0.20425339	0.487965088
hda	0.755713379	0.837647547	0.366958394	0.645121132
trpD	0.753365521	1.218926385	0.536538017	0.766428866
wecA	0.753250005	0.869989374	0.386591525	0.658505059
mfd	0.752534053	0.904015347	0.40516343	0.672067326
hyfB	0.750517817	1.427403269	0.599032443	0.803067964
ypfJ	0.747199419	0.638416305	0.241841942	0.52345551
surE	0.746020144	0.715925715	0.297395134	0.582153488
cyoA	0.745601611	1.217883876	0.540398325	0.768962727
yneM	0.745172686	4.160706049	0.857861025	0.944479745
nfo	0.744590252	0.779038731	0.339182997	0.62332179
rffH	0.744581295	0.661771579	0.260532517	0.54191833
yihT	0.743801147	1.214062718	0.540104727	0.768962727
lysP	0.740005922	0.601961386	0.218950139	0.503188131
ygeV	0.739928463	0.93007751	0.426290325	0.688805982
yjhU	0.739235472	0.757392623	0.329051201	0.615301461
ltaE	0.738093433	0.784186399	0.346591142	0.627724209
hemH	0.736006155	0.794365325	0.35416875	0.633836756
yhhN	0.735114208	0.600869506	0.221172085	0.505144582
rhaB	0.734782524	1.253249249	0.557672597	0.782285366
btuC	0.733924267	0.984243527	0.455864701	0.710524989
ynaJ	0.733040269	1.512957399	0.628025245	0.825261896
trmI	0.732146674	0.551855998	0.184608348	0.472916736
yggS	0.730604315	0.773578499	0.344940995	0.626843182
yhbV	0.728512275	0.801807199	0.363567725	0.641370025
kdgR	0.727711446	0.885854575	0.41137315	0.678686879
ilvC	0.726511614	0.765484461	0.34257619	0.625712378
yrfF	0.724832302	0.689347602	0.293040131	0.577598508
ydhC	0.723894488	0.597846976	0.225958363	0.510610481
ccmF	0.722353005	1.077242523	0.502502493	0.743875155
flgA	0.722206301	0.617105494	0.241875216	0.52345551
argF	0.721937712	1.344563668	0.591315381	0.79991618
argE	0.720179471	0.590193242	0.222372579	0.506375504

hybF	0.719136751	0.756340952	0.341700694	0.625712378
yihP	0.718872457	1.283306689	0.575362133	0.791105179
degS	0.718859814	0.566252865	0.204261656	0.487965088
ryfA	0.718115856	1.400210624	0.608047339	0.81060361
argK	0.717675537	1.132704283	0.526345299	0.756399802
yfcJ	0.717427771	1.005039801	0.475332177	0.725525846
gudX	0.716780292	0.59420687	0.227709268	0.511998543
gtrB	0.716247173	0.913683349	0.433091856	0.694678843
yhfK	0.716069712	0.625628328	0.252391104	0.532458247
ghrB	0.715823835	0.665812505	0.282324036	0.564926772
lysC	0.715170998	0.748893	0.33959243	0.623508927
ansA	0.713866939	0.798403986	0.371259052	0.648608662
fic	0.712751603	0.823954576	0.387018209	0.658678346
moaC	0.712573858	0.656455497	0.277706052	0.561506402
yggL	0.711563327	1.259588715	0.572130086	0.789485959
yceM	0.711544581	0.718585934	0.322075836	0.609458252
paaD	0.711016697	1.360278477	0.60118348	0.804886997
xthA	0.71068637	0.691163238	0.303833761	0.589632393
rhaA	0.710519065	1.288435536	0.581319229	0.794025658
ygiD	0.708956707	1.021716572	0.487752549	0.733917973
cydD	0.708040885	0.731300429	0.332947356	0.618562032
fadE	0.707999117	1.149764124	0.538041242	0.767494438
potC	0.707753885	0.811093785	0.382885545	0.655859399
wzzE	0.707514096	0.579251005	0.221923554	0.505721241
yigB	0.707158525	0.873604495	0.418243605	0.684521428
bdcA	0.706799998	0.943784838	0.453917779	0.709031176
ydiH	0.706533313	1.5149919	0.64095704	0.832453872
rstA	0.706492932	1.015544853	0.486630062	0.733109726
yqhD	0.706395773	0.61719205	0.252403262	0.532458247
ylaC	0.705127382	1.162047734	0.543985466	0.771359594
purP	0.705006188	0.61084308	0.248437572	0.528082041
ynhG	0.704969552	0.696883351	0.31172773	0.598080557
yhjK	0.704815149	0.66534241	0.289450904	0.574307993
torS	0.704275608	1.030038231	0.494141035	0.739751757
fepB	0.702636355	0.939322973	0.454445535	0.709130947
yaaJ	0.702555347	1.014745977	0.488720051	0.734075985
ybbA	0.702226492	0.648156156	0.278621255	0.561735111
gltJ	0.702011349	0.672053536	0.296218733	0.580972784
trmL	0.701331915	0.654923819	0.284232251	0.568184194
nagK	0.701317574	0.721073744	0.33075128	0.616489972
paoD	0.700816315	1.421909181	0.622104477	0.820166358
nhaA	0.700654689	0.81308745	0.388840959	0.659841461
inaA	0.700495196	0.832030574	0.399838106	0.666780619
yahG	0.69937802	1.580117998	0.658046938	0.838465291
mepA	0.698750624	0.571660216	0.22158736	0.505523443
queE	0.697659689	1.001231232	0.485926862	0.732560474
lplT	0.697654174	1.111795914	0.530330217	0.759702721
yggD	0.697606471	0.981153273	0.477080135	0.726825579
aidB	0.697252452	1.263498756	0.58105619	0.794025658

eutL	0.696544913	0.954182811	0.465395655	0.717094577
rtcR	0.694414579	1.296993388	0.592370988	0.800235515
hyfD	0.694027361	1.406199839	0.621625311	0.820166358
dinF	0.693768873	1.087820538	0.523629654	0.755174179
chrR	0.693635151	0.872223398	0.42646843	0.688805982
mglB	0.691395087	0.747425898	0.354947721	0.63390223
ftsL	0.691188134	1.047558367	0.509376593	0.74684004
trpB	0.689872656	0.911010009	0.44889331	0.706193044
ybcW	0.688761061	1.589483879	0.664779611	0.843510655
glgB	0.687302156	0.650518093	0.290718929	0.5746692
yfhM	0.686819074	0.928685461	0.459566723	0.712996526
yoaB	0.686100654	1.008221698	0.496184287	0.739780307
yeaC	0.6857081	1.519730504	0.651842708	0.835727495
rsmF	0.685561421	0.683716267	0.316006251	0.603433511
csdL	0.684970762	0.587225191	0.24343122	0.523887136
yejF	0.68394578	0.585034666	0.24237591	0.52345551
cspI	0.68376975	1.492261612	0.646801308	0.835020719
yhhX	0.682827252	0.696361084	0.32680734	0.614506937
lipB	0.681412926	0.873377354	0.435270525	0.696089431
waaN	0.681213185	0.781480573	0.383374456	0.656334478
mazF	0.679455262	1.075392986	0.52750422	0.7567387
ampE	0.67782671	0.72713529	0.351239434	0.63145218
lldD	0.676841714	0.986376134	0.492593127	0.738525346
macB	0.675697086	0.851498594	0.427464032	0.689757885
ybbO	0.674374333	0.58160301	0.246248484	0.526247418
mdtC	0.672477505	1.329741158	0.6130528	0.813666378
hisJ	0.672147773	1.002779572	0.502676339	0.743875155
dusC	0.67081374	0.998522781	0.501707116	0.743795999
fryA	0.670784661	1.360201051	0.621905818	0.820166358
hemG	0.669826908	0.682950328	0.32669916	0.614506937
fucU	0.669691458	0.913960639	0.463719733	0.716098063
tauC	0.668295083	1.55741669	0.66784691	0.844991997
yhfG	0.667360626	1.55047628	0.66688798	0.844612647
dadX	0.667232281	0.925835754	0.471105775	0.722611734
nanR	0.666675221	0.674614138	0.323039081	0.610251834
lplA	0.666237773	1.068949964	0.533111209	0.762877812
patA	0.66501205	1.248748456	0.594350079	0.801295384
yoaA	0.664523485	0.676076195	0.325650693	0.612900608
nudL	0.66240611	0.656291104	0.31282238	0.599046731
cysI	0.662327189	1.258457828	0.598679323	0.803067964
napF	0.662290253	0.606908947	0.275162266	0.558871656
rbbA	0.661374263	1.385327027	0.633067477	0.828909368
cysG	0.659480439	0.624139628	0.290683568	0.5746692
flgI	0.659214548	0.704808296	0.349628343	0.630793638
ydiJ	0.659172454	0.736657237	0.370885622	0.648608662
nikR	0.659112326	0.775707782	0.395496613	0.664992388
ymbA	0.658711498	0.621235302	0.288996641	0.574028604
sucD	0.658473148	1.181775915	0.577397953	0.791871212
fsaB	0.656789356	0.55094642	0.233217676	0.516947839

nhaB	0.656277842	0.619272112	0.289255247	0.574260907
yeaK	0.656269003	1.108193801	0.553718703	0.779435981
yqeG	0.655214189	0.857976768	0.445061789	0.703423195
nrfA	0.653366707	0.591753686	0.26954136	0.552157996
yecC	0.652959602	0.619503892	0.29188107	0.576088538
atoD	0.652660094	1.222633912	0.593469664	0.800640937
artQ	0.652249012	0.741600908	0.379122184	0.65374791
fadD	0.65214965	0.68988766	0.344506593	0.626843182
hslR	0.651518379	0.648246926	0.314874404	0.602123035
yfaQ	0.650400774	1.234362032	0.598254359	0.803067964
yggE	0.649725224	0.670606379	0.332613909	0.618477926
argI	0.647854418	1.188806949	0.585779766	0.797370552
yneG	0.647685534	0.990068808	0.512994335	0.749974408
yibL	0.647197847	1.221445917	0.596207575	0.802465308
gsiA	0.646949921	0.997666436	0.516685436	0.750963463
zraS	0.645840677	0.745736559	0.38646603	0.658505059
ybiA	0.64468301	1.143550979	0.572920626	0.789593837
sapA	0.643879894	0.670162995	0.336662278	0.620658879
hisF	0.64258929	0.626373746	0.304944394	0.590558265
flgL	0.642317369	0.574286177	0.263369651	0.544753837
cpdB	0.64122952	0.753628828	0.394849416	0.664992388
allR	0.640461513	0.731564951	0.381319457	0.654882068
gstB	0.638206124	0.96828012	0.509823136	0.74695446
citF	0.63777368	1.336880878	0.633318711	0.828909368
folP	0.637715552	0.572715214	0.265495876	0.54774569
ygfQ	0.637196299	0.606401701	0.293358492	0.577598508
ysgA	0.636967881	0.597694688	0.286555758	0.570400668
proB	0.636478531	0.753677862	0.398391859	0.666569049
yjaH	0.636445073	0.550580078	0.2477001	0.527403468
fsaA	0.63613417	0.62973767	0.312419883	0.598841705
hcp	0.635597245	1.215780749	0.601120842	0.804886997
ubiA	0.634735818	0.640633233	0.321785986	0.609304244
yecN	0.632437996	1.025175596	0.537296073	0.766803996
aroF	0.631402133	0.5820505	0.278015002	0.561570911
glf	0.630524359	0.950609485	0.507148482	0.746201681
cybB	0.630062674	0.788587192	0.42430401	0.687894336
abgA	0.629963009	1.220100531	0.605630528	0.809237363
yiaR	0.629586928	1.33425813	0.637024641	0.830919529
yoaK	0.629567804	2.380961088	0.79145822	0.913602398
rlmF	0.629351068	0.608668198	0.301145264	0.585769243
acrZ	0.628370847	1.853165164	0.734549626	0.883485372
thiQ	0.627450452	1.22650387	0.608947496	0.810996435
phnD	0.627018728	1.32056905	0.634922762	0.829512368
panF	0.626881898	0.812977181	0.440651164	0.70082364
ycjF	0.626422112	1.156890281	0.588183234	0.798660503
yjfP	0.626043986	0.785966704	0.425725578	0.688805982
epmC	0.625628431	0.595116583	0.293134432	0.577598508
xylB	0.624814648	1.070700369	0.559518427	0.783322752
proP	0.624349868	0.680136481	0.358630737	0.636275276

yncE	0.624256033	0.570592155	0.273933291	0.55726306
pfkB	0.62311268	0.677715395	0.357870075	0.636177042
wbbK	0.622640051	1.021728273	0.542260089	0.769990334
ydjN	0.621701798	0.59833805	0.298782535	0.582618757
ghrA	0.620465161	0.758381977	0.413275346	0.680044972
melB	0.619796471	0.694761098	0.372339261	0.648973612
polB	0.6174306	1.019777546	0.544875944	0.772082165
yebV	0.615852323	1.297874485	0.635137422	0.829525486
cusA	0.615267231	1.36976261	0.653303256	0.836541819
ebgA	0.6145153	1.32551544	0.642930926	0.833030199
yihM	0.613158561	0.806538462	0.447114281	0.704961613
entE	0.612412519	1.148390612	0.593840509	0.800874726
citG	0.612192751	1.344536851	0.648879986	0.835484773
cmoA	0.612032777	0.96429412	0.525627274	0.755815269
menA	0.611679698	0.56491997	0.278909501	0.561943644
folX	0.610799285	1.119576378	0.585366463	0.797370552
msrA	0.610523584	0.571525125	0.285414172	0.569812147
fiu	0.60973433	1.149977546	0.595963484	0.802403176
yqaB	0.607951042	0.736976353	0.409413537	0.677660966
basR	0.607701352	0.6927138	0.380336619	0.654569964
tsaA	0.60768745	0.67598492	0.368670779	0.64755889
nfrB	0.606807036	1.21052663	0.616177423	0.815584287
yqgF	0.605954902	1.0177302	0.551577215	0.777560072
ampG	0.6056896	0.619365715	0.328114308	0.614967825
sgrR	0.604784749	1.002848935	0.54646435	0.773791993
ubiC	0.601877265	0.898328737	0.502859911	0.743875155
grxA	0.601643754	1.288877828	0.640645427	0.832453872
fliA	0.600418706	0.861990014	0.486084775	0.732560474
ygcR	0.599938351	1.317193518	0.64877321	0.835484773
narW	0.598548475	1.23882255	0.628982724	0.826011002
yqfB	0.597309196	1.101942108	0.587783139	0.798549881
tolQ	0.594424501	0.814595828	0.465563129	0.717094577
yjjJ	0.59437187	0.701283351	0.396689293	0.66524762
livJ	0.593396246	1.059747985	0.575519746	0.791105179
rlmC	0.591654739	0.60063636	0.324601235	0.612062049
dgoK	0.591595887	1.135508323	0.602369194	0.805646453
sohB	0.591371586	0.772022651	0.443674579	0.702273064
ycjR	0.590519399	1.243484463	0.634864771	0.829512368
ytfF	0.589865376	1.203656053	0.624090448	0.821715712
rlmD	0.588173205	0.713446063	0.40970573	0.677660966
ydhQ	0.585204453	0.628860394	0.352071237	0.63210664
cheR	0.585051782	1.25495379	0.641076727	0.832453872
norW	0.58447363	1.286201898	0.649527811	0.835568019
yibQ	0.581756988	0.709698524	0.412373405	0.679025907
yphB	0.580814186	1.157091267	0.615695261	0.815584287
fhuD	0.579797438	0.633238852	0.359873463	0.637642927
ymgD	0.578785034	1.067441756	0.587669047	0.798549881
ygfS	0.578071663	0.854408767	0.498675607	0.741338802
rdgC	0.577908489	0.764411525	0.449638704	0.706193044

phoQ	0.577895409	0.648935645	0.373182401	0.649863168
frlB	0.577581245	1.17328123	0.622522404	0.820450529
tar	0.576577729	1.357746006	0.671085991	0.84716196
yceG	0.575018634	0.549361561	0.295236379	0.579606915
frlC	0.574007111	1.126066826	0.610230012	0.811904322
mgtA	0.572370793	0.70289664	0.415471432	0.682629629
yjdC	0.571645377	0.793849164	0.47146762	0.72289265
cusB	0.571318188	1.352532315	0.672728471	0.847495718
narX	0.569099269	0.772236166	0.461152893	0.713839114
yjjA	0.568850204	0.764445147	0.456794954	0.711701285
dmlA	0.568478836	0.633682104	0.369663352	0.648130467
proX	0.567215533	0.73766812	0.441934556	0.701488916
zur	0.565113539	0.894189839	0.52739743	0.7567387
nlpE	0.564137433	0.668487656	0.398724702	0.666569049
macA	0.563201259	0.593497712	0.342644667	0.625712378
sppA	0.562417721	0.612400039	0.358418442	0.636177042
lpxH	0.561502516	0.583763404	0.336116622	0.620499447
alaA	0.561157054	0.720022804	0.435767613	0.696462904
pqiB	0.561001331	0.742332983	0.449812688	0.706193044
glnD	0.560131227	0.866500261	0.518001318	0.751602485
yifL	0.56012087	1.372618542	0.683224198	0.855137666
wcaG	0.558625911	1.36682548	0.682757505	0.854817457
rfbA	0.557698962	0.795858248	0.483458031	0.73104769
yqhA	0.557500801	0.892283504	0.53210081	0.761969863
yeaD	0.556648491	0.624200166	0.372511494	0.648973612
fdnH	0.556501614	0.776182936	0.473391229	0.724186852
gntT	0.555250827	0.77198538	0.471986624	0.723099271
glcG	0.554847323	0.568917729	0.32942728	0.615436956
avtA	0.554332784	0.674549468	0.411200834	0.678686879
tauB	0.554196582	1.339760017	0.679127798	0.851588027
glpR	0.553708638	0.700147869	0.429034283	0.690456087
yfiB	0.553121535	0.936623022	0.554822827	0.780158646
tesB	0.552309997	0.573421183	0.335455291	0.620125741
yphC	0.551060442	1.223061187	0.652307884	0.835922843
ptrA	0.550969098	0.674138458	0.41376062	0.680480955
sufA	0.549327437	0.851633749	0.518909176	0.751849157
waaA	0.547684588	0.623080485	0.379404024	0.653955745
cbpA	0.54746286	0.645983429	0.396723469	0.66524762
emrB	0.547142535	1.034539097	0.596891716	0.803067964
yhhQ	0.545929041	0.773020409	0.480046052	0.728606025
yaiC	0.545113722	1.183171996	0.644997855	0.834339918
bioH	0.544838226	0.722561486	0.450826896	0.707022142
fliM	0.544590936	0.652006485	0.403574788	0.671079651
abrB	0.54400175	0.916200179	0.552673572	0.778235034
aroA	0.542241575	0.67972772	0.425025157	0.688394721
phnO	0.54178887	1.13110726	0.631945866	0.828292448
torA	0.541219418	1.263068702	0.668290315	0.844992225
kdpA	0.540379087	1.331803857	0.684926481	0.855485032
livM	0.539838542	1.353616149	0.690031637	0.858884942

modA	0.539731858	0.553418792	0.329427142	0.615436956
ygeR	0.538740611	0.974300748	0.580296937	0.794025658
ivy	0.535682439	0.934342865	0.566424432	0.786799618
tktB	0.535487252	0.990310999	0.588696214	0.798987094
pgpC	0.533369043	0.645801504	0.408859433	0.677089926
bcr	0.53156581	0.762010041	0.485437557	0.732515133
ppx	0.531445745	0.702753546	0.449509235	0.706193044
agaR	0.531359104	0.652673561	0.415572256	0.682629629
yjjV	0.530440261	0.815533729	0.515420318	0.750813499
tap	0.529876933	1.337559368	0.691992836	0.859949516
yfeH	0.528786627	0.578984615	0.361084802	0.638672682
fhlA	0.528695934	0.605407634	0.382505479	0.655677468
entB	0.525756485	0.913888683	0.56509114	0.786433053
ybjP	0.522913346	0.917238847	0.568613218	0.787912959
mdtA	0.522849946	1.02445685	0.609793704	0.811856708
mdlA	0.521990202	0.678804972	0.441902774	0.701488916
mobA	0.5206466	0.599982636	0.385520559	0.657847271
phnP	0.520584803	1.141157694	0.648253308	0.835484773
tyrB	0.519999136	0.579898801	0.369875448	0.648130467
smtA	0.51627095	0.794211155	0.515664814	0.750820131
araD	0.515746959	1.170488774	0.659484289	0.839155464
cobT	0.51528098	0.609509283	0.397885867	0.666541035
aceA	0.513769191	1.222799847	0.674370003	0.848009215
ydhB	0.513391596	1.014622166	0.612861645	0.813666378
ytfL	0.513252524	0.624965403	0.411504167	0.678686879
edd	0.510238808	0.70993031	0.472315459	0.723099271
fadB	0.509725593	1.166751627	0.662201397	0.841524323
yjbH	0.508455738	1.302643827	0.696295509	0.861655065
cynX	0.505821006	1.4927188	0.73471605	0.883485372
menD	0.505668372	0.694092926	0.466288459	0.717666444
cdsA	0.504537261	0.70811654	0.476151517	0.72622959
proY	0.503912497	0.590428427	0.393399142	0.663687108
mdtD	0.503678163	1.133580221	0.656807545	0.838288833
waaC	0.503652671	0.670406409	0.452493368	0.708213692
flk	0.502643796	0.716550799	0.483005477	0.730927125
dinJ	0.502181994	1.370263398	0.714002652	0.872382988
mocA	0.501714674	0.975739473	0.607119736	0.809969492
fdnG	0.500557164	0.857030414	0.55917996	0.783322752
fkpB	0.499765523	0.829771424	0.546979117	0.774025731
yihI	0.498697568	0.631038096	0.429363541	0.690456087
ptrB	0.498455159	0.740760792	0.501013299	0.743473243
dcp	0.496750744	0.751243085	0.508459465	0.74684004
tdcD	0.495193993	2.125553373	0.815783304	0.92234894
fpr	0.494814331	0.575209568	0.389660542	0.66012697
araA	0.494705432	1.056936781	0.639744658	0.832453872
mrdB	0.494572305	0.667269589	0.458579373	0.7125645
hcaT	0.494499645	1.079482744	0.646888186	0.835020719
ldcA	0.492277433	0.654029705	0.451640068	0.707749839
yiaS	0.492106185	1.218468639	0.686306353	0.856351479

nudF	0.491256486	0.686949597	0.474530319	0.725120962
torI	0.491200443	1.432183906	0.731618755	0.881723797
poxB	0.491198896	1.104286488	0.656456267	0.838288833
yncD	0.488632509	0.609721332	0.422898375	0.687146297
bcsZ	0.488117959	1.281968957	0.703383971	0.86593338
ccmA	0.487837634	0.637056762	0.443814403	0.702273064
fliG	0.486242214	0.969462533	0.615978089	0.815584287
pth	0.484081304	0.86266891	0.574699516	0.790577482
ygiS	0.481862941	0.829726322	0.561409462	0.783626776
ybaE	0.481249602	0.787998217	0.541382164	0.769634188
hcaB	0.481107223	1.18107312	0.683752755	0.855271111
sucC	0.480599441	1.222649726	0.694260222	0.861638946
ybhK	0.480235403	0.669525308	0.473202921	0.724184463
fsr	0.48012291	1.117355266	0.66741691	0.844741852
hycI	0.480101159	0.556669156	0.388438112	0.659622318
asnB	0.478758831	0.665123684	0.47164555	0.72289265
yefM	0.477435262	1.221594416	0.695923191	0.861638946
yggN	0.477194474	0.810609774	0.556072056	0.781121315
hyuA	0.475024394	0.73828555	0.519954504	0.752551074
yhdP	0.474414258	0.998826638	0.634807236	0.829512368
yjcD	0.471348392	1.04243544	0.651153181	0.835568019
btuR	0.471205001	0.83621039	0.573094097	0.789593837
pabC	0.471203969	0.561874796	0.401678061	0.668469015
fruA	0.470211584	0.672594483	0.484488813	0.732265615
aas	0.469654923	0.809988788	0.562029994	0.784056984
smf	0.469341937	0.578080789	0.416850857	0.683875756
prkB	0.467360784	0.595945243	0.432902561	0.694678843
mnmC	0.466556536	0.856134473	0.585783042	0.797370552
yccS	0.466131541	0.685026797	0.496214898	0.739780307
sroH	0.465031836	1.644557263	0.777352976	0.908982566
yeiH	0.464727104	0.564235799	0.410144211	0.677833115
yniC	0.464527222	0.784807502	0.553917842	0.779445655
fumA	0.464278422	0.900279657	0.606060693	0.809545322
tatD	0.463885154	0.690648419	0.501796265	0.743795999
hdfR	0.462443878	0.790521769	0.558557377	0.782984649
fhuA	0.462249617	1.277510126	0.717474318	0.874516201
ypfH	0.462151759	0.707794194	0.513790754	0.750179195
hycF	0.462067957	1.1925421	0.698412219	0.862957372
talA	0.460073295	0.648436289	0.478006839	0.726905286
rsmI	0.45916316	0.687748351	0.504368198	0.744342437
ytfB	0.458998638	0.65092195	0.480715914	0.728900347
glyV	0.458679495	3.019645822	0.879267037	0.954875052
xanQ	0.456395712	0.707447753	0.518842996	0.751849157
yhhL	0.456192632	1.17371911	0.697518244	0.862641538
mug	0.455375704	0.890031505	0.608902997	0.810996435
rnhB	0.454905601	0.95504592	0.633847815	0.828909368
ydgJ	0.454865209	0.658445379	0.489680601	0.735246355
folB	0.452621756	0.843066471	0.591353597	0.79991618
yaeQ	0.45198368	0.684800536	0.509239467	0.74684004

aer	0.451321879	1.077420725	0.675295771	0.848357316
fliF	0.451158415	0.984463738	0.646752478	0.835020719
glsB	0.450036032	0.675584532	0.505319584	0.744932943
csrD	0.449538939	0.748346306	0.548033309	0.774660751
betB	0.449442416	1.41703225	0.751113382	0.894257701
yebT	0.449167815	0.691807351	0.516165689	0.750820131
yhaJ	0.448943333	0.601010901	0.455075422	0.709840616
menF	0.44836361	0.676205958	0.50729356	0.746201681
yedJ	0.447933299	0.789427455	0.570431986	0.78845253
arsB	0.447904516	1.186419892	0.705782646	0.867306107
potG	0.447684978	1.206671213	0.710631385	0.870258245
cyoB	0.44647209	0.997111174	0.654322367	0.837582216
cysZ	0.445568051	0.827357159	0.59020169	0.799883279
acrD	0.440009553	0.9905029	0.656877436	0.838288833
yfcO	0.439791434	0.690263618	0.524036607	0.755404001
ydcF	0.439121314	0.784533579	0.575668544	0.791105179
eptC	0.43863664	0.622135848	0.48077936	0.728900347
djlA	0.437974159	0.583839772	0.453157402	0.708484422
pgsA	0.437417944	0.66777765	0.512445016	0.749712052
truC	0.435724159	0.914337781	0.63368533	0.828909368
tyrU	0.434827806	3.149188172	0.890180228	0.958903945
gcd	0.432500858	1.075290556	0.687523841	0.857079229
ydcL	0.429838787	0.876029027	0.623661786	0.82141809
gluQ	0.429438235	0.630016913	0.495473134	0.739780307
helD	0.428263984	0.672680292	0.524350838	0.755408066
dnaC	0.427746282	0.747008439	0.566907174	0.786799618
adhP	0.427331589	0.556589236	0.442624832	0.701760293
rlmM	0.427181076	0.65926738	0.517008679	0.750963463
viaA	0.427144648	0.7001637	0.541819431	0.769634188
yeeA	0.426588055	0.621947137	0.492781733	0.73853499
hcaD	0.424003002	1.103199536	0.70072694	0.86432447
metB	0.423954999	1.010487889	0.674810747	0.848273726
melR	0.42353222	0.59672623	0.477853175	0.726905286
yfbS	0.422950667	0.626043849	0.499299264	0.741721955
ybhR	0.42199118	0.636114895	0.507081896	0.746201681
yaiI	0.420507354	0.602909264	0.48551262	0.732515133
yojI	0.416863911	0.901271386	0.643702203	0.833195635
yebZ	0.416597815	0.70610451	0.555194301	0.780158646
yjdF	0.416482201	0.89321268	0.641019105	0.832453872
ybjT	0.415741351	0.842559936	0.621711303	0.820166358
ycjT	0.414710714	1.344570876	0.757752487	0.896603997
leuA	0.413505214	1.088974877	0.704153495	0.866446724
dauA	0.412549821	0.640554584	0.519542363	0.752223121
hyfF	0.412300488	1.244946445	0.740509057	0.887549959
puuA	0.411261191	1.13541097	0.717192486	0.874516201
yfhG	0.40994433	0.557753668	0.462344534	0.714853067
serA	0.409867517	0.59901714	0.493827813	0.739555949
marC	0.409253402	0.722020065	0.570838374	0.78874532
ycjO	0.408947419	1.324945779	0.757586114	0.896603997

ybjJ	0.408770305	1.086738017	0.706809626	0.867961823
dsbB	0.407992012	0.956363974	0.669665258	0.845739239
ypjD	0.407980452	0.552853382	0.460542306	0.713699736
wzxE	0.407763039	0.807000827	0.613360743	0.813666378
kefB	0.40741393	1.128848342	0.718165884	0.874577718
artP	0.404831604	0.605710033	0.503904616	0.744200114
yfcD	0.402769177	0.637889021	0.527772817	0.7567387
glpT	0.402723582	0.76563185	0.598887092	0.803067964
nrfB	0.401984205	0.571016349	0.481445102	0.729636802
mltB	0.401853822	0.593879419	0.49862237	0.741338802
waaG	0.399071239	0.601595386	0.507103292	0.746201681
yqeF	0.3983846	0.568172591	0.483198001	0.730927125
ddpF	0.398299776	1.16146665	0.731652406	0.881723797
hokA	0.397717422	1.665929977	0.811310317	0.92122904
iclR	0.396645142	0.750724844	0.597256738	0.803067964
araF	0.396426963	0.740089762	0.592202444	0.800235515
cycA	0.395988821	0.70014123	0.571676467	0.789365258
dbpA	0.395946531	0.680472984	0.56065453	0.783322752
bcsA	0.394734345	1.250593244	0.752277537	0.894612243
yceK	0.394241637	1.348043831	0.769939057	0.90629502
nirC	0.392651247	1.187903352	0.74099091	0.887549959
flgK	0.392477342	0.592343639	0.507597102	0.746201681
gntU	0.389789416	1.131384911	0.730452143	0.881723797
aslA	0.389360511	1.078780694	0.718154413	0.874577718
ibpB	0.388616101	1.043448026	0.709569331	0.869850035
modB	0.388177846	1.006437915	0.699722733	0.864049942
thiC	0.387540996	1.101234027	0.724901857	0.878813436
fepA	0.387127115	0.861241924	0.653071954	0.836509858
solA	0.386500835	0.635437259	0.543025724	0.770268119
coaA	0.386299484	0.72852115	0.595937519	0.802403176
yjhH	0.385886502	0.971635905	0.691255863	0.85988072
yheV	0.385462823	1.556482797	0.804405292	0.919399235
baeS	0.384921038	1.087636077	0.723409258	0.877266268
yeaL	0.383865493	0.572650833	0.502646935	0.743875155
rcsC	0.383059875	0.618056193	0.535401564	0.765344832
ytfE	0.382445704	0.939668627	0.684007541	0.855325901
yjgA	0.381886515	0.916475265	0.676904821	0.849852011
azoR	0.381419121	0.619945157	0.538391909	0.767523344
yijO	0.381179652	0.937657642	0.684358162	0.855485032
sapD	0.380760206	0.566423081	0.501444456	0.743795999
waaL	0.380570707	0.654516093	0.560934815	0.783337836
ybdL	0.378993671	1.050126219	0.718171984	0.874577718
galR	0.378654763	0.628739074	0.547011278	0.774025731
ybaO	0.378354335	0.803178505	0.637589826	0.831389242
sufS	0.377702319	0.973188705	0.697936114	0.862887701
yggU	0.377640685	1.141603534	0.740796728	0.887549959
rutA	0.377525709	1.299906471	0.771490901	0.90629502
astC	0.376826099	1.358517662	0.781488072	0.910531625
ydjA	0.375684386	0.568935988	0.50904334	0.74684004

yoaF	0.374305222	1.288603726	0.771454029	0.90629502
yiaN	0.374220198	1.364919039	0.783953787	0.910993273
csdA	0.373333236	0.815171609	0.646965979	0.835020719
yecJ	0.37219419	1.174475686	0.751317644	0.894257701
clsA	0.370759584	0.697937096	0.595264905	0.802143769
ydcP	0.369737224	0.705666417	0.60031006	0.804248837
mtlR	0.369103316	0.81650097	0.651229388	0.835568019
yhgE	0.36903524	0.853982299	0.665643639	0.844078609
norV	0.368761431	1.214310483	0.761371933	0.899359504
queG	0.367999851	0.577759066	0.524161681	0.755404001
sodA	0.367784088	0.858750081	0.668448462	0.844992225
ydgI	0.36744226	0.659406183	0.577368893	0.791871212
yahF	0.367331106	1.594037622	0.817749357	0.923184599
uhpC	0.366465336	1.185634641	0.757254654	0.896603997
ggt	0.366338427	1.29804133	0.777771751	0.908982566
zupT	0.36620386	0.633542735	0.563246899	0.784867886
pyrC	0.365892978	0.603610568	0.544399276	0.771676456
zntR	0.365401338	0.831789241	0.660447375	0.840117245
torT	0.361350241	0.582502606	0.535033316	0.765297267
ascF	0.360389068	1.066168655	0.73534618	0.883810677
mdtG	0.359939382	0.682609846	0.597986077	0.803067964
yphE	0.359866076	1.319871165	0.785120408	0.911216185
cutC	0.358829329	0.95785216	0.707944065	0.868645654
pstA	0.358800332	0.974416043	0.712708543	0.8719979
adiC	0.358231983	1.272206206	0.778263071	0.908982566
iaaA	0.357626917	0.784532992	0.64850009	0.835484773
fliC	0.355747277	0.854897439	0.6773159	0.850104848
cyoD	0.355519837	1.39463062	0.798784557	0.917982978
afuC	0.355283443	1.21943865	0.770783656	0.90629502
prmA	0.355238108	0.572854837	0.535179514	0.765297267
yjcS	0.355232062	1.39796255	0.7994129	0.917982978
fumC	0.355186723	0.604920058	0.557094187	0.781744491
btuF	0.354764303	0.748771481	0.635645987	0.829922328
nudK	0.351180831	0.722161841	0.626760833	0.82389378
rsmD	0.350880564	0.61559402	0.568686542	0.787912959
alkA	0.350606023	0.810831193	0.665448374	0.844078609
yidE	0.349864207	0.650241001	0.590540368	0.799883279
mqsR	0.349822887	1.064728032	0.742491236	0.88818515
rssB	0.347040677	0.836191337	0.67812328	0.850854775
glrK	0.344784947	0.743640204	0.64290222	0.833030199
yjcO	0.344128088	0.784314057	0.660833181	0.840344327
yfcE	0.344011755	0.894381793	0.700506854	0.86432447
galS	0.343704675	0.572796785	0.548475283	0.774745225
tabA	0.342554193	0.937532569	0.714828929	0.873129399
arfB	0.34218892	0.706637476	0.628208735	0.825261896
frsA	0.340077893	0.66905084	0.61124365	0.8127195
waaY	0.337717469	0.96432746	0.72618083	0.879528407
tusD	0.337700393	0.785125963	0.667106487	0.844612647
ldhA	0.336377558	0.711433065	0.636343834	0.83029865

ybjS	0.336320433	0.598158241	0.57393891	0.790335714
proA	0.336038524	0.740799626	0.650105273	0.835568019
yfjD	0.335627671	0.594154342	0.572153366	0.789485959
yoeB	0.335242374	1.225476862	0.784422198	0.910993273
ygbI	0.334630826	0.587426056	0.568910982	0.787912959
cbeA	0.332555658	1.169765165	0.776186105	0.908913481
rffG	0.331989738	0.671423719	0.620983908	0.820166358
mngA	0.33120749	1.328565457	0.803130989	0.918717545
narG	0.328905429	1.048177574	0.753682143	0.895232173
purN	0.328675752	0.631512502	0.602744374	0.805646453
bcsG	0.328217264	1.092570407	0.763865702	0.901026473
fau	0.328014471	0.760106112	0.666077419	0.844364559
prpE	0.326762435	1.691177716	0.846789882	0.941290425
ybjQ	0.325765867	0.98592192	0.741084512	0.887549959
wbbJ	0.324836247	0.822151094	0.692765185	0.860438131
cyaY	0.323549394	1.137159543	0.776008701	0.908913481
ybcF	0.321924955	1.265821985	0.799247674	0.917982978
opgB	0.321742556	0.794299754	0.685430171	0.855521525
rhaD	0.32121453	1.139583696	0.778043144	0.908982566
yfaL	0.320793703	1.036420412	0.756925372	0.896603997
treF	0.320248242	1.005547825	0.750119829	0.893619097
ybfF	0.3187412	0.731342527	0.662959891	0.841728594
yeeD	0.317163329	1.135658685	0.78003227	0.909824916
yihS	0.315338166	1.079432384	0.770184691	0.90629502
appA	0.315214259	1.040928416	0.762026809	0.899608819
glcA	0.315161255	1.331620936	0.812909007	0.92122904
mhpB	0.31411679	1.461793226	0.829857325	0.932181102
rtn	0.314024623	0.578607823	0.587319525	0.798455182
nikA	0.313430128	0.79163827	0.692159663	0.859949516
nadD	0.313328249	0.801526015	0.695860336	0.861638946
yjeH	0.312890361	0.879695898	0.722080119	0.87617863
mdtF	0.31286736	1.204132381	0.794996419	0.916642628
yhcH	0.312363722	0.859693708	0.716348649	0.874458724
kdpC	0.312035599	1.133722069	0.783138677	0.910993273
argG	0.311565503	0.658789689	0.636258686	0.83029865
plsY	0.311528404	0.662356533	0.638116834	0.831525666
frwC	0.311437542	1.237167953	0.801246485	0.918382466
yiiM	0.30975817	0.54865922	0.572364248	0.789508221
wcaJ	0.309727521	1.248544414	0.80407956	0.919399235
yobA	0.309704618	1.155636257	0.788703248	0.913188139
gnsB	0.304918072	1.727040427	0.859857739	0.944934474
cheB	0.303393766	1.330883483	0.819674085	0.92458507
ytfJ	0.302841069	0.789882372	0.701423046	0.864569483
yjbD	0.302677642	1.26456302	0.810831449	0.92122904
hofB	0.302640381	0.854791793	0.723300232	0.877266268
yahJ	0.302189	1.264449734	0.811114466	0.92122904
narQ	0.302041638	0.587507741	0.607177426	0.809969492
zapC	0.301794135	0.808118003	0.708811281	0.869238567
hyfG	0.30052602	1.140362917	0.792137927	0.913866578

dcuR	0.30012036	0.724704703	0.678780518	0.85141591
bisC	0.299841499	0.90986679	0.741744245	0.888070136
nudE	0.298657713	0.947753191	0.75266916	0.894815476
hsrA	0.298213864	0.619543626	0.630271736	0.826632681
yraN	0.298000578	0.833595348	0.720726288	0.875654502
aaeR	0.29745947	0.751055912	0.692064434	0.859949516
srlE	0.297050136	0.968758698	0.759125286	0.897490204
ygcQ	0.296019551	1.221588096	0.808529476	0.920008611
cusR	0.295620944	0.860797989	0.731277536	0.881723797
clcA	0.293558672	0.858411385	0.732366692	0.882322308
yhhW	0.291786574	0.597050351	0.625044536	0.822704724
pflD	0.291427842	1.101355402	0.791311182	0.913602398
gtrS	0.290923499	0.70450579	0.679645501	0.851973673
ybhB	0.290654532	0.905971156	0.748346078	0.892380382
atoS	0.290038173	0.62180136	0.640894663	0.832453872
argC	0.287662475	0.847849512	0.734395172	0.883485372
yrbG	0.287216966	0.572421075	0.615837622	0.815584287
adiA	0.286797711	1.039217685	0.782567556	0.910861003
trpA	0.286637922	0.575859159	0.618655334	0.818280171
ycbX	0.286229323	0.76146407	0.706996143	0.867961823
yebO	0.285408555	1.168963554	0.807110606	0.91948485
ycaQ	0.284652775	0.74554856	0.702607396	0.865503003
cyoC	0.284491803	0.914591749	0.755755929	0.896382252
eutQ	0.282450992	1.222842737	0.817331117	0.923184599
aes	0.281915538	0.559034981	0.614057871	0.8143247
dacC	0.281849981	0.695254158	0.685189911	0.855485032
ynfE	0.280786044	0.717651037	0.695607226	0.861638946
yjcB	0.27970261	1.181851665	0.812916896	0.92122904
ychN	0.275126613	1.007071919	0.784703629	0.910993273
malY	0.273781738	0.587629964	0.641281066	0.832453872
yehW	0.272529166	1.233227107	0.825101288	0.928639817
php	0.272330959	1.088214388	0.802390249	0.918717545
uraA	0.268859205	0.800748376	0.737051921	0.885202119
yaiW	0.267879112	0.78029104	0.73136777	0.881723797
sgcX	0.267853882	0.650275977	0.680406851	0.852664413
ybdG	0.266677513	0.681192563	0.69543786	0.861638946
dpiA	0.266623298	0.600866685	0.657236933	0.838288833
elaB	0.26586117	1.044266324	0.799038743	0.917982978
metN	0.265731128	0.678203729	0.695194111	0.861638946
cvrA	0.264805624	0.794753781	0.738989335	0.88634993
hicA	0.263632907	1.581627975	0.867618283	0.948065908
yddE	0.261234644	0.780388277	0.737814001	0.885202119
ttcA	0.259292155	0.744550064	0.727649823	0.880260709
yicI	0.258937836	1.077903689	0.810156969	0.921025337
gsiC	0.258296191	1.043056424	0.804417634	0.919399235
murQ	0.257971553	0.882616547	0.770072213	0.90629502
ykfA	0.256686812	1.029697527	0.803141337	0.918717545
cobB	0.255350346	0.734354512	0.728049866	0.880260709
yoaH	0.255206337	0.875604244	0.770697153	0.90629502

gadA	0.253742488	1.28209608	0.843113739	0.939264385
ygfF	0.253566862	0.705890017	0.719433641	0.874823363
glpQ	0.25326444	0.723318158	0.726231744	0.879528407
ymgG	0.253220569	0.624335685	0.685048282	0.855485032
csiD	0.252723218	1.198000348	0.832923002	0.932648078
fixA	0.250981524	1.170372726	0.830199424	0.932307054
yjcZ	0.249444799	1.264153227	0.843575839	0.939521003
yibF	0.248667643	0.724951351	0.731588778	0.881723797
ycjS	0.248104899	1.149267806	0.829080633	0.931770091
tusB	0.247619727	1.048122842	0.81323816	0.92122904
caiA	0.247454982	0.667292682	0.710760989	0.870258245
argD	0.246098296	0.9500381	0.795604032	0.916821701
agaS	0.245366991	1.16075192	0.832585888	0.932648078
yobD	0.242682944	0.987373915	0.805847692	0.91948485
tyrV	0.240286994	1.848473668	0.896572668	0.961537769
folM	0.240173524	0.670168723	0.72006068	0.875300149
ebgR	0.239228418	0.574006348	0.676846912	0.849852011
ybjD	0.238927195	0.665182586	0.719452662	0.874823363
narI	0.238472627	1.004270822	0.812301123	0.92122904
yegH	0.238037465	0.62886943	0.705046927	0.866664719
alsK	0.237631602	1.005629888	0.813198915	0.92122904
leuQ	0.237007271	2.86353061	0.934036411	0.974124305
ttdA	0.23668165	1.24029725	0.848661484	0.94173934
cysN	0.236427493	1.150712697	0.837211347	0.93577469
yjdP	0.236270843	0.78309232	0.762869192	0.900341108
ubiX	0.236121627	0.719687196	0.742844188	0.888345232
uspB	0.234426133	1.223022032	0.847994622	0.94173934
hofQ	0.234177921	1.274596862	0.854227588	0.942579924
thiH	0.232475403	1.078028301	0.829261591	0.931770091
yohK	0.232141074	0.821265237	0.777435389	0.908982566
prpD	0.22568411	1.585801809	0.886830837	0.956971295
yejH	0.224682539	0.748743913	0.764116678	0.901026473
intA	0.223530156	0.697586867	0.748639783	0.892380382
uspD	0.222543787	0.868553583	0.79777831	0.917982978
folA	0.222271976	1.014137652	0.826514992	0.929460312
yjjU	0.221398954	0.609317412	0.716339191	0.874458724
nemA	0.220312441	0.61886186	0.721843446	0.876153692
xylH	0.21881415	1.154103195	0.849625178	0.941859577
yjjP	0.21493192	0.83616279	0.797143614	0.917918719
yhfW	0.214173925	1.162080998	0.853776495	0.942579924
yhhY	0.213212128	0.846178868	0.801063852	0.918382466
mdtO	0.212865278	1.271158671	0.867009946	0.947656598
astB	0.212722273	1.260357239	0.865970203	0.947534878
pdxK	0.212338585	0.586711403	0.717416916	0.874516201
yfdY	0.210711342	1.236481359	0.864686085	0.947159521
ydeN	0.20864471	0.732317786	0.775713042	0.908884588
yebS	0.208081739	0.668591823	0.755630433	0.896382252
yihY	0.206440686	0.713526935	0.772333213	0.906607941
smrB	0.205912003	0.677674871	0.761241617	0.899359504

cheZ	0.205143555	0.872859893	0.814189633	0.921732693
yfjK	0.204829401	0.661282369	0.756754311	0.896603997
bacA	0.203653932	0.625148951	0.744599397	0.889394801
wbbH	0.203383343	0.731107887	0.780870249	0.910453836
pyrB	0.202931927	1.035486002	0.8446278	0.939917953
qseC	0.202274715	0.894674728	0.821133417	0.925839061
eutM	0.199677618	1.086316903	0.854161277	0.942579924
ccmB	0.198529149	0.934935249	0.831837621	0.932596713
tyrA	0.197177328	0.54757504	0.718778421	0.874823363
ygjJ	0.196353055	1.215275993	0.871643892	0.950159811
yjdL	0.195989688	1.090710924	0.857396058	0.944453268
tsaE	0.195442822	0.73319744	0.789806159	0.913255609
narZ	0.193907289	1.329144097	0.884009142	0.956468768
rnlB	0.193316453	1.188476042	0.870786845	0.949991062
csdE	0.192267569	0.646847628	0.766284961	0.902795476
aldA	0.190391802	0.7898928	0.809527999	0.920826742
pabB	0.189934508	0.656757865	0.772428177	0.906607941
ybaY	0.18898718	0.754065115	0.802104451	0.918717545
ydiU	0.188824405	0.818538229	0.81755961	0.923184599
appC	0.186925373	0.993560095	0.850769291	0.942354837
yjbF	0.186504147	1.244932627	0.880914019	0.955717638
ybiJ	0.185888111	0.850517258	0.826993677	0.929737206
yafE	0.184917362	0.717323609	0.796570587	0.917674669
nohD	0.184868162	1.602598266	0.908163528	0.967399578
mhpA	0.183666632	1.591191959	0.90810662	0.967399578
yfjL	0.183292162	0.645499984	0.776445799	0.90895503
ryfD	0.182710905	1.631459697	0.910829521	0.968763388
hcr	0.181760033	0.979199675	0.852741976	0.942579924
rsd	0.181635059	1.05994066	0.863938014	0.947114201
cyoE	0.18145217	0.916527779	0.843062419	0.939264385
ypdA	0.181206859	0.736889358	0.805753586	0.91948485
ydiI	0.179394784	0.892591491	0.840712683	0.937878156
malX	0.179003031	0.675874324	0.79112762	0.913602398
birA	0.178540924	0.548818861	0.744940037	0.88953959
hyfH	0.175143439	1.146806495	0.878616921	0.954424704
waaR	0.173292459	0.607096175	0.775303373	0.908667209
phoB	0.1727982	0.632293218	0.784631777	0.910993273
creA	0.172686276	0.844388398	0.837954604	0.936347289
dpiB	0.172053225	0.9830617	0.861065704	0.945749218
yciA	0.171273895	0.624455866	0.783871741	0.910993273
yqaE	0.169160245	1.531552544	0.912052363	0.969003707
flhA	0.169129185	1.043589286	0.871254753	0.949991062
fixC	0.168916657	1.277817734	0.894832816	0.961407025
dinD	0.164975384	0.776035074	0.831648695	0.932596713
tag	0.163251548	0.790392388	0.836365265	0.935344768
ydgK	0.162476704	1.054518359	0.877549263	0.953776063
mdfA	0.162115954	0.909391621	0.858512052	0.944479745
alaE	0.161560212	0.896593962	0.85700083	0.944354815
rnhA	0.160152502	0.977941556	0.869916224	0.949991062

cutA	0.15748706	0.88640079	0.858981941	0.944740312
malI	0.156893853	0.845163359	0.852729192	0.942579924
pspF	0.15678518	0.742988868	0.832872012	0.932648078
ycfQ	0.154826415	0.733822582	0.832898064	0.932648078
mltC	0.154113821	0.628902591	0.806416189	0.91948485
betI	0.15231004	1.158542347	0.895405922	0.961407025
etk	0.151798063	0.714242741	0.831693518	0.932596713
stpA	0.151620591	1.070927451	0.887412738	0.957055397
ybiU	0.148744698	1.032636564	0.885466033	0.956482627
paaF	0.148087279	1.317551604	0.910509578	0.968763388
ydaN	0.147115496	0.667857223	0.825653204	0.929003077
glk	0.146822241	0.588598757	0.803017599	0.918717545
sulA	0.146239864	0.748886741	0.845176616	0.940270582
ttdT	0.145777293	1.240783776	0.906473296	0.966555166
ycfJ	0.145552846	0.620857962	0.814644777	0.921781155
ybdD	0.145295355	1.638420257	0.929336108	0.972682993
mqo	0.144632844	1.117533343	0.897024135	0.961537769
wcaL	0.144435792	1.190716436	0.903452164	0.965571317
coaE	0.143981277	0.609759467	0.813333356	0.92122904
creC	0.143480849	1.04263722	0.890545968	0.958903945
wcaH	0.143283262	1.225457278	0.906921641	0.966778946
thiK	0.143268435	0.591352499	0.808569038	0.920008611
yjbR	0.143191213	0.878077767	0.870460566	0.949991062
yjjX	0.142864801	0.611244921	0.815196469	0.922100024
endA	0.142577417	1.020393538	0.888875005	0.958377465
rpiR	0.141729269	0.728689084	0.84578521	0.940689504
degQ	0.140473635	0.584483674	0.810068242	0.921025337
frmB	0.139521245	0.567054836	0.805647073	0.91948485
ygaZ	0.138897351	0.583231441	0.811763714	0.92122904
cysU	0.138697393	1.284850043	0.914036694	0.970330302
ravA	0.138336787	0.590215312	0.814687193	0.921781155
gudP	0.138052698	0.669619206	0.836661263	0.93541775
fliK	0.137569597	0.935624385	0.883104339	0.956224624
rimI	0.1374908	0.721598265	0.848888752	0.94173934
ybiP	0.137412387	0.652900936	0.833305254	0.932694506
mutH	0.135951512	0.703517624	0.846766821	0.941290425
yjhQ	0.135828062	0.573703635	0.812845704	0.92122904
tonB	0.135306501	0.598841422	0.821242541	0.925839061
yidK	0.135124895	1.338176328	0.919568799	0.971244791
cysM	0.134673588	0.795977915	0.865645135	0.947534878
yibH	0.132794726	1.234283208	0.91432209	0.970330302
nhaR	0.131653947	0.733505396	0.857556112	0.944453268
ychQ	0.130430037	1.034219744	0.899641343	0.963578865
aldB	0.130081223	1.075363744	0.90371887	0.965571317
yhjG	0.12831149	1.329172255	0.92309577	0.971283511
ligT	0.128290123	0.805166753	0.873406044	0.951045791
yccU	0.127345671	0.700557107	0.855757173	0.943871671
yceF	0.125955105	0.719049523	0.860946931	0.945749218
glyW	0.123419775	1.934550354	0.949131346	0.978283415

ydiR	0.12316041	1.232708103	0.920415427	0.971271272
hmp	0.122183203	0.819601647	0.881493405	0.955717638
nadB	0.118693027	0.992166081	0.904776095	0.966126825
zitB	0.118382074	0.632729334	0.851584191	0.942546152
xanP	0.117563509	1.045294141	0.910451304	0.968763388
pstB	0.1152656	0.568044554	0.839200315	0.936959963
yqjI	0.114844463	0.617649782	0.852493512	0.942579924
ygjP	0.113595585	0.602242079	0.850389767	0.942191887
epmA	0.112391924	0.636184218	0.859770992	0.944934474
xylA	0.112013075	0.898275359	0.900762698	0.964015834
yfcA	0.111344127	0.628036949	0.859281285	0.944813216
rlmI	0.110307761	0.635111666	0.862115017	0.946175419
pabA	0.11008705	0.692154553	0.873629631	0.951045791
waaQ	0.110069726	0.921093052	0.904880009	0.966126825
pgpB	0.109621233	0.762712175	0.885717188	0.956499062
yifK	0.108527788	0.819454195	0.894636979	0.961407025
malS	0.108342798	0.679091792	0.873242874	0.951045791
eutH	0.107274975	1.29511176	0.933986181	0.974124305
ydfO	0.107088607	1.121322164	0.923916032	0.971283511
flhB	0.105045562	1.132531432	0.926099879	0.971637916
mliC	0.104557442	1.05972962	0.921404844	0.971271272
bioB	0.104386579	1.170003811	0.92890786	0.972682993
yhcM	0.103115825	0.577884548	0.858380011	0.944479745
wecG	0.101919498	0.766219574	0.894180691	0.961285739
modC	0.10039771	0.827805706	0.903467889	0.965571317
sbmA	0.100178549	0.574359495	0.861537086	0.946010657
mscL	0.10010486	0.822041855	0.903076527	0.965571317
yaiA	0.098934598	1.460415481	0.945989307	0.978283415
yajR	0.096952793	1.028396356	0.924890148	0.971287022
creB	0.094174238	0.65210183	0.885171619	0.956468768
phoR	0.09360526	0.915470093	0.918559591	0.970905917
fliP	0.09321859	1.041082291	0.928652704	0.972682993
csgG	0.092929065	0.631515132	0.883011609	0.956224624
yhjY	0.092181226	0.709704448	0.896656003	0.961537769
osmF	0.091758751	0.931309905	0.921514191	0.971271272
dmlR	0.091670149	0.64567204	0.887098699	0.956971295
gltL	0.089284544	0.569071534	0.87532751	0.952600863
hyfR	0.088873128	1.143759027	0.938064572	0.975356187
aslB	0.083979151	0.932982201	0.928278056	0.972682993
yeiI	0.082396424	0.577901864	0.88662302	0.956971295
nanM	0.080538233	0.61304431	0.895479262	0.961407025
ypdB	0.079929835	0.736221561	0.913545431	0.970268058
yniB	0.079876463	0.902764368	0.929495293	0.972682993
coaD	0.077430476	0.616747509	0.900090934	0.96380577
frlR	0.077254462	0.556065501	0.889505063	0.95878232
yccF	0.076342393	0.877842139	0.930698562	0.97318854
frvX	0.076220921	0.946847343	0.93583984	0.974542694
yjdA	0.073130089	1.165187339	0.949955615	0.978283415
galP	0.073025549	0.759476656	0.923399487	0.971283511

alx	0.072869594	0.71167801	0.918446136	0.970905917
purC	0.072319613	0.69956769	0.917663307	0.970573193
ybiV	0.071732661	0.685016809	0.916600655	0.970573193
napH	0.071412739	0.947455977	0.939917824	0.975938366
ascG	0.071281001	0.604072106	0.906067012	0.966555166
yhjC	0.07108635	0.980902826	0.942227617	0.976454219
purT	0.070422319	0.819424256	0.931513145	0.973301901
araG	0.070248488	0.734116412	0.923765822	0.971283511
yegE	0.069342977	0.997604878	0.944584103	0.978044951
tamA	0.069181487	0.728451791	0.924338197	0.971287022
paaE	0.068844509	1.265754555	0.956624371	0.981314575
yhjX	0.068696432	0.622419612	0.912115971	0.969003707
ybaP	0.067787495	0.695887521	0.922399557	0.971271272
yigM	0.066392935	0.841657577	0.93712525	0.974879591
rhtC	0.066043142	0.637789744	0.917526451	0.970573193
phoA	0.065769356	1.049886287	0.950049779	0.978283415
yieE	0.063582787	0.648305184	0.921872426	0.971271272
yqfA	0.062125511	0.956026756	0.948187515	0.978283415
ydiS	0.061901913	1.323735749	0.962702083	0.98316116
fucK	0.061318981	0.571708965	0.914586218	0.970356589
gmd	0.06038585	1.294361541	0.962789789	0.98316116
waaB	0.059491002	0.612393866	0.922611245	0.971271272
caiD	0.05923305	1.196964416	0.960531948	0.98281638
kefG	0.055352957	0.825711375	0.946552495	0.978283415
yiaY	0.054566374	1.16814898	0.962742903	0.98316116
znuC	0.053087148	0.711282159	0.94050445	0.975938366
yiiQ	0.051838048	0.554837054	0.925562499	0.971553258
gspE	0.050332028	1.192558683	0.966335217	0.985292498
cpsB	0.049926884	1.266383537	0.968551729	0.985569455
yigI	0.049544584	1.006881068	0.960755134	0.98281638
cof	0.049196444	0.553409078	0.929163714	0.972682993
nirD	0.048914653	0.773456697	0.949574104	0.978283415
yfbR	0.047982651	0.800331205	0.952192675	0.979743428
yeiW	0.047460678	1.160730305	0.967384678	0.985371227
yedQ	0.046815318	0.686916519	0.94566396	0.978283415
leuT	0.046068524	2.90180999	0.987333485	0.993875244
yifB	0.045712247	1.102671609	0.966932447	0.985371227
sufD	0.043991515	0.875593875	0.959929609	0.982467719
ulaA	0.0428456	1.085358577	0.968510899	0.985569455
trpC	0.042810612	0.728337608	0.953128512	0.98015677
yecH	0.042765902	1.202679784	0.971634126	0.98746672
yfiN	0.041395895	0.629222791	0.947545869	0.978283415
rtcB	0.040939685	1.063437685	0.96929103	0.986074238
yhfX	0.040718938	1.220703994	0.973389955	0.988260175
yeiQ	0.040385346	0.628864885	0.948795505	0.978283415
rstB	0.039319908	0.604499035	0.948137818	0.978283415
mdtK	0.037870584	0.660636903	0.954286821	0.98015677
mscS	0.036656525	0.653097812	0.955240508	0.980887796
cpsG	0.036283001	1.275791428	0.977311539	0.989832706

yfjQ	0.036080465	1.272535564	0.977380444	0.989832706
clsC	0.035949842	0.591185817	0.951510752	0.979411763
lysR	0.035393056	0.864094671	0.967328026	0.985371227
pstC	0.035158313	0.943541902	0.970276057	0.986333786
ytfH	0.034436676	0.794008554	0.965406065	0.984592752
blr	0.034062076	1.690788541	0.983927164	0.992495826
yaaW	0.033399941	1.224610551	0.978241251	0.989962564
barA	0.032480694	0.693598721	0.962649333	0.98316116
yfgO	0.03217224	0.581351177	0.955867231	0.980898446
pyrD	0.03195859	0.573006489	0.955522228	0.980898446
abgR	0.029127299	0.561985764	0.958664751	0.981985466
dgkA	0.028477244	0.994255902	0.977150302	0.989832706
hyfI	0.028405197	1.319497482	0.982825039	0.99195534
fliH	0.028134174	0.950684053	0.976391161	0.989832706
nadC	0.027602726	0.585621327	0.962406361	0.98316116
wecF	0.027230205	0.622825369	0.965127239	0.984556071
mpaA	0.026246474	0.664932178	0.968513752	0.985569455
yhhJ	0.023620786	1.052844257	0.982100791	0.991887545
fucA	0.022809698	0.788086409	0.976909937	0.989832706
yfiP	0.022348868	0.7143377	0.975041345	0.989441205
yidZ	0.021546763	0.848691388	0.979745308	0.99098968
ygfT	0.020529853	1.170473648	0.986005996	0.993088882
ugpB	0.020315071	1.166233901	0.986102049	0.993088882
rluE	0.020247875	0.578621387	0.972085079	0.987677421
dsdX	0.017324292	0.592413881	0.976670338	0.989832706
yghA	0.016644094	1.062455753	0.987501106	0.993875244
yfhL	0.014652204	1.326236831	0.991185187	0.996258166
ycaD	0.014610855	1.000564292	0.988349213	0.993988516
atoC	0.014408506	0.593514997	0.980632005	0.991392057
yihW	0.013280571	0.570867976	0.98143983	0.991467
purF	0.013046651	0.755925639	0.986229857	0.993088882
speC	0.012645408	0.727434417	0.986130616	0.993088882
ycjU	0.012449735	1.421665949	0.993012899	0.997194523
ycbF	0.007858977	0.867722234	0.992773643	0.997194523
ligB	0.007405892	1.083000025	0.994543859	0.997990298
gcl	0.007030973	1.248996675	0.995508495	0.998414441
trg	0.005705678	0.92390092	0.995072585	0.998273759
ydhP	0.005279999	0.662487709	0.993640962	0.997578122
ybhS	0.005092995	0.690310123	0.994113392	0.997805321
ybiR	0.004040084	0.986283761	0.996731659	0.998414441
yafS	0.002745367	0.627855557	0.996511174	0.998414441
yhdN	0.001536305	0.708192768	0.998269125	0.999008709
msyB	-0.000201634	1.002876461	0.999839581	0.999839581
yafK	-0.000955968	1.092984303	0.999302138	0.999795377
leuV	-0.001319192	2.928771224	0.999640613	0.999839581
ykgE	-0.002158978	0.995404344	0.998269433	0.999008709
flgM	-0.002271269	0.636162258	0.997151345	0.998414441
fecI	-0.003106031	0.66802701	0.996290202	0.998414441
moaB	-0.003311482	0.617700471	0.995722574	0.998414441

allC	-0.003549899	1.005484743	0.997183047	0.998414441
mdtE	-0.004439781	1.208513341	0.997068775	0.998414441
bglF	-0.010241034	1.091386468	0.992513153	0.997186697
ydgD	-0.011728983	0.668209621	0.985995571	0.993088882
hcaF	-0.012289971	1.132729887	0.991343227	0.996258166
ybiI	-0.01241639	0.649350472	0.984744383	0.992826095
yjfC	-0.012969147	1.130235848	0.990844694	0.996251089
fliL	-0.014397438	0.548691869	0.979066256	0.990550188
nlpC	-0.016994447	0.804505075	0.983146659	0.99195534
fruK	-0.017794298	0.586456091	0.975794237	0.989832706
eptA	-0.018092985	1.187457293	0.987843306	0.993972887
ygaM	-0.018850201	0.678633164	0.977840236	0.989962564
yraR	-0.0194109	0.773897245	0.979989548	0.99098968
aroH	-0.020625077	0.598816165	0.972523825	0.987875617
ykfG	-0.021180787	0.960794409	0.982411998	0.99195473
tauD	-0.024680532	1.059247417	0.981410921	0.991467
yadE	-0.025256914	0.61752208	0.967375281	0.985371227
soxS	-0.025507448	1.300842267	0.984355755	0.992681152
ecnB	-0.025755075	1.760184941	0.988325748	0.993988516
insA	-0.030193157	1.097512192	0.978052531	0.989962564
btuD	-0.030384258	0.591270363	0.959016271	0.981985466
frmR	-0.030786432	1.070390815	0.977054518	0.989832706
yjhC	-0.03143332	0.587642502	0.957341061	0.981553025
fhuF	-0.033860821	0.629497784	0.957102306	0.981553025
mdtH	-0.034862741	1.072280848	0.974063192	0.988696089
yohF	-0.035824471	1.060617417	0.97305498	0.988167557
narH	-0.038318422	0.747624036	0.959123443	0.981985466
ldcC	-0.039156256	0.823324009	0.962067912	0.98316116
dsbE	-0.039175371	0.563527091	0.944577172	0.978044951
acpH	-0.039749047	0.607302912	0.947814311	0.978283415
fadJ	-0.03988413	0.768938747	0.958633027	0.981985466
clcB	-0.0422299	0.890982675	0.962196825	0.98316116
nudC	-0.042615491	0.568528799	0.940248535	0.975938366
cbrB	-0.043303458	0.745285743	0.953666456	0.98015677
evgS	-0.043831477	0.686417793	0.949085372	0.978283415
pheP	-0.046120836	0.598906964	0.938616904	0.975680238
dkgA	-0.048300552	0.550510899	0.930085167	0.972966762
yfgH	-0.04830174	1.217118707	0.968344009	0.985569455
ugpA	-0.048651707	1.293535886	0.969997512	0.986333786
insQ	-0.049047741	0.599836924	0.934830837	0.974124305
nadR	-0.049437022	0.625006126	0.936954308	0.974879591
yceA	-0.049785284	0.54630038	0.927387942	0.972485959
csiE	-0.050067157	0.76124383	0.947560783	0.978283415
rhaR	-0.050182026	0.724140895	0.944751857	0.978044951
ytfR	-0.05028209	1.097951076	0.963472613	0.983363034
crcB	-0.050766454	0.881772697	0.954088617	0.98015677
yehX	-0.051404231	1.117887635	0.963323512	0.983363034
ypjA	-0.052221283	1.400066573	0.970246489	0.986333786
wzxB	-0.052402137	0.764174402	0.945329088	0.978283415

cho	-0.053635996	0.587346738	0.927239014	0.972485959
yiiX	-0.055571936	0.551694836	0.919765206	0.971244791
betA	-0.056875457	1.296490236	0.965008994	0.984556071
aceK	-0.05702336	1.11510522	0.959216193	0.981985466
murJ	-0.05716638	0.901642624	0.949446011	0.978283415
uspC	-0.058171379	0.958949582	0.951628745	0.979411763
ykgB	-0.058492466	0.917547831	0.94917034	0.978283415
kup	-0.058567378	0.677924781	0.931154689	0.973301901
yrdB	-0.058694977	1.01322341	0.953805216	0.98015677
ppdC	-0.059080898	0.924655575	0.949053808	0.978283415
leuP	-0.059343877	2.800770237	0.983095357	0.99195534
metI	-0.062699513	0.545717876	0.908529427	0.967475255
yfeO	-0.062839097	0.859696502	0.941730908	0.976413581
recN	-0.063844501	0.956750003	0.946796174	0.978283415
carA	-0.064081183	0.748424012	0.93176731	0.973301901
yfaE	-0.064850902	1.263218909	0.959056334	0.981985466
chiA	-0.067606711	0.851876504	0.936744611	0.974879591
yqhC	-0.068629186	0.706686574	0.922635833	0.971271272
tehB	-0.068852615	0.776309489	0.929326513	0.972682993
feaB	-0.069278238	0.85205711	0.935197779	0.974124305
fepC	-0.070000944	0.826790761	0.932527044	0.973566127
dcuS	-0.071095188	0.588744977	0.903883361	0.965571317
trpE	-0.072493796	1.220727606	0.952644882	0.979959997
pyrE	-0.072586615	0.882267031	0.934429751	0.974124305
ygaU	-0.072750753	0.887110535	0.934639821	0.974124305
nrfG	-0.072866356	0.927518504	0.937382174	0.974896699
rseC	-0.072884343	0.769982537	0.924587164	0.971287022
ybcH	-0.072918936	0.975584216	0.940418505	0.975938366
yaaU	-0.072938463	1.263582468	0.953968836	0.98015677
hypA	-0.073648325	0.971177958	0.939551153	0.975938366
dtpD	-0.07527391	0.723063798	0.917086733	0.970573193
narV	-0.076382169	1.182301636	0.948488792	0.978283415
argT	-0.07647503	0.685510292	0.911172866	0.968763388
ascB	-0.07702342	0.808310653	0.924084978	0.971283511
fes	-0.0772232	1.154618421	0.946675635	0.978283415
nanC	-0.078188886	0.632518377	0.901619957	0.964424091
puuD	-0.078244601	0.678709569	0.908219732	0.967399578
patD	-0.079214533	1.119645	0.943596975	0.977598297
eutR	-0.080584214	0.954749979	0.932735646	0.973566127
ygdQ	-0.080584409	0.557511262	0.885071621	0.956468768
waaJ	-0.081152663	0.59142801	0.890861031	0.958988481
yciE	-0.081649975	1.001205102	0.935003214	0.974124305
torR	-0.083041955	0.770978172	0.914225844	0.970330302
hicB	-0.083161316	1.048655088	0.936791756	0.974879591
ykgF	-0.084434569	0.829626064	0.918935866	0.970905917
hscC	-0.084655648	0.967109038	0.930246465	0.972966762
wcaK	-0.084763266	1.029769367	0.934397925	0.974124305
apbE	-0.085170428	0.856593921	0.920797518	0.971271272
actP	-0.085617062	1.148784797	0.940589965	0.975938366

clsB	-0.086364635	1.192224535	0.942251826	0.976454219
aaeB	-0.086372259	1.162862041	0.940791103	0.975938366
tam	-0.086933473	0.877730598	0.921103766	0.971271272
rluF	-0.087855557	0.608592283	0.885217272	0.956468768
yfeR	-0.088600633	1.032772811	0.931634085	0.973301901
allD	-0.089172852	1.17025778	0.93926052	0.975938366
yjeM	-0.089258348	0.617770704	0.885117822	0.956468768
mazG	-0.089595934	0.717289395	0.900595557	0.964015834
ycgN	-0.091596241	0.706028235	0.896776463	0.961537769
mntR	-0.092407866	0.549316865	0.866407653	0.947534878
araC	-0.093369054	0.545859701	0.864184808	0.947114201
ycgV	-0.095740513	1.027742619	0.925779536	0.971553258
yfiM	-0.0961918	1.069201837	0.928314243	0.972682993
yfcL	-0.099129808	1.030415028	0.923358738	0.971283511
xylR	-0.099333308	0.79995707	0.901178063	0.964205824
greB	-0.099864139	0.979363307	0.918781734	0.970905917
ydhK	-0.100168031	0.967496864	0.917539814	0.970573193
ompT	-0.102326085	0.775450423	0.89501833	0.961407025
insF1	-0.103417084	4.420505629	0.98133531	0.991467
rutE	-0.104237162	1.235079839	0.93274071	0.973566127
aaeA	-0.104265352	0.979091229	0.915192027	0.970491363
ybgS	-0.105484922	0.829769123	0.898840967	0.962976025
cusS	-0.106512344	1.006657019	0.915734707	0.970573193
znuB	-0.106854536	0.736348981	0.884620876	0.956468768
uidB	-0.108143134	0.779949155	0.889723504	0.95878232
mngB	-0.108828451	0.732571301	0.881903361	0.955717638
dsrB	-0.109704629	1.494871542	0.941497901	0.976413581
yeaH	-0.109870242	1.04010775	0.915873129	0.970573193
mdaB	-0.111814678	0.824489542	0.89212418	0.959715652
deoR	-0.112470307	0.830273054	0.892246742	0.959715652
dsbG	-0.113306067	0.730409062	0.876721305	0.953339127
dgoD	-0.113328576	1.07897385	0.916349097	0.970573193
ybaN	-0.114241398	0.604510153	0.850107106	0.942136196
ygbN	-0.116514099	1.115547912	0.916815717	0.970573193
ydcI	-0.117362558	0.580872259	0.839881245	0.937208524
proV	-0.117414685	0.556963083	0.833033717	0.932648078
caiE	-0.118472575	1.007797078	0.906419484	0.966555166
wza	-0.118531858	1.25992284	0.925046668	0.971287022
ulaR	-0.119482991	0.545162804	0.826518111	0.929460312
yiaK	-0.120827837	1.202688363	0.919975332	0.971244791
mppA	-0.121688969	0.756377947	0.872184911	0.950363936
ydbH	-0.122761033	0.8914571	0.890470967	0.958903945
waaU	-0.123217337	0.679463377	0.856096728	0.943871671
lpxT	-0.125059951	0.798803961	0.875592415	0.952600863
yghJ	-0.125611169	0.970164881	0.896982563	0.961537769
intD	-0.125932966	0.618082641	0.838550602	0.936755067
narY	-0.12625322	1.216535247	0.917343161	0.970573193
mreD	-0.12706352	0.780677114	0.870706886	0.949991062
nhoA	-0.127563698	0.867230967	0.88305853	0.956224624

pqiA	-0.127719301	0.689757055	0.853099125	0.942579924
ygcS	-0.128836541	1.303446568	0.921262944	0.971271272
mnaT	-0.129179969	0.715471408	0.856719042	0.944300978
nrdE	-0.129191245	1.096104806	0.906175455	0.966555166
yfiC	-0.129224628	0.768463389	0.866457588	0.947534878
yijE	-0.130208312	0.867965321	0.880752331	0.955717638
ugpE	-0.130856428	1.22579064	0.914985194	0.970491363
dhaL	-0.133446804	0.938643846	0.886945889	0.956971295
caiB	-0.133913042	0.772563285	0.862387315	0.946175419
aroD	-0.136714525	0.732301989	0.851902555	0.942579924
ccmE	-0.137454935	0.647039344	0.831766318	0.932596713
hyfA	-0.138265521	1.170916826	0.906001792	0.966555166
potI	-0.139411335	1.218497035	0.908910777	0.967627177
queD	-0.140895302	1.104057047	0.898452898	0.962814711
sxy	-0.142349028	0.70947784	0.840980746	0.937919104
tehA	-0.142373998	0.878092275	0.871195503	0.949991062
sapB	-0.14253609	0.641465498	0.824155213	0.928090343
paaX	-0.144561938	0.633773012	0.819570569	0.92458507
yceJ	-0.148570493	1.092096242	0.891788414	0.959715652
allB	-0.149238433	0.888872639	0.866664856	0.947534878
ykgI	-0.149674336	1.007741974	0.881928881	0.955717638
fryC	-0.150001389	1.350714655	0.91157404	0.968935805
osmC	-0.152926727	0.857329934	0.858428023	0.944479745
ygiV	-0.153216364	0.579025094	0.791309205	0.913602398
yeiB	-0.153602381	0.911543371	0.866183624	0.947534878
cvpA	-0.153954311	0.808228324	0.848930125	0.94173934
wcaC	-0.155427036	1.390191325	0.910979908	0.968763388
sgcQ	-0.160470315	0.804276034	0.841854871	0.938377687
dhaM	-0.162759137	0.723834086	0.822090548	0.926440659
bcsE	-0.163895016	0.860029359	0.848863205	0.94173934
ydjY	-0.164394712	0.575493075	0.775139395	0.908667209
artM	-0.164614993	0.673698228	0.806963406	0.91948485
hyfC	-0.164880907	1.262060691	0.896056777	0.961537769
fliD	-0.167451831	1.156619572	0.884886997	0.956468768
insJ	-0.168214345	1.15779145	0.884482711	0.956468768
yfeA	-0.169077299	0.665174482	0.799352728	0.917982978
yidJ	-0.169437427	1.101875195	0.877789602	0.953781572
purM	-0.169672988	0.607754028	0.780106415	0.909824916
appB	-0.170524833	0.998612443	0.864411087	0.947114201
rihB	-0.170548079	1.132232816	0.880267699	0.955705745
nudJ	-0.173380972	0.955402052	0.85599526	0.943871671
yqcE	-0.173556392	0.856535705	0.839427377	0.936959963
gspL	-0.1798241	1.037131034	0.862347954	0.946175419
caiC	-0.181714532	0.983829006	0.853463298	0.942579924
smrA	-0.182028092	0.655947824	0.781393196	0.910531625
pstS	-0.182563782	0.731042388	0.802795557	0.918717545
modF	-0.182940092	0.793549457	0.817676885	0.923184599
rsxA	-0.184776238	0.657136496	0.778569312	0.909078338
mqsA	-0.185054852	1.005130879	0.853927001	0.942579924

yciH	-0.185694079	1.001693538	0.852930892	0.942579924
shiA	-0.186853189	0.605631765	0.757682065	0.896603997
oppF	-0.189947151	0.611433912	0.756060237	0.896480902
aroL	-0.191059734	0.750519771	0.79905532	0.917982978
yliI	-0.193064231	1.014376429	0.849052119	0.94173934
ykgM	-0.193128386	1.20151275	0.872300002	0.950363936
napB	-0.194105088	0.702857891	0.782420969	0.910861003
paaZ	-0.196282526	1.267280246	0.876912088	0.953339127
gntK	-0.198036546	0.590247837	0.737237215	0.885202119
setA	-0.198173035	0.645801476	0.758947115	0.897490204
oppC	-0.198257128	0.732566294	0.786672772	0.912495399
ygcE	-0.199504217	0.789934693	0.800609896	0.918382466
efeB	-0.200487592	1.044064403	0.847721778	0.94173934
ansP	-0.202118347	0.639713784	0.752039538	0.894612243
dhaR	-0.205047704	0.96435063	0.831617326	0.932596713
feoC	-0.206270955	1.10289431	0.851639505	0.942546152
yhgN	-0.207358385	0.604541072	0.731597468	0.881723797
kbaY	-0.207765765	1.255434829	0.868555904	0.948834717
yhaK	-0.210136044	0.78347925	0.788538587	0.913188139
ecpC	-0.210226184	1.409001635	0.881394062	0.955717638
yabI	-0.211052584	0.786182553	0.788351118	0.913188139
yjtD	-0.212873029	0.554940827	0.701277975	0.864569483
yeeR	-0.214159679	0.746084678	0.774078067	0.908018658
caiT	-0.21437114	1.072610027	0.841590585	0.938341098
ybaZ	-0.21492413	0.767447897	0.779438799	0.909824916
tadA	-0.217986223	0.555622935	0.694816159	0.861638946
yfhH	-0.218859108	0.560270585	0.696069943	0.861638946
yhfZ	-0.22013912	0.799038967	0.782928391	0.910993273
cobU	-0.221586	0.759176575	0.770380852	0.90629502
yfeW	-0.221586224	0.716561748	0.757142505	0.896603997
betT	-0.222219144	1.31294086	0.865597664	0.947534878
rsxD	-0.22237305	0.591959249	0.707172644	0.867961823
pflC	-0.223163364	1.174813681	0.849343305	0.941804639
gfcD	-0.223348647	1.137983633	0.84440118	0.939917953
insL1	-0.224849908	1.38226532	0.870780087	0.949991062
cobS	-0.225965016	0.629009361	0.719415921	0.874823363
glpE	-0.226112628	0.884442442	0.798216813	0.917982978
rbn	-0.228703221	0.571059061	0.688795934	0.857873645
ybhJ	-0.22877025	1.019566823	0.822461749	0.926440659
yeaE	-0.230831438	0.730842399	0.752121697	0.894612243
acrF	-0.231014078	1.106129189	0.834566046	0.933847847
envY	-0.231921957	0.949919129	0.8071153	0.91948485
csiR	-0.232539185	0.864140569	0.787853659	0.913100613
insF1	-0.232552968	1.563991917	0.881796734	0.955717638
yeaQ	-0.233025868	1.185630664	0.844186196	0.939917953
fecE	-0.235422504	0.836025536	0.778252338	0.908982566
insF1	-0.235531191	1.376117937	0.864100904	0.947114201
paaY	-0.23696899	0.646088924	0.713787175	0.872382637
yiiR	-0.237406864	0.697080651	0.733425041	0.882810308

lsrK	-0.237424808	0.88654719	0.788846784	0.913188139
cspF	-0.237859622	1.035425467	0.818308089	0.923558183
yhaO	-0.238479123	0.59780589	0.689949197	0.858884942
yiaJ	-0.238716117	0.698766584	0.732633139	0.882381089
ygdD	-0.240226172	0.619413475	0.698142985	0.862887701
ulaG	-0.240273421	0.935485392	0.797299523	0.917918719
yebQ	-0.241334657	0.856984303	0.778243192	0.908982566
pliG	-0.241470536	0.913003738	0.791410658	0.913602398
yicO	-0.241911817	1.138783304	0.831771529	0.932596713
atoB	-0.243232131	1.029071809	0.813152791	0.92122904
yeaX	-0.244364355	1.088743459	0.822410109	0.926440659
yejB	-0.245763515	0.787469687	0.754970099	0.896236832
chbC	-0.246060047	0.648176667	0.704228405	0.866446724
atoE	-0.246857697	1.213691259	0.838826975	0.936805663
insL1	-0.247108422	1.430754447	0.862877833	0.94645745
dgt	-0.247923894	0.576070387	0.666925907	0.844612647
vsr	-0.247979861	0.91425072	0.786207716	0.912216966
yfcC	-0.249527068	0.798042147	0.754528315	0.895974748
gsiB	-0.25202329	0.751624097	0.73739505	0.885202119
araE	-0.252163826	0.720524928	0.726359424	0.879528407
sgcC	-0.252351357	0.902635995	0.779806226	0.909824916
rhtA	-0.253029907	0.727536022	0.727997741	0.880260709
astE	-0.254309031	1.219437586	0.834802541	0.933854719
yeeJ	-0.255616934	1.034587727	0.804853045	0.91948485
yfgD	-0.255816437	0.84343931	0.761660077	0.899437795
yjgR	-0.256649038	1.051748115	0.807214253	0.91948485
fliN	-0.256960144	0.731982852	0.725553335	0.879340275
ypeC	-0.257555187	0.938847479	0.783830084	0.910993273
lacZ	-0.257636533	0.777035951	0.740219105	0.887549959
paaK	-0.257988714	0.791488141	0.74445917	0.889394801
hycH	-0.259765894	0.871546075	0.765663613	0.902448923
iap	-0.262084395	0.901403083	0.771241299	0.90629502
yeeS	-0.262380904	0.842553674	0.755487812	0.896382252
yfcF	-0.264165294	0.666109936	0.691677851	0.859949516
yadS	-0.265166671	0.771375639	0.731028168	0.881723797
yejA	-0.265502814	0.743450475	0.72100018	0.875654502
oppD	-0.26668569	0.63289769	0.673483087	0.84783718
baeR	-0.266779973	0.573578531	0.641849111	0.832924551
cheA	-0.270216236	1.064168379	0.799555475	0.917982978
yddG	-0.270459449	0.566229122	0.632899201	0.828909368
hofO	-0.271062439	1.149101969	0.813517504	0.92122904
yicN	-0.27119589	0.716357166	0.705002478	0.866664719
dcuD	-0.272218915	1.226779076	0.824393864	0.928101284
tusC	-0.274585965	0.890946584	0.757933374	0.896603997
ybeQ	-0.274664129	0.58905521	0.641015741	0.832453872
trpR	-0.274844088	0.787820326	0.727189566	0.880046843
phoH	-0.276064887	1.102292971	0.802242445	0.918717545
yecM	-0.276591367	0.729975245	0.704758313	0.866664719
btuE	-0.276871434	0.823411789	0.736683066	0.885155053

yfaA	-0.283008084	1.059595779	0.789399397	0.913255609
yddB	-0.28419136	0.708247196	0.688228616	0.857540136
cynS	-0.284794221	1.159420967	0.805964892	0.91948485
paaH	-0.28536803	1.200232174	0.812066709	0.92122904
yeaJ	-0.290313874	0.586025609	0.620321664	0.819681886
insH1	-0.290622638	1.257899351	0.817285227	0.923184599
sapF	-0.290893147	0.622336209	0.640198548	0.832453872
yfiE	-0.293375776	0.586927925	0.617180878	0.81659637
yecE	-0.293880423	0.649945378	0.65115248	0.835568019
hisM	-0.295500742	0.562332447	0.599241782	0.803083036
cmtA	-0.296047864	1.222045607	0.808581816	0.920008611
torZ	-0.297279347	1.112789539	0.789355317	0.913255609
alsE	-0.299431329	0.884136318	0.734857591	0.883485372
rsxE	-0.299633408	0.706346487	0.671418692	0.84716196
rsxG	-0.299954099	0.6871246	0.662448441	0.841524323
waaP	-0.300264924	0.676104494	0.656962889	0.838288833
yqgA	-0.300374896	0.604312818	0.619152381	0.8186705
yggP	-0.301055728	1.173095911	0.797461699	0.917918719
yoaD	-0.30149616	0.867927146	0.72830964	0.880312249
glsA	-0.302633712	0.943808565	0.748474441	0.892380382
narK	-0.305023909	0.970738741	0.75335522	0.895106114
treR	-0.305194408	0.603207135	0.612889705	0.813666378
elaA	-0.305241494	0.543495861	0.574370752	0.790393425
ykfI	-0.30627316	1.072588646	0.775226037	0.908667209
wzyE	-0.30672257	0.939443732	0.744050767	0.889263505
alsC	-0.30703315	1.150842915	0.789631009	0.913255609
ybiO	-0.307034375	1.183215189	0.795255981	0.91668119
astA	-0.307943574	1.266296497	0.807862539	0.919964812
yehP	-0.308943475	1.224054678	0.800736904	0.918382466
gcvA	-0.310866454	0.636879177	0.625472641	0.823001002
ydhX	-0.311569569	0.828310979	0.706805031	0.867961823
sgcE	-0.312078754	0.79776593	0.695656151	0.861638946
mdtL	-0.313723782	1.044538913	0.763912739	0.901026473
rfaH	-0.31376441	0.625998658	0.616214769	0.815584287
trxC	-0.316571224	0.981054928	0.746934188	0.891395702
opgC	-0.318456357	0.553967224	0.565382615	0.786568676
ykfB	-0.319717184	0.916558326	0.727221737	0.880046843
yghR	-0.325540915	1.209841843	0.787870237	0.913100613
mutM	-0.325661132	0.750033851	0.664146665	0.842971377
lsrR	-0.326477516	0.575089596	0.570239693	0.78845253
araH	-0.326673828	0.61055611	0.592620061	0.800235515
ygdB	-0.326843796	1.02883214	0.750724438	0.894076636
tdk	-0.328756604	0.615463955	0.593230416	0.800584589
ybjH	-0.330807766	0.718902303	0.645403693	0.834598588
oxyS	-0.330889946	2.483175215	0.893993488	0.961285739
lsrA	-0.331932804	1.086672849	0.760017431	0.898282993
hyaC	-0.332887907	0.789219576	0.673175547	0.847795485
agaC	-0.333906092	1.277942862	0.793873462	0.915608255
ddpD	-0.335829776	0.926464657	0.716989441	0.874516201

ybbM	-0.338064948	0.802670001	0.673626925	0.84783718
ybjM	-0.338944831	0.993974803	0.733104018	0.882685979
insH1	-0.339748761	1.080114699	0.753104274	0.89507028
yggC	-0.339785651	0.593269968	0.566825077	0.786799618
yhhA	-0.34067532	0.699707776	0.626341755	0.823727421
cadB	-0.346071481	0.93886135	0.712420188	0.8719979
argA	-0.346469918	0.765031136	0.650632689	0.835568019
torD	-0.350350445	0.762015741	0.645682762	0.834693213
ybcJ	-0.350510338	0.869898777	0.686998009	0.856687151
gspO	-0.353152352	0.949781666	0.710023318	0.870010764
ecpB	-0.356765192	1.32185185	0.787238714	0.912890659
speF	-0.357042265	1.118370588	0.74953528	0.893185193
yciW	-0.358099701	0.680728803	0.598851105	0.803067964
focB	-0.360734113	0.793717747	0.649478605	0.835568019
slyX	-0.363405834	1.327235808	0.784233304	0.910993273
yhjV	-0.36384801	0.612165786	0.552270096	0.778019674
cirA	-0.364521117	0.780056882	0.640284765	0.832453872
ygcP	-0.366476887	0.712538554	0.6070243	0.809969492
ycaI	-0.367471948	0.942841411	0.696721806	0.8619195
rclA	-0.367691764	1.123716471	0.743508723	0.888877724
frvR	-0.368894089	1.032791642	0.720955919	0.875654502
wzxC	-0.369499717	1.279267691	0.772706191	0.90667175
insL1	-0.372370693	1.471783624	0.800263259	0.918382466
ydeA	-0.374232098	0.709429984	0.597838801	0.803067964
bluF	-0.374932824	0.562045745	0.504717229	0.744381716
rutG	-0.377282389	1.050301164	0.719435356	0.874823363
yacG	-0.377509262	1.097769815	0.73093093	0.881723797
ydcT	-0.379335417	1.180491342	0.747955103	0.89235138
yibD	-0.379538023	1.031988727	0.71304219	0.8719979
yeaG	-0.380048149	0.639516465	0.552328718	0.778019674
mtr	-0.380836251	0.840588684	0.650506273	0.835568019
ypjK	-0.380975931	1.444779423	0.792017711	0.913866578
ybaV	-0.384122306	0.589932097	0.514962769	0.750686468
mntH	-0.384853837	0.757086765	0.611218302	0.8127195
rhtB	-0.387379767	0.700370298	0.580190092	0.794025658
yicL	-0.387389697	0.559483832	0.488682697	0.734075985
aat	-0.38846326	0.719619722	0.589322955	0.799302528
hisP	-0.389222791	0.581805547	0.503501091	0.743875155
insH1	-0.391678254	2.092143408	0.851492954	0.942546152
ydjZ	-0.392356923	0.910877929	0.666653148	0.844612647
puuR	-0.396285607	0.585286161	0.49835514	0.741338802
insH1	-0.397572402	2.061571179	0.847077052	0.941351526
yibB	-0.397854	0.69543441	0.567258151	0.787017297
ymcE	-0.399086168	1.439598119	0.781610769	0.910531625
yihL	-0.399538788	0.57515564	0.487267125	0.733796777
dhaK	-0.400066393	0.875400833	0.647664303	0.8353901
gfcE	-0.400448221	0.946838026	0.672344781	0.847495718
cdh	-0.400734165	0.698932982	0.566406827	0.786799618
srlA	-0.400804712	0.673241081	0.551618837	0.777560072

yhjA	-0.400842896	0.746758665	0.591421642	0.79991618
bglB	-0.401209267	1.218507284	0.741956968	0.888070136
ydcR	-0.401288708	0.812790525	0.621505876	0.820166358
tyrP	-0.401608679	0.615457543	0.514054958	0.750179195
potE	-0.403294551	0.909207965	0.657355747	0.838288833
yaaH	-0.404465667	0.768659684	0.598752019	0.803067964
ybiY	-0.405724483	1.1058165	0.713693335	0.872382637
ssuE	-0.406818397	1.105905706	0.712977679	0.8719979
metA	-0.40750541	0.743166077	0.58346057	0.79587791
ddpX	-0.407806692	0.969249668	0.673941493	0.847969837
ydcJ	-0.408138233	0.867484536	0.638008737	0.831525666
ydcS	-0.40892575	0.933221626	0.661250604	0.84061146
yiiS	-0.412401443	0.964502959	0.668957483	0.845108643
ygjV	-0.412694917	0.563397049	0.463856318	0.716098063
ygcU	-0.412760033	0.96487691	0.668807535	0.845108643
fhuE	-0.413940531	0.764628964	0.588258575	0.798660503
prpB	-0.415046748	1.393172706	0.765768203	0.902448923
ssuC	-0.415764787	1.287093062	0.746675396	0.891349251
pnuC	-0.416252137	0.56305046	0.459736783	0.712996526
yggI	-0.417514758	0.715928042	0.559772281	0.783322752
crl	-0.417968239	1.030946576	0.685167474	0.855485032
kduI	-0.419696446	0.7648369	0.58318403	0.795768448
codA	-0.420577879	0.785742551	0.592468919	0.800235515
hcaE	-0.423653992	0.826069571	0.608052683	0.81060361
mhpE	-0.42416303	1.457338951	0.771010703	0.90629502
yidG	-0.424266696	0.648446299	0.512930186	0.749974408
yeaN	-0.425998853	0.807155103	0.597653302	0.803067964
fxsA	-0.42730813	0.87786884	0.62643109	0.823727421
emtA	-0.429103483	0.600864794	0.475138659	0.725503625
yccM	-0.43039615	0.929018778	0.643163424	0.833030199
insH1	-0.43113767	4.419334631	0.922283955	0.971271272
pqqL	-0.431420822	0.707142031	0.541801684	0.769634188
yfiR	-0.43465827	0.714063777	0.542716205	0.770119433
yraK	-0.434722415	0.771691501	0.573205393	0.789593837
yjhX	-0.435389787	1.036323808	0.674391146	0.848009215
cbrA	-0.44027927	1.07358634	0.68173156	0.853796646
yedI	-0.440866159	0.593176649	0.457342976	0.711734519
hofC	-0.442656129	0.923637619	0.631758631	0.828292448
yhfT	-0.444923658	1.091984455	0.683681147	0.855271111
yaaX	-0.444994533	0.745250908	0.550435945	0.77643261
yiaU	-0.445327924	0.729538044	0.541580617	0.769634188
yigE	-0.445650378	0.976886823	0.648250133	0.835484773
mhpD	-0.446492723	1.156421178	0.699423567	0.863943675
yaeR	-0.44709462	0.581953198	0.44232941	0.701566287
yahK	-0.447260666	0.966755266	0.643621683	0.833195635
yiaV	-0.449146738	1.1387315	0.693265626	0.860795972
gspJ	-0.450546589	1.010587317	0.655722591	0.838288833
ygeA	-0.452907963	0.58122933	0.435847653	0.696462904
hdhA	-0.45320822	0.66334256	0.494468489	0.739780307

ylaB	-0.454719967	0.638791445	0.476561276	0.726581201
livK	-0.456747512	1.078149613	0.671828237	0.847414957
ynjD	-0.458175612	0.586371427	0.434582333	0.695263133
yobH	-0.458235855	1.333520738	0.731125619	0.881723797
citT	-0.458599073	1.032992377	0.657077259	0.838288833
waaZ	-0.460744983	0.63596524	0.468770247	0.720119204
gabP	-0.463130029	1.154532983	0.688316627	0.857540136
abgB	-0.463435833	1.026779086	0.65173795	0.835727495
wzb	-0.464040667	1.242768121	0.708856118	0.869238567
gfcC	-0.466024627	1.05448305	0.658528241	0.838465291
otsB	-0.466714845	0.715868934	0.514429739	0.750179195
scpC	-0.469037625	1.030426928	0.648974235	0.835484773
ydiV	-0.470015581	0.74421017	0.527672493	0.7567387
yhaV	-0.472252185	0.862669253	0.584082429	0.796190372
prpR	-0.473227404	1.438695762	0.742210066	0.888110864
yaaY	-0.474068991	1.120839598	0.672325308	0.847495718
otsA	-0.474798445	0.684475485	0.48789071	0.733917973
yiaO	-0.475135975	1.157735463	0.681511697	0.853785049
yicJ	-0.477086953	1.077396301	0.657899747	0.838465291
chpS	-0.478358993	1.131706505	0.672522761	0.847495718
intS	-0.478668894	0.671650941	0.476046835	0.72622959
putP	-0.47960699	0.879960454	0.585731209	0.797370552
yedY	-0.479842203	0.824816403	0.560730791	0.783322752
amyA	-0.482616321	0.832949761	0.562315069	0.784184826
dacD	-0.482760749	0.581267494	0.406238555	0.673575093
ppdB	-0.483482274	0.876000234	0.581002989	0.794025658
rpiB	-0.484154344	0.665247072	0.466747505	0.718100337
acrE	-0.484717002	1.060418699	0.647599431	0.8353901
moaE	-0.484955644	0.641390846	0.449589218	0.706193044
metC	-0.48625798	0.555260088	0.38117677	0.654882068
ybaT	-0.486687652	1.161690912	0.675254408	0.848357316
frlA	-0.487641301	1.209512476	0.686821857	0.856687151
ybgQ	-0.491141113	1.285108085	0.702328719	0.865422682
rutB	-0.491214647	1.289048123	0.703152989	0.865911973
ybdZ	-0.494607878	1.052215449	0.638309786	0.831525666
ydhV	-0.494891583	0.705391361	0.482938376	0.730927125
gspD	-0.495337625	1.066136346	0.642210758	0.833030199
casC	-0.496422544	1.039923413	0.633102572	0.828909368
creD	-0.496852934	0.983288382	0.61335006	0.813666378
chbG	-0.497676347	0.673817167	0.460154443	0.713371363
sfmD	-0.50214249	1.262105821	0.690732763	0.859493745
bioD	-0.502778003	1.070355786	0.638548079	0.831568876
yieL	-0.503174593	1.109509391	0.650180903	0.835568019
srlD	-0.507057281	0.767980422	0.509094908	0.74684004
bluR	-0.508018996	0.619095313	0.41188392	0.678771305
katE	-0.508511811	0.947673895	0.591551235	0.79991618
insH1	-0.50937078	1.801898997	0.777417906	0.908982566
cusC	-0.510575185	1.373688347	0.710129654	0.870010764
gspA	-0.511078299	1.102576239	0.642983779	0.833030199

leuD	-0.512191429	0.631062239	0.417001804	0.683875756
yfjY	-0.512772344	1.138431005	0.652407468	0.835922843
ybbY	-0.51391633	1.153474927	0.655931369	0.838288833
thiB	-0.515347272	0.811242426	0.52526094	0.755815269
yqcC	-0.518379298	0.770397389	0.501028342	0.743473243
rhaS	-0.519891976	0.913476647	0.569264198	0.787912959
ypeA	-0.521367759	0.577766607	0.366852534	0.645121132
xapR	-0.522024776	0.91423123	0.568000574	0.787777738
idnT	-0.524017767	1.067291672	0.623441321	0.821394577
hisQ	-0.524612505	0.62302033	0.399761552	0.666780619
chiQ	-0.524727792	0.952568078	0.581732427	0.794322418
scpB	-0.52596203	0.932836325	0.572869158	0.789593837
yhbQ	-0.528257606	0.643184839	0.411466644	0.678686879
ppsR	-0.528679107	0.570162924	0.353800813	0.633528487
yedZ	-0.529256341	1.027125468	0.606357965	0.809675622
yraJ	-0.529735046	0.962189821	0.581941166	0.794339894
cadA	-0.529945214	0.95014281	0.577012909	0.791871212
yjfF	-0.53202964	0.952421859	0.576429874	0.791653087
mglC	-0.532607042	0.572833078	0.352486537	0.632320079
yjgM	-0.533128575	0.607797704	0.380406225	0.654569964
ydhZ	-0.535874142	1.392212075	0.700305272	0.86432447
insH1	-0.540027374	1.861585993	0.77174743	0.906333743
argO	-0.540027659	0.902050536	0.549395137	0.775504139
fucP	-0.541749613	0.6751115	0.422287227	0.686703737
gsiD	-0.542508095	0.848309686	0.522486942	0.754366262
yedA	-0.542940553	0.940981842	0.563943741	0.785254688
yqiK	-0.544166267	0.891430879	0.541569485	0.769634188
waaS	-0.547706209	0.718431175	0.445842666	0.703834177
ydiQ	-0.547738837	1.135532917	0.629548243	0.826485938
tusA	-0.548126666	0.705812251	0.437400762	0.697582372
mlrA	-0.549060407	0.773115139	0.477584905	0.726905286
kduD	-0.550720147	0.808311978	0.495668225	0.739780307
torC	-0.550973603	1.030203887	0.592774359	0.800235515
ynfM	-0.553133322	0.87549982	0.527523146	0.7567387
yjhF	-0.553153023	1.080216496	0.608597768	0.810996435
ydhI	-0.553292016	1.251228698	0.658345246	0.838465291
tus	-0.55667952	0.606005268	0.358302173	0.636177042
yhcF	-0.55779918	0.88120508	0.526736337	0.756693519
ycbC	-0.558218749	0.683364031	0.414003416	0.680604155
ycgJ	-0.558720488	0.860981691	0.51638089	0.750863748
asnC	-0.560124343	0.691569131	0.417978948	0.684521428
ydhJ	-0.56186341	0.647413556	0.385471755	0.657847271
kdgT	-0.563616157	0.990246929	0.569242619	0.787912959
ycaK	-0.56404528	1.019426685	0.580060355	0.794025658
sad	-0.564650971	0.708876688	0.425716323	0.688805982
mdtJ	-0.565272577	1.017397653	0.578480003	0.793086889
wzc	-0.566610654	1.29855867	0.662591425	0.841524323
yhhM	-0.5676857	0.832223979	0.495156177	0.739780307
ddpC	-0.567822481	0.939078971	0.545405646	0.772562714

mltF	-0.568023562	0.640799247	0.375385955	0.651793399
prpC	-0.569413511	1.454784072	0.695495991	0.861638946
ydhW	-0.570358156	0.707905314	0.420416791	0.685587156
mhpT	-0.572050552	1.604502634	0.721444983	0.875932303
ybjI	-0.573066414	0.61680992	0.35284708	0.632469061
insK	-0.575035016	1.012020274	0.569895752	0.78824885
yafZ	-0.575436253	1.132801046	0.61146986	0.812753708
yqjG	-0.575704944	0.556999655	0.301331512	0.585769243
casA	-0.576160652	1.071612934	0.590813577	0.79991618
feaR	-0.577361982	0.812032892	0.477079203	0.726825579
yddW	-0.577452283	0.797452915	0.468991545	0.720186259
cbpM	-0.578511983	0.686790352	0.399596854	0.666780619
yhbP	-0.578602542	0.726761814	0.425951954	0.688805982
ygbT	-0.579614969	1.045614875	0.579353497	0.794015915
ydcO	-0.581231209	0.855510652	0.49688647	0.740035911
yhgA	-0.585186079	0.788297984	0.45788063	0.712253936
yhdV	-0.585536693	1.314406206	0.655975649	0.838288833
artJ	-0.586613207	0.625969812	0.348693239	0.629388959
hdeA	-0.587073492	0.842721599	0.486028178	0.732560474
mhpR	-0.591538855	1.068012014	0.579668432	0.794025658
ychE	-0.593489186	0.597840151	0.320845356	0.608091198
ssrS	-0.594678552	2.553823256	0.815871559	0.92234894
rsxB	-0.596905814	0.632008005	0.34493501	0.626843182
yejE	-0.597027101	0.755123272	0.429156852	0.690456087
yfjP	-0.598527174	1.084730072	0.581102367	0.794025658
mngR	-0.59891246	0.57882233	0.300805005	0.585717334
yghD	-0.600920482	0.856818255	0.483091172	0.730927125
xylF	-0.602574418	1.134657014	0.595375086	0.802143769
ypfN	-0.604481785	1.046080155	0.563362575	0.784867886
sgcA	-0.606431393	0.822229189	0.460790718	0.713811834
bglH	-0.607284482	1.211434182	0.616164346	0.815584287
gadC	-0.608145007	0.957525082	0.525349012	0.755815269
cadC	-0.609040969	0.771476768	0.429850077	0.69083882
yohD	-0.610187014	0.645199036	0.344284001	0.626843182
ttdB	-0.61369906	1.070597113	0.566488546	0.786799618
motB	-0.614379529	0.934515304	0.510903688	0.747996948
ybdK	-0.614688876	0.949059183	0.517190427	0.750963463
yegI	-0.615830004	0.963077492	0.522536915	0.754366262
hyfE	-0.616177566	1.082449458	0.569190749	0.787912959
yehL	-0.616764703	1.183828153	0.602372518	0.805646453
yehD	-0.619252152	0.644479094	0.33662415	0.620658879
ynjF	-0.620707667	0.805794095	0.441118662	0.70101727
yceI	-0.622501996	0.629364408	0.322616037	0.60989259
sfsB	-0.622859654	0.942037	0.508494415	0.74684004
casD	-0.625256564	0.918571133	0.496071537	0.739780307
entH	-0.626558038	0.751579599	0.404475462	0.672067326
yodD	-0.628811392	1.305136459	0.629949855	0.826632681
xylE	-0.62939003	0.875611167	0.472263649	0.723099271
rspA	-0.631864841	0.942092209	0.502409231	0.743875155

mglA	-0.632252816	0.946409283	0.504098915	0.744215951
xapA	-0.633162245	1.166037959	0.587127657	0.798455182
fadK	-0.633286628	1.058961411	0.549822477	0.775837216
ynjH	-0.634517673	1.149818875	0.581057011	0.794025658
ytfQ	-0.635520642	0.746471827	0.39456614	0.664825907
moaD	-0.635545787	0.889176692	0.474758846	0.725196821
gltK	-0.635634536	0.687503326	0.355197506	0.634039006
yfdN	-0.636132897	1.221742196	0.602591893	0.805646453
eamA	-0.636527437	0.627737567	0.310581579	0.597119553
ygbJ	-0.636974659	0.996975408	0.522883374	0.754366262
ydeP	-0.638014949	0.942585134	0.498483492	0.741338802
gspK	-0.638486917	0.89087563	0.473561753	0.724186852
cysH	-0.638960294	0.698754991	0.360492622	0.637903575
uacT	-0.639640472	0.817119893	0.433745272	0.694746477
rcnB	-0.640647393	0.848557168	0.450258365	0.706403796
ecpD	-0.641209648	1.028201178	0.532875365	0.76280958
gnsA	-0.643077194	1.34907873	0.633590881	0.828909368
ygaC	-0.644631218	0.850338442	0.448398409	0.706193044
metF	-0.645337356	0.923765029	0.484805355	0.732265615
ycgB	-0.645872108	0.734145267	0.378988993	0.65374791
rrlE	-0.646211766	2.440733252	0.791193333	0.913602398
yihG	-0.646289124	0.724589073	0.372425725	0.648973612
yjaA	-0.648523111	0.716305362	0.365268128	0.642823404
ydhU	-0.649737764	0.665537275	0.32893538	0.615301461
bdcR	-0.650867578	0.602613724	0.280109109	0.562159567
yhjD	-0.652699052	0.9655774	0.499061207	0.741640078
fliO	-0.653845488	0.96119238	0.496349885	0.739780307
ynbC	-0.654883349	1.08902158	0.547606758	0.774483305
ugd	-0.655927802	0.590094501	0.266325659	0.548341403
yneF	-0.656145965	0.561720962	0.242766639	0.523497849
ygjI	-0.656604907	1.293656948	0.611763908	0.812878034
dadA	-0.657646784	0.896079588	0.463000182	0.715471269
alsA	-0.657762969	1.007500257	0.513842474	0.750179195
nupX	-0.659907189	1.211414512	0.585931641	0.797370552
ybeF	-0.660025896	0.704309728	0.348694524	0.629388959
ddpB	-0.660626376	1.184096176	0.576901645	0.791871212
metW	-0.660713491	1.973163479	0.737738356	0.885202119
yidB	-0.66244593	0.879349182	0.451247665	0.707408366
ykgG	-0.663005788	0.605228898	0.273313902	0.556688785
pitB	-0.66479837	1.183128923	0.574185174	0.790393425
yphH	-0.667041275	0.826309799	0.419520589	0.685228231
yiaT	-0.667563682	1.039231021	0.520637439	0.75321007
yadC	-0.668485432	1.146285393	0.559775236	0.783322752
yafC	-0.669827216	0.54775017	0.221378825	0.505332069
yadD	-0.670128134	0.849026204	0.429942364	0.69083882
yjfJ	-0.670568998	1.179916506	0.569818363	0.78824885
sufB	-0.671047799	0.97321305	0.490497426	0.73592767
ycjM	-0.671730508	1.21792809	0.581266677	0.794025658
frvA	-0.672826537	1.11916709	0.547716733	0.774483305

cysC	-0.675678288	0.935496724	0.47013042	0.721388616
ybjK	-0.676262993	0.68156001	0.321086269	0.608263428
ygcB	-0.676636302	0.919818955	0.461962595	0.714535048
lsrB	-0.68045269	1.003621872	0.49777355	0.741084822
yciU	-0.681232751	0.885745613	0.441830062	0.701488916
pspA	-0.68157677	0.710153588	0.337176186	0.620759427
ycjN	-0.685214292	1.072137463	0.522751061	0.754366262
ydiO	-0.685889253	0.947647814	0.469200344	0.720234077
yddH	-0.686016288	0.674052621	0.30879732	0.595583075
insH1	-0.688773811	1.484148466	0.642585511	0.833030199
gadB	-0.68935486	1.203269762	0.566711497	0.786799618
elfG	-0.689612468	0.733856367	0.347366123	0.628109841
cobC	-0.690040655	0.552289445	0.211512042	0.495360959
puuP	-0.691450314	0.974592608	0.478028982	0.726905286
ychO	-0.691905761	0.675779089	0.3058996	0.591434224
ydcN	-0.692005021	0.79083454	0.381556844	0.654882068
yfgF	-0.692722025	1.035271351	0.503418157	0.743875155
fdnI	-0.695658139	0.567515017	0.220275121	0.50480234
ydjJ	-0.697523583	1.07869712	0.517868038	0.751602485
chiP	-0.69982323	0.893389738	0.43343056	0.694746477
tynA	-0.699989312	0.920638708	0.4470573	0.704961613
xylG	-0.70040005	1.027182657	0.495324228	0.739780307
ylbA	-0.703201944	0.613193858	0.251470203	0.531799793
alsB	-0.703439229	0.584558381	0.228833694	0.513487682
yjaZ	-0.704950992	0.603146639	0.242488764	0.52345551
sgcB	-0.705152788	0.812679497	0.385564793	0.657847271
setB	-0.707168672	0.779041517	0.36401457	0.641370025
ybiX	-0.709902447	0.632004447	0.261329014	0.543017849
rhaT	-0.710497453	1.055089147	0.500692595	0.743473243
ydcV	-0.710908469	1.061750427	0.503136607	0.743875155
yhdY	-0.712352109	1.096705418	0.515990537	0.750820131
ynbD	-0.71327914	0.982614983	0.467900748	0.719328643
yedP	-0.71593902	0.807989089	0.375577055	0.651793399
elfC	-0.716201511	1.085467992	0.509376431	0.74684004
ybhD	-0.717604728	1.141806547	0.529688312	0.759051402
gltV	-0.718190217	4.593697712	0.875763053	0.952600863
ypdC	-0.719187706	0.816533185	0.378435841	0.65374791
yahI	-0.720408387	1.289745706	0.576457798	0.791653087
yaeI	-0.721523185	0.773631764	0.351003699	0.63145218
nlpA	-0.722577072	1.115285376	0.517059098	0.750963463
ybjG	-0.72369308	0.544010018	0.183421534	0.472775241
ydbD	-0.724660919	1.065959784	0.496618359	0.739908426
yihF	-0.725742089	0.994131115	0.465373968	0.717094577
ydjK	-0.728296794	1.147529352	0.525646881	0.755815269
glxR	-0.728477452	1.232242922	0.554399778	0.779853122
ygbL	-0.730426764	0.82942745	0.378512849	0.65374791
yjfZ	-0.731239882	0.907488203	0.420367146	0.685587156
ydfI	-0.732064899	0.8639198	0.396785581	0.66524762
tqsA	-0.732971732	0.625058782	0.240938404	0.52345551

yeaI	-0.733740246	0.880926696	0.404890629	0.672067326
yhbX	-0.73545818	0.998385128	0.461336576	0.713839114
pppA	-0.735762377	0.947946766	0.43765202	0.697696143
ybhN	-0.739071015	1.082107142	0.494611609	0.739780307
rarD	-0.739110142	0.699845173	0.290920145	0.5746692
yfaP	-0.7407521	0.920269039	0.420860112	0.685857636
yhdZ	-0.743823841	0.919737458	0.418667612	0.684938861
yafX	-0.745181005	0.580096475	0.198938684	0.485231275
srlB	-0.745474211	0.799545491	0.351144063	0.63145218
ycjW	-0.747845185	0.688640494	0.277490808	0.561506402
erfK	-0.747922455	0.58062624	0.197700488	0.485231275
yidP	-0.750131933	0.982654042	0.445240754	0.703431806
yjcC	-0.750541082	1.093485691	0.492476757	0.738525346
insD1	-0.756038035	1.703758728	0.657225421	0.838288833
nei	-0.756982825	0.64170282	0.23814063	0.520998895
aceB	-0.757350607	0.954611765	0.42756822	0.689757885
eamB	-0.762441047	0.661436891	0.249031839	0.528408773
yjaB	-0.764126214	0.739418391	0.30140963	0.585769243
gspF	-0.766396016	1.007529773	0.44685518	0.704961613
yehI	-0.766908132	1.205281874	0.524587785	0.755480952
ybbL	-0.773337603	0.620028947	0.212302003	0.495978403
yhhT	-0.776300472	0.748092191	0.299406505	0.583554793
chbF	-0.777307164	0.839534141	0.354508784	0.633836756
allS	-0.777479553	1.039008159	0.454285175	0.709130947
hydN	-0.778297347	0.963681443	0.419304028	0.685150556
ycbV	-0.779347892	0.847808948	0.357965122	0.636177042
psrO	-0.780688377	1.820417248	0.668031414	0.844991997
ydcZ	-0.783092296	0.62436658	0.209762492	0.493398174
cynT	-0.784098898	1.183214557	0.507532124	0.746201681
ydaS	-0.784414946	0.901540682	0.384255214	0.65700997
insD1	-0.784687036	1.776881177	0.658772041	0.838512357
hyfJ	-0.784754404	0.911619341	0.389328582	0.660043224
yfdV	-0.786322853	1.186633667	0.50755471	0.746201681
ydhS	-0.787270637	0.982228578	0.422833741	0.687146297
lsrC	-0.787302996	1.126616264	0.484663963	0.732265615
ynfL	-0.787390178	0.829490347	0.342495643	0.625712378
yccT	-0.789105274	1.079283798	0.464694936	0.716846754
ttdR	-0.789535123	0.928313756	0.395044602	0.664992388
ydhT	-0.792370627	0.831972475	0.340894133	0.625049668
ydcX	-0.793323436	1.214156976	0.513502012	0.750179195
lsrF	-0.793882891	0.934946251	0.395813731	0.664992388
yfjH	-0.794597416	0.745616379	0.286562891	0.570400668
insD1	-0.794695804	1.796819831	0.658287275	0.838465291
treA	-0.796941356	1.04480571	0.445603485	0.70373063
ygaP	-0.802351126	0.589841784	0.173740721	0.463147989
yjhB	-0.803311148	0.682442231	0.239150684	0.521806712
glyX	-0.803765346	2.908658441	0.782290455	0.910861003
motA	-0.803977862	0.911008257	0.377498722	0.65317107
ydfV	-0.804345918	0.915822877	0.379793563	0.654070987

yfaT	-0.804537564	1.005159373	0.42347447	0.687464692
ybaQ	-0.805893124	1.072766867	0.452515106	0.708213692
idnD	-0.806047047	0.86338606	0.350516349	0.63145218
sbp	-0.809573993	0.827005619	0.327618522	0.61460689
ynfC	-0.809873479	0.67134419	0.227684051	0.511998543
osmY	-0.815133049	0.639732987	0.2026007	0.486866174
yfbP	-0.817788453	0.564385326	0.147340495	0.4313125
yihN	-0.818653561	0.927291832	0.377320837	0.653142047
yfdQ	-0.820174012	0.926891058	0.376229123	0.652087587
yneJ	-0.821879379	0.757147338	0.277702635	0.561506402
gmr	-0.822477028	0.915818653	0.369143709	0.64755889
efeO	-0.823519455	0.871631035	0.344759555	0.626843182
ydiB	-0.824497719	0.652150396	0.206131187	0.489461478
yjgZ	-0.824919533	0.963372257	0.391841121	0.66216086
yaeF	-0.826949988	0.783180954	0.291020194	0.5746692
yaiP	-0.827751551	1.193200632	0.487855503	0.733917973
yeaW	-0.827905826	1.004776686	0.409956616	0.677799396
pgaA	-0.828271882	0.94002728	0.378255726	0.65374791
yhhZ	-0.834566646	1.145534676	0.466283726	0.717666444
yohC	-0.838233554	0.750647438	0.264130846	0.545762716
ytfI	-0.843213735	1.087385942	0.438073634	0.698093754
ygbM	-0.843443206	0.857093929	0.3250795	0.612394188
yadN	-0.84427468	1.160620092	0.466960046	0.71815479
nudG	-0.844827282	0.651624359	0.194805235	0.48257161
ydjE	-0.846801683	1.154064088	0.463097338	0.715471269
fliS	-0.850736898	0.828699919	0.304612514	0.590295953
yieH	-0.852820422	0.644370789	0.185671383	0.473701881
ycfT	-0.85377048	0.975695643	0.381553388	0.654882068
umuC	-0.85493549	0.995877897	0.390630666	0.661494034
ycaM	-0.855634646	0.720753287	0.235172628	0.518992833
frc	-0.856292279	1.143295512	0.453876216	0.709031176
djlB	-0.857795044	1.238073638	0.488405858	0.734075985
idnO	-0.860552294	0.94748502	0.363746696	0.641370025
pspB	-0.863006817	1.089028017	0.428094821	0.690332699
djlC	-0.86494426	1.054736024	0.41218329	0.678988645
oxc	-0.866123071	1.156819951	0.454031804	0.709031176
bfr	-0.869767466	0.686953406	0.205469071	0.489461478
yihO	-0.871593605	1.130057664	0.440539552	0.70082364
yebF	-0.873606684	0.99649748	0.38066196	0.65473211
nadA	-0.87586081	0.584881592	0.134262927	0.418122342
ogrK	-0.876101121	1.546717373	0.571104143	0.788843678
murR	-0.87696025	0.821034835	0.285468296	0.569812147
yfcR	-0.880119316	0.982412655	0.370319321	0.648498717
mhpF	-0.881990824	1.144249937	0.440823947	0.70082364
dinB	-0.882930405	0.999721681	0.377141073	0.653142047
yhcG	-0.88599285	0.612675547	0.148147901	0.4313125
ybaM	-0.88738581	1.44681525	0.539653311	0.768712061
ycaL	-0.889269599	0.788905012	0.259649428	0.541192175
yiiG	-0.88935236	1.055393001	0.399410729	0.666780619

stfQ	-0.892020213	0.944535419	0.344964761	0.626843182
insH1	-0.89351674	1.357276625	0.510335183	0.747434549
yehA	-0.894809135	1.11319013	0.421498528	0.68597151
xapB	-0.895210421	1.00204269	0.371650759	0.648608662
gltU	-0.895988357	2.47370449	0.717198776	0.874516201
fadM	-0.897238426	0.586465362	0.126039344	0.409405752
ycjZ	-0.898608441	0.645636634	0.163977634	0.450994116
yijF	-0.898686391	0.932252883	0.335048823	0.620079601
lsrD	-0.900512725	1.002446877	0.369017819	0.64755889
yddA	-0.90570742	0.716221323	0.206027806	0.489461478
ppdD	-0.913498417	0.927750427	0.324801868	0.612155637
idnR	-0.914754266	0.790890249	0.247430342	0.527105941
ybdO	-0.915232308	0.917383597	0.318446694	0.606095258
leuE	-0.920274664	0.630923152	0.144670801	0.429666981
yfhR	-0.923526711	0.954133944	0.3330836	0.618562032
nemR	-0.923569473	0.636441441	0.1467392	0.4313125
yeeY	-0.924918294	0.615099156	0.132661271	0.416330989
sibC	-0.925587172	1.520625389	0.542730937	0.770119433
ilvH	-0.926846216	0.614326813	0.131370741	0.414456796
yodC	-0.929171958	1.079582524	0.389415908	0.660043224
ssuA	-0.931278552	1.063229092	0.381086348	0.654882068
wcaM	-0.934364582	0.920628813	0.310143971	0.597119553
yqeI	-0.935643341	0.564526539	0.097439685	0.390380788
yeaM	-0.936857491	0.770500581	0.224020133	0.508788564
yjfK	-0.937174031	1.17315256	0.424376846	0.687894336
yfeS	-0.93808881	0.565750622	0.097290969	0.390380788
ycjY	-0.939338435	0.720134104	0.19209936	0.48030403
yeeE	-0.940754888	1.111444567	0.397315486	0.665860678
yeeO	-0.942167602	1.020345428	0.355808679	0.634177488
yafJ	-0.943961373	0.743533481	0.204240923	0.487965088
ecpE	-0.945555074	0.739851811	0.20123777	0.485802033
yghQ	-0.95327983	1.002271942	0.341544	0.625712378
ydiE	-0.955011414	1.547447003	0.537133875	0.766803996
folK	-0.955710972	0.609432317	0.116834766	0.404381925
agaI	-0.955746854	1.001529904	0.339938444	0.62357939
yjdK	-0.958222482	0.921759725	0.298545406	0.582436515
quuQ	-0.960078687	0.704508963	0.172957243	0.462863982
dgoT	-0.962022104	1.131887064	0.395365316	0.664992388
yibA	-0.964172836	0.758336213	0.203575048	0.487965088
holE	-0.965751282	1.159820441	0.405029119	0.672067326
yjeT	-0.970476273	1.085983425	0.371515501	0.648608662
recE	-0.973162762	1.210478924	0.421426696	0.68597151
blc	-0.975380478	0.652101885	0.134719182	0.418506946
pspD	-0.977193686	0.813975415	0.229937488	0.514905463
dosP	-0.980730094	1.112257194	0.377913323	0.653609475
uidC	-0.981686617	0.981014565	0.316979093	0.604152912
htrE	-0.982690863	1.167633604	0.400006892	0.666787804
fixX	-0.984719106	1.216062171	0.418077842	0.684521428
yjfM	-0.984784114	1.110390295	0.375142944	0.651793399

frwD	-0.987305261	1.024341937	0.335124421	0.620079601
gatD	-0.9936721	1.1594812	0.391446452	0.661769773
insB1	-0.994271543	1.524600212	0.514302924	0.750179195
yccX	-0.99555715	0.679386442	0.142818136	0.427567449
yncG	-0.997340423	1.042273986	0.338623327	0.623140702
mutT	-1.000417162	0.810870715	0.217293585	0.500762051
cysD	-1.000563371	1.088431556	0.357953936	0.636177042
chbA	-1.0006947	0.737227259	0.174661722	0.464923585
ybdR	-1.000769507	0.889923325	0.260776803	0.542148286
yieK	-1.000948671	1.119935419	0.371452613	0.648608662
atoA	-1.002847362	1.3332621	0.451945401	0.707954658
yhjH	-1.003376723	1.039758749	0.334540221	0.619847375
ybbW	-1.005331412	1.164060571	0.387784673	0.659152648
yqiI	-1.008008184	0.782441527	0.19764655	0.485231275
eutP	-1.008099244	0.789141715	0.201438879	0.485802033
yfeN	-1.009467069	0.863149519	0.242195847	0.52345551
yhiD	-1.009651083	1.169830598	0.388096448	0.65940612
citC	-1.010637275	0.898169339	0.260496211	0.54191833
ydiY	-1.013424073	0.579947409	0.080560813	0.37803293
ydjL	-1.0148984	1.049324647	0.333448062	0.618672057
ompN	-1.015013073	1.098158347	0.355337104	0.634039006
aroM	-1.015185417	0.626010611	0.104873071	0.396303494
etp	-1.015554218	0.703341625	0.148767408	0.4313125
yiaC	-1.018734886	0.628017	0.10477222	0.396303494
nac	-1.022235919	1.049208248	0.329911236	0.61580782
torY	-1.024120474	0.977072664	0.294568672	0.578856712
ybdN	-1.027773633	1.112589068	0.355607374	0.634177488
yfeK	-1.029780026	0.801662504	0.198947637	0.485231275
yhaC	-1.03204254	1.163214309	0.374953275	0.651793399
yfbN	-1.032664244	1.03647097	0.319091181	0.606705216
leuO	-1.032813289	0.868270883	0.234240673	0.517780307
ydcU	-1.035264488	1.098693334	0.34605502	0.627418179
nth	-1.035408091	0.593270295	0.08094045	0.37803293
cbrC	-1.035871264	0.708423374	0.143680763	0.42798076
ppdA	-1.037440443	0.715086662	0.146838398	0.4313125
ydiZ	-1.037982911	0.911216119	0.254653836	0.534627992
yjgN	-1.040962592	1.146806086	0.364033653	0.641370025
sgcR	-1.041254798	0.823256856	0.205943238	0.489461478
cynR	-1.044284585	0.881570636	0.236186402	0.519106803
ydcK	-1.046372957	0.840877891	0.213359159	0.49681679
ulaC	-1.056834233	0.894289639	0.237301605	0.519997013
ybfC	-1.059974422	0.765640751	0.166227469	0.453730509
yfcQ	-1.060024213	0.762487753	0.164462683	0.451715257
yajI	-1.060696346	0.72775171	0.144979083	0.429756275
kch	-1.062746205	0.731978065	0.146534256	0.4310957
pspC	-1.064540653	0.776351216	0.170309461	0.46076355
ygfI	-1.064965409	0.567235223	0.060454117	0.36375562
ynbE	-1.0680233	1.657157516	0.519257015	0.752078578
adiY	-1.068089452	0.960382826	0.2660738	0.548164829

yjhI	-1.068818094	0.836638366	0.201420475	0.485802033
yjeJ	-1.073470644	0.660768867	0.104252482	0.396303494
yfiL	-1.077930179	0.942241791	0.252621185	0.532458247
ccmD	-1.078560027	0.832125107	0.194923369	0.48257161
ybaK	-1.079797706	0.778776858	0.165585037	0.453568742
pphB	-1.079923339	0.951414806	0.256345578	0.536512635
yfcP	-1.081174918	1.062813813	0.30902218	0.595708949
yfcI	-1.08269245	0.701762539	0.122874743	0.409386299
yghS	-1.086287689	1.101061532	0.323847509	0.611209405
pagP	-1.090722201	0.677023683	0.107167796	0.396303494
hdeB	-1.097758623	0.643426828	0.087987116	0.383960999
yhdT	-1.102065721	0.830289444	0.184400487	0.472916736
ygjQ	-1.102796459	0.886549319	0.213528995	0.496926834
mmuP	-1.10296293	1.457242513	0.449119614	0.706193044
yfbK	-1.105052327	1.067488873	0.300580835	0.585562088
cysP	-1.106447979	0.81344419	0.173766179	0.463147989
gadX	-1.107864589	0.751822171	0.14059669	0.426950547
wecH	-1.11027179	0.897919766	0.216275248	0.49987449
insB1	-1.115236299	0.911570271	0.221169862	0.505144582
ecpA	-1.116897297	0.663408822	0.092264186	0.387027055
dsdC	-1.11704845	0.732620504	0.12732646	0.409405752
ydeE	-1.119144328	0.850822644	0.188386473	0.476558741
yliF	-1.119302241	1.021104675	0.273005273	0.556688785
yhaH	-1.11968158	0.650528076	0.085216586	0.382674906
yecR	-1.120378263	1.035127439	0.27909357	0.561943644
yehE	-1.120389732	1.075697551	0.297621677	0.582202085
setC	-1.121877681	1.106095234	0.310454596	0.597119553
yqjF	-1.122920482	0.611955431	0.066509983	0.367589495
hcaC	-1.123778481	0.681339321	0.099072504	0.393168651
yaiL	-1.125468909	0.738105343	0.127307002	0.409405752
hyaE	-1.126023878	0.85563637	0.188171526	0.476558741
gadW	-1.126585916	0.71682265	0.116034464	0.403550127
yjgL	-1.126961409	0.840398431	0.179925431	0.46975269
hcaR	-1.1272159	0.562018449	0.04489292	0.346511754
ymiA	-1.127492175	1.615470391	0.485218291	0.732515133
ydfU	-1.13016246	1.018261368	0.267044589	0.549263705
lsrG	-1.130436059	0.986971809	0.252060888	0.532458247
ybhI	-1.131909436	1.164715214	0.331133313	0.616918405
ddpA	-1.132156758	0.943815263	0.230312854	0.514941258
cbtA	-1.134293103	0.668745225	0.089857225	0.38548274
yfbL	-1.135643702	1.0520756	0.280395347	0.562455585
rutR	-1.136510399	0.764957778	0.137354122	0.423017114
ynaI	-1.144440428	0.630090373	0.069322593	0.367589495
flgN	-1.145714264	0.873995886	0.189894235	0.47904868
ykgD	-1.146591444	0.608426452	0.059494755	0.36375562
yfdE	-1.14671377	1.075971389	0.286537819	0.570400668
ydaM	-1.150824845	0.79061389	0.145500685	0.430240539
fliJ	-1.15587264	0.740068651	0.118324471	0.405691946
ompG	-1.156222332	1.220580609	0.343499987	0.626612068

yhiJ	-1.156434	0.99980696	0.247412409	0.527105941
ycaP	-1.158909553	0.577773849	0.044875602	0.346511754
ybeR	-1.159616955	1.073129623	0.279877951	0.562128506
yihR	-1.160139738	1.066142555	0.276521973	0.560426315
hipA	-1.164967799	0.875398817	0.183259696	0.472775241
yedV	-1.166435472	0.829232116	0.159532588	0.443535602
yeiL	-1.166787165	0.977664008	0.232695563	0.516947839
dkgB	-1.167503687	0.721675223	0.105712425	0.396303494
ydhY	-1.169190928	0.558312038	0.036246337	0.328323548
entD	-1.173715496	0.597515396	0.049492208	0.357424257
psuT	-1.176499967	1.010185689	0.244165825	0.524806542
bglJ	-1.176576648	0.933895378	0.207720173	0.490627705
ycbU	-1.177393186	0.920320395	0.200780989	0.485440792
ykgH	-1.178156551	0.708998212	0.096568917	0.390380788
potH	-1.181771858	0.990661293	0.232903794	0.516947839
yfeZ	-1.182447607	0.825610723	0.152083844	0.434494647
zraP	-1.182475557	0.82954183	0.154025368	0.43699194
yehB	-1.184642292	1.162643608	0.308240349	0.595050655
ybjO	-1.185499896	0.630253536	0.059973431	0.36375562
mntP	-1.188177927	0.678005604	0.079694685	0.377432539
yegK	-1.188855517	1.226252612	0.332294312	0.618229069
ycaN	-1.19216026	0.912630509	0.1914539	0.48030403
recT	-1.193163249	0.902463682	0.186128945	0.473701881
ydiM	-1.197260739	1.103572221	0.27796786	0.561570911
yeaV	-1.197307709	1.048204479	0.253351647	0.533415372
hofP	-1.198428397	0.960753326	0.212256703	0.495978403
ybfB	-1.206896111	0.884433535	0.172379477	0.462863982
yqhG	-1.207374603	0.862595445	0.161603258	0.446280386
ybgO	-1.207686556	0.737119594	0.10134116	0.395053485
ybgA	-1.208011374	0.618072146	0.050644148	0.357424257
cheY	-1.209026525	0.887206217	0.172966199	0.462863982
chpB	-1.209661617	0.650248147	0.062842009	0.36375562
ybdM	-1.211150271	0.840358397	0.149519535	0.4313125
mcbA	-1.211324986	0.740291691	0.101780859	0.395053485
recX	-1.212025963	0.702503593	0.084474316	0.382635615
codB	-1.215206561	1.068657396	0.255482311	0.535535309
yidH	-1.217105432	0.603550143	0.043739659	0.346511754
ydjX	-1.217237652	0.625735667	0.051740024	0.357424257
yeeT	-1.220167981	1.293055435	0.345357934	0.626990813
yciQ	-1.220487472	0.936146043	0.192323547	0.48030403
ybaA	-1.222416701	0.827842906	0.139775159	0.426485017
emrK	-1.222629932	0.910599347	0.179380451	0.469426815
potF	-1.225769281	0.839169734	0.144099736	0.428546609
envR	-1.227623308	0.957937629	0.200008486	0.485231275
gatC	-1.229028733	1.076754327	0.25369526	0.533415372
ydjG	-1.229844623	0.692998709	0.075952247	0.373744519
ybcV	-1.231783003	0.642582588	0.055247386	0.35965674
trkG	-1.235000497	0.789394936	0.117702536	0.405562888
ydjM	-1.236017625	0.808136117	0.126148682	0.409405752

ygcW	-1.236618998	0.716647537	0.084426431	0.382635615
yfbM	-1.238552376	0.637864088	0.052171212	0.357424257
yidI	-1.238641628	0.64889106	0.056280266	0.360656205
ycgG	-1.238970823	1.011953545	0.220825626	0.505144582
ldrB	-1.240709635	2.35943366	0.598992006	0.803067964
yciY	-1.243757407	1.644056854	0.44933912	0.706193044
alkB	-1.244779621	0.927037704	0.179353028	0.469426815
agaV	-1.245708696	0.654933811	0.057166247	0.362679132
ybgD	-1.245808007	1.015582764	0.219938096	0.504315069
ycdT	-1.246502082	0.966301458	0.197060287	0.485231275
mobB	-1.251224	0.678198962	0.065048926	0.367589495
ybgP	-1.252968774	0.993711311	0.207345537	0.490627705
arpA	-1.25323148	1.149705948	0.275693222	0.559159889
pgaD	-1.253823249	0.92904295	0.177148855	0.466641624
elfD	-1.255161359	1.012751542	0.215213082	0.498706873
ycgR	-1.255309377	0.984078114	0.202090005	0.486536435
nanS	-1.257882212	0.800145996	0.115935276	0.403550127
sgrS	-1.261489773	1.245435877	0.311112618	0.597183028
hipB	-1.261633731	1.133815129	0.265823034	0.5481417
csgD	-1.270011639	0.989004873	0.199096186	0.485231275
ymdF	-1.270653593	0.677136489	0.060585242	0.36375562
abgT	-1.271030917	0.906515018	0.160883342	0.445202094
ydjF	-1.272177387	0.584370388	0.029480178	0.312860316
yfjW	-1.272410941	0.768487608	0.09777573	0.39091007
thiS	-1.275534656	0.994823184	0.199782237	0.485231275
yegS	-1.276449623	0.928335327	0.169135269	0.458645071
yjeN	-1.276699238	0.717040904	0.074992366	0.373744519
paaI	-1.27863106	0.76778168	0.095841432	0.390380788
ompL	-1.28092721	1.060477651	0.227094343	0.511182936
ynjI	-1.284113536	0.86283303	0.136684279	0.422024424
iraM	-1.290660741	0.904749147	0.153712564	0.436807564
yadV	-1.290910522	0.979433579	0.187497815	0.475667174
asr	-1.294391241	0.803224061	0.107071962	0.396303494
wcaA	-1.297429606	1.10638853	0.240927815	0.52345551
narU	-1.297588585	1.084359199	0.231446546	0.51581338
ilvM	-1.29895692	0.756188076	0.085838641	0.382674906
allA	-1.299547944	0.647866124	0.044868024	0.346511754
ydgC	-1.299669183	0.738942174	0.078607418	0.374816185
paaA	-1.30048565	1.074654839	0.226224162	0.510641844
yedW	-1.302549594	0.673715406	0.053189092	0.357424257
yhdJ	-1.302972293	0.988942583	0.187657384	0.475774255
pyrI	-1.30463556	0.598619451	0.02930143	0.312860316
yhjR	-1.304729312	1.298644845	0.315048436	0.602171787
rzpQ	-1.306162043	1.07414664	0.223985018	0.508788564
yjjQ	-1.308748039	1.026273934	0.202223457	0.486536435
yciF	-1.312580135	0.873188134	0.132786376	0.416330989
yraH	-1.313177032	0.87761246	0.13457386	0.418376097
yecT	-1.315018772	0.651149628	0.043431754	0.346511754
ycjP	-1.316891305	0.995536246	0.185903248	0.473701881

gspC	-1.318460701	0.949834199	0.165107927	0.453159932
psuG	-1.323276567	1.181857391	0.262859355	0.544753837
ybeU	-1.323511326	0.890294415	0.137120592	0.423017114
ydcD	-1.325572927	0.625726562	0.034136638	0.326714539
flhE	-1.325682306	0.834093753	0.11197723	0.400636634
zinT	-1.329201629	0.669546011	0.047119087	0.352557243
pgaB	-1.330359809	1.148591231	0.246760603	0.526615058
cnu	-1.33093275	1.402224783	0.342540303	0.625712378
yjbM	-1.335096453	0.931976948	0.151988665	0.434494647
yaiY	-1.335405717	0.64599446	0.038714442	0.336799028
yhdX	-1.335411598	1.029014766	0.194370689	0.482342452
ygbF	-1.338360654	0.877198671	0.1270793	0.409405752
yidX	-1.350466737	0.5961904	0.023502854	0.286927951
yfdC	-1.356546147	0.818650446	0.097509015	0.390380788
chbR	-1.356728159	0.710849629	0.056313611	0.360656205
ygiL	-1.360646308	0.908858128	0.134368727	0.418122342
yjcF	-1.360744185	1.104776393	0.21806418	0.502005784
yhiM	-1.361837064	0.82601732	0.0992135	0.393168651
ybjE	-1.362910201	0.649248429	0.035798202	0.32752865
ygbE	-1.362919024	0.752032374	0.069937647	0.367589495
ydiF	-1.366142284	1.161949863	0.239701764	0.522727785
ygeH	-1.36686315	1.165923314	0.241058991	0.52345551
yafT	-1.373266677	0.89210146	0.123716058	0.409405752
ybdF	-1.373587013	0.728858734	0.059487599	0.36375562
yccE	-1.374534237	1.033841908	0.183669857	0.472916736
yiaM	-1.377271659	1.073437148	0.199475171	0.485231275
mhpC	-1.382326247	1.198472888	0.248743634	0.528238183
yraI	-1.387210668	1.041069818	0.18270063	0.472775241
ybhM	-1.38985363	0.743755896	0.061665128	0.36375562
ydeO	-1.392181347	1.113683984	0.2112745	0.495360959
phoE	-1.397298003	1.155547442	0.226582682	0.510882198
bglG	-1.398475548	1.055285674	0.185101377	0.473439107
pgaC	-1.404870858	1.026940449	0.1713071	0.462061866
ydeJ	-1.405022418	0.769546048	0.067883283	0.367589495
sfmF	-1.408380417	1.042599398	0.176748091	0.466641624
ycaC	-1.411607506	0.5784086	0.014666853	0.253499259
citD	-1.414494961	1.636244611	0.387326202	0.658855774
hdeD	-1.416118937	0.748377042	0.058457238	0.36375562
ves	-1.41682836	0.705336831	0.044566211	0.346511754
ydeM	-1.421817181	0.846743636	0.093121064	0.387027055
yfjZ	-1.423499216	1.090542467	0.191786303	0.48030403
yfcS	-1.42482406	0.869979362	0.101470171	0.395053485
yohJ	-1.427039186	0.599327268	0.017262509	0.263454633
ybcI	-1.427276095	0.75690332	0.059338331	0.36375562
frwB	-1.429847081	0.768665689	0.062861804	0.36375562
yliE	-1.43008786	0.962560932	0.137355444	0.423017114
yqiJ	-1.430519029	0.615812462	0.020180314	0.277602331
yjeO	-1.433758202	0.64838605	0.027016977	0.30007349
insE1	-1.442577305	1.077114006	0.180473477	0.47047263

yadK	-1.443552161	1.011938615	0.153717874	0.436807564
cbl	-1.443771576	0.848630446	0.08888742	0.385449519
yahB	-1.446200842	0.747816559	0.053125688	0.357424257
aqpZ	-1.447762279	1.084326062	0.181819913	0.472195982
yjjB	-1.449112093	0.572769948	0.011406019	0.225664635
insB1	-1.452122878	1.198342682	0.225598145	0.510610481
yagK	-1.455274038	0.607882228	0.016665451	0.262886141
fliZ	-1.455395389	0.753142611	0.053306452	0.357424257
dosC	-1.456117793	1.135467786	0.199704247	0.485231275
insH1	-1.458870134	1.325146896	0.270934954	0.554732477
csgA	-1.459888806	0.794886011	0.066268752	0.367589495
oppB	-1.461801953	0.855294091	0.087428352	0.383361441
yfdR	-1.462514793	1.035876704	0.157990645	0.442556244
hyi	-1.4627087	1.083362336	0.176965788	0.466641624
yedK	-1.46554105	0.839277799	0.080777414	0.37803293
soxR	-1.475506686	0.826050991	0.074064059	0.373744519
dinI	-1.478916132	1.258689534	0.240008724	0.523115789
paaC	-1.481083218	0.946802954	0.117747291	0.405562888
perR	-1.481752606	0.801477998	0.064490287	0.367196101
ykfF	-1.486358802	1.028023005	0.148221533	0.4313125
insB1	-1.487384609	1.725000806	0.388549111	0.659622318
ydiP	-1.500200637	0.718509519	0.036803766	0.332031619
slp	-1.504586424	0.807453086	0.062409781	0.36375562
elaD	-1.507154417	1.021210255	0.139983833	0.426485017
yhfY	-1.507179194	0.987758402	0.127045199	0.409405752
insE1	-1.507286282	1.343052878	0.261741857	0.543318734
npr	-1.510860214	1.301516253	0.245704446	0.525469563
yhjB	-1.512059656	0.763838557	0.047753668	0.353745052
paoA	-1.512228715	0.979251237	0.122522837	0.409149571
yneK	-1.512464494	1.06430741	0.155293908	0.439945145
emrY	-1.516945275	1.068742086	0.15578986	0.440489098
ydjI	-1.517696237	1.051578519	0.148948576	0.4313125
yahN	-1.519396733	0.903737586	0.092716961	0.387027055
ygjH	-1.52046125	0.678621017	0.025057443	0.295116348
tfaR	-1.520914151	1.354610291	0.261535706	0.543168929
sdiA	-1.521029949	0.824710434	0.065136518	0.367589495
pphA	-1.522319945	0.634397227	0.016411557	0.262685063
yafW	-1.52356963	0.66480278	0.021919391	0.27856179
ynfA	-1.530041673	0.598789618	0.010611945	0.219558829
elfA	-1.53524539	0.572975956	0.0073749	0.179028999
sufE	-1.535975341	0.583055653	0.00842974	0.195280949
ykfH	-1.538587294	0.612516715	0.012007947	0.231521653
cheW	-1.53934267	0.613116824	0.012049747	0.231521653
ycdU	-1.543794745	0.97308544	0.112627168	0.400636634
metZ	-1.544892288	1.945668245	0.427185946	0.689690094
mdtI	-1.549456593	0.754091134	0.039904717	0.338654556
rybB	-1.549594476	2.512321455	0.537368069	0.766803996
umuD	-1.556804505	0.701592809	0.026489628	0.299970731
ygbA	-1.561235476	0.686945987	0.023043094	0.283941352

gspG	-1.562016374	0.887535456	0.0784166	0.374816185
yjcH	-1.568259022	0.756921802	0.038275663	0.336427168
bhsA	-1.569155978	0.91457277	0.086212264	0.382674906
yhdU	-1.57076954	1.072293099	0.142956504	0.427567449
ydjH	-1.571371039	0.998260932	0.115462363	0.403550127
arsR	-1.572732431	0.787648273	0.045852935	0.350071185
pyrL	-1.574830906	1.46214035	0.281447956	0.564008904
eutN	-1.575259602	0.989170245	0.111271044	0.400259817
araJ	-1.578519269	0.888248052	0.075549259	0.373744519
yfdX	-1.579004326	1.03772932	0.128110451	0.409405752
yjfY	-1.579843986	0.877788752	0.071892262	0.37116173
ydiN	-1.580523644	1.106764021	0.153275053	0.436372057
ybhH	-1.582074109	1.129208514	0.161200043	0.445774198
ybdJ	-1.583909577	0.836790319	0.058379247	0.36375562
insI1	-1.587390618	1.688614039	0.347189183	0.628070035
yfjX	-1.588491232	0.826690746	0.054667946	0.359195874
yahM	-1.589271586	0.632831232	0.012026453	0.231521653
ygaV	-1.591010716	1.123706755	0.156816689	0.441176167
yaiO	-1.591425922	0.753292295	0.034632674	0.327274732
wcaD	-1.591901686	1.215095446	0.190160577	0.47942225
sfmA	-1.594327099	0.747344067	0.03289815	0.324499023
nrdF	-1.594387508	0.823311229	0.052799759	0.357424257
appY	-1.594846499	0.822979108	0.052636123	0.357424257
napD	-1.599005674	0.869493475	0.065913973	0.367589495
psuK	-1.603319403	1.095585523	0.143348087	0.427567449
yqeH	-1.605768687	0.868472216	0.064463872	0.367196101
ycfZ	-1.605893832	0.934368189	0.085669943	0.382674906
acpS	-1.611019907	0.662909535	0.015089416	0.255951844
yfcG	-1.611311024	0.931196715	0.083564926	0.381205511
yfgI	-1.617736802	0.811231438	0.046133021	0.351547495
yneE	-1.618432772	0.721862157	0.024959777	0.295116348
sufC	-1.624858232	0.816162864	0.046497327	0.351679413
yjdI	-1.626295972	1.052747456	0.122391958	0.409049461
yegP	-1.628065458	0.674924972	0.015855844	0.258150962
psiF	-1.629425133	0.615925944	0.008157301	0.191154321
fliR	-1.630212254	0.784046537	0.037596312	0.334793019
yfjS	-1.631793832	0.811634964	0.04437807	0.346511754
arsC	-1.63394414	0.578827923	0.00475989	0.150754652
ygcG	-1.634593863	1.067797871	0.125816773	0.409405752
insA	-1.639161103	1.073609631	0.126816795	0.409405752
yehR	-1.639875664	0.708458981	0.020628918	0.277602331
yidL	-1.643815101	0.898660192	0.067372006	0.367589495
fliT	-1.644852277	0.775313746	0.033877128	0.325991311
yhhI	-1.645089784	1.187503233	0.165950058	0.453730509
ulaB	-1.65081162	0.698580576	0.018123069	0.268142043
rspB	-1.658269923	1.07199477	0.121887088	0.408034893
gspB	-1.663666163	0.599956872	0.005554617	0.164265687
ydjO	-1.663756422	0.914766333	0.068945383	0.367589495
sfmC	-1.666615426	0.916493292	0.06899235	0.367589495

yafY	-1.666918419	0.652914393	0.010678636	0.219752234
yhbO	-1.668435147	0.651009231	0.010381866	0.216948897
intF	-1.6691033	0.815015792	0.040565896	0.339781285
tyrT	-1.669147883	2.868872758	0.560692549	0.783322752
ybfP	-1.669600831	0.748689782	0.025745437	0.297356134
ycjD	-1.678451854	0.704414076	0.017183249	0.263454633
ydeQ	-1.690336848	0.996022073	0.089680074	0.385449519
pspG	-1.692651588	0.698593249	0.015395344	0.257639363
yqiH	-1.692940552	0.876516656	0.05342849	0.357424257
idnK	-1.695127607	0.801890394	0.034522584	0.327274732
gfcB	-1.705113109	0.941802958	0.070221763	0.367589495
yfjT	-1.708812914	0.712423817	0.016458314	0.262685063
yebW	-1.71011999	1.113503196	0.124587047	0.409405752
yigF	-1.713682826	0.615289418	0.005350042	0.161858725
ydbL	-1.713777799	0.584377472	0.003360766	0.120571195
yhfU	-1.718113962	0.604827517	0.00450192	0.144847498
yegL	-1.722900749	0.943234191	0.06776166	0.367589495
fliE	-1.727314529	0.864925422	0.045817841	0.350071185
yghT	-1.735191435	0.959571488	0.070560096	0.367589495
agaD	-1.736192202	0.990393384	0.079596357	0.377407758
yagN	-1.741096558	0.746679153	0.019711912	0.277602331
wcaB	-1.747073184	0.922034277	0.058118524	0.36375562
isrC	-1.747758512	0.988957312	0.077182357	0.374512315
yodB	-1.749576623	0.773171262	0.023644456	0.286927951
yiaG	-1.751039582	0.916783076	0.056135473	0.360654848
sfmH	-1.754012685	1.139604822	0.123769839	0.409405752
yjdO	-1.754811015	1.443523346	0.224120428	0.508788564
ydhO	-1.756709801	0.814787169	0.031080918	0.317916139
sibA	-1.765146922	1.534954071	0.250157358	0.529852627
dctR	-1.765580009	0.946820526	0.062216983	0.36375562
yjgH	-1.76592372	0.615254588	0.004101701	0.137423924
rutC	-1.766917219	0.744009705	0.017555714	0.265562929
lacY	-1.768746155	0.912413355	0.05255786	0.357424257
ymfA	-1.772543103	0.643739655	0.00589601	0.164265687
yafQ	-1.780747943	1.109836747	0.108600931	0.398282207
insD1	-1.783094704	1.16583317	0.126150554	0.409405752
chaB	-1.786579756	1.027898354	0.082194973	0.379645316
mcbR	-1.791613956	0.626442546	0.00423666	0.140782133
pinR	-1.792056159	0.727305774	0.013740943	0.249801717
sibD	-1.793876444	1.74662098	0.304394396	0.590295953
insD1	-1.795062491	1.529765378	0.240626058	0.52345551
casE	-1.796636819	1.014330131	0.076518388	0.373744519
yedL	-1.799258822	0.817999766	0.027836456	0.305485257
yoaC	-1.807885856	0.850542324	0.033539216	0.324660621
ylcI	-1.808178683	1.051257879	0.085429825	0.382674906
gspI	-1.809012529	0.981888609	0.065419497	0.367589495
yjdN	-1.81194874	0.897121172	0.043410685	0.346511754
hycA	-1.815512998	0.828846898	0.02849465	0.310222676
psiE	-1.816789647	0.624687464	0.003633789	0.126994659

yphF	-1.821565313	1.097285463	0.096901355	0.390380788
ytjA	-1.821733477	1.111548024	0.101230823	0.395053485
yiaA	-1.822822135	1.036864976	0.078745293	0.374816185
yahC	-1.82347211	1.170910332	0.11939655	0.406500994
intR	-1.82484015	0.84107381	0.030032978	0.315424071
ydaV	-1.830679688	0.928589018	0.048670843	0.355636903
yjfI	-1.831069682	0.901779988	0.042305131	0.345787635
insD1	-1.837421007	1.264950001	0.146344809	0.430851022
ryjA	-1.840558105	1.612453674	0.253676812	0.533415372
ygeG	-1.844899437	1.081776358	0.088113295	0.384098171
nrfF	-1.847173973	0.687789683	0.007238522	0.179028999
yggM	-1.851854751	0.893056304	0.038115101	0.335910045
yahO	-1.858383141	0.562175107	0.000947389	0.060963749
yfjJ	-1.861931424	0.966343964	0.054007156	0.357756229
fepE	-1.862948515	1.142300739	0.10291692	0.39523855
arfA	-1.864489494	0.937892621	0.046816529	0.352374686
yfdF	-1.868450609	0.871655147	0.032067652	0.321852891
yhaB	-1.875180156	0.667493311	0.004965139	0.153703466
ydaU	-1.877617886	0.913824309	0.03990978	0.338654556
yafL	-1.88023711	1.224978292	0.124804798	0.409405752
yfdK	-1.886101777	0.592016673	0.00144308	0.080714133
gspM	-1.888593511	0.820193358	0.021300241	0.277602331
bcsF	-1.896259754	1.130357599	0.093430079	0.387027055
ycgX	-1.901265596	0.596509655	0.001435991	0.080714133
fliQ	-1.905823827	0.836359531	0.022684097	0.283101448
yfjM	-1.91219283	0.873670447	0.028619458	0.310222676
iraP	-1.913003407	0.681231256	0.004982614	0.153703466
yaiV	-1.915903792	0.780840892	0.01414178	0.250352735
ynbB	-1.919474635	1.198551732	0.109267321	0.398679812
aaeX	-1.921503941	0.917734053	0.036282425	0.328323548
ydfC	-1.923146272	0.943916488	0.041608826	0.344194738
ybfD	-1.933573319	1.030885549	0.06070431	0.36375562
cmtB	-1.935774732	0.919246762	0.035219375	0.32752865
ybeT	-1.947403761	1.022825507	0.056917316	0.362203401
yjfL	-1.948326997	0.999477846	0.051254255	0.357424257
yqhH	-1.953499313	0.75291545	0.009470659	0.209803562
yadM	-1.953844879	1.019767122	0.055368706	0.35965674
gadE	-1.953846039	0.650134645	0.002653239	0.106497321
yqeJ	-1.961493797	0.834358744	0.018728077	0.273106558
ydeS	-1.962057617	0.97889104	0.045030683	0.346511754
ybfK	-1.962162873	1.005541352	0.051015454	0.357424257
yfbO	-1.962474602	0.620559378	0.001564592	0.08417859
rcsA	-1.977703882	0.612710482	0.001247483	0.075482032
yfjI	-1.979795548	1.123271006	0.077980288	0.374816185
fimZ	-1.986673278	0.823284111	0.015817142	0.258150962
yeaR	-1.993586093	0.737332237	0.006855512	0.173701536
ynbA	-1.995608571	1.062818128	0.060428016	0.36375562
rhaM	-1.999143995	0.634774914	0.001636203	0.086145031
csgB	-2.022357505	0.988725975	0.040813706	0.340359392

yehC	-2.023876051	1.16871729	0.083325722	0.381205511
rrfC	-2.029866737	1.277085269	0.111958206	0.400636634
casB	-2.030760451	0.852537387	0.017217998	0.263454633
higA	-2.044371482	0.71937115	0.004484739	0.144847498
hlyE	-2.046485218	0.831968691	0.013901044	0.250352735
ydfA	-2.046610575	1.582449788	0.195901146	0.483373856
ydfR	-2.046638108	0.781814752	0.008849751	0.203845977
yibG	-2.056233372	0.723825411	0.004500245	0.144847498
insA	-2.059260355	0.601269388	0.000615128	0.04653025
wcaF	-2.06167101	1.079213397	0.056088677	0.360654848
tfaQ	-2.063886225	1.413224914	0.144177731	0.428546609
ldrC	-2.064921769	1.91959581	0.282058521	0.564674195
ebgC	-2.071457953	0.78420941	0.00825495	0.192330853
insF1	-2.075828422	1.30891806	0.112759349	0.400636634
ydiT	-2.089447225	0.874841011	0.016923126	0.263454633
rrrQ	-2.096933692	0.922014279	0.022948176	0.283633854
agaB	-2.097880872	1.054843291	0.046722389	0.352374686
ydaQ	-2.109850333	1.153807099	0.067459725	0.367589495
insF1	-2.110432937	1.620084527	0.192687991	0.48030403
ydeR	-2.114721553	0.846341232	0.012466278	0.238388173
yicS	-2.120398651	0.858092645	0.013471311	0.24823952
sieB	-2.129980507	0.691111656	0.002056404	0.095180594
ydcC	-2.130216216	1.099627146	0.052718844	0.357424257
yebB	-2.131134163	0.996970849	0.032548466	0.321852891
lacA	-2.134406914	0.702764486	0.002388201	0.096817664
yegJ	-2.136272551	0.795724817	0.007259763	0.179028999
yjbT	-2.142126442	0.931388027	0.021452236	0.277602331
rem	-2.148422868	0.705547699	0.002326473	0.096240008
ybbC	-2.150722858	0.728391684	0.003150069	0.11504847
yfdP	-2.155353459	0.850845644	0.011302975	0.225664635
ynfN	-2.162758526	1.810388748	0.23222864	0.516947839
yfdT	-2.16618484	0.742071566	0.003510379	0.124834014
yiaL	-2.167542148	1.047828814	0.038583327	0.336799028
higB	-2.176061002	0.56860074	0.00012969	0.018129832
ymiB	-2.186207334	2.623162193	0.404605427	0.672067326
nrdH	-2.186705299	0.755201271	0.003785231	0.130045126
ymgA	-2.190692661	0.794926091	0.005854083	0.164265687
yibI	-2.191291887	0.713223489	0.002123609	0.095180594
yaiS	-2.197037436	1.179916539	0.062599167	0.36375562
yghG	-2.197091304	0.56675188	0.00010591	0.017256289
ydaG	-2.198982848	1.638724055	0.17963257	0.469523171
yhaI	-2.208423735	0.901212607	0.014265698	0.251448428
yahD	-2.211153731	0.710567812	0.001859426	0.091928216
eutS	-2.230930766	0.765452132	0.003562282	0.125578186
insA	-2.23910745	0.685723614	0.001093402	0.067161413
insI1	-2.258747208	0.92539579	0.014652963	0.253499259
yahA	-2.259788688	0.779316839	0.003735144	0.129421139
yigG	-2.265504175	0.892802699	0.011164118	0.225664635
sibB	-2.266045322	1.68994048	0.179952128	0.46975269

ydhL	-2.274683478	1.01560157	0.025107806	0.295116348
yghW	-2.278732365	0.810351969	0.004923023	0.153703466
ythA	-2.282202518	1.559384295	0.143322968	0.427567449
cedA	-2.285260375	1.088882022	0.035841437	0.32752865
rayT	-2.287942965	0.684403526	0.000828858	0.055085064
ariR	-2.290255924	0.945561447	0.015430585	0.257639363
pinQ	-2.296201494	0.664198602	0.000546021	0.043403288
ymgC	-2.306141316	1.040345811	0.026643155	0.299970731
yagL	-2.315678315	0.758529844	0.002266787	0.096240008
dicB	-2.316336787	1.394573084	0.096720519	0.390380788
yiaB	-2.321018805	0.778542256	0.002870867	0.111275325
ydiL	-2.330715931	0.619323493	0.000167669	0.019992026
ykfM	-2.331654854	0.896638347	0.009310411	0.208532625
ycgZ	-2.338484937	0.739062256	0.001555503	0.08417859
yadL	-2.360461136	0.953030996	0.013256852	0.247664866
yjjZ	-2.364580291	1.056461555	0.02520752	0.295116348
rttR	-2.365227335	1.382392514	0.08708729	0.383361441
ymdA	-2.365240456	0.772709774	0.002206221	0.095667434
yfcV	-2.375992282	0.908814347	0.008938776	0.204620921
ymgI	-2.376507579	0.86570034	0.00604768	0.164265687
metV	-2.376875315	1.918099837	0.215278004	0.498706873
yqeK	-2.379393385	0.847744462	0.005004652	0.153703466
yahL	-2.396042341	1.162187334	0.039239527	0.337743189
ydaT	-2.398089528	0.947919179	0.011411261	0.225664635
essQ	-2.410399163	1.051119016	0.021837969	0.278399774
ydeI	-2.411313218	0.619428983	0.0000991	0.017256289
wcaE	-2.413321016	1.135430677	0.033547622	0.324660621
yahE	-2.443716998	1.057652468	0.020859915	0.277602331
yfdS	-2.476250584	0.751615793	0.000985725	0.061478917
hiuH	-2.489907672	0.710516997	0.000457705	0.038733895
cusF	-2.492527792	0.655227143	0.00014234	0.018614372
ypjF	-2.500855788	0.809468563	0.002004915	0.095180594
yagU	-2.516569644	0.815879336	0.002039032	0.095180594
yiiF	-2.51909113	1.312836639	0.055007703	0.35965674
bdm	-2.528627466	1.10901051	0.022603223	0.283101448
yhcO	-2.529465481	0.683924342	0.000216913	0.025124746
yphA	-2.53383882	0.632996239	0.0000626	0.012681552
yiiE	-2.53426355	0.73805502	0.000595391	0.046417582
yedR	-2.55104997	0.728074384	0.000458615	0.038733895
yegR	-2.565074449	0.651561737	0.0000826	0.015940347
csgE	-2.571815016	0.737815393	0.000490822	0.040607971
fryB	-2.607451591	0.855135543	0.002294764	0.096240008
yhhH	-2.623716418	0.695442967	0.000161473	0.019836711
ysaB	-2.6260643	0.887096472	0.003073479	0.113271675
ytcA	-2.630712888	0.794479205	0.000928829	0.060733402
ryhB	-2.640690379	2.216144362	0.233430028	0.516947839
glnK	-2.642006614	0.789100931	0.00081361	0.055085064
ykgJ	-2.646866514	0.561676766	0.00000245	0.00158843
istR	-2.654796523	1.844017251	0.149957683	0.431773005

yniD	-2.663383839	2.134364869	0.212083139	0.495978403
yafO	-2.664280192	0.733750746	0.000282279	0.028608938
yafN	-2.689282389	0.73664754	0.000261522	0.02718489
ynaK	-2.697498663	0.698675598	0.000112982	0.017256289
hokE	-2.739734129	1.798511645	0.127675139	0.409405752
ykgO	-2.750015585	1.412239863	0.051502096	0.357424257
yjdJ	-2.769523727	0.727749787	0.000141462	0.018614372
tdcR	-2.779382776	1.214921475	0.022154666	0.280517603
bfd	-2.786774115	0.666721771	0.0000292	0.006957271
insC1	-2.792060622	1.137380865	0.014095676	0.250352735
paaB	-2.815856483	0.832414241	0.000717617	0.051950319
insC1	-2.827366212	1.169491025	0.015623068	0.257639363
ymgE	-2.846288495	0.650472831	0.0000121	0.003504712
ygiZ	-2.85492146	0.627726005	0.00000541	0.002150447
ydfB	-2.857550708	1.359291464	0.035532655	0.32752865
rzoR	-2.868241969	0.804915383	0.000366071	0.032978924
yagM	-2.868649294	0.944237512	0.002381073	0.096817664
ecpR	-2.869716831	0.793608908	0.000299151	0.029381351
csgC	-2.898914818	0.766458172	0.000155435	0.019691641
yehK	-2.900281287	1.087013676	0.007627735	0.182975376
yafP	-2.903810324	0.788565669	0.000231053	0.025511361
yaaI	-2.904994993	0.753233477	0.000114928	0.017256289
cspH	-2.919495338	0.937213351	0.001838945	0.091928216
insC1	-2.936807196	1.379532449	0.0332673	0.324660621
insI1	-2.943667299	1.26964621	0.020422481	0.277602331
csgF	-2.960162719	0.644478754	0.00000437	0.002150447
flxA	-2.967844369	0.581399321	0.000000331	0.000428719
yhfL	-2.970160619	1.412585668	0.035497158	0.32752865
racC	-2.975127533	0.777368753	0.000129621	0.018129832
ycgY	-3.011816004	0.676758996	0.00000857	0.002896229
yojO	-3.030154543	1.668287522	0.069320329	0.367589495
rrfE	-3.034681991	3.304335552	0.358412623	0.636177042
aspV	-3.053927278	3.135235286	0.330023582	0.61580782
insA	-3.087738181	1.327934942	0.020060308	0.277602331
dicC	-3.104999875	1.199483388	0.009636295	0.210528636
kilR	-3.142227918	0.715931184	0.0000114	0.003504712
rrfB	-3.159378532	3.17600301	0.319850282	0.607341004
yiaW	-3.162278667	0.860209426	0.000236759	0.025511361
ffs	-3.214325142	1.819667611	0.077322844	0.374512315
ypjJ	-3.235040137	0.907223177	0.000362651	0.032978924
serT	-3.283915498	3.890033539	0.398564953	0.666569049
safA	-3.324798969	1.237717251	0.007226232	0.179028999
aspU	-3.367020022	3.09540312	0.276706162	0.560426315
ryeA	-3.367996846	1.10560202	0.002316756	0.096240008
yoaJ	-3.444270711	3.029784397	0.255620274	0.535547592
yoaG	-3.446285891	1.497692059	0.021387991	0.277602331
yjbL	-3.493556491	0.892864002	0.0000912	0.016814845
yrhB	-3.522603286	0.773230977	0.00000522	0.002150447
serW	-3.529774407	3.121478694	0.25813843	0.538596601

ymgF	-3.574055511	0.989623418	0.000304395	0.029381351
gadY	-3.600024285	1.796132041	0.045035738	0.346511754
glyY	-3.630262976	2.939476723	0.216829336	0.500536144
yciG	-3.632015747	1.061123414	0.000619791	0.04653025
rrfG	-3.669349989	1.176167827	0.001810044	0.091723967
rrfD	-3.674108652	1.187187197	0.001969447	0.09504926
rrfH	-3.686310702	1.154898581	0.001413445	0.080714133
insA	-3.698509732	1.066724139	0.000525977	0.042646202
ykgL	-3.704653078	0.862422628	0.0000174	0.004707877
ralR	-3.729506715	0.921778517	0.0000521	0.011336942
ygeI	-3.747862185	1.112792887	0.000757216	0.053855315
yjbS	-3.793596756	1.191520515	0.001453412	0.080714133
ydaF	-3.809600003	1.395779116	0.006345514	0.169241531
ypdI	-3.812110868	0.753634619	0.000000423	0.000428719
alpA	-3.816631397	0.842099073	0.00000583	0.002150447
rrfA	-3.833901876	1.236967108	0.001938887	0.094701769
gltT	-3.851533043	2.718457825	0.156539043	0.441176167
ydfK	-3.898367095	1.808619566	0.031127586	0.317916139
ynfO	-3.948780138	0.589148522	2.05E-11	0.000000083
ymgJ	-3.968178937	0.933062163	0.0000211	0.005347768
yqfG	-4.047022606	1.70763909	0.017790397	0.266133831
yncH	-4.076795751	1.052075327	0.000106623	0.017256289
insC1	-4.183461067	1.303715964	0.001332556	0.079443846
rcbA	-4.255623395	0.90753681	0.00000274	0.00158843
ydfD	-4.306607566	1.227524909	0.000450867	0.038733895
insC1	-4.374399031	1.461981518	0.002770629	0.109049815
insC1	-4.512019499	1.782909314	0.011383318	0.225664635
yrhD	-4.518219462	1.663218358	0.00659658	0.170334607
rseX	-4.551385794	1.979907277	0.021517018	0.277602331
rrfF	-4.609780563	2.15466318	0.032399751	0.321852891
yqcG	-4.806654124	1.456833112	0.000968967	0.061378022
micC	-5.026996571	2.379023034	0.034596418	0.327274732
insE1	-5.227563806	1.754190111	0.002882069	0.111275325
insE1	-5.270712277	0.931851736	1.55E-08	0.0000314
serX	-5.874976586	1.845164949	0.001452628	0.080714133
yceO	-6.063878533	1.292913586	0.00000273	0.00158843

Table S2. Complete list of up- and down-regulated genes in gentamicin-resistant cells.

GENE	log2FoldChange	Standard error of the log2Fold Change	p-value	Corrected p-value
phnI	3.601392461	0.974426682	0.000219101	0.004335844
dgoA	3.498722805	0.633030569	3.26E-08	0.00000526
phnK	3.318951195	0.527732561	3.19E-10	0.000000161
phnM	3.14077839	1.004581306	0.001769275	0.018697809
phnG	3.076080688	1.097891126	0.005081703	0.037711093
phnH	2.993991426	0.923432065	0.001185927	0.014099149
fepD	2.97745597	0.747178325	0.0000675	0.001853697
fecC	2.970871394	0.617742336	0.00000152	0.0000956
phnF	2.966782972	0.887691809	0.000831376	0.011076781
yagF	2.948732817	1.098951424	0.007291614	0.048019975
rhsD	2.801896796	0.671476959	0.0000301	0.001038448
phnL	2.629673899	0.765530573	0.000592336	0.009080555
yjbG	2.58762849	0.654527702	0.000077	0.002008634
ypdE	2.576378079	0.652924169	0.0000795	0.002057277
yagH	2.565686443	1.141989005	0.024660341	0.109761626
gabD	2.565244501	0.853476682	0.002650217	0.024708836
nikC	2.545245432	0.659202719	0.000112879	0.002602194
yagI	2.502902771	0.647873238	0.000111884	0.002602194
phnC	2.472688014	0.500755724	0.00000079	0.000059
rhsB	2.472162202	0.779667928	0.001520257	0.016907103
dppC	2.45954318	0.645779541	0.000139732	0.003116564
emrD	2.451640782	0.455547048	7.38E-08	0.00000961
yagG	2.418646474	0.8184729	0.003125959	0.027613781
puuE	2.377137891	0.783007779	0.002398142	0.023161007
yhfS	2.356962651	0.440353317	8.68E-08	0.0000103
fecR	2.350449698	0.658866886	0.000360524	0.006300591
fecA	2.347405192	0.502259663	0.00000296	0.000163602
rtcA	2.347008941	0.663001122	0.000400161	0.006818218
phnN	2.343513369	0.835022139	0.005007849	0.037473068
insA	2.336429364	0.9653217	0.015505001	0.082350786
rhsA	2.334468257	0.741831488	0.001650123	0.018052976
thiF	2.323757662	0.410946126	1.56E-08	0.00000287
fepG	2.297595548	0.736593591	0.001813321	0.019013967
ynaE	2.296432689	1.617123005	0.155586435	0.365489836
fhuB	2.271555351	0.690688472	0.001006056	0.012613194
insH1	2.245011445	1.828859631	0.219617008	0.449819311
nanE	2.233071203	0.718272509	0.001877554	0.019335928
eutG	2.205538648	0.683214652	0.001245813	0.0146202
cysA	2.204156653	0.75643475	0.003569723	0.03027515
ugpC	2.204041514	0.582337414	0.000153821	0.003346475
zntA	2.196039701	0.468288236	0.00000274	0.000155719
tilS	2.186449315	0.560445574	0.0000957	0.002310516
nikD	2.171122303	0.352038584	6.95E-10	0.000000251
yagA	2.166100155	0.550992161	0.0000845	0.002145387

insH1	2.160230317	1.183385236	0.067930635	0.212750949
eutD	2.143307682	0.750611782	0.004298071	0.033691868
insH1	2.13394083	1.111033714	0.054771995	0.18581054
glcE	2.125381504	0.749806588	0.004588697	0.035217816
insH1	2.118031739	1.07298222	0.048385469	0.171343983
nanK	2.091293054	0.623320322	0.000793404	0.010820854
rsmJ	2.090086445	0.720873494	0.00373901	0.031006634
rrsD	2.086833931	0.868866149	0.016315076	0.084658051
yjhP	2.063230684	0.543037811	0.000145033	0.003209978
rrlE	2.062514512	1.77543831	0.245360132	0.47887512
rrsB	2.046707015	2.928085828	0.484557362	0.699877664
rrsE	2.042852619	1.789992081	0.253760288	0.489334145
hycE	2.029467793	0.34010068	2.41E-09	0.000000649
envC	2.017704222	0.410796225	0.000000903	0.0000628
fecB	1.997641471	0.487998007	0.0000425	0.001299268
insH1	1.991216019	0.847543847	0.018803903	0.092338281
thiE	1.989836195	0.30695489	9.02E-11	6.07E-08
fecD	1.981985727	0.52639282	0.000166401	0.003552675
eutA	1.963097754	0.746999576	0.008589431	0.054265307
insH1	1.957977877	0.801366136	0.014553634	0.079628823
rutF	1.931832482	0.525004626	0.000233556	0.004533001
dnaX	1.927766047	0.463715124	0.0000322	0.001102111
thiG	1.921310832	0.520598354	0.000223742	0.004406074
puuB	1.908351904	0.651923748	0.003419602	0.029309836
ilvA	1.899235485	0.534587192	0.000381279	0.006549878
yiaQ	1.895720671	0.785449502	0.015798014	0.08282673
rrsA	1.881848857	1.008255253	0.061979703	0.199213425
yjbE	1.880811316	0.589013325	0.00140727	0.016003236
nikE	1.874872046	0.600262152	0.001787612	0.018842271
flgF	1.871334986	0.663593203	0.004802316	0.036648301
hycB	1.870932978	0.529834724	0.000413736	0.006930509
hypF	1.848334428	0.564461877	0.001058465	0.013146698
gabT	1.842352368	0.786903208	0.019218296	0.093250315
astD	1.839465769	0.702657291	0.008848039	0.0556379
insB1	1.838222498	0.741244368	0.013141397	0.074198348
gutM	1.829165735	0.297146633	7.47E-10	0.000000251
rhsC	1.826477043	0.578891839	0.001604285	0.017599178
yagE	1.822842196	0.693945892	0.008619741	0.054336839
rrlA	1.822533457	1.057129013	0.084700494	0.247600212
ugpQ	1.815108316	0.526086857	0.000560158	0.008731108
fadA	1.811771261	0.210220619	6.79E-18	2.74E-14
napG	1.80965424	0.757232356	0.016856316	0.086686559
citX	1.804065527	0.534975339	0.000745593	0.010487662
eutK	1.788488005	0.56528564	0.001556885	0.017266878
ynjA	1.77573729	0.477937063	0.000202866	0.004094847
hycD	1.774241487	0.483018051	0.000239494	0.004582204
nfi	1.774236275	0.354308409	0.000000551	0.0000455
livG	1.768299279	0.323906094	4.78E-08	0.00000715
serB	1.765849123	0.371446592	0.00000199	0.000120177

eutT	1.750042031	0.697506539	0.012107441	0.069428605
metL	1.749724606	0.373661532	0.00000283	0.000158786
yidF	1.741368908	0.256347076	1.1E-11	1.11E-08
hflD	1.737542224	0.480296719	0.000297298	0.005406265
insH1	1.737170378	0.554651271	0.001736171	0.018464291
ulaD	1.723781578	0.429410064	0.0000596	0.001731612
fixB	1.718973572	0.516761134	0.000879638	0.011498307
yigA	1.702690225	0.320848537	0.000000112	0.0000126
entA	1.697024988	0.70062771	0.015429006	0.082269422
priC	1.693774533	0.509273625	0.000881451	0.011498307
paoB	1.69372521	0.793569587	0.032817238	0.134228154
priA	1.69348153	0.354731606	0.00000181	0.000111055
rrlD	1.691095741	1.00560111	0.09263159	0.259509875
lyxK	1.684402384	0.408151381	0.0000368	0.001197024
purE	1.683744337	0.556983184	0.002503057	0.023858946
rutD	1.6741315	0.650747654	0.010092969	0.060632909
hyaA	1.664531568	0.451347983	0.000226102	0.00443094
hyaD	1.65707741	0.661753843	0.012277402	0.070203785
phnP	1.655642332	0.597081906	0.005556093	0.040016757
araB	1.650339304	0.525036772	0.001670641	0.018228046
selB	1.637936886	0.479007451	0.000627503	0.009452355
copA	1.629963306	0.565548534	0.003950435	0.03199144
fhuA	1.628195999	0.830842823	0.050031806	0.175087444
uvrD	1.618663503	0.446187857	0.000285888	0.005270004
narJ	1.612326362	0.497343148	0.001187444	0.014099149
uhpB	1.610330787	0.461361932	0.000482325	0.007726771
yhjE	1.587742883	0.324671823	0.00000101	0.000068
hofN	1.586987338	0.411433464	0.000114686	0.002615754
nikB	1.582574454	0.574738058	0.005895136	0.041899058
nohD	1.577229757	0.927220099	0.088937242	0.255835955
ygbK	1.570558066	0.220794483	1.13E-12	2.29E-09
yehY	1.553813054	0.675734312	0.021479753	0.100479447
yhiI	1.548273827	0.386877941	0.0000628	0.00177618
mdtP	1.541297595	0.398872761	0.000111484	0.002602194
lhgO	1.531728365	0.626951762	0.014560253	0.079628823
insD1	1.522559845	0.907405043	0.09336126	0.260469527
ulaF	1.520511783	0.243885241	4.53E-10	0.000000203
rrlH	1.516342325	0.754216751	0.044379762	0.162283603
hisD	1.506648359	0.528071125	0.004329277	0.033730895
flgJ	1.505839924	0.507102364	0.002982859	0.026700222
rrsH	1.498063874	0.505791909	0.003058279	0.027209873
gldA	1.497590614	0.244026425	8.41E-10	0.000000261
fdrA	1.482304442	0.517589537	0.004185117	0.03299867
ssuD	1.478429694	0.622873825	0.017617431	0.089461093
yphD	1.478388952	0.527290331	0.005051245	0.037692928
yhbU	1.476115819	0.434577027	0.000682106	0.009842458
hscA	1.470312906	0.465207174	0.001574688	0.017416483
rrlC	1.468718259	0.49387729	0.002940863	0.026441569
thiP	1.467883766	0.527714196	0.005409357	0.039135439

rrsC	1.465925804	1.110279539	0.186727743	0.409239902
glxK	1.464292513	0.567864102	0.009920252	0.060042067
ybbJ	1.450688125	0.541385277	0.007371338	0.048308591
nrfE	1.449012709	0.493403816	0.003316517	0.028608506
chaC	1.447042131	0.266679214	5.76E-08	0.0000083
hycG	1.445888501	0.528985807	0.006269902	0.043653382
gutQ	1.441769332	0.63021687	0.022152945	0.10255899
qseB	1.440289513	0.276603712	0.000000192	0.0000194
pbpC	1.43464877	0.58137095	0.013598444	0.07592935
recR	1.434114924	0.243336644	3.78E-09	0.000000898
puuC	1.411263568	0.686546861	0.039821391	0.152089835
cysW	1.410462107	0.504292655	0.005159361	0.037993333
insD1	1.40796413	0.747436727	0.059602386	0.195401212
rrlG	1.405124918	1.275090469	0.27047012	0.507911127
insD1	1.399258311	0.736737352	0.057529848	0.191308071
bioC	1.390127702	0.508603266	0.006271727	0.043653382
paaJ	1.390096045	0.605020091	0.021584388	0.100636236
ilvB	1.387339039	0.297817859	0.00000319	0.000171577
hrpB	1.387252143	0.506058657	0.006119898	0.043116977
acpT	1.384699921	0.409685303	0.000725093	0.01034346
zipA	1.376334853	0.429501201	0.001352992	0.01542946
fliI	1.37034634	0.731830655	0.061138701	0.198174274
yfiF	1.365443181	0.342390648	0.0000666	0.001842682
eutE	1.363733873	0.779915374	0.080365986	0.238556975
ygdG	1.362694914	0.290235718	0.00000266	0.000153657
purL	1.359954229	0.441484351	0.002067136	0.020605006
purD	1.352693279	0.558306183	0.015399197	0.082269422
entS	1.351941048	0.651676168	0.03802737	0.148181942
stfR	1.347455098	0.451342727	0.002831738	0.025631676
yhdE	1.343222629	0.295326818	0.00000541	0.000266303
hemE	1.340666096	0.247907441	6.38E-08	0.00000888
ynjC	1.335102826	0.710581304	0.060259761	0.195868482
mutL	1.334843459	0.425712274	0.001715303	0.018416703
nnr	1.326380745	0.475514374	0.005281233	0.038415026
hycC	1.325216547	0.607350385	0.029112205	0.122830431
ybdH	1.322642813	0.337932943	0.0000908	0.002277003
bcsC	1.316791701	0.455131317	0.00381323	0.03135236
metR	1.314434283	0.302410122	0.0000138	0.000563993
yihV	1.311702669	0.280955627	0.00000303	0.000165345
paoD	1.30985815	0.596509182	0.028101066	0.120387107
mmuM	1.30236312	0.830863695	0.117002286	0.303169594
rimK	1.294198129	0.285143855	0.00000566	0.000275232
sdaB	1.278444977	0.218636559	4.99E-09	0.00000112
yihU	1.277232677	0.284592062	0.00000719	0.000329908
ssuB	1.273456208	0.374639727	0.000675959	0.009842458
hyaF	1.270411413	0.339307523	0.00018102	0.003747579
selA	1.27032135	0.292197697	0.0000138	0.000563993
bioF	1.266996004	0.695213043	0.068385645	0.213513417
amiB	1.266993719	0.257656459	0.000000877	0.0000628

tmcA	1.266160551	0.449340552	0.004835099	0.036792193
phnJ	1.264822754	0.559528732	0.023789344	0.107184801
kdpD	1.262300091	0.563287194	0.025029124	0.111100339
yjhG	1.26202377	0.249217238	0.000000411	0.0000368
bcsB	1.25676335	0.319427265	0.0000834	0.002130803
dnaA	1.253195423	0.405097698	0.001977683	0.020095075
ubiH	1.25226	0.376051581	0.000868392	0.011498307
cueR	1.248432076	0.271761331	0.00000435	0.000222358
ybgL	1.239228252	0.6479335	0.05580113	0.18788087
uhpT	1.238167822	0.233390828	0.000000113	0.0000126
insG	1.235562324	0.316277225	0.0000936	0.00228814
ypdF	1.230297238	0.483741887	0.010981318	0.064341916
glgA	1.229388377	0.212371229	7.09E-09	0.00000143
cysJ	1.223743417	0.469560202	0.009156687	0.056870067
yggW	1.221921961	0.218379629	0.000000022	0.00000386
glnG	1.221444116	0.345773081	0.000411647	0.006930509
nrfD	1.221335803	0.433476865	0.004839399	0.036792193
fhuC	1.220997627	0.450978346	0.006780396	0.046470667
dppF	1.218182371	0.247983605	0.0000009	0.0000628
argF	1.217603811	0.516292031	0.018355754	0.091710618
acs	1.21510331	0.428332999	0.004556539	0.035037619
nepI	1.214402846	0.354796262	0.000619757	0.00937063
ybbP	1.213081203	0.448145129	0.006791601	0.046470667
yihT	1.209138567	0.31723421	0.000138125	0.003097846
yjcE	1.198142984	0.354092629	0.000715163	0.010237986
dedD	1.195192593	0.508385896	0.018725276	0.092338281
ppc	1.191588286	0.208865367	1.16E-08	0.00000224
murP	1.190148536	0.627027914	0.057685672	0.191668359
yecS	1.188872364	0.298852256	0.0000695	0.00188195
kdpE	1.184993479	0.522212672	0.023257507	0.105900142
ftsN	1.183647105	0.254474604	0.0000033	0.000175179
amtB	1.181334438	0.448853886	0.008491114	0.05389721
kefF	1.179248147	0.436425612	0.006891135	0.046598845
carB	1.17780927	0.36264408	0.001162854	0.014097423
livH	1.176306147	0.310364035	0.000150596	0.0033041
yjgB	1.170924303	0.229404213	0.000000332	0.0000312
putA	1.170586599	0.533152997	0.02812114	0.120387107
recG	1.16811281	0.459208038	0.010966793	0.064341916
argH	1.167270563	0.407191484	0.004148573	0.032774538
mutS	1.165798835	0.319247481	0.000260501	0.004891364
leuC	1.165156877	0.478530911	0.014897457	0.080722588
argB	1.16272315	0.351316246	0.000934228	0.011945893
ygiF	1.16195894	0.340796753	0.000650734	0.009658135
recQ	1.160499162	0.374142015	0.001923731	0.019661019
sapC	1.158798199	0.647303578	0.073422923	0.223199053
glcF	1.154149272	0.528917203	0.029102287	0.122830431
bioA	1.153952791	0.502807643	0.02173207	0.100957844
mdtN	1.153906851	0.538399696	0.032095757	0.131811364
kbaZ	1.153337737	0.335850126	0.000594559	0.009080555

entF	1.145527324	0.523372286	0.028615539	0.12160098
yhaM	1.144087395	0.44939676	0.010901851	0.064341916
glnE	1.143618771	0.409811317	0.005261072	0.03833745
ilvI	1.142801519	0.212775036	7.83E-08	0.00000961
mdtB	1.12882044	0.585710124	0.053946226	0.183781364
hda	1.1171936	0.361591564	0.002003883	0.020173759
mokC	1.116960077	2.351766891	0.63482613	0.810278348
yhhS	1.105941359	0.501541375	0.027448131	0.118765385
yqjH	1.104420753	0.354589497	0.001841658	0.019112528
recD	1.099599112	0.458881084	0.016563265	0.085520737
yihQ	1.096645698	0.203332009	6.91E-08	0.0000093
uhpA	1.091568805	0.232412716	0.00000264	0.000153657
fabB	1.079473971	0.40692268	0.007983327	0.051401418
yiaD	1.078381459	0.253776931	0.0000214	0.000794114
glcC	1.075110937	0.214606817	0.000000545	0.0000455
qorB	1.072185357	0.412003256	0.009258162	0.057236139
tauC	1.07158409	0.670800597	0.110160893	0.29123741
eutC	1.071470599	0.421093602	0.010943585	0.064341916
rrsG	1.068818961	0.758361549	0.158723084	0.369343025
yehM	1.068218431	0.516497682	0.038621598	0.148963176
yihP	1.066233293	0.332441848	0.001339976	0.015324318
metE	1.065685921	0.272676897	0.000093	0.00228814
scpA	1.065585089	0.359456677	0.003032417	0.027083775
hofM	1.064220636	0.514916261	0.038754522	0.14914395
norW	1.063216533	0.471643873	0.024178668	0.107968693
nrfC	1.06287309	0.42257648	0.011895791	0.068604724
aegA	1.060614031	0.491261236	0.030853164	0.128010506
moeA	1.054083662	0.382094126	0.00580315	0.041597702
kdpB	1.048730462	0.548283701	0.055779875	0.18788087
ampH	1.045056408	0.230673211	0.00000589	0.000281845
paoC	1.041675588	0.677670134	0.124258409	0.313911887
ssb	1.035048599	0.308782063	0.000802196	0.01090392
lplT	1.031213247	0.305502296	0.000736902	0.010439255
cstA	1.03011983	0.511418717	0.043984384	0.161655802
yraQ	1.029986542	0.415213165	0.01311537	0.074155111
fadH	1.029216598	0.546559387	0.059689144	0.195401212
yaiC	1.027933827	0.256700505	0.0000622	0.00177618
hypE	1.026211986	0.331820301	0.00198362	0.020095075
mrcA	1.019987562	0.23511321	0.0000144	0.000579715
insK	1.019742744	0.320366646	0.001457329	0.016433627
yeiE	1.019021455	0.340704035	0.002781305	0.025398642
paaD	1.01746924	0.336850248	0.002523254	0.023858946
leuB	1.016244669	0.270617802	0.00017315	0.0036218
yiaN	1.01620988	0.384016613	0.008138593	0.05206894
ygcN	1.010792312	0.326952388	0.00199109	0.020095075
frvB	1.010322448	0.44181366	0.02220975	0.102704195
yigZ	1.007835153	0.388526457	0.009486825	0.058381571
gspH	1.007780822	0.431344453	0.019471896	0.094254249
kefC	1.007385427	0.443998528	0.023274796	0.105900142

yhgF	1.006247009	0.443951232	0.023416442	0.106335408
flu	1.006096326	0.347373673	0.003775983	0.031109478
hyfB	1.000921012	0.542310655	0.064941608	0.206435222
proW	0.999449426	0.548073361	0.068217302	0.213152669
argI	0.997091937	0.216136723	0.00000396	0.000205177
rhaB	0.996253072	0.243068849	0.0000416	0.001280668
napA	0.994840317	0.47354188	0.035654162	0.141669146
eutJ	0.994775537	0.528789013	0.05994018	0.195851341
ycjO	0.99394457	0.4409027	0.02417479	0.107968693
panB	0.993391806	0.225388655	0.0000105	0.000449177
hldE	0.993232057	0.219960524	0.00000632	0.000296518
mak	0.988971591	0.416944462	0.017694465	0.089739392
ylbF	0.988249959	0.430481495	0.021693892	0.100896594
pepB	0.987035286	0.208180732	0.00000212	0.000126104
gph	0.981606732	0.272195004	0.000310638	0.005598427
panM	0.981141942	0.48410411	0.042691381	0.159578802
yajL	0.971883416	0.50742057	0.055449031	0.187320284
guaC	0.970145926	0.236511712	0.000041	0.00127549
hyfD	0.968376925	0.493584488	0.049770939	0.174414305
livF	0.967737333	0.34435102	0.004949192	0.037253735
gfcA	0.967713973	0.472157495	0.040407624	0.153402862
ubiF	0.967560515	0.390495248	0.013220339	0.074435857
dcd	0.96647296	0.354560069	0.006413866	0.044185627
ycjQ	0.96588064	0.591093982	0.102247102	0.276900479
yggF	0.965829928	0.357316399	0.00687146	0.046598845
glcD	0.961240615	0.519131908	0.064078861	0.204818973
ribD	0.960524321	0.234196736	0.0000411	0.00127549
envZ	0.960205216	0.326567434	0.003278979	0.028467177
yiaS	0.959139274	0.261668457	0.00024688	0.004690825
tsaD	0.95850827	0.403445484	0.017510659	0.089255719
dgoK	0.95678515	0.380511858	0.011921189	0.068653124
ptsA	0.953626617	0.441925919	0.030936544	0.12822467
glpG	0.952955481	0.214589644	0.00000896	0.000393229
insD1	0.950372877	1.129924026	0.400294999	0.631204301
norR	0.949574401	0.482080232	0.048867775	0.172145907
yahG	0.949243046	0.764726235	0.21450056	0.443099451
nrdI	0.947256621	0.351817545	0.007092586	0.047326893
tar	0.945795115	0.531408905	0.075110686	0.227302728
mdlB	0.944360141	0.312215648	0.00248876	0.023808349
ulaE	0.943014036	0.487681657	0.053154056	0.182313445
ada	0.941566302	0.601891716	0.117736567	0.304485921
radA	0.940090047	0.55776004	0.091897157	0.258953448
ypfG	0.940061403	0.40923809	0.021612984	0.100636236
metH	0.939149928	0.267396733	0.000444419	0.00726364
emrA	0.938686795	0.236734965	0.0000734	0.001944517
lnt	0.937370995	0.263141366	0.000367713	0.006398524
lldD	0.937061078	0.213623765	0.0000115	0.000489489
insD1	0.931819025	1.083817041	0.38992314	0.622427726
acuI	0.930367644	0.318937154	0.003533164	0.030091528

folB	0.92980049	0.279479613	0.000878181	0.011498307
ygjK	0.925949477	0.37619611	0.01384161	0.076923605
insD1	0.92371172	1.065158001	0.385828989	0.620060362
rihC	0.923613305	0.283048648	0.001102044	0.013563876
xylB	0.922050958	0.210894314	0.0000123	0.000513782
cysS	0.921310043	0.220443497	0.0000292	0.001024928
wecD	0.918430752	0.311151413	0.00316013	0.027854679
glnL	0.916374826	0.234402471	0.0000925	0.00228814
glcB	0.915284563	0.369750359	0.013308247	0.0746186
fepB	0.913695067	0.395573718	0.020899384	0.098300179
purH	0.912933106	0.287680526	0.001506509	0.016847022
gpsA	0.912708409	0.289223375	0.001601017	0.017599178
citE	0.91177382	0.496720885	0.066418934	0.20947909
insL1	0.911720053	1.150572957	0.428124419	0.651957102
cysE	0.911114337	0.332886444	0.006199995	0.043378474
hisA	0.907506752	0.624705839	0.146308587	0.350740954
ytfT	0.907077762	0.621404147	0.14436629	0.348152159
insL1	0.904644411	1.131541477	0.424012328	0.649369412
ade	0.903517156	0.248022253	0.000269597	0.005015493
rbbA	0.90202402	0.523522675	0.084890442	0.247717609
garL	0.898718928	0.252728786	0.000376457	0.006522562
yacH	0.896234976	0.210110729	0.0000199	0.000766483
basS	0.893508684	0.249654348	0.000344934	0.006080776
rhlE	0.892665197	0.248613942	0.000329958	0.005893993
yjbB	0.892566531	0.307860232	0.003740458	0.031006634
flgH	0.892337272	0.352479643	0.011354361	0.066048353
visC	0.89159139	0.253448752	0.00043508	0.007169046
wcaI	0.89047506	0.763491318	0.243485161	0.476466115
entC	0.88952241	0.417315778	0.033045245	0.134479491
uvrA	0.888240546	0.288085664	0.002047521	0.020510771
gor	0.887375648	0.217449388	0.0000449	0.001362068
ggt	0.885799855	0.397688474	0.025922476	0.11376265
yaaJ	0.876944159	0.392576483	0.025495093	0.112607977
yggR	0.875745117	0.384580257	0.022777551	0.104610893
murF	0.873654224	0.217109743	0.0000572	0.001685897
yfjR	0.873519825	0.265548557	0.001003684	0.012613194
lldR	0.870911813	0.225632393	0.000113447	0.002602194
yidR	0.870614085	0.248421531	0.000457322	0.007414486
fdhD	0.865808285	0.322620037	0.007281721	0.048019975
purK	0.864884986	0.386731513	0.025325813	0.111982811
recF	0.861236255	0.296276667	0.003650708	0.03080262
ilvD	0.858632967	0.329547259	0.009174224	0.056891461
ilvY	0.857688801	0.433583139	0.047912746	0.169818925
cynX	0.857044157	0.638911736	0.179786577	0.399854937
dinF	0.85667037	0.391017683	0.028460931	0.121558519
recB	0.855902359	0.416941682	0.040090937	0.15254205
yphG	0.854375275	0.465089919	0.066208278	0.209141487
glyS	0.853855887	0.222420478	0.000123573	0.002802616
cbeA	0.851511064	0.315636639	0.00698083	0.046891201

ilvE	0.851276249	0.379022948	0.024705578	0.109841872
bdcA	0.849829594	0.253860716	0.000815082	0.010968293
tauA	0.848868461	0.279392399	0.002379437	0.023090837
kdpC	0.847152458	0.446673885	0.057883481	0.191882081
phnO	0.846506499	0.600585701	0.158696745	0.369343025
adiC	0.842531579	0.242285468	0.000506239	0.007983148
cdaR	0.841038753	0.261626493	0.001306017	0.015107132
insL1	0.840983581	0.991657129	0.39640521	0.628375685
ydiK	0.840140978	0.243658857	0.000564709	0.0087346
katG	0.837924069	0.212715359	0.0000818	0.002102341
uhpC	0.837422861	0.405813583	0.039058943	0.149744494
alr	0.835903454	0.279218893	0.002755979	0.02538587
livJ	0.834730595	0.242316457	0.000571492	0.008805775
folD	0.832788703	0.477296826	0.081019251	0.240137904
araD	0.827851106	0.399599409	0.038293191	0.148358554
yiaR	0.827633966	0.56854641	0.145475104	0.349573212
cusB	0.826337363	0.469618956	0.078477151	0.234502043
malK	0.825594174	0.212672457	0.000103601	0.002474788
frlB	0.822736484	0.367464678	0.025158914	0.111489064
rhaA	0.822543876	0.373536674	0.02766226	0.119308274
lldP	0.822432469	0.243835036	0.000743795	0.010487662
yphB	0.822309292	0.417373813	0.048815617	0.172112353
ebgA	0.821906646	0.307731037	0.00756571	0.049421961
fdhF	0.820591574	0.204353635	0.0000593	0.001731612
insF1	0.816163588	1.14500046	0.475965601	0.693434122
cheR	0.8152102	0.481239543	0.090269068	0.257902497
ybiW	0.814794614	0.48491767	0.092903993	0.259732285
btuC	0.813422168	0.532362506	0.126525476	0.318348837
mreC	0.81029793	0.278911994	0.003670067	0.03080262
gadA	0.810070391	0.505314331	0.108912147	0.290025288
yjbH	0.807133432	0.299113638	0.006967003	0.046876322
ycjX	0.806889223	0.515349337	0.117415791	0.30399856
thrB	0.805531098	0.31897168	0.011556665	0.066935804
kbl	0.803703019	0.239544031	0.000793257	0.010820854
rtcR	0.803037629	0.511275874	0.11626298	0.302418589
rlpA	0.802865381	0.313418566	0.010417939	0.062280087
ybgJ	0.801953438	0.516832457	0.120741195	0.309308482
cueO	0.798105114	0.266755295	0.002772472	0.02538587
moeB	0.796667663	0.395146105	0.043785922	0.161575654
mog	0.795138314	0.332890083	0.016913111	0.086867976
xylF	0.793981751	0.404035261	0.049399211	0.173448284
yjjW	0.793173834	0.403041851	0.049071615	0.172562812
patA	0.792804298	0.396667098	0.045644707	0.164880193
frlD	0.787618742	0.380691378	0.038553906	0.148963176
livM	0.786603211	0.433245001	0.069430483	0.215277157
argK	0.785827605	0.279137904	0.004874766	0.03695039
yjhH	0.785804691	0.324167409	0.015347621	0.082269422
nfrB	0.784524878	0.410168476	0.055787833	0.18788087
ybhP	0.783060227	0.402907417	0.051952881	0.179259642

kdpA	0.782371857	0.4622042	0.090513076	0.258179976
holC	0.781918468	0.290879876	0.007185671	0.047790039
wecE	0.778641977	0.351576254	0.026779481	0.116496515
fryA	0.776760067	0.565730735	0.169745881	0.384904479
yfcJ	0.775814753	0.48217017	0.107615004	0.287139306
dppB	0.775066721	0.22050816	0.0004399	0.007219012
aroC	0.775054234	0.588980092	0.188198871	0.410760621
tdcE	0.773126549	0.232192433	0.000869458	0.011498307
fic	0.771019916	0.237743314	0.001182462	0.014099149
hyaB	0.768590186	0.547163427	0.160116095	0.371274368
mdtC	0.768171072	0.664156125	0.247430838	0.481726239
ycjT	0.765107877	0.581969059	0.188614701	0.410778023
yfgC	0.764803872	0.287929112	0.007902139	0.051041494
glcA	0.764723986	0.352919733	0.030246323	0.126271361
rsxC	0.764028289	0.643971281	0.235451646	0.467572833
wcaG	0.762258692	0.511188516	0.13592215	0.333567003
yjbF	0.759874818	0.343031274	0.026747952	0.116484879
cysI	0.759758618	0.532611517	0.15373041	0.363399154
phnD	0.759302478	0.459345574	0.098328983	0.271328847
lptG	0.757559589	0.329216461	0.02138605	0.100157173
ygiD	0.755342492	0.257023019	0.003294722	0.028481355
srlE	0.754498688	0.26995646	0.005191733	0.038038161
malZ	0.75361998	0.348043343	0.030364338	0.126567523
yfaQ	0.752580271	0.336473594	0.02530794	0.111982811
hemH	0.748540476	0.219956634	0.000666192	0.009779695
ybiC	0.745477715	0.492776779	0.130327889	0.323774577
bcsG	0.745266608	0.289371862	0.01001059	0.0604077
qorA	0.743764239	0.230476804	0.001250659	0.014634521
eutL	0.741956338	0.435916126	0.088743718	0.255716197
paaG	0.740489364	0.363790679	0.041802402	0.157716167
frlC	0.740462068	0.260652331	0.004500002	0.034735199
insF1	0.739534658	0.977919181	0.449509571	0.669619977
rlmB	0.738271722	0.278198158	0.007959993	0.051333053
cheB	0.738063519	0.566203046	0.192393362	0.413574016
sufA	0.737412129	0.370095539	0.046317182	0.165911681
trpD	0.737106414	0.57643807	0.20099352	0.424378054
prpD	0.735470328	0.694379874	0.289519744	0.5288648
glpX	0.734192907	0.257377769	0.004336471	0.033730895
mscM	0.733343878	0.491122329	0.135384834	0.332848571
aidB	0.731873949	0.32211154	0.023079661	0.105518226
hemC	0.731244414	0.218779912	0.000830649	0.011076781
lhr	0.730125392	0.583844714	0.211099712	0.437969394
xylH	0.727583581	0.247847241	0.003328894	0.028654036
cusA	0.726879636	0.493550621	0.140816842	0.342456381
ccmF	0.72549488	0.528742422	0.170027952	0.384904479
fsr	0.723780709	0.374865119	0.053510716	0.18322541
lplA	0.720968785	0.587852645	0.220031358	0.45011601
malQ	0.720783111	0.250966538	0.004078459	0.032628631
galE	0.720619776	0.213489192	0.00073698	0.010439255

ygcR	0.717321702	0.457626087	0.117001535	0.303169594
adiA	0.71380818	0.220169113	0.001186564	0.014099149
glrR	0.712002682	0.513414251	0.165502877	0.378709686
tesA	0.711292355	0.233487484	0.002316124	0.022639694
yhhQ	0.71126117	0.226702563	0.001704417	0.018348621
gsiA	0.711209867	0.423153541	0.092813985	0.259660471
wcaH	0.710657969	0.674710296	0.292213287	0.53151325
ynjB	0.705220986	0.599455617	0.239420815	0.471496507
cca	0.699915252	0.212754922	0.001002685	0.012613194
mdtF	0.698975943	0.233415402	0.002748429	0.02538587
tamB	0.698891454	0.262159907	0.007678309	0.04991519
rhaD	0.698304014	0.248840669	0.005012499	0.037473068
rapA	0.698151658	0.280448316	0.012795426	0.072753713
ilvC	0.696949041	0.244796308	0.004412546	0.03419088
ycjF	0.696050501	0.584023481	0.233332077	0.465856377
eutQ	0.695617018	0.613045549	0.25650498	0.492631115
trpB	0.695404495	0.42635505	0.102880714	0.277812336
yheT	0.69526188	0.343874909	0.043192333	0.160264199
norV	0.69526023	0.360063549	0.053490775	0.18322541
yahF	0.693999337	0.637896298	0.27661714	0.51382911
yjcD	0.693665713	0.222591899	0.001831287	0.019053877
fliA	0.69345312	0.515783087	0.178797558	0.399008148
bglX	0.693325868	0.286918353	0.015672377	0.082489419
nirB	0.692685725	0.229451214	0.002537108	0.023874841
tldD	0.689452525	0.23279387	0.003060015	0.027209873
selU	0.689375606	0.252304522	0.006289085	0.043698855
phr	0.688949233	0.285968783	0.01598857	0.08350046
ugpA	0.688921176	0.292909533	0.018673125	0.092338281
yjcZ	0.688895622	0.298005805	0.020795035	0.098185682
insH1	0.688798777	0.642096246	0.283390269	0.520968359
recJ	0.686158415	0.324148367	0.034276524	0.138374326
ytfF	0.685284837	0.303923122	0.024146095	0.107968693
pdxJ	0.685148829	0.279135696	0.014106624	0.07801936
rnhB	0.684303844	0.646742083	0.29001969	0.529167081
aslA	0.682810492	0.254180343	0.007224322	0.047889307
yicH	0.681579658	0.28169135	0.015537699	0.082350786
pncC	0.681524652	0.365098562	0.061945344	0.199213425
citG	0.681507256	0.335387145	0.042153482	0.158448422
gltS	0.681350609	0.225462414	0.002510988	0.023858946
yhbW	0.680948359	0.305331981	0.025734826	0.113301556
yiiD	0.679796829	0.32890039	0.038745695	0.14914395
hcaT	0.678618248	0.503096342	0.177374457	0.396793952
yjjV	0.677530766	0.376214034	0.071715747	0.219830272
bcsZ	0.676334343	0.447294181	0.130519336	0.324050775
pflD	0.675710331	0.339619861	0.046634154	0.166898617
hcaB	0.673897566	0.524416816	0.198777653	0.42212803
betB	0.673723743	0.765021351	0.378501845	0.613112592
ftsP	0.67208301	0.244748889	0.006032484	0.042649979
feoB	0.67067808	0.369944238	0.069844673	0.21606356

dgoR	0.66793681	0.260581371	0.01036955	0.062109606
nfrA	0.667272394	0.481434349	0.165744121	0.378883928
nanT	0.666306683	0.455679207	0.14367887	0.34690885
fiu	0.665686046	0.360753645	0.064999011	0.206435222
hyfH	0.664072608	0.348457092	0.056682461	0.189898004
ycjR	0.662503371	0.52540236	0.207328738	0.433446977
ttdA	0.65986949	0.269637465	0.014395213	0.079281686
lpoA	0.659543698	0.388060914	0.089208436	0.255983572
ybgK	0.6564695	0.326139145	0.044130308	0.16181113
mdtA	0.655323688	0.48917979	0.180363162	0.400413305
modB	0.653696156	0.496414323	0.187893668	0.410760621
yicI	0.653214298	0.217703531	0.002695577	0.025016192
gyrB	0.649771366	0.252275783	0.010005502	0.0604077
ybjJ	0.648923966	0.412417724	0.115611685	0.301112498
betI	0.648910617	0.438771271	0.139159864	0.339449168
nanC	0.648672911	0.265465293	0.014544284	0.079628823
malT	0.647070256	0.247431839	0.008918946	0.055909602
folC	0.64636839	0.49407172	0.190789172	0.411983443
torA	0.645645231	0.508133883	0.203863759	0.427753635
hemF	0.645188938	0.386995472	0.095479735	0.265097448
ydhB	0.643150198	0.419818971	0.125529762	0.316727281
mdtD	0.64222128	0.46515922	0.167387128	0.381559479
ypjK	0.637625187	0.369108216	0.084082405	0.246686533
ybcF	0.637070644	0.377055815	0.091105958	0.258953448
bcsA	0.635107468	0.268985184	0.01821962	0.091523534
yjcS	0.634767294	0.377401019	0.092579743	0.259509875
kefB	0.633525205	0.325579027	0.051673773	0.178754947
hypD	0.62994384	0.246791826	0.010694332	0.06358324
dppD	0.629271409	0.306998823	0.040388651	0.153402862
dadX	0.628745779	0.543167265	0.247045836	0.481333995
yaaU	0.628054552	0.331787018	0.058365194	0.192282462
php	0.627270928	0.247597731	0.011295307	0.06589473
fabA	0.626535768	0.416808745	0.132794366	0.327884315
nuoL	0.623483078	0.251479249	0.013165437	0.074230263
recO	0.62332463	0.27985354	0.025925598	0.11376265
entE	0.623040996	0.51409223	0.225541263	0.456624913
plsB	0.622947463	0.239943486	0.009425411	0.058092189
ytfE	0.622712633	0.233689303	0.00770563	0.050012263
arsB	0.622562173	0.39000731	0.110425547	0.29174603
ascF	0.622117553	0.43112885	0.149021371	0.355920987
nirC	0.617457365	0.326278423	0.05843441	0.192282462
gntU	0.617175317	0.3972022	0.120230605	0.308563861
insB1	0.617014533	1.299239751	0.634855193	0.810278348
frwC	0.616585091	0.255543838	0.015828988	0.082881485
treF	0.615043169	0.296531087	0.038067506	0.148195297
atoD	0.613769525	0.248454778	0.013498203	0.07550954
hyfG	0.612621836	0.397550659	0.12331907	0.313500683
hyfF	0.609324456	0.418485929	0.145386712	0.349573212
thiQ	0.608160399	0.539482373	0.259614158	0.496241647

yhbV	0.6081451	0.310649533	0.050270324	0.175472242
yedZ	0.608003786	0.735997079	0.408749989	0.637359484
gltJ	0.607635234	0.234767219	0.009646616	0.059094671
dapF	0.606620314	0.258101708	0.01875753	0.092338281
yhjK	0.604854418	0.205017228	0.003175052	0.027925236
artQ	0.602777978	0.344444598	0.080118348	0.238162499
yjfC	0.601334877	0.222450507	0.006866922	0.046598845
ccmC	0.60095921	0.289465332	0.037884628	0.147960422
xerC	0.600916948	0.232784668	0.009839241	0.059710616
sbcD	0.59952441	0.302617017	0.047576416	0.169370362
yjjP	0.598805604	0.217656128	0.005938565	0.042059629
wzxE	0.598138481	0.283334432	0.034766398	0.139403565
yigB	0.597776987	0.480029315	0.21302419	0.440788649
yicO	0.596203952	0.20774047	0.004105418	0.03268949
pepA	0.595960898	0.319148012	0.061852789	0.199213425
alsK	0.594452579	0.301570736	0.04870239	0.172013602
tdcG	0.593661616	0.219471433	0.006831246	0.046515543
tap	0.593606229	0.491462777	0.227110512	0.458207332
yjdL	0.592413902	0.21923376	0.00688819	0.046598845
ybbN	0.592248712	0.30792158	0.054432954	0.184971243
kdgK	0.591675404	0.285373643	0.038141035	0.148195724
ygfS	0.591312738	0.254804008	0.020305356	0.09712408
sgrR	0.590303434	0.357960997	0.099132898	0.272244565
yhjG	0.590204645	0.33917909	0.08184192	0.241694098
ileS	0.589740854	0.218469485	0.006946049	0.046813359
abrB	0.588454972	0.407865991	0.149086547	0.355920987
fixC	0.588229598	0.358832721	0.101153628	0.276103582
mnmE	0.587741974	0.260073235	0.023826868	0.107234188
narW	0.586829804	0.366764214	0.109594313	0.291073842
damX	0.586695526	0.24995401	0.018914393	0.092369789
yhgE	0.586256966	0.231330162	0.011267648	0.065828503
gntT	0.586129662	0.248759989	0.018462699	0.091903719
puuA	0.582804916	0.363651114	0.109012277	0.290100569
gcd	0.582341624	0.289084768	0.043964192	0.161655802
potG	0.582291941	0.409589847	0.155128921	0.365376577
dsbD	0.581451295	0.277011355	0.035815481	0.142030547
tktB	0.581438955	0.412010485	0.158178668	0.369327521
mdtO	0.581220794	0.387426692	0.133560417	0.329328554
ybaE	0.580618535	0.305252738	0.057159237	0.190726867
amiD	0.579264887	0.635770806	0.362230998	0.598823316
yhiN	0.578655504	0.215827615	0.00733805	0.048168628
yidG	0.57853696	0.256403573	0.024048535	0.107871042
mdtE	0.578507356	0.228630972	0.011396162	0.066196122
bcr	0.577522532	0.368191857	0.116755874	0.303169594
fadB	0.577439384	0.319543749	0.070750652	0.217532661
rutA	0.576178604	0.399983222	0.14972403	0.357231625
yjeH	0.575227251	0.241599683	0.017269902	0.088475372
yihS	0.573949975	0.235612246	0.014850938	0.080690764
hcp	0.573052745	0.417965351	0.170358363	0.384904479

mreB	0.571077022	0.512649814	0.265291631	0.502808599
yraN	0.570771829	0.380224916	0.133318676	0.328977686
poxB	0.570275097	0.424476118	0.179116657	0.399278821
purP	0.56938669	0.265423853	0.031937028	0.131561002
yhfW	0.567589257	0.248338574	0.022280827	0.102797371
ybiS	0.56732075	0.477975085	0.235257063	0.467572833
leuA	0.567172695	0.313338842	0.070281243	0.216559535
fumB	0.566466708	0.281572319	0.044241205	0.162070548
torS	0.564175439	0.403975639	0.162546506	0.374436109
apaH	0.562962012	0.321752001	0.080174098	0.238162499
rtcB	0.562522764	0.220176696	0.010622756	0.063250835
mutY	0.562346233	0.364341304	0.122719203	0.31217229
dusC	0.561171395	0.400400977	0.161057574	0.37260139
panF	0.560349649	0.366773356	0.126566728	0.318348837
yibQ	0.559676289	0.245541237	0.022645816	0.104124325
sgcX	0.55800364	0.322715775	0.083793531	0.24619686
mdtL	0.557117843	0.253667939	0.028074158	0.120387107
ykfG	0.556852363	0.563307826	0.322888216	0.562426756
yihM	0.556750028	0.382018797	0.145009006	0.34928482
yedD	0.556587972	0.358859571	0.120903836	0.309308482
aceF	0.555776238	0.290778321	0.055960901	0.187953471
entB	0.555305006	0.377929748	0.141742235	0.344292059
yiaY	0.553151965	0.276629707	0.045542222	0.164880193
hsrA	0.552676735	0.252383949	0.028536916	0.12160098
yhbS	0.55265129	0.307250675	0.072066407	0.220236248
lpxK	0.552211215	0.407182718	0.175043344	0.393238721
ubiA	0.550117286	0.220045881	0.012418919	0.070812396
ugpE	0.550029868	0.33302332	0.098610881	0.271356895
yafV	0.549369362	0.360551395	0.127585609	0.31982398
insI1	0.549149435	0.856621232	0.521480798	0.730978466
ygjJ	0.547449326	0.225391654	0.015145536	0.081523371
kdsC	0.546636441	0.257013569	0.033430242	0.135636068
yjfP	0.543644565	0.248374226	0.028610484	0.12160098
yidK	0.540529603	0.276713219	0.050773072	0.176404667
aer	0.540456564	0.278594654	0.052387135	0.180318709
metB	0.538548143	0.341774282	0.115085238	0.300322628
eptA	0.536646696	0.265057262	0.042903894	0.159762217
ybdL	0.535908707	0.348524901	0.124134641	0.313911887
aslB	0.534417554	0.214634169	0.012777915	0.072753713
waaA	0.534199975	0.214729642	0.012854345	0.072907843
ftsE	0.533662475	0.405725672	0.188399577	0.410760621
mrcB	0.533262532	0.209648436	0.010971549	0.064341916
xanP	0.532006138	0.24945704	0.032952928	0.134467273
yidJ	0.531429561	0.253847925	0.03630492	0.143207765
nrfB	0.531381244	0.302519348	0.078999683	0.235366582
aldB	0.531083699	0.346951897	0.125840397	0.317312731
yhiD	0.529486096	0.294860042	0.072539048	0.22117835
yhhJ	0.529126305	0.248188952	0.033011001	0.13447569
glyQ	0.526992096	0.343244325	0.124703657	0.314839689

mhpB	0.526240813	0.607019754	0.385982912	0.62006089
ykfA	0.52576139	0.263266652	0.045817804	0.164880193
gspO	0.525035667	0.215197705	0.014696094	0.080064952
fliG	0.524214765	0.667383673	0.432173739	0.654911932
tsaB	0.524206437	0.338475444	0.121447092	0.310108734
lamB	0.522214418	0.316169997	0.098598023	0.271356895
ligA	0.521022929	0.374549287	0.164205292	0.376645888
ygiS	0.520508726	0.270732944	0.054531433	0.185150037
fruB	0.51896074	0.310817952	0.094986373	0.264272906
rph	0.518913305	0.36807091	0.158593181	0.369343025
yojI	0.517547553	0.398097669	0.193583248	0.415061256
hemY	0.517352469	0.261218934	0.047643776	0.169411425
lpxB	0.516259987	0.43205752	0.232131117	0.464796105
fsaB	0.516012158	0.248565001	0.03789722	0.147960422
hofB	0.515170586	0.212826744	0.015494482	0.082350786
araA	0.514310157	0.350861601	0.142689405	0.345760581
xylR	0.513183703	0.218240912	0.018700147	0.092338281
yjjK	0.511937282	0.211339954	0.015420903	0.082269422
cysN	0.510588454	0.420327508	0.224465437	0.455818395
murC	0.510517303	0.297483743	0.086140388	0.249999098
sugE	0.509681751	0.633319521	0.420947677	0.646392459
ulaA	0.509551017	0.212121231	0.016298183	0.084658051
cadB	0.509265085	0.315156286	0.106113162	0.283882594
thiH	0.506889653	0.240210783	0.034842258	0.139403565
yrfF	0.506175617	0.299922707	0.091471352	0.258953448
argD	0.504392132	0.283113367	0.074816008	0.226750918
prpE	0.50289428	0.725266546	0.488063357	0.7011811
astC	0.502439469	0.600411445	0.402690791	0.632799815
yijO	0.501388718	0.359935785	0.163621329	0.375733394
polB	0.501053415	0.428917749	0.242733904	0.475456948
wcaJ	0.500050009	0.494999826	0.312398332	0.551950915
bglF	0.499801993	0.212929947	0.018912042	0.092369789
ybgI	0.499779897	0.314448741	0.111973654	0.295445386
citF	0.497439241	0.487088382	0.307135784	0.546696279
cysU	0.495848024	0.608797505	0.415375074	0.642478611
fadE	0.495319102	0.322160611	0.12417307	0.313911887
cheZ	0.49522753	0.3504019	0.157563739	0.368317784
emrB	0.494817072	0.405373291	0.222220281	0.453082463
rne	0.493264698	0.212547986	0.020301876	0.09712408
sgcC	0.493165809	0.252505243	0.05080877	0.176404667
glpD	0.491656909	0.239101735	0.039756827	0.151987038
acrD	0.49149109	0.366407639	0.179797806	0.399854937
ypjA	0.491340532	0.224301108	0.028485102	0.121558519
trmJ	0.490809617	0.236011845	0.037562477	0.147080232
insH1	0.490756115	1.103505562	0.65651876	0.822328959
xylA	0.490555656	0.207920695	0.018307463	0.091582689
hemN	0.490350726	0.353532077	0.165440266	0.378709686
mtlD	0.49030694	0.374231693	0.190138511	0.411983443
yjjJ	0.490044176	0.237823844	0.039347495	0.150707625

garK	0.490012358	0.338980975	0.148304776	0.354473878
malG	0.488786866	0.234160985	0.03685229	0.144720522
zraR	0.488139665	0.483223565	0.312412148	0.551950915
yibH	0.487535679	0.2563438	0.057186573	0.190726867
trmH	0.48634396	0.242270059	0.044702239	0.16287269
yfiH	0.486204836	0.459572817	0.290078462	0.529167081
xylG	0.485965168	0.222540722	0.028983311	0.122647408
bglB	0.48591206	0.204939568	0.017739951	0.08985719
yhdP	0.485884593	0.295877771	0.100552682	0.275207578
cydD	0.485232	0.371420797	0.191409364	0.412337034
dnaE	0.48433277	0.20160122	0.016286562	0.084658051
yiaO	0.483978235	0.238139606	0.042120272	0.158448422
hyuA	0.483718452	0.259010731	0.061823226	0.199213425
recC	0.483607185	0.288095209	0.093222874	0.260263306
insH1	0.483075513	1.084989368	0.656149755	0.822328959
hcaD	0.482265101	0.395911869	0.223181316	0.45435349
rluA	0.481912915	0.233273856	0.038840977	0.14933431
yegD	0.481595347	0.466517745	0.301922487	0.542197989
spoT	0.481419065	0.208293986	0.020819159	0.098185682
yidE	0.480260739	0.217318143	0.027109109	0.117550452
nikA	0.480100614	0.340028543	0.157967011	0.369046774
pyrB	0.479897363	0.231997266	0.03858857	0.148963176
yhjC	0.479726996	0.250098836	0.055092038	0.186582682
dgoD	0.479219505	0.29055983	0.099086769	0.272244565
truC	0.478293498	0.472446053	0.311357852	0.551052892
yciV	0.477107127	0.281584419	0.090195715	0.257875425
mukB	0.473891376	0.311594314	0.128294935	0.320499166
oxyR	0.473763645	0.217217126	0.029178958	0.122959764
slt	0.471006018	0.260514819	0.070609354	0.217263689
wecC	0.470326595	0.237378771	0.047553846	0.169370362
yifB	0.469930714	0.211038242	0.025963572	0.113805583
pflC	0.468770939	0.221724971	0.034498187	0.138714323
parE	0.468715312	0.265895101	0.077937128	0.233753482
asnA	0.467756393	0.274729692	0.088641846	0.255605094
insB1	0.467609624	0.930355755	0.615235838	0.799075767
rsmC	0.4668873	0.365947121	0.202014668	0.424946857
tdcD	0.46660533	1.474197926	0.751611861	0.877797051
glmS	0.466154035	0.363019361	0.199106126	0.422159366
holB	0.466085947	0.274871134	0.089951694	0.257542545
ybiH	0.465949518	0.315595485	0.139832849	0.340705233
frvX	0.465905385	0.35233776	0.186059615	0.407997102
yiaK	0.465781818	0.22160047	0.035562119	0.141472322
yahJ	0.465483437	0.585970728	0.426974234	0.651434234
bisC	0.465108813	0.362367175	0.199306773	0.422362961
brnQ	0.464120433	0.23437091	0.04767188	0.169411425
prlC	0.463656084	0.292321702	0.112713033	0.296238617
argP	0.461785233	0.308047066	0.133854532	0.329695391
yjdA	0.46138937	0.299729684	0.123718482	0.313911887
macB	0.460838624	0.460028641	0.316459171	0.555356132

nanA	0.459990456	0.269083281	0.08736347	0.252821741
ttdT	0.459821335	0.463806526	0.321486569	0.561107382
ybhG	0.459764492	0.365215308	0.208071458	0.43454965
opgB	0.459087513	0.303225859	0.13002286	0.323614233
cpxA	0.458912666	0.224559963	0.040992231	0.154949098
murG	0.458273082	0.232407979	0.048627023	0.171897803
csrD	0.458184704	0.231472196	0.04776679	0.169492244
ygcQ	0.458070876	0.407607473	0.261096218	0.497895812
yicJ	0.458034669	0.230256814	0.046675387	0.166898617
cobT	0.457883411	0.233058549	0.04945231	0.173448284
yfaL	0.457577503	0.253302834	0.070848941	0.217669082
aas	0.454963854	0.292927891	0.120384939	0.308763659
cadA	0.454828549	0.26157895	0.082073264	0.242023204
agaS	0.454578319	0.332092633	0.171052459	0.385991491
chiA	0.454130874	0.203630831	0.025736321	0.113301556
mtlA	0.453726028	0.419787127	0.279764784	0.517603316
creC	0.453359873	0.390053499	0.245112906	0.478723175
lysR	0.452265945	0.257403986	0.078913081	0.235282207
sthA	0.452253801	0.445696948	0.31024339	0.54985303
malP	0.451751892	0.226119064	0.045732963	0.164880193
cynS	0.451510459	0.339954936	0.184129228	0.406191088
yibF	0.45150215	0.275855226	0.101685665	0.276665901
yfgH	0.450764241	0.334744595	0.17811182	0.397917773
glgX	0.450457114	0.228841712	0.049019476	0.17252975
glgB	0.449902303	0.207929925	0.030485784	0.12678797
abgA	0.449851451	0.339678073	0.18538801	0.407187919
mngA	0.448815898	0.47149456	0.341147456	0.577860031
viaA	0.447231495	0.242408395	0.065044737	0.206435222
aceA	0.447078683	0.233024844	0.055036489	0.186551054
btuF	0.445055406	0.374724504	0.234956764	0.467251456
wcaL	0.444619047	0.558666541	0.426114773	0.65104145
waaC	0.44392582	0.261203089	0.089216964	0.255983572
gsiC	0.4435052	0.374181056	0.235911112	0.467717972
afuC	0.442325134	0.449037972	0.324599206	0.563926663
mnmG	0.439731463	0.33668935	0.191537234	0.412392434
frmA	0.438253047	0.239604251	0.06738906	0.211711779
fepA	0.438049817	0.259761317	0.091727427	0.258953448
tauB	0.438041725	0.524921462	0.404004905	0.634124339
paaE	0.436319379	0.506339373	0.388845297	0.622009501
glpR	0.43628656	0.465539923	0.348674974	0.585320532
ybgS	0.435395268	0.2541847	0.086729277	0.251346798
wecB	0.434783853	0.227052573	0.055504763	0.187351779
menA	0.433589284	0.250387348	0.083331605	0.245196568
yigL	0.433227302	0.393159761	0.270500105	0.507911127
alkA	0.43271616	0.528497377	0.412919418	0.640088529
gudD	0.4325462	0.309061742	0.161649242	0.373542067
mutM	0.432514599	0.236686005	0.067643081	0.212179579
appA	0.432449232	0.414424499	0.296719833	0.535475176
hofO	0.432337079	0.497718126	0.385044942	0.619293399

gcl	0.43226129	0.318822999	0.175161745	0.393285853
yiaV	0.431884844	0.286194816	0.131283723	0.325548151
caiT	0.431628714	0.368521088	0.241500058	0.474652257
caiD	0.431032443	0.605069027	0.47623628	0.693566329
melB	0.4298917	0.218788263	0.049428648	0.173448284
yjdP	0.427177534	0.297819145	0.151471792	0.360124632
ybiB	0.427100913	0.248496263	0.085661398	0.248787815
ycaK	0.426841831	0.282121549	0.130286928	0.323774577
prmC	0.426161337	0.344373141	0.215901973	0.444665428
zraS	0.425462801	0.230194094	0.06456206	0.205711948
mpl	0.425013376	0.226078763	0.060116938	0.195868482
yjhC	0.424886385	0.240064878	0.076746884	0.231041888
pstA	0.422655809	0.27484014	0.124091614	0.313911887
ligB	0.42123996	0.308715452	0.172412571	0.388312622
hycI	0.421063044	0.285549686	0.140328145	0.341473612
dapB	0.420331464	0.224747875	0.061451115	0.198462522
dmsA	0.419565085	0.25173192	0.095571312	0.265169339
aroB	0.419362682	0.275373481	0.12778699	0.31982398
opgH	0.417757693	0.306012302	0.172200776	0.388148818
ygbN	0.417469267	0.290270634	0.150375322	0.358361968
yidA	0.415698977	0.281645269	0.13995289	0.340765874
bioH	0.415157702	0.432994239	0.337656221	0.575398128
dcuD	0.415111751	0.292148911	0.155348458	0.365489836
bglH	0.413218694	0.24297837	0.08901064	0.255835955
galR	0.413117373	0.24353665	0.089824297	0.257360318
hycF	0.41241725	0.40422936	0.307607276	0.546748622
mnmC	0.412093509	0.440517807	0.349543428	0.586251275
glgP	0.411660518	0.331997533	0.214993691	0.443446489
eutB	0.411200809	0.319892142	0.19864004	0.422057811
rffH	0.410410697	0.264536511	0.12079793	0.309308482
yfbT	0.409701125	0.258297755	0.112703407	0.296238617
mscK	0.408992715	0.222750997	0.066343128	0.209403602
yidZ	0.408637394	0.279272501	0.14340691	0.34654665
ccmH	0.408231273	0.271251489	0.132326178	0.327328911
pstC	0.408128129	0.280494549	0.145660813	0.349811245
sbcC	0.405700819	0.408661763	0.320829591	0.560818235
actP	0.404781795	0.272303268	0.137144018	0.336024546
amiC	0.403907362	0.213440254	0.058442056	0.192282462
eutM	0.403813411	0.359292926	0.26105062	0.497895812
insJ	0.403487577	0.22537001	0.073400243	0.223199053
ygfT	0.403389031	0.315547453	0.201116055	0.424414801
waaF	0.402861757	0.226685085	0.075537499	0.22810419
fliF	0.402400972	0.51101637	0.431017192	0.654387516
mobA	0.40185636	0.361625127	0.266460234	0.504076835
ugpB	0.400920052	0.245139125	0.101948536	0.276900479
wzzE	0.400785317	0.239511988	0.094260114	0.262614273
hemL	0.40040759	0.237505365	0.091817255	0.258953448
fliP	0.398699776	0.575369697	0.488343826	0.70133192
ispF	0.39757363	0.267474125	0.13717326	0.336024546

yfjQ	0.396252539	0.41492918	0.339583581	0.576977659
ybhF	0.395545813	0.376131224	0.29297552	0.531808531
yihW	0.395090767	0.245795378	0.107967295	0.287889015
gspE	0.39505608	0.315050018	0.209861207	0.437008586
malS	0.393184241	0.300779428	0.19113873	0.411983443
yfjY	0.39226953	0.276390307	0.155823416	0.365519541
gspL	0.392217638	0.215020382	0.068137687	0.213152669
yadC	0.391669115	0.491630608	0.425640634	0.650875469
yghJ	0.391405697	0.226226303	0.083603275	0.245816766
yjdF	0.390745951	0.226581461	0.084612667	0.247522707
yifK	0.390257061	0.231475219	0.091804252	0.258953448
hofQ	0.39020577	0.416270153	0.348560094	0.585320532
rsmD	0.389625818	0.364901371	0.285630356	0.523655652
flhB	0.389385177	0.496746968	0.433116239	0.655054902
eutH	0.388855902	0.544435232	0.475080511	0.692632727
yraK	0.387539261	0.303776005	0.202047373	0.424946857
ygiQ	0.387523339	0.238842706	0.104695393	0.281372027
rffG	0.387126827	0.292680983	0.185937789	0.407951551
ygfF	0.387120291	0.404766926	0.338868685	0.576005424
kup	0.386692096	0.200514805	0.053793402	0.183570551
ruvB	0.386637959	0.376657003	0.304656532	0.544689032
gspA	0.38596849	0.210745492	0.067034354	0.21092571
yajR	0.385317583	0.370490586	0.298330655	0.537181469
cysQ	0.385103144	0.419327739	0.358418791	0.594734274
queA	0.384299247	0.280241156	0.170276397	0.384904479
allC	0.383531486	0.332922208	0.249314775	0.484066669
casC	0.382786757	0.275881348	0.16528782	0.378698598
yheS	0.382665861	0.320855433	0.233009372	0.46567269
yiaU	0.38145637	0.226311072	0.091884429	0.258953448
wzyE	0.381126526	0.36224005	0.292736235	0.531613217
rlmH	0.380976651	0.41276615	0.356015419	0.592896394
waaB	0.380469105	0.340713147	0.264129229	0.501547365
yaaW	0.379923631	0.252534086	0.132466838	0.327476193
frvR	0.379842551	0.303342609	0.210500676	0.437585597
paaF	0.379791161	0.593680047	0.522352283	0.731438143
tnaA	0.377565195	0.467782152	0.419586434	0.645529891
cmtA	0.376931684	0.240334057	0.116795818	0.303169594
clcA	0.376301039	0.436145055	0.388253636	0.621758205
accC	0.376239241	0.501996124	0.453564124	0.672699636
ispH	0.376017407	0.265225775	0.156270853	0.365719093
nikR	0.375944404	0.419038564	0.369633844	0.604379032
narV	0.375836019	0.287260317	0.190755824	0.411983443
prkB	0.375542955	0.229428615	0.101659332	0.276665901
speB	0.375514653	0.23741368	0.11372054	0.29791682
fucO	0.375426556	0.268518836	0.162071443	0.374060132
rarA	0.375070842	0.26859751	0.162592643	0.374436109
yieL	0.374641503	0.232674993	0.107365241	0.286662354
uacT	0.374332307	0.262998948	0.154642702	0.364444009
gntX	0.374267103	0.222412466	0.092421299	0.259280599

xseA	0.372132848	0.351166186	0.289278459	0.528663258
dmsC	0.371936664	0.227725596	0.102412887	0.277105112
cyaA	0.371331632	0.306644333	0.225913722	0.45692069
yhjJ	0.369934223	0.369121673	0.316246376	0.555356132
ycjU	0.367623136	0.647678505	0.570305751	0.768209648
thiC	0.367552069	0.443556775	0.407304085	0.635841683
gudX	0.367170208	0.313199133	0.241067836	0.474264549
glmM	0.366959308	0.213256473	0.085297704	0.248267362
prfC	0.366649816	0.256908639	0.153533688	0.363315064
dnaJ	0.366614083	0.454906062	0.420293809	0.645633983
acrF	0.366004232	0.212500065	0.085002093	0.247717609
aceK	0.365763577	0.268458749	0.173053617	0.38941887
ytfH	0.365632669	0.431552579	0.396856117	0.628524184
nagE	0.365556823	0.206912833	0.077275308	0.23228624
glnD	0.365092804	0.373887198	0.328827405	0.56686713
malI	0.364856389	0.431806741	0.398137097	0.629934022
yedJ	0.364721112	0.572856463	0.52433915	0.732652886
ltaE	0.364277043	0.573202546	0.525094604	0.732989943
murD	0.364119176	0.215653908	0.091326361	0.258953448
yhgA	0.363621777	0.294365393	0.216728723	0.445712611
hcaF	0.36332059	0.400610018	0.364449958	0.600034454
casA	0.362754561	0.3046525	0.233765152	0.46608109
yhfK	0.362646788	0.273178372	0.184340316	0.406392768
aaeA	0.362462202	0.226303574	0.109230014	0.290488517
waaR	0.362323667	0.338343612	0.284225703	0.522028736
argC	0.361999658	0.22789938	0.112191671	0.295445386
casD	0.361110597	0.270350779	0.181643646	0.402025987
bcsE	0.359830194	0.314482633	0.252541803	0.488271675
pheT	0.357654082	0.219707698	0.10355389	0.279130296
rep	0.357002548	0.263067566	0.174757223	0.392814538
ybjT	0.356264403	0.301904912	0.237978338	0.470248923
yiaT	0.355883811	0.425762714	0.403226017	0.633394332
pfkB	0.3558126	0.507399958	0.483149004	0.699190852
uraA	0.355723269	0.427929086	0.40582289	0.635248936
hemD	0.355487776	0.312326758	0.255040374	0.490986166
nrfA	0.355179554	0.210446249	0.091460236	0.258953448
insF1	0.353947402	1.726351434	0.837551597	0.918054792
dnaG	0.353383122	0.220935347	0.109712529	0.29113325
yicG	0.353141722	0.341338962	0.300866113	0.54102294
kefG	0.353125255	0.291035133	0.224998953	0.456213346
waaG	0.352956396	0.261255344	0.176694909	0.395847584
fryC	0.34994076	0.527814114	0.507330552	0.720343379
dpiB	0.349832349	0.332802937	0.293180676	0.531899599
fucI	0.34973266	0.213180527	0.100891498	0.275760986
mfd	0.349710885	0.518838114	0.500294163	0.712411829
ygcE	0.349495428	0.270665795	0.196619282	0.418866512
csdA	0.349370185	0.377362099	0.354538421	0.590946162
sapA	0.34935512	0.354119965	0.323865967	0.563797719
caiE	0.348940217	0.253151531	0.16808452	0.382670705

dapE	0.348241485	0.254715479	0.171569414	0.386941745
dnaN	0.346012343	0.328700826	0.292493875	0.53151325
pyrE	0.344955273	0.364991293	0.344605025	0.580858948
yjhF	0.344799357	0.280057707	0.218258342	0.4486298
insE1	0.343862505	0.665237165	0.605224484	0.794307946
gspD	0.343565761	0.237496361	0.148004827	0.35396652
yhfX	0.34344273	0.304265894	0.258999701	0.495301654
aaeB	0.343099371	0.363133397	0.344745613	0.580858948
insI1	0.342127832	1.04726377	0.743904365	0.872446923
qseC	0.341882098	0.238875559	0.152368829	0.361831155
mqo	0.341662132	0.400288195	0.393359713	0.626427283
ispD	0.341451583	0.287866844	0.235565364	0.467572833
adiY	0.340933174	0.380929901	0.370785807	0.605771875
ycjS	0.340844736	0.51790398	0.51045883	0.722170021
btuB	0.340579732	0.384321892	0.375518635	0.61029337
hyfI	0.340139853	0.733415291	0.642808784	0.814447504
fhuD	0.339360444	0.305128395	0.266056898	0.503785975
trkA	0.338955111	0.285948819	0.235871244	0.467717972
tatD	0.336568254	0.299192919	0.260622715	0.497698156
yhjY	0.336456337	0.50072132	0.501619793	0.714047639
gcvP	0.335835374	0.20546489	0.102150832	0.276900479
ynfG	0.335749849	0.328179556	0.306275917	0.54612892
rlmM	0.335546664	0.352205249	0.340741052	0.577728528
yhfT	0.335509547	0.296218881	0.257365189	0.493447271
proB	0.334943576	0.482757259	0.487799081	0.7011811
ampC	0.334737192	0.228041863	0.142137892	0.344838143
malF	0.334452578	0.421995173	0.428039631	0.651957102
mdlA	0.334352198	0.224193885	0.13586887	0.333567003
ulaG	0.333051515	0.251722425	0.185805328	0.407882605
parC	0.333030992	0.287280086	0.246352094	0.480446089
yphE	0.332285356	0.443362948	0.45357652	0.672699636
trkH	0.332228293	0.222450704	0.13530851	0.332848571
ppdB	0.332184759	0.231765856	0.151778583	0.360641636
yqcE	0.331879201	0.24098156	0.168451196	0.383119707
yigM	0.331677214	0.357503499	0.353532414	0.589756345
yhdY	0.331558407	0.241893752	0.170475405	0.384904479
yraP	0.32860976	0.302077428	0.27666889	0.51382911
pcnB	0.328498449	0.229648425	0.15259058	0.361931946
moaC	0.328305715	0.362151067	0.364648224	0.600047487
dbpA	0.327718538	0.333569257	0.325873153	0.564613699
gmd	0.327069304	0.511228812	0.522321571	0.731438143
tktA	0.326336988	0.37023341	0.378081979	0.612978694
ampG	0.326112101	0.278798617	0.242119757	0.475407324
ytfR	0.324527379	0.376848678	0.389149384	0.622009501
rsmA	0.324396904	0.406277575	0.424602866	0.649764616
nuoN	0.323925843	0.259038365	0.211119257	0.437969394
idnT	0.323007114	0.248683611	0.193989331	0.415676715
ccmB	0.322680962	0.496976714	0.516152311	0.726283332
yohF	0.322660932	0.290894905	0.267343462	0.504771323

murB	0.320759519	0.473057272	0.497735922	0.710272152
aroE	0.320111811	0.337376642	0.342708993	0.579121896
nanR	0.319919814	0.330966159	0.333732059	0.571542559
recA	0.318552374	0.232337086	0.170350498	0.384904479
eptC	0.318404277	0.283850643	0.261976515	0.499315686
flgI	0.318129208	0.359945945	0.376790715	0.61135866
gshB	0.318076741	0.325575679	0.328585419	0.56686713
yraJ	0.317969712	0.223100547	0.154090573	0.363526107
yehW	0.317013457	0.714424467	0.657235892	0.822971866
rsmI	0.316270293	0.360550001	0.380384295	0.615228925
mhpE	0.31622829	0.619040752	0.609465912	0.796587383
creB	0.315207063	0.239662791	0.188439106	0.410760621
ppdC	0.313555938	0.408343455	0.442562604	0.662694818
yjcC	0.313457317	0.262539848	0.232500708	0.465116629
ddpF	0.312099831	0.363134231	0.390086043	0.622441643
bglJ	0.311964955	0.261897616	0.233586298	0.46608109
alsA	0.310872302	0.254595918	0.222070128	0.453005107
yjjG	0.310736177	0.307500058	0.312244325	0.551950915
hycH	0.310712443	0.405026467	0.442997638	0.662957587
folP	0.310348154	0.315748603	0.325658448	0.564483964
hflK	0.310243935	0.332676983	0.351043021	0.587545886
napF	0.309693001	0.412576079	0.452873875	0.672398615
proC	0.308270509	0.257795227	0.231776166	0.46435751
cbrB	0.308091752	0.482502469	0.523129472	0.73186377
recN	0.307655787	0.300616337	0.306110856	0.546075795
cydC	0.307575264	0.354017295	0.384948748	0.619293399
hyfR	0.307406061	0.346686554	0.375241847	0.61029337
pgl	0.307147493	0.246424187	0.212611049	0.440159387
alsE	0.306329253	0.280182606	0.274253315	0.51162691
ytjC	0.304921367	0.242472298	0.208554055	0.43510735
glrK	0.304241063	0.234030707	0.193599293	0.415061256
flhA	0.30376123	0.461114373	0.510053344	0.721979435
melR	0.303684201	0.32408383	0.34873054	0.585320532
fdnG	0.303119227	0.428858127	0.479687436	0.696832738
gspJ	0.30177923	0.2770152	0.275979329	0.513523158
allB	0.301778828	0.2669591	0.25829468	0.494656369
yidP	0.301260804	0.214614806	0.160399872	0.371718877
srmB	0.301259682	0.247881117	0.22423668	0.455812425
trpE	0.301154249	0.458809809	0.511578721	0.722751135
birA	0.30087046	0.215282078	0.162244065	0.374060132
nudL	0.300359929	0.267827162	0.262088169	0.499315686
dacB	0.300296411	0.264951206	0.257045183	0.493433858
alsC	0.300106668	0.377936113	0.427156317	0.651465829
yihO	0.299861813	0.230333345	0.192964183	0.414472668
lysC	0.298865142	0.245259973	0.223009459	0.454232688
ompL	0.298722965	0.363787582	0.411562724	0.639522216
lepA	0.297523773	0.352447135	0.398576833	0.629934022
avtA	0.296542649	0.215530643	0.168861162	0.383619871
yihF	0.296508674	0.257358338	0.249270192	0.484066669

suhB	0.296235739	0.625420745	0.63574358	0.810643346
yibD	0.296015211	0.239339981	0.216162083	0.444773867
ycaD	0.295304108	0.230935508	0.200992234	0.424378054
bglA	0.295148286	0.250562442	0.238819313	0.471218753
hofC	0.29466569	0.269336127	0.273935492	0.511270265
fliD	0.294353595	0.365071517	0.420075526	0.645633983
yfhM	0.29337323	0.295915859	0.321486593	0.561107382
yraR	0.292076513	0.245369011	0.233906438	0.46608109
hslV	0.290998337	0.289580573	0.314946969	0.55448771
narZ	0.290300114	0.547683005	0.596076331	0.787971293
ynjE	0.290249834	0.388260555	0.454722844	0.673657292
dnaT	0.289908771	0.23958708	0.226265433	0.45717395
cpsB	0.28964388	0.571503645	0.612287994	0.797871734
pgm	0.289123013	0.287795971	0.315084173	0.55448771
cusR	0.288934994	0.263952896	0.273671735	0.511250715
nanM	0.286945027	0.26099577	0.271583144	0.509169165
dgoT	0.285984239	0.409809616	0.485273145	0.700410328
yhjX	0.285920762	0.208559325	0.170395991	0.384904479
pdxA	0.285434746	0.261233091	0.274550176	0.511944138
ygcS	0.283770444	0.466017387	0.542573005	0.747310549
yhfZ	0.283031025	0.230806086	0.22009636	0.45011601
insE1	0.282323663	0.601468293	0.638790347	0.812731368
narG	0.282132516	0.397249286	0.477570642	0.695008176
hisH	0.281790055	0.345860982	0.415215726	0.6424783
trg	0.281584346	0.341561213	0.409710183	0.637655644
clsA	0.281279079	0.389765657	0.470502285	0.688801332
dfp	0.28088494	0.231494396	0.224994028	0.456213346
murE	0.279800279	0.26748227	0.29553717	0.534057097
pepE	0.279415615	0.278244409	0.315277756	0.554568708
rlmL	0.279372084	0.366203488	0.445530458	0.665903909
cbrA	0.279070901	0.348291233	0.422982692	0.648306096
metN	0.278296893	0.226628399	0.219451574	0.449723299
yphC	0.277957413	0.452555731	0.539086556	0.744335164
yqeA	0.277938932	0.414509638	0.502523395	0.71482979
ubiD	0.27670206	0.232752393	0.234508207	0.467049645
baeS	0.276593906	0.645157974	0.668124997	0.829537807
yeaD	0.276480016	0.419144313	0.509491522	0.721572898
ftsX	0.274609186	0.333602792	0.410415799	0.637985591
ybbA	0.27455006	0.400655631	0.493184935	0.706023965
yfjK	0.274371346	0.29544048	0.353051999	0.589253694
garP	0.272601403	0.221654181	0.218753267	0.449359873
ycfJ	0.271021638	0.246658378	0.271866965	0.509169165
gcvA	0.270901987	0.244772363	0.268401723	0.505865854
valS	0.27087464	0.257746735	0.293289202	0.531899599
polA	0.270747117	0.231380612	0.241945664	0.475296664
edd	0.270590469	0.291389322	0.353085134	0.589253694
pstS	0.269751865	0.219376666	0.218836143	0.449359873
yceM	0.269294564	0.500793361	0.590759398	0.784081396
phoU	0.267849276	0.427953125	0.531390628	0.73882415

metA	0.267727049	0.296525081	0.366588721	0.602050552
sgcQ	0.267608778	0.327036957	0.413195617	0.640088529
msrA	0.267415794	0.271966329	0.325475546	0.564483964
secD	0.267387102	0.46884554	0.568467747	0.7665011
gadX	0.267369767	0.341483654	0.433647635	0.655423251
ompR	0.267291208	0.403885078	0.508099306	0.720476606
fhlA	0.266776874	0.298242005	0.371055506	0.605967264
yqiK	0.266254095	0.214831869	0.215212087	0.443497292
hypB	0.266067335	0.421279192	0.527667839	0.736072933
yqhD	0.265139465	0.363112072	0.465276308	0.684020559
yejM	0.264363926	0.249748257	0.289817705	0.529167081
setC	0.263831016	0.318739057	0.407821405	0.636157269
dcuB	0.263308693	0.242655665	0.277871836	0.514605833
glgC	0.263227576	0.339436748	0.438054048	0.659079275
dinG	0.263035485	0.320845559	0.412318943	0.640088529
sufS	0.262890368	0.47946604	0.583487161	0.77791865
waaL	0.262627382	0.308880302	0.39518268	0.62720056
yeeJ	0.262477834	0.269190841	0.329529369	0.567012106
yhjA	0.260020983	0.325758344	0.424753238	0.649764616
yegE	0.259866716	0.341799909	0.447081846	0.667481292
bioB	0.259187371	0.515902971	0.615389289	0.799075767
rhtC	0.257710105	0.26445616	0.329812885	0.567012106
ybbO	0.25722154	0.351240418	0.463971266	0.682350455
yhhI	0.256920896	0.27003939	0.341391175	0.577860031
pta	0.256849937	0.321569344	0.424441837	0.649764616
hslU	0.255597704	0.222812719	0.251323169	0.486381415
melA	0.255172212	0.212773473	0.230424129	0.462566985
ybhR	0.255058931	0.219589464	0.245427947	0.47887512
yoaA	0.254488962	0.434999751	0.558525911	0.758376177
araE	0.253966915	0.233267099	0.276269136	0.513725703
gntR	0.253965358	0.343379826	0.459539845	0.677808678
clsC	0.253417937	0.229878574	0.270288195	0.507911127
yqjD	0.252098312	0.282327986	0.371896209	0.606604039
yejB	0.25178676	0.403342905	0.532463494	0.739186769
gspK	0.251667991	0.221712085	0.256328503	0.492631115
fliK	0.251224423	0.491874063	0.609526487	0.796587383
ycgV	0.250773547	0.271984253	0.356520778	0.592896394
nrdE	0.250626044	0.409771978	0.540787093	0.74615003
ycfS	0.249864661	0.25597432	0.328999179	0.56686713
lpxH	0.249452656	0.254769661	0.327515677	0.56600205
glpT	0.24916113	0.391774244	0.524788549	0.732816109
rutE	0.248434529	0.488708902	0.611208831	0.797627992
tyrR	0.248384423	0.360197112	0.490458874	0.703368552
ybaA	0.247986651	0.61242672	0.685532928	0.836607144
ysgA	0.247243844	0.371715541	0.505959284	0.718957279
gpp	0.246848336	0.274947524	0.369291314	0.604255151
tatB	0.246175592	0.27955457	0.378534672	0.613112592
ygfQ	0.245836047	0.235751092	0.297051132	0.535833521
flgL	0.245285259	0.312937303	0.433148096	0.655054902

yhhT	0.244944597	0.219992903	0.265527897	0.503020235
yigI	0.244695178	0.233060341	0.293753978	0.53235683
queC	0.244645513	0.290945707	0.400424626	0.631204301
ccmA	0.244036727	0.309280666	0.430085019	0.653463765
yaiW	0.243859853	0.417199094	0.558872709	0.758376177
yihN	0.243698268	0.23686929	0.303559496	0.544411233
alx	0.243573543	0.241863764	0.313901531	0.553604326
hemX	0.243429665	0.254507383	0.338832872	0.576005424
yhhN	0.243085774	0.348911246	0.485991799	0.700946371
malM	0.242099641	0.271988317	0.373406586	0.608084868
gadW	0.241602627	0.410143711	0.555815508	0.75646253
yjgN	0.241417305	0.253650601	0.341213095	0.577860031
yedQ	0.240977533	0.24059447	0.316540614	0.555356132
purT	0.240834723	0.33030772	0.465926857	0.684478429
ycbB	0.240703067	0.223044548	0.280511785	0.517916666
ygcU	0.240381511	0.237401571	0.311274044	0.551052892
mdtG	0.240302746	0.237136659	0.310892384	0.550711959
glsA	0.240215043	0.289411696	0.406532267	0.635374504
ybaL	0.238980434	0.234104174	0.307335251	0.546748622
frvA	0.23882018	0.280686431	0.394856383	0.62720056
fdnH	0.238755658	0.511395798	0.640592469	0.813768269
argE	0.238401796	0.258563188	0.356515391	0.592896394
gfcD	0.237371594	0.330054235	0.472024068	0.6903603
glmU	0.236281457	0.315989848	0.454610874	0.673657292
insA	0.236260645	0.349428382	0.498955145	0.711508979
atoE	0.236220696	0.418147738	0.57212708	0.76989234
ycjG	0.235991227	0.335904876	0.4823339	0.699167668
ubiG	0.235177457	0.244433231	0.335982431	0.57363869
caiA	0.233822239	0.225374613	0.299511001	0.53906639
speC	0.233600994	0.280602819	0.405128493	0.63502421
appC	0.233535595	0.342836427	0.495753398	0.708444767
ytjB	0.23327993	0.401524003	0.561249711	0.76083448
yibA	0.233021039	0.391576834	0.551787442	0.752895703
yicL	0.232872463	0.212617192	0.273399951	0.510979446
mhpA	0.232251595	0.677017722	0.731560106	0.86505804
degS	0.232078052	0.260066803	0.372190034	0.606838113
yeaL	0.231431286	0.318199803	0.467033404	0.685605036
wecF	0.231386873	0.293664726	0.430738399	0.654210277
nrdA	0.230797456	0.210084219	0.271944794	0.509169165
yiaA	0.22986986	0.318407783	0.470334176	0.688801332
yggP	0.229846388	0.382799572	0.548216146	0.750792319
phoA	0.228977195	0.367488599	0.533227664	0.739307945
torC	0.22897359	0.275283596	0.405536825	0.635047386
hscC	0.228967936	0.48134598	0.634300476	0.810169369
psd	0.228503584	0.409689063	0.577015928	0.774124131
nrfG	0.228442906	0.325931951	0.483370783	0.699190852
dsdX	0.228343836	0.265852176	0.390388629	0.622678346
ydiS	0.228252795	0.558063526	0.682533332	0.83430303
kduI	0.227731974	0.26983213	0.398682543	0.629934022

yijE	0.227421409	0.387338301	0.557110381	0.757257443
intS	0.227002459	0.217353017	0.296302559	0.53520064
yiiG	0.226687343	0.253727022	0.371626922	0.606526668
thiI	0.226535797	0.259467306	0.382619301	0.616969633
ydjN	0.226241711	0.31491753	0.472500984	0.690617839
potI	0.226175904	0.544138289	0.677659644	0.833940919
fklB	0.226089983	0.245027481	0.356156916	0.592896394
yeaH	0.225899033	0.432234777	0.601231483	0.791794473
ecpB	0.225648665	0.578123815	0.696305847	0.843886732
endA	0.225007278	0.382114534	0.55596342	0.75646253
holD	0.224848267	0.298874545	0.451860717	0.671516637
cdh	0.224696597	0.280438565	0.422996843	0.648306096
yigP	0.224689532	0.266959908	0.399978176	0.630993316
cysG	0.224399338	0.272381355	0.410029053	0.637655644
yjfF	0.223283369	0.259308877	0.38919891	0.622009501
htrE	0.221627753	0.224954892	0.324521052	0.563926663
yhhY	0.221085361	0.617035144	0.720116794	0.858061244
yehX	0.220216234	0.452139259	0.626220064	0.805368079
cusS	0.219956392	0.283253909	0.437433544	0.658788667
tauD	0.219760356	0.352027239	0.532449701	0.739186769
yfjP	0.219431472	0.291527315	0.451632557	0.671516637
ymdB	0.218195299	0.258403307	0.398446903	0.629934022
mraY	0.218139442	0.400092055	0.585600066	0.779448555
nhoA	0.217986398	0.291087077	0.453935525	0.672984838
hisI	0.217965152	0.235084972	0.353835145	0.590017547
sfmD	0.217323153	0.336655381	0.518579925	0.728429769
ypdA	0.216942668	0.286422097	0.448796516	0.669051527
hofP	0.216631987	0.290412071	0.455699485	0.674114629
yhiJ	0.215891801	0.401722335	0.590980654	0.784081396
fixA	0.215749334	0.235168472	0.358920204	0.594734274
rhaR	0.215304281	0.241864604	0.373366473	0.608084868
trmI	0.215211047	0.237056131	0.363958283	0.599779298
hisC	0.214963927	0.514217096	0.675917001	0.833183796
gspF	0.214562394	0.297284112	0.47045319	0.688801332
narX	0.214416396	0.4436539	0.628885194	0.807067525
bacA	0.214104248	0.229254914	0.35034854	0.587344027
proS	0.214065262	0.276609907	0.438996654	0.659079275
ravA	0.213605703	0.232757622	0.358766875	0.594734274
xylE	0.211881245	0.226946194	0.350500537	0.587344027
yjfZ	0.211862761	0.35827455	0.554291345	0.754950796
tsaA	0.211510238	0.337065178	0.530327963	0.738253098
rhtB	0.210536716	0.264888719	0.426722906	0.651399331
gss	0.20977057	0.22912171	0.359907114	0.59546927
oppC	0.209027065	0.253207855	0.40907886	0.637379914
idnD	0.208489609	0.243413743	0.391708238	0.624043471
ycbV	0.20833642	0.241477779	0.388271597	0.621758205
ftsK	0.208273371	0.202650771	0.304069668	0.544689032
ybcH	0.208117239	0.427243743	0.626176168	0.805368079
ydiR	0.207778255	0.511024607	0.68430805	0.836123365

yieK	0.207462653	0.256406816	0.418448884	0.645501776
rhaS	0.207229484	0.273939034	0.449361277	0.669619977
frc	0.206874008	0.334268694	0.535991871	0.74178923
setA	0.205944857	0.211374133	0.329900467	0.567012106
wecG	0.205858058	0.246832984	0.40428247	0.634313382
yfeH	0.205748394	0.247314745	0.405448826	0.635047386
fadI	0.20548685	0.211378255	0.33098657	0.567867735
yejH	0.205125149	0.444048061	0.644121784	0.814620978
gudP	0.204757982	0.357664596	0.566992752	0.765279084
yiiQ	0.204671196	0.266412365	0.442338905	0.662605625
yghA	0.204207994	0.219437906	0.352062686	0.588520523
ybiO	0.202446537	0.4423901	0.647225505	0.817244062
frlR	0.20194801	0.233450973	0.387008417	0.621213908
smf	0.201899456	0.210651558	0.337834768	0.575459476
narI	0.201178874	0.308962988	0.514954627	0.725705969
tdh	0.201092457	0.400256145	0.615379555	0.799075767
nudC	0.200912803	0.264028495	0.446686208	0.667137338
yjeM	0.200408803	0.232605224	0.388916802	0.622009501
mdtH	0.200189592	0.408708905	0.624268557	0.8040357
idi	0.200086875	0.325790823	0.53911126	0.744335164
ygeX	0.199709492	0.229643101	0.384490824	0.619293399
gluQ	0.199093008	0.391155703	0.610761622	0.797627992
hflX	0.198899964	0.401402202	0.620238129	0.802928873
dxs	0.198896806	0.283861188	0.483500654	0.699190852
tyrA	0.198701056	0.224206397	0.375487304	0.61029337
allD	0.198532994	0.314744597	0.528187714	0.736289296
mgtA	0.198035712	0.228842234	0.386829729	0.621174071
yjjI	0.196727241	0.208543925	0.345508456	0.581416273
elfC	0.195887446	0.313037626	0.531470233	0.73882415
paaH	0.194996067	0.434437556	0.65354191	0.820942771
fes	0.194917755	0.421079848	0.643436228	0.814534981
znuB	0.194158693	0.239796502	0.418123489	0.645246379
ybiY	0.193958153	0.528337348	0.713536645	0.853748499
galP	0.193829202	0.235282895	0.410045592	0.637655644
xanQ	0.192460504	0.219073825	0.379662166	0.614553394
lysA	0.191970876	0.346024773	0.579038719	0.774124131
exuT	0.191953549	0.727979866	0.792026701	0.895647744
hyfA	0.191800132	0.282082734	0.496540662	0.709067794
ssuC	0.191433891	0.547683235	0.726688479	0.863370914
etk	0.191416679	0.371963048	0.60682412	0.795201383
caiC	0.190384902	0.293666856	0.516789439	0.726926468
yhcF	0.190290297	0.538969267	0.724040945	0.861211931
nuoK	0.190032239	0.626663625	0.76170361	0.882955657
tnaB	0.189216904	0.340385841	0.578286419	0.774124131
cpsG	0.189022763	0.562461247	0.736822891	0.867963206
insF1	0.188858709	0.991852768	0.848987843	0.923651849
mdfA	0.188786622	0.340753255	0.579560129	0.774124131
barA	0.187754247	0.324516532	0.562882021	0.762023715
rhaT	0.187723168	0.265833891	0.480084182	0.697158217

torR	0.186739934	0.271988958	0.492352637	0.705332716
yadE	0.186591351	0.246204782	0.448528488	0.668898968
treB	0.186227587	0.216432746	0.389546048	0.622281223
fecI	0.186067898	0.236407669	0.431244568	0.654486588
cadC	0.185991111	0.304525781	0.541360772	0.74615003
ecpC	0.185959562	0.451731178	0.680588801	0.83430303
speF	0.185219093	0.276846147	0.503474693	0.715930728
astB	0.184741754	0.537376566	0.73100833	0.864719669
murQ	0.184606471	0.426029217	0.664783178	0.828231584
yadV	0.184460673	0.46737488	0.693083557	0.841750819
yehP	0.184114616	0.429597933	0.668233081	0.829537807
garR	0.184089311	0.240004251	0.443066527	0.662957587
ptrA	0.183272293	0.261806393	0.483908655	0.699190852
yhaJ	0.183191748	0.263540296	0.486981013	0.7011811
wcaK	0.183067172	0.365512228	0.616475976	0.800200669
elfG	0.182655585	0.236318786	0.439569554	0.659334251
yceG	0.182511719	0.233860163	0.435137232	0.656936801
citT	0.18246966	0.278173174	0.511851745	0.722751135
yggI	0.181466639	0.243176593	0.455526081	0.674105128
nuoM	0.180298427	0.358185044	0.614706473	0.799075767
gpmM	0.180284278	0.20544834	0.38020624	0.615187411
agaC	0.178910801	0.433659904	0.679928892	0.83430303
ygcB	0.178909649	0.256685301	0.485802808	0.700924209
waaZ	0.17889824	0.413431283	0.665221266	0.82834616
frwD	0.178733541	0.420313273	0.670662551	0.830765486
waaJ	0.17819777	0.290157726	0.539122125	0.744335164
ygbT	0.177940934	0.335498173	0.595849704	0.787971293
aaeR	0.177932003	0.224919423	0.428890185	0.652387716
yqeI	0.177794447	0.253604891	0.483260153	0.699190852
mngB	0.176375539	0.218788507	0.420158516	0.645633983
gabP	0.175473287	0.363362337	0.62915577	0.807067525
rng	0.175413196	0.222183702	0.42982203	0.653463765
mrdA	0.175136819	0.208873038	0.401758612	0.6323195
dsdA	0.17325261	0.354443487	0.624982032	0.804288321
atoB	0.172587296	0.311052927	0.578998187	0.774124131
fucK	0.171554898	0.234092053	0.463648521	0.682300887
nupX	0.171062267	0.555725123	0.758220726	0.880985096
ydcT	0.170316157	0.357072037	0.633376489	0.809822208
yhhW	0.16944146	0.352168922	0.63041921	0.807625212
rpe	0.169271026	0.421758232	0.688165319	0.838299153
ydhC	0.169184664	0.366454946	0.644311316	0.814620978
frlA	0.168710892	0.362110418	0.641279868	0.814102776
atoS	0.168335059	0.287972	0.558847938	0.758376177
cpdB	0.167405556	0.309689135	0.5888109	0.782690025
mutH	0.166992565	0.271307166	0.538217259	0.744102043
yfdV	0.166678346	0.217582435	0.443648408	0.663336527
dcuS	0.164730853	0.26159819	0.528884697	0.736752078
tehA	0.164500662	0.423299312	0.697560619	0.84420259
ldcC	0.164162324	0.281253189	0.559434519	0.758883452

evgS	0.163218082	0.267187232	0.541282133	0.74615003
emrK	0.162353588	0.280365063	0.562535706	0.761810347
ligT	0.162268207	0.499008349	0.745043826	0.872707636
rclA	0.162067585	0.694105069	0.815379824	0.908982164
yedE	0.162019039	0.425341145	0.703265944	0.847235039
fruA	0.161884654	0.294246452	0.582204916	0.776713973
wza	0.161471536	0.301439805	0.592188303	0.784081396
yiaJ	0.161451591	0.314206972	0.607365015	0.795201383
tsaC	0.161159967	0.413704359	0.696866768	0.84420259
yhbX	0.160802372	0.30045709	0.592516773	0.784259086
groL	0.160455938	0.449134959	0.720900535	0.858741652
epmB	0.160146662	0.352428819	0.649534814	0.819157847
ybgQ	0.159162227	0.403710591	0.693397705	0.841789168
mglA	0.158530138	0.60238418	0.792418666	0.895647744
cof	0.158365688	0.265469487	0.550808199	0.751982651
paaZ	0.158305406	0.399735523	0.692086317	0.841298543
ycjN	0.157902146	0.343004106	0.645264587	0.815315536
aroP	0.157558085	0.31015856	0.611458914	0.797627992
basR	0.156733224	0.403216236	0.697492443	0.84420259
yhiM	0.156012196	0.263550314	0.553874954	0.754638269
yggT	0.153398137	0.285815639	0.591472721	0.784081396
ybhA	0.153283787	0.276124023	0.578808076	0.774124131
pstB	0.152636562	0.284529935	0.591646421	0.784081396
hyaC	0.152482603	0.371623649	0.681575195	0.83430303
yjjQ	0.152045252	0.243686196	0.532667834	0.739216242
relA	0.15070201	0.249285292	0.545487136	0.74876966
yjhQ	0.150589898	0.311649013	0.62895124	0.807067525
caiB	0.150034477	0.336223016	0.655427504	0.822164258
dtd	0.149377597	0.355526299	0.674369257	0.832764793
eutR	0.149358963	0.502218059	0.766162189	0.885737903
mlrA	0.149088439	0.301546416	0.621014603	0.803279702
blc	0.1490805	0.24231519	0.538400314	0.744102043
tsgA	0.149005727	0.294932161	0.613404084	0.798432153
ydiU	0.148810634	0.363060073	0.681894843	0.83430303
flgG	0.148582204	0.289148166	0.607348599	0.795201383
gadB	0.147901899	0.676927083	0.827047234	0.914235948
yjgM	0.147596915	0.231446917	0.52365994	0.732253266
dauA	0.1469119	0.34872528	0.67354872	0.832452278
murA	0.146512245	0.213287965	0.492131737	0.705266533
arpA	0.146493483	0.29758731	0.622528062	0.8040357
ytfL	0.146009963	0.22870507	0.523200109	0.73186377
creD	0.145726373	0.294559978	0.620793802	0.803251468
dhaR	0.145458221	0.355141064	0.682115013	0.83430303
torZ	0.144780195	0.493207234	0.76910301	0.888374493
thiL	0.144588596	0.234024618	0.536684119	0.74249273
djlB	0.144315566	0.286986952	0.615059504	0.799075767
holA	0.143985916	0.247613054	0.560906444	0.76062456
macA	0.143901048	0.217229466	0.507690574	0.720343379
ybbY	0.143574652	0.403896946	0.72223491	0.859823749

fepC	0.143244152	0.417951269	0.731802108	0.865090808
ruvA	0.142811625	0.469563998	0.76102378	0.882831322
gspC	0.142472919	0.247797202	0.565319879	0.763787266
ygbJ	0.142314346	0.265437989	0.59185549	0.784081396
guaD	0.142109004	0.318728395	0.655696652	0.822164258
yqhC	0.141514175	0.23817782	0.55240948	0.752895703
yfjL	0.141109903	0.269606365	0.600701262	0.791794473
waaP	0.141068491	0.248288895	0.569924491	0.767952327
accA	0.140914699	0.418246468	0.736178832	0.867963206
rhtA	0.140851769	0.235292811	0.549424087	0.751363496
ygjV	0.140773654	0.251668045	0.575914384	0.773441905
betA	0.140496765	0.533108258	0.792132352	0.895647744
osmF	0.140232993	0.414419897	0.735073955	0.867963206
ptsP	0.140107127	0.230913016	0.544014952	0.747829602
wcaC	0.138663334	0.506094698	0.784094663	0.895175447
yjgR	0.138071644	0.31774208	0.663896353	0.827973301
fliO	0.137890292	0.487033446	0.777082675	0.891678156
phoB	0.137583031	0.262548851	0.600258848	0.791794473
adhP	0.136949846	0.229303749	0.550345775	0.751605512
yebT	0.13663686	0.326294726	0.675396736	0.833183796
kdsD	0.134273943	0.321544161	0.676246189	0.833335124
ybdN	0.134061156	0.249774623	0.591455176	0.784081396
panE	0.133601849	0.264069196	0.612902282	0.798156939
yjfJ	0.132918818	0.304237228	0.662189658	0.827378412
yghR	0.132238388	0.407215381	0.745379224	0.872707636
yigE	0.131219692	0.28983853	0.650740455	0.819157847
mepA	0.129873166	0.330519714	0.694366592	0.841789168
argG	0.129033568	0.371753509	0.728520387	0.8642326
yfcO	0.129007142	0.309488319	0.676795368	0.83375737
ybjD	0.128445836	0.30115373	0.669734428	0.830125234
ybaZ	0.128319561	0.442829798	0.77199087	0.890690809
ybbM	0.128189881	0.34720659	0.711976062	0.852639384
treC	0.12818064	0.270274778	0.635314184	0.810607889
hybB	0.127932655	0.682665609	0.851345736	0.9237455
speA	0.127740386	0.522977511	0.807032572	0.904626068
ybeR	0.127713424	0.283040292	0.651831039	0.820019291
narY	0.127375468	0.458235656	0.781035886	0.893736623
ynbC	0.127185515	0.3982237	0.749436795	0.876270584
insF1	0.127112653	0.887327655	0.886090103	0.944891297
yfiN	0.126895825	0.249972044	0.611705787	0.797627992
tyrB	0.126389408	0.228317449	0.579874009	0.774124131
fliM	0.125756185	0.442389076	0.77620652	0.891678156
yjhU	0.125233094	0.547920474	0.819210363	0.91057487
nadB	0.125173744	0.296743996	0.673153476	0.832452278
epmC	0.124572341	0.296300034	0.674174024	0.832764793
yggE	0.122828967	0.264772676	0.642716896	0.814447504
aroF	0.122740671	0.226913193	0.588566411	0.782690025
csiE	0.122301057	0.298456315	0.681968252	0.83430303
wzb	0.122281757	0.437361591	0.779792379	0.893055839

yhhZ	0.121409556	0.316372578	0.701160051	0.846373654
yjaH	0.120922851	0.238925512	0.612778665	0.798156939
yaaA	0.120748964	0.27289586	0.658147192	0.823602051
yhaK	0.120624548	0.270010059	0.655062118	0.822034744
ybhJ	0.120339157	0.347118909	0.728831796	0.8642326
agaR	0.119856651	0.445931753	0.788100512	0.895647744
nlpA	0.119631838	0.346599046	0.729974715	0.8643334
dcyD	0.119571883	0.347848707	0.731036705	0.864719669
ybiI	0.119560547	0.433375215	0.782638476	0.894538938
yeiH	0.119418808	0.24354012	0.623889416	0.8040357
truD	0.11861684	0.261969233	0.650700539	0.819157847
ycbF	0.118591943	0.295128446	0.687807946	0.838175989
motB	0.118513569	0.278108173	0.670004388	0.830204946
ybiR	0.117930195	0.351220519	0.737042422	0.867963206
proP	0.116741992	0.255763698	0.648070207	0.817581071
yihR	0.116636819	0.227064611	0.607481066	0.795201383
nagZ	0.116221395	0.250067281	0.642102978	0.814447504
ybaP	0.116036199	0.232659984	0.617964502	0.800934796
zitB	0.11481248	0.254797411	0.652275697	0.82032305
pabA	0.114059695	0.268944015	0.671491425	0.831537081
menD	0.113768619	0.564082444	0.840160701	0.919167683
sgcA	0.113693228	0.336176044	0.735215861	0.867963206
ghrB	0.113613733	0.261774908	0.664278945	0.828119138
uvrB	0.112725099	0.215844769	0.601495733	0.791794473
garD	0.112570501	0.266795375	0.673071931	0.832452278
ygjI	0.112468568	0.37666402	0.765251967	0.885192605
rarD	0.112411893	0.263438047	0.669589891	0.830125234
bglG	0.111812334	0.278302455	0.687856589	0.838175989
djlC	0.111584913	0.298313348	0.70836511	0.850679367
nuoF	0.111436097	0.226952524	0.623418945	0.8040357
ycaI	0.111383083	0.276304794	0.68686236	0.837723066
yccS	0.108991015	0.326049498	0.738170158	0.868802603
yegI	0.108946926	0.38770746	0.778708165	0.892573782
sgrS	0.108600584	0.723847538	0.880739053	0.942437059
clsB	0.10839864	0.559938737	0.84649679	0.922499874
ybaT	0.108055904	0.409349612	0.791803249	0.895647744
gspI	0.108042578	0.264226083	0.68261157	0.83430303
focB	0.107502144	0.348000348	0.757387025	0.88063693
rsmB	0.107312934	0.265656918	0.686247213	0.837225748
yqeB	0.105138358	0.213822961	0.622926119	0.8040357
kbaY	0.105106042	0.32086785	0.743238316	0.871971253
yidC	0.103996332	0.666804435	0.876062763	0.940102439
yfeO	0.103366216	0.395746209	0.793943527	0.896101284
coaA	0.103040539	0.325812864	0.751807585	0.877797051
ascB	0.102824804	0.324563863	0.751388414	0.877797051
yohK	0.102601694	0.369339822	0.781167781	0.893736623
tynA	0.102267772	0.281178621	0.716073789	0.854561594
mhpD	0.102010504	0.434863581	0.814534367	0.908755262
ytfQ	0.101904086	0.247662268	0.680732397	0.83430303

dcuC	0.101314112	0.217020321	0.640613217	0.813768269
ydiJ	0.100943354	0.442437579	0.819527487	0.91057487
corA	0.100890287	0.44808518	0.821855799	0.912006956
xdhA	0.100887535	0.263018628	0.701293008	0.846373654
ybhD	0.100653746	0.232389697	0.664924093	0.828231584
lexA	0.100309375	0.55064978	0.855452872	0.926606719
topB	0.098827998	0.475874408	0.835481459	0.917030628
pphB	0.098765718	0.421323623	0.814660712	0.908755262
dtpB	0.098632242	0.466981261	0.83272152	0.916441401
hyfC	0.098592396	0.43473664	0.820589771	0.911089357
rihB	0.097943469	0.25512046	0.701044651	0.846373654
hisF	0.097874446	0.394672267	0.80414279	0.902258044
ddpD	0.097304589	0.347528947	0.779485267	0.893055839
ycaQ	0.096974754	0.399033188	0.807986588	0.905204389
iaaA	0.09629934	0.445268398	0.828775291	0.915644732
tas	0.096178724	0.240015342	0.68862697	0.838497695
dut	0.095958959	0.339050183	0.777159299	0.891678156
yeeR	0.095917882	0.372377049	0.79672899	0.8979327
ytfI	0.095534939	0.287264944	0.739460899	0.869054919
kdgT	0.095190017	0.31933081	0.76563281	0.885379448
narK	0.094998682	0.324130695	0.769455209	0.888527082
helD	0.094622813	0.415793605	0.819979306	0.910662024
ttdB	0.094539708	0.300681293	0.753203341	0.878302105
ansP	0.09389596	0.354050751	0.790851695	0.895647744
csiR	0.093332532	0.419863817	0.824086275	0.913021024
oppF	0.093020237	0.213364607	0.662859619	0.827447211
nusA	0.092938672	0.406065551	0.81896527	0.91057487
htpG	0.092553995	0.205545879	0.652505766	0.820356828
coaE	0.092494982	0.299284102	0.757280366	0.88063693
yieH	0.092312171	0.452376271	0.838306146	0.918386526
rpoD	0.092163988	0.375640932	0.80618444	0.904297468
wzxC	0.09201817	0.524563251	0.860750698	0.929601543
dhaL	0.091780036	0.378409761	0.808360676	0.905204389
yihY	0.091380884	0.270080128	0.735101479	0.867963206
prmA	0.09088132	0.219276907	0.678537879	0.834122235
secA	0.090830834	0.339421846	0.789003721	0.895647744
pldB	0.08999035	0.430966976	0.834596319	0.916822141
mdtK	0.089662274	0.344177268	0.794468937	0.896136099
purN	0.089524812	0.288698551	0.756486515	0.880096848
idnO	0.089085857	0.289559545	0.758341146	0.880985096
flgE	0.088758638	0.271881723	0.744075986	0.872446923
yhjH	0.088071458	0.262501874	0.737241971	0.867963206
ydbK	0.086281831	0.214195754	0.687082372	0.837738307
pcm	0.086249194	0.268609161	0.748138018	0.875429906
betT	0.086200786	0.437952877	0.843963216	0.921063116
aes	0.086178766	0.255903489	0.736295747	0.867963206
mtlR	0.086071506	0.239400316	0.71919892	0.85722056
rluF	0.085121644	0.226853835	0.707491781	0.850549232
modA	0.085084443	0.224727759	0.704976615	0.848789322

yfcR	0.084593078	0.425352646	0.842358556	0.920325167
pheP	0.084176086	0.317334007	0.790809013	0.895647744
rutG	0.084133783	0.386006296	0.827460578	0.914442473
yehT	0.083540629	0.229590514	0.71595662	0.854561594
yqeG	0.083438062	0.416474357	0.841211696	0.919904188
alaS	0.082158975	0.383584474	0.830400894	0.916159834
wecH	0.082122334	0.219750015	0.70862159	0.850679367
yecC	0.08181891	0.432372357	0.849910583	0.923651849
gfcC	0.081301137	0.369094572	0.825659457	0.913772671
lptB	0.081230573	0.32693729	0.803779144	0.902100752
csiD	0.081181111	0.220581147	0.712848729	0.853178274
uidB	0.081043544	0.398868964	0.838991703	0.918386526
fpr	0.079342442	0.278091522	0.775406241	0.891678156
yihI	0.079320083	0.331769792	0.811042237	0.906472179
wrbA	0.078977119	0.346447617	0.819675273	0.91057487
yjjU	0.077774626	0.379321606	0.837543914	0.918054792
tusD	0.07747673	0.375541578	0.836551374	0.917955395
agaI	0.077117961	0.217627871	0.723071349	0.860565754
cvrA	0.07692568	0.347355772	0.824733673	0.91342931
hyfE	0.076667791	0.406653467	0.850458478	0.923675242
nrdD	0.076298453	0.347081983	0.826004854	0.913834364
yhaO	0.075344289	0.24988715	0.763023142	0.883373796
yqiI	0.075185902	0.263337346	0.775252118	0.891678156
ybhN	0.074550663	0.281564667	0.791184498	0.895647744
ompN	0.07445224	0.293694263	0.799879903	0.899440462
lpoB	0.07424861	0.272660571	0.785382512	0.89554997
dinD	0.074215761	0.440017765	0.866059856	0.932342304
yadM	0.074183834	0.317995716	0.815539608	0.908982164
guaB	0.074150741	0.306428523	0.80879271	0.905204389
sgcR	0.07387664	0.244810252	0.762826623	0.883373796
yfgF	0.073834211	0.336681712	0.826416566	0.914039363
appB	0.073722593	0.368163522	0.841289636	0.919904188
rlmF	0.072692202	0.354184615	0.837386048	0.918054792
dhaK	0.072533181	0.317452272	0.819268974	0.91057487
ybiU	0.072487988	0.342117472	0.832200341	0.916298342
fecE	0.071899673	0.269861425	0.789906657	0.895647744
feaB	0.071003168	0.322462346	0.82572274	0.913772671
ydcS	0.0707503	0.374460155	0.850140265	0.923651849
mhpT	0.068992253	0.688825399	0.920217876	0.958194368
idnR	0.068740471	0.25623467	0.788490307	0.895647744
ydjE	0.068631175	0.322977305	0.831720607	0.916298342
insE1	0.066993629	1.773333087	0.969864392	0.982766705
yfcC	0.066726616	0.310437218	0.829810982	0.916159834
yjdC	0.066155107	0.356263778	0.852686801	0.924602905
cdsA	0.06595925	0.226993034	0.771373859	0.890318484
ampE	0.065260176	0.337410497	0.846634143	0.922499874
yehL	0.064772146	0.532312042	0.903151833	0.952158378
rtn	0.06462029	0.22245895	0.771447624	0.890318484
sufD	0.06454335	0.430440606	0.880806298	0.942437059

galM	0.064473951	0.215454961	0.764752653	0.884868575
sbp	0.064335118	0.243958692	0.792000953	0.895647744
acrB	0.064113141	0.342332233	0.851438446	0.9237455
yddB	0.064017524	0.338257316	0.849891489	0.923651849
asmA	0.063814096	0.31037565	0.837101208	0.918054792
proY	0.062835497	0.22736296	0.782266611	0.894367123
insA	0.062438756	0.785824926	0.936669674	0.966050976
patD	0.061823759	0.497910474	0.901183512	0.952158378
purF	0.061634946	0.420819599	0.883555061	0.943877159
hslO	0.061619914	0.255616349	0.809505471	0.905506674
yggS	0.060925546	0.358369552	0.865004163	0.93191002
pncA	0.06058783	0.382025099	0.873986815	0.938696108
yjjX	0.060533957	0.290346684	0.834847528	0.916822141
obgE	0.060242063	0.313128677	0.847438634	0.923127298
rnd	0.060112734	0.377543298	0.873494941	0.938696108
livK	0.060094363	0.27990153	0.830002602	0.916159834
ydhQ	0.05993889	0.256536277	0.815259164	0.908982164
frmB	0.059707566	0.254611386	0.814593187	0.908755262
pnuC	0.059654238	0.247863983	0.809808349	0.905594544
yjhB	0.059369063	0.251876719	0.813660169	0.908392174
ebgR	0.05930717	0.256617329	0.817228321	0.909859551
prfA	0.059298896	0.343577262	0.862971662	0.93075517
yiiM	0.05807057	0.219732963	0.791565774	0.895647744
yehA	0.057947891	0.29046177	0.841869577	0.920272516
hemB	0.057658537	0.50638551	0.909346454	0.954506405
malE	0.057616939	0.224465757	0.797422136	0.898021783
yabI	0.057526827	0.379109304	0.879390381	0.94183835
argA	0.057452611	0.214103382	0.788437225	0.895647744
dmsD	0.057273568	0.34231922	0.867125815	0.932992248
waaU	0.057016648	0.311737407	0.854876794	0.926231244
yrbG	0.056902588	0.227218908	0.802254298	0.901140957
alaC	0.056488219	0.271128027	0.834959453	0.916822141
yiiX	0.056147599	0.262610645	0.830698738	0.916159834
scpC	0.056136778	0.438749566	0.898190759	0.950706895
sfmF	0.055859896	0.240743266	0.816513952	0.909589544
nuoC	0.055391763	0.313891425	0.85992636	0.92901035
yebS	0.054817784	0.278890996	0.844174766	0.921063116
ubiB	0.05469452	0.269080824	0.838928472	0.918386526
truA	0.054577588	0.237117017	0.817958479	0.910170446
rsmG	0.054571011	0.542697627	0.919903645	0.958114297
acrE	0.053992481	0.22087714	0.806885604	0.904626068
ybgF	0.053915327	0.22634852	0.811729029	0.906876628
ybgD	0.053752392	0.435065774	0.901671556	0.952158378
cysM	0.052996765	0.458390799	0.907957826	0.954423114
ycaM	0.052795769	0.287705449	0.854400759	0.925963991
astA	0.052645238	0.675657726	0.937894059	0.966050976
yhcM	0.052500834	0.220558637	0.81185339	0.906876628
ygaZ	0.052092753	0.256150859	0.838847822	0.918386526
yceJ	0.05152009	0.246578358	0.834495052	0.916822141

lsrD	0.050749392	0.440161507	0.908209553	0.954423114
ushA	0.050596988	0.269085693	0.850850788	0.9237455
sdaA	0.05046844	0.446448449	0.909995456	0.954691179
surA	0.050425058	0.268481217	0.851020922	0.9237455
pmbA	0.050291459	0.282950376	0.858927562	0.92862629
nanS	0.050262841	0.217949829	0.817612698	0.910036521
vsr	0.049597136	0.638117366	0.938047476	0.966050976
ttdR	0.049034985	0.259646509	0.850208143	0.923651849
lysU	0.048628253	0.399891802	0.903213022	0.952158378
tamA	0.047574813	0.340331216	0.888826118	0.94547321
nuoG	0.047565294	0.419228519	0.909666581	0.954594226
dsbE	0.045865549	0.292650597	0.875461895	0.939707436
rfbD	0.045510514	0.268471692	0.865390007	0.93191002
yeeA	0.045087336	0.268575925	0.86668117	0.932762433
ygjP	0.044327127	0.370089572	0.90466214	0.952765468
dkgA	0.043425136	0.227899626	0.848882076	0.923651849
scpB	0.043313911	0.376132286	0.908321428	0.954423114
yfbL	0.043153856	0.23835007	0.856326432	0.926929539
yjtD	0.042926231	0.246637906	0.861829661	0.930269076
yfeR	0.042760383	0.34458815	0.901243006	0.952158378
pppA	0.042590691	0.235427249	0.856439728	0.926929539
pyrC	0.042433163	0.384596787	0.91214643	0.95512832
acpH	0.042179033	0.346849166	0.903210849	0.952158378
sgcE	0.041840454	0.331466808	0.8995513	0.951647956
modC	0.041774563	0.388363573	0.914340282	0.956094797
slp	0.04130293	0.438904289	0.925026036	0.960955945
ddlB	0.041101852	0.447713119	0.926853772	0.961433778
zapD	0.040776076	0.274705538	0.881999023	0.943463184
fliH	0.040759492	0.616926303	0.947323165	0.970399294
yrfG	0.040743273	0.346687914	0.906446832	0.953446029
fhuE	0.04068854	0.268609255	0.879598232	0.94183835
murJ	0.040524953	0.51737562	0.937567213	0.966050976
yjbM	0.040390529	0.39641472	0.918844321	0.957803931
yghS	0.040366534	0.217106547	0.852499968	0.924602905
asd	0.040347444	0.452300813	0.92891908	0.962618358
gfcE	0.040213785	0.429990106	0.925488411	0.960955945
yqjI	0.039987108	0.286365135	0.888946972	0.94547321
ydbH	0.039965174	0.423040139	0.924734738	0.960914836
ydbD	0.039701021	0.332973706	0.905091793	0.952765468
yiaM	0.038928093	0.31918546	0.902930116	0.952158378
napH	0.038836275	0.4531433	0.931701579	0.964022656
xerD	0.037485796	0.333192269	0.910423006	0.954771134
envY	0.036683161	0.311579417	0.906279265	0.953446029
ybiT	0.036297284	0.247510244	0.883408585	0.943877159
flgK	0.03581988	0.340193882	0.916143677	0.956676381
yfaA	0.035200669	0.42104636	0.933372213	0.965169986
narH	0.034715812	0.389597926	0.928997058	0.962618358
ycjM	0.033103969	0.336629154	0.921662651	0.959259236
yebQ	0.032709598	0.317971906	0.918066577	0.957544554

pka	0.032053608	0.200493271	0.872980538	0.93854126
rcsC	0.031957911	0.267561141	0.904925564	0.952765468
ydeP	0.031761782	0.260397084	0.902919281	0.952158378
yejE	0.031399518	0.383004856	0.934660998	0.966007795
dnaB	0.031173754	0.224493435	0.889558683	0.94547321
clpB	0.030933496	0.271502237	0.909289645	0.954506405
ybjS	0.030689693	0.301902127	0.919030962	0.957803931
dtpD	0.030462929	0.248742159	0.902528468	0.952158378
nagB	0.03024977	0.388087498	0.937871209	0.966050976
lptF	0.029387201	0.365634395	0.935940453	0.966050976
coaD	0.028340064	0.337900759	0.933159022	0.965169986
chpB	0.027633273	0.253092842	0.913057853	0.955229439
djlA	0.026629141	0.23223663	0.908711586	0.954506405
tam	0.025954626	0.342598149	0.939611456	0.966915995
purM	0.02558912	0.223737589	0.908943601	0.954506405
hmp	0.025260123	0.324864148	0.938022181	0.966050976
yliF	0.025157688	0.308872321	0.93508399	0.966050976
ybgP	0.02498372	0.317616946	0.937303134	0.966050976
yfeA	0.024414563	0.294224836	0.933868021	0.965435391
ybhC	0.02433021	0.248709913	0.922070735	0.959259236
ydjK	0.023910802	0.36362112	0.947570924	0.970406854
yhdX	0.023882644	0.31024555	0.938639605	0.966408591
insC1	0.023355548	0.982117448	0.981027448	0.989359932
feaR	0.022691392	0.305723774	0.940833841	0.966941501
ydiO	0.022384971	0.439575532	0.959386034	0.976831106
apbE	0.02214823	0.480977974	0.963271732	0.979035242
yieE	0.022132171	0.400908344	0.955975095	0.975795564
fsaA	0.022127554	0.241313483	0.926939337	0.961433778
serA	0.021868854	0.291664892	0.940231116	0.966941501
xdhD	0.021820704	0.351222027	0.95046094	0.971641128
yeiB	0.021498073	0.496009032	0.965428832	0.979747661
wcaM	0.020950062	0.38731053	0.956862564	0.975961135
dpiA	0.020213221	0.302432806	0.946712733	0.970020128
ypfH	0.020066664	0.390573484	0.959024765	0.976831106
degQ	0.020021569	0.233198668	0.931580669	0.964022656
tsaE	0.019938301	0.34467552	0.953870833	0.974123906
era	0.019923215	0.256355099	0.938052967	0.966050976
dxr	0.019869954	0.255147493	0.937926417	0.966050976
envR	0.01976696	0.287019232	0.945093249	0.969657339
ybhS	0.018628577	0.415303726	0.964222638	0.979262086
ydjJ	0.018114278	0.360651257	0.959941834	0.977126874
nagC	0.017204132	0.254502141	0.94610472	0.970020128
rsmF	0.017185825	0.315851402	0.956607629	0.975947688
yafT	0.016413836	0.261667388	0.949983211	0.971641128
rlmG	0.015865893	0.231792261	0.945428406	0.969688637
wzc	0.015641428	0.4107528	0.969623973	0.982766705
hflC	0.015318742	0.636709474	0.980805361	0.989359932
mtr	0.01489104	0.238720019	0.950261273	0.971641128
queF	0.014539383	0.299789606	0.961318863	0.978035345

yqeC	0.014490973	0.411191666	0.971887244	0.983503167
ylaB	0.014354382	0.261664398	0.956251604	0.975831073
prpR	0.013640065	0.562672667	0.980659926	0.989359932
ysaA	0.013612854	0.681413182	0.984061412	0.9901934
eutP	0.013539198	0.304235353	0.964503952	0.979301422
tdcC	0.013412905	0.255491887	0.958131601	0.976761938
ssuE	0.012799428	0.447345783	0.977174094	0.986953169
yfeW	0.012440282	0.39168402	0.974662637	0.985649565
ypfJ	0.011828138	0.227754519	0.958581512	0.976831106
yadN	0.011753956	0.336576303	0.972141847	0.983503167
glxR	0.011738236	0.267598682	0.965011955	0.979570848
astE	0.010892388	0.470763603	0.981540432	0.989623746
lsrK	0.010486487	0.416719628	0.979923857	0.989235461
yddG	0.009025321	0.305287603	0.976415303	0.986680495
cysH	0.008572676	0.225322135	0.969650759	0.982766705
metF	0.008450606	0.235651832	0.971393547	0.983328924
yncD	0.007027035	0.288587225	0.980573607	0.989359932
mltB	0.006287147	0.275290541	0.981779317	0.989623746
yfdE	0.006196996	0.331250007	0.985074117	0.990965415
gshA	0.006078818	0.236678829	0.979509524	0.989064519
bdcR	0.006063242	0.287895049	0.983197316	0.9901934
yhjV	0.005440448	0.301266424	0.98559211	0.991157071
ygdB	0.004234439	0.407670725	0.991712593	0.995658726
elfD	0.003595129	0.248323411	0.988448944	0.993370273
yfjD	0.003217524	0.307254945	0.991644835	0.995658726
menF	0.00264957	0.4502635	0.995304886	0.997281167
yebK	0.002389324	0.379676489	0.994978903	0.997248962
yafZ	0.001602833	0.224729997	0.994309329	0.997248962
pgi	0.001501035	0.408318598	0.997066875	0.998055783
phoR	0.001224682	0.277536891	0.996479199	0.997772852
yhhX	0.001013098	0.233424405	0.996537067	0.997772852
plaP	0.000812591	0.244990363	0.997353559	0.99809527
lysS	0.000552912	0.473982079	0.999069247	0.999069247
yjhI	-0.000594501	0.249210844	0.998096622	0.998591341
ydiQ	-0.000774784	0.46829292	0.998679913	0.998927356
asnB	-0.00155142	0.248540608	0.995019543	0.997248962
prmB	-0.002456626	0.316079365	0.993798758	0.997248962
lsrA	-0.002550444	0.496495299	0.995901369	0.997631223
oxc	-0.002621598	0.420506575	0.995025717	0.997248962
argO	-0.003626358	0.36316307	0.992032872	0.995732646
yjbQ	-0.00373932	0.506374838	0.994108082	0.997248962
der	-0.00474842	0.265964764	0.98575567	0.991157071
iap	-0.005889797	0.2867923	0.983615155	0.9901934
yafE	-0.006729564	0.419589894	0.987203732	0.992365903
yicC	-0.006872929	0.309628915	0.982290564	0.989850093
pqiA	-0.007838977	0.392113036	0.984050052	0.9901934
dnaK	-0.009232095	0.420750834	0.982494259	0.989850093
chiP	-0.009252996	0.304700904	0.975773987	0.986519241
intD	-0.009324243	0.225102344	0.966959283	0.980561322

insC1	-0.009726196	0.925738514	0.991617245	0.995658726
dcuR	-0.009921655	0.27081609	0.970775129	0.983195985
potE	-0.010107308	0.218647314	0.963129693	0.979035242
cpxR	-0.01050873	0.522356477	0.983949299	0.9901934
mukF	-0.011077223	0.243117859	0.963658415	0.97903889
modF	-0.011314709	0.392297212	0.976990455	0.986953169
fadJ	-0.011904049	0.395880081	0.976011357	0.986519241
pitB	-0.012155778	0.350027005	0.972296542	0.983503167
insC1	-0.012614501	0.921095454	0.989073227	0.993750278
sapD	-0.012970073	0.254840502	0.959409298	0.976831106
wbbH	-0.013455725	0.28013902	0.96169049	0.97816692
rsxD	-0.013556483	0.375334652	0.97118796	0.983328924
fieF	-0.014337078	0.3155494	0.963760354	0.97903889
nudF	-0.014720401	0.354840818	0.966909634	0.980561322
yihG	-0.015510024	0.239877359	0.948446191	0.970635149
yqjE	-0.016226639	0.263074527	0.950817055	0.971759101
hcr	-0.016598377	0.349863691	0.962160628	0.978398604
nac	-0.016803804	0.225630709	0.940632586	0.966941501
alsB	-0.017036515	0.229176392	0.94074144	0.966941501
araH	-0.017525108	0.356881526	0.960834634	0.977789114
yfiC	-0.017557405	0.22363148	0.937422029	0.966050976
ydhK	-0.018943204	0.515131544	0.970665582	0.983195985
atoC	-0.01966899	0.308855308	0.949222236	0.971112561
fucR	-0.020142826	0.311945917	0.948515151	0.970635149
shiA	-0.021347146	0.310328603	0.945157698	0.969657339
tesB	-0.021733123	0.229808925	0.924656059	0.960914836
rsmH	-0.021830888	0.326570143	0.946701913	0.970020128
yghQ	-0.021866462	0.227852414	0.923546262	0.960174159
cheA	-0.022434325	0.534841856	0.966541971	0.980561322
umuC	-0.022576856	0.388029641	0.953602601	0.974112778
leuS	-0.022844949	0.389872784	0.953273971	0.974023543
trpC	-0.022862963	0.307685934	0.940766767	0.966941501
yadS	-0.022968519	0.247226345	0.925979177	0.961218292
yaiI	-0.023376702	0.317468754	0.941301096	0.967175496
yaiP	-0.023545609	0.294285307	0.93622973	0.966050976
ygiL	-0.023591324	0.351947379	0.946557161	0.970020128
ribF	-0.024567226	0.339312408	0.942281222	0.967689975
uidC	-0.024966437	0.282256794	0.929516703	0.962909656
fliS	-0.02505763	0.287073761	0.930443877	0.963406181
yghT	-0.025079634	0.24026209	0.916864235	0.956676381
yecD	-0.02581114	0.245563039	0.916288496	0.956676381
clcB	-0.025893922	0.506062506	0.959192099	0.976831106
ygiB	-0.026494488	0.406041558	0.947974411	0.970573851
yjcF	-0.026958443	0.308981518	0.930473297	0.963406181
yegH	-0.027240884	0.297138789	0.926954428	0.961433778
yeaX	-0.028782221	0.500005644	0.954096092	0.974123906
yhdZ	-0.029952775	0.271415435	0.912125723	0.95512832
yqeF	-0.030341891	0.257584319	0.906230871	0.953446029
yjgL	-0.031809775	0.348738424	0.927322669	0.961433778

metI	-0.03205791	0.232616972	0.890387302	0.94547321
srlR	-0.032202305	0.295122773	0.913111322	0.955229439
queG	-0.032560099	0.235873927	0.890208595	0.94547321
ybdK	-0.03292423	0.308671379	0.91505527	0.956271842
ybeQ	-0.03303811	0.235544543	0.888452446	0.94547321
bax	-0.033073993	0.35286748	0.925324259	0.960955945
yejF	-0.033242869	0.25815847	0.897540249	0.950516786
ybdO	-0.033707317	0.344486861	0.922053069	0.959259236
dkgB	-0.034094811	0.336443334	0.919281404	0.957803931
mlc	-0.034290879	0.246400671	0.889318066	0.94547321
yihX	-0.034619977	0.331225108	0.916755909	0.956676381
dctR	-0.034692821	0.330484679	0.916395186	0.956676381
fmt	-0.035400703	0.506079933	0.944232809	0.96920108
dgt	-0.036232614	0.280937368	0.897381044	0.950516786
cysC	-0.036379619	0.32571305	0.911067436	0.95483468
yejK	-0.036585473	0.25698112	0.886790614	0.944960793
yeiQ	-0.036603412	0.261884494	0.88884227	0.94547321
rfbB	-0.037109911	0.239580683	0.876903896	0.940505055
trpS	-0.038112187	0.370963711	0.918170663	0.957544554
yiiR	-0.038912632	0.29030182	0.893369375	0.947460535
ydcK	-0.038958851	0.31906469	0.90281721	0.952158378
narQ	-0.039095881	0.256084308	0.87866009	0.941638116
ogt	-0.039359912	0.358413611	0.912554498	0.955141952
uup	-0.039744822	0.392621541	0.9193684	0.957803931
nudK	-0.039928698	0.328608468	0.903288339	0.952158378
potH	-0.0401164	0.414931583	0.922978963	0.959831549
hslR	-0.04048171	0.29603603	0.891231656	0.94606947
paaK	-0.041424854	0.38543726	0.91441219	0.956094797
yoaE	-0.042023494	0.277845072	0.879780138	0.94183835
dhaM	-0.042479931	0.236650369	0.85754117	0.927743682
yehB	-0.043333655	0.313041325	0.889902244	0.94547321
prpB	-0.043671208	0.602714479	0.942237795	0.967689975
cirA	-0.043677415	0.338673369	0.8973844	0.950516786
gmr	-0.043962133	0.399177611	0.91230488	0.95512832
acrA	-0.0446569	0.48995558	0.927377541	0.961433778
ygbM	-0.045455836	0.313527044	0.884724936	0.944377199
pitA	-0.046044929	0.57264482	0.935913175	0.966050976
tufB	-0.046086472	0.604521658	0.939231133	0.966771056
torT	-0.046240083	0.323041354	0.886179753	0.944891297
pinR	-0.046702275	0.349533962	0.893708618	0.947460535
ybjI	-0.046922859	0.304185912	0.877406965	0.940794666
yciK	-0.047174111	0.263007092	0.857651578	0.927743682
ppdA	-0.047194428	0.331852699	0.886910192	0.944960793
ygeV	-0.047620601	0.330914535	0.885574665	0.944784599
treR	-0.048187247	0.226149929	0.831267255	0.916159834
yraI	-0.048227301	0.331128309	0.884201359	0.94421603
epmA	-0.048232657	0.325953091	0.882363108	0.943520951
amyA	-0.049175199	0.387378842	0.898985173	0.951298333
xapB	-0.049198012	0.355876834	0.890047193	0.94547321

ppx	-0.049439235	0.426398791	0.907695337	0.954423114
yqjG	-0.049578022	0.235311747	0.833128547	0.916441401
pldA	-0.049907765	0.40012609	0.900737231	0.952158378
ftsY	-0.050508182	0.421730796	0.904670073	0.952765468
ftsA	-0.051405405	0.526294637	0.922191007	0.959259236
yijF	-0.051897177	0.242315611	0.830413066	0.916159834
trmL	-0.0521845	0.406424797	0.897833295	0.950577764
yeeE	-0.052647154	0.468593084	0.910544678	0.954771134
ispB	-0.053051644	0.359724083	0.882754182	0.943520951
yhjD	-0.053865932	0.403594078	0.893825157	0.947460535
proA	-0.054141531	0.446769547	0.903544923	0.952158378
csdE	-0.054208317	0.44744524	0.903571649	0.952158378
glcG	-0.054685792	0.309995537	0.859973168	0.92901035
yccM	-0.054956058	0.349802683	0.875161404	0.939634731
ydcC	-0.055038757	0.331890332	0.868287456	0.933884791
ddpA	-0.05511755	0.400119149	0.890435755	0.94547321
ssuA	-0.055541432	0.399045684	0.88930345	0.94547321
carA	-0.055808862	0.222031583	0.801539261	0.900839086
ycfH	-0.05615365	0.326011079	0.863245232	0.930801549
nadK	-0.056398466	0.285529402	0.843418718	0.920984951
ygjR	-0.056435925	0.207206523	0.785340935	0.89554997
pdxK	-0.056522511	0.258479448	0.826904678	0.914235948
lolD	-0.056923622	0.531152706	0.914654135	0.95610014
insA	-0.057177653	0.801967623	0.943161613	0.968347771
sppA	-0.058206981	0.274911164	0.832317567	0.916298342
yiaF	-0.058480184	0.525360549	0.911367052	0.954889382
waaM	-0.058621831	0.410257705	0.88637685	0.944891297
panC	-0.058780291	0.2622672	0.822661064	0.91236111
efeB	-0.059002413	0.340115538	0.862276066	0.930502132
yhgN	-0.059502129	0.231444512	0.7971091	0.898021783
mrdB	-0.059825222	0.307127343	0.845557639	0.922073525
cusC	-0.060193069	0.538985258	0.911078322	0.95483468
plsY	-0.061116488	0.310815033	0.844114755	0.921063116
nhaB	-0.061269784	0.228387814	0.788490874	0.895647744
yehU	-0.061518583	0.265494084	0.816760489	0.909589544
yoaD	-0.061769818	0.454252225	0.891836046	0.946462176
rlmJ	-0.062451907	0.361575014	0.862870064	0.93075517
yedV	-0.062499135	0.32280141	0.846477497	0.922499874
yadD	-0.062638002	0.238095278	0.792489253	0.895647744
rutB	-0.062746241	0.592779903	0.915700712	0.956676381
rnr	-0.06275301	0.231775261	0.786583788	0.895647744
ypeA	-0.063220243	0.323538678	0.845077917	0.921799393
abgB	-0.063253106	0.448771975	0.887911738	0.94547321
ydhV	-0.063613029	0.363795585	0.861190219	0.929827471
ydiB	-0.063909096	0.303299669	0.833111455	0.916441401
phoH	-0.064071097	0.25299154	0.800072009	0.899440462
emrY	-0.065128414	0.268334925	0.808227621	0.905204389
yqhG	-0.065448812	0.247399667	0.791358541	0.895647744
rfbA	-0.065544083	0.241108505	0.78574139	0.89554997

ydeA	-0.066157771	0.334628521	0.843275785	0.920984951
eamA	-0.066359599	0.231050394	0.773952804	0.891678156
dmlR	-0.066439271	0.418954658	0.873997077	0.938696108
ydfI	-0.066685836	0.312998085	0.831283991	0.916159834
pqiB	-0.066749261	0.235937236	0.777244991	0.891678156
phoQ	-0.066835832	0.235148108	0.776235153	0.891678156
yraH	-0.066906989	0.268702074	0.803360284	0.901881386
yfdQ	-0.067179425	0.362822832	0.853105381	0.924808384
puuD	-0.0674813	0.235078866	0.774067808	0.891678156
fruK	-0.067870185	0.340661543	0.842082406	0.920272516
rluD	-0.06787968	0.326014277	0.835064407	0.916822141
yehI	-0.068389452	0.446158604	0.878173475	0.941366521
rodZ	-0.068503501	0.470912586	0.884340057	0.94421603
ynbD	-0.069259523	0.387324826	0.858082898	0.927961602
fumC	-0.070016525	0.268427372	0.7942161	0.896101284
gcvR	-0.070168056	0.475321995	0.882640972	0.943520951
galK	-0.070404701	0.244828415	0.773677808	0.891678156
kduD	-0.070588218	0.278155538	0.799671343	0.899440462
tfaR	-0.070655183	0.82541939	0.931785089	0.964022656
curA	-0.070766328	0.213227126	0.739978069	0.869225051
yfbS	-0.070843738	0.240381312	0.768212367	0.887734929
yliI	-0.071023491	0.532779702	0.893950254	0.947460535
pabC	-0.071038906	0.22821165	0.755583826	0.880061139
xapR	-0.071795038	0.318936224	0.821895398	0.912006956
gsiB	-0.072820989	0.479673906	0.879334011	0.94183835
yihL	-0.074049847	0.223556773	0.740466919	0.869225051
ycaN	-0.074657968	0.290932855	0.797475376	0.898021783
sbmA	-0.076161216	0.217865248	0.726654328	0.863370914
tatC	-0.076338725	0.529941268	0.885459965	0.944784599
dmsB	-0.076373217	0.284069365	0.788041949	0.895647744
ycaO	-0.077620524	0.214264968	0.717154619	0.855541724
fixX	-0.078021755	0.363413735	0.830008033	0.916159834
hydN	-0.079111884	0.254036709	0.755482035	0.880061139
lon	-0.080598945	0.259733244	0.756321941	0.880096848
ybjL	-0.081148515	0.234295367	0.729078688	0.8642326
yjfK	-0.08188395	0.283564121	0.772759715	0.891112203
lysP	-0.082155809	0.215859426	0.703501073	0.847265463
ypjD	-0.082239933	0.24684911	0.739014479	0.869036252
yhaC	-0.08225982	0.317775216	0.795741981	0.897321334
mltF	-0.082312776	0.319055383	0.796415405	0.897829933
gsiD	-0.082411473	0.454795279	0.856206162	0.926929539
mltC	-0.082520565	0.297733175	0.781655091	0.893921134
aroH	-0.082563993	0.395566903	0.834664321	0.916822141
yaeI	-0.083208059	0.256619443	0.745751118	0.872889899
ung	-0.083537579	0.262291955	0.750112659	0.876724031
ydgI	-0.083866718	0.415626452	0.840085685	0.919167683
ndh	-0.084107991	0.2770624	0.761455075	0.882955657
yejA	-0.084518934	0.444263697	0.849117173	0.923651849
sstT	-0.084557992	0.415663266	0.83879968	0.918386526

cbrC	-0.085035903	0.29741658	0.774943117	0.891678156
yciW	-0.085244072	0.305593964	0.780286622	0.893368432
uxaA	-0.085352693	0.55078612	0.876848736	0.940505055
yeiI	-0.085573885	0.321687007	0.790226901	0.895647744
fliL	-0.08609005	0.25867411	0.739275608	0.869054919
wecA	-0.0865166	0.305712471	0.777176911	0.891678156
lolC	-0.087649415	0.334300187	0.793176696	0.89593014
ompG	-0.08823634	0.344554301	0.797882623	0.898229825
rbsR	-0.08838996	0.467410195	0.850009928	0.923651849
araG	-0.088488596	0.520864125	0.86509817	0.93191002
yfcH	-0.088713323	0.237772193	0.709072398	0.850679367
yicS	-0.088772375	0.560041488	0.874054662	0.938696108
abgT	-0.089055391	0.397814963	0.822865197	0.91236111
ygcW	-0.089648948	0.241177462	0.710106081	0.850948683
tdcF	-0.089837751	0.668439147	0.893086838	0.947460535
leuO	-0.090147902	0.345258468	0.794013345	0.896101284
ybdG	-0.092739593	0.297216828	0.755020215	0.879912415
thiK	-0.092873213	0.391660206	0.812558261	0.907412918
ygfK	-0.09309773	0.372514831	0.802651658	0.901336507
ybeZ	-0.093377005	0.243691565	0.701588524	0.846477248
yeeO	-0.09341014	0.341614545	0.784517127	0.895175447
nagA	-0.093419473	0.230331043	0.685045456	0.836517999
thiB	-0.093709798	0.441607044	0.831949624	0.916298342
rlmC	-0.094456272	0.344015685	0.783646874	0.89493138
ugd	-0.094673757	0.252701979	0.707923842	0.850679367
hdfR	-0.094733464	0.300532406	0.752595532	0.877846912
yidX	-0.094960995	0.335025005	0.776836121	0.891678156
ydcJ	-0.095069251	0.345720696	0.783324994	0.89493138
aat	-0.09574947	0.33993778	0.778198375	0.892242783
yadH	-0.095907734	0.3321854	0.772797578	0.891112203
yfcP	-0.096197899	0.45134657	0.831221411	0.916159834
htpX	-0.096426117	0.218182971	0.65852488	0.823819318
pqqL	-0.096765847	0.438184005	0.82522183	0.913719289
yfcS	-0.096786287	0.287952137	0.736780862	0.867963206
prpC	-0.096872893	0.584740139	0.86841801	0.933884791
ansB	-0.096919092	0.512667806	0.850054785	0.923651849
thrA	-0.097516461	0.207977362	0.63915529	0.812939478
yjeJ	-0.098002829	0.268833442	0.715448461	0.854516402
yfbN	-0.098027859	0.346527684	0.777264164	0.891678156
uspD	-0.098857669	0.314923865	0.753589592	0.878498753
ispA	-0.099087056	0.294345507	0.736391924	0.867963206
csdL	-0.099402573	0.266217503	0.708859265	0.850679367
mlaE	-0.099758948	0.226647041	0.659827663	0.824937836
ygeH	-0.100147047	0.351840653	0.775921872	0.891678156
bamB	-0.100352153	0.323012209	0.756046915	0.880092675
trmA	-0.100368277	0.269138016	0.709204312	0.850679367
hdeD	-0.100413282	0.371939599	0.787181786	0.895647744
ftsW	-0.100674243	0.208610174	0.629383277	0.807067525
cheY	-0.100705622	0.332717896	0.762137019	0.883107677

cysD	-0.1010464	0.373741543	0.786879943	0.895647744
ddpB	-0.101057124	0.53302293	0.849628503	0.923651849
preA	-0.101154115	0.264875926	0.702541146	0.846867306
cycA	-0.101302895	0.291173097	0.727905549	0.864026669
pyrH	-0.102218401	0.603167407	0.865427462	0.93191002
ybdM	-0.102256661	0.338271468	0.762429697	0.88319331
ulaR	-0.102280384	0.22808636	0.653844105	0.821016688
argS	-0.102541153	0.26885722	0.702909196	0.847058037
ydeN	-0.102590752	0.366770337	0.779697032	0.893055839
hcaE	-0.102628539	0.362361501	0.777007109	0.891678156
yrdA	-0.103051016	0.606992047	0.86518846	0.93191002
ydhX	-0.103103483	0.425711144	0.808632031	0.905204389
talA	-0.103228942	0.2397412	0.66677022	0.828965058
fucP	-0.103403626	0.21968595	0.637863276	0.812063716
yciA	-0.103413487	0.298754238	0.729230726	0.8642326
rpiR	-0.10373425	0.262835647	0.693083904	0.841750819
yhcG	-0.103986205	0.283774329	0.714036934	0.854093957
yfcA	-0.10417079	0.430988365	0.809011007	0.905204389
cra	-0.104203261	0.272558311	0.702227375	0.846867306
manZ	-0.104344636	0.469545359	0.824138869	0.913021024
yfbP	-0.104430387	0.228119352	0.647104489	0.817244062
slmA	-0.104488762	0.389743762	0.788625388	0.895647744
yhjB	-0.104601428	0.277657313	0.706375368	0.849966427
yjaZ	-0.105001695	0.391127243	0.788345899	0.895647744
pgaA	-0.105194132	0.373305253	0.778103626	0.892242783
malY	-0.105365406	0.300080832	0.725495763	0.862688188
flgD	-0.10543031	0.313785079	0.736874414	0.867963206
ppsA	-0.105746736	0.233798239	0.651053515	0.819296459
yeaJ	-0.105930891	0.289823918	0.714737566	0.854382602
yphH	-0.106105198	0.332312202	0.749503798	0.876270584
ycgG	-0.106314304	0.257242029	0.679397727	0.83430303
otsB	-0.106500659	0.246586979	0.665814854	0.82839692
pfkA	-0.106758569	0.479263082	0.823725672	0.913021024
narL	-0.108004937	0.274473376	0.69395113	0.841789168
ynfF	-0.108477562	0.425397779	0.79872105	0.898923022
csgD	-0.108600073	0.271816129	0.689498997	0.839164139
abgR	-0.108882541	0.299417922	0.716121345	0.854561594
ybeU	-0.109181312	0.345739677	0.752161514	0.877797051
ynjD	-0.109626072	0.282411822	0.69788427	0.844278933
pdxB	-0.110196232	0.229806551	0.631570617	0.80838636
yhcH	-0.110364498	0.330767706	0.738634894	0.868977633
chbG	-0.110599534	0.302853789	0.714968697	0.854382602
rcsD	-0.111501014	0.239600679	0.641672353	0.81434495
ssnA	-0.112626996	0.337702053	0.738749378	0.868977633
opgC	-0.11304614	0.243722939	0.642769167	0.814447504
yaeQ	-0.113237263	0.328219535	0.730090882	0.8643334
yceA	-0.11360408	0.236708876	0.631276001	0.808265529
ecpD	-0.113650643	0.328002467	0.728972051	0.8642326
gadC	-0.11387533	0.402048558	0.77699459	0.891678156

yfcQ	-0.116049681	0.426684639	0.785637411	0.89554997
yddA	-0.116071819	0.339388641	0.732348887	0.865483741
pgaD	-0.116490662	0.292849092	0.690789465	0.839975021
intA	-0.117910207	0.245178125	0.630575841	0.807625212
purR	-0.117925805	0.239492474	0.622437454	0.8040357
tyrS	-0.118356181	0.342819001	0.72991099	0.8643334
ydiM	-0.118398228	0.216080522	0.583736068	0.777993564
ycfQ	-0.11840315	0.316196405	0.708061537	0.850679367
rsgA	-0.118610102	0.2867729	0.679164952	0.83430303
yadG	-0.118656378	0.288061791	0.680403239	0.83430303
yaeF	-0.118691513	0.251591087	0.637095548	0.81148902
ftsI	-0.118956365	0.446510924	0.789921068	0.895647744
yeiR	-0.11946719	0.454593777	0.792704827	0.895647744
mntH	-0.119527803	0.275527803	0.664423554	0.828119138
purC	-0.119721775	0.35671458	0.737154844	0.867963206
ybhI	-0.119969057	0.400370994	0.764448164	0.88476985
yahI	-0.120001838	0.67999069	0.859920232	0.92901035
mukE	-0.120920901	0.246623586	0.623917735	0.8040357
plsC	-0.121309805	0.453668507	0.789163157	0.895647744
sapB	-0.121487694	0.452162229	0.788174673	0.895647744
yfjH	-0.121555362	0.242955564	0.616850256	0.800200669
lolE	-0.121768531	0.219900795	0.579754727	0.774124131
yedA	-0.122047606	0.446364408	0.784525819	0.895175447
yfdC	-0.122259742	0.219485554	0.577507854	0.774124131
artM	-0.122821532	0.250024311	0.623257872	0.8040357
appY	-0.123043495	0.389919196	0.752335347	0.877797051
tus	-0.124362103	0.294474375	0.672792242	0.832452278
ygbI	-0.124505406	0.296499634	0.674545671	0.832764793
yddH	-0.124560093	0.334106563	0.709285794	0.850679367
rlmD	-0.124776153	0.216802798	0.564934034	0.763787266
pepN	-0.124876013	0.204778331	0.541987226	0.746758509
ydjY	-0.125234744	0.322023751	0.697350794	0.84420259
tag	-0.125643544	0.382767584	0.742722479	0.871619375
ygaH	-0.125914696	0.31434624	0.68874371	0.838497695
stfQ	-0.125964766	0.360448221	0.726738726	0.863370914
ybgO	-0.126155294	0.241535052	0.601457041	0.791794473
ygdQ	-0.126546701	0.29050258	0.663117534	0.827466829
uidA	-0.126811217	0.244879919	0.6045625	0.793697175
treA	-0.129056699	0.422797554	0.760179815	0.882286149
metK	-0.129370168	0.302395141	0.66878343	0.829674556
yfiE	-0.129546548	0.30758207	0.673625709	0.832452278
artJ	-0.130094127	0.255590141	0.610755664	0.797627992
paaY	-0.130233658	0.346429637	0.706968154	0.850172904
ampD	-0.130272452	0.351397779	0.710841286	0.851533019
yegK	-0.130434961	0.458286018	0.775939308	0.891678156
mhpR	-0.130787823	0.491793449	0.79028499	0.895647744
miaA	-0.130990517	0.427282805	0.759173493	0.881444748
galS	-0.131032949	0.26570002	0.621898669	0.8040357
ycdT	-0.131046537	0.281136591	0.641121592	0.814102776

nadR	-0.131173101	0.219474194	0.550060269	0.751605512
ydcV	-0.131556072	0.585076991	0.822093959	0.912006956
pyrF	-0.131572623	0.40407848	0.744718267	0.872707636
ppdD	-0.132382435	0.399319401	0.740251609	0.869225051
dosP	-0.132784966	0.420519524	0.752181575	0.877797051
pdxY	-0.133263291	0.311301918	0.668590152	0.829674556
pdhR	-0.133474267	0.294633452	0.650535755	0.819157847
psiE	-0.135297496	0.376092746	0.719037814	0.85722056
ytfB	-0.135435806	0.333504163	0.684669643	0.836312057
paaX	-0.135785323	0.441137921	0.758229224	0.880985096
chbC	-0.135852089	0.384580687	0.72390305	0.861211931
ycgB	-0.135991258	0.277736213	0.624387396	0.8040357
ydcI	-0.136781465	0.301257732	0.649803848	0.819157847
trkG	-0.137643173	0.265698783	0.604428851	0.793697175
ydhU	-0.138327727	0.327294444	0.672557931	0.832452278
ybeF	-0.138896499	0.323806565	0.667960348	0.829537807
yfeN	-0.138898697	0.258487276	0.591024379	0.784081396
yghB	-0.140362858	0.271012504	0.604513928	0.793697175
putP	-0.140499756	0.538379562	0.794117301	0.896101284
mreD	-0.14089355	0.258143955	0.585207207	0.779182551
metC	-0.141231367	0.217035784	0.515221773	0.725705969
ddpX	-0.141706543	0.318453138	0.656331335	0.822328959
ygfM	-0.143404336	0.537816363	0.789744951	0.895647744
ycfT	-0.143497112	0.302942607	0.635729866	0.810643346
degP	-0.143543662	0.259127764	0.579613495	0.774124131
hisP	-0.143758008	0.239795565	0.548837935	0.750936255
yafC	-0.144606487	0.219273404	0.5095874	0.721572898
ygjQ	-0.145088855	0.369195865	0.694329723	0.841789168
potB	-0.145114279	0.282488895	0.607462411	0.795201383
btuD	-0.14548125	0.27788705	0.600607875	0.791794473
amn	-0.145524995	0.212746177	0.493955546	0.706876477
anmK	-0.145951534	0.272399569	0.592097175	0.784081396
fadK	-0.146360155	0.470772117	0.755881321	0.880092675
sspB	-0.146409994	0.599783298	0.80714964	0.904626068
dsdC	-0.146703594	0.257076291	0.568228984	0.766435151
lptD	-0.146797362	0.478072939	0.75879725	0.881261363
ycaL	-0.147824349	0.296864095	0.618517211	0.801333113
murI	-0.148452925	0.45618211	0.744859666	0.872707636
yfbK	-0.149116288	0.390358413	0.702462554	0.846867306
yghU	-0.151778493	0.222104006	0.494375124	0.707226214
yfdR	-0.152301675	0.276964856	0.58239118	0.776713973
ydjZ	-0.152610033	0.63584395	0.810321131	0.905917032
yeaV	-0.152648809	0.410172314	0.709775395	0.850948683
waaQ	-0.153144958	0.218756535	0.483883387	0.699190852
frwB	-0.153145058	0.602308112	0.799291881	0.89931475
sgcB	-0.153214506	0.566850015	0.786936323	0.895647744
ytfJ	-0.153466213	0.37236421	0.680236758	0.83430303
ydhP	-0.153569862	0.306922243	0.616825631	0.800200669
sbcB	-0.153785945	0.374167979	0.681066841	0.83430303

ydcO	-0.1539511	0.42715659	0.718541262	0.85694271
srlD	-0.154330751	0.509115854	0.761787107	0.882955657
ulaC	-0.154540262	0.278071545	0.578376851	0.774124131
hyi	-0.154633555	0.338085784	0.647398194	0.817244062
xapA	-0.154697863	0.27345026	0.571580204	0.769669541
soxR	-0.155233234	0.436672249	0.722221478	0.859823749
rlmI	-0.155302883	0.394110732	0.693537477	0.841789168
nhaA	-0.155912354	0.291589097	0.59285812	0.784453697
dusA	-0.157327548	0.36656946	0.667785752	0.829537807
ydaN	-0.157650054	0.382649352	0.680342255	0.83430303
yhhA	-0.157862894	0.331078788	0.633494806	0.809822208
baeR	-0.158020946	0.352103048	0.653581884	0.820942771
rspA	-0.158356832	0.337098284	0.638523227	0.812647625
ygeA	-0.158412899	0.295222413	0.591552012	0.784081396
aceB	-0.158663556	0.367436157	0.665877633	0.82839692
gsk	-0.159083264	0.350679127	0.650085525	0.819157847
uvrC	-0.159418646	0.351794946	0.650435072	0.819157847
hrpA	-0.159444517	0.415260482	0.701006127	0.846373654
eamB	-0.159893718	0.306203474	0.601544129	0.791794473
yeaI	-0.160211263	0.305583034	0.600083373	0.791794473
yeaN	-0.160635137	0.545337799	0.768329413	0.887734929
ydaM	-0.161018451	0.333264735	0.628985838	0.807067525
flgA	-0.161058744	0.255184315	0.527944595	0.736204604
ebgC	-0.162797163	0.278150845	0.558356954	0.758376177
yhdN	-0.163131861	0.393282833	0.678291778	0.834073685
exbB	-0.164119672	0.448269074	0.714276536	0.854127481
citC	-0.165833471	0.290349645	0.567897336	0.766243831
hdeB	-0.166075635	0.348227642	0.633421322	0.809822208
ygaM	-0.166086985	0.501315465	0.740416126	0.869225051
ahpF	-0.166326086	0.278401112	0.550218149	0.751605512
dnaC	-0.167697384	0.226953003	0.459962878	0.677937254
yfjS	-0.167978352	0.526101851	0.749507636	0.876270584
sfmH	-0.168086209	0.355383693	0.636233813	0.810779838
yfaT	-0.168218577	0.298569707	0.573152495	0.770441983
gntK	-0.168420031	0.353059562	0.633340702	0.809822208
dosC	-0.168651717	0.360488306	0.639897292	0.813563576
torY	-0.169035099	0.268863308	0.529543683	0.737415608
hyaE	-0.169492952	0.437453748	0.698420561	0.844674597
rdgB	-0.169494671	0.405240086	0.675758921	0.833183796
ispU	-0.169998728	0.503589455	0.735684206	0.867963206
fabD	-0.170032307	0.406499027	0.675739005	0.833183796
yncE	-0.170149342	0.265856563	0.522169807	0.731438143
yeeZ	-0.170149676	0.347437811	0.624327095	0.8040357
leuD	-0.170644662	0.442027905	0.699459993	0.845678344
tdcA	-0.170871963	0.405764512	0.673673914	0.832452278
ymbA	-0.171035321	0.432882788	0.69276341	0.841750819
glsB	-0.17115586	0.359883171	0.634368435	0.810169369
ptrB	-0.171313531	0.355147077	0.629540658	0.807067525
tyrP	-0.171425508	0.392758494	0.662499069	0.827447211

ydbC	-0.172053043	0.262977946	0.512951435	0.723834183
ynfH	-0.172073949	0.230509252	0.455368593	0.674105128
ydeO	-0.17237287	0.336057264	0.608002334	0.795479779
glk	-0.172463004	0.28449774	0.544380847	0.748014118
ylbA	-0.172755504	0.255759049	0.499382324	0.711866682
ydcP	-0.172819744	0.450051366	0.700978416	0.846373654
pntB	-0.173023062	0.235512759	0.462543332	0.681243135
ydjM	-0.173370866	0.289942964	0.549874514	0.751605512
ybbW	-0.173667924	0.373956011	0.642355919	0.814447504
exoX	-0.173801303	0.250558062	0.487897464	0.7011811
pepT	-0.174275139	0.225790301	0.440206172	0.659872119
ydeQ	-0.174643063	0.303695489	0.565250864	0.763787266
ycbZ	-0.17470681	0.334004679	0.600927661	0.791794473
yahK	-0.175300924	0.290989897	0.54688822	0.74977246
moaB	-0.175453468	0.280757385	0.532017573	0.739186769
otsA	-0.175906463	0.458327385	0.70112602	0.846373654
hemG	-0.176028312	0.282832282	0.533693991	0.739560375
wzxB	-0.176173155	0.37356315	0.637210848	0.81148902
yncG	-0.176274704	0.269473299	0.513018171	0.723834183
yfgO	-0.176392555	0.29074914	0.544061318	0.747829602
recE	-0.17791435	0.517619971	0.731059755	0.864719669
yeaG	-0.178960936	0.292847789	0.541129645	0.74615003
yidB	-0.179074635	0.338918434	0.597241936	0.789219541
gatD	-0.180851677	0.434718571	0.677395273	0.833940919
ddpC	-0.180977267	0.434751388	0.677206878	0.833940919
ybcI	-0.181998022	0.392610148	0.642963635	0.814447504
cysB	-0.182279551	0.293740242	0.53489789	0.740529075
ycjP	-0.182915588	0.42338742	0.665720433	0.82839692
pgaB	-0.182942358	0.446922512	0.682291648	0.83430303
pgaC	-0.18317704	0.425460685	0.666804249	0.828965058
zntR	-0.183429549	0.447081924	0.681599286	0.83430303
bamC	-0.183838813	0.487177298	0.705909768	0.849659432
sulA	-0.184221618	0.474130829	0.697612049	0.84420259
yidL	-0.184269099	0.35553953	0.604262785	0.793697175
ydjL	-0.184475865	0.390057615	0.636252298	0.810779838
nhaR	-0.185482146	0.285146746	0.515382466	0.725705969
chaA	-0.185882981	0.317918076	0.558757318	0.758376177
sxy	-0.186042341	0.467123055	0.690429213	0.839975021
osmY	-0.186306465	0.31320432	0.55195041	0.752895703
efeO	-0.18663061	0.399098666	0.640049026	0.813563576
glpK	-0.187167676	0.429893142	0.663285276	0.827466829
pheS	-0.187542918	0.30842598	0.543144634	0.747491876
citD	-0.187884195	0.683730387	0.783475421	0.89493138
chbF	-0.188181267	0.324101861	0.561494001	0.760910132
mlaA	-0.18826588	0.288021525	0.513335959	0.723834183
yjfM	-0.18886246	0.384795661	0.62355918	0.8040357
diaA	-0.189034069	0.392127001	0.629753674	0.807084311
gcvT	-0.189337685	0.367148685	0.606065652	0.794797041
cynT	-0.189416335	0.583241954	0.745359245	0.872707636

rpoN	-0.189657912	0.543092595	0.726925374	0.863370914
ydjA	-0.189810087	0.316157423	0.548262511	0.750792319
ygcG	-0.189979874	0.351380087	0.588736625	0.782690025
lsrB	-0.190334039	0.521488755	0.715124799	0.854382602
ydhW	-0.19060513	0.375445216	0.611679453	0.797627992
proV	-0.191046038	0.244546596	0.434669875	0.65659441
araC	-0.191691056	0.216571031	0.376092648	0.610980289
yddW	-0.191751755	0.333263961	0.565037701	0.763787266
yjaG	-0.191875954	0.33666583	0.568725182	0.766592174
bluF	-0.191902579	0.266716782	0.471833239	0.6903603
yfjJ	-0.192227238	0.488763416	0.694103076	0.841789168
mntR	-0.193726262	0.246773442	0.432431924	0.655054902
puuP	-0.193963943	0.399385962	0.627211522	0.806386278
yneF	-0.194602384	0.281365962	0.48916694	0.702014553
agaB	-0.194613418	0.426197174	0.647938695	0.817581071
nemA	-0.194936102	0.403499848	0.629015363	0.807067525
srlB	-0.195312177	0.313343272	0.533076252	0.739307945
gltP	-0.196103854	0.455698074	0.666950337	0.828965058
wcaD	-0.196716444	0.473436932	0.67777066	0.833940919
nagK	-0.196890832	0.326886963	0.546960588	0.74977246
yfeS	-0.197251955	0.311180784	0.526157714	0.73422008
yniC	-0.198728501	0.283782925	0.483750273	0.699190852
pabB	-0.199288522	0.407362902	0.624688656	0.804167126
galT	-0.199427242	0.447394918	0.655776297	0.822164258
napC	-0.200096563	0.458788815	0.662734511	0.827447211
ldcA	-0.200167006	0.227348447	0.378620186	0.613112592
ygfI	-0.200370096	0.244844601	0.413153829	0.640088529
yfjZ	-0.201292509	0.454283083	0.657693922	0.823290036
metQ	-0.201351551	0.341265125	0.555180577	0.755906911
glnA	-0.201478093	0.725625388	0.781272366	0.893736623
gtrB	-0.201698989	0.272344833	0.458935105	0.677488686
rluC	-0.201710388	0.244698255	0.409756079	0.637655644
manY	-0.202468078	0.350739324	0.563763261	0.762705189
yicN	-0.203531467	0.440634608	0.644149224	0.814620978
insB1	-0.204302724	0.554781444	0.71268084	0.853178274
ydiY	-0.204391302	0.264016838	0.438836121	0.659079275
ydcR	-0.204505568	0.412841802	0.620345592	0.802928873
yrdD	-0.204523423	0.498094584	0.681357343	0.83430303
yqgA	-0.205428854	0.254423036	0.419418649	0.645529891
rfaH	-0.206988357	0.395784754	0.600986683	0.791794473
eda	-0.207372765	0.313145391	0.507826915	0.720343379
modE	-0.207601731	0.237812712	0.382683171	0.616969633
yadK	-0.207873515	0.26940536	0.440350629	0.659872119
dsbG	-0.208519968	0.23108382	0.366867564	0.602050552
malX	-0.208820721	0.438405128	0.633847084	0.810016042
dacD	-0.209505945	0.291393636	0.472153774	0.6903603
yhbP	-0.209556484	0.490103397	0.668959792	0.829674556
allS	-0.209664426	0.336539016	0.533283313	0.739307945
motA	-0.210223655	0.518903795	0.685381728	0.836607144

yccE	-0.211292937	0.29465708	0.473324237	0.691070505
ispE	-0.211870472	0.245177945	0.387505604	0.621270899
etp	-0.213123977	0.315957176	0.499971637	0.712203775
yeeS	-0.213756975	0.42762382	0.617165537	0.800352481
cobS	-0.216088863	0.36052619	0.548925659	0.750936255
gloB	-0.216643616	0.279572385	0.438392463	0.659079275
purB	-0.217075439	0.263475169	0.410000162	0.637655644
inaA	-0.217583016	0.22432161	0.332066371	0.569236492
ygeR	-0.218118201	0.393748027	0.57961049	0.774124131
yhaV	-0.218805444	0.306969037	0.475973731	0.693434122
cutA	-0.219004638	0.400645449	0.58463327	0.778675193
hisB	-0.219303657	0.279627808	0.432881791	0.655054902
mngR	-0.219334329	0.2412656	0.363297841	0.599779298
yedY	-0.219771659	0.489303465	0.653321967	0.820942771
katE	-0.220095169	0.42693591	0.606187786	0.794797041
ygaP	-0.220634899	0.37120404	0.552260429	0.752895703
moaA	-0.220877223	0.355634634	0.534547609	0.74029801
mltA	-0.2209613	0.457647461	0.629223622	0.807067525
atpD	-0.221421746	0.882517114	0.801892999	0.900985816
ybhM	-0.221732327	0.314286412	0.480492673	0.697500511
recT	-0.221815964	0.328769168	0.499875264	0.712203775
waaY	-0.221889623	0.534574764	0.678085627	0.834073685
ycgR	-0.222848647	0.277588321	0.422088796	0.647405953
kdsA	-0.223619035	0.240017337	0.351502609	0.587827685
ybfC	-0.223722296	0.30155521	0.458150967	0.676996872
mug	-0.223762998	0.276937156	0.419095205	0.645529891
yjcO	-0.223838549	0.226195802	0.322380082	0.56218073
yfcF	-0.223874493	0.237083423	0.345023655	0.580956219
rimO	-0.224255817	0.263672131	0.395041533	0.62720056
oppD	-0.224295246	0.45701495	0.623579814	0.8040357
yciQ	-0.224637904	0.403195993	0.577429597	0.774124131
mobB	-0.225543072	0.434594373	0.603779045	0.793697175
ppiD	-0.225707381	0.430369784	0.599965672	0.791794473
hisM	-0.226039128	0.31905318	0.478654366	0.695832797
gspB	-0.22606761	0.320796113	0.480991928	0.697974269
thrC	-0.226417127	0.280409019	0.419405493	0.645529891
yheO	-0.226965804	0.408345436	0.578335178	0.774124131
csgG	-0.227522849	0.436131554	0.601890374	0.791991994
ybiX	-0.227705913	0.270066585	0.399146065	0.630419665
setB	-0.228396387	0.460929198	0.620238082	0.802928873
yggC	-0.228785002	0.238982276	0.338400508	0.575937121
yfhH	-0.229029254	0.304325612	0.451702567	0.671516637
ynfL	-0.229039644	0.41128307	0.57760223	0.774124131
cho	-0.229594008	0.316401761	0.468059039	0.686860901
ydcZ	-0.230334916	0.408975573	0.573298914	0.770441983
iclR	-0.230415941	0.476963245	0.62903218	0.807067525
frsA	-0.230491506	0.28413485	0.417248167	0.644388237
usg	-0.230750489	0.229843669	0.315404943	0.554568708
ybfF	-0.230832583	0.371013617	0.533831789	0.739560375

ydfU	-0.23137916	0.380609556	0.543242279	0.747491876
cobC	-0.231653982	0.272010841	0.394416647	0.62720056
alaE	-0.231657085	0.382536315	0.544792396	0.748202572
rlmN	-0.232367273	0.230931487	0.31431102	0.553851413
maeB	-0.232390287	0.258876978	0.369352878	0.604255151
cbtA	-0.233701579	0.370687776	0.52839761	0.736327633
rnb	-0.233867195	0.364384462	0.520993699	0.730549344
dsbA	-0.235217862	0.463007209	0.611437895	0.797627992
ypdC	-0.235306981	0.526579184	0.654976412	0.822034744
nrdB	-0.235341222	0.317915871	0.459140684	0.677488686
fimZ	-0.235410568	0.335579107	0.482987643	0.699190852
tonB	-0.235930696	0.355563812	0.506984978	0.72016128
yqiH	-0.235976119	0.269980961	0.382092518	0.616756296
rcsF	-0.236868862	0.32817677	0.470434558	0.688801332
yfbM	-0.236981173	0.304772689	0.436824346	0.658788667
rnt	-0.237204929	0.35030969	0.498323968	0.71086002
hemA	-0.237486329	0.263842968	0.368064468	0.603034196
yciF	-0.238291652	0.279977266	0.39470785	0.62720056
bioD	-0.238469677	0.242972009	0.326361144	0.564974245
ymgG	-0.238843517	0.406361126	0.556692236	0.75694394
pepQ	-0.239310952	0.635992864	0.706709264	0.850114809
ghrA	-0.239433509	0.282358653	0.396451045	0.628375685
yhfY	-0.239527694	0.364501833	0.511092736	0.722437457
yibB	-0.239760212	0.237133176	0.311978949	0.551910174
ynjF	-0.240469223	0.695053324	0.729363504	0.8642326
yceF	-0.240626562	0.26610949	0.365868718	0.601144491
ascG	-0.240864876	0.252576702	0.340270651	0.577728528
fucA	-0.24152696	0.374719286	0.519216001	0.729069565
yqeJ	-0.241551556	0.375759321	0.520330629	0.730210744
ddlA	-0.242026854	0.434902769	0.577863652	0.774124131
alaA	-0.242044846	0.224208646	0.280341818	0.517916666
ydhS	-0.242270328	0.421579453	0.565512473	0.763791855
yjjB	-0.243231011	0.267391297	0.363010044	0.59962011
ybiV	-0.2433429	0.280066638	0.384915504	0.619293399
yciB	-0.243409027	0.25661404	0.342853934	0.579121896
ypdB	-0.243799362	0.34683586	0.482102912	0.699083856
ycjY	-0.243815635	0.259518512	0.347478062	0.584243621
ybfD	-0.244847459	0.263252235	0.352326086	0.588717057
yqiJ	-0.245002671	0.240960889	0.309261142	0.549026927
ppsR	-0.246269218	0.250257525	0.325084445	0.564246938
yegS	-0.246955074	0.221461664	0.264801434	0.502569997
ybiP	-0.247513735	0.361193643	0.493176061	0.706023965
gspG	-0.24788307	0.296028175	0.402388501	0.632682168
secF	-0.248120184	0.592156763	0.67520811	0.833183796
azoR	-0.24856755	0.396666307	0.530894292	0.738532135
yfeX	-0.249065924	0.290417376	0.3911058	0.623575875
rstB	-0.249234945	0.215114853	0.246613815	0.480724273
opgD	-0.249257177	0.336725336	0.459155077	0.677488686
gltL	-0.250157973	0.305512211	0.412891987	0.640088529

dmlA	-0.250610687	0.318655513	0.43159644	0.654774457
yeaW	-0.250732298	0.463093547	0.58821189	0.782666908
nagD	-0.251801359	0.293820995	0.391450967	0.623879808
yedW	-0.252452168	0.348855362	0.469275382	0.688395609
yiaC	-0.253173339	0.355764772	0.476693133	0.693981312
yedP	-0.253574161	0.437695414	0.562360243	0.761810347
tadA	-0.253889115	0.323008878	0.431859984	0.654819497
ybdR	-0.254366512	0.259332572	0.326666415	0.565260316
pyrD	-0.255039712	0.277347043	0.357798091	0.59417149
cbpA	-0.255118132	0.360838752	0.479557636	0.696832738
tpiA	-0.255170047	0.686560304	0.710142708	0.850948683
ydiF	-0.256235485	0.454657376	0.57304048	0.770441983
ynhG	-0.256242788	0.249010027	0.303458031	0.544411233
cytR	-0.256711933	0.392582458	0.513172519	0.723834183
dcp	-0.256876069	0.502073348	0.608909592	0.796296736
pheA	-0.258206993	0.218249871	0.23677756	0.468794022
fre	-0.259384297	0.459614847	0.572514985	0.770157613
aqpZ	-0.259432209	0.40708482	0.523934165	0.732383041
tolA	-0.259961673	0.273457541	0.341783449	0.57803929
ompT	-0.260071985	0.363157343	0.473904082	0.691666948
fliT	-0.26009756	0.327923293	0.427681197	0.651797764
agaV	-0.260174513	0.344186316	0.449702052	0.669659604
ygbL	-0.260715016	0.289821376	0.368348392	0.603254547
mzrA	-0.260960814	0.47024158	0.578928498	0.774124131
wcaN	-0.261056875	0.272814609	0.338616667	0.576005424
rmuC	-0.261425809	0.435859782	0.54864395	0.750936255
tolB	-0.261520138	0.455833577	0.566158334	0.764408426
xdhB	-0.261524192	0.314197115	0.405207423	0.63502421
sapF	-0.261822492	0.434478606	0.546766116	0.74977246
yqaB	-0.261845736	0.344887345	0.447720329	0.668187419
csgA	-0.262144243	0.363310795	0.470575693	0.688801332
quuQ	-0.262229729	0.263999516	0.320565605	0.560818235
insC1	-0.263019617	1.251284026	0.833511758	0.916613175
aceE	-0.263519133	0.568833321	0.643176238	0.814461253
ydcU	-0.263527964	0.636924229	0.679056556	0.83430303
ygfZ	-0.263542485	0.265583055	0.321043087	0.560818235
yqcC	-0.263545648	0.297127151	0.375090018	0.61029337
codA	-0.264542639	0.449888176	0.556519774	0.75694394
rimI	-0.264684261	0.361266991	0.463768549	0.682300887
solA	-0.265276439	0.283162426	0.348843498	0.585320532
gdhA	-0.265492893	0.248396762	0.285148125	0.523246809
napB	-0.265840291	0.506508263	0.599688254	0.791794473
yhdJ	-0.266300661	0.317357689	0.4014029	0.632252636
metG	-0.266662949	0.281443932	0.343393423	0.579305996
insE1	-0.267239323	0.766967173	0.727512428	0.863814021
hipA	-0.267495099	0.413205717	0.517395461	0.727525418
yafX	-0.268376225	0.316690691	0.396750152	0.628524184
zur	-0.269280368	0.525112756	0.608087837	0.795479779
flk	-0.269775806	0.349009009	0.439536215	0.659334251

yeiG	-0.26990589	0.39531357	0.494755956	0.707520296
yqaA	-0.270068673	0.250004995	0.280029484	0.517721866
yfjW	-0.27069891	0.326139008	0.406532659	0.635374504
rsxG	-0.272109719	0.457698653	0.552166246	0.752895703
nirD	-0.272293696	0.418809635	0.515588222	0.725742556
lspA	-0.272772274	0.373960312	0.465747824	0.684464494
ribB	-0.272986529	0.400530624	0.495515831	0.708356023
yfhG	-0.273378485	0.26913178	0.309734506	0.54940888
tdcB	-0.273433251	0.244104504	0.262650562	0.499915284
galU	-0.27382613	0.386120565	0.478217366	0.695447949
ycgN	-0.274066969	0.314087255	0.38289081	0.617057965
mglC	-0.274185384	0.268351092	0.306903374	0.546584028
fdnI	-0.27425756	0.276380405	0.321041879	0.560818235
yeiL	-0.274346485	0.313726588	0.381858889	0.616625733
asnS	-0.274553356	0.294199293	0.350705177	0.587344027
rsmE	-0.274881749	0.241430174	0.254888431	0.490927764
cpdA	-0.275101115	0.551668863	0.618011368	0.800934796
yceI	-0.275164173	0.419400813	0.511766994	0.722751135
ptsG	-0.275414858	0.3358007	0.412117169	0.640088529
insI1	-0.275635336	0.455266749	0.54488867	0.748202572
ppk	-0.275994882	0.240359926	0.250862632	0.485956068
insC1	-0.276089535	1.20680492	0.819042034	0.91057487
insC1	-0.278169822	1.200218187	0.816719731	0.909589544
yjfL	-0.278601771	0.30870454	0.366798332	0.602050552
potC	-0.278882972	0.420816933	0.507511156	0.720343379
zinT	-0.27937729	0.361532258	0.439665049	0.659334251
rssA	-0.279386225	0.239008601	0.242428629	0.475456948
ygcP	-0.279490904	0.24042755	0.245042763	0.478723175
fliC	-0.280019651	0.271204066	0.301835441	0.542197989
lsrF	-0.2800319	0.498535948	0.574314615	0.771550116
hisQ	-0.280272186	0.286388025	0.327755455	0.566174058
nadC	-0.280636404	0.250525688	0.262632748	0.499915284
yqiA	-0.282375969	0.443680958	0.524490176	0.732652886
creA	-0.282676878	0.439797246	0.520390466	0.730210744
ynjI	-0.283664612	0.403123372	0.481640179	0.698663817
mlaF	-0.283769054	0.643465585	0.659212174	0.824423652
fau	-0.283847738	0.393013774	0.470150893	0.688801332
ydjG	-0.283904797	0.263936813	0.282081844	0.518933632
nrdG	-0.284294664	0.410858989	0.488967528	0.70197792
yaeR	-0.284635844	0.287965854	0.322939118	0.562426756
yeiP	-0.285303799	0.401924709	0.477800948	0.695092766
leuE	-0.285339603	0.367241339	0.437169884	0.658788667
ftsQ	-0.285643586	0.3896352	0.463494277	0.682300887
mhpF	-0.28629249	0.551310605	0.603555483	0.793697175
ynfE	-0.287096849	0.380861735	0.450964162	0.671291416
yjgH	-0.287122606	0.384316878	0.455004038	0.673826596
ybjP	-0.287228349	0.281558634	0.307663544	0.546748622
potD	-0.287582754	0.225856947	0.202913013	0.42621149
ycbU	-0.288247177	0.345740105	0.404444104	0.634320454

yfiB	-0.288734963	0.302993913	0.340620566	0.577728528
syd	-0.289490785	0.339962755	0.394471248	0.62720056
aroA	-0.28958142	0.225714754	0.199508544	0.422568726
dnaQ	-0.289609357	0.48014658	0.546396512	0.74976299
flhE	-0.289721463	0.37379537	0.438292156	0.659079275
bfr	-0.289791042	0.304737891	0.341628852	0.578019981
cmoB	-0.289848028	0.319800032	0.364755148	0.600047487
yobB	-0.289941748	0.271136351	0.284908514	0.52304487
hpt	-0.290103587	0.526754222	0.581813265	0.776456248
wbbJ	-0.290353048	0.343804182	0.39837314	0.629934022
hyfJ	-0.29141778	0.299446963	0.330460555	0.567206318
fabH	-0.291738422	0.333488693	0.381678636	0.616581293
sufB	-0.292061285	0.516774194	0.571963386	0.76989234
mscS	-0.292312332	0.279265952	0.295230266	0.533966978
yegQ	-0.292344715	0.211504618	0.1669059	0.380892662
yhfU	-0.293258422	0.25840972	0.256434682	0.492631115
pyrI	-0.293668786	0.304304722	0.334520569	0.57198625
rnc	-0.29403112	0.244786749	0.229684281	0.461770639
rdgC	-0.294645058	0.24012561	0.219805475	0.44997703
ydgJ	-0.294682799	0.235519266	0.210859824	0.43788123
torD	-0.294818573	0.485887556	0.54400823	0.747829602
ygiC	-0.295005101	0.362853256	0.41620952	0.643384549
rhlB	-0.295179438	0.568544143	0.603631925	0.793697175
yqjA	-0.295279068	0.371114766	0.426232983	0.65104145
ftsL	-0.296581237	0.399421867	0.45776853	0.676679442
paaA	-0.296748787	0.382110842	0.437392475	0.658788667
dacC	-0.296830855	0.283322706	0.294787168	0.533657307
yfcD	-0.297892043	0.245214485	0.224433844	0.455818395
ybeX	-0.299284683	0.392688016	0.445974265	0.666320544
elaA	-0.299649145	0.249652767	0.230036955	0.462022383
nadD	-0.299695545	0.273888119	0.273855802	0.511270265
yafS	-0.300471196	0.230881928	0.193119417	0.414472668
hisS	-0.300623628	0.494535863	0.543260135	0.747491876
ecpE	-0.300767309	0.239599201	0.20937225	0.436588727
ygeY	-0.300912431	0.481882529	0.532330891	0.739186769
ygiW	-0.301559894	0.374046793	0.420122347	0.645633983
miaB	-0.301763027	0.272660524	0.268408387	0.505865854
yfjX	-0.301782126	0.467031948	0.518169138	0.728106095
ftnA	-0.301947268	0.280599944	0.281892626	0.518933632
gnd	-0.302428591	0.355150672	0.394463845	0.62720056
msbA	-0.302700669	0.380900915	0.42679	0.651399331
ydjO	-0.304084902	0.288599616	0.292040147	0.53151325
srlA	-0.304120411	0.435382511	0.484856335	0.700059021
fliN	-0.30429075	0.589344372	0.60563038	0.794582334
qmcA	-0.304636627	0.363859366	0.402459204	0.632682168
yfaP	-0.305563137	0.439078732	0.486479694	0.7011811
opgG	-0.305801278	0.222961429	0.170205622	0.384904479
cysK	-0.306115405	0.31420207	0.329926044	0.567012106
entD	-0.306436658	0.284927181	0.282154898	0.518933632

pepP	-0.306565376	0.305108796	0.3150057	0.55448771
asnC	-0.307301168	0.481047628	0.522942305	0.73186377
allR	-0.308027237	0.220892243	0.163176227	0.375351812
rluE	-0.309920272	0.327291227	0.343676956	0.579542135
yebA	-0.310210529	0.301396742	0.303365435	0.544411233
hybC	-0.311941895	0.63356313	0.622464032	0.8040357
ubiE	-0.312283739	0.618297071	0.613509175	0.798432153
fhuF	-0.31367279	0.305585873	0.30467309	0.544689032
yfdP	-0.315196659	0.398433828	0.428891999	0.652387716
wbbI	-0.316049417	0.260423234	0.224900829	0.456213346
yfiP	-0.316731678	0.33244548	0.340725523	0.577728528
cbpM	-0.316938298	0.479180467	0.508344671	0.720571431
insA	-0.317152168	0.519109961	0.541230211	0.74615003
ycfZ	-0.318930852	0.416085722	0.443377214	0.663176663
insQ	-0.319535586	0.382518228	0.403522167	0.633612987
casB	-0.319950345	0.256044255	0.211449352	0.438428881
pspA	-0.320608395	0.305396326	0.293805058	0.53235683
ansA	-0.32111145	0.334139656	0.336547291	0.573992992
apaG	-0.321396299	0.427290525	0.451947164	0.671516637
fkpB	-0.321621407	0.304968639	0.291606083	0.531233645
ygiV	-0.323983063	0.288136603	0.260840037	0.49787765
speD	-0.323993748	0.482470791	0.501882772	0.714170163
rlmA	-0.32413766	0.244585334	0.185086964	0.406981289
yccT	-0.324430466	0.351921556	0.356589657	0.592896394
cmtB	-0.325859151	0.415321614	0.43269122	0.655054902
agaD	-0.325868441	0.507440252	0.520755729	0.73046938
ycbX	-0.32587012	0.393169638	0.407201575	0.635841683
artP	-0.32630291	0.225085746	0.147147026	0.352332469
elfA	-0.326353559	0.29707362	0.2719595	0.509169165
sfmC	-0.326624365	0.249543455	0.190572386	0.411983443
bamA	-0.327119921	0.617190949	0.596102138	0.787971293
ycgM	-0.328509414	0.2810941	0.242532065	0.475456948
prfB	-0.329457744	0.47504762	0.487979689	0.7011811
idnK	-0.32965261	0.359660323	0.359369836	0.595068099
moaE	-0.329700894	0.473989563	0.48668762	0.7011811
yghD	-0.330012368	0.338972546	0.330271748	0.567206318
lgt	-0.33011466	0.213607768	0.122243639	0.311158619
lsrC	-0.330415615	0.596658109	0.579731382	0.774124131
ydhT	-0.330786309	0.454032443	0.466275662	0.684741669
fadD	-0.33172063	0.293844375	0.25894043	0.495301654
glnS	-0.332190307	0.337960986	0.325644346	0.564483964
yaiV	-0.332255049	0.263464107	0.207272187	0.433446977
hchA	-0.332276103	0.254188256	0.191143173	0.411983443
hldD	-0.332490873	0.442264738	0.452176451	0.67161013
mnaT	-0.333168893	0.301837347	0.269678713	0.507312659
sfmA	-0.333781227	0.387052312	0.388485472	0.621854024
yfcI	-0.335391865	0.266118224	0.207557005	0.4336996
nfsB	-0.335736146	0.261150264	0.19858087	0.422057811
glnQ	-0.338497367	0.333248719	0.309748479	0.54940888

ulaB	-0.339235083	0.368554989	0.357339573	0.593898665
yjaB	-0.339603118	0.45259524	0.453046225	0.67240721
rfbC	-0.340281894	0.274482032	0.215077589	0.443446489
kch	-0.340384803	0.410591934	0.407098771	0.635841683
pgpC	-0.340434329	0.365197171	0.351236675	0.587626381
yihA	-0.340665473	0.523513605	0.515221454	0.725705969
lepB	-0.341053662	0.283318271	0.228673538	0.460196947
wcaA	-0.341130821	0.424873716	0.422032924	0.647405953
ubiC	-0.341392881	0.411192821	0.406397394	0.635374504
glpQ	-0.341457372	0.292526338	0.243101139	0.475945343
lptC	-0.342106821	0.56938387	0.547948646	0.750792319
yibG	-0.342224435	0.492831809	0.487428816	0.7011811
orn	-0.34240133	0.330711346	0.300506421	0.540616944
ybhQ	-0.343158437	0.306610868	0.263055447	0.500449971
yqjF	-0.343249334	0.340648923	0.313630345	0.553376618
mppA	-0.343533297	0.433131291	0.427696873	0.651797764
lsrR	-0.34356401	0.310389144	0.268345152	0.505865854
ldhA	-0.34356524	0.26576588	0.196102227	0.418866512
elaD	-0.344317192	0.319314011	0.280898649	0.518275981
yigG	-0.345130946	0.369882218	0.350776926	0.587344027
araF	-0.34546836	0.324957326	0.287727916	0.526544694
entH	-0.345551158	0.312792823	0.269277234	0.50702994
ydaS	-0.346868482	0.749829603	0.643653683	0.814554834
nei	-0.348506962	0.415256836	0.401325438	0.632252636
rsuA	-0.348681806	0.467870805	0.456119195	0.674488348
ybjO	-0.349258438	0.429648829	0.416279525	0.643384549
ccmE	-0.349402626	0.471438177	0.458607355	0.677423305
yagK	-0.349675724	0.335713405	0.297601809	0.536347545
ydgD	-0.34999039	0.274378055	0.20210502	0.424946857
fadL	-0.350582688	0.350852025	0.317682155	0.556874885
mqsR	-0.350697902	0.695065227	0.613872164	0.798647092
chrR	-0.350703466	0.509151252	0.490949032	0.703821465
yecE	-0.351376659	0.491514113	0.474679362	0.692297899
pykA	-0.352417399	0.240872146	0.143443015	0.34654665
paoA	-0.354196461	0.367888084	0.335656342	0.57363869
gspM	-0.354334915	0.251320745	0.158571801	0.369343025
ychF	-0.35492065	0.226938408	0.117828972	0.304529807
ydhF	-0.355171261	0.225044632	0.114513727	0.299385538
fdhE	-0.356194425	0.435141627	0.413031076	0.640088529
ftsH	-0.356410348	0.709278996	0.61531818	0.799075767
yieP	-0.356555186	0.498621315	0.474558303	0.692297899
casE	-0.356807568	0.458862288	0.436809676	0.658788667
sad	-0.357014928	0.514348991	0.487613045	0.7011811
surE	-0.357743451	0.390510776	0.359619109	0.595236713
tfaQ	-0.358031658	0.776261876	0.644636933	0.814777489
sohB	-0.358442242	0.270529441	0.185182514	0.406981289
yfhR	-0.358572037	0.218062891	0.100103622	0.274164397
yfjT	-0.359147896	0.375034471	0.338244477	0.575914362
fdoG	-0.360247938	0.547499147	0.510545761	0.722170021

exbD	-0.360869225	0.585456132	0.537637242	0.743556543
ygdD	-0.360879284	0.402057144	0.3694084	0.604255151
yeaE	-0.361663087	0.360590358	0.31587296	0.555149821
gatC	-0.361835191	0.504471263	0.473215967	0.691070505
murR	-0.362806565	0.337301606	0.282099612	0.518933632
pgpA	-0.363036958	0.414480075	0.381092454	0.615880799
ybaO	-0.363151437	0.333944847	0.276833865	0.51382911
yggM	-0.363167935	0.384649207	0.34509116	0.580956219
yfdF	-0.363411495	0.255008221	0.154129113	0.363526107
ubiX	-0.363529904	0.308195382	0.238181768	0.470420645
nrfF	-0.363616228	0.395261196	0.357604586	0.594094532
ygeW	-0.363714675	0.536321367	0.497666733	0.710272152
ydhJ	-0.36530278	0.452567901	0.419564031	0.645529891
rssB	-0.365677157	0.291616535	0.209854442	0.437008586
ycdY	-0.366185346	0.274782035	0.182650193	0.403589946
zupT	-0.366813469	0.251076695	0.144025916	0.347538926
ycjZ	-0.366946777	0.332129172	0.269232602	0.50702994
dsbC	-0.367929052	0.530295	0.487795364	0.7011811
fucU	-0.367958321	0.335099128	0.272179108	0.509169165
ydaU	-0.368151759	0.426219656	0.3877194	0.621366898
hybE	-0.368293489	0.557063334	0.508526407	0.720576028
proX	-0.368885131	0.234380234	0.11551641	0.301058584
yigF	-0.370545638	0.34378425	0.281103578	0.518417152
dcm	-0.37214857	0.314690285	0.236972956	0.468871769
yhbJ	-0.372177809	0.411048217	0.365234508	0.600390732
psuK	-0.373924782	0.411914132	0.36399784	0.599779298
nadA	-0.373976784	0.256879562	0.145435518	0.349573212
wcaF	-0.374187242	0.444175678	0.399546896	0.630805952
ycdU	-0.374588084	0.319698064	0.241320209	0.474529802
yfiR	-0.3748854	0.358246194	0.295355031	0.533966978
hcaC	-0.37566271	0.336150154	0.263761642	0.501085058
tusC	-0.376411545	0.327335073	0.250173805	0.484854369
gfcB	-0.376610364	0.426060818	0.376730928	0.61135866
hycA	-0.377054128	0.283860319	0.184076151	0.406191088
rpiB	-0.377691599	0.483542418	0.434747797	0.65659441
prc	-0.378018806	0.204374272	0.06436547	0.205543614
pncB	-0.378188954	0.283220439	0.181772748	0.402091278
yneK	-0.380104655	0.359882605	0.290881105	0.530152154
uvrY	-0.380453487	0.399779895	0.34127077	0.577860031
tolQ	-0.380700142	0.253806378	0.133624021	0.329328554
oppB	-0.381238731	0.461695084	0.408953278	0.637379914
atoA	-0.38175129	0.573117039	0.505349382	0.718343469
tolR	-0.382286493	0.492040784	0.437193779	0.658788667
eptB	-0.382652247	0.554736972	0.490325996	0.703368552
typA	-0.382936616	0.755054222	0.612039477	0.797805414
ydeE	-0.383915042	0.418490008	0.3589427	0.594734274
dtpA	-0.384746955	0.470602169	0.413606721	0.640479605
yfdN	-0.385036075	0.303711535	0.204880449	0.42966357
yfdK	-0.385559603	0.293993708	0.189703991	0.411900695

rsxE	-0.38623017	0.537985369	0.472806943	0.690814922
truB	-0.387269998	0.510486428	0.448074298	0.668468566
flgC	-0.387858137	0.353137501	0.272064587	0.509169165
rbn	-0.388696627	0.32088223	0.225766143	0.456851088
uspC	-0.39011331	0.366862061	0.287610238	0.526544694
ydeS	-0.390268644	0.323375429	0.227486333	0.458264634
yceB	-0.391205533	0.442403587	0.376548877	0.61135866
rseP	-0.391462988	0.351136628	0.264916758	0.502569997
pagP	-0.391848234	0.297913064	0.188405511	0.410760621
mnmA	-0.392600347	0.289739189	0.175413437	0.393632044
ecpA	-0.392791259	0.438332888	0.370197946	0.605056319
znuC	-0.393200811	0.291526383	0.177412208	0.396793952
yneJ	-0.393259219	0.496841489	0.428641499	0.652387716
ydeM	-0.393267343	0.403554545	0.329804111	0.567012106
waaN	-0.394067332	0.326450031	0.22738243	0.458264634
ynfC	-0.395525121	0.379523423	0.297336296	0.536108363
yeeY	-0.396484724	0.378277894	0.294578333	0.533518498
selD	-0.396573323	0.218198597	0.06914243	0.214654492
rluB	-0.396667851	0.34572932	0.251242594	0.486381415
tehB	-0.397635868	0.332445676	0.23166036	0.464355945
acnA	-0.397764346	0.330138701	0.228265036	0.459603966
ydiN	-0.397872299	0.406234613	0.327374937	0.566001122
pbpG	-0.398241919	0.256747896	0.120877481	0.309308482
yjjA	-0.398291456	0.302510564	0.187966033	0.410760621
yliE	-0.398395953	0.409284155	0.330356048	0.567206318
ilvH	-0.398680817	0.23655578	0.091919815	0.258953448
nrdF	-0.400538362	0.379124016	0.290747262	0.53014756
dacA	-0.401306995	0.371536644	0.280085349	0.517721866
mscL	-0.402366849	0.317503694	0.205054324	0.429804936
flgB	-0.402834828	0.378122189	0.286714757	0.525405118
cvpA	-0.402931064	0.340859831	0.237165259	0.468871769
folM	-0.403123436	0.315857506	0.201855642	0.424946857
psiF	-0.403731053	0.29592036	0.172465824	0.388312622
glf	-0.40412779	0.341831233	0.237109054	0.468871769
yjcH	-0.405092168	0.523339512	0.438899848	0.659079275
cynR	-0.405266937	0.417652143	0.331874234	0.569148803
ycaP	-0.40545756	0.257749889	0.11570356	0.301157493
puuR	-0.405890108	0.293291948	0.166385546	0.379919938
psuG	-0.40643316	0.500778582	0.417019743	0.644281938
ydjI	-0.406803316	0.398175042	0.306937328	0.546584028
tdk	-0.406827827	0.398013898	0.306712383	0.546584028
cysZ	-0.407289886	0.241337041	0.091480481	0.258953448
smrA	-0.408049945	0.453244379	0.367967628	0.603034196
rsxA	-0.408570934	0.504267965	0.417810391	0.645009769
manA	-0.409209201	0.31221356	0.189969003	0.411983443
yegL	-0.409235815	0.41918122	0.328928595	0.56686713
yqeH	-0.410173494	0.544939255	0.451632728	0.671516637
yehD	-0.410368919	0.235967805	0.08201918	0.242023204
ycbJ	-0.410679803	0.307742616	0.18204294	0.402468428

emtA	-0.411317973	0.361694003	0.255455204	0.491337944
trpA	-0.411615317	0.28006897	0.141645005	0.344263024
yoaH	-0.411751033	0.713210941	0.563723218	0.762705189
yebZ	-0.412433373	0.279355669	0.13984359	0.340705233
ymfA	-0.41309474	0.253257309	0.102863603	0.277812336
yjfI	-0.414380461	0.379386161	0.274727949	0.512039118
yccU	-0.41590905	0.264169932	0.115395084	0.300936662
mmuP	-0.416091678	0.663938887	0.53085474	0.738532135
yaaH	-0.416586887	0.311349383	0.180894958	0.400808423
argT	-0.417099522	0.383501356	0.276767761	0.51382911
mglB	-0.417911098	0.385145906	0.277889699	0.514605833
nlpE	-0.418558182	0.386101086	0.278336545	0.515196988
ygbA	-0.418638277	0.2937855	0.154163165	0.363526107
ivy	-0.418784997	0.541153909	0.439005244	0.659079275
purU	-0.41961446	0.265229403	0.113631028	0.297875624
phoE	-0.42110845	0.32254682	0.191697775	0.412518079
ychQ	-0.421807259	0.31170262	0.175980195	0.394568148
codB	-0.421983429	0.496365451	0.395243553	0.62720056
gltX	-0.423469171	0.412473319	0.304581375	0.544689032
btuR	-0.424958402	0.34146784	0.213313795	0.441161778
nudG	-0.427104243	0.398644771	0.283993857	0.521840328
cybB	-0.427132179	0.256979133	0.096487139	0.267159519
yhbE	-0.427253794	0.262479928	0.103576216	0.279130296
ybeT	-0.427369729	0.366840466	0.244018346	0.47727813
ycjW	-0.427970927	0.434918014	0.325102397	0.564246938
yccF	-0.428265158	0.341613836	0.209968214	0.437008586
nfo	-0.42835932	0.275189672	0.119566281	0.30705412
yaiO	-0.428466955	0.283467054	0.130655268	0.324188886
yafK	-0.428766027	0.204733817	0.036236746	0.143138693
yodB	-0.429015189	0.235926091	0.068998367	0.214654492
yibI	-0.430170628	0.535329603	0.421649867	0.647224529
ydiP	-0.430223984	0.451252671	0.340387792	0.577728528
pntA	-0.430854996	0.434152771	0.321000433	0.560818235
yceH	-0.431198683	0.250010753	0.084577846	0.247522707
lpxT	-0.43282812	0.390039252	0.267126597	0.504628016
tabA	-0.433407745	0.553058279	0.433241662	0.655054902
yacL	-0.433623043	0.792582773	0.584309159	0.778500355
fadM	-0.434020059	0.320761548	0.176026067	0.394568148
ydfR	-0.434058434	0.340976747	0.203023209	0.42621149
hybD	-0.43407828	0.368838333	0.239243598	0.471496507
topA	-0.434428808	0.21158155	0.0400488	0.152525476
ynbA	-0.435273652	0.479920995	0.364422984	0.600034454
perR	-0.435608059	0.20427862	0.032972236	0.134467273
pyrG	-0.435696385	0.424039424	0.304189662	0.544689032
ydcF	-0.436452326	0.273142788	0.1100673	0.29123741
ybhH	-0.43653144	0.4511404	0.333235331	0.57099789
sodC	-0.438078166	0.372070103	0.23903286	0.4714097
aroM	-0.438481553	0.232813495	0.059645821	0.195401212
yfbR	-0.439052129	0.285210301	0.123707195	0.313911887

hscB	-0.439105415	0.279754313	0.116505664	0.302854711
yjbR	-0.439195499	0.538052327	0.414346232	0.641378735
ybjH	-0.439548395	0.435495843	0.312828093	0.552444013
chiQ	-0.439554923	0.417547246	0.292475284	0.53151325
plsX	-0.440021189	0.403478826	0.275462916	0.513172032
ychO	-0.440576697	0.405353095	0.277082731	0.514054681
smrB	-0.441248127	0.271991955	0.104742608	0.281372027
yfdX	-0.441838211	0.562236526	0.431950537	0.654819497
lolB	-0.441867507	0.248016872	0.074814058	0.226750918
thiS	-0.442889191	0.496897895	0.372763826	0.607528286
ybjE	-0.44291189	0.233966972	0.058350815	0.192282462
hypC	-0.443508732	0.455134372	0.329829836	0.567012106
maa	-0.443706156	0.535987994	0.407767612	0.636157269
chbR	-0.44469746	0.471677981	0.345783799	0.581637165
yecT	-0.444758826	0.344287835	0.196418567	0.418866512
ychE	-0.444932643	0.270338802	0.099798054	0.273513064
ybjG	-0.446074621	0.24850322	0.07264643	0.221338593
minD	-0.446222403	0.40238345	0.267452529	0.504771323
mcbR	-0.446862224	0.333805269	0.180671175	0.400752492
ybgA	-0.447278094	0.337247802	0.184753765	0.406901772
ygaC	-0.447842754	0.388823491	0.249407647	0.484066669
zraP	-0.448723173	0.659148324	0.496022058	0.708577866
ycaC	-0.449298249	0.255299247	0.078426687	0.234502043
arfB	-0.449849164	0.277529867	0.105038312	0.28175393
ydcL	-0.450091974	0.258256823	0.081367215	0.240644284
gtrS	-0.450249962	0.294968401	0.126901676	0.318755738
yedL	-0.45155754	0.258901384	0.081136513	0.240137904
yfgI	-0.451765719	0.497049289	0.363405441	0.599779298
kdgR	-0.453338253	0.311203033	0.145191406	0.349515628
fepE	-0.453728286	0.416251413	0.275698069	0.513373204
ybhK	-0.45441594	0.278838877	0.103170815	0.27840948
cobB	-0.45557222	0.326910775	0.163448259	0.375561936
mpaA	-0.456093832	0.291805504	0.11805112	0.304908748
tqsA	-0.456947128	0.408418089	0.263216099	0.50049183
rnlA	-0.457352954	0.475170866	0.335797452	0.57363869
yfjI	-0.459300153	0.51732982	0.374633016	0.609836083
ycfD	-0.459305132	0.229997382	0.045825013	0.164880193
lpxA	-0.459407762	0.421755866	0.276033008	0.513523158
allA	-0.459879789	0.387065655	0.234786804	0.467241882
potF	-0.460240378	0.478566757	0.336197459	0.57363869
ydfG	-0.461186695	0.477635793	0.334263688	0.57198625
yfgD	-0.462150394	0.282037477	0.101293796	0.2761128
folK	-0.462249291	0.251571249	0.066143667	0.209101006
focA	-0.462806879	0.312581035	0.138713708	0.33877026
artI	-0.463482631	0.303461652	0.126681801	0.31843987
spy	-0.464470471	0.284120132	0.102097713	0.276900479
bluR	-0.464563011	0.303628389	0.126007293	0.317337143
yajO	-0.465319906	0.387682601	0.230038392	0.462022383
xdhC	-0.465393613	0.298532621	0.119011241	0.306541079

yecA	-0.465971192	0.491585873	0.343183532	0.579193946
ybbL	-0.46719596	0.330282472	0.157205018	0.367692154
ykgF	-0.467359751	0.407816125	0.251792644	0.487056495
rspB	-0.468404055	0.373751585	0.210114854	0.437008586
deoA	-0.468431381	0.571908316	0.412748036	0.640088529
smtA	-0.468843894	0.381490247	0.219079294	0.449630458
fbp	-0.468931515	0.468105058	0.316456845	0.555356132
mrp	-0.469264753	0.358873685	0.191007578	0.411983443
yddE	-0.469751517	0.384417024	0.221713615	0.452506504
guaA	-0.469861933	0.537949681	0.382428194	0.616969633
csgE	-0.470622781	0.397137234	0.236002355	0.467717972
sdhA	-0.471321933	0.559702965	0.399735915	0.630857658
acpS	-0.472987485	0.360382923	0.189365616	0.411900695
yciC	-0.476237246	0.2332046	0.041137304	0.155352009
cysP	-0.476722044	0.528830461	0.367341243	0.602582933
ydeR	-0.477004678	0.30172102	0.113890356	0.298168201
yqgE	-0.477178992	0.240418496	0.047168412	0.168512284
yadL	-0.477521601	0.372545248	0.199919311	0.423216706
ygaU	-0.478043275	0.343603192	0.164144924	0.376645888
yedI	-0.479039126	0.370982912	0.19661005	0.418866512
yiaB	-0.480638054	0.424226037	0.257224199	0.493447271
dld	-0.481479748	0.212239119	0.023294359	0.105900142
yajG	-0.481662389	0.518205263	0.352639425	0.588996838
yehC	-0.481671808	0.498410724	0.333836352	0.571542559
yhdT	-0.481682197	0.353805991	0.173377746	0.389930897
cobU	-0.482033491	0.350334821	0.168845691	0.383619871
yeeN	-0.482559091	0.348024566	0.165574089	0.378709686
waaS	-0.483269968	0.349032366	0.166175027	0.37965398
hisG	-0.48576645	0.362396879	0.180106662	0.400159931
yeaR	-0.487985892	0.281311872	0.082797618	0.243981009
rdoA	-0.489331134	0.428149213	0.253080695	0.489057512
ygaV	-0.489744601	0.498897909	0.326270856	0.564974245
yfcG	-0.489778651	0.39864628	0.219220092	0.449690809
potA	-0.49051474	0.257180631	0.056484708	0.189392663
alkB	-0.490675516	0.481033015	0.307706928	0.546748622
yggD	-0.491813658	0.356866798	0.168158987	0.382670705
higA	-0.492308091	0.464107919	0.28879803	0.52820317
accD	-0.492806433	0.544305694	0.365261243	0.600390732
rrrQ	-0.493630624	0.46357592	0.286951815	0.525600943
dapD	-0.493737551	0.340121236	0.146598491	0.351227364
yhbO	-0.494683777	0.244382851	0.04294789	0.159762217
preT	-0.494838697	0.24343893	0.042082682	0.158448422
yecM	-0.49495671	0.255933319	0.053121934	0.182313445
ves	-0.495229075	0.387724367	0.201506602	0.424794857
xthA	-0.495505543	0.426259558	0.24505261	0.478723175
tusB	-0.495582049	0.753656146	0.510814156	0.722296584
sanA	-0.497567955	0.314474177	0.113598905	0.297875624
wcaE	-0.498030231	0.542595002	0.358688322	0.594734274
yhaI	-0.499533715	0.350202803	0.153749258	0.363399154

arsC	-0.499899271	0.274372947	0.068459542	0.213578958
ttcA	-0.500432272	0.23852171	0.035900063	0.142092
yidH	-0.500683828	0.285447635	0.079425299	0.236460128
lipB	-0.501284099	0.29599591	0.090350681	0.257953112
ycbC	-0.50315239	0.523813089	0.3367749	0.574138628
ydhY	-0.504704216	0.256098702	0.048753507	0.172043625
wcaB	-0.505316688	0.571906915	0.376931311	0.61135866
chbA	-0.506759852	0.304440896	0.096000758	0.266177926
bcp	-0.506957482	0.443944727	0.253479671	0.489147912
yrdB	-0.50951944	0.787662788	0.517712786	0.727718147
nadE	-0.510854314	0.385569699	0.185193121	0.406981289
ycfL	-0.512036114	0.448712334	0.253818603	0.489334145
hdhA	-0.513490016	0.396617396	0.1954329	0.418281084
yohD	-0.513531419	0.40236161	0.201851864	0.424946857
nfsA	-0.51361347	0.321381482	0.110011222	0.29123741
ygdH	-0.517919208	0.647134199	0.423521153	0.648863337
rzpQ	-0.519255677	0.520288801	0.318272412	0.557667415
yciM	-0.519257583	0.365804128	0.155754394	0.365519541
uxaC	-0.519587328	0.749344045	0.48806512	0.7011811
flgN	-0.519791093	0.437537208	0.23483621	0.467241882
yfdT	-0.520126496	0.387658606	0.179689151	0.399854937
cptA	-0.520791452	0.394884254	0.187220659	0.41004487
ratA	-0.521059426	0.568077915	0.359020913	0.594734274
trxB	-0.522692544	0.378353835	0.16712794	0.381183895
arsR	-0.523041204	0.510031774	0.305123979	0.544796772
umuD	-0.523066326	0.282342598	0.06394051	0.204700903
wzzB	-0.524838289	0.486399509	0.280575613	0.517916666
ruvC	-0.524860562	0.327878108	0.10942576	0.290817507
ygfB	-0.525740362	0.650405549	0.418902368	0.645529891
glpE	-0.526075322	0.62807956	0.402259076	0.632682168
rraB	-0.526396002	0.678977916	0.438175962	0.659079275
fliR	-0.526587385	0.371419042	0.156257445	0.365719093
yeaM	-0.526601623	0.612183052	0.389677219	0.622281223
epd	-0.526998391	0.500689602	0.292549527	0.53151325
ftsZ	-0.527077274	0.652700304	0.419359259	0.645529891
infB	-0.527682324	0.679642763	0.437506172	0.658788667
lacI	-0.527738031	0.224851081	0.018922422	0.092369789
yiaL	-0.527858057	0.275404109	0.055280209	0.187063037
yafY	-0.52833303	0.466633761	0.257541095	0.493447271
nupG	-0.529456054	0.612387294	0.387270925	0.621250121
ydjX	-0.530504741	0.265599456	0.045783224	0.164880193
cheW	-0.531032923	0.292083095	0.069050603	0.214654492
add	-0.531184285	0.3168563	0.093655926	0.261111169
yhaB	-0.531701372	0.262708075	0.042977896	0.159762217
yfeY	-0.531822593	0.417745402	0.20299027	0.42621149
msrC	-0.532856515	0.416307029	0.20055906	0.424126204
paaC	-0.532911527	0.473129384	0.260015394	0.496773376
aspS	-0.533860431	0.383093936	0.163453634	0.375561936
marC	-0.535250629	0.32315806	0.097658616	0.269663361

rseC	-0.536520102	0.281148431	0.056350362	0.189099262
yneE	-0.536584519	0.416200938	0.197313309	0.41990186
acrR	-0.537247622	0.401980865	0.18138574	0.401675389
yohC	-0.537462937	0.366027479	0.142004363	0.344721355
ybaN	-0.540529063	0.298266733	0.069950074	0.216167474
exuR	-0.545669428	0.541081013	0.313224042	0.55290138
ynfK	-0.546856689	0.320249639	0.087711512	0.253543815
ffh	-0.546924986	0.522286503	0.295019212	0.533837991
pgsA	-0.54735505	0.41166625	0.183647212	0.405571003
psuT	-0.547400737	0.6300699	0.38496	0.619293399
mtn	-0.547510939	0.432557182	0.205601206	0.430504185
yidI	-0.547823669	0.423966695	0.196309201	0.418866512
matP	-0.548077249	0.454097854	0.227448139	0.458264634
hybF	-0.548732029	0.33122568	0.097585978	0.269647223
nsrR	-0.551282798	0.745873299	0.459839892	0.677937254
yhhM	-0.552252951	0.35447179	0.119242676	0.306613175
ybaV	-0.552346488	0.379744302	0.145801605	0.349941189
yhbT	-0.553386566	0.52179358	0.288895863	0.52820317
ygjH	-0.553922806	0.433402531	0.201221465	0.424415389
pinQ	-0.555299743	0.444862511	0.211939425	0.438994079
glpF	-0.555580623	0.639878815	0.385252491	0.619380448
yahB	-0.555784481	0.424506138	0.190449824	0.411983443
yahL	-0.555785201	0.27726604	0.045014434	0.163714656
tmk	-0.557432555	0.331018017	0.092182711	0.259257256
ybjK	-0.557768217	0.26775466	0.037239222	0.145956056
yafJ	-0.560046318	0.378464611	0.138930588	0.33909479
ybgC	-0.560654717	0.546586618	0.305015052	0.544796772
ymdA	-0.560868193	0.279339702	0.044660767	0.162868577
ybfB	-0.560991338	0.800728286	0.483551821	0.699190852
serC	-0.561887179	0.324391652	0.083251151	0.245138508
nuoH	-0.563468265	0.511886295	0.270997462	0.508372098
nupC	-0.563821675	0.594719364	0.343105728	0.579193946
nemR	-0.563853566	0.45155532	0.211777707	0.438884294
pgpB	-0.564363658	0.397460348	0.155629682	0.365489836
ydeH	-0.565545363	0.249623344	0.023476201	0.1064337
hlyE	-0.565940048	0.393936101	0.150823424	0.358988723
dppA	-0.566160752	0.521358397	0.277508242	0.514371337
lpxD	-0.566722686	0.386789508	0.142867277	0.345776498
yjgZ	-0.567493213	0.712934681	0.426034313	0.65104145
ykgG	-0.568356083	0.298313193	0.056749815	0.189966006
yaiS	-0.568936423	0.261422112	0.029531841	0.124187542
gadE	-0.568990605	0.434475827	0.190330361	0.411983443
map	-0.570111671	0.459371185	0.214579992	0.443099451
fnr	-0.570944749	0.338178795	0.09135508	0.258953448
hybA	-0.571082921	0.624123119	0.360182994	0.595681584
yaiL	-0.571917023	0.317742702	0.071870499	0.219970586
yqhA	-0.572056441	0.369613718	0.121691095	0.310436755
speE	-0.572128868	0.580161158	0.324057022	0.563887155
ydjF	-0.57228056	0.327450036	0.080518035	0.238832703

hcaR	-0.572347259	0.234103014	0.014491153	0.079592905
rpoC	-0.572496025	0.778295787	0.46198852	0.680674327
gpmA	-0.572579025	0.370257083	0.121999212	0.310732379
ynbB	-0.57336375	0.506282547	0.257425309	0.493447271
dcrB	-0.574533609	0.655683413	0.380901586	0.615818863
narU	-0.576743911	0.484442612	0.233837783	0.46608109
ybjM	-0.577622307	0.375020211	0.123501098	0.313765847
nuoJ	-0.578705196	0.560488107	0.301836923	0.542197989
ydaV	-0.580134447	0.391462707	0.138349325	0.33825081
thyA	-0.580668991	0.412584217	0.159310274	0.369618146
glnP	-0.581245029	0.390447714	0.136576158	0.334968377
ydfV	-0.581738238	0.433759346	0.179870341	0.399854937
loiP	-0.58299623	0.41113554	0.15618648	0.365719093
gmhA	-0.585342314	0.409735236	0.153123055	0.36255588
wbbK	-0.585374832	0.347161478	0.091762193	0.258953448
yggN	-0.585901829	0.24145286	0.015242386	0.081935433
rob	-0.588679629	0.646866479	0.362797128	0.599513716
nuoI	-0.588982584	0.330077691	0.074362651	0.225715805
proQ	-0.589117862	0.234916358	0.012149328	0.069569982
yjaA	-0.589335301	0.357739474	0.099478559	0.272822651
yqcA	-0.589746927	0.603976003	0.328845976	0.56686713
yfeK	-0.589881064	0.559931243	0.292117213	0.53151325
pflA	-0.591369151	0.458228826	0.196858295	0.419154502
ykgB	-0.59235064	0.222021009	0.00763057	0.049765124
pssA	-0.593617179	0.453901858	0.190937919	0.411983443
caiF	-0.595439134	0.698243764	0.393788612	0.626863022
fabR	-0.597173612	0.55902912	0.285415215	0.523498965
yadI	-0.597289375	0.597498232	0.317479701	0.556761751
yjdN	-0.598026069	0.369750613	0.105796967	0.283224375
mlaC	-0.598448287	0.594409727	0.314033665	0.553604326
ydcN	-0.599354219	0.306888975	0.050819576	0.176404667
yphF	-0.599832983	0.543259265	0.269533452	0.507275778
mocA	-0.60152201	0.495992801	0.22522022	0.456432744
mazG	-0.602675456	0.321997241	0.061251035	0.198292243
rna	-0.602827326	0.285027134	0.034431461	0.138671982
ydeJ	-0.6029453	0.280547778	0.031620804	0.130658329
ybfP	-0.603412049	0.524375847	0.249845661	0.484450976
nudE	-0.603480247	0.404234715	0.135464504	0.332848571
zwf	-0.604511288	0.277872888	0.029592986	0.124315176
marR	-0.605789534	0.294225114	0.039500743	0.151151183
yfcV	-0.606095812	0.38144622	0.112073552	0.295445386
ispG	-0.606097689	0.473065543	0.200118887	0.423417163
fdoH	-0.607169663	0.596535669	0.308760535	0.548379357
yaaX	-0.608591191	0.528079034	0.249131143	0.484066669
ychJ	-0.610679812	0.405163245	0.131748163	0.326415717
yehR	-0.611996334	0.378092677	0.105524514	0.282870162
yhbQ	-0.612113493	0.428262308	0.15291925	0.362365683
ynaI	-0.613431256	0.426622431	0.150468508	0.358372487
yohJ	-0.61634403	0.284293617	0.030159899	0.126171516

pspC	-0.616680303	0.381098733	0.105627229	0.282957613
queE	-0.617701933	0.517058535	0.232225351	0.464796105
dinB	-0.621410959	0.626178668	0.321009254	0.560818235
yqgF	-0.622507068	0.269521323	0.02090607	0.098300179
lptA	-0.623313397	0.578797376	0.281519924	0.518933632
araJ	-0.62398409	0.569464005	0.273192879	0.510828927
intF	-0.624057321	0.425569085	0.142537545	0.345600041
gtrA	-0.626787387	0.576852504	0.277229372	0.51409048
gltK	-0.627857316	0.321662033	0.050948161	0.176699078
mhpC	-0.628266115	0.612221327	0.304793788	0.544689032
mltD	-0.630290252	0.537177056	0.24066029	0.473693608
ygbF	-0.630758165	0.518775222	0.224038167	0.455638329
ygeG	-0.632708893	0.645766129	0.327194608	0.565931719
ilvM	-0.635204451	0.525910854	0.227117382	0.458207332
yahE	-0.640127566	0.466207591	0.169735981	0.384904479
yjeI	-0.640194906	0.425867956	0.132769201	0.327884315
pphA	-0.640277885	0.374346922	0.087194444	0.25251361
ydiI	-0.640471293	0.356495948	0.072403301	0.221098431
oppA	-0.641994695	0.495965601	0.195515582	0.418281084
yafD	-0.642034732	0.361273928	0.075545034	0.22810419
mioC	-0.642405827	0.617514901	0.298196726	0.537179913
minC	-0.643376311	0.399572435	0.107362516	0.286662354
glyA	-0.643648252	0.557940492	0.248658243	0.483593615
sbmC	-0.645083933	0.526823627	0.220771966	0.451040702
aphA	-0.646400579	0.247291377	0.008950925	0.055936352
hdeA	-0.647694272	0.306926362	0.03483601	0.139403565
paaI	-0.651269569	0.371573584	0.079647716	0.236947552
rimJ	-0.651445059	0.238540966	0.006315076	0.04374322
smg	-0.651602883	0.825703877	0.430025284	0.653463765
ychA	-0.651650611	0.232006068	0.004973188	0.037317395
atpG	-0.65269627	0.7858045	0.406194563	0.635374504
sdiA	-0.652864877	0.571514127	0.253311681	0.489057512
fliJ	-0.652880294	0.505468166	0.196483633	0.418866512
nusG	-0.653198859	0.696872857	0.348589557	0.585320532
yafL	-0.653580228	0.414196603	0.114577819	0.299385538
csgB	-0.655852895	0.388921465	0.091730791	0.258953448
rnhA	-0.656121642	0.257911781	0.01095983	0.064341916
aspC	-0.656226918	0.400449524	0.101270404	0.2761128
napD	-0.657056971	0.47006557	0.162173571	0.374060132
fkpA	-0.657066875	0.486772936	0.177066458	0.39645995
yedR	-0.657354246	0.335463824	0.050049767	0.175087444
fxsA	-0.657618678	0.307990888	0.032745596	0.134070963
yahN	-0.658565427	0.558506161	0.238336394	0.470495854
sodA	-0.659721394	0.593378182	0.266221593	0.503861496
aroL	-0.660160315	0.296623601	0.026042155	0.113936011
gstA	-0.660301994	0.262254126	0.01180922	0.068300604
gstB	-0.663451396	0.329236306	0.043891845	0.161655802
ybaY	-0.668551145	0.379092162	0.077806367	0.233534798
yjhX	-0.668641064	0.678406855	0.324327073	0.563926663

yecN	-0.668894367	0.332119697	0.044007859	0.161655802
yjdK	-0.669521402	0.696063118	0.336115508	0.57363869
intR	-0.67093485	0.476572427	0.15918024	0.369528827
ynfA	-0.671281368	0.330504324	0.042246681	0.158503581
yciO	-0.671827665	0.318036562	0.034650207	0.13918695
cyoC	-0.672570157	0.792489232	0.3960591	0.628247775
mazF	-0.673931522	0.590742	0.253943967	0.489342146
yebC	-0.674325192	0.482416607	0.162171105	0.374060132
ybeY	-0.674425809	0.508133089	0.184421985	0.406392768
ybdZ	-0.676219225	0.594650072	0.255466521	0.491337944
rihA	-0.677042687	0.516113121	0.189584031	0.411900695
manX	-0.677853053	0.262629238	0.009850699	0.059710616
rcsA	-0.678517157	0.301428772	0.024385329	0.108657367
pth	-0.67892585	0.322142507	0.035071435	0.140181567
erfK	-0.680364764	0.242973837	0.005107784	0.03783509
ydgA	-0.682442106	0.251455355	0.006648233	0.045722177
tgt	-0.682551168	0.368520815	0.064006102	0.204748521
sdaC	-0.683150658	0.752048658	0.363674189	0.599779298
iscS	-0.684785337	0.760362392	0.367798959	0.603034196
ybjQ	-0.68594719	0.303082011	0.023620927	0.106664075
frmR	-0.688348246	0.488418385	0.158734245	0.369343025
higB	-0.689275023	0.255617461	0.007007031	0.046911083
ydiV	-0.690728541	0.319250865	0.030495694	0.12678797
secM	-0.692407756	0.442300322	0.117472815	0.30399856
hybO	-0.692767019	0.801392942	0.387338755	0.621250121
cmoA	-0.694015811	0.380516951	0.06817079	0.213152669
yjeO	-0.695863275	0.413508939	0.09240893	0.259280599
rpoB	-0.696067655	0.766700182	0.363944455	0.599779298
lipA	-0.697469579	0.42319008	0.0993273	0.272593005
amiA	-0.69781351	0.257233338	0.006672447	0.045810663
mntP	-0.698722846	0.521642908	0.180419074	0.400413305
mtfA	-0.699089413	0.237425678	0.003235232	0.028208704
speG	-0.69919227	0.247244071	0.004684807	0.035819253
ydjH	-0.700006847	0.521903093	0.179836609	0.399854937
ynfD	-0.700772996	0.432247201	0.104967891	0.281752245
gyrA	-0.701113238	0.7168364	0.328041732	0.566426207
apt	-0.701857695	0.442073296	0.112365492	0.295710228
crcB	-0.702406025	0.364598151	0.054039342	0.183943357
yffB	-0.703833266	0.382167173	0.065520583	0.207672124
yafW	-0.704310285	0.266868124	0.008310935	0.053003546
flgM	-0.7043287	0.365412656	0.053918777	0.183781364
nrdR	-0.70472077	0.414405787	0.089026156	0.255835955
yjeN	-0.705095622	0.347413703	0.042401675	0.158937382
ydgK	-0.707301196	0.334289581	0.034358945	0.138568493
gmhB	-0.707850933	0.6377388	0.267025471	0.504628016
yjbT	-0.707981156	0.792549098	0.371698533	0.606526668
maeA	-0.710274466	0.421828271	0.092220317	0.259257256
lpxP	-0.710957227	0.374876526	0.057892587	0.191882081
slyD	-0.711170191	0.771152664	0.356415334	0.592896394

narP	-0.711508687	0.749948403	0.342751075	0.579121896
yfbO	-0.71159569	0.332529923	0.032359225	0.132693978
fdx	-0.711757294	0.64123643	0.267009203	0.504628016
rutC	-0.711957771	0.321083255	0.026598372	0.115958563
udk	-0.713919594	0.382370964	0.061889535	0.199213425
rimL	-0.714995856	0.436863777	0.101702303	0.276665901
ygiM	-0.715411213	0.321710643	0.026163768	0.11431075
ydgH	-0.718877777	0.388881078	0.064518485	0.205711948
dedA	-0.719188247	0.263370697	0.00631986	0.04374322
fdoI	-0.721461209	0.486910901	0.138417225	0.33825081
zapC	-0.721645797	0.242269212	0.002894799	0.0260855
sseA	-0.723245863	0.348948248	0.038205324	0.148302783
ycdX	-0.723427813	0.307524477	0.018651525	0.092338281
kdsB	-0.723879659	0.553314489	0.190784864	0.411983443
cfa	-0.725774257	0.225944176	0.0013173	0.015158485
nlpC	-0.728750889	0.296903908	0.014108034	0.07801936
aroD	-0.729416593	0.3881643	0.060224842	0.195868482
yahD	-0.729833166	0.274734728	0.007895614	0.051041494
rseB	-0.730129182	0.60047243	0.224013657	0.455638329
hslJ	-0.732296394	0.283238435	0.009725439	0.059467689
znuA	-0.732965674	0.255164017	0.004072025	0.032628631
fliZ	-0.733564151	0.570421264	0.19844159	0.422057811
yfeZ	-0.735117237	0.643415406	0.253236474	0.489057512
elaB	-0.735451969	0.387607819	0.057773501	0.191802322
yeaP	-0.73606903	0.234514294	0.001697015	0.018317781
nth	-0.736447329	0.35025865	0.035502375	0.141472322
adhE	-0.73785039	0.526047357	0.160726869	0.372049524
ysaB	-0.7383025	0.631959113	0.242695558	0.475456948
frdA	-0.7385024	0.719188643	0.304488782	0.544689032
mprA	-0.738555385	0.428733846	0.084953093	0.247717609
efp	-0.738596583	0.719109571	0.30437394	0.544689032
pnp	-0.740758818	0.747511642	0.321702055	0.561240793
yfgM	-0.741087316	0.270658983	0.006179809	0.043312308
ibpB	-0.74138216	0.40088813	0.0644074	0.205543614
ydbL	-0.742876719	0.336407808	0.027225986	0.117930585
lptE	-0.748806122	0.605224646	0.215999233	0.444665428
ptsN	-0.748964484	0.531824811	0.159044703	0.36942662
ydaT	-0.750507824	0.628990078	0.232793007	0.465470713
ydfH	-0.750786294	0.230011429	0.001098023	0.013555716
lacY	-0.752334366	0.571724878	0.18820667	0.410760621
grpE	-0.752339693	0.297130159	0.011340732	0.066048353
ppiA	-0.752658903	0.507698875	0.138209754	0.338153197
yiiS	-0.752759223	0.643875105	0.242360317	0.475456948
sseB	-0.752946295	0.267481425	0.004878513	0.03695039
yagU	-0.754804981	0.36564179	0.038986526	0.149608939
yajI	-0.756023219	0.326528565	0.020594572	0.097582495
cbl	-0.759147958	0.496156068	0.126002176	0.317337143
hisJ	-0.760754251	0.449584943	0.090622003	0.258179976
rpiA	-0.761443035	0.361991713	0.035423661	0.141309605

yagM	-0.762013718	0.266536064	0.004250436	0.033383286
upp	-0.763746123	0.626650499	0.222929505	0.454232688
fabG	-0.764629405	0.710319387	0.281722212	0.518933632
mqsA	-0.765175263	0.273042946	0.00507241	0.037711093
yghG	-0.765198992	0.250578141	0.002260124	0.022190741
lolA	-0.766067317	0.432214838	0.076324444	0.230113354
yegJ	-0.766832037	0.361885879	0.034091434	0.137902925
uspB	-0.76815894	0.757065697	0.310271291	0.54985303
yidQ	-0.768598596	0.648755643	0.23612524	0.467731891
gloA	-0.772926962	0.295433115	0.008890137	0.055815684
yeaK	-0.775048137	0.417620125	0.063472269	0.203524663
rraA	-0.776832825	0.782712987	0.320959794	0.560818235
rsd	-0.777943108	0.49079093	0.112948057	0.29666318
yfdS	-0.779973901	0.342803114	0.022888993	0.104884069
fliY	-0.780186135	0.248320119	0.001678804	0.01826774
btuE	-0.781588293	0.393964865	0.047266593	0.168713738
yniB	-0.781628486	0.238245244	0.001035231	0.012898856
ybhL	-0.782255311	0.637031981	0.219458731	0.449723299
yfbU	-0.783601822	0.401180479	0.050791144	0.176404667
ybdF	-0.784681687	0.373754183	0.035776434	0.142015205
aldA	-0.785897792	0.379011295	0.038121388	0.148195724
lrhA	-0.786070681	0.798065076	0.324638485	0.563926663
arcA	-0.78613852	0.814545981	0.334482311	0.57198625
eco	-0.787899636	0.255758449	0.002065564	0.020605006
ybaK	-0.78832002	0.309310247	0.010814432	0.064108461
yagL	-0.788548718	0.376706888	0.03632518	0.143207765
ytcA	-0.789146978	0.392600368	0.044425733	0.162304692
deoR	-0.789390517	0.509470516	0.121277625	0.309872007
yfcL	-0.791869315	0.362443628	0.028903013	0.122435953
atpA	-0.791889488	0.936593241	0.39783179	0.629822328
pspG	-0.792626923	0.483493243	0.101135104	0.276103582
hypA	-0.793211293	0.280078053	0.004624223	0.035423126
sufC	-0.79497392	0.670240248	0.235581655	0.467572833
pykF	-0.796420387	0.462464619	0.085047463	0.247717609
yciT	-0.800819288	0.39696423	0.043658082	0.161251306
nfuA	-0.801353148	0.626628497	0.2009559	0.424378054
ftnB	-0.802244798	0.241263908	0.000883629	0.011498307
fabF	-0.802377908	0.605410121	0.185056405	0.406981289
yebE	-0.802442827	0.52180222	0.124090318	0.313911887
hiuH	-0.802948397	0.388783021	0.038895883	0.149403119
pliG	-0.804466703	0.384643426	0.036486764	0.143704455
dgkA	-0.804892932	0.510759435	0.115054655	0.300322628
rstA	-0.805079681	0.344323757	0.019379605	0.093920128
sixA	-0.805572766	0.466521542	0.084209994	0.246881442
yhaH	-0.806440125	0.468771527	0.085373092	0.248307761
iraM	-0.80913826	0.641474974	0.207175026	0.433446977
dsbB	-0.80931078	0.310762989	0.009207015	0.057007238
yicR	-0.809718811	0.559041453	0.14750363	0.352976973
atpI	-0.809729933	0.518378062	0.118277355	0.305102671

ykfM	-0.810717911	0.233382615	0.000513198	0.008061396
rhaM	-0.811621926	0.309016779	0.008627672	0.054336839
nuoE	-0.813599503	0.61890382	0.188650596	0.410778023
atpC	-0.813676918	0.730349244	0.265239328	0.502808599
rcnB	-0.814415596	0.372718758	0.028883907	0.122435953
yahC	-0.814637686	0.511756882	0.111419514	0.294179579
pal	-0.816213969	0.6512277	0.210079627	0.437008586
eutN	-0.818618492	0.511870517	0.109760913	0.29113325
yahA	-0.820613546	0.347130869	0.018079351	0.091119026
clpX	-0.82191392	0.514402997	0.110087143	0.29123741
yhcO	-0.825292813	0.41390176	0.046159262	0.165639948
flhC	-0.82569048	0.408548953	0.04327646	0.160428896
pflB	-0.826485418	0.681914835	0.225509777	0.456624913
yeaY	-0.829288608	0.322348213	0.010092368	0.060632909
pdxH	-0.832642353	0.301875546	0.005811519	0.041597702
evgA	-0.833570431	0.493175189	0.090987478	0.258953448
ycgX	-0.835265648	0.27531604	0.0024146	0.023264299
mdaB	-0.835638077	0.276655789	0.002523599	0.023858946
yjfY	-0.835876565	0.701185921	0.233226047	0.465856377
tufA	-0.836165962	0.845327413	0.322583767	0.562293034
purA	-0.837219739	0.802153925	0.296617467	0.535475176
rlmE	-0.83855582	0.700146713	0.231039335	0.46334118
greB	-0.83971378	0.291065124	0.003914525	0.031925129
rsxB	-0.840820146	0.463656141	0.069761474	0.215971681
ptsI	-0.841016779	0.668373465	0.208281957	0.434764354
yqfB	-0.841999529	0.456374228	0.06504102	0.206435222
ydiE	-0.842027551	0.655543669	0.19897682	0.422159366
clpA	-0.842044535	0.702700586	0.23080112	0.4630935
ibpA	-0.843207554	0.697788878	0.22689373	0.458207332
folE	-0.843516173	0.35777432	0.018389702	0.091766657
yebY	-0.843635242	0.593831948	0.155414673	0.365489836
yobD	-0.844951274	0.272538558	0.001933248	0.019708386
yedK	-0.845491276	0.485760749	0.081762343	0.241635854
gpr	-0.846951113	0.533160117	0.11216219	0.295445386
ybhB	-0.847675516	0.294149952	0.003954354	0.03199144
yggU	-0.848055794	0.499103438	0.089289344	0.256009292
fliE	-0.849903597	0.336087044	0.011444674	0.0663824
ybjX	-0.855794223	0.364324528	0.018824475	0.092338281
sufE	-0.857458705	0.360426777	0.017359231	0.08870787
sfsA	-0.858884869	0.608806451	0.158312931	0.369343025
ydiL	-0.86322557	0.275579023	0.001733795	0.018464291
ykgE	-0.863594943	0.392425773	0.02776024	0.119603081
yajQ	-0.863855406	0.394815312	0.028669694	0.121703001
gcvH	-0.864045045	0.452065148	0.055962366	0.187953471
folA	-0.864623132	0.491516809	0.078561937	0.234581761
recX	-0.864870203	0.477391279	0.070038904	0.216167474
msyB	-0.865060145	0.403404002	0.032000731	0.131689042
agp	-0.865179816	0.546429829	0.11334582	0.297514354
lacZ	-0.867625024	0.596646494	0.145899369	0.349967769

secE	-0.871317029	0.712726618	0.22151397	0.452327717
rho	-0.874865287	0.760252521	0.249832557	0.484450976
eno	-0.876078181	0.614497953	0.153960385	0.363526107
ypeC	-0.88459903	0.334362992	0.00815402	0.052085091
pepD	-0.88559241	0.319474553	0.005570824	0.040016757
ackA	-0.88563412	0.61908129	0.152555808	0.361931946
cusF	-0.888104789	0.378844854	0.019065692	0.092956762
ybcV	-0.888134245	0.456875061	0.0519036	0.179242802
uspF	-0.88825748	0.582014088	0.126965377	0.318755738
yccJ	-0.889389825	0.529091259	0.092767533	0.259660471
yqeK	-0.889993581	0.47343376	0.060125896	0.195868482
folX	-0.891346531	0.445068958	0.045207827	0.164019302
yhhH	-0.892791164	0.440959025	0.042902507	0.159762217
yfiL	-0.893942919	0.77298759	0.247485811	0.481726239
ykfB	-0.894762811	0.346507557	0.009816365	0.059681723
mdh	-0.895710147	0.813641856	0.270955254	0.508372098
stpA	-0.899438627	0.335090989	0.007271178	0.048019975
yjcB	-0.901181564	0.726912228	0.215071883	0.443446489
yebB	-0.901780451	0.603876342	0.135353515	0.332848571
sieB	-0.902771876	0.373574956	0.015667301	0.082489419
ribC	-0.90824709	0.289310637	0.001693236	0.018317781
aroG	-0.908876368	0.468053759	0.052158838	0.179816593
phoP	-0.910722155	0.59933409	0.128622418	0.321118553
cyoE	-0.910877747	0.774279666	0.239427258	0.471496507
mutT	-0.914198244	0.265085077	0.000563297	0.0087346
ribE	-0.914272448	0.589757062	0.121080887	0.309565258
yfcE	-0.916401193	0.303316488	0.002517163	0.023858946
can	-0.917090683	0.602154988	0.127755117	0.31982398
yiaW	-0.918837965	0.50146097	0.066903462	0.210678063
insA	-0.919262628	0.821822544	0.263325402	0.50049183
ybfE	-0.920708887	0.712052708	0.195999044	0.418866512
trpR	-0.92286969	0.458486851	0.044129395	0.16181113
ymgD	-0.925908097	0.314118658	0.003202118	0.028041105
deaD	-0.930047163	0.772866624	0.228831843	0.460286074
serS	-0.93243011	0.611902924	0.127553602	0.31982398
yacC	-0.932690198	0.560701274	0.096225366	0.266617571
tolC	-0.933663481	0.662924454	0.159012356	0.36942662
ariR	-0.935273972	0.620595079	0.131795298	0.326415717
xseB	-0.938398973	0.719268227	0.192009364	0.412968461
yafO	-0.943240669	0.394541954	0.016815217	0.0865855
cyoB	-0.943589868	0.61373894	0.124184372	0.313911887
ybcJ	-0.944579948	0.40795198	0.020589988	0.097582495
yciE	-0.946326794	0.339664062	0.005335186	0.038737674
ycjD	-0.948348681	0.350475037	0.006812056	0.046515543
fliQ	-0.949078169	0.496437794	0.055904852	0.187953471
hicB	-0.951833028	0.331209186	0.004055499	0.032625037
yjgA	-0.953066475	0.290347709	0.001028908	0.012859762
ratB	-0.953314291	0.672037999	0.156032514	0.365719093
ykgH	-0.953401149	0.396916076	0.016304728	0.084658051

csgF	-0.955783842	0.395714005	0.01572053	0.08263513
secB	-0.961697808	0.677149715	0.155545259	0.365489836
ybjN	-0.967027244	0.673257031	0.150905094	0.358988723
ydaQ	-0.967273181	0.365387442	0.00811485	0.052014371
ecpR	-0.967619215	0.260373088	0.000202178	0.004094847
yidD	-0.96790188	0.672683683	0.150188408	0.358127941
yibL	-0.968119063	0.310509726	0.001821828	0.019031654
fadR	-0.973652202	0.228369631	0.0000201	0.000766483
yajD	-0.974266742	0.758645103	0.199065207	0.422159366
rppH	-0.975206755	0.522947471	0.062205606	0.199780455
yiaG	-0.977762094	0.649425521	0.132175019	0.327155457
aroK	-0.982101902	0.592347418	0.097320914	0.269098993
greA	-0.984845436	0.577903581	0.088349497	0.254944188
yehS	-0.989147994	0.261386825	0.000154185	0.003346475
ylaC	-0.989258289	0.65301129	0.129793275	0.323268421
tusA	-0.989521765	0.48169641	0.039952121	0.152300955
yegP	-0.98967946	0.291203924	0.00067737	0.009842458
dadA	-0.991892235	0.706859884	0.160546055	0.37184419
ycfP	-0.994221799	0.608053485	0.102030311	0.276900479
mgsA	-0.996518084	0.498775949	0.045724543	0.164880193
nudJ	-0.996881289	0.562258518	0.076229609	0.229999203
bcsF	-0.997952298	0.44062953	0.02352293	0.106459717
clpP	-0.998852446	0.684867403	0.144713498	0.34878113
dicA	-0.998949011	0.551966632	0.070326864	0.216559535
rbsK	-1.000992187	0.799875577	0.210775862	0.43788123
argR	-1.00211155	0.774438933	0.195670965	0.418391783
ndk	-1.003534613	0.336817767	0.002887582	0.026078676
yciU	-1.005515843	0.372840252	0.006998661	0.046911083
rnlB	-1.005722708	0.649878276	0.121729349	0.310436755
paaB	-1.006907013	0.423957904	0.017548166	0.089330411
cyaY	-1.006930922	0.502811444	0.045220085	0.164019302
sfsB	-1.009401561	0.481930742	0.036215902	0.143138693
yciH	-1.009441612	0.552126483	0.067507593	0.211919246
yahM	-1.009527547	0.426389596	0.017903025	0.090343142
yqfA	-1.009839676	0.557562616	0.07011482	0.216236461
pspF	-1.010436925	0.352194956	0.004118103	0.03269036
fumA	-1.016208347	0.420128662	0.015571683	0.082389102
ykgD	-1.017078852	0.236313832	0.0000168	0.000657649
metJ	-1.017123603	0.626995385	0.104756541	0.281372027
atpF	-1.017799292	0.729146844	0.162751545	0.374588361
ycgJ	-1.0209965	0.432397848	0.018213779	0.091523534
ppiC	-1.021350485	0.452056667	0.023862137	0.107267742
tsx	-1.022299763	0.807544538	0.205535969	0.430504185
ccmD	-1.022929234	0.458850474	0.02579194	0.113422727
yaaI	-1.025664095	0.262630244	0.0000941	0.00228814
csgC	-1.026783799	0.3878818	0.00811718	0.052014371
pgk	-1.02731084	0.792590108	0.19492591	0.417462016
yphA	-1.027442182	0.367951384	0.005232974	0.038225217
ribA	-1.027705467	0.305592255	0.000771015	0.010693737

marA	-1.027726526	0.302864444	0.000690421	0.009918973
ymdF	-1.030473279	0.330953344	0.001847878	0.019127906
seqA	-1.030608878	0.704329839	0.143399667	0.34654665
rimP	-1.034742736	0.786612635	0.188361638	0.410760621
lpd	-1.036460978	0.790417985	0.189762464	0.411900695
acnB	-1.041758884	0.790040793	0.187298177	0.41004487
yhcN	-1.042661919	0.711496907	0.142798653	0.345776498
sucB	-1.042946288	0.904118926	0.248684752	0.483593615
yggX	-1.043167913	0.458611631	0.02292864	0.104946623
ydfK	-1.044977203	1.24579394	0.401579187	0.632283611
lacA	-1.045157255	0.457166871	0.022244818	0.102748661
insB1	-1.048610625	0.779612609	0.178612118	0.398814779
yeeD	-1.050114944	0.583389404	0.071856845	0.219970586
yccX	-1.0510946	0.520300601	0.043365969	0.160613227
yhfA	-1.056659371	0.754448586	0.161342176	0.373046027
mdtJ	-1.057763898	0.310143913	0.0006483	0.00965752
fldB	-1.061528974	0.603787117	0.078727441	0.234902201
ygcO	-1.064143657	0.789771324	0.177849087	0.397550811
yfgJ	-1.064573713	0.692357382	0.12414468	0.313911887
mliC	-1.064979964	0.348326236	0.002232505	0.022089757
ymgA	-1.066492235	0.367546356	0.003711987	0.030971664
atpB	-1.069813156	0.883761017	0.22607822	0.457024423
def	-1.071408836	0.56983563	0.060079897	0.195868482
crl	-1.072519212	0.30963158	0.000532491	0.008332046
rutR	-1.075983688	0.585424059	0.066068883	0.209028276
ypjF	-1.076363554	0.627892959	0.086483392	0.250814262
yijD	-1.079024493	0.580650285	0.063125919	0.202574987
yfeD	-1.080499094	0.317104286	0.000655865	0.009698134
pck	-1.082216259	0.871877102	0.214513915	0.443099451
deoB	-1.087471397	0.835601722	0.193113665	0.414472668
osmC	-1.091689257	0.265209743	0.0000385	0.001223697
ycbL	-1.095789479	0.634237986	0.084037354	0.246686533
deoC	-1.099563057	0.791928494	0.164996241	0.378245215
ydiT	-1.101875344	0.380155435	0.003749686	0.031019433
yoaB	-1.102985242	0.272078164	0.0000504	0.001495019
yqhH	-1.10414768	0.414174457	0.007678253	0.04991519
bfd	-1.105795514	0.38253524	0.00384382	0.031475661
sucA	-1.106673456	0.826925504	0.180799035	0.400808423
talB	-1.108210734	0.803168002	0.167648415	0.381939419
yafP	-1.108220114	0.42603603	0.009288862	0.057338128
yfjM	-1.112808104	0.778634849	0.152952966	0.362365683
pspD	-1.11328225	0.628824094	0.076657333	0.230944518
glnK	-1.115697862	0.59589786	0.061165505	0.198174274
yaiY	-1.115895609	0.308125969	0.000292836	0.005372707
trxC	-1.116572025	0.513894984	0.029798157	0.125046943
yegR	-1.117070478	0.393091662	0.004486585	0.03469798
ydcY	-1.118102072	0.622500007	0.072471	0.221137889
yifE	-1.120758721	0.854735194	0.189778373	0.411900695
ppa	-1.121219577	0.71519845	0.116950193	0.303169594

dapA	-1.121799502	0.698365082	0.108203082	0.28832729
yhhL	-1.12318095	0.46147217	0.014936778	0.080722588
ykfI	-1.124244981	0.631449225	0.075007212	0.227159876
yahO	-1.130191316	0.278749023	0.0000502	0.001495019
yoeB	-1.131498992	0.724299506	0.118241432	0.305102671
ybiJ	-1.134145496	0.434266879	0.009011106	0.056225402
csrC	-1.137321804	1.006600929	0.258533681	0.494879313
ygbE	-1.137554323	0.412530644	0.005824475	0.041616647
glnH	-1.140504393	0.491986841	0.020440607	0.097582495
cutC	-1.145341862	0.337078341	0.000679175	0.009842458
nudB	-1.145575625	0.627500472	0.06790795	0.212750949
ychN	-1.146725635	0.29007717	0.0000771	0.002008634
lrp	-1.155304933	0.686306422	0.092304251	0.259280599
ykgJ	-1.159651639	0.233019007	0.000000647	0.0000522
uidR	-1.167286906	0.569814221	0.040507172	0.153402862
pspB	-1.168691847	0.374603007	0.00180965	0.019013967
ydhR	-1.1713609	0.478121376	0.014288648	0.078802285
ycgY	-1.172929028	0.277295766	0.0000234	0.000850358
iraP	-1.175428103	0.426398131	0.005839779	0.041652273
mlaD	-1.176332517	0.754103451	0.118781464	0.306207388
ygiZ	-1.178800646	0.328917404	0.000338527	0.006020414
yagN	-1.181603808	0.314068127	0.000168389	0.003559094
ygdI	-1.182862947	0.860883452	0.169438406	0.384714762
cspF	-1.189842528	0.753163601	0.114154297	0.298665519
yehK	-1.190257232	0.587224523	0.042670577	0.159578802
grxB	-1.190930117	0.592911024	0.044577954	0.162713563
ybaB	-1.194759225	0.510173689	0.019187521	0.093250315
mcbA	-1.201361364	0.455618315	0.00836992	0.053211604
panD	-1.203021367	0.386489587	0.001853921	0.019141379
atpH	-1.203806486	0.793791755	0.129386194	0.322626352
prlF	-1.205398136	0.737067976	0.101966027	0.276900479
mipA	-1.20584129	0.574745824	0.035901372	0.142092
yecR	-1.210334338	0.542526961	0.025686418	0.113301556
yqjC	-1.213763761	0.469945359	0.009800883	0.059677475
rayT	-1.214881051	0.262339606	0.00000364	0.000190838
ridA	-1.215669725	0.71839363	0.090606779	0.258179976
rrfE	-1.218085233	1.190131219	0.306077086	0.546075795
rcsB	-1.219467735	0.605038898	0.043850238	0.161655802
chpS	-1.22810363	0.645353801	0.057041486	0.190726867
fryB	-1.229778838	0.296571139	0.0000337	0.001116345
asr	-1.23230877	0.349927787	0.000428937	0.007108318
ytfP	-1.232483205	0.790865012	0.119138705	0.306541079
ydcD	-1.232526734	0.401863373	0.002161977	0.021444472
yefM	-1.2343333	0.521112305	0.01785308	0.09020386
gpt	-1.234653742	0.479511675	0.010029378	0.060430746
ydbJ	-1.235583728	0.708373116	0.081114542	0.240137904
gltI	-1.235900451	0.587363656	0.035365481	0.14121706
ompF	-1.239580998	0.817296642	0.129346406	0.322626352
yobA	-1.240445051	0.453582939	0.006242386	0.043599503

ynjH	-1.244213053	0.676671392	0.065955547	0.208833367
ucpA	-1.244472704	0.741321067	0.093206047	0.260263306
ydiZ	-1.246136708	0.302537517	0.0000381	0.001223697
yihD	-1.246351962	0.566834816	0.027892703	0.119999014
gmk	-1.246576814	0.772301309	0.106504257	0.284740189
yfdY	-1.246579756	1.017445597	0.220497871	0.450708814
yghW	-1.246713403	0.685698342	0.069038767	0.214654492
yqjK	-1.249621915	0.688414822	0.069490651	0.215298357
bolA	-1.255482987	0.644844365	0.051540125	0.178445527
rbsC	-1.256223848	0.846717406	0.13790444	0.33761081
lsrG	-1.260341481	0.374971322	0.000776137	0.010693737
ybbC	-1.263020046	0.527093012	0.016566068	0.085520737
ykfH	-1.263898801	0.432614616	0.00348311	0.029773896
yehE	-1.264488469	0.835738833	0.130274913	0.323774577
ymgI	-1.267720243	0.560061229	0.023602504	0.106664075
iscR	-1.269615495	0.541952473	0.019146338	0.093237354
yaaY	-1.273870094	0.629015785	0.0428489	0.159762217
ynfM	-1.274430297	0.441704609	0.003910971	0.031925129
yebO	-1.275736422	0.679584877	0.060486718	0.196448014
accB	-1.276615847	0.66171621	0.053699862	0.183570551
yaeP	-1.280864934	0.822604799	0.119450496	0.306952039
groS	-1.286349816	0.502230203	0.010428868	0.062280087
adk	-1.287026849	0.645087189	0.04602971	0.165322009
moaD	-1.290225661	0.449472309	0.004097788	0.03268949
rbsA	-1.292715318	0.787467827	0.100670786	0.275344149
queD	-1.293156698	0.248241568	0.00000019	0.0000194
dcuA	-1.294298046	0.963201871	0.179030744	0.399278821
bamD	-1.295638771	0.776778656	0.095322889	0.265026518
uxaB	-1.296962665	0.61330057	0.034453306	0.138671982
fabI	-1.29947116	0.651015021	0.04592637	0.165097734
eutS	-1.300754493	0.403518477	0.001266204	0.0147736
ypjJ	-1.302010106	0.62329957	0.036716779	0.144328758
yjeT	-1.302519172	0.554875141	0.018904466	0.092369789
yqiB	-1.305517166	0.592848233	0.027657522	0.119308274
tusE	-1.305807766	0.392298172	0.000872804	0.011498307
ybiA	-1.31107669	0.391477376	0.000810896	0.010948455
ilvN	-1.318800903	0.706132025	0.061812171	0.199213425
frdD	-1.323393046	0.783213919	0.091086222	0.258953448
hybG	-1.326531594	0.94170405	0.158938629	0.36942662
rpoH	-1.32743225	0.87131873	0.127639925	0.31982398
nrdH	-1.327945792	0.366023376	0.000285586	0.005270004
rnk	-1.329110035	0.680121023	0.050674522	0.176404667
ykgI	-1.33264255	0.333027697	0.0000629	0.00177618
yjbL	-1.333643764	0.746581568	0.074045255	0.224921514
secG	-1.335690212	0.734084086	0.068830154	0.214569369
mazE	-1.33681944	0.365649354	0.000256167	0.004832451
rrfB	-1.338527941	1.21318466	0.269889283	0.507472303
frdB	-1.347035701	0.98222476	0.17024628	0.384904479
yceK	-1.348722939	0.666855381	0.043123496	0.160155984

ydeI	-1.350346113	0.401098506	0.000760956	0.010629681
slyB	-1.352223036	0.81104147	0.095461238	0.265097448
dps	-1.353022942	0.714266126	0.058187315	0.192070474
yiiE	-1.354553354	0.555896941	0.014822024	0.080642196
sdhB	-1.360514744	0.719391824	0.058597479	0.192636827
yibN	-1.361230298	0.894061704	0.127877866	0.319853125
ydfO	-1.362088818	0.445628297	0.00223894	0.022099271
yacG	-1.370115155	0.545897329	0.012078575	0.069361603
iscA	-1.371547582	0.744768817	0.065537351	0.207672124
ydhO	-1.372039965	0.68484794	0.045131928	0.163994234
cmk	-1.372843914	0.740393103	0.063709196	0.20412224
ydgC	-1.373737703	0.414151516	0.000909919	0.011715654
yeaO	-1.375577814	0.331214933	0.0000328	0.00111259
mdtI	-1.377472784	0.603351024	0.022428182	0.103241243
yrhB	-1.390499442	0.312355032	0.00000852	0.000378004
cyoA	-1.398387981	0.860445136	0.104121796	0.280413402
rpoS	-1.400018772	0.847621056	0.098594983	0.271356895
ydhI	-1.403988027	0.367232636	0.000131754	0.002971454
osmE	-1.405033189	0.834276385	0.092155783	0.259257256
flxA	-1.407263358	0.253919647	2.99E-08	0.00000503
gltA	-1.407546313	0.929149603	0.129803842	0.323268421
yneG	-1.407956319	0.480155615	0.003364706	0.028900673
ybaQ	-1.411185526	0.283918183	0.000000668	0.0000529
ycgZ	-1.412601016	0.344352158	0.0000409	0.00127549
frr	-1.415375731	0.684395583	0.038633749	0.148963176
yecJ	-1.423841933	0.382979376	0.000200963	0.004094847
yebG	-1.42443681	0.430425927	0.000935076	0.011945893
ybfK	-1.433322024	0.743174247	0.053774493	0.183570551
ybjC	-1.438168802	0.301317544	0.00000182	0.000111055
yjbD	-1.443487216	0.761764313	0.058102169	0.192070474
rpoZ	-1.444751609	0.481026979	0.002669171	0.024828212
gapA	-1.445409265	0.852961494	0.090155619	0.257875425
yggL	-1.44980512	0.753706416	0.054408826	0.184971243
ybdJ	-1.454924439	0.390391532	0.0001939	0.003993748
nlpD	-1.45839951	0.628294559	0.020275865	0.09712408
fabZ	-1.460531952	0.514762655	0.004549798	0.035037619
ypfN	-1.464767865	0.447440187	0.001061636	0.013146698
ymgE	-1.465060713	0.451679518	0.001180358	0.014099149
nuoB	-1.470468115	0.686074677	0.032088519	0.131811364
rplP	-1.479070108	0.972234456	0.128182034	0.3204154
feoC	-1.479895004	0.74027552	0.045596043	0.164880193
ybeL	-1.480238225	0.694683711	0.03310489	0.134586547
yeaQ	-1.480589204	0.576486457	0.010219916	0.061304311
arfA	-1.483818813	0.496950266	0.002827916	0.025631676
yjdI	-1.48826256	0.962049402	0.12187023	0.310599823
ftsB	-1.491024144	0.375856312	0.0000728	0.001944517
yeiW	-1.501882024	0.442191562	0.000682657	0.009842458
ygiN	-1.503716719	0.694525044	0.030379968	0.126567523
yfiM	-1.524149213	0.447435688	0.000658233	0.009698134

bhsA	-1.525973109	0.341481571	0.00000787	0.000356991
rsfS	-1.532229749	0.889654495	0.085019703	0.247717609
ynfO	-1.532895044	0.255333444	1.93E-09	0.000000557
yciI	-1.535432201	0.844790496	0.069135975	0.214654492
luxS	-1.538996006	0.662783421	0.020231887	0.09712408
yoaC	-1.539148869	0.356864727	0.0000161	0.000637497
ynaK	-1.540240257	0.361448649	0.0000203	0.000766816
rnpA	-1.540356176	0.668406756	0.021193518	0.099370769
smpB	-1.552585748	0.764375575	0.042236557	0.158503581
kilR	-1.552654698	0.419186999	0.000212254	0.00424193
yrbL	-1.554782488	0.830900456	0.061317105	0.198347079
sucD	-1.555517228	0.973616779	0.110116688	0.29123741
hspQ	-1.556543381	0.954284902	0.10286717	0.277812336
aaeX	-1.558916192	0.442725692	0.000429633	0.007108318
yaiE	-1.561018979	0.939920628	0.096753877	0.267714464
tig	-1.572123095	0.834996354	0.059728783	0.195401212
pptA	-1.575527218	0.913436054	0.084557066	0.247522707
clpS	-1.580287922	0.831060908	0.057232521	0.190726867
ynbE	-1.582720356	1.206013458	0.189399666	0.411900695
yebF	-1.583295075	0.304005163	0.000000191	0.0000194
yaeH	-1.587189999	0.730780733	0.02986245	0.125186617
ykgM	-1.59133859	0.748647977	0.033535194	0.135925278
ralR	-1.595876506	0.387644655	0.0000384	0.001223697
racC	-1.599475733	0.327637633	0.00000105	0.0000692
mlaB	-1.602295772	0.692183619	0.020621576	0.097595899
prs	-1.604702619	0.908196744	0.077243171	0.23228624
yjdM	-1.607523002	0.713226317	0.02420403	0.107968693
ppiB	-1.612528778	0.664943612	0.015305818	0.082167002
ycgL	-1.617399528	0.833784702	0.052400506	0.180318709
fbaA	-1.619513762	0.91808164	0.077728724	0.233475342
hinT	-1.620940593	0.760155621	0.032975626	0.134467273
dusB	-1.621346507	0.904454134	0.07303301	0.222348612
ydhL	-1.621547721	0.503466009	0.001278471	0.014871362
nusB	-1.623229719	0.954565796	0.08903877	0.255835955
relB	-1.629406366	0.986735434	0.098675234	0.271356895
ynfB	-1.629543641	0.331736683	0.000000901	0.0000628
yfaE	-1.630317393	0.451382435	0.000304042	0.005504112
mraZ	-1.634203089	1.000152908	0.102268668	0.276900479
grxC	-1.635469062	0.789297907	0.038260266	0.148358554
tatA	-1.637679321	0.894976281	0.067271206	0.211506122
yodC	-1.641441081	0.739013098	0.02634244	0.114966951
alpA	-1.641997635	0.633734789	0.009570153	0.058715361
yoaF	-1.642406664	0.592065708	0.005536697	0.039985053
yfhL	-1.642893961	0.96219201	0.087738595	0.253543815
yhcB	-1.642999141	0.923560685	0.075242481	0.227531009
cpxP	-1.645710375	0.865413977	0.057217548	0.190726867
ybgE	-1.646502553	0.686987894	0.01654355	0.085520737
zapB	-1.651077082	0.89967152	0.066476076	0.209495643
slyA	-1.653205631	0.510862253	0.001211714	0.014311782

ymgC	-1.657089111	0.871548311	0.05726058	0.190726867
rof	-1.657997821	0.414678901	0.0000638	0.001788823
racR	-1.660039751	0.652446843	0.010948775	0.064341916
yncJ	-1.674618712	0.829912357	0.043609389	0.161218958
fldA	-1.675890608	0.705766953	0.017569568	0.089330411
cydB	-1.686452567	0.870059069	0.05258424	0.180666022
yajC	-1.689333331	0.737644845	0.022011367	0.102137802
skp	-1.693584708	0.737182413	0.021597366	0.100636236
nuoA	-1.693927873	0.802627416	0.034817165	0.139403565
ryeA	-1.695361232	1.015367541	0.094978335	0.264272906
hokB	-1.70058305	0.945431922	0.072060348	0.220236248
uspG	-1.703904949	0.82104333	0.037959447	0.148060182
rbfA	-1.704863046	0.736058759	0.020547073	0.097582495
yecH	-1.71300655	0.57956474	0.00311983	0.027613781
sspA	-1.718484073	0.976537688	0.078446404	0.234502043
isrC	-1.71926183	0.78019949	0.027551203	0.119083734
soxS	-1.722493665	1.00166059	0.085498255	0.248492769
yfbV	-1.725251885	0.66756334	0.009754808	0.059486649
hipB	-1.730536133	0.531858657	0.001138917	0.013848818
ypeB	-1.730683884	0.585591998	0.003122202	0.027613781
yhdV	-1.73227126	0.919164608	0.059481857	0.195385076
glnB	-1.737320824	0.557295072	0.001824437	0.019031654
chbB	-1.740220621	0.342825881	0.000000385	0.0000353
essQ	-1.740928846	0.882775846	0.048597338	0.171897803
sucC	-1.746504234	0.769769946	0.023276525	0.105900142
yqiC	-1.750259439	0.842822001	0.037832435	0.147960422
rpoE	-1.750272586	0.993623594	0.078153026	0.234053238
yafQ	-1.759990727	0.547935706	0.001317966	0.015158485
rzoR	-1.773584322	0.601547251	0.003194537	0.028035533
cydA	-1.78008729	0.982511167	0.070021265	0.216167474
yebV	-1.786262393	0.639748581	0.005236201	0.038225217
yhfG	-1.788457041	0.783059087	0.022375277	0.103115291
yafN	-1.789020752	0.287790497	5.09E-10	0.000000205
relE	-1.797759916	0.658963704	0.006368827	0.043950349
sdhD	-1.805006101	0.843566335	0.03237641	0.132693978
iscU	-1.808234358	0.774777105	0.019602171	0.094771213
yfeC	-1.812446248	0.926949748	0.050550037	0.176226685
yciS	-1.814389825	0.427850788	0.0000223	0.000817738
kgtP	-1.816115795	0.963916084	0.05955124	0.195401212
yobH	-1.821573547	1.002370697	0.069176491	0.214654492
yodD	-1.828815936	0.776025597	0.018440925	0.091903719
ycdZ	-1.831483842	0.434206383	0.0000246	0.00088055
rpsG	-1.839673312	1.020820512	0.071521558	0.219568464
cspH	-1.841107906	0.815033809	0.023887466	0.107267742
dksA	-1.843468349	0.908620372	0.042472011	0.159053349
erpA	-1.854888485	0.578077553	0.001333277	0.015291018
uspE	-1.855803946	0.79001427	0.01882004	0.092338281
ylcI	-1.857877037	0.456328124	0.0000467	0.0014081
rem	-1.864066197	0.290717502	1.44E-10	8.29E-08

ymgF	-1.876669029	0.646321932	0.003688833	0.030895893
deoD	-1.879196615	0.999008012	0.059963403	0.195851341
ibaG	-1.883630797	0.903388839	0.037062763	0.145405611
yniA	-1.883661203	0.827510935	0.0228282	0.104724368
fis	-1.887965651	1.004545593	0.060186818	0.195868482
thrS	-1.892827795	0.801948261	0.018260818	0.09154307
yheU	-1.91179123	1.019125636	0.0606672	0.196875793
ypdI	-1.919747126	0.462672982	0.0000334	0.001112885
ompC	-1.924358141	1.16526964	0.098651346	0.271356895
ykfF	-1.931240194	0.73476692	0.008579484	0.054265307
hpf	-1.950074344	0.791102973	0.013701059	0.076291274
ydfC	-1.954697455	0.489575347	0.0000653	0.001819214
hupA	-1.960671844	0.964835944	0.04214052	0.158448422
rplA	-1.961638068	0.991060443	0.047778591	0.169492244
pspE	-1.970026879	1.015730953	0.052438485	0.180318709
frdC	-1.972803999	0.793165706	0.012873371	0.072907843
ygdR	-1.976046523	0.723751243	0.006327976	0.04374322
rcbA	-1.988926108	0.612790156	0.001171669	0.014099149
ryfA	-1.998644984	0.839715113	0.017305757	0.088546691
fusA	-2.000716669	1.110639932	0.071638453	0.219760209
ompA	-2.012479451	1.034623488	0.051759071	0.178896719
yhdU	-2.017203298	0.66070067	0.002264698	0.022190741
rplS	-2.020542072	1.032211912	0.050290162	0.175472242
ogrK	-2.023676444	1.065040382	0.057420834	0.191102973
yceQ	-2.034749389	0.574804004	0.000400277	0.006818218
yifL	-2.039957122	0.773557735	0.008361562	0.053211604
rplI	-2.041100206	0.963926184	0.034218622	0.138278855
rplD	-2.043551294	1.107301672	0.064961418	0.206435222
cyoD	-2.047397501	1.011086902	0.042872795	0.159762217
crp	-2.051202785	1.034998772	0.047497062	0.169370362
yjfN	-2.055039853	1.166308494	0.078068677	0.2339742
yjdJ	-2.062388764	0.346668574	2.7E-09	0.00000068
yejG	-2.063154691	0.847258157	0.014887852	0.080722588
bamE	-2.072426347	1.064175001	0.05148095	0.178393645
flhD	-2.074958987	0.793779836	0.008948293	0.055936352
tatE	-2.07623129	0.965620819	0.031543214	0.130471264
yjjZ	-2.077304279	0.927066519	0.025043673	0.111100339
icd	-2.084314691	0.948050614	0.027911586	0.119999014
dinJ	-2.091965736	0.556233657	0.000169271	0.003559094
rnpB	-2.10109878	1.044620215	0.044288308	0.162096009
feoA	-2.110938669	0.86061163	0.014173518	0.07827427
chaB	-2.113759948	0.674958159	0.001738031	0.018464291
dctA	-2.121826086	1.134327773	0.061406347	0.198462522
ymjA	-2.13393383	0.743068882	0.00408161	0.032628631
hns	-2.144124454	0.899473303	0.017136983	0.087905971
trxA	-2.145566275	0.908768021	0.018227625	0.091523534
rplL	-2.14758794	0.990836148	0.030200548	0.12621078
tsf	-2.150484343	0.928124292	0.020502537	0.097582495
yeeT	-2.160914075	0.991318625	0.029269467	0.123212554

dicC	-2.162814375	0.643448836	0.00077581	0.010693737
ydcX	-2.166707235	0.495654787	0.0000123	0.000513782
yjbJ	-2.171181994	0.760690446	0.004314191	0.033694092
spr	-2.183085267	0.945267843	0.020916486	0.098300179
yccA	-2.186920123	1.031209149	0.033944208	0.137445104
hfq	-2.187437544	0.941902893	0.020213563	0.09712408
gcvB	-2.205356904	0.892792431	0.013504555	0.07550954
fur	-2.21054772	0.977804483	0.023776248	0.107184801
yeeX	-2.213122181	0.717414456	0.002036489	0.020451012
bdm	-2.213746641	0.955862997	0.0205601	0.097582495
hha	-2.21449952	0.679020655	0.001108972	0.013607658
holE	-2.221218904	0.604703182	0.000239496	0.004582204
iscX	-2.227446435	1.076243737	0.038485255	0.148959708
ydcA	-2.235446386	0.745918848	0.002727375	0.025253244
aspA	-2.237629311	1.115533544	0.044868776	0.163332056
uspA	-2.24711626	1.09893464	0.04087326	0.154644189
ytjA	-2.254492493	0.638289061	0.000412294	0.006930509
ydhZ	-2.255533421	0.719547776	0.001720568	0.018424229
minE	-2.262372875	0.821998491	0.005918127	0.041988536
hupB	-2.264829079	0.826827372	0.006159262	0.043312308
cptB	-2.268053413	1.175919163	0.053761977	0.183570551
dsrB	-2.272530536	0.824992018	0.005876229	0.041838337
ycaR	-2.280750764	0.629967985	0.000294121	0.005372707
osmB	-2.289031792	1.008411485	0.023211331	0.105900142
yeiS	-2.289329759	0.530076399	0.0000157	0.000626862
dam	-2.29546392	0.816916112	0.004955476	0.037253735
rttR	-2.302230645	0.978288501	0.018606371	0.092338281
cedA	-2.316878615	0.474818461	0.00000106	0.0000692
yibT	-2.318155778	0.765294641	0.002452816	0.023576233
ymcE	-2.326909861	0.961751997	0.015544042	0.082350786
ydfZ	-2.329778745	0.632329165	0.000229206	0.004470074
sodB	-2.332203015	1.001382638	0.019859973	0.095902764
rpsC	-2.340608849	1.159101802	0.043452949	0.16078786
ymgJ	-2.36299628	0.68823899	0.000596073	0.009080555
ompW	-2.363900865	1.101486029	0.031864878	0.131397869
ihfB	-2.369016851	0.932433935	0.011063692	0.064730617
secY	-2.372632426	1.158001343	0.040471601	0.153402862
ycbK	-2.37449281	0.812889076	0.003488495	0.029773896
tomB	-2.379866789	0.956860795	0.012876713	0.072907843
marB	-2.397370089	0.744527173	0.00128195	0.014871362
crr	-2.405540577	0.961239344	0.012330545	0.070407934
yaiZ	-2.405633447	1.036371415	0.02027563	0.09712408
yaiA	-2.415243673	0.760235213	0.00148823	0.016688842
rpsA	-2.416702248	1.103967918	0.028589172	0.12160098
yncH	-2.423976754	0.803460318	0.002553559	0.023918137
rpsB	-2.42679993	1.118992194	0.030102646	0.126062637
ynaJ	-2.428302412	0.710549177	0.000631989	0.009484533
trmD	-2.44738709	1.064763376	0.021531828	0.100606469
sibA	-2.448391586	1.249907355	0.050129366	0.175214069

psrO	-2.449373562	0.819591513	0.002803233	0.025487952
msrB	-2.449538715	0.853977777	0.004125706	0.03269036
ygeI	-2.4509789	1.042037017	0.018667581	0.092338281
yjdO	-2.451852826	1.082325415	0.023490817	0.1064337
rpsI	-2.452215121	1.107581478	0.026826933	0.116577316
sdhC	-2.453065038	1.05891648	0.020526607	0.097582495
yedF	-2.45442315	0.813355906	0.002547432	0.023916244
sibB	-2.4639983	1.232543932	0.045595804	0.164880193
atpE	-2.464289783	1.121935641	0.028058842	0.120387107
glgS	-2.470821295	1.07038204	0.020979422	0.098481312
yeaC	-2.480274508	1.019397108	0.014971001	0.080799374
ydaF	-2.485707093	0.753740553	0.000974356	0.012330646
yciG	-2.491743283	0.665129173	0.000179494	0.003735148
npr	-2.50269541	1.15750074	0.030606271	0.127116785
rseA	-2.507408334	1.012421073	0.013262347	0.074504838
ydfA	-2.515071202	0.772734856	0.001134858	0.013841153
ydfD	-2.518869477	0.79814618	0.001600037	0.017599178
yciN	-2.523915388	0.87677083	0.003993827	0.032246158
slyX	-2.534895722	0.71055269	0.000360405	0.006300591
cnu	-2.535354135	0.839802439	0.002536199	0.023874841
rpsE	-2.551465658	1.081147487	0.018276868	0.09154307
rplR	-2.560480346	1.189585248	0.031364735	0.129866089
sibE	-2.574863411	1.209510914	0.033266939	0.13510929
rplY	-2.577750785	1.233368445	0.036617018	0.144076902
ybeD	-2.589283518	1.115055151	0.020226878	0.09712408
dinI	-2.598203778	0.571108637	0.00000538	0.000266303
cspI	-2.601840198	0.673887589	0.000112951	0.002602194
rplB	-2.607253408	1.269187628	0.039949617	0.152300955
yciX	-2.608545122	1.179754117	0.027029453	0.117331078
grxA	-2.611689694	0.450399715	6.69E-09	0.00000142
rpsK	-2.624219249	1.107158918	0.017777111	0.089932578
rbsB	-2.629573667	1.181581833	0.026049774	0.113936011
ompX	-2.635708107	1.090721522	0.015671333	0.082489419
nlpI	-2.639990445	1.089728446	0.015409327	0.082269422
yhbY	-2.650685961	0.919396252	0.003938169	0.03199144
hicA	-2.651648596	0.929046165	0.004315047	0.033694092
torI	-2.653209682	0.980894192	0.006832726	0.046515543
yddM	-2.654379993	0.52948934	0.000000536	0.0000455
rplQ	-2.662665212	1.137162729	0.019206486	0.093250315
sroH	-2.668256602	1.302033045	0.040432767	0.153402862
yecF	-2.675471122	0.635783768	0.0000257	0.000911775
yqaE	-2.677633379	0.745231457	0.000326864	0.005864673
rplV	-2.693729344	1.103238423	0.014620047	0.079758284
ybcW	-2.697124246	1.16088379	0.020161056	0.09712408
cspC	-2.719325391	0.886406342	0.002156344	0.021441279
rplC	-2.720767394	1.267586383	0.031839823	0.131397869
yciZ	-2.726327701	0.952436644	0.004203442	0.033078546
ihfA	-2.726334353	0.809533558	0.000757749	0.010621638
yebW	-2.727144823	0.640729588	0.0000208	0.000776849

grxD	-2.728692995	0.973881353	0.005080669	0.037711093
dicB	-2.736722645	0.959988954	0.004361137	0.03385752
yheV	-2.737912546	0.973600256	0.004921114	0.037133716
yhjR	-2.743316514	0.722189788	0.000145511	0.003209978
yejL	-2.745952011	1.1376439	0.015790661	0.08282673
ydaE	-2.751624093	0.556404074	0.00000076	0.0000579
tdcR	-2.783157808	1.123848331	0.013269502	0.074504838
ykgL	-2.788110212	0.526505568	0.000000119	0.000013
ahpC	-2.801728807	1.001511909	0.005149989	0.037993333
rpsH	-2.807443549	1.166449504	0.016091738	0.083930681
safA	-2.812300896	0.807159883	0.000493623	0.00787651
yojO	-2.820659984	1.051115124	0.007285752	0.048019975
yjbS	-2.827032147	1.032412543	0.006176192	0.043312308
rpoA	-2.836306501	1.257886789	0.024144719	0.107968693
rrfD	-2.856371889	0.985082656	0.003736074	0.031006634
sibD	-2.875949974	1.242962507	0.020679432	0.097755112
bssR	-2.877291894	1.177899538	0.014576592	0.079628823
cdd	-2.880502535	1.034985048	0.005383649	0.039019375
yqgC	-2.88429638	0.980462564	0.003263411	0.028393083
tpx	-2.900069365	0.962237108	0.002579279	0.02410312
gnsA	-2.919777355	0.416347937	2.34E-12	3.14E-09
cspD	-2.926204539	0.828635057	0.000413436	0.006930509
yiiF	-2.93644999	1.226681758	0.016674127	0.085968648
ydfB	-2.953631957	0.80593836	0.000247497	0.004690825
yoaG	-2.965209281	1.103579875	0.007211844	0.04788522
sibC	-2.973016432	0.711643882	0.0000295	0.001024928
yfgG	-2.974548354	1.208888059	0.013871822	0.076923824
ydiH	-2.983291558	0.584498026	0.000000333	0.0000312
rplF	-2.99266631	1.307365547	0.022074616	0.102313693
rpmA	-3.038600767	1.08648849	0.005162474	0.037993333
rplO	-3.055869238	1.318329058	0.020450043	0.097582495
ptsH	-3.056290215	0.977134148	0.001761138	0.01866067
rimM	-3.065958354	1.182689684	0.009531975	0.058570143
rpsD	-3.070869917	1.261689375	0.014935677	0.080722588
ybaM	-3.076185653	0.9199462	0.000826168	0.011076781
rrfF	-3.079672911	2.352391431	0.190477834	0.411983443
rpsR	-3.108010144	1.294419752	0.016346598	0.084712729
bsmA	-3.116112579	1.218542588	0.010550472	0.062913229
rplE	-3.184815322	1.317658032	0.015647847	0.082489419
rrfC	-3.190843912	1.053807669	0.002462444	0.023612552
udp	-3.196726213	1.320022041	0.015447169	0.082269422
grcA	-3.203431708	1.272734383	0.011836958	0.06836309
rpsS	-3.204055194	1.347885465	0.017449474	0.089056293
rplK	-3.221790817	1.113992951	0.003826632	0.031398606
ytfK	-3.235203235	1.251513335	0.009736969	0.059467689
rrfH	-3.23553534	1.062588224	0.002327137	0.022692392
ydaG	-3.247991737	1.482651662	0.02847611	0.121558519
infA	-3.257080764	1.120665156	0.003656351	0.03080262
ykgO	-3.299182037	0.992911757	0.000891397	0.011533883

rpmD	-3.316360553	1.364355994	0.015069063	0.081220039
infC	-3.340390452	1.158815896	0.003944212	0.03199144
rpmG	-3.381734178	1.297174588	0.009133935	0.056816175
metZ	-3.387662578	1.768472104	0.055417656	0.187320284
hokE	-3.402351731	1.240182621	0.006080138	0.042911743
zapA	-3.409476452	1.169406136	0.003550482	0.03017536
hokA	-3.411852778	1.033031535	0.000957391	0.012154049
ymiA	-3.449254408	0.708626032	0.00000113	0.0000724
priB	-3.4531769	1.283962358	0.00715658	0.047675107
rplU	-3.464958033	1.103283079	0.001686088	0.018297683
csrB	-3.483620964	1.202780153	0.003775824	0.031109478
glmY	-3.516731784	0.702309217	0.000000552	0.0000455
cspG	-3.52203438	1.051479842	0.00080929	0.010948455
rpsF	-3.530192757	1.316049261	0.007309231	0.0480576
lpxC	-3.541243266	1.201723565	0.003210771	0.028056024
yqgB	-3.551947294	1.360907837	0.009054653	0.056409931
rplX	-3.576280362	1.327345651	0.007053458	0.047143722
rplW	-3.581023095	1.337870431	0.007436025	0.048653536
rrfG	-3.582329999	1.127344448	0.00148463	0.016688842
yhaL	-3.584633951	1.008890684	0.000380799	0.006549878
pyrL	-3.591268661	1.200360043	0.002773141	0.02538587
yceD	-3.606887725	1.289833514	0.005167574	0.037993333
rplN	-3.616047345	1.37486057	0.00853549	0.054093838
rplJ	-3.624110203	1.364378503	0.007901848	0.051041494
hokD	-3.632071954	1.546095517	0.018814309	0.092338281
yrhD	-3.659901861	1.457021316	0.012008077	0.069054996
yfcZ	-3.660932846	0.99562001	0.000235958	0.004557709
leuQ	-3.669378515	1.84123528	0.046273545	0.165902576
bssS	-3.686381773	1.156533141	0.001435375	0.016231394
rpsN	-3.704493769	1.220032012	0.00239429	0.023161007
ydcH	-3.709963195	1.003069556	0.000216783	0.004311104
valT	-3.710461375	1.765361536	0.035569583	0.141472322
rpsP	-3.742725651	1.289905951	0.003713224	0.030971664
rbsD	-3.766630017	1.296066443	0.00365846	0.03080262
ybfA	-3.783490473	1.125630469	0.000775993	0.010693737
ryfD	-3.783602084	0.895587311	0.0000239	0.00086234
yqcG	-3.802842916	1.249395587	0.002336523	0.022729017
rpsJ	-3.828808278	1.369457031	0.005176203	0.037993333
yqfG	-3.834376885	1.42044134	0.006945911	0.046813359
yciY	-3.839150688	1.143446647	0.000786436	0.010798788
rrfA	-3.848463822	1.164889596	0.000954126	0.01215081
ynfN	-3.901076015	1.19693247	0.001117158	0.013666564
mgrB	-3.926290697	1.230356936	0.001416929	0.016067813
rpmE	-3.93760357	1.35532692	0.003669252	0.03080262
gnsB	-3.945626184	0.590869099	2.43E-11	1.96E-08
cspB	-3.969608358	1.132183947	0.000454636	0.007400676
ryjA	-4.018430301	1.15525312	0.00050442	0.007983148
rpsM	-4.073109228	1.360814298	0.00276122	0.02538587
yhfL	-4.084834342	1.080171558	0.00015579	0.003363236

leuV	-4.090912548	1.874942061	0.029117841	0.122830431
glmZ	-4.130748964	1.35240024	0.002255236	0.022190741
ecnB	-4.147445789	1.101866871	0.000167205	0.003552675
leuP	-4.148383772	1.763281542	0.018640186	0.092338281
csrA	-4.157277404	1.253481475	0.00091125	0.011715654
yjjY	-4.164417109	1.252647886	0.0008858	0.011498307
ybdD	-4.173048218	1.292656338	0.001245398	0.0146202
rpsL	-4.241668221	1.476029259	0.004056916	0.032625037
rplT	-4.247364374	1.308990408	0.001175482	0.014099149
glyW	-4.298173759	1.78367548	0.015964393	0.083482194
acpP	-4.29865152	1.19995726	0.000340534	0.00602954
rpsQ	-4.309119837	1.502444979	0.00412982	0.03269036
rpmC	-4.356886613	1.77032944	0.013852728	0.076923605
valZ	-4.445929461	1.80216614	0.01362534	0.075974444
metW	-4.469807017	1.827869876	0.014470605	0.079588328
rpmB	-4.477727975	1.411611492	0.001513574	0.016879281
rpsT	-4.503514911	1.456477797	0.001987735	0.020095075
raiA	-4.509915044	1.508186053	0.002787119	0.025398642
rplM	-4.56649561	1.472088103	0.001921834	0.019661019
valU	-4.6217416	1.811054937	0.01071185	0.063593735
rpsU	-4.776351576	1.398197358	0.0006353	0.00949891
blr	-4.815662337	1.365882846	0.000422401	0.007046418
rpmF	-4.852809371	1.499661354	0.001212442	0.014311782
yceO	-4.880932509	1.103740416	0.00000977	0.000424157
metV	-4.907964614	1.852740113	0.00807231	0.051891588
rpsO	-4.90834348	1.669831807	0.00328823	0.028481355
ychH	-4.942539134	1.412144838	0.000465218	0.007493407
tyrV	-4.943134718	1.591987922	0.001902741	0.019545458
glyV	-5.107086752	1.842319528	0.005569659	0.040016757
ybgT	-5.135582304	1.329492902	0.000112089	0.002602194
glyX	-5.204948694	1.849552047	0.004890379	0.036970896
psrD	-5.246961517	1.269984209	0.000036	0.001182798
istR	-5.448508112	1.463023035	0.000195979	0.004016082
acrZ	-5.507352091	1.327229455	0.0000333	0.001112885
valY	-5.521058056	1.857447889	0.002954918	0.026508901
micC	-5.538076664	2.176412707	0.010940631	0.064341916
lpp	-5.560534038	1.425740764	0.0000962	0.002310516
rpmI	-5.570437403	1.250605638	0.00000842	0.000377728
rmf	-5.587786458	1.142825455	0.00000101	0.000068
mokB	-5.684416775	1.55996163	0.000268489	0.005015493
ythA	-5.900745512	1.310359104	0.0000067	0.000310678
yshB	-5.955112203	1.581154495	0.00016568	0.003552675
ssrA	-6.103951839	1.539836296	0.0000737	0.001944517
cspE	-6.120411463	1.336298694	0.00000465	0.000234501
leuT	-6.165826542	3.800801911	0.104751201	0.281372027
fnrS	-6.362744648	1.818118449	0.000465902	0.007493407
rrlB	-6.437053147	3.776225036	0.088264112	0.254879985
yobF	-6.499621402	1.523185897	0.0000198	0.000766483
cspA	-6.642219096	1.669058555	0.000069	0.00188195

valX	-6.700045978	1.92590125	0.000503449	0.007983148
glyY	-6.745492611	1.968208927	0.000609786	0.009254536
tyrT	-6.81882544	1.761807316	0.000108676	0.002580725
tff	-6.829855072	1.741575022	0.0000879	0.002218915
mntS	-6.937935773	1.747375016	0.0000717	0.001930354
sra	-7.054448255	1.805466804	0.0000933	0.00228814
rpmH	-7.267505342	1.60476055	0.00000593	0.000281845
dinQ	-7.267511076	1.815500049	0.0000625	0.00177618
ssrS	-7.340105851	1.344011424	4.73E-08	0.00000715
ffs	-8.001193716	1.553080374	0.000000258	0.0000254
yneM	-8.5358958	1.589430751	7.86E-08	0.00000961
rpmJ	-9.458718994	1.91013624	0.000000735	0.0000571

Table S3. Complete list of up- and down-regulated genes in ciprofloxacin-resistant cells.

GENE	log2FoldChange	Standard error of the log2Fold Change	p-value	Corrected p-value
dadA	4.167941068	1.657006082	0.011891614	0.999867943
ryfD	3.389708708	1.522734357	0.026009694	0.999867943
astD	3.369494287	1.590722128	0.034156714	0.999867943
sufA	3.288525198	1.447071715	0.023054047	0.999867943
mmuM	3.011551065	1.730747876	0.08185397	0.999867943
dcd	2.990502895	1.531533241	0.050864604	0.999867943
rrfE	2.875678035	2.059694813	0.162664196	0.999867943
gapA	2.83005135	1.299746181	0.029451653	0.999867943
fliA	2.826089565	1.365003501	0.03841584	0.999867943
gabD	2.824941171	1.614321707	0.080131336	0.999867943
rrfB	2.792589635	2.030190339	0.168966899	0.999867943
lhgO	2.755227627	1.503713671	0.066909404	0.999867943
dadX	2.709003192	1.475996738	0.066450579	0.999867943
acs	2.686065122	1.336412365	0.044440989	0.999867943
yqjK	2.499840179	1.546565539	0.10601182	0.999867943
ybjQ	2.484325886	1.24627117	0.046216879	0.999867943
ryeA	2.470823639	1.70951499	0.148363517	0.999867943
aer	2.461468798	1.172558964	0.035796793	0.999867943
astA	2.427875994	1.445041513	0.092929549	0.999867943
glcD	2.42122486	1.328573918	0.068390691	0.999867943
pal	2.365031128	1.40210469	0.091647175	0.999867943
yhdE	2.300528461	1.32924612	0.08350499	0.999867943
ssb	2.288053654	1.159706823	0.048500269	0.999867943
aspT	2.28661841	1.894013717	0.22732158	0.999867943
rrsE	2.278582072	2.4223181	0.346878207	0.999867943
glpK	2.247448887	1.187903701	0.058498262	0.999867943
wrbA	2.24592493	1.27660386	0.078527131	0.999867943
ybeY	2.209799374	1.20402827	0.066455558	0.999867943
mhpR	2.193513235	1.331548926	0.099488269	0.999867943
ybgJ	2.162195455	1.460516803	0.138758061	0.999867943
fabA	2.130660829	1.278338308	0.095565612	0.999867943
tnaA	2.130653323	0.774666457	0.005952005	0.999867943
yodD	2.124845595	1.439054043	0.139794374	0.999867943
rrsA	2.124534597	1.677141744	0.205241434	0.999867943
glmY	2.078350838	1.334490549	0.119372834	0.999867943
sibD	2.07323534	1.605316053	0.196536905	0.999867943
yqjD	2.056273088	1.333550563	0.12308487	0.999867943
envZ	2.049526446	1.267230485	0.105807651	0.999867943
fecA	2.038453981	1.290834427	0.114295829	0.999867943
cysA	2.032695919	1.613896642	0.207851334	0.999867943
astC	2.007680982	1.362504124	0.140610103	0.999867943
yhhA	1.982414176	1.084803723	0.067633576	0.999867943
ypfJ	1.963461667	1.119789158	0.079529793	0.999867943
yjbJ	1.919408425	1.135261645	0.090890485	0.999867943

zipA	1.906507148	1.201006827	0.112416631	0.999867943
zapD	1.892894089	1.175810744	0.107427791	0.999867943
rrlA	1.816835846	1.736745948	0.295507978	0.999867943
thiS	1.811938647	1.785336016	0.310153198	0.999867943
fdoG	1.809400852	1.263597497	0.152159849	0.999867943
aldB	1.79019205	1.07528406	0.095941687	0.999867943
yraP	1.785006701	1.241462538	0.150483525	0.999867943
fliJ	1.773174659	1.318787238	0.178770776	0.999867943
tusB	1.771340341	1.262494691	0.160602599	0.999867943
tufA	1.770950827	1.37077141	0.19637883	0.999867943
yhiI	1.765920891	1.270253219	0.164464601	0.999867943
dppB	1.75379208	1.193810177	0.141813494	0.999867943
psrO	1.738880467	1.521766609	0.253174698	0.999867943
hycG	1.729075734	1.349959133	0.200251412	0.999867943
fliC	1.712005752	0.972829613	0.078438201	0.999867943
gutQ	1.706036303	1.392958926	0.220666762	0.999867943
nuoK	1.70275822	1.614761847	0.29165637	0.999867943
rrlD	1.698706047	1.685558337	0.313550385	0.999867943
yejL	1.688143901	1.492739755	0.258095911	0.999867943
rrsD	1.672651065	1.570818233	0.286953821	0.999867943
bdcA	1.668574567	1.096320842	0.128014967	0.999867943
coaD	1.66302237	1.124632354	0.139213734	0.999867943
dedD	1.642812483	1.377963904	0.233181719	0.999867943
cutA	1.640520831	1.186192959	0.166660738	0.999867943
yajO	1.640504505	1.201049443	0.171972686	0.999867943
ytjC	1.633157646	1.258230032	0.194294145	0.999867943
mog	1.62045806	1.120922138	0.148276153	0.999867943
ygaM	1.61478313	1.101878353	0.142789401	0.999867943
ypdI	1.610330471	1.033198224	0.119093898	0.999867943
osmY	1.608894724	0.947365396	0.089454327	0.999867943
ibpA	1.591234305	1.028850523	0.121956426	0.999867943
yjeI	1.586728554	1.201653198	0.186683267	0.999867943
gcvB	1.582387718	1.30499524	0.225297301	0.999867943
yncE	1.561636311	1.029008941	0.129112241	0.999867943
dps	1.5582185	1.196668652	0.19287185	0.999867943
nikR	1.54675239	1.365078038	0.257177655	0.999867943
rrsG	1.542775119	1.494336057	0.301877698	0.999867943
ydfD	1.531945568	1.324077497	0.247276093	0.999867943
ybgI	1.531646961	1.124673879	0.173242467	0.999867943
yiaS	1.530264609	1.214620251	0.207715967	0.999867943
yrdB	1.524887351	1.218090489	0.210618312	0.999867943
ubiF	1.515222131	1.294071385	0.241640856	0.999867943
lpoB	1.512463302	1.387277148	0.275607988	0.999867943
waaY	1.5106915	0.822618474	0.066292244	0.999867943
murE	1.504183687	1.187025111	0.205088163	0.999867943
yedP	1.497077106	1.096701106	0.172230096	0.999867943
dppC	1.494969949	1.471700025	0.309719114	0.999867943
putP	1.493631017	1.146843196	0.192784915	0.999867943
fadD	1.488413071	1.008839287	0.140112603	0.999867943

bfr	1.486422625	0.860350818	0.084043167	0.999867943
fabB	1.484095779	1.205213821	0.218174699	0.999867943
gabT	1.478577546	1.521393226	0.331121403	0.999867943
dgoK	1.475165623	1.199752241	0.218862461	0.999867943
ygdI	1.469593993	1.315323603	0.263871622	0.999867943
sibE	1.457981446	1.837080811	0.427404839	0.999867943
narG	1.452075436	1.036654928	0.161294315	0.999867943
folM	1.444178463	1.130440337	0.201413005	0.999867943
ydeJ	1.441278296	0.988451843	0.144808335	0.999867943
sroH	1.438328922	1.75491716	0.412444558	0.999867943
ucpA	1.436121023	1.324888582	0.278384276	0.999867943
yaaW	1.433269598	0.936252848	0.125804641	0.999867943
ydjA	1.431322782	1.254917594	0.254048435	0.999867943
yceK	1.429459161	1.208611776	0.236916907	0.999867943
glmZ	1.425516125	1.574098702	0.365143458	0.999867943
paaJ	1.41594135	1.26406657	0.262650797	0.999867943
lysC	1.411623246	0.903323904	0.11812344	0.999867943
soxS	1.407447008	1.441410497	0.328847793	0.999867943
rpsQ	1.403467143	1.76467271	0.426431455	0.999867943
rhsD	1.400584413	1.167618167	0.230324791	0.999867943
yacL	1.39065422	1.273598325	0.274872844	0.999867943
yidG	1.379753894	1.057042127	0.191791743	0.999867943
yagI	1.376429182	1.331896985	0.301400297	0.999867943
rsmJ	1.375541463	1.492091114	0.3565868	0.999867943
ilvB	1.37509131	1.041308832	0.186654359	0.999867943
yeaH	1.370315144	1.029370286	0.183117645	0.999867943
yhhM	1.369905979	1.022322881	0.18024747	0.999867943
yfcD	1.365547075	1.10822411	0.217876632	0.999867943
gadW	1.360197894	0.735605986	0.06444556	0.999867943
slt	1.35665075	1.02481796	0.185570534	0.999867943
cbpM	1.346336371	1.397048647	0.335196143	0.999867943
rrlE	1.343643187	2.299046397	0.558927683	0.999867943
cysS	1.343580538	0.937483024	0.151806825	0.999867943
lpp	1.34226786	1.534775001	0.381808028	0.999867943
yibF	1.34210253	0.920200558	0.144705763	0.999867943
yggF	1.338805056	1.280224496	0.29567276	0.999867943
dinI	1.338524166	1.127809455	0.235292559	0.999867943
cysQ	1.336729862	1.014736509	0.18773232	0.999867943
sibB	1.335321061	1.742097799	0.443377794	0.999867943
yggE	1.32958772	0.957015198	0.164739478	0.999867943
wecC	1.325580425	0.992930974	0.181870554	0.999867943
yrdD	1.324287499	1.008451381	0.18911919	0.999867943
rsmH	1.321499877	1.197443593	0.269766282	0.999867943
gcvH	1.310073662	1.345013498	0.330045275	0.999867943
sthA	1.307544283	0.987045611	0.185269005	0.999867943
ubiX	1.306763066	1.056054152	0.215938036	0.999867943
moeB	1.305166248	1.243291496	0.293825301	0.999867943
gpmM	1.30381033	0.937867342	0.164472313	0.999867943
recA	1.301176388	1.009640466	0.197484243	0.999867943

marB	1.295918617	1.426867641	0.363758709	0.999867943
dgoR	1.289453644	1.174551905	0.272280467	0.999867943
mukF	1.283577994	1.049179048	0.221174211	0.999867943
fucU	1.275897503	0.986523984	0.195898197	0.999867943
glnE	1.27540821	1.083488332	0.239143037	0.999867943
fadE	1.275239122	1.182181931	0.28071414	0.999867943
frwD	1.268989735	1.232381044	0.303148198	0.999867943
ytfB	1.264029233	0.978631364	0.196485449	0.999867943
thrB	1.263478071	1.19625839	0.290880685	0.999867943
uvrD	1.255420067	1.144090653	0.272506683	0.999867943
fepD	1.254320494	1.545276204	0.416956441	0.999867943
flgD	1.251293297	1.142354462	0.273357451	0.999867943
gnsA	1.249585731	1.078398979	0.246561539	0.999867943
yggR	1.247243894	1.062300972	0.24035636	0.999867943
rrsH	1.237315573	1.258451805	0.325506763	0.999867943
sucD	1.236716852	1.471020118	0.400504502	0.999867943
yqhC	1.230822989	0.930582667	0.185956151	0.999867943
hslO	1.229943776	1.019997414	0.227882934	0.999867943
rrlH	1.229097734	1.379091422	0.372801871	0.999867943
rrlG	1.225010174	1.883889739	0.515527022	0.999867943
bcsC	1.224663299	1.077546077	0.255734873	0.999867943
yagF	1.224310438	1.666524005	0.462553198	0.999867943
yibI	1.222846905	0.933650455	0.190281068	0.999867943
ydhR	1.220035634	1.003491622	0.224064718	0.999867943
mak	1.216456642	1.155925778	0.292631798	0.999867943
bax	1.214849911	0.765829895	0.112667143	0.999867943
yjdI	1.214334598	1.337733332	0.36400749	0.999867943
rng	1.210942832	0.904405462	0.18059086	0.999867943
dppA	1.209868457	0.983012106	0.218406369	0.999867943
fecD	1.203422749	1.403757794	0.391286511	0.999867943
bamE	1.202030529	1.452161077	0.407810459	0.999867943
emrD	1.200212948	1.09296545	0.272149805	0.999867943
glcB	1.197952571	0.926147052	0.195845141	0.999867943
fecB	1.196798986	1.219039826	0.326220343	0.999867943
insB1	1.188293393	1.313869993	0.365771489	0.999867943
lpxB	1.187801835	1.14702663	0.300412777	0.999867943
pyrL	1.186189765	1.646676843	0.471307246	0.999867943
ftsY	1.176611125	1.037619332	0.256814437	0.999867943
priA	1.176408753	1.087786326	0.279487905	0.999867943
valU	1.175935756	2.204493831	0.593738275	0.999867943
ybhB	1.175921762	1.024171361	0.250898822	0.999867943
ybdF	1.174779912	0.881530934	0.182643816	0.999867943
rimI	1.170036446	1.158517999	0.312522888	0.999867943
chaC	1.16856283	1.020363673	0.252109129	0.999867943
gyrB	1.156650232	1.038911246	0.265567067	0.999867943
ftsZ	1.15584794	1.156231045	0.317470883	0.999867943
dppF	1.154697259	1.103492325	0.295375184	0.999867943
pgaD	1.153211274	0.978838974	0.238740014	0.999867943
yihI	1.149699375	1.024517498	0.261783289	0.999867943

ynjC	1.145703127	1.40290605	0.414120364	0.999867943
tehB	1.145606898	0.833678893	0.169392496	0.999867943
ybgS	1.138162377	1.048658437	0.277766271	0.999867943
yhaM	1.138023722	1.099363925	0.300591579	0.999867943
tap	1.132960613	1.041245118	0.276558679	0.999867943
ynhG	1.132045582	0.995117459	0.255287563	0.999867943
sodA	1.124871935	0.964933178	0.243715084	0.999867943
fliE	1.124224991	1.050427483	0.284504661	0.999867943
yidF	1.12143582	1.1256494	0.319125409	0.999867943
ariR	1.119739873	1.329957122	0.399823318	0.999867943
osmE	1.118081075	1.208407147	0.354834864	0.999867943
fadM	1.117452044	0.935758902	0.232412798	0.999867943
nfi	1.116183743	1.224781931	0.362120137	0.999867943
lolD	1.114407762	1.252190366	0.373483752	0.999867943
dnaX	1.113782958	1.29139756	0.388432638	0.999867943
valT	1.112188785	2.397999606	0.642792069	0.999867943
yagG	1.110985268	1.486005676	0.454682209	0.999867943
yegR	1.108151286	0.911181875	0.223920493	0.999867943
yccJ	1.108056169	1.091759848	0.310140792	0.999867943
ycdZ	1.10685247	1.341177363	0.409210267	0.999867943
orn	1.103952645	0.937974837	0.239214119	0.999867943
mazE	1.103225369	1.059887511	0.297927016	0.999867943
ycgY	1.103098758	0.816968352	0.176940813	0.999867943
fabG	1.103041524	1.26096608	0.381705008	0.999867943
ompA	1.102672389	1.298019078	0.39560091	0.999867943
yhcF	1.10118963	0.844608295	0.192306089	0.999867943
zntA	1.100515055	1.212814067	0.364192009	0.999867943
glxK	1.099712637	1.176559853	0.349950751	0.999867943
rhsB	1.099177944	1.176977508	0.350356071	0.999867943
rrrQ	1.08807328	1.041849471	0.296315593	0.999867943
manY	1.087684006	0.980672946	0.267378406	0.999867943
diaA	1.087549208	0.961550234	0.25803938	0.999867943
rpoD	1.086215046	0.918826037	0.237135461	0.999867943
glnQ	1.08611116	1.070545794	0.310325318	0.999867943
ydeQ	1.085104974	0.770277695	0.158918217	0.999867943
asmA	1.079049898	0.908384437	0.234881474	0.999867943
fixX	1.074900785	1.369404026	0.432488411	0.999867943
eutJ	1.073060323	1.258882594	0.393997056	0.999867943
ycbU	1.072109582	1.019758406	0.293103912	0.999867943
ymjA	1.071452037	1.223149731	0.381042091	0.999867943
ypjF	1.071187218	1.0531831	0.30910826	0.999867943
ybiB	1.068999618	1.19000704	0.369018335	0.999867943
ycbB	1.068396892	0.94148075	0.256457107	0.999867943
cheB	1.06063952	1.165005819	0.362603352	0.999867943
yceH	1.058440352	1.032051043	0.305094423	0.999867943
glcE	1.056641402	1.354642214	0.435381979	0.999867943
narJ	1.051973277	1.427597689	0.461193158	0.999867943
ibaG	1.048883847	1.333792825	0.431637939	0.999867943
lptA	1.047541649	1.028134307	0.30826171	0.999867943

glpD	1.046185821	0.939553762	0.26549708	0.999867943
rpmF	1.043552724	1.853671509	0.573458518	0.999867943
ebgC	1.042710014	0.948652526	0.271703292	0.999867943
deoA	1.041923325	1.244001105	0.402278878	0.999867943
ahpF	1.040367559	1.002771703	0.29950665	0.999867943
ddlB	1.040110813	0.968615278	0.282906902	0.999867943
ybgT	1.038565263	1.845824833	0.573668839	0.999867943
yajD	1.036048315	1.132260639	0.360177799	0.999867943
nuoC	1.035803723	1.169620256	0.375838631	0.999867943
glnL	1.027063285	0.980469942	0.29485915	0.999867943
ihfA	1.026293315	1.285773667	0.424759503	0.999867943
yjbQ	1.024780723	1.023076363	0.316504973	0.999867943
moaE	1.024375037	1.349689696	0.447870039	0.999867943
yiiF	1.022283714	1.514954803	0.499806107	0.999867943
rluA	1.01792388	0.91318104	0.264978543	0.999867943
nanE	1.017395113	1.545611946	0.510379143	0.999867943
yjfY	1.016890832	1.164413538	0.382495608	0.999867943
can	1.016445014	1.066599352	0.340601551	0.999867943
yrbG	1.015116321	0.879269333	0.248295296	0.999867943
malT	1.014169783	0.904781663	0.262330423	0.999867943
appC	1.006145996	0.941196471	0.285066333	0.999867943
ratB	0.998181526	1.375096725	0.467900606	0.999867943
allR	0.993846885	0.981084024	0.311055892	0.999867943
flgE	0.992998352	0.86029274	0.248395089	0.999867943
priC	0.990212969	1.479426333	0.503289934	0.999867943
gspM	0.988675586	0.759086585	0.192761131	0.999867943
ymdB	0.986149605	1.150387857	0.391316526	0.999867943
ibpB	0.985923843	0.828150829	0.233845054	0.999867943
rrlC	0.985347185	1.285041899	0.443211053	0.999867943
yibQ	0.984274388	0.847125742	0.245276573	0.999867943
poxB	0.983575269	0.943703703	0.297295745	0.999867943
yedD	0.983256586	1.33171207	0.46030773	0.999867943
degP	0.980575918	0.845398714	0.24609051	0.999867943
gcd	0.978459133	0.884609265	0.268686749	0.999867943
ygeX	0.976102623	1.122861805	0.38468362	0.999867943
malM	0.975875589	0.78142962	0.211725931	0.999867943
cobU	0.972520896	0.91927382	0.290090559	0.999867943
nuoF	0.971285813	1.162891031	0.403586122	0.999867943
cpdA	0.966693305	1.303468578	0.458310657	0.999867943
pmbA	0.965653999	0.906991021	0.287021363	0.999867943
ycaL	0.962704865	0.95443607	0.313136023	0.999867943
glgA	0.962195893	1.001628017	0.336737249	0.999867943
csiD	0.962161681	0.924979309	0.298247894	0.999867943
phr	0.95966861	0.961014823	0.3179889	0.999867943
alsK	0.9589217	0.875563249	0.27342661	0.999867943
frlR	0.955382426	0.772660801	0.21627885	0.999867943
yceG	0.954211272	0.895219895	0.286470674	0.999867943
oxyR	0.954017544	1.12790446	0.397646541	0.999867943
yifE	0.952105098	1.264910868	0.451627032	0.999867943

mutH	0.950023627	0.858967757	0.268723855	0.999867943
ffh	0.948571194	1.133539375	0.402692378	0.999867943
eutC	0.947364454	1.113632578	0.39493741	0.999867943
agaI	0.944712846	0.721463365	0.190385416	0.999867943
ycaC	0.938964047	0.854558143	0.27186779	0.999867943
galE	0.938933046	1.024616805	0.359470335	0.999867943
yheT	0.938914911	0.990559925	0.343199302	0.999867943
yhiD	0.932795798	0.792827555	0.239377916	0.999867943
ygiB	0.929661226	1.10936337	0.40202325	0.999867943
ubiA	0.923802889	0.835181662	0.268678859	0.999867943
cheR	0.922923974	1.097471758	0.400373343	0.999867943
cobT	0.922493832	0.828848167	0.265715776	0.999867943
yrhD	0.920329571	2.739794607	0.736937314	0.999867943
ppdD	0.9192604	1.015791352	0.365481437	0.999867943
elfA	0.918780605	0.835741806	0.271611335	0.999867943
apaH	0.9156346	1.023742257	0.37110816	0.999867943
yjfJ	0.912928163	0.815685617	0.263048122	0.999867943
yedF	0.912116374	1.236790295	0.460826438	0.999867943
fecR	0.910231207	1.42772529	0.523773517	0.999867943
ssrS	0.908992238	1.581941156	0.565558068	0.999867943
tatB	0.908316678	1.349777871	0.500986803	0.999867943
yjfI	0.907682302	0.96037419	0.344590468	0.999867943
alsE	0.907246594	0.745581116	0.22366833	0.999867943
ilvE	0.906657837	0.909083825	0.318603681	0.999867943
sdaA	0.906371726	1.037162181	0.382174975	0.999867943
nuoE	0.904876266	1.28540209	0.48145545	0.999867943
tar	0.904017541	1.017079523	0.374090884	0.999867943
ybiJ	0.903961303	1.172978935	0.440911845	0.999867943
yehY	0.902940444	1.293313195	0.485076702	0.999867943
kbl	0.90179604	0.72299942	0.212288094	0.999867943
sspB	0.901149242	1.43622827	0.530369861	0.999867943
yfbT	0.900911173	1.216940298	0.459112861	0.999867943
ydhW	0.899142289	1.084298075	0.406969161	0.999867943
cueR	0.895320459	1.399511028	0.522342912	0.999867943
ytfP	0.893022644	1.211900094	0.46119648	0.999867943
frlD	0.892124502	1.197844821	0.45640794	0.999867943
yhfS	0.88903625	1.094046697	0.416440142	0.999867943
nadK	0.887729525	0.91013499	0.329370679	0.999867943
cbeA	0.887448549	1.254854666	0.479434608	0.999867943
gntX	0.885937359	1.117454351	0.427884162	0.999867943
hemH	0.88559845	0.813030174	0.276040764	0.999867943
yehL	0.88528137	1.001739081	0.37683417	0.999867943
queF	0.885220425	0.766468856	0.248117731	0.999867943
ygbJ	0.884593596	0.819907151	0.280634609	0.999867943
prlC	0.884229688	0.874760132	0.312100044	0.999867943
argF	0.88413684	1.038798555	0.394705577	0.999867943
panD	0.883971871	0.998609362	0.376047226	0.999867943
frsA	0.883675202	0.921974038	0.337830871	0.999867943
ugpB	0.883261866	0.746108401	0.236482297	0.999867943

ybaQ	0.880765306	0.855171454	0.303043651	0.999867943
bglH	0.877211638	0.578834756	0.129651266	0.999867943
yjaB	0.877024849	0.881009399	0.319504184	0.999867943
fic	0.873555652	0.817427558	0.285220962	0.999867943
arsR	0.872201512	0.985544155	0.376159404	0.999867943
aldA	0.871235338	0.891600769	0.328490652	0.999867943
dam	0.871202668	1.467448608	0.552722616	0.999867943
clpB	0.870228619	0.781350913	0.26538698	0.999867943
ygeR	0.86942097	0.746614603	0.244227809	0.999867943
amiD	0.868924132	1.315125387	0.508794584	0.999867943
ybiI	0.868375573	1.164473304	0.455834219	0.999867943
ybgC	0.867918882	1.155438314	0.452556454	0.999867943
glcC	0.867535259	1.015416716	0.392903461	0.999867943
yidE	0.866845939	0.669091761	0.195128449	0.999867943
fldA	0.866198666	1.12204489	0.440124979	0.999867943
yccX	0.86512427	1.123367191	0.441230441	0.999867943
waaR	0.862759016	0.740959726	0.244269869	0.999867943
sdaC	0.862269773	1.06750189	0.419237145	0.999867943
agaB	0.853072264	0.933549314	0.360824817	0.999867943
fecC	0.853037982	1.491834504	0.567454292	0.999867943
hflX	0.852193308	1.001539317	0.394834055	0.999867943
treB	0.848332094	0.766953056	0.268680238	0.999867943
yjdK	0.847651325	1.270792768	0.504755773	0.999867943
gadY	0.847560708	3.14457991	0.787521349	0.999867943
pdxA	0.846961687	1.157452473	0.464323366	0.999867943
cstA	0.844357688	1.040301226	0.416994039	0.999867943
gspL	0.843682403	0.654561284	0.1974231	0.999867943
folB	0.840332163	1.063721508	0.429532066	0.999867943
glyS	0.838825907	0.945133392	0.374798367	0.999867943
spy	0.834307918	0.976172686	0.392732497	0.999867943
yaiV	0.83411659	0.766921517	0.276764389	0.999867943
gadE	0.833381309	0.886748369	0.34731137	0.999867943
ydcN	0.832591659	0.945670826	0.378629449	0.999867943
dtd	0.831484353	1.118445307	0.457222167	0.999867943
garL	0.830952645	0.955569628	0.384525161	0.999867943
entD	0.830943864	0.771688269	0.281575453	0.999867943
yqeH	0.830155784	0.998910456	0.405939022	0.999867943
ygjR	0.827880961	0.843937741	0.326605575	0.999867943
allB	0.826221526	0.745938881	0.268023238	0.999867943
copA	0.82529979	1.158356219	0.476170717	0.999867943
ybjP	0.821454831	0.971348104	0.397728316	0.999867943
yhaJ	0.820868189	0.967227877	0.396058668	0.999867943
fmt	0.817413792	0.981668726	0.405026447	0.999867943
ubiE	0.815188534	1.071557167	0.446805613	0.999867943
ydcT	0.815141318	1.020622647	0.424481429	0.999867943
ydcL	0.814984187	0.727100238	0.262343587	0.999867943
ravA	0.81474749	0.657188338	0.215068864	0.999867943
yjjZ	0.814287268	1.272676682	0.522287956	0.999867943
ythA	0.813155678	2.762211628	0.768463172	0.999867943

visC	0.811872469	0.896630531	0.365216197	0.999867943
psiF	0.80965045	0.976797236	0.407170717	0.999867943
yraQ	0.809454742	1.097795084	0.460912005	0.999867943
yagE	0.808376572	1.35697123	0.551361472	0.999867943
fadH	0.808067156	1.099522898	0.462385011	0.999867943
ratA	0.806911665	0.919041246	0.379947586	0.999867943
yjdC	0.805250208	0.940019547	0.391648657	0.999867943
envC	0.803348941	1.114198382	0.47090294	0.999867943
bglJ	0.802005831	0.724800804	0.268501963	0.999867943
dinF	0.799761587	0.98164262	0.415233937	0.999867943
ybaE	0.798490789	0.807565366	0.32277909	0.999867943
hyaC	0.798140109	0.924725718	0.388076934	0.999867943
ynjB	0.797762971	1.286109381	0.53506576	0.999867943
napH	0.797255046	1.25254068	0.524443869	0.999867943
yegP	0.796692036	0.945545043	0.39946652	0.999867943
rnhB	0.791284603	1.397700969	0.571303391	0.999867943
ynfG	0.789408907	1.058304733	0.455716747	0.999867943
mqsA	0.789230024	0.826461724	0.339602737	0.999867943
yraR	0.788830456	1.060131323	0.456823547	0.999867943
rfaH	0.784389238	0.886458073	0.376233393	0.999867943
yegI	0.784269225	0.804892214	0.329868914	0.999867943
uspB	0.782428712	1.172354048	0.504516873	0.999867943
yijF	0.780409603	0.73957766	0.291329378	0.999867943
ssuC	0.780278941	1.136195982	0.492242307	0.999867943
ispD	0.779299133	0.946096018	0.410110134	0.999867943
yafT	0.779135084	0.651749132	0.231910311	0.999867943
acpT	0.77622736	1.187316746	0.513262553	0.999867943
fliO	0.774649832	1.353468793	0.567088902	0.999867943
yhcG	0.773450838	0.715191238	0.279492469	0.999867943
aceF	0.773068299	0.937198884	0.40944476	0.999867943
ybdN	0.771065218	0.63494119	0.224599442	0.999867943
ugpC	0.770709377	1.247237819	0.53661952	0.999867943
hyaF	0.768822654	0.964775794	0.425513431	0.999867943
argP	0.768122409	0.896320787	0.391459994	0.999867943
yniC	0.767882787	0.890771179	0.388664024	0.999867943
yqjF	0.767603699	0.836517615	0.358818842	0.999867943
dsbC	0.765027348	0.981938046	0.43592116	0.999867943
glpE	0.764805703	1.091434745	0.48346888	0.999867943
folD	0.764213591	1.066368776	0.473589849	0.999867943
tyrA	0.763935298	0.843268874	0.364977469	0.999867943
nusA	0.759010964	0.923670825	0.411228771	0.999867943
pepQ	0.757737751	0.985140989	0.441794292	0.999867943
torD	0.755436794	1.200337174	0.529117439	0.999867943
ryfA	0.754236451	1.471783939	0.60832623	0.999867943
ygjH	0.751579497	1.020412677	0.461399306	0.999867943
yicJ	0.751259362	0.58873197	0.201933082	0.999867943
fecI	0.750707221	0.97183616	0.439840408	0.999867943
mlaB	0.750277385	1.238092074	0.544518192	0.999867943
gcvR	0.746680445	0.964215507	0.438699215	0.999867943

groL	0.745923003	0.811043148	0.357725286	0.999867943
nrfB	0.742663727	1.091866335	0.496391606	0.999867943
yegS	0.741695605	0.69118926	0.283238967	0.999867943
atoS	0.741255676	0.681424782	0.276682245	0.999867943
ydjG	0.740579321	0.745674298	0.320628436	0.999867943
kefG	0.740474202	0.985501554	0.452431294	0.999867943
yihS	0.739131853	0.770614879	0.337485412	0.999867943
fadB	0.739098857	0.907298356	0.415292718	0.999867943
cpxP	0.738921944	1.124885071	0.511253827	0.999867943
insA	0.738214917	1.568079602	0.637800398	0.999867943
cbrC	0.737993516	0.738319752	0.317524391	0.999867943
hemE	0.736940652	1.091849517	0.499709382	0.999867943
rne	0.735733024	0.983923538	0.454608392	0.999867943
gtrA	0.73439178	1.144806689	0.521198852	0.999867943
dacC	0.733998511	0.728515327	0.313681821	0.999867943
pabC	0.732944545	0.913070644	0.422133755	0.999867943
yecT	0.732905037	0.884751256	0.407458662	0.999867943
yhgA	0.730502094	0.672065955	0.277058904	0.999867943
ompL	0.728975596	0.771180932	0.344520126	0.999867943
pstA	0.72862094	0.920080022	0.428412921	0.999867943
yidX	0.728488766	0.748263667	0.330268954	0.999867943
ygdD	0.728003093	1.32011185	0.581311001	0.999867943
uspA	0.727585142	1.285622603	0.571434629	0.999867943
abgR	0.727497916	0.748621583	0.331158389	0.999867943
csdE	0.725476386	1.235841717	0.557183438	0.999867943
valZ	0.725415491	2.300148957	0.752474961	0.999867943
yobB	0.724222797	0.82026861	0.37728527	0.999867943
rnlB	0.718916253	1.066561805	0.500279366	0.999867943
holC	0.718273994	1.142329865	0.529493198	0.999867943
insG	0.717151594	0.678661534	0.290641831	0.999867943
rseX	0.715825164	2.890355414	0.804397552	0.999867943
ybbM	0.715133609	0.784875649	0.362220263	0.999867943
yidR	0.714193172	0.8920695	0.423361738	0.999867943
lsrD	0.712462742	0.970978696	0.463096646	0.999867943
hfq	0.712008572	1.278762924	0.577667623	0.999867943
tolC	0.71148679	1.062421069	0.503059014	0.999867943
allC	0.711440616	0.749780399	0.342689116	0.999867943
tabA	0.711157292	1.044388208	0.495914555	0.999867943
rtn	0.710986996	0.729619682	0.329826966	0.999867943
glmM	0.710905645	0.914526503	0.436953291	0.999867943
nagB	0.710824418	0.883066651	0.420848588	0.999867943
caiA	0.710213995	0.943759986	0.451728388	0.999867943
chrR	0.710213627	0.993109558	0.474521757	0.999867943
rrfC	0.709216447	1.561642023	0.649722378	0.999867943
aspV	0.708882744	1.835767799	0.699385175	0.999867943
dctR	0.708358893	0.94280799	0.452454739	0.999867943
btuE	0.707515691	0.917683252	0.44071862	0.999867943
yfcJ	0.706960014	1.109198083	0.523889482	0.999867943
wecE	0.705828194	0.968977072	0.466352788	0.999867943

hokA	0.705491366	2.75569027	0.797941126	0.999867943
ypfH	0.705164784	1.119778294	0.528867278	0.999867943
hflK	0.704116199	1.109541589	0.525688677	0.999867943
waaZ	0.702381544	1.088388697	0.518706411	0.999867943
yfbN	0.70208709	0.671211127	0.29556086	0.999867943
plaP	0.700551512	0.746951404	0.348305581	0.999867943
recD	0.699415222	1.034524858	0.498993747	0.999867943
tdcR	0.699164767	2.644586609	0.791490338	0.999867943
yhhL	0.698761852	1.183311789	0.554846287	0.999867943
yhaH	0.698734947	0.933597243	0.454199049	0.999867943
ftsA	0.695129651	0.99818253	0.486181288	0.999867943
ptsP	0.69318698	0.85547429	0.417770353	0.999867943
yihA	0.692815558	0.907634026	0.445272345	0.999867943
hldD	0.692204309	0.77766003	0.373405899	0.999867943
yciA	0.688541598	0.986502486	0.485200737	0.999867943
yjhQ	0.686953732	0.925566541	0.457967317	0.999867943
yoaG	0.686268059	2.916577977	0.813976624	0.999867943
mltA	0.686206079	0.832792133	0.409949545	0.999867943
zur	0.685767928	0.95858673	0.474365146	0.999867943
ygbA	0.685527885	0.959663148	0.475015603	0.999867943
ryjA	0.685111755	2.817220543	0.807860443	0.999867943
ygiW	0.685099378	0.974200364	0.481904459	0.999867943
xylB	0.684449282	0.793991521	0.388667702	0.999867943
ydcS	0.683304984	0.840164868	0.416046829	0.999867943
prfA	0.68306901	1.072330669	0.524128261	0.999867943
motA	0.682810335	0.990522297	0.490606985	0.999867943
yidB	0.682304811	0.880649753	0.438473028	0.999867943
ygdG	0.681699849	0.921474877	0.459426144	0.999867943
kdsC	0.680686981	0.915922785	0.457378338	0.999867943
gsiC	0.68021775	0.893442741	0.446451306	0.999867943
yfdR	0.680050498	0.973896792	0.485003487	0.999867943
ygcE	0.679602322	0.66404228	0.306103491	0.999867943
yieE	0.678760411	0.780516053	0.384502473	0.999867943
fdhF	0.678191462	0.776208976	0.382269244	0.999867943
dut	0.677604198	0.998026683	0.497173364	0.999867943
mrcB	0.675901883	0.858840561	0.431285709	0.999867943
yjgH	0.675004446	0.979032454	0.490533368	0.999867943
rob	0.673674301	1.011835887	0.505542757	0.999867943
cdh	0.672633563	0.741869822	0.364579584	0.999867943
queG	0.670381518	0.792520066	0.397616421	0.999867943
mrcA	0.669706408	0.824617033	0.416709959	0.999867943
malE	0.669332749	0.600349209	0.264890838	0.999867943
amiB	0.6686314	1.007958066	0.507104885	0.999867943
yggI	0.668487722	1.017138785	0.511037085	0.999867943
preA	0.667156126	0.776938153	0.390506523	0.999867943
rlmL	0.666536125	0.850091756	0.432995806	0.999867943
kup	0.666070993	0.667100062	0.318057613	0.999867943
yfhG	0.664953071	1.0148704	0.512332661	0.999867943
lysP	0.664857793	0.764295116	0.384356863	0.999867943

pyrH	0.664562933	1.025756251	0.517065106	0.999867943
nuoL	0.662653555	1.130109972	0.557632268	0.999867943
yncD	0.662252523	0.669069557	0.322266423	0.999867943
rimL	0.661290905	0.927527892	0.475870097	0.999867943
cusS	0.658369017	0.749442696	0.379684302	0.999867943
hspQ	0.6572335	1.432324922	0.646336266	0.999867943
emrA	0.656918632	0.837098453	0.432596223	0.999867943
ytjA	0.6569132	1.186880274	0.579935518	0.999867943
glnG	0.656703017	1.069007069	0.539009702	0.999867943
micC	0.656616222	3.319853121	0.843213501	0.999867943
uspE	0.656552899	1.197704715	0.583571106	0.999867943
chiA	0.656308467	0.609293941	0.281407761	0.999867943
aspU	0.656178654	1.834492122	0.720575658	0.999867943
yicN	0.655195067	0.963172222	0.496348043	0.999867943
apaG	0.65479313	1.202236048	0.585996968	0.999867943
cysB	0.653976531	0.765570717	0.392975352	0.999867943
exbB	0.652056106	1.076358063	0.544648562	0.999867943
gspJ	0.64982216	0.760916024	0.39310508	0.999867943
rbbA	0.649249068	0.940119405	0.489815204	0.999867943
speB	0.649064633	0.896591869	0.469112216	0.999867943
yjeO	0.648935703	0.924592229	0.482765531	0.999867943
waaL	0.648842189	0.62080355	0.295946682	0.999867943
yrfF	0.647641074	0.77248397	0.401812984	0.999867943
yhbO	0.645672298	0.840132327	0.442168557	0.999867943
bcsB	0.645362185	0.728860268	0.375919256	0.999867943
rlmJ	0.643806032	0.860822538	0.454522681	0.999867943
ompC	0.643136518	1.25492154	0.608307126	0.999867943
trmJ	0.642200578	1.169889016	0.583045628	0.999867943
secF	0.640168545	0.936757264	0.494361787	0.999867943
ymdF	0.639966989	1.124066254	0.569130794	0.999867943
mtlA	0.639471955	0.737769457	0.386071369	0.999867943
artQ	0.639277071	1.051865714	0.543349712	0.999867943
aaeB	0.63873313	0.818177361	0.434991857	0.999867943
napC	0.638733107	1.059116597	0.546454799	0.999867943
nepI	0.636952042	0.688343729	0.354789036	0.999867943
rbsC	0.635821989	1.258036192	0.61327206	0.999867943
gcvT	0.633650626	0.910474423	0.486456049	0.999867943
rnpA	0.633476851	1.300313195	0.62613608	0.999867943
yohF	0.633095152	0.990991394	0.522920321	0.999867943
caiT	0.632216236	0.698556356	0.365448146	0.999867943
yecD	0.632094109	0.96645321	0.513089334	0.999867943
sfsA	0.632020839	0.919999543	0.492095638	0.999867943
yagH	0.631030501	1.858977643	0.734270539	0.999867943
livH	0.630711847	1.017638963	0.535402915	0.999867943
mutL	0.629418159	1.11368093	0.571958685	0.999867943
ytfR	0.629116871	0.942667896	0.50453	0.999867943
pka	0.628921276	0.908636703	0.488837342	0.999867943
fliF	0.628358827	0.974306967	0.518973191	0.999867943
puuA	0.628316859	0.835861336	0.452231568	0.999867943

zupT	0.628029591	0.735715042	0.393309157	0.999867943
yaeF	0.627355603	0.70128953	0.371014685	0.999867943
ynjD	0.62712939	0.950917965	0.509575427	0.999867943
paaZ	0.625909048	0.793757213	0.430381106	0.999867943
tktB	0.623381822	0.846144041	0.461285034	0.999867943
yghS	0.621877018	0.705847569	0.378298412	0.999867943
alkA	0.620887625	1.182104735	0.599416988	0.999867943
appY	0.620528397	0.872327109	0.476868435	0.999867943
yjdO	0.619839988	2.82048293	0.82605513	0.999867943
ecpR	0.619519578	0.589647749	0.29341456	0.999867943
grpE	0.619256996	0.788335408	0.432145949	0.999867943
ybhD	0.619212547	0.683317952	0.364837887	0.999867943
yfcQ	0.618960437	1.138098006	0.586541169	0.999867943
dsbD	0.618305268	0.870313253	0.477431382	0.999867943
yhaK	0.618078226	0.844665525	0.464325344	0.999867943
sfmA	0.614684961	1.009711452	0.542674984	0.999867943
yadV	0.614546957	0.957932788	0.521175456	0.999867943
malY	0.611822797	0.782432595	0.434244237	0.999867943
yihX	0.61103657	0.82482939	0.45881254	0.999867943
rfbC	0.610484125	0.891638743	0.493548059	0.999867943
leuD	0.609739061	0.936384352	0.514941122	0.999867943
fdoH	0.607964611	1.172826042	0.604196112	0.999867943
yiaV	0.606230684	0.67490194	0.36905227	0.999867943
yeiP	0.605965373	0.928974666	0.514210993	0.999867943
gor	0.605671271	0.827834048	0.464392185	0.999867943
livF	0.605604254	1.159502283	0.601463744	0.999867943
insH1	0.604307452	1.447238769	0.67626964	0.999867943
ycaN	0.603046568	0.747563686	0.419849409	0.999867943
insH1	0.602494608	1.579866365	0.702937654	0.999867943
pgm	0.600905276	0.958203734	0.530582938	0.999867943
sgcX	0.600509272	0.733045512	0.412673569	0.999867943
yfjX	0.599525288	0.989935192	0.544766624	0.999867943
prkB	0.599445264	0.769314096	0.435865187	0.999867943
yihY	0.598011987	0.726460441	0.41040229	0.999867943
gcvP	0.59710141	1.010705136	0.554669807	0.999867943
phoH	0.596940101	0.8023689	0.456893335	0.999867943
ydfU	0.595480755	0.86854972	0.492963155	0.999867943
ykgI	0.594431768	0.909277917	0.513279029	0.999867943
ygfB	0.594213259	1.062198654	0.575876343	0.999867943
hda	0.594137623	0.913874102	0.51560779	0.999867943
phnL	0.593303896	1.573273075	0.706088607	0.999867943
zitB	0.590998057	0.899540843	0.511181114	0.999867943
chpS	0.589852479	1.332538849	0.658016656	0.999867943
ypdB	0.588622684	0.832557211	0.479562943	0.999867943
tldD	0.588614492	0.800713493	0.462270986	0.999867943
insH1	0.588424994	1.513767523	0.697486569	0.999867943
cmtB	0.588130531	0.966326595	0.542773025	0.999867943
mscS	0.586921762	0.769651662	0.445713297	0.999867943
paaI	0.585310432	1.292090508	0.650552402	0.999867943

yhhX	0.584160696	0.67022677	0.383433924	0.999867943
insH1	0.583897561	2.587398693	0.821458304	0.999867943
ymgE	0.583129887	0.97903686	0.551431875	0.999867943
gstA	0.582723341	0.908075544	0.521059933	0.999867943
wcaK	0.581902122	0.856211221	0.496742253	0.999867943
rybB	0.58169056	3.422590171	0.86504455	0.999867943
ydeA	0.578595217	0.72654177	0.425817691	0.999867943
pgi	0.578004121	0.688109636	0.400914883	0.999867943
yoaK	0.576813891	3.524613894	0.870004262	0.999867943
yggP	0.575406325	0.787905461	0.465207629	0.999867943
ynfN	0.574153883	2.913879355	0.843795405	0.999867943
yjtD	0.573353305	0.778557435	0.461468856	0.999867943
yciN	0.57290347	1.336102863	0.668078277	0.999867943
dsdC	0.572610685	0.661718439	0.386852823	0.999867943
yiiM	0.571452837	0.743873193	0.442360827	0.999867943
wecA	0.570873706	0.657993139	0.385614296	0.999867943
yqcG	0.570372591	2.850648555	0.841413947	0.999867943
yibD	0.570128457	0.668353433	0.393640239	0.999867943
ilvA	0.569548338	1.216827689	0.6397417	0.999867943
dppD	0.568900418	1.02697832	0.579609215	0.999867943
menA	0.568659163	0.749768265	0.448183693	0.999867943
yafO	0.568056638	0.830999658	0.49423898	0.999867943
rnpB	0.567153745	1.312244605	0.665595158	0.999867943
racR	0.56563214	0.942337583	0.548343874	0.999867943
acrE	0.56496278	0.651028224	0.38550342	0.999867943
yheS	0.562469375	0.916832611	0.539551141	0.999867943
ybiY	0.559438073	1.001455553	0.576417698	0.999867943
nuoG	0.558565639	1.150769751	0.627403725	0.999867943
insH1	0.558316491	1.156854635	0.629368456	0.999867943
insH1	0.557717652	1.216060882	0.646502459	0.999867943
cadC	0.557416564	0.684618977	0.415530558	0.999867943
tsgA	0.557123433	0.66354063	0.401120256	0.999867943
ulaD	0.556786744	1.157367237	0.630459327	0.999867943
recQ	0.555183769	0.853924451	0.515591713	0.999867943
zinT	0.554685052	0.912724097	0.543370024	0.999867943
rsmB	0.553393593	0.703406073	0.431437497	0.999867943
purL	0.552824175	0.960349238	0.564852386	0.999867943
idnK	0.552411131	0.882079009	0.531144173	0.999867943
yjbS	0.55222033	2.608602474	0.832347313	0.999867943
yibT	0.551914761	1.324423469	0.676882664	0.999867943
yadM	0.551595168	0.875864898	0.528843783	0.999867943
mobA	0.551159603	0.87532098	0.528913324	0.999867943
yfjP	0.550060439	0.783697382	0.482754862	0.999867943
msyB	0.549950053	0.932226584	0.555236413	0.999867943
rrfH	0.548128587	1.600036792	0.731920101	0.999867943
ftnB	0.547734952	0.760576081	0.471427718	0.999867943
hslJ	0.546782438	1.006358254	0.586904111	0.999867943
xylF	0.546728804	0.626048322	0.38249888	0.999867943
yfcO	0.546669444	0.722471551	0.449250167	0.999867943

ybgK	0.544388397	1.156878499	0.637950254	0.999867943
paoD	0.544317054	1.240154654	0.660726298	0.999867943
ytfL	0.54341229	0.641927669	0.39725605	0.999867943
acrB	0.541301168	0.830158901	0.514371982	0.999867943
nrdA	0.540555997	0.881770507	0.539853337	0.999867943
pliG	0.539864806	0.970117358	0.577872972	0.999867943
gadX	0.539769752	0.928172132	0.560876183	0.999867943
fusA	0.539707986	1.300702156	0.678188847	0.999867943
hns	0.538707544	1.101077763	0.624661341	0.999867943
nirB	0.538260097	0.702709147	0.443689096	0.999867943
galS	0.537914978	0.981360039	0.583601174	0.999867943
bolA	0.537322375	1.063289893	0.613320409	0.999867943
glgS	0.537006396	2.626509207	0.837996926	0.999867943
ygeI	0.536974485	2.196412996	0.806860699	0.999867943
agp	0.536779883	0.96483938	0.577977638	0.999867943
smf	0.536659307	0.727367151	0.460629456	0.999867943
gss	0.535320623	0.699132013	0.443858879	0.999867943
ttcA	0.535165191	0.710930862	0.451590025	0.999867943
yjbM	0.533857966	1.027974908	0.603530801	0.999867943
map	0.533728361	0.88877312	0.548157991	0.999867943
atpG	0.533351022	1.209580995	0.659257405	0.999867943
nhaB	0.531456511	0.806120669	0.509718152	0.999867943
glmS	0.530170277	0.685405733	0.43921881	0.999867943
paaK	0.530127817	0.887387659	0.550238526	0.999867943
hyi	0.528329719	0.706544087	0.45460125	0.999867943
rsfS	0.52828645	1.326885121	0.690526764	0.999867943
rsxD	0.527911787	0.858284959	0.538503525	0.999867943
yhjC	0.527827839	0.699001544	0.450178878	0.999867943
ydgJ	0.527338239	0.807654302	0.513804189	0.999867943
rpoN	0.526698707	0.90776746	0.56177082	0.999867943
ynbE	0.52614731	3.073526515	0.86407685	0.999867943
yjbL	0.525611352	2.496435188	0.83324252	0.999867943
ftsP	0.524872057	0.864009651	0.543529744	0.999867943
soxR	0.524061326	0.967297049	0.58797069	0.999867943
ydiU	0.522891023	0.931828111	0.574698446	0.999867943
rsmG	0.522876761	0.998001752	0.600331617	0.999867943
rlmB	0.521797414	0.906405679	0.564833142	0.999867943
gadB	0.520744801	1.082984152	0.630628446	0.999867943
fbp	0.520360828	0.852664409	0.541678834	0.999867943
ymgD	0.518508562	0.916724599	0.571658957	0.999867943
yiaU	0.517861477	0.707684876	0.464309899	0.999867943
sstT	0.517077049	0.832966837	0.534753947	0.999867943
zapC	0.516570001	0.864905836	0.55033663	0.999867943
secA	0.516371243	0.767013962	0.500805594	0.999867943
yhfW	0.515819469	0.82625053	0.532437527	0.999867943
nanR	0.515499195	0.807727882	0.523337646	0.999867943
torY	0.515213322	0.716613116	0.472168011	0.999867943
kdpD	0.514947386	1.018031648	0.612978453	0.999867943
htrE	0.514814938	0.603399436	0.393553421	0.999867943

wcaF	0.513812536	0.971158669	0.596755735	0.999867943
lsrF	0.513581053	0.920107916	0.576725026	0.999867943
eutH	0.513192032	0.978391438	0.599912539	0.999867943
yigZ	0.511716618	0.9876782	0.604388117	0.999867943
kdgK	0.511530166	0.922666862	0.579302414	0.999867943
gspG	0.511281802	0.912897896	0.57543537	0.999867943
csgC	0.511270533	1.025873932	0.618219334	0.999867943
tyrV	0.511206539	1.910532194	0.789028134	0.999867943
higA	0.510815678	0.878301331	0.5608395	0.999867943
ybbO	0.510558666	0.991911683	0.606747368	0.999867943
tauB	0.50881911	1.164276253	0.66209242	0.999867943
crl	0.508537035	0.837486705	0.543706222	0.999867943
yicH	0.508421432	0.74992618	0.497795805	0.999867943
yjeH	0.507371201	0.715720059	0.478388919	0.999867943
yqeK	0.507262967	1.212280962	0.675627785	0.999867943
yhjB	0.505460244	0.926109178	0.585210954	0.999867943
yihL	0.505384774	0.687347748	0.462176319	0.999867943
yhiM	0.504830264	0.760860974	0.507011214	0.999867943
rfbB	0.504361621	0.711319699	0.478293111	0.999867943
yjhH	0.50375318	0.809795548	0.533892866	0.999867943
gnsB	0.503531211	2.495907943	0.840117878	0.999867943
gnd	0.502891811	0.7128661	0.48052987	0.999867943
rsxC	0.502038078	1.139406548	0.659492719	0.999867943
metE	0.50183507	0.81590433	0.538511088	0.999867943
nfsB	0.501688047	0.828689783	0.54491385	0.999867943
fixA	0.501183211	0.828099067	0.545031952	0.999867943
htpG	0.500516311	0.649748764	0.441108413	0.999867943
quuQ	0.500502782	0.724942276	0.489940392	0.999867943
ymiB	0.498882441	3.512158395	0.887044933	0.999867943
fldB	0.49883076	0.969106375	0.606739806	0.999867943
yqeI	0.497520629	0.81135445	0.539745669	0.999867943
ykfF	0.497350992	1.182327016	0.674007507	0.999867943
yaeQ	0.497021726	0.922037648	0.589854351	0.999867943
yhfA	0.495809156	1.065175625	0.641593667	0.999867943
ybbP	0.495778328	0.969203391	0.608978746	0.999867943
bdcR	0.495189128	0.855037993	0.562492799	0.999867943
aroM	0.494168659	0.699326458	0.479793238	0.999867943
glgB	0.493751412	0.59253347	0.404681959	0.999867943
narL	0.493716106	0.889648953	0.578924694	0.999867943
ybaZ	0.49343739	1.228841314	0.688017534	0.999867943
rlmN	0.492804911	0.925610352	0.594441535	0.999867943
hsrA	0.492748903	0.631793535	0.43543751	0.999867943
yfjR	0.491934225	0.954298644	0.606208593	0.999867943
insC1	0.489500284	1.256223593	0.696787861	0.999867943
yfcG	0.488773814	0.800520334	0.541484195	0.999867943
yohC	0.488677857	0.849514437	0.565126469	0.999867943
rhaA	0.488554982	0.984657111	0.619776149	0.999867943
yhhT	0.488493432	0.633792276	0.440856967	0.999867943
flhE	0.488331529	1.124287269	0.664036009	0.999867943

galM	0.486576914	1.005216633	0.628349112	0.999867943
gldA	0.486400659	0.7780742	0.531883096	0.999867943
yfjK	0.486207682	0.649874315	0.454365776	0.999867943
ydbK	0.486038826	0.893192755	0.586332102	0.999867943
ydeH	0.485753073	0.657055098	0.45973202	0.999867943
miaB	0.485605949	0.864170392	0.574161599	0.999867943
pncB	0.483932936	0.780924724	0.535460472	0.999867943
glgP	0.483849478	0.669475824	0.469846501	0.999867943
trpR	0.483642225	1.26383673	0.701958697	0.999867943
yjcD	0.483600444	0.679902962	0.476911595	0.999867943
yfcA	0.482705242	0.993801813	0.62716868	0.999867943
alaU	0.482400224	2.604504577	0.853058324	0.999867943
ybjC	0.481712236	0.942473592	0.609270648	0.999867943
ybfE	0.480190461	1.364892832	0.72497663	0.999867943
nlpD	0.479357099	1.075178395	0.655713119	0.999867943
upp	0.478429761	0.986491991	0.627689904	0.999867943
yfeR	0.478207649	0.919114731	0.602860369	0.999867943
yqeG	0.477789615	0.885611428	0.589540144	0.999867943
bglG	0.477729522	0.915137242	0.601649118	0.999867943
kduI	0.477352182	0.785006776	0.543129971	0.999867943
ykfG	0.477270429	1.372874188	0.72810813	0.999867943
tdcB	0.476626758	0.627489914	0.447507613	0.999867943
mtlR	0.476326693	0.803758519	0.553432727	0.999867943
aslB	0.476124573	0.71685488	0.506571632	0.999867943
eutE	0.475579962	1.442651728	0.741659294	0.999867943
glnH	0.475400816	0.827708363	0.565725653	0.999867943
cyoD	0.47490081	1.306933797	0.716328351	0.999867943
motB	0.474680643	0.761439555	0.533022367	0.999867943
cheZ	0.474493005	0.979825807	0.628199506	0.999867943
ribD	0.474429462	1.013370346	0.639663118	0.999867943
ycjZ	0.474114109	0.7881254	0.547459126	0.999867943
ycgB	0.473953959	0.699728207	0.498190207	0.999867943
thrU	0.473273472	2.79618955	0.86559483	0.999867943
yjjA	0.473013345	0.776534222	0.542435667	0.999867943
ygcW	0.471953889	0.767807694	0.538767915	0.999867943
fabI	0.471134786	0.954333745	0.621532708	0.999867943
mglB	0.471110081	0.91542217	0.606806734	0.999867943
trkA	0.470333198	0.689792696	0.495335594	0.999867943
fucP	0.470002554	0.596519554	0.430750497	0.999867943
yrfG	0.469003686	0.789071552	0.552261934	0.999867943
hupA	0.468383234	1.296968171	0.717997012	0.999867943
viaA	0.468185562	0.706912896	0.507781559	0.999867943
aphA	0.467603192	0.692414708	0.499470928	0.999867943
yeaM	0.466245088	1.01626467	0.646389909	0.999867943
iap	0.465646478	0.66623822	0.484602721	0.999867943
yhjV	0.465591899	0.59006043	0.430078112	0.999867943
yiaA	0.465588282	0.952010637	0.624800736	0.999867943
sgcC	0.464218959	0.670233228	0.488546163	0.999867943
yafX	0.464014829	0.872400569	0.594807193	0.999867943

ydbC	0.462883026	0.9170829	0.613745597	0.999867943
yfgC	0.461683985	0.856050041	0.589666815	0.999867943
yphF	0.46056991	0.833468556	0.580541298	0.999867943
glpQ	0.459642534	0.739218611	0.534076587	0.999867943
fliL	0.459635795	0.895878209	0.607912067	0.999867943
wecH	0.459600067	0.714505818	0.520067161	0.999867943
nrdF	0.459365091	0.666829324	0.490899063	0.999867943
valX	0.458586921	2.197533135	0.834696027	0.999867943
tpx	0.4577487	1.235931024	0.711108676	0.999867943
blr	0.455343564	2.820956497	0.871766868	0.999867943
sdhA	0.455316783	1.085188479	0.674796745	0.999867943
yidL	0.454272812	0.961452598	0.636579992	0.999867943
blc	0.45340712	0.893121301	0.611687752	0.999867943
yiaJ	0.453175659	0.827376161	0.583879871	0.999867943
chaA	0.45197164	0.731614877	0.536725233	0.999867943
uhpT	0.450484958	0.617301892	0.465534196	0.999867943
yifK	0.45046601	0.644551005	0.48462489	0.999867943
sufE	0.450130302	0.919369071	0.62441143	0.999867943
glgC	0.449773943	0.709477703	0.526112944	0.999867943
waaJ	0.449471683	0.843685668	0.594208173	0.999867943
safA	0.448931314	2.787209884	0.872039563	0.999867943
frvA	0.447548882	0.940535204	0.634184878	0.999867943
yjfM	0.447544113	0.765208429	0.558638067	0.999867943
qseC	0.446985119	0.723848089	0.536896852	0.999867943
yfiE	0.446968509	0.727660279	0.539047247	0.999867943
xapR	0.446926907	0.755426718	0.55410388	0.999867943
yhjX	0.446606365	0.622030669	0.47276884	0.999867943
nrdE	0.44495343	0.771188349	0.563958944	0.999867943
ycgJ	0.444406027	0.930036856	0.63276622	0.999867943
frvR	0.442674513	0.62212388	0.476741221	0.999867943
ftsW	0.441488718	0.770722239	0.566763409	0.999867943
yifB	0.440142181	0.586489639	0.452972402	0.999867943
plsY	0.440016974	0.776145367	0.570764471	0.999867943
pdhR	0.439609393	0.897820355	0.624388166	0.999867943
aceK	0.4389666	0.755220398	0.561076663	0.999867943
hokE	0.438700198	2.922885143	0.880692431	0.999867943
ylaB	0.438699272	0.609050408	0.471340038	0.999867943
yhfZ	0.43834341	0.813126334	0.589828884	0.999867943
dnaG	0.437550505	0.679138378	0.519398435	0.999867943
coaA	0.437422734	0.770494321	0.570227138	0.999867943
hycE	0.437184442	0.941827312	0.642513434	0.999867943
glpF	0.437164978	0.893127681	0.624504415	0.999867943
yciK	0.436960464	0.875745861	0.617808924	0.999867943
moaB	0.436500488	0.993620293	0.660441918	0.999867943
ybiC	0.434747663	1.055976826	0.680557934	0.999867943
dcrB	0.434638918	1.018779445	0.669650958	0.999867943
lsrA	0.434616596	0.860159336	0.613366036	0.999867943
pepD	0.434521654	0.822789028	0.597424221	0.999867943
glyQ	0.434376125	0.88603647	0.623959602	0.999867943

yiaG	0.434374671	2.39938567	0.856339487	0.999867943
rraB	0.434125279	1.060040585	0.682146009	0.999867943
yafZ	0.433846113	0.939691609	0.644303724	0.999867943
basS	0.433267965	0.741299092	0.558903341	0.999867943
treF	0.432177287	0.612100216	0.480153052	0.999867943
yibB	0.432123342	0.814787701	0.595868703	0.999867943
yjjV	0.431716791	1.113937979	0.698342347	0.999867943
yihO	0.431478099	0.659811967	0.513149817	0.999867943
waaC	0.431081569	0.674795615	0.52293171	0.999867943
rtcB	0.43090827	0.627073118	0.491973159	0.999867943
fepA	0.429284025	0.656048046	0.512887335	0.999867943
hslU	0.429190117	0.751895491	0.568127856	0.999867943
ogrK	0.428538869	2.880301382	0.881725142	0.999867943
asnA	0.427149345	0.841844307	0.611876233	0.999867943
dhaL	0.425774158	1.095593427	0.697554136	0.999867943
yfjT	0.425515607	0.917267968	0.642723301	0.999867943
waaP	0.425487301	0.778117534	0.584504988	0.999867943
proV	0.425292381	0.6485937	0.512007751	0.999867943
scpC	0.42501447	0.792442065	0.591726997	0.999867943
yfdF	0.424518901	0.832838536	0.610243902	0.999867943
dauA	0.42280698	0.887987638	0.633974204	0.999867943
betB	0.421510826	1.342203407	0.753487678	0.999867943
tsaC	0.42098639	0.914090013	0.645119737	0.999867943
melR	0.420486342	0.79154965	0.595266916	0.999867943
cspI	0.42005496	2.434194366	0.862993943	0.999867943
yedQ	0.419056602	0.674838485	0.534617268	0.999867943
yicO	0.418775952	0.587031561	0.475611302	0.999867943
djlA	0.41715377	0.704820769	0.553945719	0.999867943
hcaC	0.416905235	1.34887072	0.757262775	0.999867943
nrfA	0.416098734	0.604620223	0.491327784	0.999867943
ilvC	0.415878383	0.688053478	0.545559515	0.999867943
atpA	0.415670958	1.221690538	0.733673967	0.999867943
yceO	0.415630227	2.639710913	0.874887899	0.999867943
fhuC	0.41555874	1.082774155	0.701133521	0.999867943
ydaG	0.415283204	3.130652166	0.894469633	0.999867943
cysG	0.412308181	0.783943191	0.598928922	0.999867943
rep	0.41227611	0.647303295	0.524181287	0.999867943
tyrS	0.411149384	0.853206375	0.629885952	0.999867943
cho	0.409534973	0.886709847	0.644182397	0.999867943
groS	0.408699108	1.066514102	0.701563898	0.999867943
pyrF	0.40833405	0.934387841	0.662106254	0.999867943
fhuE	0.408063463	0.598981159	0.495705343	0.999867943
yidI	0.407828632	0.751645045	0.587418037	0.999867943
mhpA	0.407430343	1.23042632	0.740546737	0.999867943
folP	0.407194713	0.821750413	0.620232293	0.999867943
yhfX	0.406989089	0.783887277	0.603625892	0.999867943
yiaO	0.406173481	0.72329495	0.57441587	0.999867943
fliD	0.405699511	0.727451772	0.577049579	0.999867943
yjfZ	0.405178132	0.982168872	0.679948007	0.999867943

emrB	0.404895641	0.991845249	0.683108781	0.999867943
yihR	0.404828202	0.766143285	0.597223468	0.999867943
uspG	0.404067542	1.111430285	0.716189324	0.999867943
nfuA	0.40396087	0.971455995	0.677534161	0.999867943
tmk	0.403874626	0.996951338	0.6853969	0.999867943
envR	0.403114953	0.84127795	0.631817866	0.999867943
yfjL	0.403050046	0.668591615	0.546618839	0.999867943
yjcC	0.402686245	0.670806684	0.548305333	0.999867943
aceE	0.40222635	0.841961952	0.632845884	0.999867943
yjjI	0.401486543	0.723964173	0.579190957	0.999867943
alsC	0.401116928	0.761522184	0.598380782	0.999867943
gmhA	0.40056626	0.894400092	0.654254048	0.999867943
metH	0.400266609	0.730264166	0.583614949	0.999867943
ryhB	0.399951511	3.055522683	0.895858663	0.999867943
raiA	0.399858644	1.597038254	0.802297269	0.999867943
secB	0.399402851	1.02630877	0.697154526	0.999867943
arsC	0.398245495	1.026079223	0.697924617	0.999867943
ompR	0.39822208	0.935398589	0.670308621	0.999867943
php	0.398135381	0.830698648	0.631741047	0.999867943
recF	0.398116765	0.906416723	0.660501805	0.999867943
fes	0.397868225	0.905873601	0.660509795	0.999867943
grxD	0.397048285	1.321546995	0.763839995	0.999867943
agaR	0.396657945	0.893286731	0.657011313	0.999867943
tag	0.396252089	0.947335304	0.675741888	0.999867943
xanP	0.396115536	0.711480814	0.577699602	0.999867943
yfdY	0.394928956	2.594794442	0.879028816	0.999867943
ydcJ	0.39458233	0.725327733	0.586437637	0.999867943
glpX	0.394067522	1.088854213	0.717419085	0.999867943
damX	0.393787288	0.774904981	0.611330667	0.999867943
flgJ	0.393564768	1.107226112	0.722251653	0.999867943
yjgL	0.392435542	0.820077995	0.632269887	0.999867943
sfmF	0.392147322	0.903552634	0.664284038	0.999867943
ygeG	0.391316783	2.025523406	0.846807883	0.999867943
yccU	0.391160863	0.834037504	0.639071973	0.999867943
yjcB	0.390468299	1.276355646	0.759662229	0.999867943
yfjH	0.390194595	0.763012233	0.609080043	0.999867943
ycfT	0.389989519	0.701690251	0.578357253	0.999867943
mtlD	0.389660529	0.740620482	0.598799879	0.999867943
tyrB	0.389556317	0.638572226	0.54183353	0.999867943
fnr	0.388480765	0.90926786	0.669200393	0.999867943
yhhZ	0.387401645	0.91488231	0.671971173	0.999867943
yfdQ	0.385165298	0.801035375	0.630634258	0.999867943
phoR	0.384974169	0.708941161	0.587111235	0.999867943
sppA	0.384505048	0.661321057	0.560957666	0.999867943
yadD	0.384008387	0.732489536	0.600103959	0.999867943
yigA	0.383857125	1.16644907	0.742093883	0.999867943
insH1	0.38326018	0.977259967	0.694926456	0.999867943
sgcQ	0.383120907	0.756792162	0.612685912	0.999867943
emrK	0.383007288	0.883307025	0.664574526	0.999867943

nfrA	0.382813312	0.93510692	0.68226141	0.999867943
hemC	0.382572442	0.944170756	0.685334824	0.999867943
rpoH	0.382545482	1.102652766	0.728642438	0.999867943
ydfB	0.382406548	3.078934478	0.90115618	0.999867943
nikD	0.381959201	1.077120217	0.72288067	0.999867943
trpB	0.381756526	0.98761829	0.699094871	0.999867943
paoB	0.381559663	1.366401189	0.780057394	0.999867943
puuR	0.381539577	0.843473321	0.651021626	0.999867943
msrA	0.381294754	0.89063099	0.668565543	0.999867943
fadJ	0.38087091	0.7767404	0.623889663	0.999867943
suhB	0.380621961	1.050656118	0.717149728	0.999867943
rseP	0.379018754	0.705739907	0.591231964	0.999867943
ydaV	0.377829344	0.77790277	0.62717809	0.999867943
yciU	0.376717501	0.936334722	0.687439645	0.999867943
opgD	0.37543624	0.750936234	0.617104968	0.999867943
purA	0.374936725	1.145478732	0.743426774	0.999867943
insC1	0.373959444	1.411973552	0.791125973	0.999867943
yihM	0.373610783	1.052691324	0.722656942	0.999867943
dicB	0.373403762	2.423111327	0.877530138	0.999867943
insC1	0.372585619	1.337650231	0.780599859	0.999867943
pqiA	0.372212888	0.782440501	0.63428271	0.999867943
tas	0.371179388	1.020508232	0.716067009	0.999867943
yigM	0.371079757	0.848997185	0.662053274	0.999867943
ychJ	0.370453166	0.956773655	0.698615572	0.999867943
hcaE	0.369669139	0.742262219	0.618462626	0.999867943
yddH	0.369557762	0.832374289	0.657056848	0.999867943
ygfI	0.369431242	0.770878681	0.631772198	0.999867943
ygeH	0.368797176	0.962881085	0.701709199	0.999867943
hisD	0.368743767	1.246708403	0.767402781	0.999867943
sbcD	0.368423213	0.839327895	0.660697565	0.999867943
glmU	0.368233151	0.677102245	0.58655375	0.999867943
ygeA	0.367435562	0.803147313	0.64731558	0.999867943
dnaQ	0.367243279	0.918309806	0.689221199	0.999867943
prfC	0.367017475	0.719968445	0.610213422	0.999867943
mcbR	0.366537079	0.827729457	0.657894257	0.999867943
yghJ	0.36552841	0.526636859	0.487631685	0.999867943
degQ	0.365222645	0.708722447	0.606325686	0.999867943
bcsZ	0.364385064	0.963352075	0.705247089	0.999867943
ygbT	0.364343894	0.753484838	0.628708752	0.999867943
trmH	0.363887153	0.962649577	0.705426263	0.999867943
ccmA	0.363787161	1.226170923	0.766706611	0.999867943
valY	0.362903378	2.220305841	0.870166128	0.999867943
yeaG	0.361882907	0.614842776	0.556144422	0.999867943
gloB	0.361308824	0.773991842	0.640634256	0.999867943
rpiR	0.360420612	0.669729598	0.590467693	0.999867943
dnaT	0.36017283	1.095289986	0.742278235	0.999867943
alaT	0.35964927	2.624678882	0.891010237	0.999867943
slp	0.35935504	1.229004428	0.76998445	0.999867943
sgcR	0.358995823	0.724197614	0.620095305	0.999867943

yqjH	0.358716367	1.064627714	0.736161632	0.999867943
yafE	0.35796402	1.017238502	0.724914888	0.999867943
lptB	0.357677938	1.036729896	0.73008993	0.999867943
yehT	0.357409671	0.795231177	0.653113401	0.999867943
cheA	0.357380632	0.896907823	0.690292172	0.999867943
ubiD	0.3573504	0.704074364	0.611771496	0.999867943
ycgX	0.356541708	0.888257599	0.688129704	0.999867943
ygcB	0.356460867	0.607849214	0.557586752	0.999867943
lptD	0.356181988	0.717082949	0.619393848	0.999867943
yhaC	0.355537172	0.884771073	0.687801146	0.999867943
ftnA	0.354997674	0.784846683	0.651042304	0.999867943
yfeN	0.354677612	0.735458985	0.629625931	0.999867943
yjgA	0.35438168	0.81203939	0.662539669	0.999867943
lolB	0.353851534	0.912903898	0.698303975	0.999867943
hdfR	0.353791579	0.742957039	0.633936274	0.999867943
ydiY	0.353543229	0.74550467	0.635333464	0.999867943
mpl	0.353344346	0.849368745	0.677404017	0.999867943
gltJ	0.353118232	0.791143928	0.655352532	0.999867943
yhcM	0.351834703	0.61062934	0.564490987	0.999867943
phoB	0.351625457	0.820755592	0.66834772	0.999867943
gmd	0.351204491	0.999206022	0.725225603	0.999867943
gpr	0.350946939	0.874873531	0.688316909	0.999867943
talA	0.350725994	0.707751574	0.620212235	0.999867943
ydjX	0.350634066	0.75302358	0.641476851	0.999867943
ybgE	0.350587991	1.105775515	0.751204384	0.999867943
hrpA	0.35047585	0.835612753	0.674906456	0.999867943
waaB	0.350382143	1.020849023	0.731428287	0.999867943
yciV	0.350273	1.080837763	0.745880902	0.999867943
ylcI	0.350002589	2.004767061	0.861405606	0.999867943
ruvA	0.348425466	1.094907492	0.750315289	0.999867943
uhpC	0.348115613	0.764187813	0.648723154	0.999867943
yqaB	0.347190316	0.911038979	0.703134455	0.999867943
nanA	0.346713129	0.751353393	0.64447473	0.999867943
ddlA	0.346609575	0.77660156	0.655369118	0.999867943
dcuD	0.345879807	0.642886073	0.590569424	0.999867943
mukB	0.345648952	0.941912996	0.713645245	0.999867943
higB	0.345371934	0.937237297	0.712500453	0.999867943
exoX	0.344512003	0.970328254	0.722554483	0.999867943
uacT	0.34447059	0.702558199	0.623915278	0.999867943
yccS	0.344425425	0.821142575	0.674889805	0.999867943
gspK	0.344064895	0.733065836	0.638819048	0.999867943
lexA	0.343968139	0.979399926	0.7254361	0.999867943
fumB	0.342798691	0.73707532	0.641874458	0.999867943
mioC	0.342662862	0.961898651	0.721663885	0.999867943
yaiS	0.342639639	1.119926469	0.759643983	0.999867943
yqcA	0.342232828	1.078237815	0.750940005	0.999867943
hpf	0.342067189	1.20579923	0.776651769	0.999867943
hcaR	0.341244858	0.685664312	0.618706066	0.999867943
cpxR	0.341020504	0.897302952	0.703907872	0.999867943

fumC	0.339545061	0.909059791	0.708767153	0.999867943
yieL	0.338618329	0.821898412	0.680342827	0.999867943
gpp	0.336925148	0.764020221	0.659220359	0.999867943
yiaY	0.336897088	0.810729432	0.677740699	0.999867943
yfbR	0.335947752	0.88765041	0.705082563	0.999867943
clpX	0.335793217	0.969915328	0.729185805	0.999867943
yfcS	0.335537266	0.743480682	0.651769047	0.999867943
ydeM	0.335316197	0.740212741	0.650548912	0.999867943
hdhA	0.334983197	0.769234444	0.663216901	0.999867943
rluF	0.334528083	0.705705993	0.635476219	0.999867943
ansA	0.33441032	0.795908046	0.674367109	0.999867943
rpe	0.334279323	0.887120143	0.706311808	0.999867943
yafC	0.333085602	0.677799404	0.623127941	0.999867943
alaS	0.332128918	0.781228104	0.670736858	0.999867943
yfaQ	0.330786928	0.803522886	0.680580714	0.999867943
xylR	0.330471975	0.704064012	0.638799657	0.999867943
yciF	0.33018093	0.895127893	0.712228703	0.999867943
yibH	0.330178767	0.676956475	0.625733998	0.999867943
tamB	0.329981004	0.659929719	0.617057852	0.999867943
ycjY	0.329979449	0.750723254	0.660263985	0.999867943
xerD	0.329908125	0.825596949	0.689451528	0.999867943
yedI	0.328933211	0.815938284	0.686848976	0.999867943
stfR	0.328819782	0.722195276	0.648889171	0.999867943
psd	0.32818303	0.814022268	0.686828885	0.999867943
yojO	0.328085391	3.326654708	0.921437435	0.999867943
dinJ	0.327343237	0.87453556	0.708177239	0.999867943
creD	0.326758409	0.621187588	0.598872841	0.999867943
malS	0.32654466	0.575642271	0.570530674	0.999867943
purD	0.326302755	1.166702585	0.779723434	0.999867943
gntT	0.325753488	0.689775123	0.636740919	0.999867943
creC	0.325727674	0.871231442	0.708500717	0.999867943
yqiH	0.325542964	0.848881149	0.701351653	0.999867943
hypE	0.325467235	1.112316069	0.769825472	0.999867943
ompT	0.325168614	1.10221696	0.767983753	0.999867943
rppH	0.324297279	0.869259619	0.709094126	0.999867943
pyrD	0.324228053	0.698145369	0.642351604	0.999867943
yhdY	0.324162334	0.64584703	0.615725111	0.999867943
fsaB	0.324000704	0.896143457	0.717688304	0.999867943
yfeA	0.323360767	0.586590748	0.58145925	0.999867943
endA	0.32318672	1.038747008	0.755700797	0.999867943
cadA	0.322824234	0.847312839	0.703204963	0.999867943
cpsB	0.322662857	1.008138246	0.748924253	0.999867943
yieK	0.322580632	0.759397198	0.670993323	0.999867943
ygbL	0.322067719	0.828217636	0.697373453	0.999867943
dnaK	0.32158049	0.715504129	0.653109933	0.999867943
yfeS	0.321477423	0.905719355	0.722633387	0.999867943
flgL	0.321181136	0.779413042	0.680280214	0.999867943
yqhD	0.320889162	0.770176959	0.676939245	0.999867943
thiP	0.320725021	1.073505056	0.765119868	0.999867943

cspH	0.320030495	2.098002905	0.878760619	0.999867943
yfhL	0.319360268	2.208190083	0.88500668	0.999867943
leuS	0.318384624	0.773031404	0.680438333	0.999867943
cynX	0.318311553	1.248786996	0.798802966	0.999867943
hchA	0.317729599	0.754181821	0.673543002	0.999867943
acrZ	0.317492679	2.73791483	0.907683063	0.999867943
corA	0.316920092	0.831573854	0.703122537	0.999867943
fabD	0.315590493	1.162578229	0.78603924	0.999867943
lamB	0.314185411	0.748329746	0.674595849	0.999867943
clsC	0.313678078	0.663417275	0.636340397	0.999867943
ygbN	0.313600497	0.687834738	0.648444493	0.999867943
minD	0.313590546	0.814460497	0.700216262	0.999867943
setC	0.311633602	1.09173917	0.775301752	0.999867943
gntR	0.311419731	0.768985662	0.685496115	0.999867943
lepB	0.309872427	0.794775981	0.696620475	0.999867943
hycH	0.308293085	1.310417172	0.814004368	0.999867943
mnmG	0.308029955	0.705118993	0.662221449	0.999867943
iaaA	0.308006377	1.068043263	0.773052966	0.999867943
yfjM	0.306628109	2.47000675	0.901203956	0.999867943
paaY	0.306324444	0.833423575	0.713209153	0.999867943
hemY	0.305924724	0.900927656	0.734183116	0.999867943
yahB	0.305282507	0.732968771	0.677043155	0.999867943
yicS	0.305120071	2.058651637	0.882174237	0.999867943
ycaK	0.305065602	0.917845671	0.739608872	0.999867943
rodZ	0.304519425	0.933884497	0.74436515	0.999867943
yrdA	0.303770426	1.034818006	0.769102083	0.999867943
yafV	0.303721802	0.852937793	0.721773882	0.999867943
nhaR	0.303602255	0.795105281	0.702580742	0.999867943
xseB	0.303005397	1.353818083	0.822900912	0.999867943
tsx	0.30247388	1.020951625	0.767026481	0.999867943
gudX	0.30222751	0.800899753	0.705906108	0.999867943
mglA	0.30212603	0.887155199	0.733437855	0.999867943
tdcE	0.30055511	0.671127108	0.654271312	0.999867943
yjfL	0.300007177	1.039818721	0.772949696	0.999867943
argK	0.29971212	0.736442747	0.684028012	0.999867943
kdsA	0.298253659	0.911790882	0.74358656	0.999867943
ybcW	0.29814648	2.740225463	0.913358244	0.999867943
serX	0.298021865	2.101402884	0.887221845	0.999867943
ybfK	0.297133243	2.241105401	0.894522908	0.999867943
rnr	0.296524002	0.899880205	0.741766478	0.999867943
yeaO	0.296512169	1.205600546	0.805724332	0.999867943
yqeA	0.295757229	0.920097569	0.747876096	0.999867943
mltD	0.295706677	0.956954404	0.757315354	0.999867943
fdnG	0.295342183	0.789710425	0.708413281	0.999867943
alpA	0.29517943	2.518352973	0.906692611	0.999867943
yqiC	0.294904083	1.44382454	0.83815656	0.999867943
psuK	0.294901964	0.789306882	0.70868599	0.999867943
yaeI	0.29374644	0.687496202	0.669182748	0.999867943
sufD	0.293196166	0.923677282	0.750922861	0.999867943

yibA	0.293104721	1.152154187	0.799188766	0.999867943
frmB	0.293048533	0.783525176	0.708394709	0.999867943
fadA	0.292508966	1.150699647	0.799340301	0.999867943
sanA	0.291856411	0.895163532	0.74439638	0.999867943
rsxA	0.291529097	1.075064484	0.786257523	0.999867943
yiaW	0.29112291	2.355484936	0.901637044	0.999867943
cadB	0.290644595	0.797566188	0.715548592	0.999867943
lon	0.290623038	0.645887554	0.652739869	0.999867943
holA	0.290223086	0.824654101	0.724888424	0.999867943
bluF	0.289996843	0.704340011	0.680538447	0.999867943
ygjV	0.289681745	0.924082882	0.753915813	0.999867943
phoU	0.288728896	1.017191129	0.776525942	0.999867943
yncH	0.288165443	2.675651537	0.91423433	0.999867943
nac	0.287551709	0.798475103	0.71875285	0.999867943
djlC	0.287368807	0.649103141	0.657970655	0.999867943
yhfL	0.287057507	2.678908523	0.914666292	0.999867943
csgF	0.286724209	0.861753544	0.739344276	0.999867943
ygiS	0.286660693	0.682279374	0.674374793	0.999867943
yahL	0.28529933	0.596941485	0.632696349	0.999867943
mobB	0.284716771	1.013072819	0.778677611	0.999867943
wzyE	0.28468768	0.640715611	0.656805692	0.999867943
yjcF	0.284470494	1.110780794	0.797873947	0.999867943
bdm	0.284195377	2.779692033	0.918566275	0.999867943
ftsQ	0.283124562	0.976988617	0.771974525	0.999867943
ycjN	0.283101603	0.716290758	0.692671087	0.999867943
yqiK	0.282693118	0.638411052	0.657905073	0.999867943
serA	0.282159463	0.806675766	0.726503425	0.999867943
cobB	0.28128994	0.870141323	0.746491334	0.999867943
ydcK	0.281128875	0.730135475	0.700210305	0.999867943
ybbL	0.280347158	0.777041275	0.718257943	0.999867943
tatE	0.279874981	1.42202444	0.84397273	0.999867943
yfgF	0.278742495	0.592189052	0.63785606	0.999867943
aroK	0.27796759	0.909258302	0.759826937	0.999867943
yeeZ	0.27795484	0.839415804	0.740547116	0.999867943
cyoE	0.27772305	1.042282104	0.789887503	0.999867943
yigI	0.277495025	0.856015922	0.745809029	0.999867943
ybhC	0.276964341	0.723486369	0.701853959	0.999867943
spoT	0.276931105	0.68311551	0.685187731	0.999867943
ytfJ	0.276471015	0.843144283	0.742983682	0.999867943
yhhH	0.276122632	2.34825066	0.906395283	0.999867943
pepP	0.276073103	0.839964188	0.742403267	0.999867943
livG	0.276054818	0.962386907	0.774231844	0.999867943
ycaP	0.275574316	0.867611767	0.75077017	0.999867943
metK	0.275074539	0.742270372	0.710946382	0.999867943
dgoA	0.274668399	1.651856177	0.867937675	0.999867943
cyaA	0.274612287	0.617283761	0.656412228	0.999867943
yhiJ	0.274288034	1.239520381	0.824870047	0.999867943
amiC	0.273995538	0.670375901	0.682745703	0.999867943
yfjJ	0.273950419	1.201067521	0.819576968	0.999867943

yadH	0.272675604	0.730074469	0.708783695	0.999867943
yggW	0.272258582	0.71082214	0.701705471	0.999867943
yhgE	0.271755815	0.606246588	0.653965899	0.999867943
ydhX	0.271346773	0.882149092	0.758388694	0.999867943
yicG	0.270620376	0.854748838	0.751541015	0.999867943
caiE	0.270342788	0.987119314	0.784184199	0.999867943
ybjI	0.270182146	0.745533438	0.717052309	0.999867943
tolQ	0.27015523	0.838716348	0.747372699	0.999867943
rclA	0.269523455	0.920860047	0.769761665	0.999867943
rhtA	0.267553629	0.728387111	0.713377803	0.999867943
yqfG	0.267453	3.000108431	0.928964467	0.999867943
ybbJ	0.267146642	1.61410579	0.868544533	0.999867943
yjhI	0.266762486	0.990888927	0.787763986	0.999867943
qorA	0.265722228	0.721184575	0.712535889	0.999867943
nudC	0.265638053	0.733331155	0.717176587	0.999867943
nadR	0.265211801	0.635885647	0.676623787	0.999867943
pgpB	0.265204602	0.75190785	0.724306675	0.999867943
yiaB	0.263686336	1.997820323	0.894994564	0.999867943
rihB	0.263550391	0.776042126	0.734151485	0.999867943
yibG	0.262944868	2.314684598	0.909556018	0.999867943
yqhG	0.262802382	0.79712344	0.741635391	0.999867943
hemG	0.262761097	0.822874697	0.749483178	0.999867943
glpR	0.262608037	1.088081036	0.80928409	0.999867943
dinD	0.262534975	1.032324552	0.799252694	0.999867943
prpC	0.262373458	1.004726295	0.793985134	0.999867943
dfp	0.260043793	0.667520531	0.696856955	0.999867943
glnD	0.259765089	0.730499512	0.722140694	0.999867943
yfhM	0.259673631	0.700506498	0.710865379	0.999867943
yfiL	0.258144644	2.51266643	0.918171444	0.999867943
gspB	0.25713969	1.061477328	0.808588785	0.999867943
asd	0.25675624	0.840144908	0.75990201	0.999867943
betT	0.256619974	0.865536651	0.766858538	0.999867943
ndk	0.255659347	0.859407708	0.76609764	0.999867943
radA	0.255643588	1.241523375	0.836860264	0.999867943
tesB	0.255448381	0.742249604	0.73073026	0.999867943
yfbL	0.25531484	0.806106974	0.751451704	0.999867943
kefF	0.25512336	1.226747183	0.835254461	0.999867943
proC	0.254259352	0.853058412	0.765660267	0.999867943
gspH	0.253179254	1.150217841	0.825782234	0.999867943
atoD	0.252497838	0.885689667	0.775578157	0.999867943
wecD	0.251615798	1.187714629	0.832225075	0.999867943
aroA	0.251510307	0.67157705	0.708027149	0.999867943
ygiF	0.251415025	0.889074844	0.777343382	0.999867943
yhhI	0.250339828	0.814879206	0.758682799	0.999867943
focB	0.24842259	0.724017027	0.731510401	0.999867943
paaG	0.248014598	0.99339132	0.802846775	0.999867943
folK	0.247930222	0.86950314	0.775536801	0.999867943
yfdX	0.247717909	0.8349562	0.766707989	0.999867943
yfgG	0.247677353	2.67424396	0.926208759	0.999867943

yjdJ	0.247595324	1.293019256	0.848144703	0.999867943
cbrB	0.246285971	1.99607606	0.901802184	0.999867943
ycfQ	0.244974072	0.911908669	0.788207633	0.999867943
def	0.244718602	0.904920662	0.786828692	0.999867943
avtA	0.244100145	0.710408554	0.731143137	0.999867943
queC	0.243881158	0.800401183	0.76059568	0.999867943
ybaM	0.243458293	2.705593474	0.928300467	0.999867943
ybgL	0.243304163	1.54659816	0.874996052	0.999867943
yfcL	0.241740088	1.199143644	0.840234186	0.999867943
yfeD	0.241675788	0.90997962	0.790559812	0.999867943
fliI	0.241624705	1.445473072	0.867244469	0.999867943
ridA	0.240314734	1.088916992	0.825332644	0.999867943
yahO	0.240256165	0.75810213	0.751305869	0.999867943
panM	0.240047809	1.141329524	0.833415613	0.999867943
mraY	0.239958468	0.873293202	0.783489697	0.999867943
yadS	0.239106256	0.8294296	0.773133993	0.999867943
fau	0.238732508	0.926793977	0.796723642	0.999867943
pdxJ	0.23852904	1.230864366	0.846340423	0.999867943
istR	0.238418005	2.964910286	0.935908607	0.999867943
ptsG	0.237814484	0.777685881	0.759758748	0.999867943
yadC	0.237417344	1.134357336	0.834216561	0.999867943
ygjP	0.237321341	0.987188288	0.810019176	0.999867943
tktA	0.236344966	0.832186503	0.776406698	0.999867943
ybdK	0.236106485	0.672206707	0.725407632	0.999867943
yggS	0.235006474	0.904452249	0.794992652	0.999867943
speF	0.234091002	0.598148493	0.695531639	0.999867943
gyrA	0.234013506	1.078318044	0.828194981	0.999867943
csrD	0.232221193	0.639803841	0.71663711	0.999867943
dsrB	0.231738227	2.718504264	0.932066812	0.999867943
fucI	0.230982123	0.739325989	0.754719403	0.999867943
melB	0.230559321	0.69827454	0.741260676	0.999867943
nanT	0.230445988	0.86403158	0.789692343	0.999867943
ysaB	0.230106177	2.41843145	0.924198172	0.999867943
pppA	0.229983656	0.761742812	0.762714785	0.999867943
racC	0.229889656	2.453479684	0.92534792	0.999867943
gpt	0.229752862	0.842791643	0.785153542	0.999867943
efeB	0.229570412	0.759278791	0.762382787	0.999867943
rimO	0.22919481	0.676533003	0.734776628	0.999867943
aroF	0.2289348	0.683261667	0.73757856	0.999867943
sgrR	0.228280904	0.71710836	0.750230611	0.999867943
hofQ	0.228107425	0.804124837	0.776662017	0.999867943
mdaB	0.226829831	0.809046682	0.779196104	0.999867943
xylH	0.226067238	0.673346527	0.737069319	0.999867943
lgt	0.2254968	0.67263749	0.737442023	0.999867943
ispF	0.225314135	1.173403736	0.847728442	0.999867943
insL1	0.225067342	1.265042231	0.858791374	0.999867943
perR	0.224118416	0.546120558	0.681525253	0.999867943
adiY	0.223903942	1.109771493	0.840106858	0.999867943
waaS	0.223864354	1.057398002	0.832331351	0.999867943

yjfK	0.223709626	0.822013342	0.785507906	0.999867943
pabB	0.223680203	0.767948718	0.770845049	0.999867943
tdcG	0.223583929	0.662188602	0.735631571	0.999867943
yaiO	0.222740033	0.886224047	0.801554321	0.999867943
nfsA	0.222228996	0.819259117	0.786194103	0.999867943
yehA	0.221524139	0.815986949	0.786021878	0.999867943
ascB	0.221228112	0.665681626	0.739637666	0.999867943
polB	0.220709286	0.795592361	0.781461435	0.999867943
nuoN	0.220611667	1.093400922	0.840099279	0.999867943
dkgA	0.220575588	0.843081198	0.79360648	0.999867943
scpB	0.220269215	0.811401057	0.786031305	0.999867943
hicA	0.219195537	2.864580442	0.939006002	0.999867943
maa	0.21909647	1.102075806	0.842416499	0.999867943
yedE	0.218708117	0.88141454	0.80403143	0.999867943
yphD	0.218617172	1.050911005	0.835208399	0.999867943
yncJ	0.218339072	2.687511514	0.935249421	0.999867943
yqjI	0.217838404	0.972000667	0.822669042	0.999867943
yahM	0.217601219	0.896887921	0.808301279	0.999867943
syd	0.217569061	1.022160207	0.831442238	0.999867943
fdnI	0.217121352	0.977685455	0.824254022	0.999867943
rdgB	0.216297238	1.064015987	0.838913198	0.999867943
yafQ	0.215630008	1.131365781	0.848844745	0.999867943
metZ	0.215550417	2.520508391	0.931849088	0.999867943
hlyE	0.215546992	0.743275102	0.771819107	0.999867943
pqqL	0.215394343	0.659639255	0.744020715	0.999867943
guaA	0.214595672	0.954154387	0.822051892	0.999867943
ybcV	0.214401218	2.003428294	0.914775362	0.999867943
yebW	0.213988305	2.6468532	0.935564181	0.999867943
ynhF	0.213950753	2.976279273	0.942693182	0.999867943
ybhN	0.213156487	0.693374828	0.758524588	0.999867943
rnlA	0.212805231	0.780120382	0.785018399	0.999867943
ymgF	0.211815392	2.570177897	0.934318509	0.999867943
apt	0.211810104	0.957057118	0.824847979	0.999867943
dosC	0.210659654	0.751591298	0.779258973	0.999867943
yadG	0.210472785	0.687416006	0.759467874	0.999867943
ypjA	0.209163713	0.577014363	0.716983594	0.999867943
gsk	0.2089754	0.71543692	0.770214147	0.999867943
hdeA	0.208595379	1.995949547	0.916765153	0.999867943
yiiR	0.208109136	0.868368451	0.81059746	0.999867943
yhdJ	0.207928156	0.713648795	0.770776601	0.999867943
ccmH	0.207110144	0.881902625	0.814329284	0.999867943
gdhA	0.206275419	0.784078393	0.792488845	0.999867943
nupX	0.205544477	0.82489995	0.803225387	0.999867943
ybgD	0.205387224	1.066382945	0.847270873	0.999867943
yajR	0.20533926	0.766521255	0.788788127	0.999867943
yeeO	0.204605935	0.60421101	0.734886035	0.999867943
rdgC	0.204021173	0.737120529	0.781948047	0.999867943
adiA	0.203646735	0.599963038	0.734284214	0.999867943
argA	0.20318646	0.668016703	0.761003147	0.999867943

yfiB	0.20297834	0.936433879	0.828397939	0.999867943
ftsK	0.201308831	0.711422234	0.777202332	0.999867943
gspI	0.201144778	1.045040241	0.847369656	0.999867943
insJ	0.20025608	0.662126427	0.762313715	0.999867943
casB	0.200186185	1.065334283	0.850947778	0.999867943
aas	0.199612517	0.635179954	0.753322541	0.999867943
yhdU	0.198873259	2.658899846	0.940377567	0.999867943
mdtK	0.198505128	0.752977421	0.792067403	0.999867943
hyfG	0.198256096	0.761956985	0.794714679	0.999867943
mdlB	0.198168176	0.874591117	0.820747393	0.999867943
tsaB	0.196548106	1.081096359	0.855736209	0.999867943
kduD	0.19625268	0.815480462	0.809819449	0.999867943
ydhF	0.196191546	0.785817346	0.802845976	0.999867943
slyA	0.195970625	0.969458604	0.83980384	0.999867943
ygiL	0.195666579	1.184532776	0.868798682	0.999867943
tnaB	0.195194559	0.675178736	0.772504416	0.999867943
yodC	0.195069169	2.592322279	0.940016749	0.999867943
yghW	0.195067891	2.061219287	0.924603052	0.999867943
rnc	0.194038255	0.804881235	0.809495533	0.999867943
ycgG	0.193688739	0.74347547	0.794464237	0.999867943
malI	0.193401722	0.871636736	0.824404654	0.999867943
acrF	0.193393465	0.562223414	0.730861521	0.999867943
yjcH	0.192991585	1.1324045	0.864674837	0.999867943
dmsC	0.191868784	0.910365489	0.833074421	0.999867943
yidA	0.19065052	0.85725495	0.824005219	0.999867943
fsaA	0.190587006	0.942553175	0.839758089	0.999867943
rhaS	0.190150426	0.873571743	0.827686182	0.999867943
ppsA	0.189984249	0.735140198	0.796073001	0.999867943
yiiE	0.189367513	2.449533625	0.938378862	0.999867943
yraI	0.188324099	1.122315154	0.866740891	0.999867943
ilvI	0.188011206	0.628657805	0.764888436	0.999867943
ecpD	0.18739623	0.721044617	0.794944405	0.999867943
paaE	0.187212343	0.970152653	0.84698086	0.999867943
yeiB	0.186688501	0.967708296	0.847023065	0.999867943
yphH	0.18639942	0.667286247	0.77998428	0.999867943
lysU	0.186079619	0.985341362	0.850212051	0.999867943
yjbF	0.185335224	0.935839277	0.843012419	0.999867943
ydfH	0.185239736	0.807380781	0.818532447	0.999867943
mqsR	0.184205838	2.462259819	0.940364534	0.999867943
ynfL	0.183937997	0.801592551	0.818506976	0.999867943
yhjY	0.183920486	1.433058603	0.897878948	0.999867943
adhP	0.183549002	0.826327418	0.824215612	0.999867943
ygjI	0.183212998	0.683326797	0.788607608	0.999867943
pinQ	0.182555281	0.768705148	0.812281222	0.999867943
metL	0.182134726	0.918942287	0.842888277	0.999867943
citT	0.181797508	0.681825675	0.789751425	0.999867943
gltX	0.181732499	0.762778552	0.811686682	0.999867943
yaiI	0.180962238	1.051106673	0.863308966	0.999867943
yhhQ	0.180797471	0.851881424	0.831925152	0.999867943

pth	0.179745965	0.935104938	0.847569801	0.999867943
carA	0.179510659	0.664012976	0.786897083	0.999867943
isrC	0.178833871	2.60137569	0.945191894	0.999867943
yjhU	0.178349337	1.043522988	0.864293913	0.999867943
mlaA	0.17805399	0.760445895	0.814873039	0.999867943
yiiS	0.17760306	2.481407629	0.94294132	0.999867943
chaB	0.177421078	2.563475614	0.944821553	0.999867943
yicI	0.176848208	0.678644599	0.794408461	0.999867943
umuC	0.176157211	0.701236016	0.801652071	0.999867943
asnC	0.176054053	1.139400159	0.877203936	0.999867943
speE	0.175799439	0.930107113	0.850085068	0.999867943
yedV	0.175632714	0.799039471	0.826023178	0.999867943
yfdV	0.175590883	0.87846358	0.841571243	0.999867943
chbB	0.175362994	1.028858191	0.864660751	0.999867943
pgaA	0.175267502	0.623928696	0.778779809	0.999867943
cpsG	0.174903175	0.971798066	0.857169111	0.999867943
hofN	0.174560218	1.268087395	0.890512059	0.999867943
yedY	0.174533118	0.957625634	0.855381745	0.999867943
yigL	0.174525256	0.879715661	0.842741305	0.999867943
aroP	0.174456994	0.681812946	0.798049504	0.999867943
nlpA	0.174151845	0.83723504	0.835222479	0.999867943
hflC	0.174013	1.022465651	0.864861046	0.999867943
hisB	0.173900427	0.840894591	0.836162875	0.999867943
rhlB	0.173543545	0.860339949	0.84013947	0.999867943
nanS	0.173529587	0.894893581	0.846245677	0.999867943
potB	0.173488386	0.849262978	0.838133829	0.999867943
ampD	0.1733672	0.912041929	0.849241055	0.999867943
chbA	0.172825602	1.164252245	0.881992824	0.999867943
yoeB	0.172639513	2.860902059	0.951881314	0.999867943
mdh	0.172578925	1.070428829	0.8719169	0.999867943
hyfF	0.171734319	0.799888041	0.830002824	0.999867943
ydeP	0.171580015	0.659868989	0.794847317	0.999867943
cpdB	0.170662039	0.663718432	0.797078282	0.999867943
ytfI	0.170577034	0.97960786	0.861764968	0.999867943
rsmD	0.170100098	0.838677784	0.839276219	0.999867943
emrY	0.169888544	0.79807279	0.831425637	0.999867943
aegA	0.169822514	0.872999066	0.845762696	0.999867943
ygcG	0.169202146	0.713471629	0.81253787	0.999867943
speD	0.169103463	0.821418591	0.836894345	0.999867943
hyfR	0.168585278	0.659365995	0.798199603	0.999867943
dapE	0.167881072	0.760511031	0.825288877	0.999867943
acpS	0.167807034	1.160623974	0.885039754	0.999867943
trg	0.167688807	0.687737914	0.807365107	0.999867943
ecpE	0.167637779	0.721805329	0.816345468	0.999867943
gtrB	0.167590408	0.82578099	0.839175858	0.999867943
waaU	0.167536171	1.414958389	0.905747871	0.999867943
malG	0.16740106	0.716625462	0.815298396	0.999867943
ybdO	0.167330583	1.206042797	0.889652853	0.999867943
adhE	0.166735233	0.797186921	0.834327629	0.999867943

yeeJ	0.166666854	0.535136979	0.755460762	0.999867943
bamC	0.166047196	1.080652517	0.877882108	0.999867943
ecpB	0.16599877	1.014061556	0.86996973	0.999867943
ubiH	0.165788933	1.33434702	0.901119521	0.999867943
srmB	0.165002536	0.794073861	0.835391045	0.999867943
argS	0.163843952	0.684363071	0.810787009	0.999867943
rffH	0.163510611	0.786215025	0.83525093	0.999867943
tufB	0.162751909	0.868028323	0.851271696	0.999867943
hycI	0.162396146	0.9291601	0.861254581	0.999867943
yijE	0.162203013	0.800295165	0.839385949	0.999867943
yqfA	0.16219018	0.891358033	0.855615286	0.999867943
otsB	0.161718803	0.789731941	0.83774649	0.999867943
citF	0.161391501	1.068430304	0.879932506	0.999867943
dpiB	0.161354482	0.612273792	0.792139625	0.999867943
yihF	0.160782761	0.859187816	0.85155602	0.999867943
moaA	0.160705184	0.968694617	0.868236665	0.999867943
ydjK	0.160377034	0.71486023	0.822486996	0.999867943
acnA	0.160333685	0.788637544	0.838896916	0.999867943
ybhK	0.160202151	0.74172606	0.828998969	0.999867943
ampE	0.158983787	0.794235872	0.841346061	0.999867943
panF	0.158803394	0.816636694	0.8458155	0.999867943
yaaA	0.158803203	0.810161089	0.84459893	0.999867943
uvrA	0.158642829	0.824221335	0.847369362	0.999867943
gshB	0.158610891	0.796908963	0.842237216	0.999867943
ydeR	0.158348343	0.976261949	0.871149445	0.999867943
yhaO	0.158180307	0.648812899	0.807386052	0.999867943
ycgM	0.158042514	0.980921079	0.872001691	0.999867943
nrdD	0.15793566	0.666778807	0.812762573	0.999867943
phnC	0.157661903	1.048822186	0.880509934	0.999867943
secY	0.156817491	1.355867834	0.907923285	0.999867943
argD	0.155994617	0.706857652	0.825335799	0.999867943
ydjH	0.155328802	0.822284325	0.850171868	0.999867943
yfeX	0.15433029	0.961850521	0.872525481	0.999867943
bglB	0.153710133	0.88192627	0.861638268	0.999867943
yihQ	0.153605243	0.635936858	0.809135334	0.999867943
yhjK	0.153276107	0.611472117	0.80207129	0.999867943
ydiR	0.153013169	1.006961741	0.879222195	0.999867943
hinT	0.152879705	1.288705609	0.905568153	0.999867943
caiF	0.152697921	2.414077875	0.949564958	0.999867943
yidQ	0.152060904	1.139725691	0.89386213	0.999867943
insL1	0.151924349	1.470366492	0.917705719	0.999867943
yjdF	0.150984516	0.839048275	0.8571939	0.999867943
sdaB	0.150819498	0.703066583	0.830144173	0.999867943
araE	0.150810891	0.756095257	0.841902794	0.999867943
asnS	0.149626046	0.727729323	0.837098121	0.999867943
yddG	0.149297667	0.818966802	0.85534728	0.999867943
mdtF	0.149203545	0.54545092	0.784436881	0.999867943
livK	0.146390595	0.714640827	0.837693234	0.999867943
ykfH	0.145664634	1.215969273	0.90464711	0.999867943

nei	0.14523097	0.766203038	0.849664651	0.999867943
ykfI	0.144699121	1.008820072	0.885947415	0.999867943
trkH	0.143453326	0.596955553	0.810091324	0.999867943
ydcD	0.143434272	2.396262611	0.952269135	0.999867943
hycF	0.142727193	1.217681103	0.906691826	0.999867943
tolA	0.142040013	0.748341748	0.849460883	0.999867943
fucR	0.141332978	0.941194279	0.880635664	0.999867943
ymgJ	0.139644437	2.144097363	0.948070726	0.999867943
betI	0.138665683	1.079695414	0.897808397	0.999867943
yiaF	0.137599826	0.926105577	0.881885857	0.999867943
yajQ	0.137550304	0.898318921	0.878303898	0.999867943
npr	0.136201336	2.604884799	0.958300096	0.999867943
pstC	0.136191668	0.687537397	0.842977625	0.999867943
metA	0.135913897	0.970223553	0.888592726	0.999867943
narK	0.135807568	0.701379405	0.846466194	0.999867943
ybeQ	0.135768589	0.824589701	0.869219575	0.999867943
intD	0.135359621	0.878529766	0.877550468	0.999867943
tsaE	0.134967683	1.024147287	0.895154017	0.999867943
ygbI	0.134674407	0.762739165	0.859848681	0.999867943
entE	0.134300249	0.954588325	0.888115493	0.999867943
wecF	0.133730501	0.690529049	0.846439114	0.999867943
waaQ	0.133027274	0.777968724	0.864229201	0.999867943
glnK	0.13290419	2.075105154	0.94893283	0.999867943
macA	0.132889792	0.751597621	0.859657826	0.999867943
pstS	0.132522811	0.795102997	0.867626748	0.999867943
dcuR	0.132327949	0.88312859	0.880890885	0.999867943
rhsC	0.131775001	0.926160819	0.886858128	0.999867943
ppc	0.131599835	0.64303518	0.837842325	0.999867943
ynjA	0.131509195	1.42619974	0.926531573	0.999867943
rplT	0.130549707	1.537813891	0.932346419	0.999867943
wcaN	0.130440865	0.790722399	0.868972183	0.999867943
yehE	0.130249719	2.549396351	0.959253473	0.999867943
cvpA	0.129623857	0.911947916	0.886969787	0.999867943
adk	0.128112097	0.916371912	0.888815165	0.999867943
ydaS	0.127782903	2.780112377	0.963339569	0.999867943
narU	0.127673215	0.719383413	0.859134598	0.999867943
ymfA	0.127577574	1.092846057	0.907067016	0.999867943
nhaA	0.12755514	0.764416849	0.86747553	0.999867943
yigG	0.127304657	2.411075371	0.957891302	0.999867943
iraM	0.127302501	2.470192679	0.958898851	0.999867943
fliR	0.127164717	0.766650716	0.868258884	0.999867943
yjcZ	0.12706031	0.804733724	0.874542594	0.999867943
rlmD	0.125394421	0.64816502	0.846598227	0.999867943
tonB	0.125355222	0.881407705	0.886904964	0.999867943
yjbG	0.124551546	1.405310526	0.929376602	0.999867943
fabH	0.12378509	1.00247084	0.901727018	0.999867943
srlA	0.123427444	1.178416613	0.916582062	0.999867943
yjbH	0.123210338	0.644376407	0.848362133	0.999867943
yjbD	0.122550633	2.095168279	0.953356716	0.999867943

yehK	0.122430224	2.518901394	0.961234395	0.999867943
pepA	0.121721969	0.778606985	0.875770543	0.999867943
hdeB	0.121608274	2.395066528	0.959505221	0.999867943
hycC	0.12148084	1.119115799	0.913558839	0.999867943
ihfB	0.120758316	1.335266495	0.927939468	0.999867943
napF	0.120463283	1.047072166	0.908407284	0.999867943
ydfI	0.119953878	0.742279671	0.871619284	0.999867943
yccF	0.119925602	0.88411025	0.892101512	0.999867943
psuG	0.119915301	0.781648842	0.878072385	0.999867943
mipA	0.11950751	0.963643219	0.901302334	0.999867943
aslA	0.119335324	0.617863712	0.846847899	0.999867943
glnS	0.11919534	0.685280397	0.861915291	0.999867943
yobH	0.119122212	2.776539699	0.965778763	0.999867943
casD	0.117848623	1.129422523	0.916896249	0.999867943
wzzE	0.117667427	0.756241737	0.876352273	0.999867943
yhjE	0.117517426	0.678368631	0.86246644	0.999867943
trmI	0.117151632	0.834320253	0.888331583	0.999867943
fimZ	0.117147899	1.163534868	0.9198023	0.999867943
cspF	0.11506663	2.498488329	0.963266811	0.999867943
yciM	0.114958297	0.824970197	0.889174826	0.999867943
hscA	0.11489252	1.173335689	0.921996164	0.999867943
kdgT	0.114674954	0.652510388	0.860494864	0.999867943
mngR	0.11442627	0.807882385	0.887366512	0.999867943
xylE	0.114235707	0.778842907	0.883389644	0.999867943
yaiZ	0.114150855	2.604740656	0.965044482	0.999867943
yciX	0.112354584	2.80282513	0.968024411	0.999867943
yjfF	0.111651857	0.67142942	0.867928901	0.999867943
hycB	0.111592518	1.344225774	0.933838657	0.999867943
nupG	0.111200043	0.855000298	0.896520155	0.999867943
yihN	0.111199295	0.864403526	0.897640293	0.999867943
yqeJ	0.109274298	2.368418399	0.963200188	0.999867943
yeeE	0.109174195	0.725279001	0.880348717	0.999867943
ygcU	0.109066176	0.674887891	0.871615848	0.999867943
ppiA	0.108163047	0.900882099	0.904432856	0.999867943
iscR	0.107711607	1.041161047	0.917603165	0.999867943
pptA	0.106621105	2.902948555	0.970701441	0.999867943
rzpQ	0.104748959	1.052859685	0.920749266	0.999867943
yfiN	0.104666433	0.705531215	0.882065576	0.999867943
yihP	0.104327627	0.69088907	0.87997186	0.999867943
hupB	0.102718594	1.157377385	0.929279668	0.999867943
baeS	0.102309362	1.096773451	0.92567943	0.999867943
yccE	0.102013201	0.9138972	0.911121246	0.999867943
essQ	0.101984987	2.73006385	0.970200944	0.999867943
yidZ	0.101880467	0.827472607	0.902010122	0.999867943
ydcV	0.101382531	1.106972731	0.927027459	0.999867943
yqaE	0.101343963	2.707679403	0.970143471	0.999867943
ybgF	0.101318673	0.858630988	0.906067458	0.999867943
yfgO	0.101219698	0.666811026	0.879347303	0.999867943
amiA	0.100940196	0.746197885	0.892396276	0.999867943

mdtL	0.100821564	0.613144215	0.869389747	0.999867943
yagK	0.100319748	0.765530708	0.895738907	0.999867943
rraA	0.10013689	1.17672522	0.932183529	0.999867943
yoaB	0.099796908	0.942360486	0.915660909	0.999867943
dcuC	0.099381152	0.671218289	0.882294744	0.999867943
bamD	0.098731225	1.083581071	0.927400675	0.999867943
glcF	0.097932968	0.984262386	0.920742207	0.999867943
yhjA	0.097727681	0.72278913	0.892446538	0.999867943
sfmC	0.096343804	1.096835586	0.930005464	0.999867943
yadK	0.09626797	1.112818848	0.931062428	0.999867943
glpT	0.096062726	0.746155551	0.897560562	0.999867943
yjeJ	0.09605731	0.772866883	0.901088036	0.999867943
yhbX	0.095991661	0.906198376	0.915639584	0.999867943
fixB	0.095568346	1.059055653	0.928097126	0.999867943
mdtJ	0.095294464	0.973644141	0.922032332	0.999867943
ptsI	0.094991613	0.926207705	0.918312404	0.999867943
yggC	0.094742745	0.737767963	0.897818124	0.999867943
ydfZ	0.094023644	2.765680051	0.972879883	0.999867943
ybbC	0.093967621	2.331457352	0.967850572	0.999867943
wbbK	0.093155675	0.880172625	0.915710918	0.999867943
yqgF	0.092974392	0.991688243	0.925304851	0.999867943
yqiI	0.092267785	0.89003547	0.917433189	0.999867943
wcaM	0.091450415	0.728995888	0.900169608	0.999867943
mdtB	0.091183349	0.97613419	0.925575686	0.999867943
potA	0.090414414	0.701220607	0.897406221	0.999867943
dsbA	0.09007492	0.859297189	0.91651551	0.999867943
epmC	0.089971989	0.946178919	0.924243479	0.999867943
patA	0.089736507	0.780380901	0.908452533	0.999867943
ecnB	0.089540025	2.851070707	0.974945953	0.999867943
wbbJ	0.088876977	0.872955929	0.918906275	0.999867943
rplR	0.088862958	1.556901789	0.954484025	0.999867943
gabP	0.088829501	0.683890251	0.896654617	0.999867943
pykF	0.088640708	0.752229701	0.906196584	0.999867943
yjhB	0.088162303	1.108403646	0.936603205	0.999867943
ubiB	0.088137889	0.624496384	0.887763811	0.999867943
fryA	0.087313537	0.890797041	0.921918542	0.999867943
tehA	0.086877784	0.768856899	0.910033696	0.999867943
yaaU	0.086335896	0.659994634	0.895923217	0.999867943
yjbT	0.085742095	2.559274315	0.97327387	0.999867943
lsrK	0.085712906	0.700214399	0.902574702	0.999867943
cyoA	0.085283592	1.051617199	0.935364364	0.999867943
torA	0.085173316	0.858650591	0.920983934	0.999867943
hisJ	0.084327463	0.810316383	0.917115917	0.999867943
pstB	0.083855226	0.829561907	0.91948398	0.999867943
sgrS	0.082937459	3.260301267	0.979705133	0.999867943
cysZ	0.082880831	0.719293874	0.908266569	0.999867943
ycdT	0.082493253	0.832053394	0.921023793	0.999867943
casA	0.081841654	1.075400357	0.939336813	0.999867943
rna	0.081768766	0.698810085	0.906850998	0.999867943

rimJ	0.081733102	0.813432267	0.919963818	0.999867943
yhhJ	0.080998123	0.727533287	0.911352659	0.999867943
hdeD	0.080187661	1.971679236	0.967559194	0.999867943
yhjR	0.079996353	2.555159882	0.975024095	0.999867943
torI	0.079703887	2.59667103	0.975513065	0.999867943
ykfB	0.079683232	0.742061895	0.914486851	0.999867943
yafY	0.079412728	0.933322205	0.932192956	0.999867943
pagP	0.079274644	1.016930621	0.937863991	0.999867943
yihW	0.077986043	0.805552909	0.922876823	0.999867943
arpA	0.077965679	0.938432304	0.933787316	0.999867943
xapB	0.077852711	0.694018637	0.910683314	0.999867943
adiC	0.076817583	0.677235703	0.909691134	0.999867943
yiiG	0.076017701	0.863007712	0.929809426	0.999867943
recC	0.075785636	0.589682695	0.897737987	0.999867943
ileS	0.075397313	0.785196318	0.923501894	0.999867943
gfcB	0.074484268	0.87700969	0.932317191	0.999867943
frlC	0.074267644	0.880155974	0.932754234	0.999867943
dmsB	0.074150731	0.858184058	0.931145086	0.999867943
ecpA	0.074021292	0.84311696	0.930039764	0.999867943
truD	0.073705478	0.762077953	0.922951572	0.999867943
evgS	0.073378862	0.688918238	0.91517521	0.999867943
usg	0.073146589	0.937993735	0.937842404	0.999867943
fhuB	0.073079057	1.222921268	0.952348554	0.999867943
ybhI	0.072692072	0.695319113	0.916736933	0.999867943
yagA	0.072624441	1.044734884	0.944579926	0.999867943
paaH	0.072297845	0.796014735	0.927631838	0.999867943
ycjG	0.072208991	0.915637979	0.937142433	0.999867943
yfeO	0.071611675	0.762854794	0.925209826	0.999867943
entB	0.070117995	0.859814224	0.935004429	0.999867943
ybdL	0.070036682	0.755224045	0.92611306	0.999867943
rpsR	0.069938679	1.616600697	0.965492042	0.999867943
kdpA	0.068447796	0.869478156	0.937253073	0.999867943
yegJ	0.068097088	2.320943111	0.976593213	0.999867943
lpxD	0.067485963	0.897694825	0.94007392	0.999867943
ydcZ	0.067251799	1.046914779	0.948780654	0.999867943
yhcO	0.066703604	2.468401071	0.978441389	0.999867943
surA	0.066455018	0.732137067	0.927676502	0.999867943
fixC	0.065648329	0.761731353	0.931320907	0.999867943
feaB	0.065100697	0.682166839	0.923971523	0.999867943
ykgD	0.064836599	0.552631046	0.906603717	0.999867943
pepN	0.063969962	0.733639046	0.930516182	0.999867943
gspA	0.06380412	0.732985561	0.930634234	0.999867943
epmA	0.063385857	0.833424819	0.939375596	0.999867943
xylG	0.062985392	1.106539019	0.954608065	0.999867943
cysW	0.062348721	1.141716063	0.956449446	0.999867943
ychF	0.062023702	0.681952676	0.927532197	0.999867943
yrhB	0.062008937	2.430599527	0.979646747	0.999867943
fecE	0.061576961	0.909322477	0.946010592	0.999867943
astB	0.061066677	1.048398957	0.953551441	0.999867943

topA	0.060844139	0.718776766	0.932539995	0.999867943
cheW	0.060062816	0.987781524	0.951513895	0.999867943
yniD	0.059863418	3.095110182	0.984568847	0.999867943
yiaC	0.058900566	0.921410957	0.94903049	0.999867943
lysR	0.058314502	0.744359807	0.937556152	0.999867943
yraJ	0.057577319	0.793426787	0.942149968	0.999867943
atpB	0.057495573	1.096584051	0.958184856	0.999867943
dmlA	0.057332995	0.708475398	0.935502021	0.999867943
yajG	0.05681125	0.88133385	0.948603531	0.999867943
yahF	0.056635256	1.071395529	0.957842497	0.999867943
glsB	0.056151864	0.804799491	0.944375733	0.999867943
uxaA	0.05591649	0.941652125	0.952648439	0.999867943
yahN	0.05589428	0.83817819	0.946832127	0.999867943
insL1	0.055224732	1.532084014	0.97124608	0.999867943
hofO	0.055208626	1.196907384	0.963209773	0.999867943
ydhZ	0.055180788	2.528442261	0.982588329	0.999867943
zraR	0.05421107	1.007374283	0.957083173	0.999867943
sgcB	0.053533347	2.580747522	0.983450389	0.999867943
yagM	0.052649991	0.718029422	0.941546962	0.999867943
sucA	0.052600822	1.146648393	0.963411043	0.999867943
uhpB	0.05252494	0.999053741	0.958070784	0.999867943
ybfB	0.052229711	2.556983532	0.983703305	0.999867943
tgt	0.052181823	0.764613149	0.945589775	0.999867943
ugd	0.051553909	0.925057842	0.955556525	0.999867943
waaF	0.051469768	0.733721323	0.94407512	0.999867943
yniB	0.051307265	0.847736769	0.951739387	0.999867943
yhdZ	0.051253513	0.891521816	0.95415494	0.999867943
atpC	0.05108086	1.179271438	0.965449949	0.999867943
insD1	0.05063609	1.267400966	0.968130837	0.999867943
oppF	0.05015428	0.722262331	0.944638968	0.999867943
chbF	0.049967229	0.764482453	0.947886688	0.999867943
argO	0.049728808	0.762862848	0.948025042	0.999867943
mdtD	0.048184459	0.853819046	0.954996039	0.999867943
garR	0.048096085	0.831194762	0.95385712	0.999867943
ygfQ	0.047822298	0.610770637	0.937590775	0.999867943
rssB	0.047786026	0.734468641	0.948124606	0.999867943
bcsE	0.047384104	0.715656633	0.947210104	0.999867943
nuoM	0.047158585	0.914502111	0.958873318	0.999867943
mscM	0.04714167	0.806026619	0.953361122	0.999867943
fadL	0.047030891	0.719862417	0.947908736	0.999867943
ychE	0.046432197	0.895812593	0.958662167	0.999867943
yghQ	0.046408176	0.703786398	0.947425027	0.999867943
kilR	0.046163614	2.483088188	0.985167215	0.999867943
nagD	0.045135387	0.79684309	0.954829777	0.999867943
der	0.044810877	0.690584617	0.948262919	0.999867943
yieH	0.044737824	1.879571781	0.981010436	0.999867943
phoQ	0.044628951	0.668344645	0.946760543	0.999867943
cusF	0.044480946	2.400371451	0.985215359	0.999867943
yfjS	0.044346568	2.384759348	0.985163533	0.999867943

setA	0.044191633	0.83714	0.957900169	0.999867943
pheT	0.04356211	0.818648686	0.957562824	0.999867943
ydeN	0.042182276	0.766865289	0.956133596	0.999867943
proP	0.041178601	0.622741267	0.947278519	0.999867943
yhjJ	0.040516641	0.771611682	0.958123039	0.999867943
yehU	0.03807411	0.643855286	0.952844918	0.999867943
ykgO	0.038059585	2.721392093	0.988841679	0.999867943
ydfO	0.036979073	2.394816612	0.987680118	0.999867943
rbsR	0.036375733	0.839521565	0.965439177	0.999867943
birA	0.035047507	0.780262121	0.964172983	0.999867943
dgt	0.034632666	0.659384786	0.958112212	0.999867943
yagL	0.034079295	0.941246132	0.971117646	0.999867943
bioA	0.03400384	0.982280818	0.972384963	0.999867943
ydfA	0.033191389	2.773420593	0.990451407	0.999867943
ybeF	0.032546109	1.10598252	0.976523779	0.999867943
yijD	0.032389923	0.963755607	0.973189722	0.999867943
yicL	0.032195267	0.951175128	0.972998451	0.999867943
hofP	0.031677798	0.936512716	0.973016483	0.999867943
ybjT	0.031584369	0.773716195	0.967438082	0.999867943
prpB	0.031451227	1.080060356	0.976768981	0.999867943
pgaB	0.031410568	0.701216883	0.964271213	0.999867943
cfa	0.031341827	0.6732779	0.962871006	0.999867943
potH	0.03109381	0.787313594	0.968496894	0.999867943
ttdB	0.030892376	0.881709848	0.972050321	0.999867943
ymgI	0.030439115	2.66748993	0.990895421	0.999867943
waaA	0.030290106	0.70446064	0.965703459	0.999867943
ygaZ	0.030203415	0.779886947	0.969107297	0.999867943
acrR	0.029581406	0.998777513	0.976372018	0.999867943
ynaK	0.029574703	2.507546591	0.990589745	0.999867943
csiR	0.029417134	0.907934354	0.974153009	0.999867943
yggL	0.028436339	2.493905501	0.990902452	0.999867943
efp	0.028408701	1.09425407	0.979287884	0.999867943
ysaA	0.028341712	1.235267979	0.981695121	0.999867943
pta	0.027274563	0.913424957	0.976178974	0.999867943
marC	0.02685753	0.815176102	0.973716928	0.999867943
ulaG	0.026735589	0.845869624	0.974785284	0.999867943
yhhY	0.026555996	2.061234062	0.989720705	0.999867943
dtpD	0.026403793	0.638504344	0.967014828	0.999867943
eutD	0.025663672	1.323071508	0.984524371	0.999867943
ymiA	0.025608762	2.648796393	0.992286113	0.999867943
insF1	0.025238394	1.261401468	0.984036818	0.999867943
yaiL	0.023370237	0.582342057	0.967988321	0.999867943
yoaC	0.022594374	1.034083141	0.982567873	0.999867943
yhgN	0.022214998	0.923631952	0.980811302	0.999867943
yjgR	0.021661244	0.594362667	0.970927941	0.999867943
hcaF	0.021419623	1.053489481	0.983778472	0.999867943
yaeR	0.021186353	0.977999502	0.982716819	0.999867943
ydiF	0.020753257	0.703822639	0.976476597	0.999867943
yhbT	0.020712791	0.891241951	0.981458538	0.999867943

chiP	0.020386357	0.716936913	0.977314925	0.999867943
dtpA	0.020272122	0.782325774	0.979327022	0.999867943
ydhS	0.019926244	0.727421703	0.978146302	0.999867943
flgK	0.019904073	0.7142113	0.977766948	0.999867943
yjeT	0.019659323	2.503475642	0.993734427	0.999867943
ycjO	0.018944593	0.960588272	0.984265248	0.999867943
yidD	0.018895307	1.206113622	0.987500633	0.999867943
rlmM	0.018569557	0.713460763	0.979235452	0.999867943
ynjI	0.018505961	0.74323946	0.98013548	0.999867943
nfrB	0.018349596	0.672918013	0.978245425	0.999867943
eutA	0.017964192	1.27990462	0.988801603	0.999867943
yagU	0.01786221	0.616983149	0.97690376	0.999867943
yeeR	0.017583823	0.780326176	0.982022039	0.999867943
yeeT	0.017301092	2.851527978	0.99515902	0.999867943
anmK	0.016927381	0.771670536	0.982498991	0.999867943
dkgB	0.016846921	1.136898141	0.988177124	0.999867943
sufS	0.016731885	0.985621364	0.986455781	0.999867943
lipA	0.016631417	0.837447609	0.984155332	0.999867943
ispU	0.015938481	0.957238421	0.986715451	0.999867943
slmA	0.014481881	0.932243242	0.987605805	0.999867943
flk	0.014112066	0.777253985	0.985514155	0.999867943
ydjM	0.01351454	0.977590907	0.988970131	0.999867943
yceF	0.013344554	0.88582313	0.987980659	0.999867943
ydbD	0.013127378	0.749166741	0.986019673	0.999867943
arfA	0.012831908	2.581916963	0.996034598	0.999867943
msbA	0.012752863	0.729292547	0.986048405	0.999867943
napA	0.011694629	0.867452599	0.989243584	0.999867943
allS	0.011680006	0.773300266	0.987949129	0.999867943
ssuB	0.011335207	0.952450504	0.990504522	0.999867943
yddW	0.010847277	0.774791759	0.988829783	0.999867943
insK	0.010795954	1.177473689	0.992684504	0.999867943
ppsR	0.010747071	0.783788831	0.989059995	0.999867943
srlR	0.010500036	0.952754684	0.991206922	0.999867943
ybjG	0.010464992	0.807696546	0.989662427	0.999867943
ycaI	0.009650153	0.618613475	0.987553785	0.999867943
yfcF	0.009101138	0.857416345	0.991530928	0.999867943
glyA	0.008889342	0.85039952	0.991659756	0.999867943
hldE	0.007996308	0.730920633	0.991271278	0.999867943
idi	0.007757262	1.012946883	0.99388977	0.999867943
polA	0.007331434	0.61980261	0.990562315	0.999867943
deoC	0.006581146	1.048305117	0.994991	0.999867943
yjjK	0.006559844	0.639395892	0.991814294	0.999867943
ybjJ	0.006209972	0.774290728	0.993600869	0.999867943
yfeK	0.005739777	1.971497293	0.997677058	0.999867943
rarA	0.005339487	0.786295406	0.994581856	0.999867943
treA	0.005247296	0.688399822	0.993918221	0.999867943
phoA	0.005214231	0.767988954	0.994582836	0.999867943
ybeD	0.004887759	1.501781261	0.997403177	0.999867943
qmcA	0.004794239	0.812408487	0.995291498	0.999867943

hslR	0.004488123	0.974587009	0.996325632	0.999867943
hscC	0.003177581	0.743839275	0.996591556	0.999867943
yafW	0.002527222	0.889118515	0.997732104	0.999867943
ycbZ	0.002429431	0.695487144	0.997212886	0.999867943
yafN	0.00238261	0.803369457	0.99763366	0.999867943
ybaO	0.001972108	0.943045929	0.998331456	0.999867943
ynfD	0.0000687	1.303187656	0.999957923	0.999957923
yjhC	-0.000493947	1.172494382	0.999663868	0.999909425
efeO	-0.000785031	0.721037395	0.999131302	0.999867943
fkpA	-0.001052316	0.814330874	0.998968937	0.999867943
yaiW	-0.0012032	0.781735681	0.998771945	0.999867943
rapA	-0.001232099	0.627388489	0.998433073	0.999867943
dpiA	-0.001626496	0.922496853	0.998593214	0.999867943
cnu	-0.001716442	2.653006537	0.999483785	0.999909425
ycfD	-0.002289356	0.832189108	0.997805019	0.999867943
yjdL	-0.002439744	0.815878402	0.997614067	0.999867943
yghA	-0.002512293	0.807775251	0.997518473	0.999867943
mrp	-0.002983671	0.814863514	0.997078505	0.999867943
hypA	-0.003349854	1.078983247	0.99752286	0.999867943
wzxC	-0.003666896	0.881443366	0.996680727	0.999867943
pqiB	-0.004033986	0.660401134	0.995126243	0.999867943
yciQ	-0.00430218	0.641821139	0.994651754	0.999867943
dapB	-0.004691972	0.807874814	0.995366075	0.999867943
bfd	-0.004798988	2.406640414	0.998408971	0.999867943
yfbU	-0.005133006	0.875309949	0.99532106	0.999867943
ldcC	-0.006900414	0.664230588	0.99171126	0.999867943
yqgB	-0.007123611	3.057977593	0.998141316	0.999867943
lldP	-0.007226672	0.631938117	0.990875809	0.999867943
yiaN	-0.007389823	0.788049457	0.992518059	0.999867943
rpoB	-0.007423978	1.017057654	0.99417592	0.999867943
ygjJ	-0.007870334	0.760487187	0.991742786	0.999867943
pntB	-0.008670558	0.669060474	0.98966026	0.999867943
rplM	-0.008754511	1.610859539	0.995663772	0.999867943
hydN	-0.009238987	1.000090347	0.992629125	0.999867943
slyX	-0.009435	2.633132433	0.997141038	0.999867943
recX	-0.009702309	1.016007678	0.992380762	0.999867943
rcsD	-0.010386144	0.614098375	0.986506154	0.999867943
yigF	-0.010601792	2.426926419	0.99651453	0.999867943
ybiH	-0.010615355	0.966650125	0.991238135	0.999867943
nikA	-0.011157025	0.6384446	0.98605742	0.999867943
trkG	-0.011721421	0.849222319	0.98898752	0.999867943
ydaQ	-0.011852365	2.719728039	0.996522892	0.999867943
ytfQ	-0.011894959	0.720419669	0.986826605	0.999867943
frwB	-0.011938472	2.469548827	0.996142824	0.999867943
selA	-0.01273744	0.817667523	0.987571237	0.999867943
zapA	-0.012951107	1.385794897	0.992543386	0.999867943
aaeR	-0.013440628	0.722221382	0.985152128	0.999867943
ybfP	-0.013664561	2.006234743	0.994565612	0.999867943
tdcA	-0.015099594	1.033124859	0.988338966	0.999867943

deoB	-0.015109495	1.149060731	0.989508574	0.999867943
yfcV	-0.015177718	0.975845916	0.987590686	0.999867943
yihU	-0.016520847	0.917289195	0.985630468	0.999867943
leuO	-0.016827827	1.250141355	0.989260208	0.999867943
fliG	-0.017355576	1.182819212	0.988293013	0.999867943
artJ	-0.017553095	1.056702971	0.986746796	0.999867943
yraK	-0.017793136	1.158157767	0.987742335	0.999867943
ybjO	-0.017805104	1.01274689	0.985973113	0.999867943
ppdA	-0.017992019	1.007689075	0.985754742	0.999867943
feoB	-0.018193078	0.71240983	0.979626336	0.999867943
yqiJ	-0.018202304	1.125517309	0.987096863	0.999867943
ldhA	-0.018213942	0.892233687	0.98371322	0.999867943
mdtE	-0.018288586	0.727575089	0.979946202	0.999867943
bacA	-0.018929521	0.678984324	0.97775852	0.999867943
eno	-0.018996198	0.854750109	0.982269057	0.999867943
nagZ	-0.019277476	0.755394275	0.979640392	0.999867943
guaC	-0.019756287	0.793379994	0.980133596	0.999867943
csiE	-0.020863042	0.688391004	0.975822242	0.999867943
nemR	-0.021170748	1.058331116	0.984040263	0.999867943
yaaH	-0.021214023	0.76802543	0.977964025	0.999867943
yghD	-0.021932188	0.977529389	0.982099888	0.999867943
sibA	-0.022050626	2.942071787	0.994019966	0.999867943
potE	-0.022064411	0.746109196	0.976407894	0.999867943
ilvD	-0.022886468	0.708277766	0.974222567	0.999867943
ygdR	-0.022981431	2.443197019	0.992494974	0.999867943
htpX	-0.02360269	0.74187482	0.974619646	0.999867943
insA	-0.023838229	0.883861322	0.97848322	0.999867943
ubiG	-0.024025996	0.813970734	0.97645224	0.999867943
miaA	-0.024556099	0.839997323	0.976678328	0.999867943
ybeR	-0.025654877	1.082900676	0.981099176	0.999867943
dcyD	-0.025683227	0.841922312	0.975663939	0.999867943
rrsC	-0.026903112	1.59202991	0.986517492	0.999867943
kdgR	-0.027091475	0.795069078	0.972817849	0.999867943
opgC	-0.027247273	0.96488596	0.977471649	0.999867943
eutL	-0.027844362	1.193736939	0.981390731	0.999867943
yhdX	-0.027952552	0.689811352	0.967676949	0.999867943
rluC	-0.028028242	0.777912098	0.971258367	0.999867943
yfdP	-0.028744817	2.038356118	0.988748637	0.999867943
pspA	-0.028905959	0.813920798	0.971669515	0.999867943
ybjN	-0.029710517	1.096911407	0.97839145	0.999867943
yhfG	-0.029743255	3.194093757	0.992570243	0.999867943
envY	-0.030011178	1.088298516	0.978000135	0.999867943
yeiS	-0.030354079	1.07267966	0.977424929	0.999867943
insF1	-0.031202959	1.50297261	0.983436444	0.999867943
dtpB	-0.032017768	0.777782089	0.967163978	0.999867943
mutT	-0.032232212	0.980726491	0.973781727	0.999867943
yfaP	-0.032410508	0.822084703	0.968551724	0.999867943
glcA	-0.032534377	0.656383056	0.960468127	0.999867943
ansB	-0.032788147	1.010401963	0.974112713	0.999867943

gtrS	-0.033160397	1.242081443	0.978701053	0.999867943
rluB	-0.033399165	0.838333289	0.968220718	0.999867943
yfbM	-0.033648506	1.232752822	0.97822411	0.999867943
lacI	-0.034530707	1.075956898	0.974397869	0.999867943
mreB	-0.034588962	0.861095579	0.967958749	0.999867943
shiA	-0.035203643	0.692458375	0.959454099	0.999867943
prpR	-0.035257739	0.905646386	0.968945384	0.999867943
proB	-0.03574622	1.001114644	0.971516451	0.999867943
tomB	-0.036100822	1.381727899	0.979155801	0.999867943
gltK	-0.036248588	0.716055511	0.959626258	0.999867943
yjjY	-0.038805555	2.722684894	0.988628392	0.999867943
alsA	-0.038924312	1.008249176	0.969204641	0.999867943
garP	-0.039018918	0.632332963	0.950796734	0.999867943
xapA	-0.039937088	0.841795391	0.962160358	0.999867943
opgB	-0.040121546	0.636333624	0.9497258	0.999867943
metQ	-0.04020002	0.988656466	0.967565944	0.999867943
cyoB	-0.040289491	0.917349705	0.964968615	0.999867943
yghG	-0.040445267	1.164032576	0.972282423	0.999867943
yqjG	-0.041417241	1.022328432	0.967684416	0.999867943
pphB	-0.042128743	2.347365156	0.985680937	0.999867943
pflD	-0.042711714	0.639055806	0.946712538	0.999867943
yaiC	-0.042791258	0.722762015	0.952788681	0.999867943
yiaK	-0.04395476	0.918377058	0.961826748	0.999867943
ybgO	-0.044064841	0.89866196	0.960892333	0.999867943
ybhS	-0.044494661	0.809647614	0.956173845	0.999867943
truB	-0.045377361	0.902129587	0.959883109	0.999867943
leuE	-0.0454872	0.968322281	0.962532938	0.999867943
ycaQ	-0.04618299	0.754352636	0.951182395	0.999867943
araD	-0.046186685	0.959507148	0.961607981	0.999867943
accD	-0.04618695	0.917865165	0.959867408	0.999867943
rpiA	-0.04623786	0.806987376	0.954308705	0.999867943
puuC	-0.046740417	1.174944045	0.968267746	0.999867943
ynfO	-0.047003515	2.324386807	0.983866359	0.999867943
narQ	-0.047901355	0.63537159	0.939903551	0.999867943
mazF	-0.048807455	2.417964395	0.983895515	0.999867943
pntA	-0.048823777	0.819697666	0.952503566	0.999867943
bioF	-0.049321916	1.390750105	0.971709549	0.999867943
yigB	-0.050278525	1.011866952	0.960370326	0.999867943
cbl	-0.050403059	1.002113715	0.959885917	0.999867943
sodC	-0.050476253	1.010303988	0.960153107	0.999867943
cueO	-0.051487316	0.67797056	0.939464215	0.999867943
yphB	-0.051701166	0.879780461	0.953138501	0.999867943
cmk	-0.052023234	1.183185522	0.964929283	0.999867943
osmB	-0.0520427	2.748534	0.984893188	0.999867943
yehC	-0.052389452	0.882598394	0.952666806	0.999867943
pfkA	-0.052426283	0.819222828	0.948974094	0.999867943
waaG	-0.052568244	1.123235006	0.962672029	0.999867943
uidC	-0.05267915	0.771812094	0.945583557	0.999867943
lptF	-0.053712701	0.771727708	0.94451157	0.999867943

ydgK	-0.053803648	1.06848831	0.959839556	0.999867943
yphG	-0.05444145	0.773131438	0.943861912	0.999867943
gntU	-0.054753042	0.762523575	0.942757055	0.999867943
rihC	-0.055054831	0.857938349	0.948834004	0.999867943
dxr	-0.055816466	0.727777044	0.938866613	0.999867943
clcB	-0.056037527	0.884146572	0.949463627	0.999867943
sufB	-0.057121078	0.789085545	0.942292374	0.999867943
ycdU	-0.057283717	0.785795156	0.941886454	0.999867943
paaA	-0.057325148	0.845088492	0.945918329	0.999867943
ydeI	-0.05829051	1.133674654	0.958992985	0.999867943
rho	-0.058383925	0.953522903	0.951176274	0.999867943
lpoA	-0.058399054	0.710868005	0.934526038	0.999867943
nnr	-0.059879471	0.928413274	0.948574839	0.999867943
glyW	-0.06018959	2.144487273	0.977608615	0.999867943
ydjO	-0.06054631	1.178913894	0.959040488	0.999867943
ycfS	-0.060821841	0.858730346	0.943534936	0.999867943
malP	-0.060877786	0.635928006	0.923734517	0.999867943
ecpC	-0.061229014	0.81219291	0.93990658	0.999867943
dicC	-0.061308312	2.046713305	0.976103326	0.999867943
rpoE	-0.061411332	1.255678588	0.9609935	0.999867943
greB	-0.061483928	1.163414393	0.95785315	0.999867943
dnaC	-0.062309067	1.007746658	0.950698141	0.999867943
dapD	-0.062314498	0.801754232	0.938048643	0.999867943
ycfP	-0.063177336	0.985710623	0.948896026	0.999867943
proA	-0.063720548	0.893660267	0.943156706	0.999867943
grxC	-0.064962544	1.231166647	0.957919125	0.999867943
intA	-0.064964711	1.006462439	0.948534226	0.999867943
murB	-0.065929716	1.160116067	0.954680396	0.999867943
spr	-0.066401698	1.157630925	0.954258434	0.999867943
rfbA	-0.066723646	1.112734639	0.952184574	0.999867943
feaR	-0.068708395	0.917003783	0.940272749	0.999867943
insA	-0.069086906	1.245662808	0.955770432	0.999867943
ychA	-0.069484976	0.763209481	0.927458325	0.999867943
brnQ	-0.069933075	0.710732561	0.921618036	0.999867943
csgB	-0.070263166	2.493950356	0.977523819	0.999867943
yhjH	-0.070732793	0.799932168	0.929540093	0.999867943
proX	-0.070921438	0.698986589	0.919182744	0.999867943
ynfB	-0.070927641	0.928553463	0.939112731	0.999867943
yciG	-0.071129317	2.814673801	0.979838891	0.999867943
dmsA	-0.071595333	0.601375029	0.905233588	0.999867943
mutM	-0.071692148	0.792039822	0.927877309	0.999867943
codA	-0.071779623	0.810929531	0.929467171	0.999867943
rlmF	-0.071927952	0.812437635	0.929452656	0.999867943
yeaJ	-0.072473831	0.79620394	0.927473358	0.999867943
zapB	-0.072996653	1.302937246	0.95532214	0.999867943
btuB	-0.07338936	0.918289378	0.936301167	0.999867943
hyuA	-0.073660739	0.682540412	0.914058016	0.999867943
pflA	-0.073736363	0.829367568	0.929156031	0.999867943
clpP	-0.074542305	1.0199386	0.941738406	0.999867943

yaiE	-0.074789198	2.571925651	0.976801531	0.999867943
dsdA	-0.076133253	0.787449979	0.922977912	0.999867943
ykgJ	-0.076170092	0.793702832	0.923545969	0.999867943
fliK	-0.077229351	0.865484082	0.928897098	0.999867943
yacH	-0.077948945	0.651647714	0.90478558	0.999867943
yiaT	-0.078130068	2.32350471	0.973175424	0.999867943
gspC	-0.07815993	1.040384347	0.940114449	0.999867943
mltC	-0.078452681	1.08340748	0.942273317	0.999867943
aroG	-0.078518431	0.819227128	0.923644059	0.999867943
sxy	-0.07859221	2.322515259	0.973005325	0.999867943
yeeN	-0.079816407	1.289560429	0.950647027	0.999867943
ydiP	-0.07996849	0.815616901	0.921895265	0.999867943
lpxH	-0.080014497	0.810292872	0.921338654	0.999867943
tynA	-0.08064258	0.755524782	0.914997491	0.999867943
ftsN	-0.080905399	0.76192778	0.91543547	0.999867943
phnN	-0.080971465	1.664068269	0.961191258	0.999867943
yeiL	-0.081553163	1.134000862	0.942668507	0.999867943
qorB	-0.081905645	0.852187653	0.923431448	0.999867943
yejM	-0.082101879	0.682612827	0.904264586	0.999867943
ygbF	-0.082201292	2.013263812	0.96743153	0.999867943
yciZ	-0.082321302	2.662734375	0.975336468	0.999867943
gcvA	-0.082405133	0.86560541	0.92415643	0.999867943
psiE	-0.082504076	2.37228617	0.972256526	0.999867943
yqjC	-0.082530468	1.139138461	0.942243874	0.999867943
wcaJ	-0.083021621	0.908536761	0.927191062	0.999867943
flxA	-0.084463573	1.975978819	0.965904663	0.999867943
argE	-0.085025813	0.736745543	0.908122294	0.999867943
argH	-0.085361897	0.797472221	0.914756779	0.999867943
clcA	-0.086988212	0.78879645	0.91218758	0.999867943
sfmD	-0.087390371	0.743758552	0.906465186	0.999867943
fucA	-0.087517103	2.340650349	0.970173982	0.999867943
ptrA	-0.087946204	0.609971942	0.885357752	0.999867943
grxB	-0.0879568	0.961377125	0.927102915	0.999867943
uhpA	-0.088999476	1.18910271	0.940337312	0.999867943
yccT	-0.089155539	0.846554299	0.91612523	0.999867943
erfK	-0.089482038	0.81205114	0.91225662	0.999867943
nrdB	-0.090545863	0.816850248	0.911737225	0.999867943
yaiA	-0.090682117	2.540979121	0.971531248	0.999867943
galT	-0.091619898	1.087221034	0.932841924	0.999867943
ymgC	-0.092934223	2.75011218	0.97304231	0.999867943
ydaE	-0.093005331	2.647270103	0.971974052	0.999867943
atoE	-0.093650524	0.801588733	0.906993868	0.999867943
ynjH	-0.093797682	2.715844365	0.972448772	0.999867943
bglF	-0.093838389	0.935948956	0.920137788	0.999867943
iraP	-0.093946573	2.507742311	0.970116152	0.999867943
yjgN	-0.094221124	1.385594933	0.94578525	0.999867943
trpS	-0.094451625	0.865913262	0.913141047	0.999867943
yqfB	-0.094649265	2.421705429	0.968823638	0.999867943
mscK	-0.094836631	0.580696968	0.870270281	0.999867943

cusC	-0.095494777	0.833315073	0.908765158	0.999867943
arsB	-0.096331068	0.714739004	0.892787428	0.999867943
ygaC	-0.096940382	2.440652526	0.96831712	0.999867943
phnP	-0.098088696	1.081308827	0.927720694	0.999867943
nrfE	-0.098118336	0.823415117	0.905148413	0.999867943
wbbI	-0.098476397	1.786620462	0.956043809	0.999867943
cynS	-0.100001169	0.831189725	0.904236884	0.999867943
bluR	-0.100823458	1.131788032	0.929015678	0.999867943
scpA	-0.100974047	0.690540283	0.883744006	0.999867943
lpxT	-0.101316741	0.801579756	0.899418205	0.999867943
alaC	-0.103207971	0.756108845	0.891426951	0.999867943
yhfK	-0.103997046	0.621770881	0.867165921	0.999867943
yigE	-0.104207478	1.954295228	0.957475128	0.999867943
tdcC	-0.104555823	0.813349752	0.897714012	0.999867943
thrC	-0.10555948	0.704400894	0.880877353	0.999867943
amyA	-0.105820713	0.707420239	0.881090642	0.999867943
yjcE	-0.10589973	0.71254618	0.881852253	0.999867943
thrA	-0.106016755	0.568424855	0.852045065	0.999867943
ispG	-0.106071329	0.960809711	0.912093856	0.999867943
mreC	-0.106184571	0.821816962	0.897193793	0.999867943
rplL	-0.10675062	1.222883346	0.930437666	0.999867943
loiP	-0.107343556	0.954577825	0.91046555	0.999867943
dmsD	-0.108260624	0.943996445	0.908696154	0.999867943
dld	-0.10876724	0.693545234	0.875380487	0.999867943
yjbE	-0.109034847	2.227007406	0.960950971	0.999867943
smtA	-0.109275877	0.795820003	0.890783947	0.999867943
dcuS	-0.109775396	0.7488704	0.883457245	0.999867943
ydbL	-0.11010014	2.338901032	0.962454688	0.999867943
yihG	-0.110904626	1.023733769	0.913731178	0.999867943
trpA	-0.111065914	0.79194588	0.888466953	0.999867943
ybgQ	-0.111545365	0.62622885	0.858626877	0.999867943
ydaF	-0.112178845	2.869597107	0.968816885	0.999867943
bcsF	-0.113496549	2.806503684	0.967741903	0.999867943
ygiM	-0.113579249	0.74844778	0.879381697	0.999867943
thiF	-0.113617493	1.137866832	0.920462373	0.999867943
prmA	-0.113624399	0.745651027	0.878885014	0.999867943
wza	-0.113790549	0.805977511	0.887725154	0.999867943
yecR	-0.113994918	2.537248947	0.96416426	0.999867943
galP	-0.114241819	0.675802061	0.865760245	0.999867943
ybgA	-0.114452286	0.869499934	0.895276946	0.999867943
hemD	-0.115179483	0.836613869	0.890498536	0.999867943
ybhM	-0.116038614	1.321460512	0.930026988	0.999867943
yeaQ	-0.116889963	2.169309083	0.957027984	0.999867943
mazG	-0.117369375	0.790876456	0.882023798	0.999867943
yciI	-0.11737221	2.120694948	0.95586273	0.999867943
ycfH	-0.117372658	0.978971421	0.904567232	0.999867943
ybdR	-0.117766335	0.887964832	0.894489961	0.999867943
dxs	-0.118761799	0.70287409	0.865823413	0.999867943
ulaA	-0.118965708	0.794961728	0.88104107	0.999867943

ygfT	-0.119432811	0.628957112	0.849395148	0.999867943
atpH	-0.11987659	1.126728255	0.915270131	0.999867943
yfdC	-0.119994043	1.050413183	0.909051437	0.999867943
eamA	-0.120040019	0.915047652	0.8956294	0.999867943
ydiH	-0.121067971	1.087526691	0.911359316	0.999867943
fiu	-0.121361542	0.703263545	0.862990159	0.999867943
ybfD	-0.121536148	1.243029652	0.922111647	0.999867943
yeaI	-0.12214686	0.968132527	0.899599338	0.999867943
tam	-0.122566469	0.948877422	0.897223157	0.999867943
ushA	-0.122605327	0.627526952	0.845096582	0.999867943
wcaE	-0.122886193	0.882358719	0.889236711	0.999867943
ycgV	-0.123051521	0.620300755	0.842752485	0.999867943
serW	-0.123071038	2.123757573	0.953788723	0.999867943
eptB	-0.123290055	0.795098673	0.876771979	0.999867943
ycjX	-0.123428211	0.960783322	0.897780022	0.999867943
rutA	-0.123529084	0.801774309	0.877554821	0.999867943
yihD	-0.124114741	2.452896188	0.95964485	0.999867943
mrdB	-0.124692037	0.676453309	0.853752958	0.999867943
yeaW	-0.126034118	0.777579096	0.871238705	0.999867943
yciE	-0.126117046	1.046037551	0.904034435	0.999867943
gudD	-0.126613817	0.689152006	0.854229919	0.999867943
glpG	-0.12685891	0.789816337	0.872394088	0.999867943
pldB	-0.126933806	0.868213534	0.883762608	0.999867943
dhaK	-0.126949406	0.788911367	0.872158617	0.999867943
pbpG	-0.127622689	0.797484182	0.872856162	0.999867943
mtn	-0.127955077	0.857779057	0.88141933	0.999867943
insD1	-0.127985611	1.411331796	0.92774346	0.999867943
ompX	-0.128030419	1.26553237	0.9194177	0.999867943
yqjA	-0.128299147	0.822463668	0.876037991	0.999867943
dusA	-0.128311286	0.79451367	0.87170226	0.999867943
ykgF	-0.128367739	0.631135424	0.838828847	0.999867943
prmB	-0.128716896	0.852404252	0.879972149	0.999867943
yfjW	-0.129760708	1.044917834	0.901170627	0.999867943
wbbH	-0.130790721	1.176058778	0.911448996	0.999867943
stpA	-0.131324295	2.435117823	0.956991461	0.999867943
glyV	-0.131840511	2.14756423	0.951048044	0.999867943
rtcR	-0.131849154	0.832199468	0.874114385	0.999867943
proS	-0.131931218	0.759359641	0.862069723	0.999867943
ppx	-0.132067548	0.699750803	0.850300429	0.999867943
insD1	-0.132273891	1.467681669	0.928188381	0.999867943
ugpE	-0.132562281	0.830283057	0.873149587	0.999867943
ubiC	-0.132673706	1.088398824	0.902979751	0.999867943
yhaV	-0.133006244	1.8568058	0.942894974	0.999867943
yhaB	-0.133342406	2.286109289	0.953487984	0.999867943
yheV	-0.133507271	2.738232485	0.961113163	0.999867943
sapF	-0.133632232	0.852340402	0.875416106	0.999867943
yqeF	-0.133989034	0.814948744	0.869405193	0.999867943
yhdV	-0.134576056	2.663920656	0.959709572	0.999867943
hslV	-0.13464071	0.922442601	0.88395212	0.999867943

cysK	-0.134709964	0.763246278	0.859904241	0.999867943
xanQ	-0.135477603	0.976609171	0.889669488	0.999867943
hmp	-0.13552177	0.733177046	0.853353116	0.999867943
hofM	-0.135631086	1.099216295	0.901799149	0.999867943
recN	-0.135675852	0.703508189	0.84707166	0.999867943
ydiM	-0.135705748	1.123287979	0.903840593	0.999867943
matP	-0.136388558	2.383699969	0.954372204	0.999867943
yjdM	-0.136615514	2.430271617	0.955171255	0.999867943
rcsC	-0.136622827	0.636382948	0.83001175	0.999867943
yeiQ	-0.136781783	0.645384783	0.832155135	0.999867943
panE	-0.137121632	0.775152922	0.859589897	0.999867943
yagN	-0.137230402	0.983613907	0.889041993	0.999867943
yjdA	-0.137815513	0.757165501	0.855570919	0.999867943
oppA	-0.138523735	0.777310049	0.858558753	0.999867943
holD	-0.138830779	1.494471302	0.925985985	0.999867943
glk	-0.13973513	0.659404123	0.8321763	0.999867943
afuC	-0.139857051	0.866670776	0.871799823	0.999867943
leuQ	-0.139903935	2.861339583	0.961003325	0.999867943
dacD	-0.139927078	0.799071654	0.860991588	0.999867943
cytR	-0.140143867	0.86982956	0.87200167	0.999867943
eptC	-0.140705475	1.043077577	0.892695252	0.999867943
prlF	-0.140884481	2.524599016	0.955497394	0.999867943
thiE	-0.141908891	1.469711955	0.923079333	0.999867943
rhaM	-0.14242029	2.437390818	0.953404961	0.999867943
murC	-0.142628311	0.832642938	0.863991115	0.999867943
yqcC	-0.142741929	1.303120197	0.912775327	0.999867943
exuR	-0.143211282	0.879720881	0.870682422	0.999867943
ydaT	-0.143353158	1.151315574	0.900909519	0.999867943
barA	-0.143637657	0.667188237	0.829542743	0.999867943
yfdT	-0.143851712	2.538199717	0.954804327	0.999867943
ybiV	-0.143854051	0.992701054	0.884780546	0.999867943
yhiN	-0.143902753	0.73646927	0.845083369	0.999867943
ybfC	-0.144095645	1.950417886	0.941106371	0.999867943
yjaZ	-0.144276452	1.965881	0.941495595	0.999867943
insB1	-0.144656725	0.977184245	0.882315748	0.999867943
pnuC	-0.145267715	0.961577212	0.879918655	0.999867943
entF	-0.145363552	0.817226394	0.858821714	0.999867943
eco	-0.145531789	1.063926294	0.891198786	0.999867943
ydcA	-0.145758213	2.791501619	0.958357394	0.999867943
yhaI	-0.146345218	1.976383166	0.940972998	0.999867943
lptC	-0.147212334	1.04924257	0.888420252	0.999867943
yhcN	-0.147442554	1.870958524	0.937187018	0.999867943
mutY	-0.147724504	0.791869368	0.852012212	0.999867943
yrbL	-0.147996638	1.13565956	0.89631501	0.999867943
ilvM	-0.150194972	2.05651274	0.941779208	0.999867943
alsB	-0.150540709	0.943056342	0.873172024	0.999867943
cusR	-0.150572118	0.884857788	0.864880116	0.999867943
cydC	-0.150778244	0.699880177	0.829428677	0.999867943
yigP	-0.15150267	1.05568697	0.885886641	0.999867943

yejH	-0.151505656	0.725247902	0.834524831	0.999867943
erpA	-0.15166175	1.020022741	0.881802454	0.999867943
sibC	-0.152043201	3.071260354	0.960516734	0.999867943
accA	-0.152076533	0.83280185	0.855105369	0.999867943
gshA	-0.153381825	0.717030167	0.830615173	0.999867943
selU	-0.155148414	0.77822872	0.841980408	0.999867943
hemX	-0.155314331	0.71665753	0.828426222	0.999867943
yehB	-0.155331853	0.825055916	0.850666355	0.999867943
ddpD	-0.155511143	0.856612977	0.855942252	0.999867943
leuP	-0.156356678	2.78781371	0.95527348	0.999867943
nemA	-0.156483416	0.769433878	0.838842141	0.999867943
accB	-0.157146448	0.993083847	0.874267003	0.999867943
argI	-0.157468874	1.035797747	0.879165907	0.999867943
elfC	-0.157527396	0.81188446	0.846154847	0.999867943
araA	-0.157905158	0.833869595	0.849807279	0.999867943
ycbF	-0.158345972	0.929999954	0.864802133	0.999867943
yecH	-0.158484135	2.600537383	0.951404731	0.999867943
mdtN	-0.158835559	0.899884866	0.859896015	0.999867943
cmtA	-0.159396956	0.836701451	0.848912352	0.999867943
minE	-0.159522047	2.663693244	0.9522452	0.999867943
yjhF	-0.159998558	0.959343288	0.867543743	0.999867943
ebgR	-0.161190529	1.111780291	0.88472338	0.999867943
uup	-0.161459485	0.723613413	0.823434735	0.999867943
ybbN	-0.16289933	0.926047106	0.86036602	0.999867943
acrA	-0.163084858	0.883934277	0.853622129	0.999867943
yidP	-0.163334518	1.122667947	0.884325728	0.999867943
artI	-0.163903433	0.837429922	0.844827788	0.999867943
epd	-0.16422234	0.849183785	0.846654757	0.999867943
mlrA	-0.164711981	1.017960242	0.871458701	0.999867943
plsB	-0.164738952	0.711909737	0.817000749	0.999867943
eutK	-0.165089684	1.427816932	0.907950664	0.999867943
alx	-0.165156397	0.838309063	0.843818658	0.999867943
nagA	-0.165278025	0.688880966	0.810390358	0.999867943
ldrD	-0.165735677	3.632757728	0.963611094	0.999867943
ssuA	-0.166404418	0.787652745	0.83267954	0.999867943
nanC	-0.166580208	1.848974142	0.928213062	0.999867943
galR	-0.167732167	0.732698914	0.818928197	0.999867943
yahD	-0.167791561	0.918269121	0.855013084	0.999867943
dsdX	-0.167810404	0.935002908	0.85756411	0.999867943
eamB	-0.168048881	0.901526929	0.852127386	0.999867943
yebV	-0.168333856	2.563903382	0.947652258	0.999867943
thrW	-0.168424421	2.694147234	0.950152774	0.999867943
gudP	-0.169111047	0.704897377	0.810400999	0.999867943
nikE	-0.169552739	1.049947138	0.871709905	0.999867943
asnB	-0.169675423	0.787500798	0.829408199	0.999867943
torZ	-0.169684601	0.71781482	0.81312967	0.999867943
yahI	-0.170395267	1.075166272	0.874076442	0.999867943
ypeC	-0.170427317	1.173785629	0.884557242	0.999867943
phnF	-0.171210044	1.511162813	0.909795184	0.999867943

mutS	-0.171317995	0.678110279	0.800546174	0.999867943
ligA	-0.171841852	0.72077578	0.811561285	0.999867943
ddpA	-0.172318425	0.768106396	0.822491299	0.999867943
rcsA	-0.172583905	1.9730349	0.930296906	0.999867943
yebY	-0.172633562	1.928987223	0.928689	0.999867943
djlB	-0.173267898	0.933471523	0.852745361	0.999867943
ybaA	-0.17367989	2.497629595	0.944561474	0.999867943
yeiG	-0.174008728	0.848428226	0.837497589	0.999867943
citC	-0.174119709	0.826985895	0.833240481	0.999867943
gspD	-0.174218003	0.830980689	0.833938127	0.999867943
ydiK	-0.174474005	0.762841192	0.819089639	0.999867943
ybhL	-0.174718297	1.004529627	0.861920116	0.999867943
yeaR	-0.175159087	2.474023897	0.943557513	0.999867943
torT	-0.176034857	0.747080819	0.81371958	0.999867943
ttdT	-0.176658364	0.77725196	0.820201484	0.999867943
ygbE	-0.17767746	2.338464707	0.9394347	0.999867943
ghrB	-0.177888558	0.770097676	0.817318842	0.999867943
rutC	-0.178503052	1.129928494	0.874474686	0.999867943
mokB	-0.178777415	2.623328475	0.945666963	0.999867943
ruvB	-0.179007031	0.811324345	0.825376182	0.999867943
ygiZ	-0.179225415	1.996259497	0.928461549	0.999867943
mscL	-0.179831352	1.20815399	0.881673544	0.999867943
ydcX	-0.180433135	2.595831501	0.944584627	0.999867943
ydbH	-0.180558456	0.678488706	0.79014788	0.999867943
yfbP	-0.18164968	1.044538511	0.861940717	0.999867943
yidK	-0.182118093	0.701634024	0.795201033	0.999867943
mocA	-0.182345538	1.97397113	0.92640012	0.999867943
yhcH	-0.182576475	2.387063752	0.939032613	0.999867943
purB	-0.183023969	0.671185973	0.785093509	0.999867943
ycaM	-0.184394912	0.881575093	0.834319206	0.999867943
potG	-0.184731932	0.864943566	0.830877045	0.999867943
atpE	-0.185479468	1.430022146	0.896800687	0.999867943
recT	-0.186103874	1.09054665	0.864497474	0.999867943
yebT	-0.186504915	0.634171625	0.768687467	0.999867943
tatC	-0.186626589	1.072575667	0.861866617	0.999867943
ychO	-0.186760231	0.701583921	0.79008673	0.999867943
metY	-0.187511454	2.681438398	0.94424983	0.999867943
ddpB	-0.187771366	0.876335111	0.830337364	0.999867943
mgsA	-0.188149555	1.10188399	0.864418302	0.999867943
mngA	-0.188302221	0.796177257	0.813038568	0.999867943
ulaR	-0.188463129	0.979301292	0.847392453	0.999867943
hyfE	-0.188922866	0.945205803	0.841578462	0.999867943
ydiO	-0.189853578	0.827747483	0.818587794	0.999867943
yihV	-0.190362414	0.844146505	0.821583528	0.999867943
sucB	-0.191078494	1.161557089	0.869335952	0.999867943
rseA	-0.191586718	1.306618588	0.883425744	0.999867943
aaeX	-0.191742493	2.428018529	0.937055872	0.999867943
hybC	-0.191920678	0.890620622	0.829384572	0.999867943
talB	-0.19251383	1.026180328	0.851188392	0.999867943

glnA	-0.192592727	0.950919283	0.839499905	0.999867943
ralR	-0.192637356	2.71942353	0.94352703	0.999867943
yecF	-0.192967074	2.635937264	0.941641996	0.999867943
rhaT	-0.193110661	0.896217666	0.829398602	0.999867943
yaeH	-0.193759835	1.21743542	0.873547468	0.999867943
aroD	-0.193948624	0.859510546	0.821473586	0.999867943
ptsA	-0.194459788	0.73689643	0.791864484	0.999867943
hemA	-0.194643748	0.884748435	0.825871954	0.999867943
yqiA	-0.194906341	0.903805453	0.829259978	0.999867943
ftsH	-0.195239781	0.973288974	0.841012955	0.999867943
yeeX	-0.19556502	1.379136731	0.887236019	0.999867943
tauD	-0.195892596	0.869085313	0.821667425	0.999867943
ybaB	-0.195953094	1.0664894	0.854220126	0.999867943
bcsA	-0.196175878	0.617687115	0.750790277	0.999867943
rhsA	-0.196451247	0.916104267	0.830202379	0.999867943
yehP	-0.197092356	0.799357238	0.805246019	0.999867943
tauA	-0.197358306	0.765291712	0.7964946	0.999867943
flhB	-0.198219031	0.869223764	0.819614032	0.999867943
relB	-0.198360726	2.854964575	0.944608169	0.999867943
ynjE	-0.198537425	0.776868585	0.798289724	0.999867943
yahK	-0.198619469	0.603680792	0.74214506	0.999867943
yjaG	-0.199056719	1.165097583	0.86434185	0.999867943
apbE	-0.199316024	0.828953261	0.809986832	0.999867943
parC	-0.199376366	0.664291286	0.764074986	0.999867943
hemN	-0.199682648	0.903594613	0.825102568	0.999867943
yibN	-0.20000303	1.264874743	0.874361596	0.999867943
amn	-0.201137111	0.766297462	0.792952053	0.999867943
sfsB	-0.201179704	1.91149378	0.916179519	0.999867943
rplC	-0.201330364	1.481881622	0.89193093	0.999867943
ispB	-0.2019625	0.851259554	0.812461782	0.999867943
yciT	-0.202202216	1.104937986	0.854799038	0.999867943
recE	-0.202270876	0.742197968	0.785214875	0.999867943
ydcI	-0.202909492	0.922990552	0.8259964	0.999867943
katG	-0.203310052	0.586577955	0.728889114	0.999867943
dnaN	-0.204481687	0.800816926	0.798459454	0.999867943
sbcB	-0.20531919	0.716151235	0.774343502	0.999867943
fdoI	-0.205583019	0.845342011	0.807854277	0.999867943
caiC	-0.205971972	0.727675882	0.777135018	0.999867943
yhcB	-0.206240126	1.212820946	0.864970864	0.999867943
fruA	-0.206588947	0.750452577	0.783096664	0.999867943
narZ	-0.206926614	0.806213365	0.797437526	0.999867943
dacB	-0.206985491	0.675241224	0.759196859	0.999867943
glnU	-0.207104781	2.518246744	0.934454549	0.999867943
ybjD	-0.207154945	0.660029166	0.753629453	0.999867943
recG	-0.207932426	0.805890841	0.796394852	0.999867943
cbpA	-0.208347856	0.772039373	0.787262669	0.999867943
yehD	-0.20836722	1.857991071	0.910707238	0.999867943
srlD	-0.208631271	0.871956857	0.810897868	0.999867943
mppA	-0.208729952	0.771505728	0.786738076	0.999867943

allA	-0.209052862	1.988262216	0.916261937	0.999867943
nrdG	-0.209597079	2.385795013	0.929994236	0.999867943
eutR	-0.209993002	0.904326428	0.816375459	0.999867943
pepB	-0.21024034	0.862938911	0.807515118	0.999867943
ydjF	-0.2103236	1.251031304	0.866488739	0.999867943
yfdS	-0.210434097	2.408546	0.930377602	0.999867943
uspD	-0.211065942	2.360594612	0.928754333	0.999867943
argG	-0.211073554	1.126889716	0.851420477	0.999867943
ybiR	-0.211211259	0.723095498	0.770215385	0.999867943
yjcO	-0.211350385	0.991077135	0.831129463	0.999867943
yhbE	-0.211487445	0.772997647	0.784396718	0.999867943
rsxG	-0.211643873	1.060688262	0.84184468	0.999867943
gluQ	-0.212655647	0.890344288	0.811224586	0.999867943
trpC	-0.214107997	0.785267323	0.785117514	0.999867943
ynaJ	-0.214205739	2.457202561	0.9305327	0.999867943
norW	-0.214751507	0.831331794	0.796158209	0.999867943
chbR	-0.21531476	0.883811798	0.807524673	0.999867943
znuB	-0.215593799	0.783173791	0.783099444	0.999867943
ivy	-0.215939727	2.398894914	0.928274234	0.999867943
frc	-0.216112013	1.078189858	0.841136741	0.999867943
rarD	-0.216441803	1.06447079	0.838874937	0.999867943
fruB	-0.217299717	1.015713942	0.830595477	0.999867943
frlB	-0.217604314	0.764319965	0.775871565	0.999867943
nudF	-0.21777835	0.990906241	0.826044873	0.999867943
yjaA	-0.217999337	2.399245686	0.927602551	0.999867943
flgG	-0.218418525	0.817731297	0.789389818	0.999867943
fre	-0.218429222	0.82081972	0.790153662	0.999867943
dsbB	-0.219901327	0.981144268	0.822658162	0.999867943
pepE	-0.220067749	0.860335929	0.798110823	0.999867943
tig	-0.220588562	1.056560549	0.834620095	0.999867943
caiB	-0.220788987	0.73931315	0.765214217	0.999867943
cysE	-0.221046068	0.767115226	0.773230109	0.999867943
dgoD	-0.221052637	0.823362107	0.788333253	0.999867943
yijO	-0.221161311	0.82081631	0.787590579	0.999867943
ytcA	-0.221320447	2.424669469	0.927271218	0.999867943
yodB	-0.221387194	2.30688734	0.923546046	0.999867943
yqhA	-0.222038908	0.951333646	0.815452743	0.999867943
yadE	-0.222239779	1.087040058	0.838005859	0.999867943
malX	-0.222265465	0.741175874	0.764266998	0.999867943
cheY	-0.22266647	2.472082948	0.928229673	0.999867943
ylaC	-0.223022884	1.253950244	0.858835873	0.999867943
insA	-0.2230422	2.365803997	0.924888685	0.999867943
thiI	-0.223215267	0.720851415	0.756823303	0.999867943
creA	-0.22424664	2.364838781	0.92445356	0.999867943
rlmA	-0.22464621	0.912420994	0.805520491	0.999867943
yafK	-0.226145212	0.701369596	0.747124137	0.999867943
ykgH	-0.227387219	1.012455569	0.822298381	0.999867943
nagC	-0.227740701	0.740617858	0.758462094	0.999867943
glsA	-0.227764546	1.038483762	0.826397568	0.999867943

lhr	-0.227978746	0.904685267	0.801042722	0.999867943
casC	-0.227994023	1.296149266	0.860371677	0.999867943
yddB	-0.229248499	0.922950853	0.803835405	0.999867943
era	-0.230012191	0.82113293	0.779388772	0.999867943
ybjK	-0.231024482	1.024609718	0.821609312	0.999867943
argZ	-0.231146033	2.457425274	0.925061297	0.999867943
mdtC	-0.231421023	0.984156858	0.814095042	0.999867943
ydiE	-0.231522377	3.193767627	0.942210428	0.999867943
serB	-0.231704424	0.921136754	0.801395227	0.999867943
fhuD	-0.232394417	0.901501248	0.796572056	0.999867943
sucC	-0.232511662	1.133835406	0.837520167	0.999867943
wecB	-0.232533702	0.805688698	0.77287615	0.999867943
ypdA	-0.232536226	0.756855477	0.758660752	0.999867943
yaaX	-0.233074484	2.05345209	0.909631201	0.999867943
ydiZ	-0.233278888	2.000861181	0.907185563	0.999867943
plsC	-0.233375054	1.237013004	0.85035919	0.999867943
lsrR	-0.233710439	0.846136374	0.782387584	0.999867943
eptA	-0.234013309	1.02615931	0.819609129	0.999867943
artP	-0.234145213	0.784254328	0.765277502	0.999867943
proW	-0.234497548	1.058644981	0.824697485	0.999867943
araB	-0.235233065	0.885339424	0.790471673	0.999867943
intS	-0.235787605	1.574224875	0.880938087	0.999867943
gspO	-0.235999151	0.903595088	0.793955248	0.999867943
yfhR	-0.236339414	1.031109771	0.818706648	0.999867943
yiaM	-0.236523982	2.360602223	0.920188358	0.999867943
yeaY	-0.236667043	0.911510989	0.795139507	0.999867943
xdhB	-0.237284252	0.808469736	0.769141526	0.999867943
intR	-0.237612961	0.834762598	0.775914332	0.999867943
yefM	-0.23785563	2.564735549	0.926109482	0.999867943
yegD	-0.237877689	0.979196	0.808058355	0.999867943
cedA	-0.238055332	2.481075887	0.923561532	0.999867943
oppB	-0.238443194	0.861578867	0.781970968	0.999867943
guaD	-0.238504001	1.001493186	0.811765996	0.999867943
leuV	-0.239232662	2.848599113	0.933070293	0.999867943
hiuH	-0.239453442	2.019656897	0.905622811	0.999867943
ybiU	-0.240179207	0.764837968	0.753501094	0.999867943
serC	-0.240330858	0.805048904	0.765299054	0.999867943
yliF	-0.240902984	1.14500769	0.833360014	0.999867943
etk	-0.241497933	0.727988251	0.740090665	0.999867943
cusA	-0.242349414	0.770881412	0.753232724	0.999867943
metI	-0.242754303	0.96396308	0.801172885	0.999867943
hisS	-0.244030084	0.866083799	0.778125496	0.999867943
ykgM	-0.244818481	2.365186908	0.91755889	0.999867943
ykfM	-0.245449494	2.273328243	0.914020102	0.999867943
yqhH	-0.245671882	2.6486859	0.926100295	0.999867943
yicR	-0.245803869	1.099928368	0.823167694	0.999867943
narI	-0.246820657	0.968471295	0.798834372	0.999867943
oxc	-0.247082985	0.813152302	0.761235933	0.999867943
yghR	-0.247566624	0.796811616	0.756031317	0.999867943

pgaC	-0.247579333	0.882354382	0.779025306	0.999867943
ymdA	-0.249271056	2.384054196	0.916726837	0.999867943
xdhC	-0.249392564	0.918086884	0.785895956	0.999867943
idnR	-0.249731511	1.844134627	0.892280327	0.999867943
yjjQ	-0.249923841	2.316158749	0.914071486	0.999867943
idnT	-0.250058753	0.977330288	0.798059712	0.999867943
sbp	-0.250241193	1.193909878	0.833981395	0.999867943
modB	-0.250749936	0.894995404	0.779347876	0.999867943
ydhY	-0.250821704	1.151083448	0.827506607	0.999867943
mdtO	-0.251028785	0.664714188	0.705691206	0.999867943
nrdR	-0.251546101	1.032917563	0.807595083	0.999867943
tdk	-0.251809836	2.359740674	0.915018407	0.999867943
mtfA	-0.252042654	0.835832079	0.762997418	0.999867943
wzc	-0.252616691	0.6819037	0.711041254	0.999867943
ynbB	-0.252932677	0.896258334	0.777782822	0.999867943
ycbV	-0.252951874	2.40305516	0.916167426	0.999867943
yfaL	-0.25371787	0.647287185	0.695079567	0.999867943
ydaM	-0.253788002	1.025955346	0.804623829	0.999867943
patD	-0.25640986	0.835426208	0.758903493	0.999867943
amtB	-0.257512469	0.775384699	0.739806729	0.999867943
ynaI	-0.257979335	0.915309484	0.778059069	0.999867943
pspG	-0.258452346	2.047502247	0.899551358	0.999867943
yjgB	-0.259780125	0.955728871	0.785765332	0.999867943
ycgL	-0.260118149	2.457172972	0.915692844	0.999867943
fdhE	-0.261611922	0.855573902	0.759777021	0.999867943
yqeB	-0.261798248	0.753344454	0.7282047	0.999867943
osmF	-0.262289448	0.929998783	0.777918948	0.999867943
alaE	-0.262527952	1.990233637	0.895056976	0.999867943
yahA	-0.262927269	0.62350052	0.673247401	0.999867943
ppiC	-0.262988264	2.00400881	0.895592501	0.999867943
sugE	-0.263657568	2.626122047	0.920028307	0.999867943
ydjL	-0.263724505	0.850324029	0.756450015	0.999867943
argC	-0.264035273	0.761068063	0.728645951	0.999867943
ybhR	-0.26413002	0.779614721	0.734763733	0.999867943
typA	-0.264482938	0.949175164	0.780517255	0.999867943
yieP	-0.266833926	0.996471695	0.788869643	0.999867943
norV	-0.267079234	0.750472591	0.721929389	0.999867943
ygeW	-0.267312643	0.960858259	0.780857336	0.999867943
rlmI	-0.267418748	0.766928185	0.727323463	0.999867943
yddA	-0.268344378	1.020205818	0.792527727	0.999867943
uraA	-0.26844444	0.761159393	0.724329591	0.999867943
nirC	-0.270109916	0.823482458	0.74290462	0.999867943
relE	-0.270383988	2.543665177	0.915346709	0.999867943
sseB	-0.270617213	0.788007012	0.731282664	0.999867943
solA	-0.270719045	0.783311021	0.729636863	0.999867943
yhfT	-0.270969308	0.806600244	0.736916092	0.999867943
yafJ	-0.271210646	0.590182709	0.645848766	0.999867943
yifL	-0.271391538	2.526203083	0.914447372	0.999867943
tyrT	-0.271494778	1.995605159	0.891784648	0.999867943

ybeU	-0.271570839	1.954121135	0.889471177	0.999867943
elfG	-0.271836012	1.07522743	0.800409459	0.999867943
gltT	-0.271854551	2.958095309	0.926775989	0.999867943
frvX	-0.272587461	0.861399611	0.751663126	0.999867943
sieB	-0.272947542	1.97212041	0.889921862	0.999867943
ybdJ	-0.27310809	2.533864437	0.914167647	0.999867943
queD	-0.273418984	2.33715913	0.906869762	0.999867943
luxS	-0.273602821	1.111960928	0.805640162	0.999867943
yhdN	-0.273972418	2.002123402	0.891156534	0.999867943
folC	-0.274880558	0.972655644	0.777477117	0.999867943
yfbS	-0.275178052	0.613852745	0.653950964	0.999867943
pdxH	-0.275558056	0.805322084	0.732222028	0.999867943
ynfE	-0.275674029	0.701688209	0.694413624	0.999867943
ycaD	-0.275916035	0.806178525	0.732161421	0.999867943
yjdN	-0.276810518	0.8904703	0.755907976	0.999867943
nanM	-0.27706116	1.940768322	0.886480919	0.999867943
rsmA	-0.277830578	0.864339758	0.747879424	0.999867943
purU	-0.277919583	0.936146754	0.766561115	0.999867943
fieF	-0.278247169	0.841076965	0.740778578	0.999867943
truC	-0.278461617	0.905267045	0.758385481	0.999867943
gatC	-0.278870927	0.772038557	0.717939679	0.999867943
ygeV	-0.279171269	0.880838976	0.751290712	0.999867943
yejK	-0.27998735	0.764763421	0.714283177	0.999867943
cydD	-0.280356622	0.649817798	0.66614933	0.999867943
ytfE	-0.280592907	0.947968592	0.767234751	0.999867943
yaaY	-0.281113046	2.507366114	0.910732321	0.999867943
sulA	-0.281460119	1.982743333	0.887115634	0.999867943
yphC	-0.281777112	0.850315681	0.740357912	0.999867943
maeB	-0.282172832	0.698251323	0.68612921	0.999867943
cmoA	-0.282385666	0.972322075	0.771491885	0.999867943
ygcS	-0.28282919	0.773933261	0.714780075	0.999867943
dcp	-0.282864391	0.727889485	0.697565423	0.999867943
yadN	-0.284315439	2.393014136	0.905425413	0.999867943
phoP	-0.28451277	0.945635336	0.763514058	0.999867943
fadI	-0.284621311	0.752708049	0.705334205	0.999867943
ysgA	-0.284879055	0.87377042	0.744398466	0.999867943
grxA	-0.285152418	2.597733607	0.912592028	0.999867943
lysS	-0.285183183	0.795720988	0.720047018	0.999867943
yhhN	-0.286458824	0.88987472	0.747521513	0.999867943
hemB	-0.286697404	0.942508473	0.760986576	0.999867943
speC	-0.287933378	0.730470293	0.693451589	0.999867943
rsmE	-0.288871499	0.838222957	0.730377284	0.999867943
ydhU	-0.289634191	0.981184306	0.767849989	0.999867943
rpiB	-0.289656983	2.35188244	0.901980689	0.999867943
acnB	-0.290136142	1.107276888	0.793300835	0.999867943
garK	-0.290140737	0.731277316	0.691545309	0.999867943
ttdR	-0.290717701	1.068635317	0.785586839	0.999867943
lrhA	-0.291041413	1.038666942	0.779318944	0.999867943
ppiD	-0.291479542	0.709965754	0.681399329	0.999867943

helD	-0.291506824	0.711513967	0.682026492	0.999867943
gpsA	-0.291815346	0.892488719	0.743692008	0.999867943
yraH	-0.293047431	1.953239078	0.880739746	0.999867943
alr	-0.293439409	0.720318717	0.683733022	0.999867943
sohB	-0.293500056	0.764924244	0.701202147	0.999867943
nudK	-0.29359304	1.98105725	0.88218479	0.999867943
idnD	-0.293937875	1.939946113	0.879566657	0.999867943
dinB	-0.294172478	0.943744884	0.755262731	0.999867943
dapF	-0.29446514	0.794333853	0.710855757	0.999867943
yihT	-0.295231515	0.913596858	0.746579415	0.999867943
tamA	-0.295816197	0.684900594	0.665805998	0.999867943
ycjM	-0.295971302	0.938976151	0.752604939	0.999867943
yhfU	-0.296019935	2.412971484	0.90236162	0.999867943
srlE	-0.296475938	0.835213534	0.722611896	0.999867943
ymgA	-0.297081728	2.585622126	0.908526302	0.999867943
metR	-0.297391068	0.875381082	0.734061715	0.999867943
hyfC	-0.298600529	0.80944564	0.712205917	0.999867943
yhjG	-0.299105615	0.758838446	0.693461113	0.999867943
yahE	-0.299411858	0.699846707	0.668778595	0.999867943
rfbD	-0.299584739	1.125145196	0.790036527	0.999867943
ydhV	-0.300347336	0.889086593	0.735502141	0.999867943
yadI	-0.300736159	2.393053396	0.899992774	0.999867943
otsA	-0.30176617	0.791353231	0.702958919	0.999867943
yfcC	-0.302312682	0.747524013	0.685904859	0.999867943
ydeS	-0.302339839	1.966637118	0.877819135	0.999867943
cpxA	-0.302455115	0.685434426	0.659024442	0.999867943
rpsN	-0.303256094	1.611549721	0.850738144	0.999867943
yjgZ	-0.303447582	2.062848639	0.883052115	0.999867943
melA	-0.304188775	1.062267986	0.774604056	0.999867943
kefC	-0.305626911	0.720308442	0.671346999	0.999867943
ilvN	-0.305711458	2.527479921	0.903726639	0.999867943
hyfA	-0.30727207	0.970568093	0.751554789	0.999867943
gspE	-0.307430028	0.766746164	0.688453908	0.999867943
kdsD	-0.307624971	0.817469293	0.706683626	0.999867943
cyaY	-0.307825389	2.454897621	0.900212942	0.999867943
ebgA	-0.307843433	0.68616029	0.653686662	0.999867943
yoaA	-0.308067797	0.82326452	0.708253524	0.999867943
ykgE	-0.308882095	0.665114375	0.642357981	0.999867943
paoA	-0.310195244	0.739490344	0.674871461	0.999867943
holE	-0.310328228	2.506618804	0.901470846	0.999867943
yegL	-0.310769774	0.894019072	0.72813326	0.999867943
yedZ	-0.310795552	0.94534994	0.742335256	0.999867943
cysI	-0.312091975	0.851508616	0.713979687	0.999867943
yfcR	-0.312537789	2.378030411	0.895437402	0.999867943
setB	-0.312587466	0.783270085	0.68983422	0.999867943
fis	-0.313553662	1.353478959	0.816798241	0.999867943
mdtH	-0.313794541	0.767035636	0.682466025	0.999867943
msrB	-0.314291238	1.344515022	0.815172802	0.999867943
fucO	-0.314696057	0.875405564	0.719231539	0.999867943

csgD	-0.315938227	2.369334784	0.893920781	0.999867943
ycgZ	-0.316410891	2.489126452	0.898847604	0.999867943
serT	-0.317879304	2.70571166	0.90647609	0.999867943
rspA	-0.317885996	1.042039998	0.760319524	0.999867943
nusG	-0.318086127	1.130919618	0.778508495	0.999867943
tsf	-0.319238532	1.12533079	0.776652426	0.999867943
speG	-0.319842468	1.052393114	0.761189454	0.999867943
wcaI	-0.32005035	1.25326289	0.798434501	0.999867943
curA	-0.322159848	0.88365506	0.715427577	0.999867943
yfgJ	-0.322886649	3.004514488	0.914418374	0.999867943
xdhA	-0.323186203	0.60202023	0.591380821	0.999867943
lysT	-0.323299931	2.507424188	0.897407443	0.999867943
glgX	-0.323678505	0.6201415	0.601710028	0.999867943
yaaJ	-0.323806979	0.666280331	0.626972766	0.999867943
yfhH	-0.324069322	0.820296125	0.692795788	0.999867943
ydiB	-0.324434671	1.147948571	0.777467182	0.999867943
yggX	-0.324520043	2.499531283	0.896699061	0.999867943
hpt	-0.32480341	1.990726839	0.870393876	0.999867943
inaA	-0.325249277	0.976857466	0.739168484	0.999867943
narY	-0.325581559	0.777998097	0.675591588	0.999867943
ynbD	-0.326526102	0.756670292	0.66608288	0.999867943
ybaL	-0.326640316	0.59964441	0.585943798	0.999867943
rpmB	-0.326955853	1.579307102	0.835990433	0.999867943
intF	-0.3278804	0.637105074	0.606803901	0.999867943
elfD	-0.328731713	1.773065044	0.852912896	0.999867943
dnaB	-0.328938735	0.664053257	0.620353131	0.999867943
pyrB	-0.329405714	0.950860369	0.72902025	0.999867943
rayT	-0.329425534	0.708261573	0.641846625	0.999867943
clpS	-0.330112324	2.516614844	0.895638327	0.999867943
mukE	-0.330463437	0.951092194	0.728248178	0.999867943
yffB	-0.331348695	2.412185122	0.89074266	0.999867943
katE	-0.331586495	0.697367044	0.634442419	0.999867943
ytfT	-0.331912346	1.047826332	0.751423634	0.999867943
astE	-0.331963016	0.86636526	0.70159557	0.999867943
mgtA	-0.332501362	0.664255212	0.616678941	0.999867943
ybjE	-0.332726451	1.215797778	0.78433882	0.999867943
xylA	-0.333261408	1.090046371	0.75980916	0.999867943
leuA	-0.333357722	0.69354365	0.630759157	0.999867943
hha	-0.333816798	2.05525419	0.870974198	0.999867943
ybhJ	-0.333841405	0.709965406	0.638196366	0.999867943
truA	-0.333972194	1.002154144	0.738942321	0.999867943
elaA	-0.334355705	1.073651344	0.755481915	0.999867943
dgkA	-0.334720008	2.411472876	0.889605712	0.999867943
ansP	-0.335026345	0.790686244	0.671773007	0.999867943
yebO	-0.335072971	2.431731722	0.890404874	0.999867943
modE	-0.335224656	0.927453963	0.71776596	0.999867943
ybaT	-0.335372986	0.723627439	0.643034073	0.999867943
clsA	-0.336041874	0.772428564	0.663529117	0.999867943
hyfD	-0.336225643	0.855828476	0.694418685	0.999867943

ligB	-0.336408165	0.914055628	0.712844116	0.999867943
chiQ	-0.33714143	2.589178432	0.896398906	0.999867943
yhbY	-0.337254674	1.250218448	0.787347516	0.999867943
ydcC	-0.337355094	1.571335996	0.830006554	0.999867943
mhpF	-0.338458581	0.960469571	0.724546809	0.999867943
preT	-0.339033259	0.733505284	0.643930946	0.999867943
bisC	-0.339282746	0.635985271	0.593704264	0.999867943
ycjF	-0.339402714	1.044785297	0.74529155	0.999867943
gltP	-0.339582549	0.752534527	0.65180809	0.999867943
ylbA	-0.341557543	1.837431165	0.852532107	0.999867943
metG	-0.341726101	0.648355983	0.598148071	0.999867943
kch	-0.342096286	0.930497718	0.713135189	0.999867943
yjfC	-0.342420768	0.94374653	0.716730919	0.999867943
pinR	-0.343160734	2.308149476	0.881811248	0.999867943
yejB	-0.343431536	0.829123131	0.678719927	0.999867943
yjjP	-0.343470463	1.238538525	0.781535049	0.999867943
mqo	-0.343515346	0.73206601	0.638897422	0.999867943
macB	-0.343939241	0.72757689	0.636413845	0.999867943
ypjJ	-0.344254354	2.661275432	0.897075246	0.999867943
recJ	-0.344888833	0.839939289	0.681357341	0.999867943
yhbW	-0.345136158	0.782795551	0.659283915	0.999867943
hokD	-0.345459765	3.370819471	0.918371405	0.999867943
pheA	-0.346438783	0.734466766	0.63714987	0.999867943
secM	-0.346804449	0.973830864	0.721747776	0.999867943
yiaL	-0.347560794	2.411950467	0.88542192	0.999867943
yjhX	-0.348694446	2.114155547	0.868996558	0.999867943
yliE	-0.349011684	1.033938523	0.735698146	0.999867943
chbC	-0.349726945	0.807076902	0.664778367	0.999867943
ybbW	-0.350192476	1.028101289	0.733389221	0.999867943
yeaX	-0.350265038	0.891732	0.694473244	0.999867943
kbaY	-0.350356038	0.995996673	0.72501506	0.999867943
yfcP	-0.351360768	1.083578269	0.745741425	0.999867943
ynfC	-0.352000635	1.117864483	0.752847696	0.999867943
lyxK	-0.352347556	0.773933468	0.64891606	0.999867943
narH	-0.352731102	0.773359845	0.648316511	0.999867943
lldR	-0.352760709	0.908873566	0.697920148	0.999867943
ydfR	-0.354542009	2.639522509	0.893149136	0.999867943
insQ	-0.354749889	0.864992291	0.681718558	0.999867943
bcr	-0.355074688	0.715069417	0.619499545	0.999867943
ycaO	-0.356107758	0.781077843	0.64844866	0.999867943
yeeY	-0.356272612	0.955398548	0.709219371	0.999867943
yehW	-0.356400737	1.269110086	0.778842877	0.999867943
mzrA	-0.35698246	1.088672351	0.742982553	0.999867943
btuR	-0.357124849	1.060942284	0.736410158	0.999867943
ttdA	-0.357460355	1.011453883	0.723779283	0.999867943
zwf	-0.357774275	0.682804906	0.600294325	0.999867943
nanK	-0.359201285	1.242299185	0.772472459	0.999867943
rpoA	-0.359556471	1.462462355	0.805793004	0.999867943
ycbJ	-0.35984011	0.759315949	0.635570955	0.999867943

obgE	-0.360176307	0.711838242	0.612870302	0.999867943
yfiR	-0.360425935	2.342978201	0.877741895	0.999867943
fdrA	-0.360437238	0.849578582	0.671380475	0.999867943
bcsG	-0.360494984	0.961276431	0.707647839	0.999867943
putA	-0.360746247	0.824056728	0.661553959	0.999867943
betA	-0.360926401	0.896609821	0.687282485	0.999867943
cspE	-0.361096488	1.405616089	0.797259423	0.999867943
alaV	-0.36133805	2.460905912	0.883265167	0.999867943
dicA	-0.361643823	1.999876282	0.856498583	0.999867943
ydhQ	-0.36174741	0.888238376	0.683814187	0.999867943
cbtA	-0.362483981	2.569290529	0.887804138	0.999867943
nsrR	-0.363147655	2.408128149	0.880132854	0.999867943
yiaD	-0.363202533	1.164592957	0.755138717	0.999867943
lolE	-0.363492371	0.75973053	0.63233061	0.999867943
cybB	-0.365063841	1.947443438	0.851301556	0.999867943
yecM	-0.365358291	1.775079387	0.836926478	0.999867943
ltaE	-0.365394708	1.114177865	0.742949416	0.999867943
mtr	-0.365559476	0.658529711	0.578816257	0.999867943
yqgA	-0.365832732	1.260563291	0.771652759	0.999867943
dapA	-0.366224578	0.943480102	0.697895012	0.999867943
hisP	-0.366582716	1.067511924	0.731298179	0.999867943
nuoB	-0.367585184	1.063999739	0.729737391	0.999867943
yggN	-0.368053074	0.958894898	0.701104107	0.999867943
mlc	-0.36872261	0.853850955	0.665861439	0.999867943
yjbB	-0.368772454	0.693796372	0.595052687	0.999867943
ybiT	-0.3696226	0.668751807	0.580465293	0.999867943
zntR	-0.37036402	2.449232905	0.879805066	0.999867943
fabR	-0.370797089	0.934055762	0.691385658	0.999867943
ribA	-0.371574875	0.826397614	0.652975755	0.999867943
bamA	-0.371739748	0.870312862	0.66928212	0.999867943
purM	-0.372036323	0.959430681	0.698187877	0.999867943
yhfY	-0.372473651	2.603302327	0.886229077	0.999867943
yajI	-0.372926517	2.323977136	0.872511562	0.999867943
atoB	-0.373108942	0.893116257	0.67612203	0.999867943
ppiB	-0.373617814	1.150542741	0.745384066	0.999867943
metJ	-0.374208952	1.606916667	0.815859326	0.999867943
elaD	-0.374856277	1.23682637	0.761829548	0.999867943
argQ	-0.375412375	2.45841599	0.87863093	0.999867943
yecJ	-0.375551202	2.449792141	0.878162323	0.999867943
ygfK	-0.375569607	0.636011409	0.554850354	0.999867943
yfiP	-0.375959423	0.974252913	0.699574362	0.999867943
ydcR	-0.376593535	0.796848793	0.63649608	0.999867943
glnV	-0.377125543	3.018044109	0.900557644	0.999867943
rpsC	-0.377235346	1.395213647	0.786869314	0.999867943
pdxK	-0.377443277	1.062268636	0.722351294	0.999867943
cirA	-0.378044938	0.719519918	0.599296292	0.999867943
ung	-0.378198482	0.98137273	0.699958118	0.999867943
hybO	-0.378667551	1.071931136	0.723895518	0.999867943
ydeO	-0.38017464	1.837504624	0.83609011	0.999867943

yiiQ	-0.380397666	2.296114355	0.868416519	0.999867943
yojI	-0.380406846	0.752313763	0.613102942	0.999867943
mltB	-0.381777025	0.704411378	0.587832141	0.999867943
glf	-0.382700879	1.311171517	0.770380629	0.999867943
nrfF	-0.383643217	2.40094924	0.873047966	0.999867943
mcbA	-0.384297969	2.540112847	0.879745623	0.999867943
aidB	-0.384711175	0.710208799	0.588033854	0.999867943
rpsG	-0.384771215	1.225583962	0.753559553	0.999867943
yfeC	-0.385187947	2.54073746	0.879498662	0.999867943
ygiQ	-0.38534222	0.617010705	0.532279017	0.999867943
ypfN	-0.385939778	2.442473092	0.874447433	0.999867943
mglC	-0.386140634	0.834871065	0.643711839	0.999867943
yjeN	-0.387341034	2.40387117	0.871989285	0.999867943
dksA	-0.387493376	1.208519286	0.74848714	0.999867943
paaC	-0.387965798	0.945121201	0.681444213	0.999867943
xerC	-0.388035154	0.845984047	0.646464665	0.999867943
wcaH	-0.389359025	1.429827866	0.78538227	0.999867943
yceM	-0.390076418	0.994155419	0.694785104	0.999867943
valS	-0.392478728	0.615773558	0.523880549	0.999867943
rpsI	-0.393086294	1.30846222	0.763857933	0.999867943
mraZ	-0.395242778	1.292045849	0.759677278	0.999867943
puuD	-0.395523763	1.097999263	0.718680938	0.999867943
ydiJ	-0.395963177	0.675480572	0.557744638	0.999867943
yfjQ	-0.396189184	1.028469947	0.700072992	0.999867943
yegH	-0.396203177	0.777351165	0.610273111	0.999867943
yjaH	-0.397408201	0.851854161	0.640842329	0.999867943
serV	-0.397723498	2.177565917	0.855075863	0.999867943
ydiN	-0.398032609	1.161781021	0.731895237	0.999867943
insI1	-0.39878424	0.669393669	0.551349276	0.999867943
alkB	-0.398918776	0.916448625	0.663353642	0.999867943
yidJ	-0.399453257	1.075565502	0.710347776	0.999867943
eutM	-0.399517591	1.574527352	0.799697891	0.999867943
uspC	-0.399521428	2.440904161	0.869984922	0.999867943
rnd	-0.400016445	0.822715429	0.626814855	0.999867943
ydcO	-0.400228161	0.785468198	0.610373148	0.999867943
dcm	-0.400348485	0.684490585	0.55862484	0.999867943
murP	-0.40103845	1.020261418	0.694264672	0.999867943
yfdK	-0.40109762	2.399911451	0.867267624	0.999867943
yibL	-0.401320416	1.267758321	0.751578184	0.999867943
yiaR	-0.401457175	1.028148771	0.696191965	0.999867943
hyaB	-0.402907256	0.867706702	0.642407203	0.999867943
yehM	-0.40300257	0.831153225	0.627767449	0.999867943
nadA	-0.403316088	1.016652685	0.691581469	0.999867943
yidC	-0.403751997	0.915841792	0.659319108	0.999867943
dacA	-0.404042357	0.911878789	0.657702237	0.999867943
nudE	-0.405420915	1.955772914	0.835779891	0.999867943
degS	-0.405567221	0.858687269	0.636705004	0.999867943
zraS	-0.405727034	0.743781316	0.585414925	0.999867943
yedR	-0.405748675	1.978706483	0.837527173	0.999867943

pheP	-0.406157107	0.780726743	0.602903605	0.999867943
frdB	-0.406976421	1.261025396	0.74689631	0.999867943
yfbV	-0.407207282	1.038330319	0.694928852	0.999867943
galU	-0.407212586	2.287500871	0.858710125	0.999867943
ulaB	-0.407886129	2.571350525	0.873962599	0.999867943
queE	-0.408281176	1.095611397	0.709407875	0.999867943
ydgA	-0.408540038	0.70880872	0.564361346	0.999867943
yacG	-0.40928541	2.511534039	0.870548104	0.999867943
agaC	-0.409824116	0.986727151	0.677895351	0.999867943
emtA	-0.410163614	0.966902181	0.671417163	0.999867943
uspF	-0.410189195	2.370244002	0.862606001	0.999867943
ybfF	-0.410891697	1.951401877	0.833228821	0.999867943
rhlE	-0.413453454	0.731862444	0.572119144	0.999867943
ydfV	-0.414639628	2.096398961	0.843212029	0.999867943
mdtG	-0.414689593	0.776223841	0.593175704	0.999867943
crcB	-0.414690154	2.334562432	0.859012947	0.999867943
opgG	-0.415464203	0.787738071	0.597906022	0.999867943
rspB	-0.4155403	1.154010229	0.718784525	0.999867943
ompF	-0.417059301	1.015495758	0.681295999	0.999867943
ygcR	-0.418902047	0.99399701	0.67343973	0.999867943
bioH	-0.419600849	0.902527063	0.641990361	0.999867943
yegE	-0.419657021	0.696086755	0.546588294	0.999867943
hipA	-0.420763481	1.093210842	0.700320569	0.999867943
hcp	-0.42098794	0.762902734	0.58106903	0.999867943
rpsB	-0.421830575	1.282019136	0.742128403	0.999867943
ybdG	-0.422761069	1.250564617	0.735320869	0.999867943
actP	-0.423511692	0.659225605	0.520588722	0.999867943
lysA	-0.423553821	0.885189922	0.632302057	0.999867943
yeiI	-0.424091658	0.770165835	0.581873768	0.999867943
proY	-0.425270928	1.003689224	0.671778944	0.999867943
nuoI	-0.425366035	0.853006766	0.618014236	0.999867943
atpD	-0.425916167	1.079193872	0.693092791	0.999867943
dsbG	-0.426176579	1.932843684	0.825487998	0.999867943
pepT	-0.427853556	1.145953705	0.708880344	0.999867943
rmf	-0.428143594	1.303148814	0.742499297	0.999867943
folE	-0.428149125	0.863698086	0.620094662	0.999867943
dcuB	-0.428962699	1.221945744	0.725551669	0.999867943
yfbO	-0.429166388	2.342510022	0.85463488	0.999867943
ccmC	-0.429997854	0.953424279	0.6519869	0.999867943
wzxB	-0.430769051	1.838721803	0.814770438	0.999867943
yegQ	-0.431053005	0.690693858	0.532570501	0.999867943
rutG	-0.431667479	0.705267903	0.54049693	0.999867943
metF	-0.43208999	2.267175028	0.848850592	0.999867943
sbmC	-0.432455522	2.336793581	0.853179121	0.999867943
ftsX	-0.432510069	0.748151878	0.563193477	0.999867943
yeeD	-0.433462103	2.699566092	0.872434193	0.999867943
uxaC	-0.433631513	0.997121237	0.663647036	0.999867943
ygeY	-0.434030335	0.891411503	0.62632755	0.999867943
leuT	-0.434558873	2.854095264	0.87898344	0.999867943

topB	-0.434785242	0.848609984	0.608406146	0.999867943
yqgC	-0.434961886	2.45287148	0.859251049	0.999867943
glnW	-0.43651182	2.451778815	0.858692485	0.999867943
ynfK	-0.437500121	0.98474226	0.656841018	0.999867943
tyrU	-0.439097393	2.03065617	0.828805165	0.999867943
gadC	-0.43935629	1.139017781	0.699694716	0.999867943
gfcC	-0.439767567	0.981580966	0.654139011	0.999867943
secG	-0.440233971	1.171097912	0.706979645	0.999867943
yqeC	-0.441010885	1.357918072	0.745355244	0.999867943
nuoH	-0.442731553	0.967342373	0.647183625	0.999867943
atoC	-0.443517308	1.038408715	0.669297413	0.999867943
ypeA	-0.443892049	1.970268939	0.821749667	0.999867943
yejE	-0.445575301	0.800803298	0.577930316	0.999867943
allD	-0.445633581	1.031007921	0.665573534	0.999867943
ddpX	-0.446671349	1.07606708	0.678071486	0.999867943
trxA	-0.44737938	1.191715061	0.707357048	0.999867943
nupC	-0.44797614	1.080220542	0.678355648	0.999867943
uof	-0.448221083	2.767860695	0.871354889	0.999867943
frr	-0.449028511	1.034021617	0.664103661	0.999867943
kefB	-0.44939312	0.659479602	0.495595671	0.999867943
yedL	-0.449658316	1.992965741	0.821494882	0.999867943
ygjK	-0.449783116	0.686913525	0.512603898	0.999867943
rtcA	-0.450099075	1.14225471	0.693548257	0.999867943
dcuA	-0.450421073	1.114321375	0.686057489	0.999867943
ynbC	-0.450938294	0.855212702	0.597997748	0.999867943
araH	-0.451256468	1.026191646	0.660126167	0.999867943
glrK	-0.4513967	0.863268264	0.601048903	0.999867943
rcnB	-0.451750851	2.510653362	0.857204711	0.999867943
yafL	-0.45345407	0.614363291	0.460461077	0.999867943
appA	-0.454353864	0.797496297	0.568863987	0.999867943
aceA	-0.454415128	0.725102671	0.530861977	0.999867943
argY	-0.454697206	2.48327797	0.854716709	0.999867943
hyfI	-0.455209714	1.186135196	0.701144794	0.999867943
dnaE	-0.455304263	0.567727176	0.422566135	0.999867943
gltS	-0.455484343	0.818770504	0.578003857	0.999867943
yjhG	-0.455513297	0.878709689	0.604186999	0.999867943
ybjL	-0.455809754	0.708027577	0.519721988	0.999867943
ivbL	-0.456399612	3.473468777	0.895462119	0.999867943
ybiO	-0.456689465	0.698933698	0.513492734	0.999867943
cvrA	-0.45786276	0.688771124	0.506208407	0.999867943
gpmA	-0.457982838	0.872617812	0.599695931	0.999867943
pldA	-0.458793133	1.942618442	0.813298786	0.999867943
ackA	-0.458797699	0.896040265	0.608631418	0.999867943
menF	-0.459527386	0.815896717	0.573286724	0.999867943
manX	-0.46035138	0.864633911	0.594432739	0.999867943
murI	-0.460982087	1.659261615	0.78114775	0.999867943
yceA	-0.46209388	1.10984568	0.677147561	0.999867943
gspF	-0.462837212	1.055443385	0.661006506	0.999867943
yhaL	-0.462856199	2.783799589	0.867946215	0.999867943

ompW	-0.46311234	2.394322609	0.846629249	0.999867943
yjgM	-0.463149114	1.956817452	0.812901262	0.999867943
bcp	-0.463326212	2.341917371	0.843169822	0.999867943
ccmF	-0.463593849	0.889901802	0.60240199	0.999867943
nagK	-0.463627146	0.775376472	0.549881227	0.999867943
ygjQ	-0.463872163	1.077911245	0.666945745	0.999867943
prs	-0.464193971	1.176328004	0.693128739	0.999867943
rplJ	-0.464409038	1.509580471	0.758355422	0.999867943
ompN	-0.46461051	1.829556651	0.799536467	0.999867943
uidR	-0.465470456	2.315532306	0.840682209	0.999867943
ybhG	-0.465505037	0.998845069	0.641184518	0.999867943
ampG	-0.466113115	0.731758918	0.524140257	0.999867943
aceB	-0.466523162	0.750266666	0.534066945	0.999867943
rssA	-0.46676979	1.113170467	0.674985465	0.999867943
prpE	-0.467130024	1.188050382	0.694178848	0.999867943
ydiL	-0.467226931	2.442537982	0.848300371	0.999867943
carB	-0.467474117	0.652198979	0.473518434	0.999867943
mlaE	-0.468585748	0.704037353	0.505686344	0.999867943
hypF	-0.468621123	0.877972811	0.59351204	0.999867943
ygdH	-0.470200729	0.898727445	0.600845511	0.999867943
nlpE	-0.473269755	1.15739583	0.682606328	0.999867943
phnD	-0.473666652	0.80833757	0.557891447	0.999867943
ycjW	-0.473936872	1.114236144	0.670583821	0.999867943
ybcI	-0.474214416	1.053566462	0.652635495	0.999867943
csgE	-0.474671151	2.391846977	0.842690018	0.999867943
fur	-0.475319584	1.230421911	0.699269927	0.999867943
yghT	-0.477074246	2.278497229	0.834150749	0.999867943
ssuE	-0.477426436	1.007476739	0.635583105	0.999867943
hisM	-0.477956746	0.940108688	0.611168759	0.999867943
ybcH	-0.478349125	0.95792188	0.61752487	0.999867943
thiC	-0.478902765	0.729802556	0.51168987	0.999867943
purC	-0.479194781	2.293616062	0.834506432	0.999867943
tdh	-0.479203145	2.248860179	0.831259009	0.999867943
yeeA	-0.479731602	1.137930211	0.673329693	0.999867943
thiL	-0.479827654	0.756316175	0.525801976	0.999867943
lsrG	-0.480435155	2.508790131	0.848133305	0.999867943
pspB	-0.480677677	2.11890957	0.820539267	0.999867943
bglX	-0.481444524	0.669789369	0.472264226	0.999867943
yahJ	-0.481551854	0.934141943	0.606202412	0.999867943
ycjT	-0.482491601	0.819142227	0.555847492	0.999867943
phnJ	-0.483714186	1.13258614	0.669315081	0.999867943
wzzB	-0.484212477	1.590641012	0.760812942	0.999867943
ybhQ	-0.484573563	0.954225899	0.611580636	0.999867943
gloA	-0.485172856	1.426168344	0.733710995	0.999867943
hycA	-0.485185021	2.38427233	0.838748965	0.999867943
lnt	-0.485754537	0.710499633	0.494177124	0.999867943
znuA	-0.485874027	0.708044635	0.49257471	0.999867943
potC	-0.485938882	0.912358058	0.594297616	0.999867943
wcaC	-0.485978509	0.903481448	0.590649269	0.999867943

pyrI	-0.486526665	2.375635546	0.837729536	0.999867943
citG	-0.486728387	0.895399913	0.58672522	0.999867943
argR	-0.486874863	2.375653675	0.837616241	0.999867943
cdsA	-0.487108407	0.957486296	0.610936803	0.999867943
malQ	-0.487868423	0.641247388	0.446769631	0.999867943
recB	-0.487933616	0.652653998	0.454692694	0.999867943
rrfA	-0.488538006	2.725023636	0.857719069	0.999867943
yafP	-0.488823629	0.846260359	0.563515339	0.999867943
sapA	-0.489241548	0.763763125	0.521803966	0.999867943
nlpI	-0.489802979	1.237025389	0.692140259	0.999867943
fhuF	-0.489946768	0.831860487	0.555876643	0.999867943
cynT	-0.490765997	1.06624156	0.645317724	0.999867943
yneF	-0.491018129	1.163994851	0.673142722	0.999867943
ftsI	-0.491164883	0.739847818	0.506771691	0.999867943
rsxE	-0.491715422	1.058541121	0.642273948	0.999867943
yedW	-0.492160819	2.291581702	0.829947387	0.999867943
osmC	-0.492181887	2.345758094	0.833809852	0.999867943
glyY	-0.493357296	2.208802626	0.823255636	0.999867943
nrdH	-0.493677595	2.586837251	0.848649261	0.999867943
malF	-0.495000479	0.784270093	0.52793544	0.999867943
kbaZ	-0.49519191	0.834369059	0.552851495	0.999867943
glrR	-0.495691484	0.851946025	0.560678247	0.999867943
aroE	-0.495811251	1.837966015	0.787344253	0.999867943
ygfZ	-0.495942003	0.816249162	0.543461731	0.999867943
yneK	-0.496729358	1.833409299	0.786443287	0.999867943
yheO	-0.497249136	1.94548333	0.798266229	0.999867943
fnrS	-0.498276345	2.03210784	0.806300242	0.999867943
yceI	-0.498371262	1.123349377	0.657297381	0.999867943
rpsS	-0.498583835	1.555504078	0.748567512	0.999867943
manA	-0.499882556	0.80902144	0.536650868	0.999867943
pcnB	-0.499916329	0.822040689	0.543094218	0.999867943
yacC	-0.500047298	2.418364388	0.83618881	0.999867943
ykgL	-0.500463748	1.97306315	0.799767422	0.999867943
yhhW	-0.50074319	1.952241194	0.797567421	0.999867943
yehI	-0.501264108	0.745152757	0.501138297	0.999867943
rpmI	-0.503385259	1.464628404	0.731075774	0.999867943
ykgG	-0.503640186	0.664092102	0.448218105	0.999867943
ygaU	-0.503798646	2.352566639	0.830431246	0.999867943
ydhI	-0.504895358	2.543467563	0.842648642	0.999867943
fliH	-0.504943051	1.216919197	0.678189075	0.999867943
proQ	-0.505028963	0.773563521	0.513846303	0.999867943
ydfC	-0.50504781	2.076917281	0.807872276	0.999867943
ydcU	-0.505468774	0.939059343	0.590389703	0.999867943
dinG	-0.506173753	0.683711008	0.459098022	0.999867943
yjfN	-0.506764673	1.497937026	0.735130797	0.999867943
pitB	-0.507025243	0.855854919	0.553569633	0.999867943
glxR	-0.507321579	1.826456375	0.781194474	0.999867943
lysZ	-0.507918699	2.617961664	0.846165672	0.999867943
ykgB	-0.508053931	1.026795734	0.620744466	0.999867943

kdpB	-0.508435439	0.884631063	0.565465177	0.999867943
hycD	-0.508665051	1.001599079	0.61155647	0.999867943
yecA	-0.508764603	0.985683956	0.605746935	0.999867943
yoaD	-0.50883101	0.850151641	0.549494391	0.999867943
metC	-0.509554248	1.761678391	0.772394948	0.999867943
yfjI	-0.509617512	0.991615005	0.607303179	0.999867943
flhA	-0.510816338	0.745203857	0.493046158	0.999867943
pbpC	-0.511637233	0.889168521	0.565013454	0.999867943
lldD	-0.515575434	0.899947103	0.566715611	0.999867943
yiiX	-0.515720639	1.943958429	0.790782934	0.999867943
yggM	-0.51601986	1.248064702	0.679272541	0.999867943
aroB	-0.516608059	0.771990937	0.503374744	0.999867943
sad	-0.516814698	0.800677406	0.518621478	0.999867943
ampC	-0.517265031	1.92734038	0.788404668	0.999867943
yniA	-0.517648086	1.056793538	0.624254932	0.999867943
gstB	-0.517944078	1.120128448	0.643796622	0.999867943
umuD	-0.517980285	2.424299933	0.830810803	0.999867943
rihA	-0.518218596	1.097462156	0.636786013	0.999867943
yejG	-0.518799867	1.272315571	0.683449612	0.999867943
rem	-0.518954352	2.355268256	0.825608174	0.999867943
trpD	-0.518972256	1.011689994	0.607968407	0.999867943
lysY	-0.519569063	2.584778302	0.840689915	0.999867943
paaX	-0.519775054	0.96424706	0.589854018	0.999867943
yghB	-0.519859858	0.927394369	0.575097769	0.999867943
rhaD	-0.519995296	0.85509559	0.543112103	0.999867943
folX	-0.520390296	2.484542318	0.834096051	0.999867943
ada	-0.521157423	1.270629687	0.681690919	0.999867943
mprA	-0.522246703	1.21030808	0.666105594	0.999867943
feoA	-0.523174204	2.483232859	0.833134875	0.999867943
lysW	-0.52337739	2.599857428	0.840456156	0.999867943
araC	-0.523524963	1.92888408	0.786073106	0.999867943
pck	-0.523953846	1.01679452	0.606343653	0.999867943
sapD	-0.52400857	0.907327911	0.563581864	0.999867943
yaiP	-0.5251756	1.089719621	0.629851096	0.999867943
malK	-0.525954425	1.166455305	0.652061792	0.999867943
cysH	-0.526725538	2.294623404	0.818443247	0.999867943
nadE	-0.527358874	1.766717933	0.765324353	0.999867943
rnb	-0.527419091	0.672948106	0.433190295	0.999867943
yeaN	-0.52750536	0.861717104	0.5404345	0.999867943
bhsA	-0.528084414	2.396998584	0.825629195	0.999867943
csrC	-0.52918597	2.494946441	0.832026571	0.999867943
gatD	-0.529367521	0.952544755	0.578388394	0.999867943
gfcD	-0.529489222	0.877472119	0.546225534	0.999867943
yjjB	-0.529855885	1.975832976	0.78856973	0.999867943
feoC	-0.529896377	2.744723619	0.846911969	0.999867943
ybeT	-0.529961761	1.96927843	0.787841424	0.999867943
dedA	-0.531544679	0.996952493	0.593916241	0.999867943
fepE	-0.533661165	1.213263798	0.660041522	0.999867943
fumA	-0.533934116	0.711097876	0.452737622	0.999867943

hipB	-0.534088231	2.505484514	0.831196205	0.999867943
yqgE	-0.534103269	1.096975668	0.626338362	0.999867943
dhaM	-0.534526817	1.135870324	0.637935059	0.999867943
yecE	-0.535013428	0.906829269	0.55520236	0.999867943
yohJ	-0.53592192	2.402346222	0.82347113	0.999867943
paoC	-0.53595693	1.102684715	0.626933629	0.999867943
yeaD	-0.536846835	1.064659678	0.614090861	0.999867943
deoD	-0.538014661	1.154809287	0.641293891	0.999867943
clpA	-0.538809795	0.908943114	0.553323611	0.999867943
insE1	-0.538881227	0.954168618	0.572233483	0.999867943
murR	-0.540469195	0.956962453	0.572226297	0.999867943
gsiB	-0.540893747	0.766498633	0.480394471	0.999867943
tsaD	-0.541784288	0.917498804	0.554854577	0.999867943
arfB	-0.542309155	1.053130797	0.606588324	0.999867943
mepA	-0.542395192	0.909141439	0.550773436	0.999867943
hyfB	-0.542778027	0.786909883	0.490345873	0.999867943
iclR	-0.542820354	0.9903236	0.583606609	0.999867943
nadC	-0.543402968	0.912107532	0.551331332	0.999867943
yfaA	-0.543648628	0.797432425	0.495397781	0.999867943
eutN	-0.543859326	1.956903047	0.781075014	0.999867943
rsmC	-0.544918798	0.838267811	0.515657762	0.999867943
yebC	-0.54524607	0.9700456	0.57405952	0.999867943
manZ	-0.54535558	1.019178806	0.592585459	0.999867943
mngB	-0.545464422	1.04181087	0.600575312	0.999867943
yhdP	-0.546831307	0.739279379	0.459493254	0.999867943
yhgF	-0.547421849	0.744464442	0.462142786	0.999867943
yfgM	-0.548767766	0.883992102	0.53474204	0.999867943
wzxE	-0.548899244	1.013109944	0.587958824	0.999867943
ydjZ	-0.550358774	0.976535138	0.573037839	0.999867943
marA	-0.550656977	2.400921069	0.81859504	0.999867943
citX	-0.551411677	1.507336655	0.714500493	0.999867943
csgG	-0.552159712	0.951948661	0.561893728	0.999867943
metV	-0.55321361	2.429743045	0.819891914	0.999867943
hicB	-0.555403293	2.418668259	0.818377847	0.999867943
ydeE	-0.555601011	1.043042387	0.594259632	0.999867943
cobC	-0.555679094	2.309410057	0.809853391	0.999867943
ykgR	-0.556021291	2.051390922	0.786355658	0.999867943
mreD	-0.55805487	2.344655041	0.811872172	0.999867943
insF1	-0.558088528	1.089987232	0.608641336	0.999867943
yncG	-0.558131249	1.968408031	0.776759483	0.999867943
rplK	-0.558512613	1.276095497	0.661623306	0.999867943
fliT	-0.558662012	2.527893437	0.825093184	0.999867943
ydbJ	-0.558921797	2.11142414	0.791230424	0.999867943
ydjE	-0.561366128	1.104969546	0.611426942	0.999867943
flu	-0.561595127	0.674948823	0.405377412	0.999867943
trmA	-0.561933251	1.92661389	0.770539831	0.999867943
araJ	-0.561936545	0.784441	0.473773502	0.999867943
sfmH	-0.562790535	1.926438098	0.770179224	0.999867943
fadK	-0.563891743	0.81185871	0.487325561	0.999867943

uidA	-0.564657842	0.894153906	0.527713905	0.999867943
gmhB	-0.566215616	1.986075717	0.775573151	0.999867943
pssA	-0.567130352	0.756702434	0.453570328	0.999867943
ybiW	-0.567435807	0.80553565	0.481170957	0.999867943
ynjF	-0.567502397	1.09625755	0.604686782	0.999867943
yaiY	-0.567874989	2.423975559	0.814772097	0.999867943
ppdB	-0.568867559	1.763021754	0.74694804	0.999867943
ampH	-0.570677305	0.919323773	0.534759063	0.999867943
yghU	-0.571122632	0.909617834	0.53008847	0.999867943
yheU	-0.571456473	2.930106799	0.845370065	0.999867943
tolB	-0.572468862	0.89231869	0.521164091	0.999867943
yadL	-0.572720912	1.927775089	0.766398507	0.999867943
ybgP	-0.573845312	1.942958177	0.767729428	0.999867943
acpH	-0.573987122	2.394013277	0.810516804	0.999867943
fhuA	-0.57423988	1.065462307	0.589915546	0.999867943
ygaP	-0.574649294	1.831635055	0.753721789	0.999867943
gmr	-0.575191499	1.112847638	0.605250712	0.999867943
pheS	-0.57573624	0.794750746	0.468805682	0.999867943
yecC	-0.575763135	0.961518721	0.549302292	0.999867943
ygcP	-0.578833691	1.831924705	0.752025096	0.999867943
mhpC	-0.581074944	0.914053561	0.524964063	0.999867943
ycaR	-0.581164624	2.617130293	0.824265786	0.999867943
ybdM	-0.582415692	1.928162664	0.762608349	0.999867943
modA	-0.582448117	1.19918259	0.627176849	0.999867943
etp	-0.584863273	2.576333625	0.820413015	0.999867943
ybjX	-0.586568742	0.79638486	0.461402564	0.999867943
ycjS	-0.586611054	0.925721572	0.526289814	0.999867943
ydgH	-0.586715505	0.999114854	0.557045691	0.999867943
rzoR	-0.587607068	2.561550823	0.818561886	0.999867943
yebK	-0.587770426	1.072387034	0.583626382	0.999867943
ycgR	-0.588001164	1.938766413	0.761671736	0.999867943
ycgN	-0.588446654	1.9594466	0.763938664	0.999867943
yabI	-0.588968762	0.790300208	0.456122488	0.999867943
ydiV	-0.589099782	1.938211946	0.761173743	0.999867943
ybiP	-0.589371316	1.063845482	0.579578254	0.999867943
azoR	-0.591275085	1.018890612	0.561703816	0.999867943
dosP	-0.59141589	1.12618229	0.599478142	0.999867943
pyrG	-0.592038551	1.005520296	0.556003319	0.999867943
aqpZ	-0.594774493	1.04182609	0.568070048	0.999867943
rplE	-0.595123845	1.466859025	0.684953454	0.999867943
fhlA	-0.595493612	0.891522769	0.504164809	0.999867943
yfjZ	-0.596783504	2.098593488	0.776124483	0.999867943
rpoC	-0.597871169	0.961620913	0.534117645	0.999867943
yshB	-0.5999227	3.059911637	0.844564119	0.999867943
yfeH	-0.600579155	1.104309713	0.586544501	0.999867943
ydiT	-0.60096776	2.828756428	0.831756407	0.999867943
torS	-0.601700549	0.726647433	0.407642074	0.999867943
ispE	-0.602268762	0.820527456	0.462947543	0.999867943
trxC	-0.602588799	2.386101233	0.800622795	0.999867943

lipB	-0.603270656	2.27988444	0.791313148	0.999867943
nrfG	-0.604134837	2.38891245	0.800352322	0.999867943
yahG	-0.604377936	1.225924199	0.62201414	0.999867943
ompG	-0.604631348	1.821820155	0.739977521	0.999867943
yaaI	-0.60482331	1.955805518	0.757135075	0.999867943
rdoA	-0.604936378	1.029506953	0.556802055	0.999867943
yciH	-0.607963405	2.59048015	0.814448266	0.999867943
pflC	-0.608743823	0.989948892	0.538604585	0.999867943
crp	-0.608759126	1.265642988	0.630525007	0.999867943
ptrB	-0.608952702	0.885068532	0.491434601	0.999867943
ppk	-0.609244613	0.959176784	0.525314627	0.999867943
ymcE	-0.612303985	2.681314779	0.81936669	0.999867943
purH	-0.612661718	0.726155372	0.398833657	0.999867943
fepB	-0.613283715	0.885565755	0.488602523	0.999867943
hemL	-0.615069948	0.799616606	0.441770987	0.999867943
rpsH	-0.615260582	1.415103716	0.663721206	0.999867943
yceJ	-0.615275823	2.395629146	0.797307951	0.999867943
deaD	-0.615722434	1.169044598	0.598409871	0.999867943
gmk	-0.615736684	1.097805103	0.574879495	0.999867943
cycA	-0.616175634	0.899342536	0.493255518	0.999867943
mntH	-0.616433213	1.019587937	0.545451099	0.999867943
metN	-0.616933526	1.018874892	0.544843709	0.999867943
mnmC	-0.616976766	0.727894931	0.396650807	0.999867943
csdA	-0.617027639	0.741237771	0.405166974	0.999867943
yoaE	-0.617374149	0.744107201	0.406717234	0.999867943
cptB	-0.617860189	2.538129131	0.807671264	0.999867943
agaV	-0.617915608	1.828598627	0.735425305	0.999867943
yfcI	-0.618456829	2.272595737	0.785516869	0.999867943
eda	-0.619925929	0.935544994	0.507563614	0.999867943
yicC	-0.621743927	1.230610811	0.613395917	0.999867943
yfbK	-0.622071892	1.1850564	0.599631715	0.999867943
dnaJ	-0.622694118	0.790052119	0.430598208	0.999867943
phnM	-0.623075191	1.693763183	0.71297434	0.999867943
cysJ	-0.623749235	0.814509176	0.443796696	0.999867943
xdhD	-0.624636545	0.639993783	0.329062441	0.999867943
murQ	-0.624874441	0.934446211	0.503679863	0.999867943
marR	-0.625101429	1.998324158	0.75442287	0.999867943
murF	-0.625707394	0.878048142	0.476086059	0.999867943
ribB	-0.625748207	2.296138084	0.785220656	0.999867943
rplU	-0.626105864	1.285290266	0.626164876	0.999867943
abrB	-0.626598328	0.85317178	0.462684296	0.999867943
fbaA	-0.627415518	1.094431452	0.566455214	0.999867943
agaD	-0.627425496	1.09522043	0.566728658	0.999867943
ybdH	-0.627816659	0.773506194	0.416992228	0.999867943
ygfF	-0.628323204	0.905625682	0.487807569	0.999867943
speA	-0.629617078	0.799776764	0.431140787	0.999867943
epmB	-0.63015916	1.935131311	0.744695572	0.999867943
codB	-0.630380596	0.679599031	0.353626906	0.999867943
yehR	-0.630970412	2.304092982	0.784201716	0.999867943

yciB	-0.631985961	2.365354937	0.78932718	0.999867943
ycbC	-0.633050872	0.944749865	0.502811596	0.999867943
yjhP	-0.633536335	1.140315087	0.578497959	0.999867943
artM	-0.63395947	1.067676527	0.552662713	0.999867943
ybbY	-0.636976085	1.110131072	0.566113588	0.999867943
insF1	-0.637769784	1.237651531	0.606338911	0.999867943
yggD	-0.63777264	1.763799549	0.717658286	0.999867943
hofB	-0.638326469	0.962987639	0.507419901	0.999867943
ilvH	-0.639526977	2.305623959	0.781490667	0.999867943
opgH	-0.640077806	0.640500281	0.317629822	0.999867943
idnO	-0.640303397	2.311819915	0.781803595	0.999867943
yqcE	-0.640473476	1.918528486	0.738503473	0.999867943
garD	-0.640965076	0.964717235	0.506429726	0.999867943
treC	-0.64135419	0.924908706	0.488043305	0.999867943
yjdP	-0.641715322	1.824102565	0.724989887	0.999867943
wecG	-0.642792598	1.138329489	0.572291021	0.999867943
ghrA	-0.643032018	1.041247075	0.536865752	0.999867943
rsmI	-0.644779377	0.914599355	0.480819196	0.999867943
mug	-0.648742206	1.951461284	0.739557651	0.999867943
gcl	-0.649607886	0.984630099	0.509415462	0.999867943
pflB	-0.649981907	0.909089699	0.474620753	0.999867943
yfgH	-0.650072453	2.320318031	0.779350735	0.999867943
ispA	-0.650103522	0.905971739	0.473018797	0.999867943
rsd	-0.650372127	1.974263401	0.741834319	0.999867943
yjjU	-0.650378355	0.861944758	0.45052047	0.999867943
elaB	-0.651020496	2.541593477	0.79783765	0.999867943
mntP	-0.651178814	1.005377213	0.517181582	0.999867943
murD	-0.651474664	0.784148409	0.406083621	0.999867943
gltL	-0.651861971	1.271731393	0.608246305	0.999867943
evgA	-0.652415699	1.958042217	0.738984848	0.999867943
rbsD	-0.65347941	1.36680517	0.632573899	0.999867943
ygbM	-0.653891755	1.933197091	0.73517954	0.999867943
moeA	-0.654431981	0.772808039	0.397093527	0.999867943
cusB	-0.654914623	0.875966346	0.45467241	0.999867943
greA	-0.654925386	2.370798049	0.78235851	0.999867943
rimP	-0.65512167	2.403304634	0.785166845	0.999867943
cobS	-0.655999717	0.986998912	0.506280286	0.999867943
yafS	-0.656515912	2.276234332	0.773023779	0.999867943
yfdE	-0.657401826	1.920387401	0.732104282	0.999867943
rseC	-0.657759179	0.927172297	0.478060797	0.999867943
flgA	-0.659322061	2.335067045	0.777669936	0.999867943
yegK	-0.660540085	1.068703737	0.53652527	0.999867943
rutB	-0.660762857	1.098990842	0.547676751	0.999867943
caiD	-0.660817885	0.999817381	0.5086517	0.999867943
mliC	-0.661927972	2.038724834	0.745425206	0.999867943
modF	-0.662327523	0.773532276	0.391866295	0.999867943
yoaF	-0.662649961	2.703015077	0.806338624	0.999867943
ybiA	-0.664432783	2.284613434	0.771181789	0.999867943
ybeX	-0.665595572	1.166095563	0.568142069	0.999867943

ytfK	-0.665978402	2.085775965	0.749502619	0.999867943
fliS	-0.667290941	2.528890059	0.791882311	0.999867943
phnG	-0.668739418	1.999696124	0.738062257	0.999867943
tyrP	-0.669264955	1.062579902	0.528793342	0.999867943
focA	-0.669346831	1.921802292	0.7276215	0.999867943
yddE	-0.670848711	0.888359344	0.450156181	0.999867943
mntR	-0.671752521	1.069535831	0.52995248	0.999867943
atpF	-0.672246335	1.115038614	0.546581448	0.999867943
yjeM	-0.672365624	1.76674183	0.703523738	0.999867943
cbrA	-0.672966289	1.92284785	0.726350586	0.999867943
ypjD	-0.673049228	1.004600367	0.502878742	0.999867943
rhaR	-0.673613774	1.745213264	0.699513026	0.999867943
maeA	-0.67381611	0.795452873	0.396947806	0.999867943
ybdD	-0.673862505	2.828747113	0.811710913	0.999867943
ychN	-0.675053964	1.972352846	0.732156578	0.999867943
ggt	-0.677846801	0.834887427	0.416847846	0.999867943
dhaR	-0.678214955	0.890977108	0.446535487	0.999867943
ygcQ	-0.678450878	0.844718679	0.42187773	0.999867943
cof	-0.678742458	2.306814623	0.768579379	0.999867943
panC	-0.679907287	1.042560619	0.514303558	0.999867943
ydhL	-0.68058862	2.59503036	0.793116258	0.999867943
pspE	-0.682134222	2.446228083	0.780358837	0.999867943
nrfC	-0.682257159	1.16916502	0.559528339	0.999867943
yfdN	-0.682456239	1.978236605	0.73010782	0.999867943
yqaA	-0.683073107	1.95642046	0.726980959	0.999867943
kgtP	-0.683566311	1.099916206	0.534289598	0.999867943
ulaC	-0.683895454	1.973906561	0.728991031	0.999867943
ulaE	-0.684353289	1.021262922	0.502790921	0.999867943
tusA	-0.685008187	2.450804387	0.77985852	0.999867943
rof	-0.685046596	1.839320705	0.709561233	0.999867943
mltF	-0.685488813	1.053540488	0.515270795	0.999867943
ydjJ	-0.68558811	1.816977885	0.705933351	0.999867943
ydjI	-0.686062723	1.927233107	0.721853102	0.999867943
puuB	-0.686603231	1.251661378	0.583311904	0.999867943
yaeP	-0.686628697	2.613184537	0.792739011	0.999867943
ydcF	-0.687179521	2.306483112	0.76575361	0.999867943
yhhS	-0.687318091	0.798912693	0.38961438	0.999867943
mlaF	-0.687974112	0.965951447	0.476325879	0.999867943
thiH	-0.688375899	1.032977112	0.505155493	0.999867943
ynbA	-0.688589516	2.313639549	0.765991851	0.999867943
cysN	-0.69144323	0.919336815	0.451984376	0.999867943
holB	-0.692364877	0.857258463	0.419292041	0.999867943
xseA	-0.692627424	0.772028671	0.369637567	0.999867943
ascG	-0.693736769	1.917605405	0.717522096	0.999867943
parE	-0.69435002	0.997874536	0.486535938	0.999867943
ypdC	-0.694807392	0.918367328	0.44930868	0.999867943
ydcH	-0.695161012	1.266616575	0.583120041	0.999867943
prpD	-0.695636056	0.918418876	0.448793562	0.999867943
ugpA	-0.695709844	0.897065805	0.438021014	0.999867943

rpsJ	-0.696255511	1.600518591	0.663548856	0.999867943
mdtP	-0.696363052	0.87892878	0.428193877	0.999867943
mgrB	-0.697110191	2.657332073	0.793063476	0.999867943
yfjY	-0.697635268	2.50898205	0.780970058	0.999867943
glyX	-0.69764607	2.146005872	0.745112597	0.999867943
atpI	-0.697954945	2.315623862	0.763100962	0.999867943
yneE	-0.69806166	1.8198745	0.701292129	0.999867943
pphA	-0.698117047	1.930813777	0.717676209	0.999867943
yiiD	-0.699248889	0.911759986	0.443127829	0.999867943
cysP	-0.699338596	0.911137308	0.442757737	0.999867943
ydjY	-0.700281426	2.317615899	0.762533185	0.999867943
exbD	-0.700325469	2.410262736	0.771388006	0.999867943
rplA	-0.700794037	1.163749668	0.547050174	0.999867943
agaS	-0.701294202	0.820493268	0.392704693	0.999867943
rlmC	-0.701525809	0.790891471	0.375075446	0.999867943
yphA	-0.703043192	1.93779837	0.716750458	0.999867943
frdA	-0.703389707	0.97727277	0.471680428	0.999867943
yfjD	-0.703733669	0.932759937	0.450570734	0.999867943
frlA	-0.704510486	0.803282764	0.380465337	0.999867943
appB	-0.705764481	1.018772738	0.488460499	0.999867943
fsr	-0.706315029	1.018808885	0.488136804	0.999867943
slyB	-0.708255082	1.103737748	0.521075892	0.999867943
yejA	-0.708313957	0.898952353	0.430736071	0.999867943
dgoT	-0.708974774	1.182362661	0.548755865	0.999867943
lysQ	-0.709561444	2.647552141	0.78869424	0.999867943
ybhA	-0.710390177	1.829556879	0.69780486	0.999867943
gntK	-0.711172054	1.837688781	0.698761648	0.999867943
lysV	-0.71153979	2.587959732	0.783360512	0.999867943
rplX	-0.711964333	1.504354735	0.636021337	0.999867943
yhdT	-0.712954429	2.600288472	0.783944129	0.999867943
yeiR	-0.712978923	0.938253495	0.447314303	0.999867943
purT	-0.714137244	1.113176707	0.521177818	0.999867943
basR	-0.714172574	1.834002194	0.696975384	0.999867943
yjcS	-0.714324746	1.058427825	0.499744219	0.999867943
ynfF	-0.715060691	0.970477802	0.461236058	0.999867943
ygiV	-0.715380977	2.382832733	0.764007131	0.999867943
ndh	-0.715631559	0.735808507	0.330762816	0.999867943
uvrB	-0.716909066	0.841902603	0.394472537	0.999867943
folA	-0.717050897	2.379940354	0.763193793	0.999867943
frmR	-0.721012159	2.546466411	0.777067817	0.999867943
yobD	-0.72123826	2.381894327	0.762042073	0.999867943
fdhD	-0.72286741	1.926379213	0.707477014	0.999867943
nudJ	-0.722983748	1.126845676	0.521133185	0.999867943
puuP	-0.723172508	0.929913134	0.436759168	0.999867943
rhtB	-0.723369853	2.286984464	0.751775917	0.999867943
casE	-0.723558468	2.261748041	0.749035668	0.999867943
fucK	-0.723625705	1.747517555	0.678809805	0.999867943
nadB	-0.723722613	0.887948928	0.415043794	0.999867943
ymbA	-0.72448375	2.447727301	0.767243663	0.999867943

yddM	-0.724599455	2.445700412	0.767020212	0.999867943
yebQ	-0.725651627	0.933399651	0.436905917	0.999867943
insE1	-0.725987604	1.019570236	0.476432225	0.999867943
fliQ	-0.726377634	2.083021232	0.727304405	0.999867943
udk	-0.726870948	2.308438394	0.7528561	0.999867943
ybaK	-0.727436454	1.932931158	0.706665245	0.999867943
yajL	-0.72773213	1.16359094	0.531695955	0.999867943
clsB	-0.729082436	0.902544164	0.419201093	0.999867943
livM	-0.731606162	0.759862402	0.335640872	0.999867943
yoaH	-0.732220136	3.014216888	0.808065683	0.999867943
sdiA	-0.73337575	1.061270042	0.48954295	0.999867943
csdL	-0.733767876	0.850554423	0.38830594	0.999867943
aspC	-0.734031442	0.916404636	0.423137124	0.999867943
uvrY	-0.73421253	2.324032651	0.75206188	0.999867943
rlpA	-0.734424048	0.889237411	0.408858989	0.999867943
rcbA	-0.734994837	2.409610517	0.760346041	0.999867943
norR	-0.735390232	0.828412031	0.374696383	0.999867943
yccM	-0.736262553	2.241736101	0.742583472	0.999867943
hisA	-0.73832891	1.275095646	0.562563492	0.999867943
dbpA	-0.73888075	0.994008484	0.457279219	0.999867943
csgA	-0.739668834	2.360094591	0.753971895	0.999867943
ygaV	-0.739936636	2.735249315	0.786761079	0.999867943
yehS	-0.743023322	2.340074828	0.75084817	0.999867943
ade	-0.744000114	1.213200711	0.539708445	0.999867943
fliY	-0.744757482	1.203819418	0.536138965	0.999867943
msrC	-0.74580974	1.717456653	0.664105137	0.999867943
hcaD	-0.746005705	0.866086753	0.38904411	0.999867943
sbmA	-0.746897019	1.16594691	0.521787377	0.999867943
potF	-0.746942583	0.98663947	0.449015553	0.999867943
ydhK	-0.747294003	0.83005792	0.367965199	0.999867943
ybjM	-0.747848348	1.969387405	0.704140998	0.999867943
rmuC	-0.749575056	0.794092894	0.345200947	0.999867943
yebB	-0.75009678	1.035928505	0.469015634	0.999867943
nadD	-0.750804696	1.537788548	0.625382243	0.999867943
ybfA	-0.752923795	2.506706211	0.763899675	0.999867943
lpxA	-0.754602544	0.934339458	0.419302399	0.999867943
wcaD	-0.756019104	1.729684865	0.662049791	0.999867943
mrdA	-0.756632351	1.102478148	0.492523035	0.999867943
ffs	-0.757965389	1.657093349	0.647378841	0.999867943
frmA	-0.759297396	0.855236189	0.374636575	0.999867943
ahpC	-0.759874337	1.254911299	0.544833303	0.999867943
waaN	-0.760203754	0.995788646	0.445213683	0.999867943
fxsA	-0.761743992	1.920458948	0.691627899	0.999867943
sodB	-0.763170551	1.182183638	0.518564305	0.999867943
yciW	-0.76411879	1.688400388	0.65085865	0.999867943
ssnA	-0.765566088	0.908271895	0.399294407	0.999867943
infB	-0.767521044	0.91082518	0.399415493	0.999867943
sgcA	-0.768402038	1.157205136	0.506680451	0.999867943
nudL	-0.7684167	1.975097239	0.697237242	0.999867943

insF1	-0.769024731	2.090237755	0.712938432	0.999867943
pgpC	-0.76994055	1.072714511	0.472911615	0.999867943
prc	-0.772648163	1.114164424	0.488009826	0.999867943
fpr	-0.773761592	0.873186111	0.37554428	0.999867943
lacY	-0.774204235	0.722573578	0.283965416	0.999867943
minC	-0.775417912	2.280582671	0.733849988	0.999867943
flgF	-0.775452231	1.315096101	0.555422358	0.999867943
yedA	-0.776978631	1.044243062	0.456840676	0.999867943
sixA	-0.777055282	1.973992368	0.69384201	0.999867943
priB	-0.77715206	1.529625188	0.611406367	0.999867943
yeiE	-0.777294595	0.911199563	0.393634129	0.999867943
lpd	-0.777323758	0.986640142	0.430784853	0.999867943
rsgA	-0.77789308	0.988552198	0.431339605	0.999867943
hyfJ	-0.779157375	2.55200657	0.760128792	0.999867943
hybG	-0.779400737	2.836492745	0.783488173	0.999867943
yeeS	-0.780026785	2.09051666	0.709054432	0.999867943
queA	-0.78117967	0.801433747	0.329695351	0.999867943
hofC	-0.782132871	1.909638665	0.682120576	0.999867943
hisF	-0.782159807	1.031038285	0.448083653	0.999867943
hemF	-0.784248207	0.926099685	0.397090359	0.999867943
sdhB	-0.784569915	1.040580635	0.450865312	0.999867943
uvrC	-0.784649061	0.970262019	0.418688816	0.999867943
zraP	-0.784721717	2.379367675	0.7415491	0.999867943
prmC	-0.785943061	1.008729662	0.435896413	0.999867943
sapC	-0.786627168	1.107149627	0.47739561	0.999867943
ydaU	-0.786729799	1.814295115	0.664558344	0.999867943
psuT	-0.787737784	0.908926091	0.386123566	0.999867943
abgT	-0.788965962	1.814534377	0.663705039	0.999867943
secD	-0.788988351	0.836495061	0.345574817	0.999867943
ycfZ	-0.790720144	1.917526973	0.680072244	0.999867943
pgl	-0.791583084	0.900706107	0.379484104	0.999867943
ycjD	-0.793642745	2.464278012	0.747408049	0.999867943
cspD	-0.793817599	2.3719304	0.737872873	0.999867943
eutP	-0.794336027	2.412101062	0.741919185	0.999867943
gltA	-0.796137726	1.074878353	0.458889279	0.999867943
hrpB	-0.796164691	0.84001011	0.343229396	0.999867943
tusE	-0.796520751	1.998926282	0.690280599	0.999867943
yeaC	-0.796633107	2.48728818	0.748754727	0.999867943
tolR	-0.796707049	2.532185344	0.753040927	0.999867943
nfo	-0.79691823	0.853125127	0.350243818	0.999867943
oppD	-0.797186687	0.974600986	0.413378818	0.999867943
rutR	-0.798293948	1.015695236	0.431892006	0.999867943
rlmG	-0.79847817	0.940462235	0.395866068	0.999867943
ydcP	-0.798519731	1.068957618	0.455058823	0.999867943
yebG	-0.798883886	1.979390438	0.686506178	0.999867943
yjbR	-0.79908689	2.560316578	0.754960939	0.999867943
smg	-0.800462022	2.015904835	0.691313395	0.999867943
rffG	-0.800583256	0.927362645	0.387977828	0.999867943
ppa	-0.801106908	1.978002403	0.685471589	0.999867943

ogt	-0.80217895	1.700398886	0.637098621	0.999867943
cca	-0.802933588	0.773349463	0.299151569	0.999867943
yidH	-0.803921788	2.404708541	0.738144492	0.999867943
cyaR	-0.804081164	2.97149532	0.786700112	0.999867943
selB	-0.804094479	0.834690778	0.335374843	0.999867943
cynR	-0.804668581	0.708668976	0.256180679	0.999867943
torC	-0.805091525	1.916898411	0.674487667	0.999867943
nhoA	-0.805404029	2.285261184	0.724512712	0.999867943
abgB	-0.80688483	0.937847324	0.389591505	0.999867943
insI1	-0.808127818	0.967729047	0.403674618	0.999867943
frvB	-0.808250552	0.793780033	0.30856872	0.999867943
ydgD	-0.809822046	1.285469226	0.528706579	0.999867943
rrfG	-0.811398923	2.769795432	0.769563861	0.999867943
coaE	-0.811695279	2.361372999	0.731042806	0.999867943
sra	-0.811871234	2.569892678	0.752065966	0.999867943
argT	-0.81467048	2.284309311	0.721363162	0.999867943
ybhF	-0.817290939	0.708165453	0.2484608	0.999867943
ydcY	-0.818391725	2.748095656	0.76585336	0.999867943
tmcA	-0.818974951	0.7638296	0.28363206	0.999867943
dinQ	-0.81954783	2.913825021	0.778509565	0.999867943
pgpA	-0.820267936	2.321208126	0.723803769	0.999867943
aroC	-0.820380032	1.068695106	0.442697243	0.999867943
lacA	-0.820842776	0.875166971	0.348281901	0.999867943
ynfA	-0.823113308	1.959471472	0.674435016	0.999867943
tyrR	-0.823871512	0.959031065	0.390303799	0.999867943
thrS	-0.823888709	0.978676874	0.399877879	0.999867943
tatD	-0.824494609	0.868360976	0.342374649	0.999867943
yceQ	-0.826148178	2.400471195	0.730725332	0.999867943
purP	-0.828617546	1.69437653	0.624813526	0.999867943
yeaV	-0.828695407	1.327249589	0.532384335	0.999867943
thyA	-0.828728198	1.818296828	0.648554172	0.999867943
aes	-0.832884058	1.802336852	0.643999983	0.999867943
yfgD	-0.833601922	2.451616952	0.733840506	0.999867943
cysD	-0.834168476	1.738529372	0.63136058	0.999867943
yeiW	-0.834786502	2.717141244	0.758668537	0.999867943
tusC	-0.83502021	2.477059223	0.736040438	0.999867943
stfQ	-0.835112043	2.294289493	0.715861047	0.999867943
purN	-0.835210691	1.270442675	0.510912784	0.999867943
paaB	-0.835292787	2.532498248	0.741528749	0.999867943
rpsP	-0.838118814	1.519594801	0.581262879	0.999867943
gfcA	-0.83908476	2.720723708	0.757774188	0.999867943
ybjS	-0.839151574	0.826538627	0.309981926	0.999867943
sbcC	-0.839458596	0.709826844	0.23695801	0.999867943
ypeB	-0.840843416	2.341448561	0.71951094	0.999867943
fdx	-0.842286146	2.504518576	0.736639668	0.999867943
gltW	-0.842392106	2.511862548	0.73734937	0.999867943
fabZ	-0.843060436	1.941547731	0.6641281	0.999867943
cysM	-0.846051586	0.821056888	0.302802518	0.999867943
leuC	-0.847807478	0.805109267	0.292325383	0.999867943

ybaP	-0.848079569	1.733458694	0.62467088	0.999867943
chbG	-0.84901283	2.301210301	0.712171054	0.999867943
mhpT	-0.849111497	0.918852795	0.355434463	0.999867943
ydiI	-0.84942431	2.645322028	0.748131343	0.999867943
trxB	-0.850344484	1.284287591	0.507898319	0.999867943
bioD	-0.855102116	2.360909016	0.717208634	0.999867943
mdtA	-0.857104477	0.964794453	0.374336168	0.999867943
yciC	-0.858025286	1.915611878	0.654216806	0.999867943
pspC	-0.858165384	2.461759483	0.727390839	0.999867943
pspF	-0.862006588	0.825229659	0.296223744	0.999867943
phoE	-0.863027642	1.065587546	0.417993181	0.999867943
tfaQ	-0.863877078	1.146934296	0.451326509	0.999867943
ycfL	-0.863991975	2.001188353	0.665930786	0.999867943
yedK	-0.864132437	2.316669467	0.709143713	0.999867943
iscX	-0.865435669	2.728799219	0.751130622	0.999867943
acuI	-0.866017053	1.173712281	0.460608999	0.999867943
ynfM	-0.867958665	0.737037816	0.2389437	0.999867943
hyaA	-0.868414935	0.873150751	0.319942439	0.999867943
rsuA	-0.869206346	1.769016647	0.623178915	0.999867943
ygcO	-0.870059442	3.056025772	0.775871758	0.999867943
yceB	-0.871285288	1.824899193	0.633046723	0.999867943
fklB	-0.873100183	1.916097758	0.648630332	0.999867943
rbsK	-0.87909194	1.009674634	0.38393516	0.999867943
alaA	-0.880196986	1.902598282	0.643630376	0.999867943
rpmE	-0.880502917	1.561929249	0.572939405	0.999867943
tdcF	-0.880926011	2.575009532	0.732271181	0.999867943
oppC	-0.881728019	2.251003741	0.695276655	0.999867943
yhbV	-0.884120763	1.074930302	0.410797367	0.999867943
asr	-0.884525314	2.394118946	0.711786711	0.999867943
rpsE	-0.886871993	1.248599893	0.477522346	0.999867943
lrp	-0.887781695	2.334331543	0.703711477	0.999867943
citE	-0.888176012	1.057384171	0.40092254	0.999867943
livJ	-0.888530217	1.687087311	0.598426089	0.999867943
ygdQ	-0.891525683	1.912090819	0.641031571	0.999867943
tfaR	-0.891671639	1.175535274	0.448137391	0.999867943
yeaP	-0.892177812	2.230775932	0.689200285	0.999867943
ygiN	-0.892738981	2.45812612	0.716471418	0.999867943
rplD	-0.893428644	1.360651767	0.511426477	0.999867943
bsmA	-0.893888684	1.382793603	0.517996509	0.999867943
accC	-0.895248357	0.871431777	0.304264912	0.999867943
chpB	-0.896398085	2.415430078	0.710553189	0.999867943
ldrA	-0.896625952	3.721426249	0.809604637	0.999867943
ybiS	-0.896933934	2.301506416	0.696746545	0.999867943
treR	-0.896979956	1.90783497	0.638243606	0.999867943
leuB	-0.897305918	0.889921311	0.313311402	0.999867943
nth	-0.898710645	2.308728273	0.697078992	0.999867943
nlpC	-0.899453279	2.378097545	0.705264288	0.999867943
lplA	-0.899880983	0.930721989	0.333612348	0.999867943
cutC	-0.90023777	0.780219415	0.248571342	0.999867943

rnhA	-0.900600457	1.919840779	0.638996987	0.999867943
tesA	-0.900704584	1.078935947	0.403825704	0.999867943
murJ	-0.900729714	0.824929092	0.274882579	0.999867943
lepA	-0.901334365	0.888627185	0.310439726	0.999867943
ybiX	-0.902077644	2.353201696	0.701467273	0.999867943
gsiD	-0.902113834	1.017521211	0.37530513	0.999867943
ldrC	-0.90288174	3.368246088	0.78865549	0.999867943
yqjE	-0.903714242	2.422000051	0.709053731	0.999867943
flgN	-0.905195436	2.399934881	0.706043558	0.999867943
ykfA	-0.910415771	0.738570188	0.217697667	0.999867943
lpxK	-0.912102872	0.892662275	0.306885879	0.999867943
ygbK	-0.912772015	1.918098867	0.634164653	0.999867943
rph	-0.913390935	1.932497701	0.636464419	0.999867943
relA	-0.914089378	1.035321709	0.377288299	0.999867943
qseB	-0.914112127	1.937610894	0.637088964	0.999867943
narP	-0.915161395	2.440066982	0.707618931	0.999867943
narV	-0.916710077	2.379578417	0.700059209	0.999867943
araF	-0.919034527	2.229586462	0.68019317	0.999867943
selD	-0.921454862	0.79081249	0.24393797	0.999867943
yajC	-0.921801771	2.343058806	0.694010695	0.999867943
wcaA	-0.923445289	1.916897523	0.629990999	0.999867943
mdfA	-0.92447283	1.680202636	0.582171861	0.999867943
yeaE	-0.925644981	1.912971885	0.628472465	0.999867943
ascF	-0.92677896	0.870532789	0.287051908	0.999867943
eutB	-0.927602905	0.730262117	0.204001711	0.999867943
bglA	-0.927853051	1.73129094	0.592006013	0.999867943
acpP	-0.930565735	1.483808163	0.530562926	0.999867943
cspC	-0.930973207	2.597080791	0.71999229	0.999867943
purF	-0.931177764	0.978458621	0.341260196	0.999867943
tilS	-0.931384255	1.046614398	0.373518505	0.999867943
ssrA	-0.937104999	1.543834851	0.543852097	0.999867943
atoA	-0.938445852	1.082325969	0.38590696	0.999867943
ftsB	-0.938870585	2.403404668	0.696061944	0.999867943
ybhH	-0.938949717	1.913733305	0.623682713	0.999867943
znuC	-0.939020317	2.25244821	0.676759783	0.999867943
ypdF	-0.940053032	0.998050741	0.346249463	0.999867943
smrB	-0.940326356	2.31105523	0.68409479	0.999867943
ygiC	-0.940794713	1.743355637	0.589441259	0.999867943
pabA	-0.941899513	1.93351832	0.626157143	0.999867943
lpxP	-0.943389403	2.217662809	0.670546538	0.999867943
yciY	-0.943417873	2.833387917	0.739161005	0.999867943
csrA	-0.946503787	2.482995468	0.703059058	0.999867943
gltU	-0.947818424	2.896327726	0.743480066	0.999867943
ftsE	-0.94816972	0.892700538	0.288173775	0.999867943
prfB	-0.949674199	1.079779638	0.379126214	0.999867943
tsaA	-0.952158783	1.954882931	0.626210917	0.999867943
insA	-0.954394391	1.226165177	0.436358513	0.999867943
ybjH	-0.955321157	2.708242505	0.724279023	0.999867943
yhbU	-0.956741884	1.065090644	0.369040165	0.999867943

fliZ	-0.958566274	1.930728432	0.619556444	0.999867943
tqsA	-0.9593829	1.730374178	0.579280035	0.999867943
yobF	-0.960849497	2.699653712	0.721903599	0.999867943
insB1	-0.960895873	1.166169586	0.409953135	0.999867943
rbn	-0.960932485	1.793722491	0.592152283	0.999867943
yhjD	-0.961164814	1.802174929	0.593800994	0.999867943
gutM	-0.963110929	2.064410033	0.640835584	0.999867943
kdpC	-0.963367308	1.10962041	0.385287386	0.999867943
plsX	-0.964434744	1.154860008	0.40365597	0.999867943
ybaN	-0.967316204	2.375307449	0.683832963	0.999867943
insE1	-0.967346799	1.031197291	0.348202679	0.999867943
torR	-0.967381258	1.798229416	0.590602452	0.999867943
rnk	-0.968305396	1.491873124	0.51630383	0.999867943
yciO	-0.96938706	2.305420922	0.674133674	0.999867943
ytfF	-0.969992484	1.109508505	0.381979829	0.999867943
ynfH	-0.972291864	1.907778004	0.610299331	0.999867943
nikB	-0.972859833	1.016506109	0.338535732	0.999867943
hisH	-0.973284199	1.102580131	0.377380385	0.999867943
rbsA	-0.97378718	0.954000073	0.307377069	0.999867943
yobA	-0.975001611	0.914847025	0.286534991	0.999867943
yjjJ	-0.975241224	1.89873707	0.607513315	0.999867943
mdlA	-0.977525107	1.149425369	0.395076735	0.999867943
acrD	-0.982746425	1.001216558	0.326320452	0.999867943
purR	-0.982903944	1.147314051	0.391610705	0.999867943
metW	-0.982949991	2.465094693	0.69007937	0.999867943
icd	-0.983424246	1.088668443	0.366352055	0.999867943
waaM	-0.983454868	0.988200248	0.319639995	0.999867943
tpiA	-0.984204022	2.311089647	0.670208796	0.999867943
ves	-0.985103804	2.305476758	0.669169177	0.999867943
sgcE	-0.985711988	1.133126685	0.384352603	0.999867943
nagE	-0.985889762	1.065146251	0.354658583	0.999867943
rhtC	-0.988495089	1.901312194	0.60313222	0.999867943
mnmE	-0.989004135	0.835477747	0.236508556	0.999867943
ftsL	-0.989020911	2.402680432	0.680608941	0.999867943
ytjB	-0.98956747	1.95559451	0.612843494	0.999867943
ydfG	-0.99076369	2.31161042	0.668211995	0.999867943
lacZ	-0.992064432	0.978794559	0.310794012	0.999867943
ybaY	-0.992195834	2.363558296	0.674639583	0.999867943
trpE	-0.992393794	1.107530838	0.370230502	0.999867943
ydaN	-0.992985427	1.904459922	0.602088143	0.999867943
insA	-0.993833058	2.36498079	0.674318312	0.999867943
cdaR	-0.995879267	1.788933791	0.577740123	0.999867943
gltV	-0.997284483	4.382391552	0.819983447	0.999867943
entS	-0.997663466	1.096659669	0.362965272	0.999867943
rplN	-0.998012545	1.525642032	0.51300934	0.999867943
citD	-1.000464264	3.063120222	0.743958294	0.999867943
fryB	-1.000476065	2.442892302	0.682139228	0.999867943
yeaK	-1.001674283	1.262904117	0.427689566	0.999867943
yebF	-1.002746172	1.940795095	0.605388135	0.999867943

araG	-1.003132536	1.032703372	0.33136625	0.999867943
tus	-1.004190619	2.285856022	0.660439715	0.999867943
yebS	-1.004581747	1.897151551	0.596444022	0.999867943
yqiB	-1.005065613	2.315078141	0.664187536	0.999867943
insH1	-1.006327449	0.906589993	0.266993058	0.999867943
rpoZ	-1.010359414	2.431331649	0.677733436	0.999867943
trmD	-1.010937851	1.243493009	0.416228186	0.999867943
rsmF	-1.011008367	2.212387754	0.647688212	0.999867943
trmL	-1.014430775	1.820734452	0.577421731	0.999867943
yfaT	-1.016237978	2.328609396	0.662536309	0.999867943
yfeZ	-1.016664912	1.049470327	0.332674441	0.999867943
yecS	-1.01710074	2.437433216	0.676471033	0.999867943
mhpB	-1.018937121	0.966727115	0.291879724	0.999867943
pitA	-1.019567618	1.190981478	0.391957155	0.999867943
yggU	-1.020667655	2.345047091	0.663385334	0.999867943
ygcN	-1.026778889	1.035352452	0.32133453	0.999867943
edd	-1.026786021	1.101366905	0.351190113	0.999867943
bioB	-1.028435878	0.925948836	0.266704708	0.999867943
yphE	-1.031543911	0.87905117	0.240605678	0.999867943
ydhO	-1.033405418	0.980646891	0.291974622	0.999867943
aat	-1.03460159	1.795280452	0.5644194	0.999867943
yeiH	-1.034620616	2.243575044	0.644692275	0.999867943
pncA	-1.038111226	1.534972913	0.498846452	0.999867943
pspD	-1.038526441	2.236743182	0.642430716	0.999867943
ygiD	-1.038752626	1.718028029	0.545432088	0.999867943
pcm	-1.039561271	2.358554345	0.659385263	0.999867943
rseB	-1.043385413	1.079366202	0.333711583	0.999867943
pyrE	-1.04341069	1.911441787	0.585150999	0.999867943
hypB	-1.044254478	0.92837708	0.260666518	0.999867943
lsrB	-1.045185613	1.911945168	0.58461174	0.999867943
mhpD	-1.045841998	0.827780455	0.206434976	0.999867943
pgk	-1.046187679	1.023259123	0.306588129	0.999867943
smpB	-1.046222712	2.315442552	0.65138016	0.999867943
yjfP	-1.047177265	2.314948318	0.651013627	0.999867943
ychQ	-1.047750311	2.474929663	0.672043209	0.999867943
yfeW	-1.04885514	2.227238941	0.637696654	0.999867943
yhbJ	-1.049444332	2.260209244	0.642423576	0.999867943
mdtI	-1.052317322	2.010861561	0.600753921	0.999867943
rluD	-1.052826403	1.910264977	0.581536643	0.999867943
rbsB	-1.053958905	1.267651106	0.405732652	0.999867943
narX	-1.055947842	0.863855175	0.221568967	0.999867943
bssR	-1.059135328	2.448072964	0.665276016	0.999867943
flgC	-1.059768341	2.517272616	0.673756077	0.999867943
flgB	-1.060173399	2.587170988	0.681966638	0.999867943
yfiC	-1.060566294	1.784938693	0.552394942	0.999867943
yggT	-1.062696722	2.307371661	0.645110076	0.999867943
malZ	-1.06474508	1.006716169	0.290218787	0.999867943
cysC	-1.066485438	2.396650773	0.656327038	0.999867943
ribC	-1.067208728	2.28495899	0.640458272	0.999867943

yohD	-1.06766826	0.970848922	0.271451317	0.999867943
ldcA	-1.069108341	2.263206021	0.636650822	0.999867943
mnaT	-1.071339806	1.945050104	0.581768596	0.999867943
ptsH	-1.075140032	2.367300615	0.649711608	0.999867943
lsrC	-1.075747302	1.037326424	0.299717976	0.999867943
ycbX	-1.077676793	1.729352871	0.533174347	0.999867943
kdsB	-1.077795567	1.28431739	0.401358654	0.999867943
ygfS	-1.07840456	2.449900397	0.659804556	0.999867943
entH	-1.081282239	2.426139936	0.655828356	0.999867943
yfiF	-1.082943438	0.880887054	0.218929998	0.999867943
ydiQ	-1.085557988	1.929176998	0.573635622	0.999867943
mpaA	-1.086441163	1.902126909	0.567883223	0.999867943
yahC	-1.086743871	0.905650354	0.230155016	0.999867943
paaD	-1.086861753	2.556530753	0.670740816	0.999867943
seqA	-1.087583457	2.35004731	0.643513356	0.999867943
ybbA	-1.090765188	1.031528791	0.290317288	0.999867943
mfd	-1.091696917	0.764432253	0.153258855	0.999867943
rpsD	-1.094250303	1.387525195	0.430325634	0.999867943
btuC	-1.094250602	0.99870223	0.273222107	0.999867943
dnaA	-1.094818818	1.185513332	0.355747906	0.999867943
lptE	-1.095003818	1.851647077	0.554274242	0.999867943
ydjN	-1.096284627	1.889906772	0.561865128	0.999867943
dmlR	-1.09695042	1.790507024	0.54010916	0.999867943
eutS	-1.097904158	2.474819593	0.657310129	0.999867943
arcA	-1.103001339	1.981448821	0.577756979	0.999867943
rstB	-1.1052607	1.887585498	0.558183294	0.999867943
rlmE	-1.105574671	2.283793776	0.628317976	0.999867943
ybcF	-1.10797982	0.915522623	0.226196149	0.999867943
ycjP	-1.110761008	2.280414512	0.626196447	0.999867943
cdd	-1.113495709	1.265173603	0.378798109	0.999867943
yfaE	-1.116521501	2.851709745	0.695407699	0.999867943
thiB	-1.117871655	1.791143243	0.53255489	0.999867943
slyD	-1.118853659	1.091681981	0.305415217	0.999867943
aaeA	-1.119562123	1.903006403	0.556322674	0.999867943
aroL	-1.120114023	2.260335582	0.620210462	0.999867943
glcG	-1.121341477	1.649350965	0.49658784	0.999867943
tatA	-1.121958035	2.037745522	0.581916203	0.999867943
hisQ	-1.124777479	1.463959692	0.442301977	0.999867943
ddpF	-1.127439741	1.454440892	0.43823889	0.999867943
metB	-1.128143242	1.889431186	0.55045338	0.999867943
panB	-1.128855917	2.306416416	0.624529159	0.999867943
gadA	-1.132818933	1.900555743	0.551144397	0.999867943
tusD	-1.135217792	2.464818757	0.64510824	0.999867943
mhpE	-1.140032547	0.7945991	0.151364992	0.999867943
argV	-1.140531602	2.439245747	0.640088173	0.999867943
ydgC	-1.140555592	2.367188236	0.629934734	0.999867943
eutT	-1.141686131	1.41416667	0.419481698	0.999867943
ldrB	-1.146179916	3.489445006	0.742555909	0.999867943
fliM	-1.147560464	1.091203846	0.292961867	0.999867943

yfcE	-1.147795854	2.327190386	0.621863519	0.999867943
yeaL	-1.158798837	1.945975309	0.551519416	0.999867943
frwC	-1.158812594	1.775910996	0.514067536	0.999867943
ypjK	-1.159373811	2.653607736	0.662180431	0.999867943
ygdB	-1.159815841	2.407369687	0.629964224	0.999867943
tff	-1.160301009	2.88061765	0.68709837	0.999867943
purK	-1.161582341	1.019782275	0.25468257	0.999867943
fkpB	-1.162887786	2.350499753	0.6207832	0.999867943
gfcE	-1.164056207	1.784926423	0.51429847	0.999867943
infA	-1.164930891	2.424203749	0.630842288	0.999867943
uxaB	-1.166982172	0.861763248	0.1756791	0.999867943
abgA	-1.168612612	1.783158077	0.512235228	0.999867943
sapB	-1.171315509	1.714527824	0.494498861	0.999867943
uidB	-1.175304539	1.780722489	0.509243848	0.999867943
glnB	-1.176060732	2.429831385	0.628379344	0.999867943
aroH	-1.176375629	1.897634501	0.535312522	0.999867943
rstA	-1.176760297	2.268434095	0.603931981	0.999867943
ydhC	-1.178089973	1.891971733	0.533495865	0.999867943
yliI	-1.180811989	2.21258815	0.593563591	0.999867943
rpmG	-1.182894392	2.672444479	0.65803601	0.999867943
sseA	-1.183022891	2.222496845	0.594522027	0.999867943
gltI	-1.183767053	1.252373008	0.344546963	0.999867943
nirD	-1.184762236	2.209070436	0.591739419	0.999867943
ydhT	-1.188386545	1.714823146	0.48830439	0.999867943
creB	-1.189366206	2.321610019	0.60843944	0.999867943
ycfJ	-1.189376379	1.93399537	0.538565229	0.999867943
lptG	-1.191842074	1.248771086	0.339875149	0.999867943
aspA	-1.195866956	1.277625461	0.349269349	0.999867943
gsiA	-1.196281807	1.078454783	0.267320013	0.999867943
rnt	-1.197021959	1.126872132	0.288121387	0.999867943
btuF	-1.206278455	2.249229551	0.591746116	0.999867943
flhD	-1.207460029	2.374326037	0.611068711	0.999867943
potD	-1.210845488	1.879693452	0.519464044	0.999867943
yfgI	-1.216736645	2.304865236	0.597569261	0.999867943
ddpC	-1.217051895	2.255721264	0.589514291	0.999867943
potI	-1.220190632	1.025117443	0.233931054	0.999867943
yecN	-1.220338064	2.499568454	0.625394373	0.999867943
insB1	-1.221073427	1.167906014	0.295780989	0.999867943
ybeL	-1.222077297	2.292932822	0.594050456	0.999867943
bssS	-1.223973987	2.10994502	0.56184882	0.999867943
grcA	-1.225306523	2.424151279	0.613237229	0.999867943
ycjR	-1.225926068	1.967963485	0.533322984	0.999867943
rcsB	-1.227303867	1.493377966	0.411173256	0.999867943
ybaV	-1.230961416	2.456619283	0.616315215	0.999867943
rpoS	-1.232241817	1.052826044	0.241834605	0.999867943
sspA	-1.232368199	1.318519724	0.349963331	0.999867943
ycbL	-1.234817696	1.115020439	0.268103949	0.999867943
argB	-1.23934717	2.272080629	0.585431716	0.999867943
lspA	-1.239883698	2.429624424	0.609827939	0.999867943

rhaB	-1.242088941	2.231326342	0.577760113	0.999867943
rpmJ	-1.242924679	1.944635792	0.522721772	0.999867943
dsbE	-1.243180507	2.386287573	0.602388759	0.999867943
phnO	-1.243227611	1.467635855	0.396941938	0.999867943
guaB	-1.2454434	1.272607482	0.327750581	0.999867943
ytfH	-1.248094727	1.988235436	0.530174338	0.999867943
rttR	-1.248688369	2.520491129	0.620307417	0.999867943
rplQ	-1.252975913	1.410150483	0.374250043	0.999867943
dctA	-1.255503543	1.31587654	0.340023127	0.999867943
ycdX	-1.256893449	2.238694711	0.574497412	0.999867943
flhC	-1.260793003	2.289523141	0.581853536	0.999867943
fruK	-1.262278635	2.227223621	0.570884153	0.999867943
rplP	-1.262869707	1.240255134	0.308566856	0.999867943
yfcH	-1.268686616	0.874685153	0.146933221	0.999867943
gph	-1.272469203	2.312092286	0.582076603	0.999867943
yafD	-1.274090824	2.234317894	0.568516936	0.999867943
yebE	-1.274117907	2.270051505	0.574611658	0.999867943
mnmA	-1.277159433	2.213359775	0.563923938	0.999867943
rplY	-1.277785974	1.553357553	0.410737677	0.999867943
recO	-1.280191821	1.787009533	0.473751928	0.999867943
fepC	-1.280216947	2.273608623	0.573382385	0.999867943
ybdZ	-1.280597663	3.179959966	0.687162505	0.999867943
pnp	-1.281416998	0.944970309	0.175086623	0.999867943
fepG	-1.28199845	1.292357246	0.321205051	0.999867943
srlB	-1.282409428	2.594136138	0.621059501	0.999867943
ulaF	-1.284655647	1.91959326	0.503346696	0.999867943
xthA	-1.285787641	2.274963116	0.571944168	0.999867943
rluE	-1.286686333	1.904422594	0.499275157	0.999867943
lpxC	-1.286829712	1.320997986	0.329989762	0.999867943
hybE	-1.295433255	1.833482696	0.479850898	0.999867943
hybA	-1.297588026	1.008105946	0.19804047	0.999867943
yebA	-1.30020866	1.707281382	0.446318652	0.999867943
rimK	-1.300300804	1.779062056	0.46484561	0.999867943
yfeY	-1.300848581	2.383185737	0.585172902	0.999867943
fadR	-1.301045631	2.241156876	0.561561224	0.999867943
yneG	-1.305603596	1.995059233	0.512842243	0.999867943
lplT	-1.307739213	1.763509315	0.458356975	0.999867943
iscS	-1.307812285	1.086229877	0.228592598	0.999867943
lolA	-1.311084784	2.273332535	0.56412611	0.999867943
ydgI	-1.314588691	1.569779607	0.40234799	0.999867943
hypD	-1.316001307	2.2604605	0.560443533	0.999867943
pykA	-1.316045853	2.19508874	0.548812131	0.999867943
mntS	-1.317572217	2.849963176	0.643857585	0.999867943
glnP	-1.320356416	2.274452221	0.561566549	0.999867943
iscU	-1.322822463	2.331437811	0.570452749	0.999867943
hcaT	-1.329365745	0.949263929	0.161388356	0.999867943
cspG	-1.330541489	2.451182429	0.58725637	0.999867943
wzb	-1.331096819	2.5467528	0.601207872	0.999867943
ribE	-1.334855794	2.35143228	0.5702538	0.999867943

ygaH	-1.335689367	2.541293415	0.599170109	0.999867943
frdC	-1.336073358	2.314883204	0.563826891	0.999867943
yjjG	-1.341883825	2.271404965	0.554672794	0.999867943
murA	-1.346674725	1.375512719	0.327562819	0.999867943
fabF	-1.347431525	0.941849687	0.152538383	0.999867943
cmoB	-1.350944157	1.761466383	0.443115459	0.999867943
flgI	-1.35132741	2.273385049	0.552236568	0.999867943
ydhJ	-1.352528602	1.698105922	0.425745843	0.999867943
lolC	-1.352572772	1.28681817	0.293213309	0.999867943
hokB	-1.359679528	4.114258964	0.741037444	0.999867943
yejF	-1.361622554	2.187622085	0.533664887	0.999867943
ydhB	-1.36376434	1.794634495	0.447307171	0.999867943
hisG	-1.364352742	1.18665826	0.25024949	0.999867943
mmuP	-1.366742099	0.803565405	0.088971653	0.999867943
smrA	-1.368853456	1.374391069	0.319264304	0.999867943
rrfD	-1.369291942	2.948592834	0.642369588	0.999867943
sdhC	-1.372772495	2.348111247	0.558797615	0.999867943
flgH	-1.375892871	2.371195702	0.561744173	0.999867943
hscB	-1.38252374	1.956940563	0.479894651	0.999867943
ccmE	-1.387685798	2.319347288	0.549633933	0.999867943
iscA	-1.391482402	2.380245721	0.55881932	0.999867943
secE	-1.392197859	1.956320722	0.476687167	0.999867943
ispH	-1.394539825	1.879527224	0.458109699	0.999867943
baeR	-1.395260033	2.314497277	0.546618441	0.999867943
insI1	-1.39549952	1.169071737	0.232602508	0.999867943
yccA	-1.4015563	1.302938614	0.282066518	0.999867943
pfkB	-1.4044154	2.235752134	0.529897566	0.999867943
yneJ	-1.409750938	1.88458119	0.454433207	0.999867943
rcsF	-1.409915513	1.928524011	0.464727066	0.999867943
yciS	-1.410236421	1.893546445	0.456417213	0.999867943
cra	-1.410953049	1.755625285	0.4215844	0.999867943
ybhP	-1.412399673	1.918633777	0.461640236	0.999867943
flgM	-1.414140975	2.667795621	0.596057538	0.999867943
insH1	-1.417366108	1.22682247	0.247961554	0.999867943
btuD	-1.417374987	2.355476814	0.547349942	0.999867943
nudG	-1.417497792	2.469932781	0.56603455	0.999867943
insD1	-1.419338721	1.089881141	0.192818081	0.999867943
serS	-1.421345497	2.212818538	0.520663115	0.999867943
ydiS	-1.422180845	1.111163069	0.200579284	0.999867943
rutD	-1.424353991	1.219762843	0.242915584	0.999867943
tadA	-1.426903138	1.793842118	0.426354565	0.999867943
narW	-1.436290023	1.963979273	0.464585987	0.999867943
mlaC	-1.436768416	2.264903142	0.525844623	0.999867943
pdxY	-1.437868158	1.761090099	0.414234321	0.999867943
yraN	-1.438520147	2.07139861	0.487388733	0.999867943
wcaB	-1.439174817	2.430001379	0.553681401	0.999867943
mlaD	-1.442891122	1.946281835	0.458476555	0.999867943
insH1	-1.445808799	1.217815935	0.235143003	0.999867943
surE	-1.446930531	1.199271066	0.227621549	0.999867943

yohK	-1.450489445	2.307631477	0.529635848	0.999867943
ydhP	-1.453433477	1.859938178	0.434542682	0.999867943
sdhD	-1.454165901	2.342928863	0.534822321	0.999867943
ypfG	-1.454272567	1.682766849	0.387469724	0.999867943
modC	-1.459203576	1.756339191	0.406074728	0.999867943
rbfA	-1.461783254	2.313486091	0.527482902	0.999867943
infC	-1.464390148	2.077593844	0.48090415	0.999867943
rplS	-1.464802378	2.377827855	0.53787775	0.999867943
fryC	-1.465201602	1.883218328	0.436550937	0.999867943
hyfH	-1.465772908	1.943188047	0.450661019	0.999867943
sufC	-1.468762413	1.917786179	0.443757533	0.999867943
dusB	-1.471355071	1.203360897	0.221441237	0.999867943
pgsA	-1.480939096	2.260478602	0.512375016	0.999867943
pyrC	-1.487292934	1.758394077	0.39765061	0.999867943
rpsA	-1.493189981	1.21513646	0.219137451	0.999867943
ribF	-1.503618312	2.223641938	0.498916061	0.999867943
ycdY	-1.503651481	2.419390852	0.534270677	0.999867943
rpsF	-1.514373631	1.462826195	0.300557659	0.999867943
rpsL	-1.515668447	1.594704816	0.341889619	0.999867943
skp	-1.517879855	2.358464596	0.519842539	0.999867943
moaC	-1.518408838	2.582749101	0.556596651	0.999867943
thiK	-1.524968676	1.873634352	0.415696836	0.999867943
ybeZ	-1.526770214	2.210042438	0.489670504	0.999867943
pdxB	-1.529348053	1.855235751	0.409745474	0.999867943
insB1	-1.53027411	1.684164234	0.363547986	0.999867943
nusB	-1.542223312	2.475386543	0.533269259	0.999867943
bamB	-1.543412591	1.747068197	0.377003946	0.999867943
pncC	-1.550136902	1.952282135	0.42718801	0.999867943
galK	-1.553167966	1.85655769	0.402825986	0.999867943
hcaB	-1.55742558	1.080978913	0.149653961	0.999867943
yebZ	-1.558625124	1.662034373	0.348356699	0.999867943
fliP	-1.564308614	2.313331192	0.498904325	0.999867943
nuoA	-1.565189563	1.251762947	0.211157789	0.999867943
yehX	-1.567186761	1.749209382	0.370284776	0.999867943
rplF	-1.569423401	1.481963658	0.289592439	0.999867943
fdnH	-1.573568319	1.785154244	0.378060937	0.999867943
hisI	-1.585457706	1.88441409	0.400150131	0.999867943
add	-1.588110128	1.852737909	0.391351305	0.999867943
nuoJ	-1.59069751	1.784145243	0.372621312	0.999867943
psrD	-1.593983081	2.600653376	0.539931625	0.999867943
yfcZ	-1.602080944	1.262560925	0.20447182	0.999867943
recR	-1.611613529	1.764229577	0.36098267	0.999867943
yfiM	-1.616833643	2.340884658	0.489758222	0.999867943
hcr	-1.636096483	1.861406546	0.379424542	0.999867943
cydA	-1.638126747	1.138459257	0.150179425	0.999867943
ptsN	-1.640419963	1.900368873	0.38802128	0.999867943
insD1	-1.640800469	1.047095587	0.117114315	0.999867943
fliN	-1.645563825	1.856466781	0.375404436	0.999867943
deoR	-1.649403382	2.204918326	0.454426107	0.999867943

yjjX	-1.654569971	2.271700214	0.466405526	0.999867943
crr	-1.660271943	2.286193166	0.467705859	0.999867943
ychH	-1.661247099	1.545061166	0.282285968	0.999867943
ccmD	-1.664728939	2.667425612	0.532564717	0.999867943
ycjU	-1.666821285	2.363672889	0.480696532	0.999867943
yfiH	-1.678446378	1.749818385	0.337452076	0.999867943
napD	-1.690145959	2.91842201	0.562501408	0.999867943
insE1	-1.694211593	2.140813892	0.428718382	0.999867943
hybB	-1.695168085	1.89737017	0.371626759	0.999867943
kdpE	-1.703926589	2.293650498	0.457549186	0.999867943
ygfM	-1.704965092	2.261471694	0.450898229	0.999867943
ymgG	-1.705569756	2.480880884	0.491776804	0.999867943
ycbK	-1.705947434	1.105763441	0.122884666	0.999867943
udp	-1.707934879	1.388346692	0.218624707	0.999867943
yhbQ	-1.713946051	2.510607375	0.49480791	0.999867943
insD1	-1.724761212	1.153233907	0.13476141	0.999867943
wcaL	-1.731019437	1.419898048	0.222800433	0.999867943
yjjW	-1.737271903	2.28615976	0.447309367	0.999867943
wcaG	-1.742356305	2.297195548	0.448169041	0.999867943
cspB	-1.758804163	2.361865767	0.456472882	0.999867943
exuT	-1.761851216	1.132571501	0.119798409	0.999867943
cptA	-1.764524664	2.317176945	0.446359917	0.999867943
yhbP	-1.771227787	2.316099819	0.444422882	0.999867943
ppdC	-1.7789275	2.523947804	0.480922604	0.999867943
rlmH	-1.780701576	1.909845496	0.351140222	0.999867943
hyaE	-1.783163635	2.593246699	0.491693242	0.999867943
hybD	-1.784586155	2.289099299	0.435625239	0.999867943
ybcJ	-1.792285516	2.746124743	0.513975556	0.999867943
rpsO	-1.795957681	1.800510962	0.318535886	0.999867943
aspS	-1.803886353	2.15043124	0.401554244	0.999867943
rpmH	-1.81595926	2.609983833	0.486570286	0.999867943
dusC	-1.827279633	1.638822144	0.264852267	0.999867943
rpsM	-1.835683858	1.515084457	0.225663631	0.999867943
paaF	-1.854066489	2.331308657	0.426444822	0.999867943
rsxB	-1.855276122	1.897593574	0.328223013	0.999867943
ligT	-1.859114481	1.764599945	0.292083745	0.999867943
nrdI	-1.88084444	2.731338304	0.491064677	0.999867943
ylbF	-1.885050416	1.638206753	0.249864242	0.999867943
murG	-1.910636361	1.819908142	0.293785618	0.999867943
yneM	-1.91086946	2.561536955	0.455676166	0.999867943
rpsU	-1.914384981	2.478423427	0.439865436	0.999867943
nudB	-1.916366907	2.416323792	0.427724262	0.999867943
rplB	-1.927689976	1.403236928	0.169520803	0.999867943
ruvC	-1.929400255	2.383590313	0.41825561	0.999867943
purE	-1.929978766	2.382781779	0.41795814	0.999867943
hypC	-1.939751387	3.074779699	0.528132947	0.999867943
yceD	-1.9578464	2.094941778	0.350015689	0.999867943
rpsK	-1.966140053	1.344235685	0.143564451	0.999867943
ilvY	-1.968167328	1.708325534	0.249278665	0.999867943

entC	-1.977294971	1.704195895	0.245946633	0.999867943
ccmB	-1.979226101	2.371864152	0.404021738	0.999867943
thiG	-1.980082449	1.887138848	0.294062581	0.999867943
ssuD	-1.995569163	1.549112815	0.197675895	0.999867943
yedJ	-1.997602967	2.282203504	0.38141302	0.999867943
csrB	-1.999892836	1.379611609	0.147168509	0.999867943
ycjQ	-2.000703361	1.734690996	0.248767346	0.999867943
thiQ	-2.010276722	1.432804197	0.160605927	0.999867943
napB	-2.019735754	2.527504615	0.424230866	0.999867943
hisC	-2.020724947	1.832453792	0.27013894	0.999867943
rimM	-2.03873685	2.336646513	0.382932689	0.999867943
frdD	-2.057394256	2.314168938	0.373980305	0.999867943
nrfD	-2.063234822	1.818117674	0.256451085	0.999867943
vsr	-2.06341002	2.011733094	0.305038784	0.999867943
cysU	-2.085833221	1.859504464	0.261983826	0.999867943
cspA	-2.087598663	1.809156147	0.248538136	0.999867943
rutE	-2.089864176	2.366150688	0.377109983	0.999867943
rpmD	-2.093123246	2.798638898	0.454515905	0.999867943
yhbS	-2.100442672	1.627307554	0.196791351	0.999867943
phnK	-2.13354282	1.761125334	0.225716998	0.999867943
hybF	-2.143925216	2.650956701	0.418666778	0.999867943
hyaD	-2.148527771	2.424189806	0.375462924	0.999867943
cyoC	-2.183333709	1.912005925	0.253492514	0.999867943
rplV	-2.188027999	2.309997598	0.343537256	0.999867943
ugpQ	-2.219035142	1.845719787	0.229262794	0.999867943
cydB	-2.222919523	1.128313949	0.048824046	0.999867943
ypdE	-2.227670118	1.763356267	0.206476874	0.999867943
moaD	-2.240788119	2.850593064	0.431821869	0.999867943
rpsT	-2.244635763	2.499697849	0.369205364	0.999867943
eutQ	-2.251483267	2.268523036	0.320959235	0.999867943
nohD	-2.259115632	1.805414291	0.210824898	0.999867943
rplI	-2.279232331	2.273831903	0.316162495	0.999867943
entA	-2.310945546	1.881322976	0.219311127	0.999867943
bioC	-2.402673391	1.937663894	0.21498109	0.999867943
menD	-2.42159326	1.929036301	0.209355956	0.999867943
rplW	-2.46677392	2.38536979	0.301077067	0.999867943
tdcD	-2.543290237	1.600638143	0.112077852	0.999867943
tauC	-2.545084969	1.806192684	0.158809061	0.999867943
ydfK	-2.557871301	2.759824421	0.354017961	0.999867943
rpmA	-2.590450759	2.343969575	0.269092235	0.999867943
hflD	-2.603316557	1.830434313	0.154956644	0.999867943
eutG	-2.825725117	1.50883177	0.061097383	0.999867943
puuE	-2.916486509	1.679792502	0.08252524	0.999867943
rplO	-2.963760543	2.328968719	0.203172984	0.999867943
yiaQ	-3.053409541	2.322089249	0.18852985	0.999867943
napG	-3.06173146	2.346209428	0.191903275	0.999867943
rutF	-3.101129296	1.676959481	0.064420691	0.999867943
phnH	-3.470572916	2.050997334	0.090619443	0.999867943
nikC	-3.511840413	1.610137756	0.029177447	0.999867943

insC1	-3.658927426	2.197675676	0.095931147	0.999867943
insC1	-3.700418678	2.197302516	0.092167488	0.999867943
insC1	-3.787168169	2.21336214	0.08707231	0.999867943
rpmC	-4.037289746	2.807163246	0.15037453	0.999867943
phnI	-4.074845184	1.817550807	0.024965061	0.999867943

Table S4. GO Functional Enrichment analysis for up-regulated genes in ampicillin-resistant cells

**Biological processes**	***Escherichia coli* - Reflist (4392)**	**Gene list (306)**	**Expected**	**Fold Enrichment**	**P-value**
threonine catabolic process (GO:0006567)	14	8	0.98	8.2	4.82E-02
regulation of developmental process (GO:0050793)	48	16	3.34	4.78	2.26E-03
regulation of cell morphogenesis (GO:0022604)	48	16	3.34	4.78	2.26E-03
regulation of anatomical structure morphogenesis (GO:0022603)	48	16	3.34	4.78	2.26E-03
regulation of cell shape (GO:0008360)	48	16	3.34	4.78	2.26E-03
glycosaminoglycan biosynthetic process (GO:0006024)	46	14	3.2	4.37	2.36E-02
aminoglycan biosynthetic process (GO:0006023)	46	14	3.2	4.37	2.36E-02
peptidoglycan biosynthetic process (GO:0009252)	46	14	3.2	4.37	2.36E-02
cell wall macromolecule biosynthetic process (GO:0044038)	47	14	3.27	4.28	2.88E-02
cellular component macromolecule biosynthetic process (GO:0070589)	47	14	3.27	4.28	2.88E-02
peptidoglycan-based cell wall biogenesis (GO:0009273)	49	14	3.41	4.1	4.25E-02
peptidoglycan metabolic process (GO:0000270)	76	18	5.3	3.4	2.95E-02
glycosaminoglycan metabolic process (GO:0030203)	76	18	5.3	3.4	2.95E-02
aminoglycan metabolic process (GO:0006022)	78	18	5.43	3.31	3.98E-02
external encapsulating structure organization (GO:0045229)	102	22	7.11	3.1	1.35E-02
cell division (GO:0051301)	93	20	6.48	3.09	3.53E-02
cellular component biogenesis (GO:0044085)	316	49	22.02	2.23	7.43E-04
cellular amino acid metabolic process (GO:0006520)	259	40	18.05	2.22	9.44E-03
organonitrogen compound biosynthetic process (GO:1901566)	520	75	36.23	2.07	4.51E-06
cellular macromolecule biosynthetic process (GO:0034645)	285	41	19.86	2.06	3.83E-02
small molecule biosynthetic process (GO:0044283)	311	44	21.67	2.03	2.90E-02

cellular component organization or biogenesis (GO:0071840)	469	64	32.68	1.96	6.07E-04
carbohydrate derivative metabolic process (GO:1901135)	396	54	27.59	1.96	5.53E-03
carboxylic acid metabolic process (GO:0019752)	507	68	35.32	1.93	5.40E-04
cellular component organization (GO:0016043)	412	55	28.7	1.92	8.04E-03
gene expression (GO:0010467)	393	52	27.38	1.9	2.04E-02
organic substance biosynthetic process (GO:1901576)	910	119	63.4	1.88	3.52E-09
biosynthetic process (GO:0009058)	925	120	64.45	1.86	4.63E-09
organonitrogen compound metabolic process (GO:1901564)	986	127	68.7	1.85	1.06E-09
oxoacid metabolic process (GO:0043436)	531	68	37	1.84	2.25E-03
cellular nitrogen compound biosynthetic process (GO:0044271)	509	65	35.46	1.83	3.86E-03
cellular biosynthetic process (GO:0044249)	888	113	61.87	1.83	1.27E-07
organic acid metabolic process (GO:0006082)	545	68	37.97	1.79E+00	4.69E-03
small molecule metabolic process (GO:0044281)	876	104	61.03	1.70E+00	3.95E-05
nitrogen compound metabolic process (GO:0006807)	1530	168	106.6	1.58E+00	5.70E-09
cellular metabolic process (GO:0044237)	2036	211	141.85	1.49E+00	1.80E-11
organic substance metabolic process (GO:0071704)	1987	204	138.44	1.47E+00	4.42E-10
cellular nitrogen compound metabolic process (GO:0034641)	1038	106	72.32	1.47E+00	3.70E-02
primary metabolic process (GO:0044238)	1724	174	120.11	1.45E+00	2.95E-06
macromolecule metabolic process (GO:0043170)	1090	110	75.94	1.45E+00	3.69E-02
metabolic process (GO:0008152)	2202	220	153.42	1.43E+00	1.21E-10
cellular process (GO:0009987)	3004	273	209.3	1.30E+00	1.49E-13
biological_process (GO:0008150)	3405	292	237.23	1.23E+00	3.45E-14
Unclassified (UNCLASSIFIED)	987	14	68.77	2.00E-01	0.00E+00
**Molecular function**	***Escherichia coli* - Reflist (4392)**	**Gene list (306)**	**Expected**	**Fold Enrichment**	**P-value**
guanosine tetraphosphate binding (GO:0097216)	34	13	2.37	5.49	5.12E-03
ligase activity (GO:0016874)	110	23	7.66	3	1.08E-02
identical protein binding (GO:0042802)	432	73	30.1	2.43	9.42E-09
RNA binding (GO:0003723)	183	30	12.75	2.35	4.13E-02
purine ribonucleoside triphosphate binding (GO:0035639)	490	71	34.14	2.08	8.54E-06

carbohydrate derivative binding (GO:0097367)	581	84	40.48	2.08	2.52E-07
purine ribonucleotide binding (GO:0032555)	515	74	35.88	2.06	5.56E-06
purine nucleotide binding (GO:0017076)	516	74	35.95	2.06	5.85E-06
ATP binding (GO:0005524)	443	63	30.86	2.04	1.53E-04
adenyl ribonucleotide binding (GO:0032559)	449	63	31.28	2.01	2.88E-04
nucleotide binding (GO:0000166)	713	100	49.68	2.01	1.15E-08
nucleoside phosphate binding (GO:1901265)	713	100	49.68	2.01	1.15E-08
adenyl nucleotide binding (GO:0030554)	450	63	31.35	2.01	2.97E-04
ribonucleotide binding (GO:0032553)	553	77	38.53	2	9.15E-06
anion binding (GO:0043168)	733	101	51.07	1.98	2.04E-08
protein binding (GO:0005515)	1365	187	95.1	1.97	3.08E-22
small molecule binding (GO:0036094)	827	110	57.62	1.91	1.22E-08
ion binding (GO:0043167)	1338	155	93.22	1.66	1.37E-09
transferase activity (GO:0016740)	645	74	44.94	1.65	2.30E-02
catalytic activity (GO:0003824)	2035	227	141.78	1.6	1.16E-18
metal ion binding (GO:0046872)	803	88	55.95	1.57	1.45E-02
organic cyclic compound binding (GO:0097159)	1436	157	100.05	1.57	1.11E-07
heterocyclic compound binding (GO:1901363)	1436	157	100.05	1.57	1.11E-07
cation binding (GO:0043169)	815	89	56.78	1.57	1.57E-02
binding (GO:0005488)	2479	257	172.72	1.49	2.26E-20
molecular_function (GO:0003674)	3290	293	229.22	1.28	1.34E-18
Unclassified (UNCLASSIFIED)	1102	13	76.78	0.17	0.00E+00
**Cellular component**	***Escherichia coli* - Reflist (4392)**	**Gene list (306)**	**Expected**	**Fold Enrichment**	**P-value**
oxidoreductase complex (GO:1990204)	65	17	4.53	3.75E+00	2.06E-03
catalytic complex (GO:1902494)	166	34	11.57	2.94E+00	1.81E-05
cytosol (GO:0005829)	1063	153	74.06	2.07E+00	1.10E-18
protein-containing complex (GO:0032991)	635	88	44.24	1.99E+00	9.57E-08
intracellular anatomical structure (GO:0005622)	1467	194	102.21	1.90E+00	9.19E-23
cytoplasm (GO:0005737)	1434	187	99.91	1.87E+00	1.34E-20
cellular anatomical entity (GO:0110165)	2908	273	202.61	1.35E+00	2.15E-17
cellular_component (GO:0005575)	2969	274	206.86	1.32E+00	5.04E-16
Unclassified (UNCLASSIFIED)	1423	32	99.14	3.20E-01	0.00E+00

Table S5. GO Functional Enrichment analysis for down-regulated genes in ampicillin-resistant cells

**Biological processes**	***Escherichia coli* - Reflist (4392)**	**Gene list (228)**	**Expected**	**Fold Enrichment**	**P-value**
Unclassified (UNCLASSIFIED)	987	105	51.46	2.04	0.00E+00
biological_process (GO:0008150)	3405	124	177.54	0.7	5.30E-11
cellular process (GO:0009987)	3004	96	156.63	0.61	1.64E-12
nitrogen compound metabolic process (GO:0006807)	1530	37	79.77	0.46	1.25E-06
cellular metabolic process (GO:0044237)	2036	48	106.16	0.45	8.38E-12
metabolic process (GO:0008152)	2202	50	114.81	0.44	1.40E-14
primary metabolic process (GO:0044238)	1724	39	89.89	0.43	1.52E-09
organic substance metabolic process (GO:0071704)	1987	43	103.6	0.42	3.43E-13
establishment of localization (GO:0051234)	721	13	37.59	0.35	2.96E-03
transport (GO:0006810)	712	12	37.12	0.32	1.03E-03
organonitrogen compound metabolic process (GO:1901564)	986	16	51.41	0.31	1.17E-06
cellular biosynthetic process (GO:0044249)	888	14	46.3	0.3	6.67E-06
cellular nitrogen compound biosynthetic process (GO:0044271)	509	8	26.54	0.3	3.87E-02
organic substance biosynthetic process (GO:1901576)	910	14	47.45	0.3	2.99E-06
biosynthetic process (GO:0009058)	925	14	48.23	0.29	1.31E-06
transmembrane transport (GO:0055085)	596	9	31.08	0.29	3.16E-03
small molecule metabolic process (GO:0044281)	876	10	45.67	0.22	4.31E-08
ion transport (GO:0006811)	383	4	19.97	0.2	2.68E-02
carbohydrate derivative metabolic process (GO:1901135)	396	4	20.65	0.19	1.90E-02
carboxylic acid metabolic process (GO:0019752)	507	4	26.44	0.15	7.28E-05
oxoacid metabolic process (GO:0043436)	531	4	27.69	0.14	3.32E-05
organic acid metabolic process (GO:0006082)	545	4	28.42	0.14	1.39E-05
small molecule biosynthetic process (GO:0044283)	311	2	16.22	0.12	2.27E-02
organonitrogen compound biosynthetic process (GO:1901566)	520	3	27.11	0.11	6.19E-06
**Molcular function**	***Escherichia coli* - Reflist (4392)**	**Gene list (228)**	**Expected**	**Fold Enrichment**	**P-value**

Unclassified (UNCLASSIFIED)	1102	136	57.46	2.37E+00	0.00E+00
binding (GO:0005488)	2479	74	129.26	5.70E-01	8.81E-10
molecular_function (GO:0003674)	3290	93	171.54	5.40E-01	1.67E-23
organic cyclic compound binding (GO:0097159)	1436	40	74.87	5.30E-01	5.67E-04
heterocyclic compound binding (GO:1901363)	1436	40	74.87	5.30E-01	5.67E-04
protein binding (GO:0005515)	1365	38	71.17	5.30E-01	1.26E-03
transferase activity (GO:0016740)	645	12	33.63	3.60E-01	1.43E-02
catalytic activity (GO:0003824)	2035	35	106.11	3.30E-01	2.62E-19
metal ion binding (GO:0046872)	803	12	41.87	2.90E-01	1.89E-05
cation binding (GO:0043169)	815	12	42.49	2.80E-01	1.30E-05
ion binding (GO:0043167)	1338	16	69.76	2.30E-01	1.67E-14
oxidoreductase activity (GO:0016491)	442	4	23.05	1.70E-01	1.47E-03
ATP binding (GO:0005524)	443	4	23.1	1.70E-01	1.48E-03
transmembrane transporter activity (GO:0022857)	559	5	29.15	1.70E-01	3.39E-05
adenyl ribonucleotide binding (GO:0032559)	449	4	23.41	1.70E-01	9.95E-04
adenyl nucleotide binding (GO:0030554)	450	4	23.46	1.70E-01	9.94E-04
transporter activity (GO:0005215)	567	5	29.56	1.70E-01	2.24E-05
purine ribonucleoside triphosphate binding (GO:0035639)	490	4	25.55	1.60E-01	1.34E-04
purine ribonucleotide binding (GO:0032555)	515	4	26.85	1.50E-01	3.93E-05
purine nucleotide binding (GO:0017076)	516	4	26.9	1.50E-01	3.91E-05
ribonucleotide binding (GO:0032553)	553	4	28.83	0.14	7.38E-06
carbohydrate derivative binding (GO:0097367)	581	4	30.29	0.13	2.12E-06
nucleotide binding (GO:0000166)	713	4	37.18	0.11	1.62E-09
nucleoside phosphate binding (GO:1901265)	713	4	37.18	0.11	1.62E-09
anion binding (GO:0043168)	733	4	38.22	0.1	6.49E-10
small molecule binding (GO:0036094)	827	4	43.12	0.09	3.22E-12

**Cellular component**	***Escherichia coli* - Reflist (4392)**	**Gene list (306)**	**Expected**	**Fold Enrichment**	**P-value**
Unclassified (UNCLASSIFIED)	1423	126	74.2	1.70E+00	0.00E+00
cellular_component (GO:0005575)	2969	103	154.8	6.70E-01	1.55E-09
cellular anatomical entity (GO:0110165)	2908	97	151.62	6.40E-01	1.12E-10
protein-containing complex (GO:0032991)	635	14	33.11	4.20E-01	2.56E-02
intracellular anatomical structure (GO:0005622)	1467	27	76.49	3.50E-01	3.49E-11

cytoplasm (GO:0005737)	1434	26	74.77	3.50E-01	4.45E-11
cytosol (GO:0005829)	1063	12	55.43	2.20E-01	9.49E-12

Table S6. GO Functional Enrichment analysis for up-regulated genes in gentamicin-resistant cells

**Biological processes**	***Escherichia coli* - Reflist (4392)**	**Gene list (249)**	**Expected**	**Fold Enrichment**	**P-value**
organic phosphonate catabolic process (GO:0019700)	8	8	0.46	17.5	6.57E-04
organic phosphonate metabolic process (GO:0019634)	10	9	0.57	1.58E+01	2.24E-04
siderophore-dependent iron import into cell (GO:0033214)	15	9	0.86	1.05E+01	2.49E-03
primary alcohol catabolic process (GO:0034310)	22	9	1.26	7.16E+00	2.75E-02
iron import into cell (GO:0033212)	22	9	1.26	7.16E+00	2.75E-02
primary alcohol metabolic process (GO:0034308)	48	16	2.74	5.83E+00	1.78E-04
iron coordination entity transport (GO:1901678)	39	13	2.23	5.83E+00	2.69E-03
transition metal ion transport (GO:0000041)	72	19	4.11	4.62E+00	2.64E-04
iron ion transport (GO:0006826)	50	13	2.86	4.55	2.60E-02
metal ion transport (GO:0030001)	115	24	6.57	3.65	2.97E-04
alcohol metabolic process (GO:0006066)	90	18	5.14	3.5	1.66E-02
organic hydroxy compound metabolic process (GO:1901615)	124	21	7.09	2.96	3.01E-02
organic acid catabolic process (GO:0016054)	195	29	11.14	2.6	1.15E-02
small molecule catabolic process (GO:0044282)	298	38	17.03	2.23	8.98E-03
organic substance catabolic process (GO:1901575)	487	59	27.83	2.12	9.72E-05
catabolic process (GO:0009056)	502	60	28.69	2.09	9.03E-05
organic acid metabolic process (GO:0006082)	545	59	31.15	1.89	3.07E-03
carboxylic acid metabolic process (GO:0019752)	507	53	28.97	1.83	2.78E-02
small molecule metabolic process (GO:0044281)	876	80	50.06	1.6	2.04E-02
cellular metabolic process (GO:0044237)	2036	157	116.36	1.35	7.51E-04
organic substance metabolic process (GO:0071704)	1987	153	113.56	1.35	1.46E-03
metabolic process (GO:0008152)	2202	166	125.84	1.32	8.04E-04
cellular process (GO:0009987)	3004	217	171.68	1.26	1.56E-07
biological_process (GO:0008150)	3405	225	194.59	1.16	2.19E-03
Unclassified (UNCLASSIFIED)	987	26	56.41	0.46	0.00E+00
**Molecular functions**	***Escherichia coli* - Reflist (4392)**	**Gene list (249)**	**Expected**	**Fold Enrichment**	**P-value**
ATP hydrolysis activity (GO:0016887)	125	22	7.14	3.08	9.14E-03
ATP-dependent activity (GO:0140657)	190	28	10.86	2.58E+00	1.51E-02

adenyl ribonucleotide binding (GO:0032559)	449	51	25.66	1.99E+00	4.37E-03
adenyl nucleotide binding (GO:0030554)	450	51	25.72	1.98E+00	4.51E-03
ATP binding (GO:0005524)	443	50	25.32	1.97E+00	6.45E-03
purine ribonucleoside triphosphate binding (GO:0035639)	490	52	28	1.86E+00	2.47E-02
purine ribonucleotide binding (GO:0032555)	515	53	29.43	1.80E+00	3.77E-02
purine nucleotide binding (GO:0017076)	516	53	29.49	1.80E+00	3.87E-02
small molecule binding (GO:0036094)	827	84	47.26	1.78E+00	1.07E-04
ribonucleotide binding (GO:0032553)	553	56	31.6	1.77E+00	4.11E-02
anion binding (GO:0043168)	733	74	41.89	1.77E+00	1.12E-03
carbohydrate derivative binding (GO:0097367)	581	58	33.2	1.75E+00	3.91E-02
nucleotide binding (GO:0000166)	713	71	40.75	1.74E+00	3.22E-03
nucleoside phosphate binding (GO:1901265)	713	71	40.75	1.74E+00	3.22E-03
ion binding (GO:0043167)	1338	117	76.47	1.53E+00	2.38E-04
catalytic activity (GO:0003824)	2035	160	116.3	1.38E+00	7.38E-05
molecular_function (GO:0003674)	3290	227	188.02	1.21E+00	1.30E-06
Unclassified (UNCLASSIFIED)	1102	24	62.98	3.80E-01	0.00E+00
**Cellulkar component**	***Escherichia coli* - Reflist (4392)**	**Gene list (249)**	**Expected**	**Fold Enrichment**	**P-value**
carbon phosphorus lyase complex (GO:1904176)	5	5	0.29	1.75E+01	1.26E-02
ATP-binding cassette (ABC) transporter complex, substrate-binding subunit-containing (GO:0055052)	34	14	1.94	7.21E+00	1.65E-05
ATP-binding cassette (ABC) transporter complex (GO:0043190)	129	21	7.37	2.85E+00	6.26E-03
ATPase dependent transmembrane transport complex (GO:0098533)	133	21	7.6	2.76E+00	9.31E-03
Unclassified (UNCLASSIFIED)	1423	72	81.32	8.90E-01	0.00E+00

Table S7. GO Functional Enrichment analysis for down-regulated genes in gentamicin-resistant cells

**Biological processes**	***Escherichia coli* - Reflist (4392)**	**Gene list (427)**	**Expected**	**Fold Enrichment**	**P-value**
ribosomal small subunit assembly (GO:0000028)	21	16	2.06	7.76E+00	3.18E-05
ribonucleoprotein complex subunit organization (GO:0071826)	51	34	5	6.79E+00	6.16E-12
ribonucleoprotein complex assembly (GO:0022618)	51	34	5	6.79E+00	6.16E-12
ribosomal large subunit assembly (GO:0000027)	29	18	2.85	6.32E+00	4.23E-05
ribosomal small subunit biogenesis (GO:0042274)	28	17	2.75	6.19	1.29E-04
ribosomal large subunit biogenesis (GO:0042273)	30	18	2.94	6.11	6.22E-05
ribosome assembly (GO:0042255)	57	34	5.59	6.08	6.82E-11
(GO:0034249)	27	15	2.65	5.66	1.70E-03
negative regulation of cellular macromolecule biosynthetic process (GO:2000113)	26	14	2.55	5.49	5.06E-03
negative regulation of translation (GO:0017148)	26	14	2.55	5.49	5.06E-03
translation (GO:0006412)	116	61	11.38	5.36	3.66E-19
non-membrane-bounded organelle assembly (GO:0140694)	76	37	7.46	4.96	6.04E-10
organelle assembly (GO:0070925)	76	37	7.46	4.96	6.04E-10
peptide biosynthetic process (GO:0043043)	128	62	12.56	4.94	4.30E-18
(GO:0032269)	31	14	3.04	4.6	2.39E-02
negative regulation of protein metabolic process (GO:0051248)	34	15	3.34	4.5	1.51E-02
negative regulation of gene expression (GO:0010629)	43	18	4.22	4.27	3.72E-03
peptide metabolic process (GO:0006518)	154	63	15.11	4.17	1.83E-15
amide biosynthetic process (GO:0043604)	164	64	16.09	3.98	6.43E-15
regulation of cellular amide metabolic process (GO:0034248)	58	22	5.69	3.87	1.14E-03
regulation of translation (GO:0006417)	57	21	5.59	3.75	2.95E-03
(GO:2000112)	58	21	5.69	3.69	3.69E-03
ribosome biogenesis (GO:0042254)	107	38	10.5	3.62	5.34E-07
ribonucleoprotein complex biogenesis (GO:0022613)	108	38	10.6	3.59	6.64E-07
post-transcriptional regulation of gene expression (GO:0010608)	63	21	6.18	3.4	1.06E-02
protein-containing complex assembly (GO:0065003)	121	40	11.87	3.37	1.04E-06
protein-containing complex organization (GO:0043933)	128	42	12.56	3.34	4.62E-07
organelle organization (GO:0006996)	126	40	12.36	3.24	2.75E-06

cellular amide metabolic process (GO:0043603)	217	66	21.29	3.1	4.85E-11
regulation of cellular protein metabolic process (GO:0032268)	71	21	6.97	3.01	4.71E-02
regulation of protein metabolic process (GO:0051246)	78	23	7.65	3	2.11E-02
(GO:0010558)	153	37	15.01	2.46	3.91E-03
(GO:0031327)	154	37	15.11	2.45	6.93E-03
negative regulation of biosynthetic process (GO:0009890)	154	37	15.11	2.45	6.93E-03
negative regulation of metabolic process (GO:0009892)	191	45	18.74	2.4	5.98E-04
cellular macromolecule biosynthetic process (GO:0034645)	285	67	27.97	2.4	7.56E-07
(GO:0010605)	187	43	18.35	2.34	2.48E-03
(GO:0051172)	172	39	16.88	2.31	1.10E-02
negative regulation of biological process (GO:0048519)	235	53	23.06	2.3	1.98E-04
negative regulation of cellular metabolic process (GO:0031324)	173	39	16.98	2.3	1.16E-02
cellular protein metabolic process (GO:0044267)	307	69	30.13	2.29	2.59E-06
cellular component assembly (GO:0022607)	219	48	21.49	2.23	1.57E-03
negative regulation of cellular process (GO:0048523)	216	47	21.2	2.22	2.41E-03
gene expression (GO:0010467)	393	84	38.57	2.18	2.12E-07
macromolecule biosynthetic process (GO:0009059)	443	84	43.47	1.93	4.41E-05
protein metabolic process (GO:0019538)	419	78	41.12	1.9	3.30E-04
cellular nitrogen compound biosynthetic process (GO:0044271)	509	94	49.95	1.88	1.33E-05
Unclassified (UNCLASSIFIED)	987	140	96.86	1.45	0.00E+00
biological_process (GO:0008150)	3405	291	334.14	0.87	7.97E-03
cellular process (GO:0009987)	3004	235	294.79	0.8	1.19E-05
small molecule metabolic process (GO:0044281)	876	35	85.96	0.41	1.23E-07
localization (GO:0051179)	775	30	76.05	0.39	8.39E-07
cellular catabolic process (GO:0044248)	382	14	37.49	0.37	2.24E-02
establishment of localization (GO:0051234)	721	26	70.75	0.37	5.95E-07
carbohydrate metabolic process (GO:0005975)	389	14	38.17	0.37	1.66E-02
transport (GO:0006810)	712	25	69.87	0.36	4.56E-07
catabolic process (GO:0009056)	502	16	49.26	0.32	5.73E-05
ion transport (GO:0006811)	383	12	37.58	0.32	2.36E-03
organic substance transport (GO:0071702)	485	15	47.59	0.32	5.71E-05
organic substance catabolic process (GO:1901575)	487	15	47.79	0.31	5.80E-05
transmembrane transport (GO:0055085)	596	18	58.49	0.31	5.75E-07

carboxylic acid metabolic process (GO:0019752)	507	15	49.75	0.3	1.44E-05
nitrogen compound transport (GO:0071705)	281	8	27.58	0.29	3.07E-02
oxoacid metabolic process (GO:0043436)	531	15	52.11	0.29	2.40E-06
organic acid metabolic process (GO:0006082)	545	15	53.48	0.28	7.96E-07
anion transport (GO:0006820)	193	2	18.94	0.11	4.58E-03
**Molecular functions**	***Escherichia coli* - Reflist (4392)**	**Gene list (427)**	**Expected**	**Fold Enrichment**	**P-value**
structural constituent of ribosome (GO:0003735)	58	53	5.69	9.31	1.77E-24
large ribosomal subunit rRNA binding (GO:0070180)	10	9	0.98	9.17	1.27E-02
structural molecule activity (GO:0005198)	72	55	7.07	7.78	7.99E-23
rRNA binding (GO:0019843)	60	43	5.89	7.3	1.37E-16
mRNA binding (GO:0003729)	23	12	2.26	5.32	2.56E-02
RNA binding (GO:0003723)	183	65	17.96	3.62	1.30E-13
Unclassified (UNCLASSIFIED)	1102	168	108.14	1.55	0.00E+00
nucleic acid binding (GO:0003676)	706	107	69.28	1.54E+00	9.71E-03
molecular_function (GO:0003674)	3290	263	322.86	8.10E-01	1.78E-06
ion binding (GO:0043167)	1338	58	131.3	4.40E-01	2.88E-12
catalytic activity (GO:0003824)	2035	86	199.7	4.30E-01	7.17E-25
hydrolase activity (GO:0016787)	672	24	65.95	3.60E-01	1.58E-06
transferase activity (GO:0016740)	645	23	63.3	3.60E-01	4.73E-06
carbohydrate derivative binding (GO:0097367)	581	19	57.02	3.30E-01	5.66E-06
small molecule binding (GO:0036094)	827	26	81.16	3.20E-01	2.22E-10
purine ribonucleotide binding (GO:0032555)	515	15	50.54	3.00E-01	5.64E-06
purine nucleotide binding (GO:0017076)	516	15	50.64	3.00E-01	5.71E-06
ribonucleotide binding (GO:0032553)	553	16	54.27	2.90E-01	9.51E-07
active transmembrane transporter activity (GO:0022804)	277	8	27.18	2.90E-01	3.52E-02
nucleotide binding (GO:0000166)	713	20	69.97	2.90E-01	8.10E-10
nucleoside phosphate binding (GO:1901265)	713	20	69.97	2.90E-01	8.10E-10
anion binding (GO:0043168)	733	20	71.93	2.80E-01	1.71E-10
transporter activity (GO:0005215)	567	15	55.64	2.70E-01	9.92E-08
transmembrane transporter activity (GO:0022857)	559	14	54.86	2.60E-01	4.38E-08
adenyl ribonucleotide binding (GO:0032559)	449	11	44.06	2.50E-01	4.46E-06
adenyl nucleotide binding (GO:0030554)	450	11	44.16	2.50E-01	4.50E-06
ATP binding (GO:0005524)	443	10	43.47	2.30E-01	1.90E-06

purine ribonucleoside triphosphate binding (GO:0035639)	490	11	48.09	2.30E-01	1.50E-07
pyrophosphatase activity (GO:0016462)	185	3	18.15	1.70E-01	3.55E-02
ATP-dependent activity (GO:0140657)	190	2	18.65	1.10E-01	3.52E-03
**Cellular component**	***Escherichia coli* - Reflist (4392)**	**Gene list (427)**	**Expected**	**Fold Enrichment**	**P-value**
cytosolic small ribosomal subunit (GO:0022627)	25	23	2.45	9.38E+00	2.28E-10
small ribosomal subunit (GO:0015935)	25	23	2.45	9.38E+00	2.28E-10
cytosolic ribosome (GO:0022626)	60	55	5.89	9.34E+00	2.31E-26
ribosomal subunit (GO:0044391)	59	54	5.79	9.33E+00	7.90E-26
cytosolic large ribosomal subunit (GO:0022625)	34	31	3.34	9.29E+00	3.03E-14
large ribosomal subunit (GO:0015934)	34	31	3.34	9.29E+00	3.03E-14
ribonucleoprotein complex (GO:1990904)	61	55	5.99	9.19E+00	4.05E-26
ribosome (GO:0005840)	64	56	6.28	8.92E+00	3.56E-26
intracellular non-membrane-bounded organelle (GO:0043232)	102	61	10.01	6.09E+00	2.85E-22
intracellular organelle (GO:0043229)	109	62	10.7	5.80E+00	8.52E-22
non-membrane-bounded organelle (GO:0043228)	129	61	12.66	4.82E+00	2.97E-18
organelle (GO:0043226)	136	62	13.35	4.65E+00	5.99E-18
cytosol (GO:0005829)	1063	160	104.32	1.53E+00	2.09E-06
intracellular anatomical structure (GO:0005622)	1467	187	143.96	1.30E+00	5.77E-03
cytoplasm (GO:0005737)	1434	181	140.72	1.29E+00	1.61E-02
Unclassified (UNCLASSIFIED)	1423	151	139.64	1.08E+00	0.00E+00
membrane (GO:0016020)	1455	101	142.78	7.10E-01	4.32E-03
cell periphery (GO:0071944)	1352	84	132.68	6.30E-01	6.50E-05
plasma membrane (GO:0005886)	1241	73	121.78	6.00E-01	3.01E-05
intrinsic component of membrane (GO:0031224)	1140	67	111.87	6.00E-01	1.22E-04
integral component of membrane (GO:0016021)	1111	62	109.03	5.70E-01	2.01E-05
integral component of plasma membrane (GO:0005887)	590	29	57.9	5.00E-01	4.16E-03
intrinsic component of plasma membrane (GO:0031226)	606	29	59.47	4.90E-01	1.83E-03
transmembrane transporter complex (GO:1902495)	164	3	16.09	1.90E-01	3.55E-02
transporter complex (GO:1990351)	168	3	16.49	1.80E-01	2.43E-02

Table S8. KEGG pathway analysis in antibiotic-resistant cells

KEGG pathway for up-regulated genes in ampicillin-resistant cells

Term ID	Term description	Observed gene count	Background gene count	Strength	False discovery rate (FDR)	Matching genes in the network
eco01100	Metabolic pathways	120	890	0.26	2.25E-09	thrB,mog,pdxA,murE,murF,murD,murG,murC,ddlB,guaC,aceF,panB,hemL,pyrH,accA,frmA,hemB,ribD,thiI,folD,ubiF,pgm,galK,galE,pgl,moeA,dmsA,dmsC,pflB,fabA,mgsA,fabD,ptsG,ispE,kdsA,add,gdhA,gapA,sdaA,manX,manY,manZ,pykA,hisI,gnd,dcd,preT,preA,nrdA,nuoN,nuoM,nuoL,nuoK,nuoF,yfbT,pta,accD,fabB,guaB,pepB,suhB,pdxJ,ispF,sdaB,lysA,gcvP,gcvH,ubiI,ubiH,tktA,speB,gshB,ansB,cpdA,hldE,tdcE,tdcB,garK,garR,garL,glmM,murA,kdsD,nanA,accC,gph,aroB,malQ,glgA,gor,cysE,gpmM,kbl,spoT,ilvB,tnaA,atpG,ilvE,gpp,hemX,hemC,hemN,tpiA,glpX,katG,gldA,ppc,hemE,plsB,melA,fumB,frdC,psd,rsgA,miaA,treC,pepA,serB,ytjC,tdcG
eco01110	Biosynthesis of secondary metabolites	58	339	0.36	6.75E-07	thrB,aceF,panB,hemL,accA,frmA,hemB,ribD,ubiF,pgm,pgl,fabD,ispE,gapA,sdaA,pykA,hisI,gnd,accD,guaB,suhB,ispF,sdaB,lysA,gcvP,gcvH,ubiI,ubiH,tktA,ansB,tdcB,garK,accC,gph,aroB,malQ,glpD,glgA,cysE,gpsA,gpmM,ilvB,ilvE,hemX,hemC,hemN,tpiA,glpX,katG,hemE,plsB,fumB,frdC,psd,miaA,serB,ytjC,tdcG
eco01200	Carbon metabolism	27	109	0.52	1.40E-05	aceF,accA,frmA,folD,pgl,gapA,sdaA,pykA,gnd,pta,accD,sdaB,gcvP,gcvH,tktA,tdcB,accC,cysE,gpmM,tpiA,glpX,ppc,fumB,frdC,serB,ytjC,tdcG
eco00260	Glycine, serine and threonine metabolism	12	37	0.64	0.0025	thrB,sdaA,sdaB,gcvP,gcvH,tdcB,garK,gpmM,kbl,serB,ytjC,tdcG
eco00970	Aminoacyl-tRNA biosynthesis	9	25	0.68	0.0083	ileS,proS,cysS,tyrS,pheT,pheS,hisS,glyS,glyQ
eco03430	Mismatch repair	8	22	0.69	0.0139	dnaX,holB,mutS,recJ,dnaN,ssb,mutL,holC
eco00061	Fatty acid biosynthesis	6	13	0.79	0.019	accA,fabA,fabD,accD,fabB,accC
eco00550	Peptidoglycan biosynthesis	8	24	0.65	0.019	murE,murF,murD,murG,murC,ddlB,mrcB,murA
eco00520	Amino sugar and nucleotide sugar metabolism	11	46	0.51	0.0192	nagE,pgm,galK,galE,ptsG,manX,manY,manZ,glmM,murA,nanA
eco01120	Microbial metabolism in diverse environments	35	269	0.24	0.0192	thrB,aceF,hemL,accA,frmA,hemB,folD,nfsB,pgm,pgl,nfsA,pflB,mgsA,gdhA,gapA,pykA,gnd,pta,accD,lysA,tktA,tdcE,garK,accC,cysE,gpmM,hemX,hemC,tpiA,glpX,ppc,fumB,frdC,serB,ytjC
eco03440	Homologous recombination	8	27	0.6	0.0226	dnaX,recR,holB,recA,recJ,dnaN,ssb,holC
eco03030	DNA replication	6	17	0.68	0.0367	dnaX,holB,dnaG,dnaN,ssb,holC
eco00010	Glycolysis / Gluconeogenesis	10	47	0.46	0.0439	aceF,frmA,pgm,ptsG,gapA,pykA,gpmM,tpiA,glpX,ytjC
eco00620	Pyruvate metabolism	11	55	0.43	0.0439	aceF,accA,pflB,pykA,pta,accD,tdcE,accC,ppc,fumB,frdC
eco01230	Biosynthesis of amino acids	18	117	0.32	0.0439	thrB,gapA,sdaA,pykA,hisI,sdaB,lysA,tktA,tdcB,aroB,cysE,gpmM,ilvB,ilvE,tpiA,serB,ytjC,tdcG

KEGG pathway for up-regulated genes in gentamicin-resistant cells

Term ID	Term description	Observed gene count	background gene count	Strength	False discovery rate (FDR)	Matching genes in the network
eco00440	Phosphonate and phosphinate metabolism	7	8	1.16	0.00095	phnP,phnM,phnL,phnJ,phnI,phnH,phnG
eco01100	Metabolic pathways	82	890	0.18	0.0036	carB,araB,leuC,leuB,ilvI,yagE,yagF,yagH,argF,paoB,glxK,purE,bioA,bioC,moeA,ssuD,rutF,rutD,putA,chaC,puuC,puuB,puuE,paaD,paaJ,astD,hisD,fabB,eutC,eutA,eutG,eutD,eutT,purL,lhgO,gabD,gabT,cysJ,sdaB,ubiH,scpA,kbaZ,nanK,nanE,mrcA,glgA,lyxK,sgbH,selA,ilvB,ilvA,metE,fadA,yihU,metL,gldA,ppc,argB,argH,thiG,thiF,thiE,hemE,purD,acs,phnP,phnN,phnM,phnL,phnJ,phnI,phnH,phnG,ulaD,ulaF,qorB,ahr,yjhG,serB,glcF,glcE,dgoA
eco02010	ABC transporters	24	179	0.34	0.0191	thiP,fhuC,fhuB,fepG,fepD,ssuB,yecS,yehY,cysA,cysW,yphD,ugpC,livG,livH,nikB,nikC,nikD,nikE,dppF,dppC,phnC,fecD,fecC,fecB

KEGG pathway for down-regulated genes in gentamicin-resistant cells

Term ID	Term description	Observed gene count	background gene count	Strength	False discovery rate (FDR)	Matching genes in the network
eco03010	Ribosome	52	56	0.95	6.77e-24	rpsT,rpsB,ykgM,rpsA,rpmF,rplT,rpmI,rplY,rpsP,rpsU,rpsO,rpmA,rplU,rpsI,rplM,rplQ,rpsD,rpsK,rpsM,rpmJ,rplO,rpmD,rpsE,rplR,rplF,rpsH,rpsN,rplE,rplX,rplN,rpsQ,rpmC,rpsC,rplV,rpsS,rplB,rplW,rplC,rpsJ,rpsL,rpmG,rpmB,rpmH,rpmE,rplK,rplA,rplJ,rplL,rpsF,rpsR,rplI,ykgO

Table S9. Topological characteristics of Protein-Protein Interaction networks

A) PPI network for up-regulated genes in ampicillin-resistant cells

Number of nodes	Number of edges	Average node degree	Average local clustering coefficient	Expected number of edges	PPI enrichment p-value
307	685	4.46	0.471	415	< 1.0e-16

Betweenness Centrality	Closeness Centrality	Degree	Display name
0	0.666666667	1	*rimK*
0			
1	1	2	*nfsA*
0			
0	0.232535885	1	*argP*
0			
0.01416138	0.213157895	4	*lptA*
0.295454545			
0	0	0	*htpX*
0.014158115	0.263843648	4	*nrdA*
0.025995564	0.25498426	7	*bamC*
0	0.175832127	1	*ptsN*
0			
0.008230453	0.244466801	5	*glyQ*
0.428571429			
0.018490847	0.262702703	7	*rluD*
0.230414747			
0	0.194711538	3	*manZ*
0.034768194	0.262987013	9	*surA*
0.178294574			
0.01112588	0.2541841	4	*pdxA*
0.294642857			
0.078368237	0.303370787	14	*rsmA*
0.129539952			
0.011971466	0.293833132	6	*pyrH*
0.255952381			
0	0	0	*proQ*
0			
0.003729047	0.23387873	4	*ybgF*
0.002209678	0.224791859	3	*pal*
0.421052632			
0.091859938	0.319316689	14	*gyrB*
0.162337662			
0.009287159	0.275822928	10	*pheS*
0.061744161	0.295980512	15	*pheT*
0.151190476			
0	0.164745763	1	*flu*
0.002517227	0.226046512	6	*accA*
0.425925926			
0.046277077	0.249230769	6	*rlpA*
0.2			
0.019066723	0.255520505	2	*opgH*
0.035243006	0.255252101	10	*tktA*
0.203333333			
0	0	0	*speB*
0			
0	1	1	*galE*
0			
0	0	0	*nanA*
0			
0.013801266	0.244959677	5	*rlmH*
0.246153846			
0	1	1	*galK*
0			
0	0.176214648	1	*yebE*
0			
0	0	0	*sspB*
0	0	0	*bepA*
0			
0	0.232313576	2	*proS*
0.735294118			
8.50E-06	0.18984375	3	*cpxR*
0.566666667			
0.02977348	0.266447368	10	*pykA*
0.236111111			
0	0	0	*brnQ*
0.023847461	0.243731194	5	*lptE*
0.25			
4.56E-04	0.176470588	3	*gshB*
0.444444444			
0	0	0	*srlM*
0.003232511	0.264994547	11	*murD*
0.315789474			
0.122184592	0.304511278	12	*tpiA*
0.154320988			
4.56E-05	0.201492537	4	*tdcF*
0.486111111			
0	0.178414097	2	*secF*
0.7			
0	0.184090909	1	*preT*
0			
0.038169164	0.2109375	4	*secD*
0.3			
0.033411607	0.237304687	10	*tdcE*
0.236363636			
0.013646232	0.232981783	4	*folD*

Hub genes																		
ftsZ	*recA*	*pheT*	*murF*	*dnaA*	*groL*	*rsmA*	*gyrB*	*ftsA*	*dnaN*	*lepA*	*tdcB*	*rsmH*	*murG*	*ftsN*	*mreB*	*dnaG*	*tpiA*	
ompF	*murD*	*murE*	*pta*	*lamB*	*dnaX*	*mutL*	*pheS*	*tktA*	*pykA*	*tdcE*	*murC*	*hisS*	*pflB*	*murA*	*ileS*	*surA*	*dmsA*	
ddlB	*nuoF*	*frdC*	*malE*	*prfA*	*mrcB*	*ompC*	*dmsC*	*accC*	*tdcG*	*ppc*	*guaB*	*gapA*	*rodZ*	*cysE*	*bamB*	*aceF*	*recJ*	
malF	*malG*	*malK*	*hemE*	*ptsG*	*accD*	*hflX*	*bamC*	*rluD*	*cysS*	*nuoN*	*nuoM*	*nuoL*	*nuoK*	*ribD*	*mreC*	*glmM*		
recR	*obgE*	*prfB*	*thrB*	*hflC*	*sdaA*	*pyrH*	*accA*	*rlpA*	*gnd*	*pgm*	*holB*	*malM*	*amiB*	*lexA*	*truA*	*ssb*	*sdaB*	*miaA*
hflK	*malT*	*fabB*	*malQ*	*ilvE*	*fabA*	*glyQ*	*rlmH*	*lptE*	*hemY*	*hemC*	*tyrS*	*hslV*	*fabD*	*gpsA*	*holC*	*hybD*	*nusA*	
hybB	*hybA*	*acrA*	*serB*	*gpmM*	*lptG*	*hemB*	*plsB*	*lpoB*	*era*	*hemL*	*gcvP*							

0.357142857			
0.003007484	0.250515464	4	*hisI*
0.404761905			
0.008230453	0.22541744	2	*preA*
0.5			
7.71E-04	0.199180328	4	*tdcC*
0.426470588			
0.033516442	0.229461756	13	*tdcB*
0.230769231			
0.03411876	0.273957159	7	*cysS*
0.244360902			
0.003577729	0.19239905	3	*queA*
0.428571429			
0	0.197721725	1	*pgl*
0			
0.008935764	0.246200608	6	*gnd*
0.355072464			
0	0.186923077	2	*tdcA*
0.653846154			
4.39E-04	0.19724026	4	*cpxA*
0.01343602	0.273957159	15	*murF*
0.255284553			
0.01467319	0.270300334	11	*murE*
0.313397129			
0.026035825	0.284543326	13	*rsmH*
0.216524217			
7.23E-04	0.209663503	5	*hemY*
0.5			
0.005011509	0.263843648	3	*add*
0.385416667			
0	0	0	*yggW*
0			
0.005343512	0.195809831	8	*dmsC*
0	0	0	*yebC*
0			
0.006647976	0.219314079	4	*hemX*
0	0	0	*ansB*
0			
2.83E-05	0.205583756	3	*tsx*
0.533333333			
0.00280203	0.208583691	2	*glpX*
0.5			
0.026574041	0.200164745	9	*dmsA*
0.452380952			
7.06E-04	0.209663503	5	*hemC*
0.54			
0.011097117	0.236842105	3	*lysA*
0.333333333			
0	0	0	*rihA*
0			
0	0.200660611	1	*rarA*
0			
0	0	0	*tldD*
0			
5.84E-04	0.204717776	7	*nuoN*
0.571428571			
0.016392885	0.194555645	4	*garK*
0.428571429			
5.84E-04	0.204717776	7	*nuoM*
0.571428571			
0	0.163087248	2	*garR*
0.75			
5.84E-04	0.204717776	7	*nuoL*
5.84E-04	0.204717776	7	*nuoK*
0.571428571			
0	0.163087248	2	*garL*
0.75			
0	1	3	*rppH*
1			
0	0.2211101	2	*yhdE*
0.769230769			
0	1	1	*lnt*
0			
0.033050271	0.233653846	7	*ribD*
0.181818182			
0.123935638	0.323568575	27	*ftsZ*
0.008633858	0.278350515	14	*ftsA*
0.232142857			
1.02E-04	0.260450161	9	*ddlB*
0.382716049			
0.001166095	0.264417845	10	*murC*
0.351351351			
0.003394621	0.270300334	13	*murG*
0.298076923			
0	0.207161125	1	*thiI*
0			
0	0.139175258	3	*hypB*
0.733333333			
0	0.13869863	1	*ubiF*
0			

0	0.139175258	3	*hypD*
0	1	1	*ybeZ*
0	1	1	*flgF*
0.029347547	0.236381323	8	*accC*
0.291666667			
1.50E-04	0.250515464	5	*tyrS*
0.472727273			
0	0	0	*yigA*
0	0	0	*ynjE*
0			
0.012023914	0.270601336	5	*hslV*
0.251851852			
0.066268178	0.284876905	13	*ftsN*
0.14439946			
0.00157814	0.255252101	7	*mreC*
0.398268398			
0.017430997	0.283216783	13	*mreB*
0.218623482			
0.066955408	0.235237173	9	*nuoF*
0.385620915			
0	1	1	*flgB*
0.008230453	0.174946004	2	*gldA*
0.5			
0	0	0	*rhlE*
0			
0	0	0	*ybiH*
0			
0	0	0	*tnaA*
0			
0.003037868	0.20894239	3	*gdhA*
0.41025641			
0.010855519	0.246700508	4	*damX*
0.3125			
0.010162349	0.237073171	3	*aroB*
0.380952381			
0	0.139175258	3	*hypE*
0.733333333			
0	0.123664122	1	*treC*
0			
0.034086602	0.243486974	4	*katG*
0.291666667			
0	0	0	*ydhF*
0			
0.034695237	0.293478261	14	*dnaN*
0.17364532			
0	0.231428571	2	*mutS*
0.657894737			
0.016392885	0.163857047	2	*nagE*
0.068069577	0.302615193	15	*dnaA*
0.164179104			
0	0	0	*melA*
0			
0.005320681	0.22293578	8	*tdcG*
0.326086957			
0	0	0	*yfbT*
0			
0.004212858	0.246200608	3	*gph*
0.37037037			
5.38E-04	0.22752809	3	*mpl*
0.5			
0.039558414	0.266155531	11	*pta*
0.230958231			
0	1	3	*rne*
0.015879865	0.257688229	8	*ppc*
0.279166667			
0.03441743	0.255252101	6	*pgm*
0.23655914			
7.59E-04	0.206808511	2	*ispF*
0.5			
0	0.177113703	2	*marB*
0.039441605	0.190737834	3	*tatB*
0.004098221	0.214854111	4	*marA*
0.057527955	0.289976134	8	*guaB*
0.158536585			
0.004098221	0.214854111	4	*marR*
0.383333333			
0.00118627	0.253653445	6	*holB*
0.385416667			
0	0	0	*yhbS*
0			
0	0	0	*yejM*
0			
0	1	1	*ygiB*
0	1	1	*ygiC*
0			
0.031826563	0.286219081	8	*gapA*
0.230263158			
0.024555317	0.160927152	4	*ubiJ*
0.375			
0	0	0	*yraP*
0			

0.008230453	0.141033082	2	*treB*
0.5			
0.008230453	0.241071429	2	*parE*
0.5			
0	0.232535885	1	*hupA*
0			
0	0.1944	1	*cpdA*
0			
0	0	0	*dcd*
0			
0	0.186206897	1	*fumB*
0			
0	0.1867794	1	*ridA*
0			
3.91E-05	0.205063291	5	*fabD*
0.618181818			
0	0.666666667	1	*nfsB*
0			
0.012172558	0.275822928	8	*rodZ*
0.037135271	0.244221106	8	*cysE*
0.259259259			
0.026258222	0.248212462	5	*gpsA*
0.215384615			
0	1	1	*ygeX*
0			
0.008849553	0.231870229	5	*holC*
0.410526316			
0.024487297	0.161354582	5	*hybD*
0.475			
0.008696642	0.257415254	5	*nusA*
0.242424242			
0.008298473	0.197080292	4	*oxyR*
0.45			
0.016772485	0.191188041	5	*hybB*
0.016772485	0.191188041	5	*hybA*
0.028010833	0.266739846	8	*bamB*
0.1875			
0.030806614	0.278032037	10	*hisS*
0.1925			
4.88E-05	0.209482759	2	*prfC*
0.7			
0.013003714	0.252336449	2	*hscA*
0.5			
9.50E-04	0.23661149	3	*guaC*
0.454545455			
0	0.171489061	1	*kbl*
0			
0.032251035	0.253918495	5	*acrA*
0	0.202668891	1	*acrB*
0			
0	0.261290323	2	*efp*
0.58			
0	0.206281834	5	*serB*
0.494117647			
0.068579569	0.268211921	10	*pflB*
0.214285714			
0.02487348	0.240118577	5	*gpmM*
0.37			
0.032472082	0.257961783	3	*atpG*
0.333333333			
0.00564815	0.205236486	3	*pepB*
0.466666667			
0.002608789	0.194711538	3	*pepA*
0.416666667			
0	0	0	*yjjI*
0			
0.006831303	0.212598425	5	*lptG*
0.44			
0	0.18984375	2	*hldE*
0.666666667			
0.168791933	0.333333333	15	*groL*
0.114056225			
0.00108938	0.274886878	4	*groS*
0.013971354	0.246700508	7	*glmM*
0.487012987			
0	0	0	*cca*
0			
0	1	1	*ygfM*
0			
0.016649421	0.241550696	8	*aceF*
0.284482759			
0	0	0	*frmA*
0			
0.00853696	0.250773994	7	*recR*
3.58E-04	0.231208373	2	*dsbA*
0.533333333			
0	0.221917808	6	*malM*
0.006304974	0.263271939	10	*murA*
0.328947368			
0.005979191	0.25498426	8	*recJ*
0.246621622			
0.006139801	0.253388947	4	*nusG*
0.020063594	0.24251497	11	*lamB*

0.269592476			
0.00895673	0.21793722	4	*trmJ*
0.325			
0.002484385	0.197560976	3	*gor*
0.476190476			
0	0.255520505	4	*zipA*
0.478571429			
0.003392733	0.2211101	3	*suhB*
0.4			
0.005097777	0.23776908	2	*ispE*
0.5			
8.50E-06	0.20385906	3	*hemN*
0.666666667			
0.001807065	0.228813559	2	*slt*
0.5			
0.057813585	0.262702703	3	*ampC*
0.345238095			
0	0.20610687	1	*ettA*
0			
0.060850817	0.228598307	9	*frdC*
0.011188845	0.26880531	7	*obgE*
0.003946302	0.229245283	8	*malF*
0.388157895			
0.003946302	0.229245283	8	*malG*
0.388157895			
0.001823116	0.204717776	3	*ytjC*
0.487179487			
0.003946302	0.229245283	8	*malK*
0.388157895			
0.033360482	0.30489335	13	*dnaG*
0.180252583			
0.012528782	0.275198188	4	*tufB*
0.291666667			
0.010237599	0.255789474	7	*prfB*
0.236607143			
0.038855661	0.267032967	9	*malE*
0.00952317	0.268211921	11	*dnaX*
0.247357294			
0.00465942	0.217741935	5	*hemB*
0.008837527	0.214854111	4	*kdsA*
0.339285714			
0	0	0	*cueR*
0.015424083	0.224791859	7	*thrB*
0.301587302			
0.001883094	0.224584104	4	*prmC*
0.397058824			
0.026777335	0.255520505	9	*prfA*
0.218390805			
0.008489953	0.257142857	6	*amiB*
0.252525253			
8.50E-05	0.213908451	4	*ldtD*
0.037088889	0.245951417	8	*hemE*
0.232954545			
0	0.156976744	1	*mog*
0			
0	0.197560976	1	*nfi*
0			
0.007581427	0.246450304	4	*rsgA*
0.315217391			
0.002553642	0.221917808	2	*psd*
0.020784151	0.235009671	5	*plsB*
0.271428571			
0.001501211	0.265283843	6	*lexA*
0.315972222			
0.012693211	0.212971078	4	*kdsD*
0.288461538			
0.004629464	0.261009667	5	*lpoB*
0.034607536	0.261009667	6	*truA*
0.166666667			
0.0060173	0.224584104	3	*usg*
0.333333333			
0	0.181750187	1	*panB*
0			
0.054094186	0.240356083	8	*ptsG*
0.237068966			
6.60E-04	0.198205546	3	*tesA*
0.566666667			
0.001157519	0.250773994	6	*ssb*
0	0	0	*ftnA*
0			
0	0	0	*yfeX*
0			
0	0.255789474	2	*hslO*
4.29E-04	0.211488251	6	*sdaB*
0.421052632			
0	0	0	*dps*
0			
0	0	0	*yicG*
0			
4.39E-04	0.19724026	4	*envZ*
3.68E-05	0.19724026	4	*ompR*
0.517857143			
0.023369376	0.244221106	8	*accD*

0.298913043			
0	0	0	*qorA*
0			
0.00860945	0.255520505	6	*miaA*
0.275641026			
0.003241634	0.242031873	6	*hflK*
0.014633819	0.259338314	8	*hflX*
0.019663418	0.273648649	7	*hflC*
0.032656202	0.244221106	12	*ompF*
0.166666667			
0.002726033	0.22541744	2	*ybbN*
0.5			
0.014704552	0.267916207	11	*mutL*
0.237762238			
0.043482151	0.270601336	9	*mrcB*
0.189701897			
0	0	0	*hns*
0	0.221917808	6	*malT*
0.555555556			
0.016263937	0.279310345	10	*ileS*
0.216666667			
0.008230453	0.213720317	3	*ldtC*
0.481481481			
0	1	3	*pcnB*
1			
0.008230453	0.186064319	2	*moeA*
0.001425821	0.230769231	3	*pdxJ*
0.416666667			
0.001715774	0.205236486	6	*fabB*
0.545454545			
0.005729782	0.265283843	5	*era*
0.303030303			
0.051841952	0.306431274	14	*lepA*
0.146825397			
0.013599797	0.233653846	6	*malQ*
0.427083333			
0	0.197560976	1	*gpp*
0			
0	0.21334504	2	*ldtB*
0.722222222			
0.014597646	0.221917808	6	*ilvE*
0.310606061			
0.012411043	0.245951417	4	*spoT*
0.008834325	0.250515464	4	*rlmB*
0.333333333			
0	0.208583691	2	*glgA*
0.6			
0.004597088	0.217741935	5	*hemL*
0.428571429			
0	0	0	*tsaB*
0			
0	1	3	*dppD*
1			
0	0	0	*yedD*
0	1	3	*dppC*
1			
0	1	3	*dppB*
1			
0	1	3	*srmB*
1			
0.090089629	0.31640625	20	*recA*
0.018379645	0.231870229	9	*ompC*
0.226495726			
0.017447363	0.211488251	4	*glpD*
0.3			
0.006427401	0.21677074	6	*fabA*
0.416666667			
0.052648886	0.227954972	4	*fdhF*
0.329545455			
0	0	0	*yecS*
0			
0	0.203688181	1	*prmB*
0			
0	1	3	*dppF*
1			
0	0	0	*rihC*
0			
0	0.148988351	1	*mgsA*
0.010697365	0.206808511	5	*gcvP*
0.261538462			
0.003838608	0.213532513	4	*gcvH*
0.316666667			
0	0.138777841	2	*ubiI*
0.75			
0	0.138777841	2	*ubiH*
0.75			
0	0.21677074	2	*hflD*
0.8125			

0	0.194711538	3	*manY*
0.024487297	0.195180723	4	*manX*
0.416666667			
0	0	0	*yidF*
0			
0	0	0	*yoaE*
0			
0	0.19628433	2	*ahpF*
0.8			
0.005222178	0.21334504	7	*sdaA*
0.331168831			
0	0.196601942	1	*glyS*
0			
0	0	0	*yfiF*
0			
0	0.189547582	2	*ilvB*
0.633333333			

B) PPI network for up-regulated genes in gentamicin-resistant cells

Number of nodes	Number of edges	Average node degree	Average local clustering coefficient	Expected number of edges	PPI enrichment p-value
252	311	2.47	0.581	121	< 1.0e-16

Betweenness Centrality	Closeness Centrality	Degree	Display name
0	0	0	*gspH*
0			
0	0	0	*rimK*
0			
0.097222222	0.529411765	4	*hyaA*
0	0	0	*scpA*
0			
1	1	2	*tmcA*
0			
0	0.375	2	*hyaF*
1			
0	0	0	*citX*
0.472334683	0.26	3	*ugpQ*
0.541666667			
0.097222222	0.529411765	4	*hyaD*
0.625			
0.002469136	1	10	*phnM*
0.98			
0.002469136	1	10	*phnN*
0.98			
0.002469136	1	10	*phnK*
0.98			
0	0.909090909	9	*phnL*
1			
0.002469136	1	10	*phnI*
0.98			
0.002469136	1	10	*phnJ*
0.98			
0.002469136	1	10	*phnP*
0.98			
0	0	0	*tilS*
0			
0	0	0	*thiP*
0			
0.051282051	0.179723502	2	*paaJ*
0.5			
0	1	1	*livG*
0			
0.333333333	0.6	3	*yiaN*
0	1	1	*livH*
0	0.666666667	1	*yjhP*
0			
0.456140351	0.240740741	3	*ugpC*
0.416666667			
0	0	0	*yehM*
0	0	0	*flu*
0	0.4	1	*araB*
0			
0	0.152941176	1	*paaD*
0	0.248677249	4	*ilvI*
0.583333333			
0	0.274853801	5	*recD*
0.557142857			
0	0.233830846	1	*hda*
0			
0	1	1	*nanK*
0			
0	1	1	*nanE*
0			
0	0	0	*fdrA*
0			
0	0.666666667	1	*zntA*
0			
0	0	0	*glxK*
0			
0.066666667	0.6	3	*lyxK*
0.666666667			
0.4	0.75	4	*sgbH*
0.5			
0	0.171052632	1	*yehY*
0			
0	0.248677249	4	*leuB*

Hub genes

phnM	*phnN*	*phnK*	*phnI*	*phnJ*	*phnP*	*phnF*	*phnG*	*phnH*	*mutL*	*phnL*	*phnC*	*recQ*	*purL*	*ilvA*	*recG*	*recR*		
gabT	*nikB*	*uvrD*	*hycE*	*dnaA*	*dnaX*	*yihU*	*carB*	*eutG*	*eutA*	*fhuC*	*recD*	*hycD*	*ftsN*	*hypE*	*puuE*	*fepG*	*entF*	
fepD	*fecC*	*fecD*	*eutC*	*eutT*	*ilvB*	*hyaA*	*hyaD*	*ilvI*	*sgbH*	*leuB*	*leuC*	*purE*	*nikC*	*hisD*	*hycG*	*hypF*	*hycB*	*hycC*
metL	*argB*	*argH*	*entA*	*entS*	*gabD*	*amiB*	*yihQ*	*eutK*	*fecR*	*fecA*	*fecB*	*acs*	*eutD*	*ugpQ*	*yiaN*	*ugpC*	*lyxK*	
nikD	*nikE*	*metE*	*purD*	*mrcA*	*puuC*	*priC*	*cysJ*	*glnE*	*thiF*	*ampH*	*ssb*	*yihV*	*sdaB*	*ygdG*	*yjhG*	*yihT*	*nrfD*	*nrfC*
ulaD	*ulaF*	*dppF*	*argF*															

0.583333333			
0	0.248677249	4	*leuC*
0.583333333			
6.94E-04	0.286585366	4	*purE*
0.477272727			
0	0.219626168	1	*srlM*
0.042553191	0.279761905	2	*srlQ*
0.5			
0	0.140794224	1	*acpT*
0			
0.642857143	0.875	6	*nikB*
0.444444444			
0.047619048	0.636363636	4	*nikC*
0.625			
0	0.583333333	3	*nikD*
0.722222222			
0	1	1	*selB*
0			
0	1	1	*selA*
0			
0	0	0	*rhsA*
0			
0.038852914	0.286585366	4	*hisD*
0.454545455			
0	0	0	*yhhS*
0	1	1	*ypdE*
0			
0	1	1	*ypdF*
0			
0	0	0	*yggW*
0			
0	0	0	*lplT*
0	0.666666667	1	*bioA*
0			
0	0.5	4	*hycG*
0.791666667			
0	0	0	*yhiI*
0	0.4	1	*yeiE*
0			
0	0	0	*yhdE*
0			
0	0.194029851	1	*astD*
0			
0	0	0	*norW*
0			
0.125	0.642857143	4	*hypF*
1	1	2	*bioC*
0			
0	0.583333333	3	*nikE*
0.722222222			
0	0	0	*rhsB*
0			
0	0	0	*qorB*
0			
0	0.5	4	*hycB*
0.02004317	0.281437126	6	*uvrD*
0.458333333			
3.08E-04	0.247368421	2	*priA*
0.666666667			
0	0	0	*ynjA*
0			
0	1	1	*flgF*
0	0	0	*rsmJ*
0			
0	0	0	*yhfS*
0			
0.002469136	1	10	*phnF*
0.98			
0	1	2	*glcE*
1			
0	1	2	*glcF*
0	0	0	*yigA*
0.305555556	0.692307692	6	*hycE*
0.541666667			
0	0.909090909	9	*phnC*
1			
0.125	0.642857143	5	*hycD*
0.575			
0	0.5	4	*hycC*
0.200740056	0.231527094	5	*ftsN*
0.3			
0	1	1	*kbaZ*
0			
0.002469136	1	10	*phnG*
0.98			
0.002469136	1	10	*phnH*
0.98			
0	0	0	*gldA*
0			
0	0	0	*ygbK*
0			
0	0.235	2	*metR*
0.010329941	0.246073298	3	*metE*
0.481481481			
0	1	1	*dgoA*
0			

0	0	0	*qseB*
0			
0.305555556	0.692307692	5	*hypE*
0.511111111			
0.066604995	0.288343558	4	*metL*
0.35			
0.022047487	0.279761905	8	*recQ*
0.423076923			
0	0.259668508	2	*mutS*
0.65			
0.140918902	0.303225806	6	*dnaA*
0.363636364			
0	0	0	*yhaM*
0			
0	0.666666667	1	*rtcA*
0			
0	1	1	*flgJ*
0			
0	0.210810811	1	*ppc*
0			
6.94E-04	0.230392157	4	*argB*
0.607142857			
0	1	2	*glcC*
1			
0	0	0	*yhbU*
0			
0	0.284848485	3	*purD*
0.545454545			
0	0	0	*yraQ*
0	1	1	*hofN*
0	1	1	*hofM*
0			
0.020814061	0.191056911	3	*mrcA*
0.555555556			
0	0	0	*yigZ*
0			
0.033288349	0.203125	5	*puuE*
0.4			
0	0.170305677	2	*puuB*
0.8			
0	1	1	*kdpD*
0			
0.020692758	0.196969697	3	*puuC*
0.583333333			
0	0.161512027	2	*pbpC*
1			
0	1	1	*kdpE*
0			
0	0.75	2	*amtB*
0.833333333			
6.94E-04	0.230392157	4	*argH*
0.607142857			
0.099865047	0.215469613	2	*fadA*
0.5			
0.091767881	0.189320388	5	*fepG*
0.48			
0	0.666666667	1	*hscA*
0			
0.001850139	0.259668508	3	*priC*
0.611111111			
0.051282051	0.163179916	5	*entF*
0.72			
0	1	1	*yjbE*
0	0.243523316	2	*serB*
0.611111111			
0	1	1	*yjbG*
0			
0.051282051	0.205263158	2	*ahr*
0.5			
0.666666667	0.8	3	*cysJ*
0.555555556			
0	0.220657277	1	*envC*
0			
0	1	1	*ygcN*
0			
0.666666667	1	3	*glnE*
0.666666667			
0	0.6	1	*ygiF*
0			
0	0.75	2	*glnG*
0	0	0	*ybdH*
0			
0	0	0	*cstA*
0	0.1625	4	*entA*
0.791666667			
0.04702436	0.321917808	7	*recR*
0	0	0	*insG*
0			
0	0.18875502	1	*zipA*
0			
0	0.1625	4	*entS*
0.537311132	0.385245902	6	*dnaX*
0.303030303			
0.091767881	0.189320388	5	*fepD*

0.48			
0	0	0	*ybbJ*
1	1	2	*cueR*
0	0.666666667	1	*copA*
0			
0.666666667	1	3	*thiF*
0.666666667			
0.004498426	0.202072539	4	*gabD*
0.454545455			
0.264569843	0.281437126	4	*amiB*
0.285714286			
0.070850202	0.1875	5	*fecC*
0.511111111			
0	0.75	2	*thiE*
0.833333333			
0.439496176	0.239263804	7	*gabT*
0.273809524			
0	0.571428571	2	*cysA*
0.833333333			
0	0.571428571	2	*cysW*
0.833333333			
0.042553191	0.236180905	2	*hemE*
0.5			
0	0	0	*chaC*
0			
0	0.148854962	1	*yihP*
0	0	0	*yphD*
0	0.191836735	1	*nfi*
0			
0.051282051	0.174107143	4	*yihQ*
0.5			
0.070850202	0.1875	5	*fecD*
0.511111111			
0	0.270114943	2	*nnr*
0.583333333			
0	0.75	2	*thiG*
0.833333333			
0	0.195	2	*lhgO*
0.785714286			
0	0	0	*yqjH*
0			
0	1	2	*b0259*
0	0.224137931	4	*eutK*
0.785714286			
0	0	0	*narJ*
0	0	0	*yhgF*
0			
0	1	1	*rhsD*
0			
0.020814061	0.191056911	3	*ampH*
0.555555556			
0	1	1	*ybbP*
0			
0.018655566	0.29375	3	*ssb*
0.188933873	0.205263158	6	*yihU*
0.361111111			
0	0.173333333	3	*yihV*
0.619047619			
0.5	0.666666667	2	*ssuD*
0.5			
0.003854456	0.246073298	3	*sdaB*
0.481481481			
0	0	0	*yhjE*
0.001079248	0.264044944	3	*ygdG*
0.555555556			
0	0.18875502	1	*dedD*
0	0.444444444	1	*ssuB*
0			
0.633132902	0.379032258	8	*purL*
0.196428571			
0	0.160493827	4	*fecR*
0.9			
0.083256244	0.206140351	3	*yjhG*
0.444444444			
0	0.173333333	3	*yihT*
0.619047619			
0.393308665	0.345588235	10	*mutL*
0.3			
0	0.160493827	4	*fecA*
0.9			
0	0.160493827	4	*fecB*
0.9			
0	0.172161172	2	*yagF*
0.833333333			
0	0	0	*hrpB*
0			
0	0.172161172	2	*yagE*
0.833333333			
0.285714286	0.583333333	2	*yagA*
0.5			
0	1	2	*b0022*

0	0	0	*yaiC*
0			
0	1	2	*b0021*
0.325624422	0.315436242	8	*ilvA*
0.258333333			
1	1	2	*bcsC*
0			
0	0.6	1	*moeA*
0.565452092	0.265306122	4	*acs*
0.25			
0.056120876	0.330985915	8	*recG*
0.375			
0	0.666666667	1	*bcsB*
0	0.666666667	1	*fabB*
0			
0	0	0	*frvB*
0			
0	0.75	2	*napG*
0	0	0	*rhsC*
0			
0	0.666666667	1	*yjcE*
0.050607287	0.245283019	5	*eutC*
0.6			
0	1	1	*rutF*
0			
0	0	0	*glgA*
0			
0.080481036	0.290123457	6	*carB*
0.393939394			
0	0.207446809	2	*fhuB*
0.642857143			
0	0	0	*nepI*
0	0.538461538	2	*dppC*
0.75			
0.116059379	0.253246753	6	*eutG*
0.520833333			
0	1	2	*uhpT*
0	1	1	*rutD*
0			
0.504723347	0.276595745	4	*eutD*
0.5			
0	1	2	*uhpB*
1			
0.016194332	0.25	5	*eutT*
0	1	2	*uhpA*
1			
0.166666667	1	3	*nrfD*
0.166666667	1	3	*nrfC*
0.777777778			
0.066666667	0.6	3	*ulaD*
0.666666667			
0	0.75	2	*nrfE*
0.333333333	0.6	3	*ulaF*
0.583333333			
0.023809524	0.583333333	3	*dppF*
0.666666667			
0	0	0	*yecS*
0			
0.431174089	0.276595745	6	*eutA*
0	0.229268293	3	*argF*
0.666666667			
0.456140351	0.220338983	6	*fhuC*
0.320512821			
0	0.388888889	1	*yagI*
0			
0	0.5	1	*yagH*
0			
0.666666667	0.75	2	*yagG*
1	1	2	*kefC*
0			
0	0.666666667	1	*kefF*
0			
0	0	0	*putA*
0			
0	0	0	*hflD*
0			
0	0	0	*ubiH*
0			
0.666666667	0.75	2	*paoB*
0.5			
0	0	0	*yiaD*
0			
0	0	0	*emrD*
0			
0	0	0	*yidF*
0			
0	0	0	*aegA*
0			
0	0	0	*insK*
0			
0	0	0	*yfiF*
0			
0.122109158	0.252688172	5	*ilvB*
0.44			

0	1	1	*fixB*
0			
0	0	0	*stfR*
0	0.5	1	*paoD*
0			
0	1	1	*mdtP*
0			
0	1	1	*mdtN*

C) PPI network for down-regulated genes in gentamicin-resistant cells

Number of nodes	Number of edges	Average node degree	Average local clustering coefficient	Expected number of edges	PPI enrichment p-value	Betweenness Centrality	Closeness Centrality	Degree	Display name
432	2630	12.2	0.498	930	< 1.0e-16	0	0	0	*paaB*
						0			
						0.01470548	0.218548387	3	*skp*
						9.17E-05	0.278520041	51	*ykgM*
						0.007380074	0.144379329	2	*ecnB*
						0	0.16035503	5	*hicA*
						0.011739861	0.315116279	71	*rpsJ*
						0	0	0	*ythA*
						0.001491126	0.2873807	49	*raiA*
						0	0	0	*ynfO*
						0.019944848	0.290149893	51	*hpf*
						0.720479303			
						0	0	0	*yrhB*
						0	0.18175721	2	*hokE*
						0	0	0	*yfaE*
						0	0	0	*ggt*
						0			
						0.020571946	0.241317898	4	*yhbJ*
						0.25877193			
						9.48E-04	0.203912716	2	*npr*
						0.5			
						0	1	1	*yecJ*
						0.00128833	0.294245385	68	*rplC*
						0	0	0	*ykgL*
						0.028756133	0.271543086	56	*frr*
						0.711309524			
						0.003101559	0.277096115	57	*tsf*
						0	0	0	*yciS*
						0	0	0	*ycdZ*
						0	0.224709784	5	*rof*
						0.48			
						0	0.207503828	1	*ribA*
						0			
						0	0.6	1	*yeeT*
						0.015061304	0.229466554	8	*ypfN*
						0	0	0	*ypeB*
						0	0	0	*bfd*
						0			
						0	0.6	1	*yeeX*
						0	0.6	1	*ypjJ*
						0	0	0	*yehK*
						0	0.6	1	*yciN*
						0			
						0.00874672	0.230638298	5	*fabZ*
						0.246875			
						0.001398741	0.213217939	3	*fabI*
						0.407407407			
						0	1	1	*ymgA*
						2.08E-04	0.172063492	3	*yibT*
						0	1	1	*ypdI*
						0.004379127	0.226778243	7	*yhhL*
						0.036463031	0.201486989	3	*yifL*
						0.033991077	0.180426099	9	*yefM*
						0.37254902			
						0	0	0	*slyX*
						0			
						0	0.167801858	1	*yhaL*
						0.009692802	0.169058016	2	*ghoT*
						0	0.263875365	49	*arfA*
						0.004873501	0.309360731	68	*rpsL*
						0	0	0	*yheV*
						0	1	1	*ycgZ*
						0	0	0	*csgC*
						0			
						0	0.194404591	1	*osmB*
						0	0.219255663	3	*yceO*
						0.009811757	0.171518987	3	*ycgJ*
						0.004873501	0.309360731	68	*rpsR*
						0.635620915			
						0	0.159693577	1	*yshB*
						1.66E-04	0.269116187	54	*priB*
						0	0	0	*yeiS*
						0	0.666666667	1	*fryB*
						0			
						0	0	0	*yjjZ*
						0.010260758	0.298129813	66	*rplI*
						0.640383426			
						0.021427069	0.247262774	7	*crp*
						0.255639098			
						0	0	0	*ytjA*
						0	0	0	*tdcR*
						0.017270244	0.233822261	6	*yqcG*
						0.005927958	0.31006865	70	*rpsI*
						0.007380074	0.1899089	2	*yqfG*
						0.003803632	0.29845815	70	*rplM*
						0.639880952			
						0.005499337	0.309714286	69	*rpsF*
						0.628312107			
						0	1	1	*cdd*
						0			
						0	0.170226131	1	*minE*
						0	0	0	*ymjA*
						0	0.126222636	1	*yceK*
						0.037523002	0.216973579	3	*hokA*
						0.016126579	0.234226448	6	*yfeD*

Hub genes																		
rpsJ	*rplV*	*rpsI*	*rplM*	*rpsK*	*rpmA*	*rplO*	*rpsQ*	*rpsF*	*rpsO*	*rpsD*	*rplU*	*rpsP*	*rpsT*	*rplE*	*rplX*	*rpsE*	*rpsH*	*rpsB*
rplN	*rpsC*	*rplB*	*rplC*	*rpsL*	*rpsR*	*rplQ*	*rpsM*	*rpsN*	*rpmF*	*rpoA*	*rpmB*	*rplR*	*rplF*	*rpsS*	*rplI*	*rpmJ*	*rpmD*	
rplW	*rpmC*	*rpsA*	*rpsU*	*rpmI*	*rplK*	*rplA*	*rplL*	*rpmG*	*rplY*	*rplT*	*rplJ*	*rpmH*	*secY*	*rpmE*	*tsf*	*frr*	*priB*	*smpB*
ykgO	*rpoZ*	*trmD*	*ykgM*	*hpf*	*rmf*	*rimM*	*raiA*	*arfA*	*yceD*	*infA*	*infC*	*sra*	*thrS*	*adk*	*acpP*	*rnpA*		

0.272727273			
0	1	2	*ytfK*
0	0	0	*yhfL*
2.45E-04	0.220863896	2	*yhfG*
0	0	0	*aaeX*
0.03811306	0.216108453	4	*grxB*
0.367647059			
0	0.18767313	2	*lpxC*
0.8			
0	0.666666667	1	*dinI*
0			
0.024455183	0.218548387	3	*bssS*
0.018513343	0.273737374	8	*nuoB*
0.001500758	0.241532977	5	*nuoA*
0.307692308			
0	0.192334989	2	*yqhH*
0.642857143			
0	0	0	*ylcI*
0	0	0	*yjdM*
0	0.177240026	2	*uspA*
0.75			
0	1	1	*ycgY*
0	0	0	*ymgE*
0.00414489	0.297475302	67	*rpmF*
0.001040908	0.273185484	48	*yceD*
0	0	0	*dam*
0			
0	0	0	*ygiZ*
0	0	0	*yneM*
0	1	1	*ygiN*
0			
0	0	0	*ymgJ*
0.045928093	0.204992436	8	*ymgI*
0	0	0	*yhdU*
0.015035478	0.179351423	3	*yfbV*
0.093009244	0.249539595	11	*yciZ*
0	0.138335886	1	*mepS*
0			
0	0.666666667	1	*yciY*
0	0.191926346	1	*yceQ*
0	0	0	*pyrL*
0	0.121907332	1	*yjdJ*
0			
0.007095661	0.230834753	7	*yejG*
0.007380074	0.195809249	2	*hupA*
0.5			
9.35E-04	0.292972973	64	*rplY*
0.690692204			
0	0	0	*yqiB*
0.011047724	0.175291074	3	*yejL*
0.444444444			
0.031289616	0.268849206	15	*acpP*
4.88E-06	0.248168498	8	*rbfA*
0.785			
0.015935507	0.241748439	4	*glgS*
0.277777778			
0.007380074	0.199411332	2	*aspA*
0.5			
0	0.6	1	*yqiC*
0.014732814	0.16044997	4	*nlpI*
0.375			
0.02175563	0.314385151	69	*rpsO*
0.593640073			
0	0	0	*ccmD*
0.002214219	0.296823658	66	*rpmJ*
0.003137252	0.297802198	68	*rplQ*
0.64966299			
0.043635597	0.300443459	67	*rpoA*
0.60358209			
0	1	1	*yegR*
0.031081824	0.314385151	69	*rpsD*
0.626409018			
0.047219862	0.270189432	11	*groS*
0.008048044	0.313657407	70	*rpsK*
0.004873501	0.309360731	68	*rpsM*
0.635620915			
0.004801413	0.234226448	2	*ihfB*
0.028675222	0.304152637	65	*rpsA*
0.614775324			
0.00493632	0.256385998	7	*frdC*
0.003803632	0.29845815	70	*rpmA*
0.005029696	0.308304892	65	*rpsU*
0.003231207	0.298129813	69	*rplU*
0	0	0	*sfsB*
0	0	0	*yhbY*
0			
0	0.175745785	1	*mcbA*
0	0	0	*ycbK*
0.00130775	0.2	2	*ybiJ*
0	0	0	*ycaR*
0	0	0	*yjbJ*
0			
0.007380074	0.172941927	3	*ibaG*
0	0.147523136	1	*mlaB*
0	0	0	*yecH*
0	1	1	*yecR*
0.007380074	0.179708223	2	*ompX*
0.5			
0	0	0	*glnH*
0			
0.058589565	0.279669763	8	*hfq*
0.221153846			
0	0.183604336	1	*yjeT*
0	0.20942813	2	*yecF*

0	0.1989721	2	*uvrY*
0.666666667			
0	0.208141321	1	*yqjC*
0.008866938	0.23402418	4	*bsmA*
0.043548262	0.257849667	10	*bssR*
0	1	1	*fliZ*
0.059585298	0.299116998	50	*rmf*
0	0	0	*yoaC*
0.057409956	0.278520041	12	*sdhC*
0	0	0	*yedF*
4.39E-04	0.225833333	5	*sucC*
0.470588235			
0.048502792	0.206554878	7	*grxA*
0.268907563			
0	0.177240026	2	*uspG*
0.75			
1	1	2	*tusE*
0.060189426	0.238556338	6	*ahpC*
0.246376812			
0	0.146486486	1	*yccX*
0			
0.026153657	0.245693563	9	*sdhD*
0.247863248			
0	0	0	*yoaB*
0			
0	0	0	*ydiT*
0	0	0	*ybjC*
0	0.666666667	1	*yccA*
0			
0	1	1	*mgrB*
0.009853097	0.239823009	5	*yobF*
0	0.20530303	1	*ybgE*
0.041079584	0.242831541	5	*cspC*
0.226666667			
0	0.666666667	1	*zapA*
0.063705268	0.263618677	11	*yodD*
0.006587698	0.223413026	3	*dsrB*
1.86E-06	0.253507951	23	*thrS*
0.861204013			
3.46E-04	0.268316832	46	*infC*
0.719833903			
0.001876563	0.293290043	65	*rpmI*
0.674441687			
0	0	0	*tatE*
0			
0.001067866	0.292972973	64	*rplT*
0.002392357	0.211388456	2	*yebW*
0.016259742	0.243049327	5	*yodC*
0	0	0	*cspH*
0.033989232	0.262342691	4	*yebV*
8.43E-05	0.199411332	2	*ihfA*
0.5			
0	0.228306655	2	*cspE*
0.590909091			
0.033017434	0.230246389	12	*holE*
0	1	1	*mdtJ*
0	0	0	*yobA*
0			
0	0	0	*ybeD*
0			
0.01407135	0.152247191	2	*ymcE*
0.099456024	0.275406504	8	*yqgB*
0	0.13085466	2	*cspG*
0.005926531	0.212216132	6	*ydgC*
1	1	2	*yebG*
0			
0	0.666666667	1	*yebF*
0.018527567	0.236062718	3	*yniA*
0.333333333			
0.08552118	0.262596899	9	*ydiZ*
0.01462955	0.255419416	7	*fumA*
0.275862069			
0	0.191114245	1	*ybeL*
0.05593091	0.249769585	9	*cedA*
0.245210728			
0.001494293	0.270729271	47	*infA*
0.060671388	0.258834766	11	*acrZ*
0.06258411	0.266208251	4	*cspD*
0.257936508			
0	0	0	*uidR*
0			
0	0.186510668	1	*chbB*
0			
0	0.218548387	1	*yajC*
0	0	0	*yqgC*
0	0	0	*ppiB*
0			
0	0	0	*gltI*
0			
0	1	1	*safA*
0	0.149064906	2	*cyaY*
0.75			
2.19E-05	0.160735469	7	*hipB*
0.595238095			
6.66E-04	0.196234613	2	*cnu*
0	0	0	*ygdR*
0.010935532	0.213050314	4	*rclB*
6.54E-04	0.291084855	60	*rpmE*
0	0	0	*cutC*
0			
0	0	0	*moaD*
1.67E-04	0.267786561	52	*trmD*
0.764932127			

4.18E-04	0.267258383	50	*rimM*
0.005499337	0.309714286	69	*rpsP*
0.628312107			
9.35E-04	0.234429066	4	*cyoD*
0	0	0	*mliC*
0.007380074	0.186767746	2	*lsrG*
0.5			
0	0	0	*yggX*
0			
0.00116154	0.292340885	62	*rpmH*
0	0	0	*ybiA*
0			
9.96E-06	0.247714808	14	*rnpA*
0.831766917			
0	0	0	*yneG*
0.022030887	0.168427595	3	*ydhI*
0	0.144225652	1	*slyA*
0			
0	0	0	*uxaB*
0			
0	0	0	*ygeI*
0	0	0	*ydhL*
0	0	0	*ybcW*
0.04170519	0.260076775	6	*sodB*
0.225490196			
0	0	0	*yfgG*
0.007380074	0.197810219	3	*yeaC*
0	0.138335886	1	*mepH*
1	1	2	*ftsB*
0.026958881	0.188063845	6	*grxD*
0.311111111			
0	1	1	*ygbE*
0.02219679	0.255178908	6	*marB*
0	1	1	*fldA*
0			
0.021667562	0.262596899	2	*marA*
0	1	1	*fur*
0			
0	0.163845224	1	*hupB*
0			
0	1	1	*flhD*
0			
0.003217911	0.208301307	5	*ydcA*
2.24E-05	0.269383698	54	*smpB*
0	1	1	*udp*
0			
0.019341885	0.246587807	3	*yahO*
0	0.666666667	1	*nlpD*
0	0	0	*yahM*
0	0.197665937	2	*msrB*
0	0.208782743	1	*ydeI*
0	0	0	*alpA*
0	0	0	*ybfA*
0	1	1	*ydhR*
0			
0.021654979	0.234429066	3	*pspF*
0.593137255			
0.010039133	0.261583012	4	*ndk*
0.305921053			
0	0	0	*ydfZ*
0	0	0	*mokB*
0	0.165243902	1	*mipA*
0	0	0	*ydfO*
0			
0.015091715	0.193019943	2	*pspB*
0	1	2	*ydcH*
0	0	0	*mntS*
0	0.16064019	6	*dinJ*
0	0	0	*yncJ*
0.007380074	0.130917874	3	*cspB*
0.5			
1	1	3	*yihD*
0			
0	0.23402418	2	*atpE*
2.19E-05	0.160735469	7	*yafQ*
0.595238095			
0	0.115811966	1	*essQ*
0	0.194683908	2	*lpp*
0	0	0	*ybaM*
0.067868724	0.268849206	12	*ydhZ*
0	0.188981869	1	*yoaG*
0.025634676	0.174951582	6	*iscU*
0	0	0	*yacG*
0.009685829	0.232817869	3	*yeaQ*
0	0	0	*lacA*
0			
0	0.666666667	1	*cspI*
1	1	2	*ynfN*
0	0	0	*yoaF*
0	0.177588467	2	*tomB*
0	0.170333124	2	*gnsB*
0	0	0	*yeaO*
0.001051983	0.186126374	4	*grxC*
0.416666667			
0	0	0	*queD*
0			
0	0	0	*rayT*
0			
1.37E-05	0.149146946	3	*iscX*
0.611111111			
0.051773596	0.212382445	7	*hha*
0.229813665			
0	0.199411332	3	*ybdJ*
0.059404034	0.262596899	10	*ychH*
0	1	1	*rbsD*
0			

0.001607387	0.205614568	2	*crr*
0.5			
0.001674193	0.210567211	3	*tpx*
0.533333333			
0	0.188850174	2	*ydcX*
0	0	0	*flxA*
0.007380074	0.138760881	2	*yafP*
0.5			
0.00959302	0.247714808	8	*ydiH*
0.01470548	0.160830861	7	*yafN*
0.032245464	0.176088369	9	*relE*
0.435897436			
0.043292711	0.276530612	19	*adk*
0.610411899			
0.026793152	0.18767313	3	*hokD*
0	0	0	*rem*
2.62E-04	0.151143335	3	*iscR*
0.619047619			
0.009478905	0.291084855	65	*rplK*
0.662684124			
0.003414116	0.2873807	65	*rplA*
0.688926458			
5.09E-04	0.286469345	63	*rplJ*
0.715343915			
0.006562821	0.296823658	65	*rplL*
0.028131782	0.229078614	5	*ptsH*
0.2			
0	0.186896552	2	*dicC*
0	0	0	*gpt*
0	0	0	*ybaB*
0.001434383	0.293926247	67	*rpmB*
0	0.202389843	4	*ydcD*
0	1	1	*yncH*
0.010856532	0.224709784	5	*ydfD*
0.003760048	0.191249118	4	*dicB*
0.036018931	0.215079365	4	*uspE*
0.321428571			
0.066118313	0.269116187	4	*ynaJ*
0.023511289	0.240461402	4	*ydfC*
0	0.1899089	1	*ybaQ*
0.043573111	0.263106796	5	*ydfB*
2.19E-05	0.160735469	7	*mazE*
0	1	1	*rbsB*
0			
0	0.218548387	1	*yphA*
0	0.666666667	1	*ychN*
0			
0	0.158017493	1	*yqaE*
1	1	3	*ykfF*
0			
0.001006571	0.293290043	65	*rpmG*
0.014732814	0.187543253	3	*chaB*
0.333333333			
0.010049609	0.224709784	5	*ydfA*
0.02264119	0.193848355	7	*ralR*
0.004920649	0.180666667	5	*ydaC*
0.001460699	0.235039029	6	*yaiY*
0.016571712	0.271	53	*rpoZ*
0.709433962			
0	1	1	*feoA*
0	0	0	*hinT*
0			
0	0	0	*ybbC*
0	0	0	*yaaI*
0.01541767	0.201038576	6	*ynfB*
0	0.16635973	1	*panD*
0	0	0	*nrdH*
0			
0	0.163154726	1	*glnB*
0.007380074	0.194823868	2	*glrR*
0.5			
0	0.162470024	1	*dinQ*
0	1	1	*yiiE*
0.124402636	0.304152637	54	*ykgO*
0.019140523	0.21473851	3	*hns*
0	1	1	*mdtI*
0	0.208141321	1	*yaiA*
0			
0	0	0	*yddM*
0.044614268	0.228306655	9	*racR*
0.256038647			
0.007380074	0.237302977	2	*yaaY*
0	0	0	*asr*
0.001080651	0.236888112	3	*dksA*
0.04053151	0.235652174	5	*ydaG*
0.027278989	0.31006865	69	*rpsT*
0.005749804	0.221767594	3	*bhsA*
0	0	0	*ynfM*
0.005670717	0.192062367	6	*kilR*
0	1	1	*feoC*
0	0	0	*torI*
0	0	0	*ppiC*
0			
0	0	0	*yjbS*
0	0.157466589	1	*luxS*
0			
0	1	1	*yeiW*
0	0	0	*yhjR*
0	0.6	1	*ykfH*
0	1	1	*yiiF*
0.009530236	0.210077519	7	*racC*
0.002632424	0.222861842	4	*iraP*
0.032278523	0.255901794	7	*yaiZ*
0.004873501	0.309360731	68	*rpsN*

0	0.158017493	1	*ymdF*
0.012172734	0.301781737	69	*rplE*
0	0	0	*yciU*
0.019393018	0.17394095	3	*gnsA*
0.010023227	0.307256236	69	*rplX*
0.022847691	0.163056558	4	*erpA*
0.375			
0	0	0	*ynaK*
0.018488974	0.279381443	61	*secY*
0	0	0	*ybdD*
0.004773067	0.301111111	70	*rplO*
0.62755102			
0.00445129	0.223045267	5	*csrA*
0.285714286			
0.001052103	0.293607801	66	*rpmD*
0.685239492			
0.005295289	0.309714286	69	*rpsE*
0.62962963			
0.001114846	0.293926247	67	*rplR*
0.682233991			
0	0	0	*grcA*
0.001114846	0.293926247	67	*rplF*
0.007582013	0.313294798	69	*rpsH*
0.616587746			
0.02686406	0.258095238	6	*cydX*
0	0.198389458	1	*rcsB*
0			
0.001858967	0.197378004	4	*trxA*
0.475			
0	0	0	*rseA*
0.007380074	0.241103203	3	*osmC*
0	0.233218589	1	*bdm*
0.166483751	0.303811659	29	*sra*
0.001052103	0.293607801	66	*rplW*
0.02197622	0.22985581	3	*yciG*
0	0	0	*rzoR*
0.010159009	0.314385151	69	*rpsB*
0.615439805			
0.020816066	0.248395967	5	*icd*
0.286666667			
0.007380074	0.216973579	3	*blr*
0	0.152418448	1	*ompW*
0			
0	0	0	*ykgJ*
0.002581731	0.297802198	69	*rplN*
0.654614798			
0.006166341	0.211223694	2	*glgX*
0.5			
0	0.178406847	1	*ymgF*
0	0.236888112	1	*yfcZ*
0.015043829	0.318075117	70	*rpsQ*
0.002218962	0.296823658	66	*rpmC*
0	0	0	*yfiM*
0	0	0	*yjjY*
1	1	2	*yciX*
0	0.666666667	1	*ymiA*
0.007582013	0.313294798	69	*rpsC*
0.616587746			
0.026755236	0.150305047	4	*cspA*
0.012274995	0.304836895	71	*rplV*
0.00474611	0.309007982	67	*rpsS*
0.638022011			
4.41E-04	0.205614568	4	*ydaF*
0.003305415	0.195809249	5	*ydaE*
0.002581731	0.297802198	69	*rplB*
0.001063276	0.197234352	3	*trxC*
0.533333333			
0	0	0	*eutS*
0	1	2	*yaeH*
1			
0	0	0	*yagN*
